# Bootstrap Synthesis Revisited: A Solid, Cobalt-Derived Catalyst Enables the Hydrogenation of N=N Bonds to Afford Anilines

**DOI:** 10.1021/acsomega.6c03689

**Published:** 2026-08-04

**Authors:** Fabian Schmiedbauer, Jiri Duchoslav, Laura Zellner, Christoph Topf

**Affiliations:** † Institute of Inorganic Chemistry, 27266 Johannes Kepler University (JKU), Linz 4040, Austria; ‡ Center of Surface and Nanoanalytics (ZONA), Johannes Kepler University (JKU), Linz 4040, Austria; § Institute of Analytical and General Chemistry, Johannes Kepler University (JKU), Linz 4040, Austria; ∥ Institute of Catalysis (INCA), Johannes Kepler University (JKU), Linz 4040, Austria

## Abstract

We communicate a solvent-free and low-waste method for the fabrication of a heterogeneous, Co-based hydrogenation catalyst. Careful pyrolysis of a solid [Co­(acac)_2_]/phthalocyanine/Vulcan mixture (acac = acetylacetonate) under an argon atmosphere furnishes a ready-to-use, catalytically active material that can be handled without any protection from air. The composite so obtained was then used in the pressure hydrogenation of azo dyes (60 °C, 50 bar H_2_) to yield the corresponding aniline derivatives, without any additive. The potential of the catalyst to design a bootstrap synthesis approach of aromatic amines, i.e., their generation from within themselves, by way of reduction of symmetric azo compounds is explored herein, too. To further demonstrate the practicality of the introduced catalyst, we also applied it to the hydrogenation of nitroarenes as another means for the production of arylamines. In all investigated cases, the NO_2_ motif was neatly and selectively converted into the desired NH_2_ group under very mild reaction conditions (room temperature, 20 bar H_2_) and, again, without the need for any auxiliary reagents.

## Graphical abstract

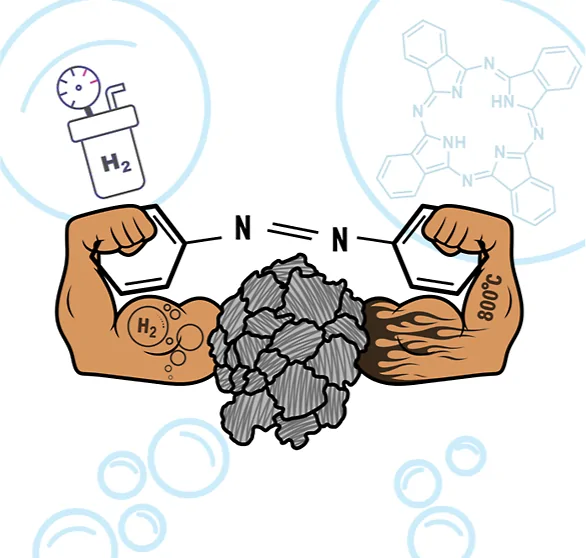

## Introduction

The catalytic (transfer) hydrogenation of azo compounds is a worthwhile chemical transformation as it enables the syntheses of valuable organic products in an atom-economical way. In particular, aromatic amines, which rank among the most important bulk chemicals,^1,2^ are accessible through this process.

The homogeneous azo-to-amine reduction is, for instance, possible with H_2_ and well-defined Ru^3^ or Mn^4^ complexes that incorporate phosphine donor moieties. Moreover, Mo_3_S_4_ cluster catalysis deploying either silanes^5^ or gaseous hydrogen^6^ has been described to bring about this reaction. Additionally, to decolorize dye baths, an electrochemical method for the degradation of azo compounds was devised.^7^

Yet, regarding recyclability as well as product separation, heterogeneous catalysis is the preferred practice for subject transformation, whereby a corresponding procedure relying on Raney Ni and H_2_ was already published in 1940;^8^ a more recent approach uses NaBH_4_ in combination with Si-supported Ru nanoparticles,^9^ while photochemical methods with solid Pd^10^ or Zn^11^ catalysts have also been invented. Furthermore, Co is apt for being worked into various heterogeneous formulations, in particular supported and pyrolytically activated complexes that contain this abundant 3d metal. In this context, composites prepared from Co^II^(phenanthroline) and Co^II^(melamine) precursors were shown to promote the reduction of N=N bonds in the presence of hydrogen transfer reagents such as alcohols^12,13^ or hydrazine hydrate (N_2_H_4_·H_2_O),^14^ albeit with the aid of base additives.

Typically, the pyrolytic syntheses of solid catalysts consist of multistep sequences that entail a separate complexation step in the liquid phase, wet impregnation of a carrier material, solvent removal, and final annealing of the loaded support. Hence, in terms of time efficiency as well as energy consumption, the whole procedure is not ideal and leaves room for improvement. In the given report, we present a streamlined way for the fabrication of a Co-derived composite catalyst that combines precursor formation and support impregnation into one step, but without the use of any solvent. Therefore, the time-consuming and energy-intensive drying period was eliminated. For the reduction of azo compounds, we chose H_2_ gas as the stoichiometric reagent because of its unexcelled atom efficiency (100%), which eventually translates into minimum waste generation.^15^ Strikingly, our catalyst is also self-contained in that it facilitates hydrogen transfer without the need for any additives (vide supra). In order to achieve this goal, the deployment of the nitrogen-rich phthalocyanine scaffold in the catalyst synthesis proved to be key.

Noteworthy, the reductive cleavage of symmetric Ar–N=N–Ar motifs can be recast as bootstrap syntheses for arylamines, meaning that aniline derivatives are essentially prepared from themselves.^16^ The feasibility of this approach was demonstrated in a late 19th century patent that delineates the manufacture of phenetidine,^17^ a direct antecedent of the analgesic phenacetin. The process contains three stages, i.e., (1) diazotation of phenetidine (1 equiv), (2) electrophilic, aromatic substitution on phenetole using the formed diazonium salt, and (3) reduction of the resultant azo dye to afford phenetidine (2 equiv). Thus, one iteration of steps (1)–(3) releases a net amount of 1 equiv of the target aniline. However, since the last stage is not atom-efficient and environmentally benign as it deploys excess tin metal and aqueous HCl solution, a newer protocol relies on catalytic hydrogenation using either Pd/C or Raney Ni.^18^ Yet, herein, we offer a more robust and easily implemented method that avoids the issues associated with these metals, i.e., high price (Pd) as well as safety risks and selectivity problems due to exuberant reactivity (Ni).^19,20^ As an aside, the bootstrap synthesis of methanol (MeOH), which includes the carbonylation of MeOH (1 equiv) to afford methyl formate and subsequent hydrogenation of this ester to MeOH (2 equiv), may serve as another illustrative example for the generation of a molecule from within itself.^21^

To expand upon the utility of our catalyst, we also applied it to the hydrogenation of nitroarenes as this is another convenient and brisk route to various aniline derivatives. Although occasional reports on homogeneous catalysis have appeared in the past (selected examples describe the use of Ru,^22^ Fe,^23^ Co,^24,25^ Cu,^24^ and Mn^26^), the vast majority of strategies for effective and practical nitroarene-to-aniline conversions revolves around heterogeneous approaches including, for example, Fe,^27,28^ Ni,^29^ and Cu.^30^ Among the first row transition metals, Co has long been known to catalyze this transformation as well,^31^ and this element still figures very prominently in the design of thermally activated (transfer) hydrogenation catalysts. Again, Co^II^(phenanthroline) complexes^32–36^ turned out to be proper solution phase precursors while bisamidinate,^37^ melamine/polyacrylonitrile,^38^ cucurbit[6]­uril,^39^ and 2-methylimidazol^40^ allow for the rational design of functional composite materials. Strikingly, even shrimp chitosan can serve as a platform for making biobased, catalytically active materials.^41^ Yet, due to their beneficial N content (vide infra), phthalocyanine-derived coordination compounds are almost predestined for crafting solid, multicomponent catalysts, and related carbonaceous, Co-loaded assemblies that affect the prescribed target transformation were previously reported.^42,43^ However, the preparation of pertinent materials involves handling of toxic HF solution which necessitates taking meticulous lab-technical precautions. In addition to using metal complexes as precursors, MOF-augmented Co catalysts were developed, too^44–46^ and, finally, Co may also function as a dopant to ameliorate the hydrogenation capabilities of MoS_2_.^47^

Notwithstanding these achievements on base-metal-catalyzed (transfer) hydrogenations of nitro compounds, reports on non-noble catalysts that operate under room-temperature and additive-free conditions are still very rare. Yet, the cobalt catalyst that we describe later in the text achieves exactly this goal.

## Results and Discussion

### Catalyst Preparation and Characterization

Phthalocyanine (H_2_Phc), [Co­(acac)_2_], and Vulcan XC 72R (all solid) were blended together and then pestled in a mortar made of agate. The triturated mixture was transferred into a ceramic crucible and then pyrolyzed under a protective atmosphere (800 °C, Ar, 2 h) to obtain the full-fledged catalyst (Scheme 1). Apart from its very high complex formation constant, we opted for the addition of the H_2_Phc ligand on the basis of its intrinsically high nitrogen content and, as shown earlier, increased N-doping aids in the catalytic activity.^48^ Indeed, the material described herein had an almost ten times higher nitrogen content compared to a congeneric composite that was previously published by us^49^ and which is based on a N_2_O_4_ salen-type chelator (1.57% vs 0.18%); the bulk elemental analysis of the present catalyst further revealed 2.90% Co, 95.41% C, and 0.08% H.

**Scheme 1:**
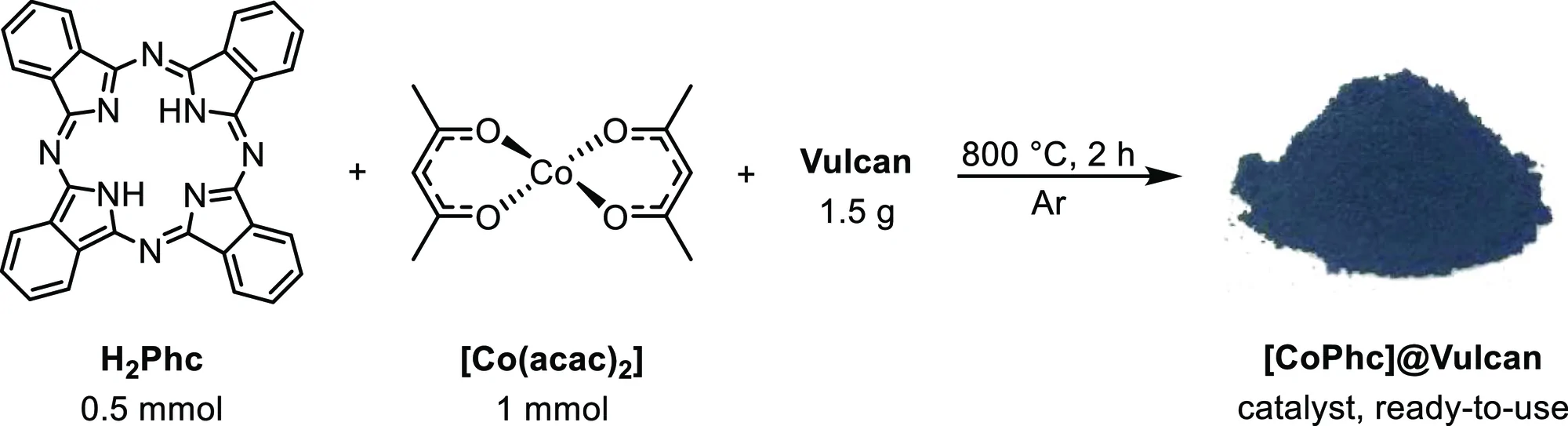
Pyrolytic, Solvent-Free Synthesis of Air-Stable **[CoPhc]@Vulcan**

The results of the XPS measurements (Figure 1) are in good accord with those obtained from the bulk elemental analysis. The 1s signal for nitrogen in the 396–406 eV range contains two major fractions of pyridinic and pyrrolic N that are accompanied by a smaller one which is due to graphitic N.^50^ Concerning cobalt, the asymmetric 2p_3/2_ signal at 780.5 eV and the 2p_1/2_ peak at 796.0 eV are a strong indication that an oxide species is present. Furthermore, if the peak at 803.0 eV is taken into account, it can be concluded that cobalt is largely present as Co_3_O_4_; in addition, the shoulder at 779.0 eV is attributable to metallic Co that had been formed during the pyrolysis process.^51^ A complete list of XPS graphs (including a plot containing the carbon 1s signal^52^) which were recorded in the course of this work is given in the Supporting Information (Figures S1–S6).

**Figure 1:**
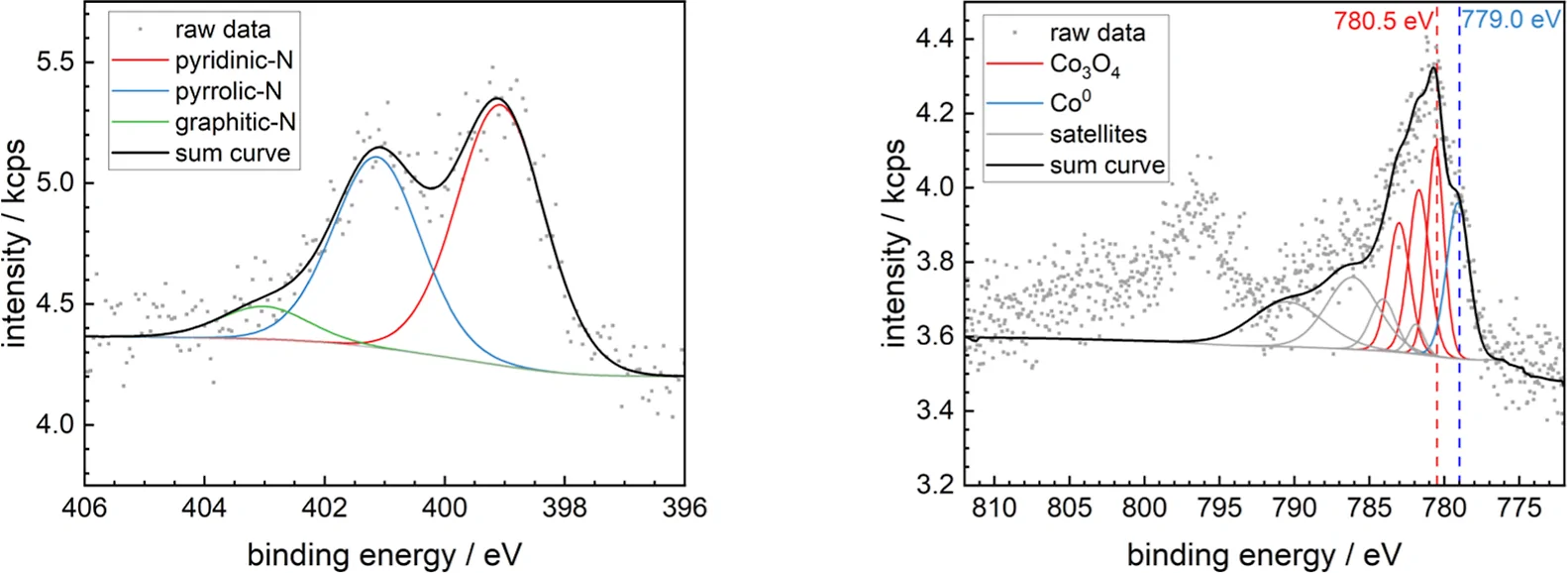
XPS analysis of the N 1s and Co 2p regions of solid **[CoPhc]@Vulcan**.

Closer spectroscopic and microscopic examination of the surface structure revealed the presence of powdery aggregates together with sprinkles of fibrous structures (Figure 2a–c). The illuminated regions pertain to the powder region and show a relatively low Co content, which does not exceed 0.5%. By contrast, the fibers feature a significantly higher concentration of the metal and are the only sites at which N was detected. Hence, it is conceivable that these peculiar entities are the locus where actual catalytic transformations take place.

**Figure 2:**
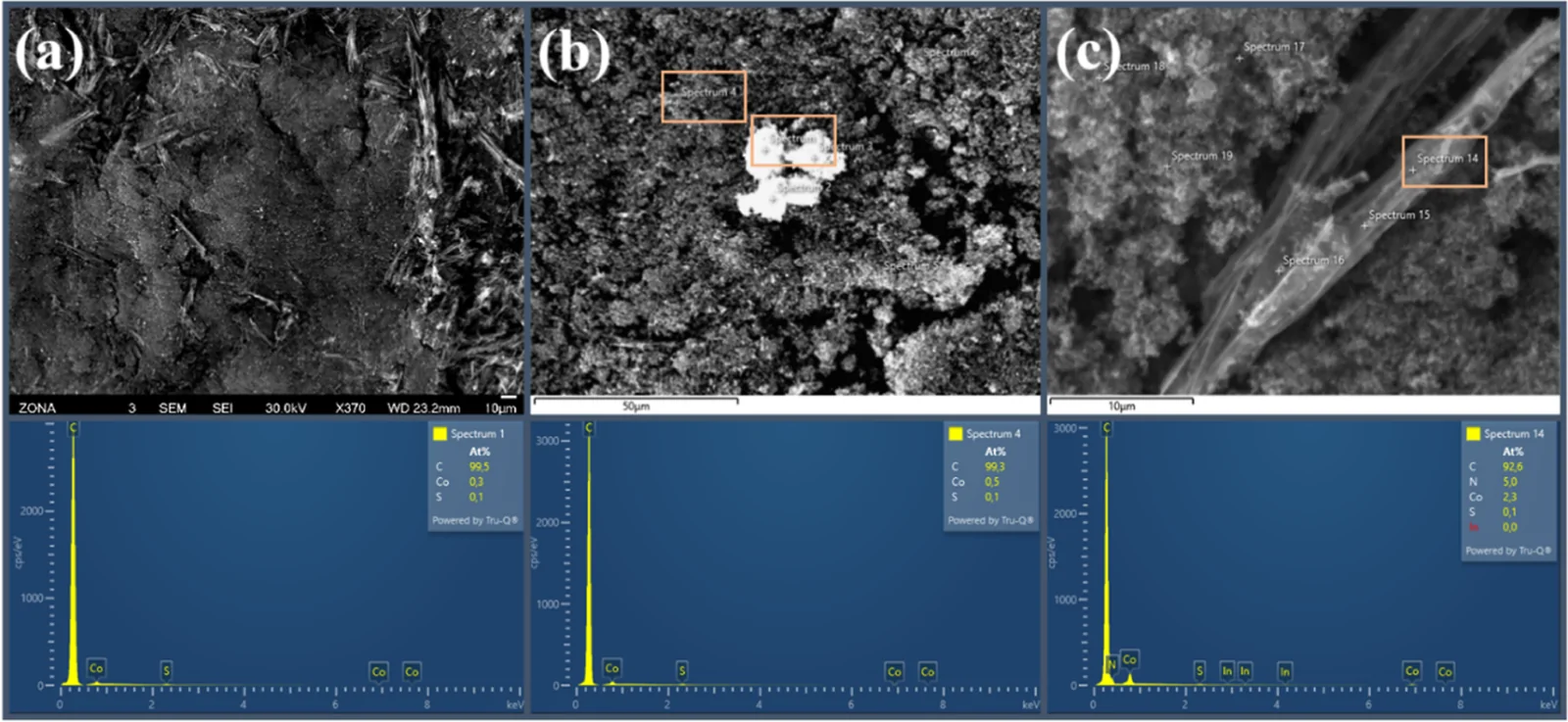
(a–c) Secondary electron images (a–c) of **[CoPhc]@Vulcan** combined with EDX spectra of the indicated excerpts (Spectrum 1, 4, 14).

### Initial Catalytic Tests

Guided by previous reports,^53–56^ we first set out for finding the optimal support and tested various thermoresistant oxides (SiO_2_, Al_2_O_3_, TiO_2_, and CeO_2_) as well as carbonaceous materials (Vulcan, graphite) as carrier materials in the Co-catalyzed hydrogenation of azobenzene **1a** to aniline **2a**, with Vulcan powder proving superior (Table S2). Next, we sought to identify the ideal solvent for the same reaction and established, as shown in Scheme 2, that polar alcohols (MeOH, 100% yield) heavily outperformed ethers (Et_2_O, 72% yield) and nonpolar hydrocarbons (*n*-heptane, 22% yield). Rewardingly, with MeOH, EtOH, and *i*-PrOH, we also achieved decent results at low temperatures (40 °C, 80–90% yield).

**Scheme 2:**
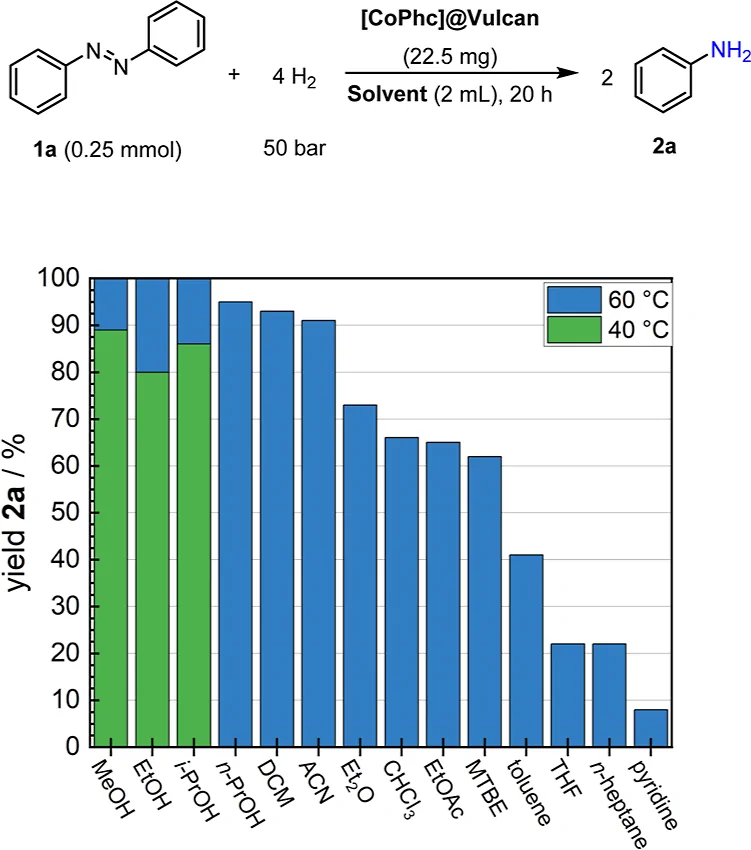
Solvent Variation Study at Different Temperatures

Then, we checked for the recyclability of our material and performed five consecutive reactions **1a** → **2a** with one individual catalyst batch (60 °C, 50 bar, 16 h). Notably, the catalyst performance was impeccable after two iterations (100% yield) but started to deteriorate after the third run (92% yield) and finally reached 70% of its original value after the last cycle (Figure S8). This decline in activity is attributable to Co loss due to chemical leaching and/or mechanical abrasion caused by stirring (2.34% Co in the used catalyst compared to 2.90% of the metal in the fresh one). Eventually, we conducted Maitlis’ hot filtration test to demonstrate the existence of an authentic, heterogeneous reaction pathway (Figure S10).^57^

### Scope and Limitations

According to GCMS analyses, the catalytic hydrogenation of parent azobenzene **1a** to aniline **2a** proceeded quantitatively with our Co catalyst, and treatment of the crude product with etheric HCl in DCM gave the solid hydrochloride salt in 88% yield. After that, we investigated different substitution patterns on the phenyl core (Table 1). Putting a methyl group in either position (**1b–e**) caused no major difficulties, and the corresponding ammonium salts were isolated in very good quantities (77–91%). However, increasing steric bulk ortho to the N=N group (**1f**) drastically worsened the reaction outcome (19%); yet, the system could cope well with the presence of the more flat and rigid Ph motif in **1g** (74%). Halides were tolerated throughout, although the yield was diminished in the case of iodo-substituted **1n** (52%), presumably due to detrimental hydrodehalogenation that took place as a side reaction. Moreover, strongly donating MeO groups (**1o–q**) did not obstruct the catalytic transformation in any way, and, rewardingly, it was also possible to reduce ester-functionalized **1s** in a selective manner to obtain 60% of the desired product.

**Table 1:**
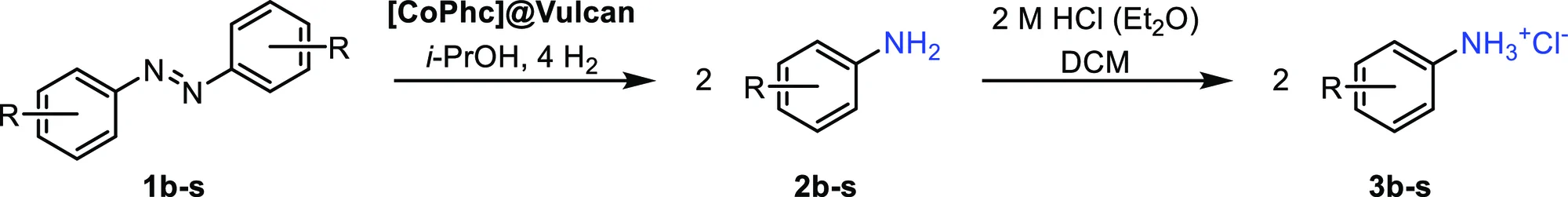

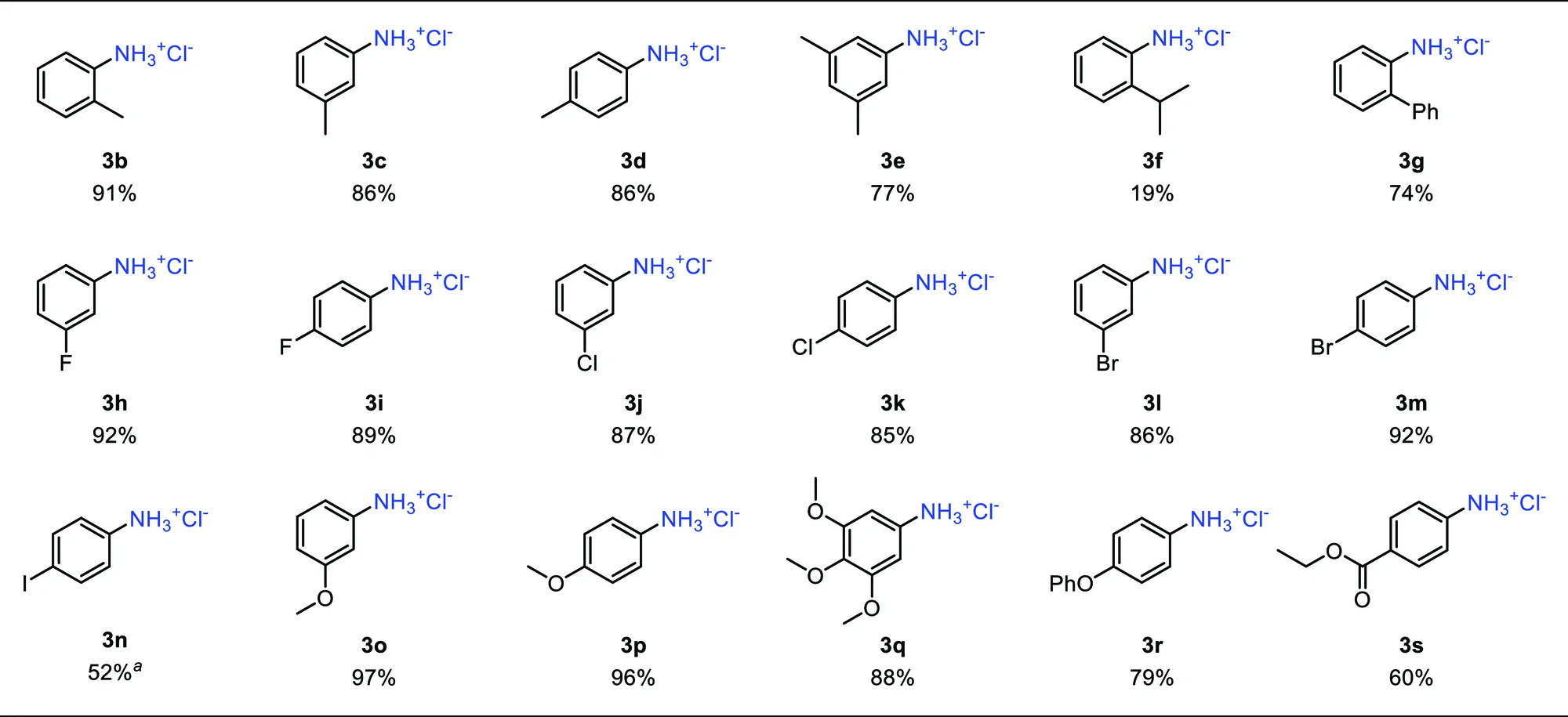
Functional Group Tolerance Survey for the Hydrogenation of Symmetrically Substituted Azobenzenes over **[CoPhc]@Vulcan** ^b^

a The substrate was purified by flash chromatography prior to precipitation.

b Reaction conditions: substrate (0.25–0.5 mmol), *i*-PrOH (2 mL), **[CoPhc]@Vulcan** (20–40 mg), 60 °C, H_2_ (50 bar), and 16 h. Percentages refer to isolated yields of the hydrochloride salt.

We then probed unsymmetrical substrates (Table 2) and were able to reproduce decent yields regarding methyl-substituted dyes **1t–v**; equally good results were achieved with electron-donating groups (**1w**) as with electron-withdrawing groups (**1x**), and NR_2_ functionalities (R: H, Me, or CH_3_CO) were also neatly converted into the respective anilines without loss of selectivity (**1y–aa**). On the other hand, the yields were somewhat diminished with a cyano (**2ac**) or an acetyl group (**2ad**) being present, although the outcome in the case of the benzoyl-tagged substrate was again very satisfactory (**2ae**). Strikingly, even sulfur-containing substrates such as **1af**, which are infamous for their ability to function as strong catalysts poisons, were flawlessly converted into amine **2af**. However, the presence of NO_2_ substituents like in the more elaborated, commercial Eriochrome Black T **1ag** heavily obstructed the azo hydrogenation process. This is a remarkable observation given the fact that **[CoPhc]@Vulcan** is a very effective catalyst for the reduction of nitroarenes that are devoid of the N=N motif (vide infra).

**Table 2:**
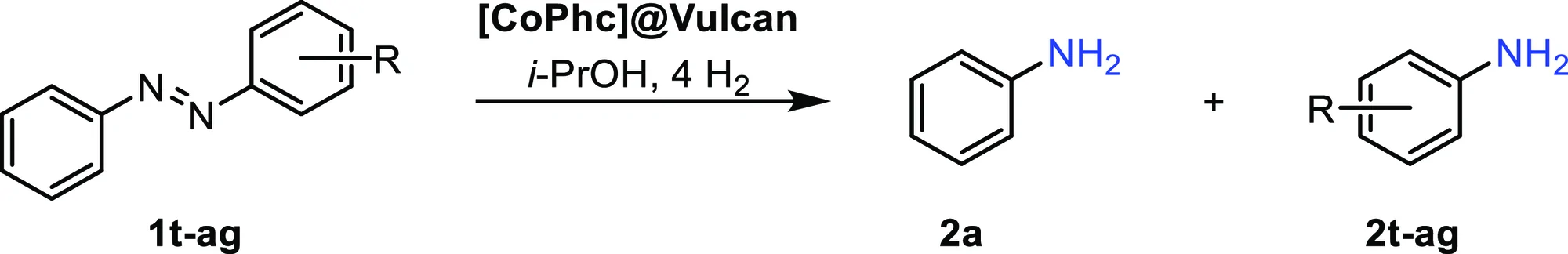

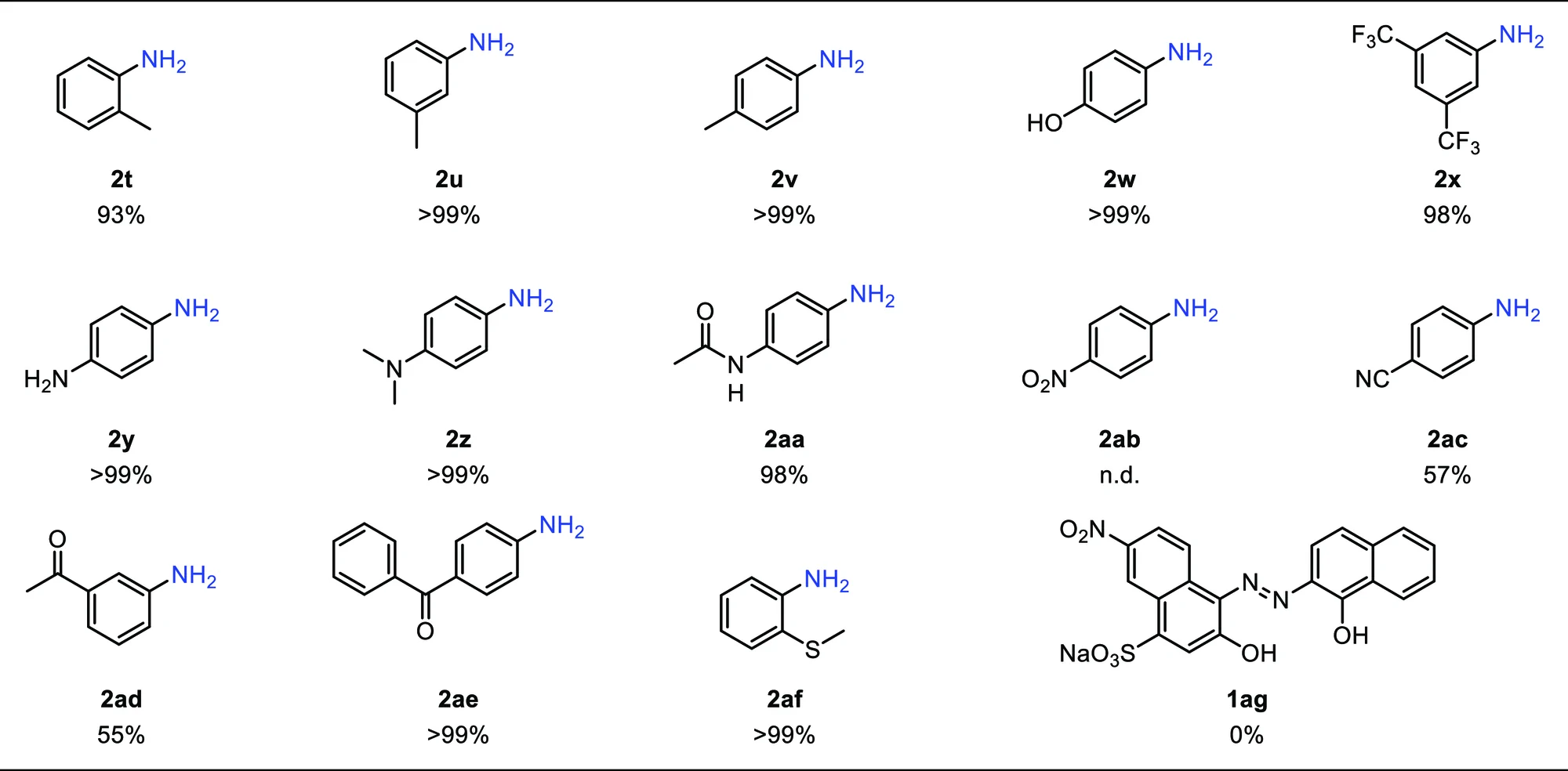
Catalytic Hydrogenation of Unsymmetrically Substituted Azobenzenes over **[CoPhc]@Vulcan** ^a^

a Reaction conditions: substrate (0.25–0.5 mmol), *i*-PrOH (2 mL), **[CoPhc]@Vulcan** (20–40 mg), 60 °C, H_2_ (50 bar), and 16 h. Yields were determined by GCMS analysis using *n*-hexadecane as an internal standard and referencing to equimolar byproduct **2a** (the GCMS spectra can be found in the Supporting Information).

To round off the topic of catalytic R–N=N–R reduction, we finally elaborated on the bootstrap synthesis of *p*-phenetidine **2ah**.^17^ Diazotation of this arylamine and subsequent reaction with phenolate afforded the corresponding azo dye **4ah** which upon etherification gave substrate **1ah** (82% yield).^16^ Reduction of the latter over **[CoPhc]@Vulcan** and under an atmosphere of H_2_ gave back two equivalents of **2ah** which was isolated as its hydrochloride salt **3ah** in 95% yield (Scheme 3). Hence, if the described reaction sequence is completed once, it will always produce one net equivalent of *p*-phenetidine per iteration. As mentioned earlier, our Co catalyst circumvents the problems that come with the use of Raney Ni and Pd/C, namely, pyrophoricity and high price of the noble metal.^18^

**Scheme 3:**
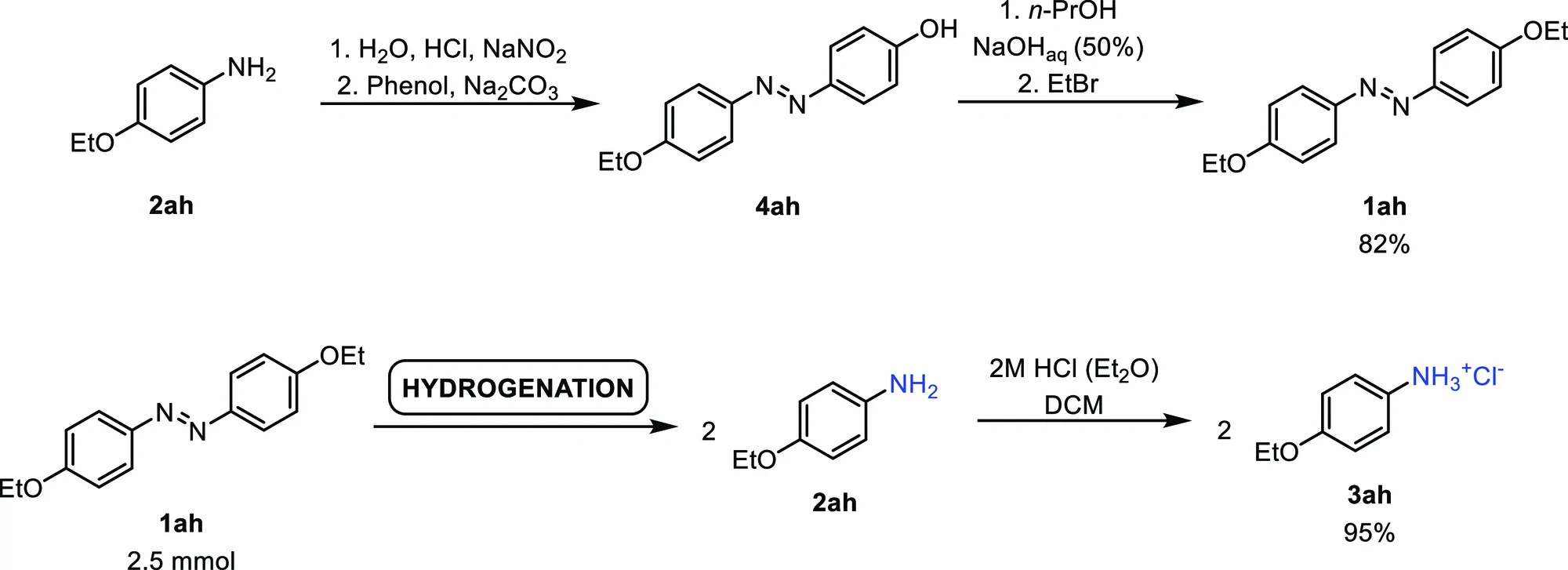
Bootstrap Synthesis of *p*-Phenetidine^a^

a Hydrogenation protocol: **1ah** (2.5 mmol), *i*-PrOH (20 mL), **[CoPhc]@Vulcan** (200 mg), 60 °C, H_2_ (50 bar), and 24 h.

Eventually, we turned our attention to the heterogeneous hydrogenation of nitroarenes as this process provides low-waste and quick access to a wide array of anilines, too (Table 3). In this context, a remarkable feat of **[CoPhc]@Vulcan** is its capability to promote this reaction at room temperature. Parent compound **5a** as well as its methylated and phenyl-tagged congeners **5b–e** produced medium to very good yields (54–92%). Again, halide-tagged nitrobenzenes did not compromise catalysis and gave proper results throughout (67–94%); in the case of electron-releasing groups such as methoxy **5n–o** or hydroxy **5p–q**, we found a preference for the meta derivatives. Chemoselectivity was achieved for ketone **5u–v**, nitrile **5w**, and ester **5z** functionalities. In this connection, a further striking example was the hydrogenation of cinnamic acid derivative **5y** which left the COOH and the conjugated C=C bond untouched to produce the desired salt in 98% yield. Interestingly, the presence of a simpler aldehyde group effectively forestalled the isolation of tagged ammonium salts such as **7t**, presumably owing to imine condensation reactions that led to ill-defined, high-molecular-weight side products. Moreover, alkynes (**5x**) and carboxylic acid chlorides (**5aa**) proved to be problematic, too. With the former, we observed (semi)­hydrogenation of the C≡C bond and no conversion of the nitro group, while in the latter case, total esterification into isopropyl carboxylate, but still, along with complete reduction of the NO_2_ motif occurred. Yet, more challenging substrates, especially heterocycle **5ac**, were hydrogenated into the projected amine (24% yield), whereby the yield could be readily ameliorated on raising the reaction temperature.

**Table 3:**
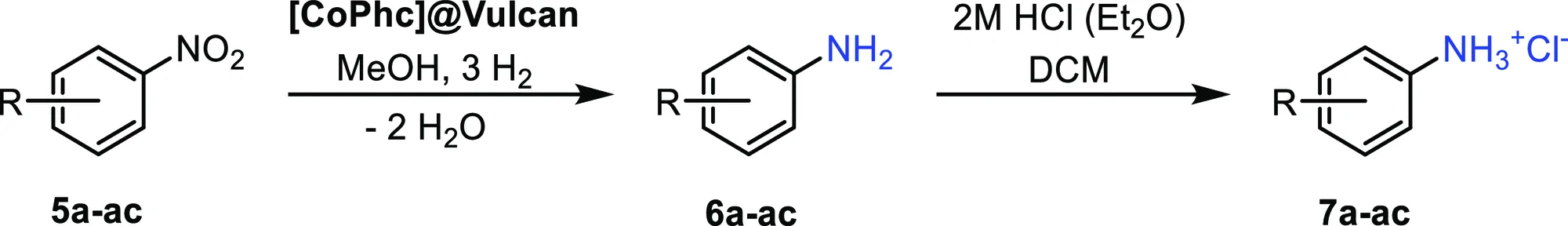

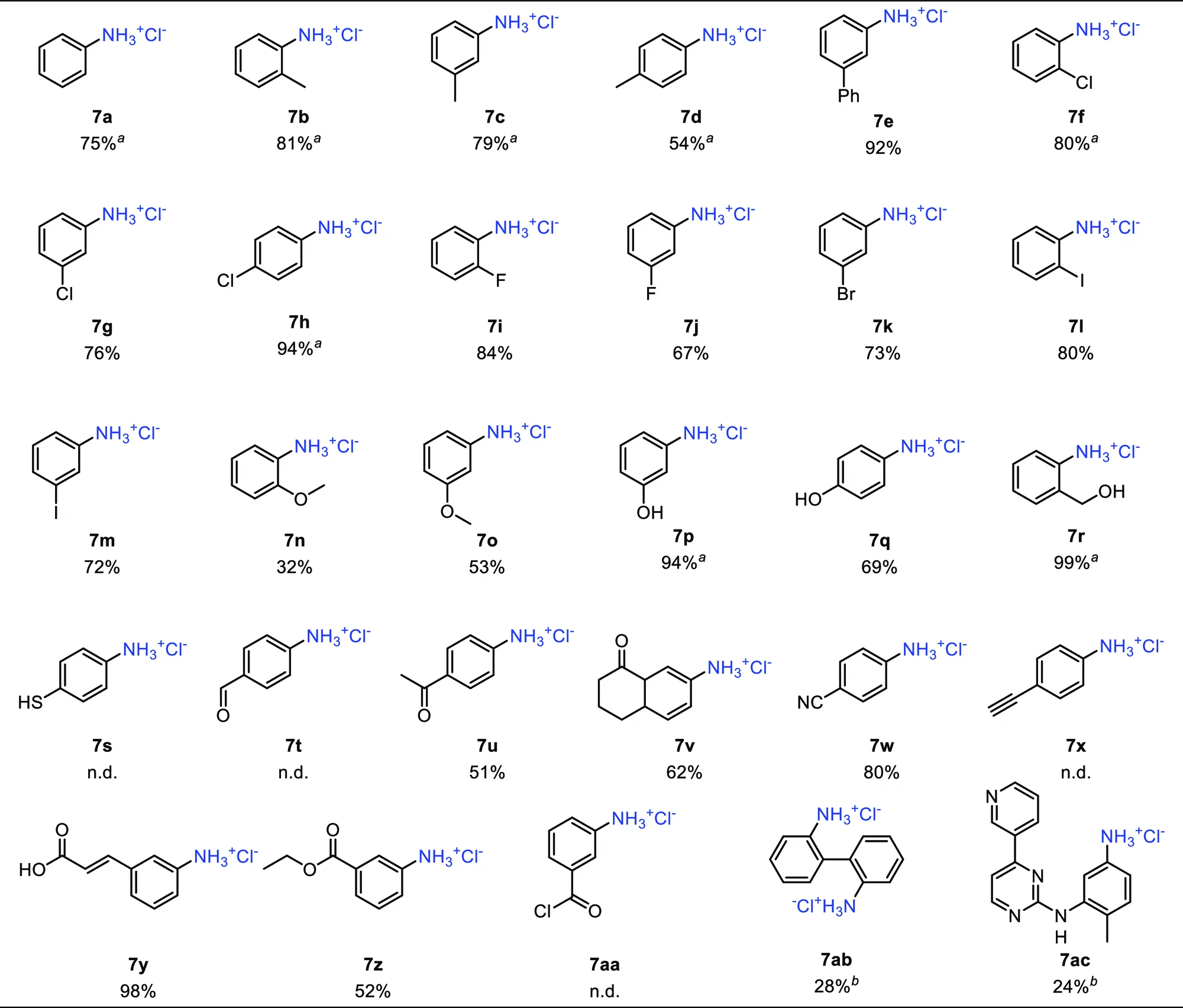
Catalytic Hydrogenation of Substituted Nitroarenes over **[CoPhc]@Vulcan** ^c^

a Complete conversion was observed according to GCMS.

b The substrate was purified by flash chromatography prior to precipitation.

c Reaction conditions: substrate (0.25–0.5 mmol), MeOH (2 mL), **[CoPhc]@Vulcan** (10–20 mg), R.T., H_2_ (20 bar), and 16 h. Percentages refer to isolated yields of the hydrochloride salt.

Since **[CoPhc]@Vulcan** worked well in the discontinuous pressure hydrogenation of azo dyes and nitroarenes, we also tested its aptitude for continuous flow mode using ThalesNano’s H-Cube Mini Plus reactor. Regrettably, the fine powdery nature of the catalyst produced such a high flow resistance that it was impossible to pump the reactions solution through the catalyst cartridge. Our future work therefore includes the development of coarse-grained formulations that are supposed to allow for continuous operation.

## Experimental Section

### Materials and Methods

If not otherwise, chemicals and solvents were purchased from commercial suppliers (Acros Organics, Alfa Aesar, BLDpharm, Fluorochem, Macherey-Nagel, Merck, or TCI). Procedural details for own-synthesized compounds can be found either in the main text or in the Supporting Information. Pyrolysis of the impregnated support was carried out in a Dekema Austromat 626 furnace under Ar (5.0, Linde Gas GmbH). The hydrogenation reactions were conducted in 300 mL Parr steel autoclaves with H_2_ gas (5.0, Linde Gas GmbH). The GCMS analyses were performed on a Shimadzu QP2020 device using an SH-Rxi-5Sil MS column (30 m, 0.25 mm, 0.25 μm) and electron impact ionization was achieved at 70 eV while He (5.0, Linde GmbH) served as the carrier gas (1.7 mL min^–1^). The sample was injected in split mode and processed through the following temperature ramp: 50 °C (hold for 1 min)–320 °C (30 °C min^–1^, then hold for 1 min). The signals were interpreted based on the *m*/*z* and existing databases, whereby conversion rates and yields were calculated with the aid of a calibration curve with *n*-hexadecane serving as the internal standard. All NMR spectra (^1^H, ^13^C, and ^19^F) were recorded on Bruker AVANCE Machines at 300 MHz with chemical shift given in parts per million (ppm) and referenced to the respective solvent residue signal according to the literature.^58^ Coupling constants are given in Hz and signals interpreted with the following abbreviations: s-singlet, d-doublet, t-triplet, q-quartet, and m-multiplet. High-resolution mass spectra were obtained using an Agilent QTOF 6520 with an ESI source operated in positive ionization mode, whereby reference mass standards (*m*/*z* = 121.050873 and *m*/*z* = 922.009798) were obtained from the same company and infused throughout all measurements. The drying gas temperature was set at 350 °C and the drying gas flow rate amounted to 10 L min^–1^. The nebulizer pressure was maintained at 45 psi, the capillary voltage set to 3500 V, and that of the fragmentor to 150 V. Spectra were recorded from 45 to 1500 *m*/*z*, and data analysis was conducted using an Agilent Mass Hunter Qualitative Analysis B.07.00. Elemental analyses were done on a Leco Microanalysator TruSpec for C, H, N, and S, while the respective metal content was determined by means of atomic absorption spectroscopy (AAS Analyst 300 from PerkinElmer). XPS measurements were performed using the Nexsa G2 system, and the acquired spectra assessed by means of the Avantage software package provided by the system manufacturer. Samples were probed with a monochromated Al Kα X-ray source (1486.7 eV) focused to a spot having 400 μm in diameter. The hemispherical analyzer was operated in constant energy mode with a pass energy of 200 eV and an energy step of 1 eV for survey spectra and 50 and 0.05 eV step, respectively, for high-resolution spectra. The built-up surface charge was neutralized with a dual flood gun. A standard charge shift referencing of the spectra via the C 1s peak of adventitious carbon at 285.0 eV was applied. Electron micrographs were recorded with a scanning AES microscope JAMP-9500 F (JEOL, JP). For the purpose of energy-dispersive X-ray spectroscopy (EDX), the system was equipped with a custom designed windowless detector Ultim Max (Oxford Instruments, UK). The images and EDX spectra were recorded with an acceleration voltage of 10 kV and an electron beam current of 5 nA.

### Ligand Synthesis

Phthalocyanine was purchased but also synthesized according to a published procedure:^59^ phthalonitrile (3.841 g, 30.0 mmol) was placed in a 100 mL pressure vial and dissolved in EtOH (50 mL) whereupon DBU (2.24 mL, 15.0 mmol—freshly distilled) was added. The flask was sealed, and the reaction mixture stirred (160 °C, 24 h). Upon completion, the suspension was filtered and washed with H_2_O (20 mL) and then with EtOH until the filtrate ran clear. The solid remains were dried at 120 °C for 24 h, yielding sufficiently pure phthalocyanine as a purple solid (2.681 g, 5.2 mmol, 70%).

### Catalyst Preparation

Solid phthalocyanine (259.7 mg, 0.50 mmol), [Co­(acac)_2_] (258.7 mg, 1.04 mmol), and Vulcan XC 72R (1.499 g) were triturated in a mortar (20 min) with the help of a pestle (both made from agate) upon which the mixture was transferred to a ceramic crucible. The latter was then placed in a furnace, and the content was pyrolyzed (800 °C, 2 h, 25 °C min^–1^ heating rate, Ar atmosphere). On cooling to R.T., the raw catalyst was again homogenized (10 min) to obtain ready-to-use **[CoPhc]@Vulcan**.

### General Hydrogenation Procedure

Without any protection from air, a glass vial (4 mL) was charged with **[CoPhc]@Vulcan**, the substrate, solvent, and a magnetic stirrer bar, in that order. The vial was then sealed with a septum cap that was pierced and fitted with a steel cannula. Hereafter, the thus-prepared glass container was placed in an inlet made from aluminum and transferred into the autoclave. After the pressure vessel had been assembled, it was flushed with H_2_ (3 × 50 bar), and the desired pressure was set. The filled autoclave was placed on a heating plate, and the reaction was started. On completion of the reaction, the autoclave was immersed in an ice bath, allowed to reach R.T., and depressurized. The liquid reaction mixture was filtered, and the clear filtrate was submitted to GCMS analysis. In case an internal standard was used, the required amount of the reference compound was added prior to filtration.

### Product Isolation

The reaction mixture was diluted with THF (2 mL), and the resulting suspension was centrifuged (30 min, 3000 rpm) whereupon the supernatant liquid phase was separated with a pipet and then filtered (Whatman filter) to obtain a clear solution. The remaining solid from the first centrifugation step was slurried with THF (2 mL) and again centrifuged, and the liquid portion was filtered. Washing was repeated one more time, and on combining both filtrates and removing the solvent under reduced pressure, the crude product was obtained. The latter was dissolved in DCM (3 mL), and hereafter, 1 M HCl in Et_2_O (2.5 mL) was added to initiate precipitation of the amine as its hydrochloride salts. Eventually, the formed solid was collected on paper filter and washed with DCM (2 × 2 mL).

## Conclusion

Controlled pyrolysis of a solid [Co­(acac)_2_]/phthalocyanine/Vulcan mixture under Ar afforded a N-doped, merged Co/C composite that was applied to the heterogeneous hydrogenation of aromatic azo and nitro compounds to yield a wide array of substituted anilines. The catalyst was ready for immediate use and could be handled in air without the need for further additives. The feasibility of a bootstrap synthesis approach for aromatic amines through reduction of the N=N motif of the corresponding symmetric azo dye was demonstrated for the phenacetin precursor *p*-phenetidine. In the case of the nitroarene-to-aniline reduction, the substrates were even converted at room temperature and comparably low H_2_ pressure (20 bar).

However, due to reactor blockage caused by finely powdered Vulcan, the supported catalyst was not suitable for continuous flow conditions (ThalesNano’s H-Cube Mini Plus). Corresponding experiments with coarser-grained carrier materials, which are supposed to permit a smooth flow of the reaction solution through the catalyst cartridges, are currently being carried out in our research laboratories.

## Supplementary Material



## Data Availability

The data underlying this study are available in the published article and its Supporting Information.

## Notes

Characterization of solid **[CoPhc]@Vulcan** (elemental analysis, XPS scans including aged samples), influence of crucial parameters on the catalyst performance regarding azo hydrogenation (support, catalyst loading, temperature, H_2_ pressure), comparison experiments (Raney Ni and Pd/C vs Co catalyst), recycling study, yield vs time plot and control experiments for **1a** → **2a**, hot filtration test, influence of crucial parameters on the catalyst performance regarding nitroarene hydrogenation (solvent variation study, catalyst loading, temperature, H_2_ pressure), yield vs time plot for **5a** → **6a**, synthetic procedures and characterization data for (un)­symmetrical azo compounds and the products of the catalytic hydrogenation experiments, and spectra (HRMS, ^1^H, ^13^C­{^1^H}, ^19^F NMR, and GC/MS) (PDF)

## References

[1] Blaser H.-U., Steiner H., Studer M. (2009). Selective Catalytic Hydrogenation
of Functionalized Nitroarenes: An update. ChemCatChem.

[2] Orlandi M., Brenna D., Harms R., Jost S., Benaglia M. (2018). Recent Developments
in the Reduction of Aromatic and Aliphatic Nitro Compounds to Amines. Org. Process Res. Dev..

[3] Toti A., Frediani P., Salvini A., Rosi L., Giolli C. (2005). Hydrogenation
of Single and Multiple N–N or N–O Bonds by Ru­(II) Catalysts
in Homogeneous Phase. J. Organomet. Chem..

[4] Das U. K., Kar S., Ben-David Y., Diskin-Posner Y., Milstein D. (2021). Manganese Catalyzed
Hydrogenation of Azo (N = N) Bonds to Amines. Adv. Synth. Catal..

[5] Pedrajas E., Sorribes I., Junge K., Beller M., Llusar R. (2015). A Mild and
Chemoselective Reduction of Nitro and Azo Compounds Catalyzed by a
Well-Defined Mo_3_S_4_ Cluster Bearing Diamine Ligands. ChemCatChem.

[6] Guillamón E., Oliva M., Andrés J., Llusar R., Pedrajas E., Safont V. S., Algarra A. G., Basallote M. G. (2021). Catalytic
Hydrogenation of Azobenzene in the Presence of a Cuboidal Mo_3_S_4_ Cluster via an Uncommon Sulfur-Based H_2_ Activation
Mechanism. ACS Catal..

[7] López-Grimau V., Riera-Torres M., López-Mesas M., Gutiérrez-Bouzán C. (2013). Removal of
Aromatic Amines and Decolourisation of Azo Dye Baths by Electrochemical
Treatment. Color. Technol..

[8] Whitmore W. F., Revukas A. J. (1940). The Quantitative Hydrogenation of Substituted Azo Compounds
with Raney Nickel at Normal Temperature and Pressure. J. Am. Chem. Soc..

[9] Sahoo A., Patra S. (2018). A Combined Process for the Degradation of Azo-Dyes and Efficient
Removal of Aromatic Amines Using Porous Silicon Supported Porous Ruthenium
Nanocatalyst. ACS Appl. Nano Mater..

[10] Selvam K., Sakamoto H., Shiraishi Y., Hirai T. (2015). Photocatalytic Secondary
Amine Synthesis from Azobenzenes and Alcohols on TiO_2_ Loaded
with Pd Nanoparticles. New J. Chem..

[11] Shuwanto H., Abdullah H., Kuo D.-H., Gultom N. S. (2021). Surface Active Sites
of Y-Doped Zn­(O,S) for Chemisorption and Hydrogenation of Azobenzene
and Nitroaromatic Compounds Under Light via Self-Generated Proton. Appl. Surf. Sci..

[12] Panja D., Dey S., Saha R., Sahu R., Das G. K., Bhobe P., Kundu S. (2023). Co-SAC Catalyzed
Utilization of Methanol and Ethanol in the Transfer
Hydrogenation of Azo Bonds: Experimental and Theoretical Studies. Green Chem..

[13] Panja D., Dey S., Kundu S. (2023). Heterogeneous Cobalt-Catalyzed Degradation of Azo Compounds
Using Alcohols as the Stoichiometric Hydrogen Source. J. Environ. Chem. Eng..

[14] Dey S., Panja D., Sau A., Thakur S. D., Kundu S. (2023). Reusable Cobalt-Catalyzed
Selective Transfer Hydrogenation of Azoarenes and Nitroarenes. J. Org. Chem..

[15] Sheldon R. A. (2007). The E Factor:
Fifteen Years On. Green Chem..

[16] Ault, A. Techniques and Experiments for Organic Chemistry, 6th ed.; University Science Books: Sausalito, 1998.

[17] Riedel, J. D. Verfahren zur Darstellung von *p*-Acetphenetidin. D. R. Patent 48543, December 28, 1888.

[18] Kalo, J. Process for the Manufacture of p-Phenetidine. EP 0782981 A1, December 2, 1998.

[19] Sales J., Mushtaq F., Nomen R. (2007). Study of Major
Accidents Involving
Chemical Reactive Substances: Analysis and Lessons Learned. Process Saf. Environ. Prot..

[20] Tamaru, Y. Modern Organonickel Chemistry; Wiley-VCH: Weinheim, 2005.

[21] Arpe, H.-J. Industrial Organic Chemistry, 5th ed.; Wiley-VCH: Weinheim, 2010.

[22] Paul B., Chakrabarti K., Shee S., Maji M., Mishra A., Kundu S. (2016). A Simple and Efficient in situ Generated
Ruthenium Catalyst for Chemoselective
Transfer Hydrogenation of Nitroarenes: Kinetic and Mechanistic Studies
and Comparison with Iridium Systems. RSC Adv..

[23] Wienhöfer G., Baseda-Krüger M., Ziebart C., Westerhaus F. A., Baumann W., Jackstell R., Junge K., Beller M. (2013). Hydrogenation
of Nitroarenes Using Defined Iron-Phosphine Catalysts. Chem. Commun..

[24] Sharma U., Kumar P., Kumar N., Kumar V., Singh B. (2010). Highly Chemo-
and Regioselective Reduction of Aromatic Nitro Compounds Catalyzed
by Recyclable Copper­(II) as well as Cobalt­(II) Phthalocyanines. Adv. Synth. Catal..

[25] Timelthaler T., Schöfberger W., Topf C. (2021). Selective and Additive-Free Hydrogenation
of Nitroarenes Mediated by a DMSO-Tagged Molecular Cobalt Corrole
Catalyst. Eur. J. Org. Chem..

[26] Zubar V., Dewanji A., Rueping M. (2021). Chemoselective Hydrogenation
of Nitroarenes
Using an Air-Stable Base-Metal Catalyst. Org.
Lett..

[27] Kim S., Kim E., Kim B. M. (2011). Fe_3_O_4_ Nanoparticles: A Conveniently
Reusable Catalyst for the Reduction of Nitroarenes Using Hydrazine
Hydrate. Chem.—Asian J..

[28] Jagadeesh R. V., Surkus A.-E., Junge H., Pohl M.-M., Radnik J., Rabeah J., Huan H., Schünemann V., Brückner A., Beller M. (2013). Nanoscale Fe_2_O_3_-Based Catalysts for Selective Hydrogenation of Nitroarenes
to Anilines. Science.

[29] Zheng Y., Ma K., Wang H., Sun X., Jiang J., Wang C., Li R., Ma J. (2008). A Green Reduction
of Aromatic Nitro Compounds to Aromatic
Amines Over a Novel Ni/SiO_2_ Catalyst Passivated with a
Gas Mixture. Catal. Lett..

[30] Kour G., Gupta M., Vishwanathan B., Thirunavukkarasu K. (2016). (Cu/NCNTs):
A New High Temperature Technique to Prepare a Recyclable Nanocatalyst
for Four Component Pyridine Derivative Synthesis and Nitroarenes Reduction. New J. Chem..

[31] Brown O. W., Henke C. O. (1922). Catalytic Preparation of Aniline. II. J. Phys. Chem..

[32] Westerhaus F. A., Jagadeesh R. V., Wienhöfer G., Pohl M.-M., Radnik J., Surkus A.-E., Rabeah J., Junge K., Junge H., Nielsen M., Brückner A., Beller M. (2013). Heterogenized Cobalt
Oxide Catalysts for Nitroarene Reduction by Pyrolysis of Molecularly
Defined Complexes. Nat. Chem..

[33] Jagadeesh R. V., Banerjee D., Arockiam P. B., Junge H., Junge K., Pohl M.-M., Radnik J., Brückner A., Beller M. (2015). Highly Selective Transfer Hydrogenation
of Functionalised
Nitroarenes Using Cobalt-Based Nanocatalysts. Green Chem..

[34] Jagadeesh R. V., Stemmler T., Surkus A.-E., Bauer M., Pohl M.-M., Radnik J., Junge K., Junge H., Brückner A., Beller M. (2015). Cobalt-Based Nanocatalysts
for Green Oxidation and
Hydrogenation Processes. Nat. Protoc..

[35] Formenti D., Topf C., Junge K., Ragaini F., Beller M. (2016). Fe_2_O_3_/NGr@C-
and Co–Co_3_O_4_/NGr@C-Catalysed
Hydrogenation of Nitroarenes Under Mild Conditions. Catal. Sci. Technol..

[36] Eckardt M., Zaheer M., Kempe R. (2018). Nitrogen-Doped Mesoporous SiC Materials
with Catalytically Active Cobalt Nanoparticles for the Efficient and
Selective Hydrogenation of Nitroarenes. Sci.
Rep..

[37] Schwob T., Kempe R. (2016). A Reusable Co Catalyst
for the Selective Hydrogenation of Functionalized
Nitroarenes and the Direct Synthesis of Imines and Benzimidazoles
from Nitroarenes and Aldehydes. Angew. Chem.
Int. Ed..

[38] Cui X., Liang K., Tian M., Zhu Y., Ma J., Dong Z. (2017). Cobalt Nanoparticles
Supported on *N*-Doped Mesoporous
Carbon as a Highly Efficient Catalyst for the Synthesis of Aromatic
Amines. J. Colloid Interface Sci..

[39] Nandi S., Patel P., Khan N. H., Biradar A. V., Kureshy R. I. (2018). Nitrogen-Rich
Graphitic-Carbon Stabilized Cobalt Nanoparticles for Chemoselective
Hydrogenation of Nitroarenes at Milder Conditions. Inorg. Chem. Front..

[40] Sun X., Olivos-Suarez A. I., Osadchii D., Romero M. J. V., Kapteijn F., Gascon J. (2018). Single Cobalt Sites in Mesoporous *N*-Doped Carbon Matrix for Selective Catalytic Hydrogenation of Nitroarenes. J. Catal..

[41] Sahoo B., Formenti D., Topf C., Bachmann S., Scalone M., Junge K., Beller M. (2017). Biomass-Derived
Catalysts for Selective
Hydrogenation of Nitroarenes. ChemSusChem.

[42] Zhang F., Zhao C., Chen S., Li H., Yang H., Zhang X.-M. (2017). In situ Mosaic Strategy Generated
Co-Based *N*-Doped Mesoporous Carbon for Highly Selective
Hydrogenation
of Nitroaromatics. J. Catal..

[43] Zhou P., Jiang L., Wang F., Deng K., Lv K., Zhang Z. (2017). High Performance of a Cobalt-Nitrogen Complex for the
Reduction and
Reductive Coupling of Nitro Compounds into Amines and Their Derivatives. Sci. Adv..

[44] Wang X., Li Y. (2016). Chemoselective Hydrogenation of Functionalized Nitroarenes Using
MOF-Derived Co-Based Catalysts. J. Mol. Catal.
A: Chem..

[45] Yang S., Peng L., Oveisi E., Bulut S., Sun D. T., Asgari M., Trukhina O., Queen W. L. (2018). MOF-Derived Cobalt
Phosphide/Carbon Nanocubes for Selective Hydrogenation of Nitroarenes
to Anilines. Chem.—Eur. J..

[46] Ji P., Manna K., Lin Z., Feng X., Urban A., Song Y., Lin W. (2017). Single-Site
Cobalt Catalysts at New
Zr_12_(μ_3_-O)_8_(μ_3_-OH)_8_(μ_2_-OH)_6_ Metal-Organic
Framework Nodes for Highly Active Hydrogenation of Nitroarenes, Nitriles,
and Isocyanides. J. Am. Chem. Soc..

[47] Sorribes I., Liu L., Corma A. (2017). Nanolayered
Co–Mo–S Catalysts for the
Chemoselective Hydrogenation of Nitroarenes. ACS Catal..

[48] Formenti D., Ferretti F., Topf C., Surkus A.-E., Pohl M.-M., Radnik J., Schneider M., Junge K., Beller M., Ragaini F. (2017). Co-Based Heterogeneous Catalysts from Well-Defined
α-Diimine Complexes: Discussing the Role of Nitrogen. J. Catal..

[49] Schmiedbauer F., Monkowius U., Schwarzinger C., Müllegger S., Bartling S., Rockstroh N., Topf C. (2025). Hydrogenation of Quinolines
and Aldehydes Catalyzed by a Pyrolyzed, Augmented Cobalt-Salen Complex. ACS Omega.

[50] Lazar P., Mach R., Otyepka M. (2019). Spectroscopic Fingerprints
of Graphitic,
Pyrrolic, Pyridinic, and Chemisorbed Nitrogen in *N*-Doped Graphene. J. Phys. Chem. C.

[51] Biesinger M. C., Payne B. P., Grosvenor A. P., Lau L. W. M., Gerson A. R., Smart St., Resolving R. C. (2011). Resolving
Surface Chemical States
in XPS Analysis of First Row Transition Metals, Oxides and Hydroxides:
Cr, Mn, Fe, Co, and Ni. Appl. Surf. Sci..

[52] Hinterreiter A. P., Duchoslav J., Kehrer M., Truglas T., Lumetzberger A., Unterweger C., Fürst C., Stifter D. (2019). Determination of the
Surface Chemistry of Ozone-Treated Carbon Fibers by Highly Consistent
Evaluation of X-Ray Photoelectron Spectra. Carbon.

[53] Chen F., Surkus A.-E., He L., Pohl M.-M., Radnik J., Topf C., Junge K., Beller M. (2015). Selective Catalytic
Hydrogenation of Heteroarenes with N-Graphene-Modified Cobalt Nanoparticles
(Co_3_O_4_-Co/NGr@α-Al_2_O_3_). J. Am. Chem. Soc..

[54] Ferraccioli R., Borovika D., Surkus A.-E., Kreyenschulte C., Topf C., Beller M. (2018). Synthesis of Cobalt Nanoparticles
by Pyrolysis of Vitamin B_12_: A Non-Noble-Metal Catalyst
for Efficient Hydrogenation of Nitriles. Catal.
Sci. Technol..

[55] Michalke J., Haas M., Krisch D., Bögl T., Bartling S., Rockstroh N., Schöfberger W., Topf C. (2022). Generation of Cobalt-Containing Nanoparticles
on Carbon via Pyrolysis
of a Cobalt Corrole and Its Application in the Hydrogenation of Nitroarenes. Catalysts.

[56] Michalke J., Böhm D., Schmiedbauer F., Schwarz J. F., Vogl L. S., Bartling S., Rockstroh N., Topf C. (2024). A Robust, Versatile,
and Reusable Heterogeneous Hydrogenation Catalyst Based on a Simple
Ni­(II) Diimine Complex and its Application to the Syntheses of Amines. Tetrahedron Green Chem.

[57] Hamlin J. E., Hirai K., Millan A., Maitlis P. M. (1980). A Simple Practical
Test for Distinguishing a Heterogeneous Component in a Homogeneously
Catalyzed Reaction. J. Mol. Catal..

[58] Fulmer G. R., Miller A. J. M., Sherden N. H., Gottlieb H. E., Nudelman A., Stoltz B. M., Bercaw J. E., Goldberg K. I. (2010). NMR Chemical Shifts
of Trace Impurities: Common Laboratory Solvents, Organics, and Gases
in Deuterated Solvents Relevant to the Organometallic Chemist. Organometallics.

[59] Tomoda H., Saito S., Shiraishi S. (1983). Synthesis of Metallophthalocyanines
from Phthalonitrile with Strong Organic Bases. Chem. Lett..

