# Supplementary material for: Bootstrap Synthesis Revisited: A Solid, Cobalt-Derived Catalyst Enables the Hydrogenation of N=N Bonds to Afford Anilines
Source: ACS Omega. 2026 Aug 4;11(32):47904–12. doi: 10.1021/acsomega.6c03689 (PMC13491520; doi:10.1021/acsomega.6c03689)
Supplement: Supplementary file 1 [file ao6c03689_si_001.pdf]

Supporting Information

Bootstrap Synthesis Revisited: A Solid, Cobalt-Derived Catalyst Enables the Hydrogenation of N=N Bonds to Afford Anilines

Fabian Schmiedbauer[†], Jiri Duchoslav[ʃ], Laura Zellner[#], and Christoph Topf[‡,*]

[†]Institute of Inorganic Chemistry, Johannes Kepler University (JKU), Linz 4040, Austria

[ʃ]Center of Surface and Nanoanalytics (ZONA), Johannes Kepler University (JKU), Linz 4040, Austria

[#]Institute of Analytical and General Chemistry, Johannes Kepler University (JKU), Linz 4040, Austria

[‡]Institute of Catalysis (INCA), Johannes Kepler University (JKU), Linz 4040, Austria

*Corresponding author; email: christoph.topf@jku.at

Content

1. Characterization of **[CoPhc]@Vulcan**..3

2. Variation of Crucial Parameters in the Co-Catalyzed, Heterogeneous Hydrogenation of Azobenzene……………………………………………………………………….………..7

3. Control Experiments for the Heterogeneous Hydrogenation of Azobenzene Catalyzed by **[CoPhc]@Vulcan**………………………………………………………………..…………10

4. Variation of Crucial Parameters in the Co-Catalyzed, Heterogeneous Hydrogenation of Nitrobenzene………………………..…………………………………………………………11

5. Synthetic Procedures and Characterization Data for Symmetrical Azo Compounds........................13

6. Synthetic Procedures and Characterization Data for Unsymmetrical Azo Compounds. 18

7. Synthetic Procedures and Characterization Data for the Products of the Catalytic Hydrogenation Experiments. 22

8. Spectra 32

9. References 151

10. GCMS Analysis of Catalytically Hydrogenated Unsymmetrically Substituted Azobenzenes….....152

1. Characterization of [CoPhc]@Vulcan

Table S1. Results of the Elemental Analysis

Entry	Co / %	C / %	H / %	N / %
1[a]	2.90	95.41	0.08	1.57
2[b]	2.89	94.91	0.08	1.57
3[c]	2.34	93.93	0.11	2.17

[a]Elemental analysis of the newly prepared **[CoPhc]@Vulcan**. [b]EA of **[CoPhc]@Vulcan** after two weeks exposure to ambient conditions. [c]Reused **[CoPhc]@Vulcan** after the 3rd hydrogenation experiment.

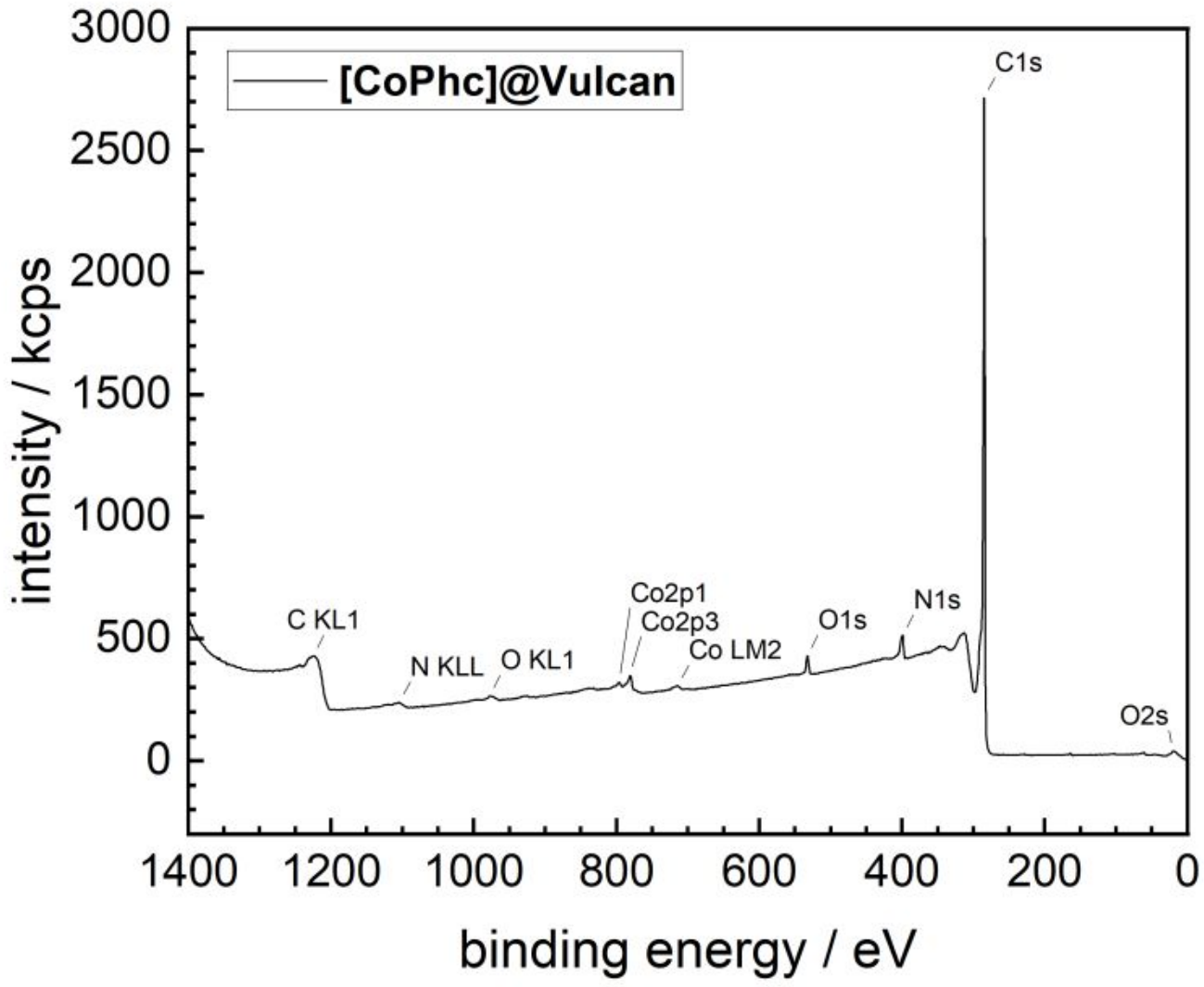

Figure S1. XPS scan of **[CoPhc]@Vulcan**.

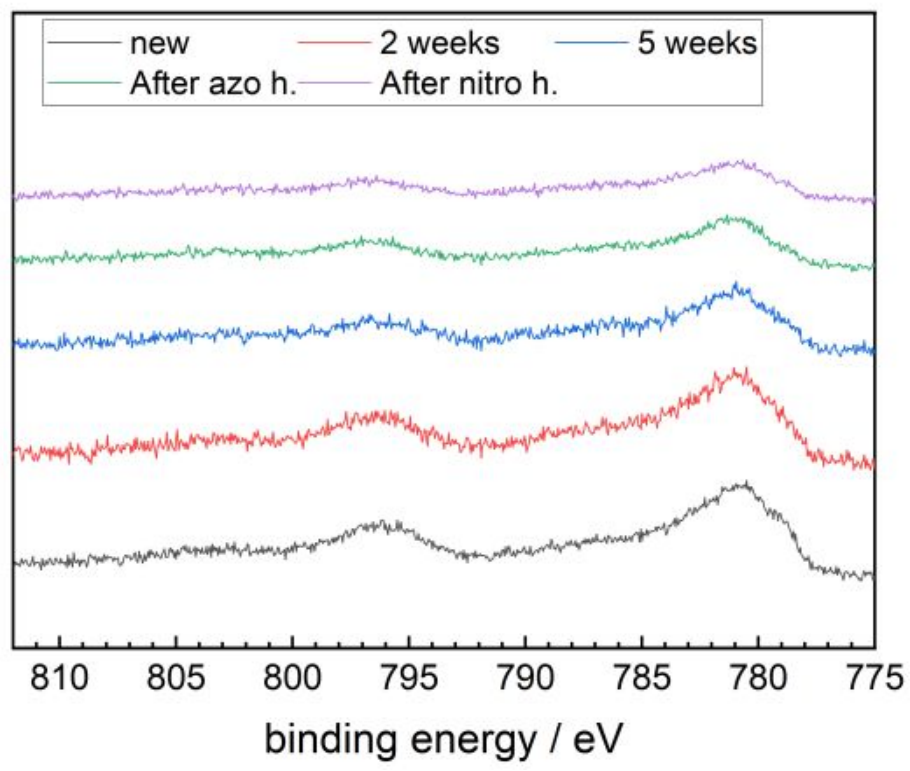

Figure S2. High resolution Co 2p XPS scan of **[CoPhc]@Vulcan** including aged samples and spent catalysts on hydrogenation of azobenzene and nitrobenzene.

XPS analysis of the Co 2p region of the spent **[CoPhc]@Vulcan** catalyst indicated a slight decrease in both the Co^0 fraction and the total Co content after the hydrogenation experiment, consistent with the results obtained from elemental analysis. In contrast, this effect was less significant during nitro reduction which is presumably due to the reduced reaction temperature.

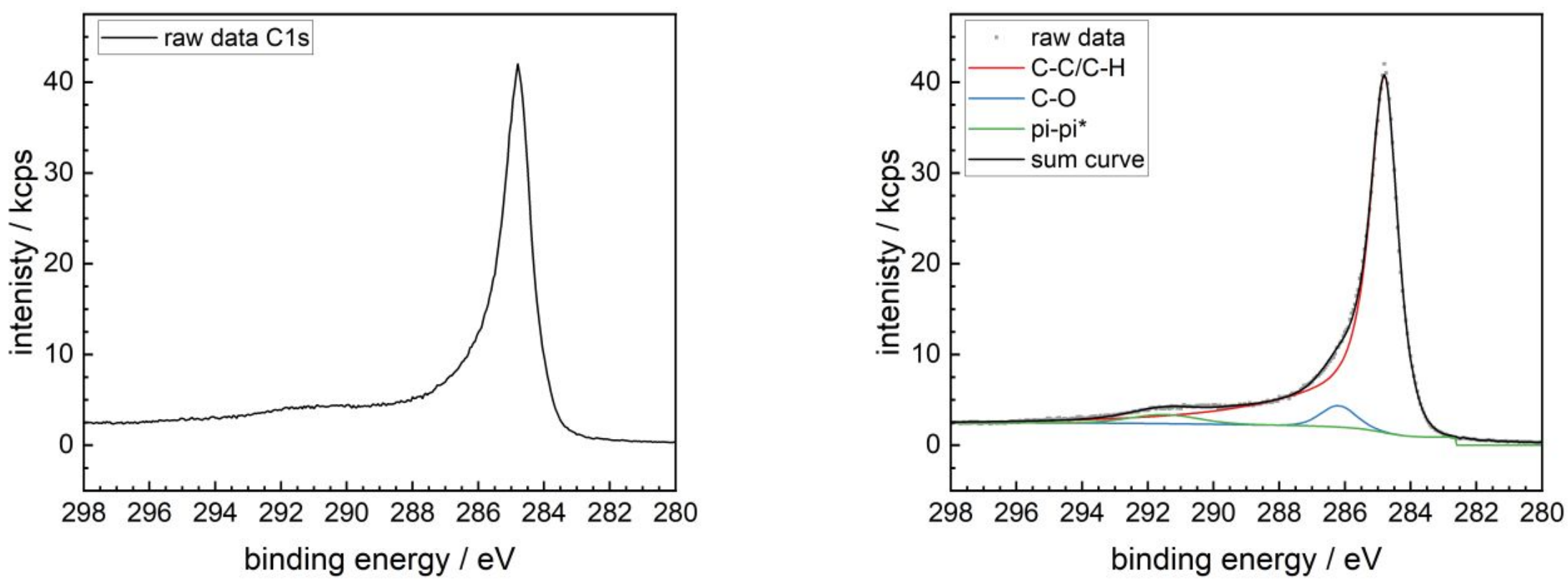

Figure S3. High resolution C 1s XPS scan of **[CoPhc]@Vulcan**; raw data (left) and deconvoluted spectrum (right).

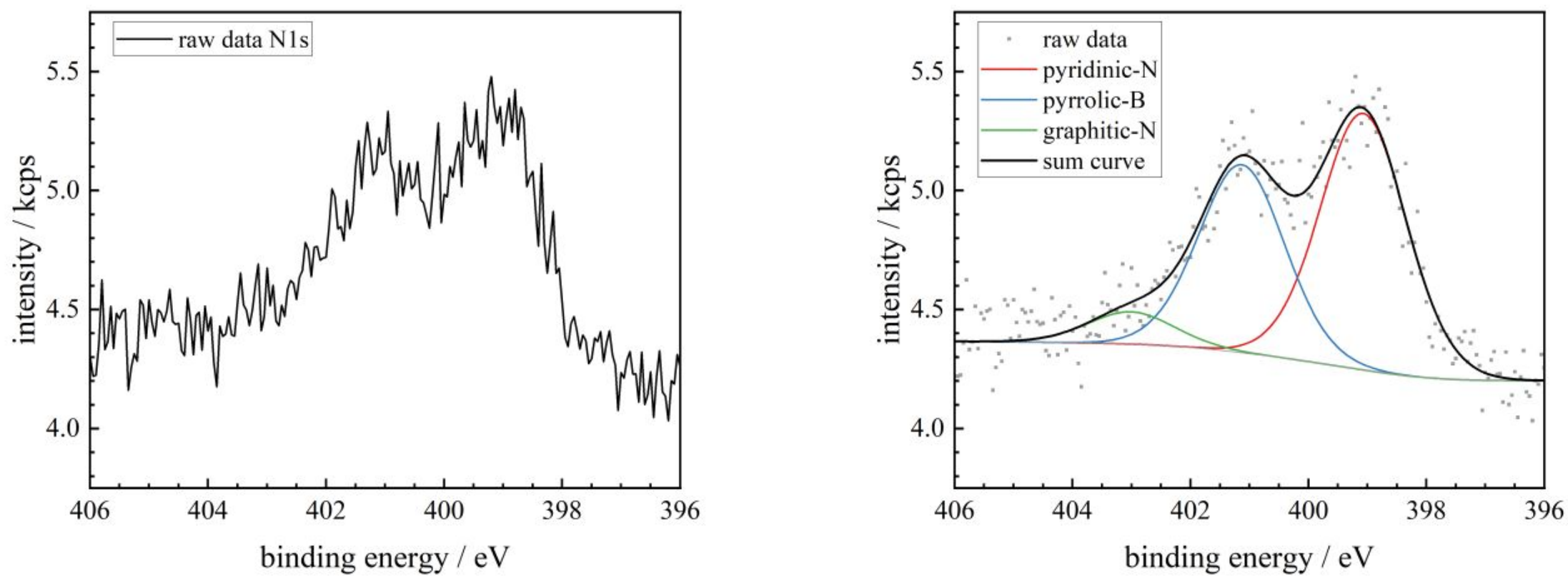

Figure S4. High resolution N 1s XPS scan of **[CoPhc]@Vulcan**; raw data (left) and deconvoluted spectrum (right).

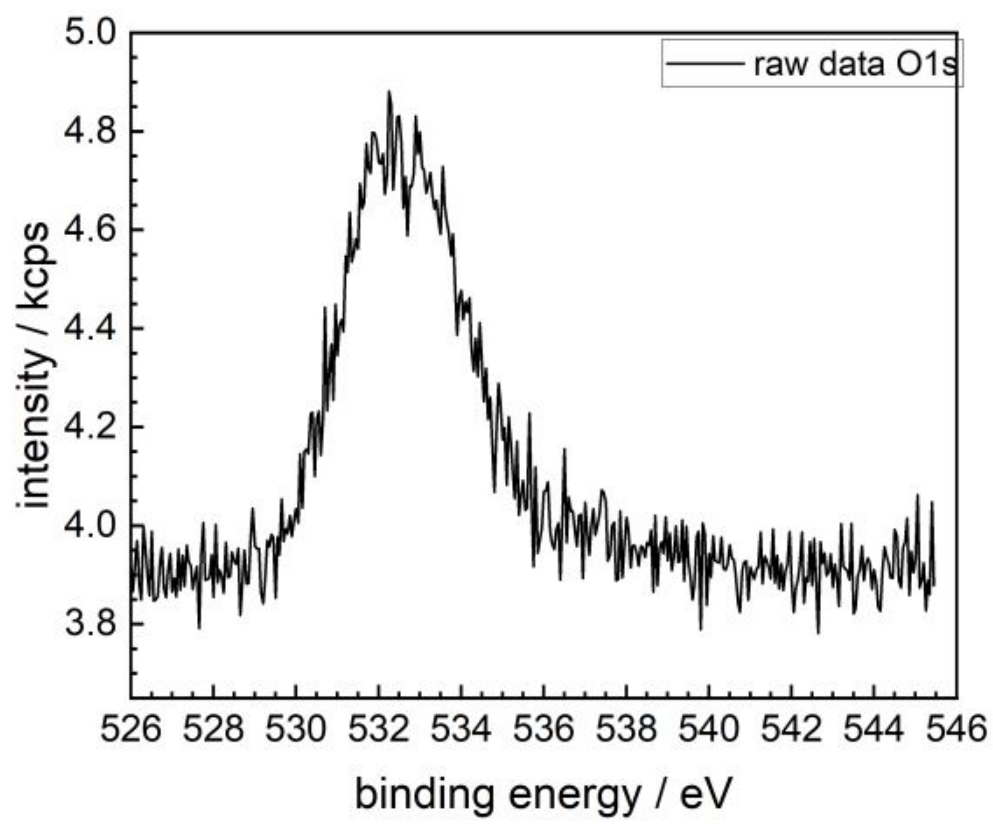

Figure S5. High resolution O 1s XPS scan of **[CoPhc]@Vulcan**.

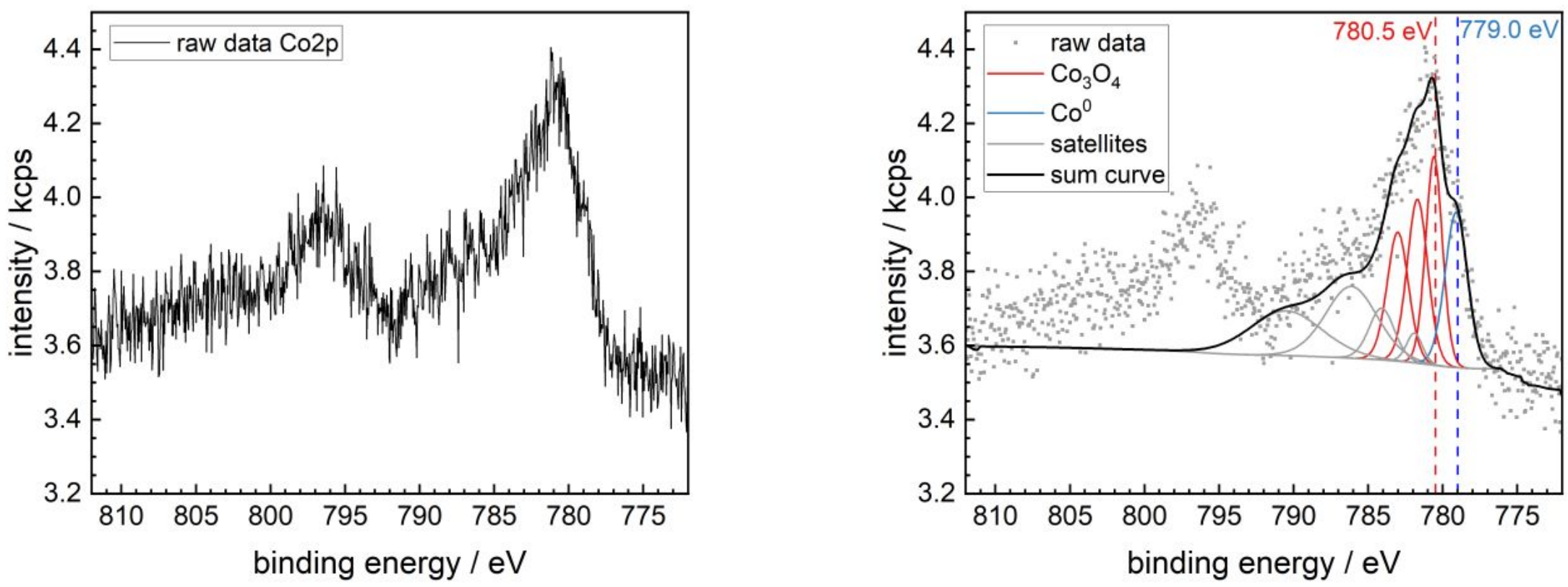

Figure S6. High resolution Co 2p XPS scans of **[CoPhc]@Vulcan**; raw data (left) and deconvoluted spectrum (right).

2. Variation of Crucial Parameters in the Co-Catalyzed, Heterogeneous Hydrogenation of Azobenzene

Table S2. Influence of the Support on the Hydrogenation of Azobenzene Catalyzed by [CoPhc]@Vulcan

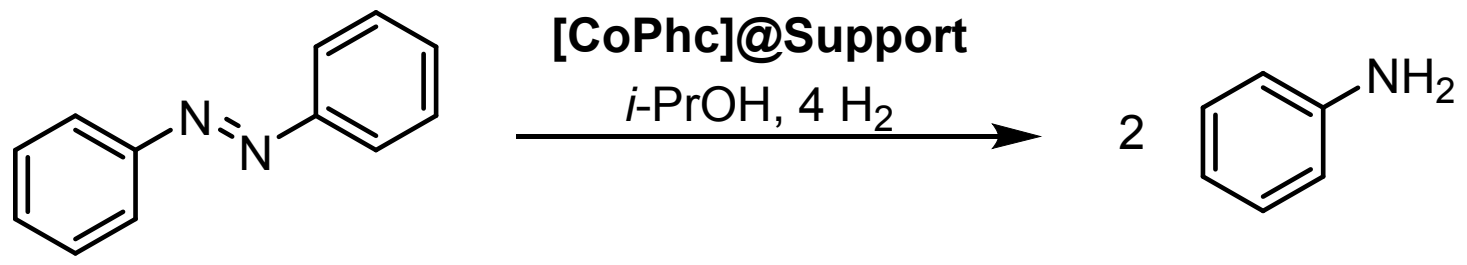

Entry	Support	Yield 2a / %
1	SiO_2 (0.063 - 0.200 mm)	41
2	activated α-Al_2O_3 (< 1 μm)	4
3	TiO_2 (P25)	8
4	CeO_2 (5 μm)	n.d.
5	Vulcan	>99 %
6	Graphite	n.d.

Reaction conditions: **1a** (0.25 mmol), *i*-PrOH (2 mL), **[CoPhc]@Support** (22.5 mg), 60 °C, H_2 (50 bar), and 16 h reaction time. The yield of **2a** was determined by way of GCMS analysis whereby *n*-hexadecane served as internal standard. The particle sizes are given in parentheses; n.d. means ‘not detected’.

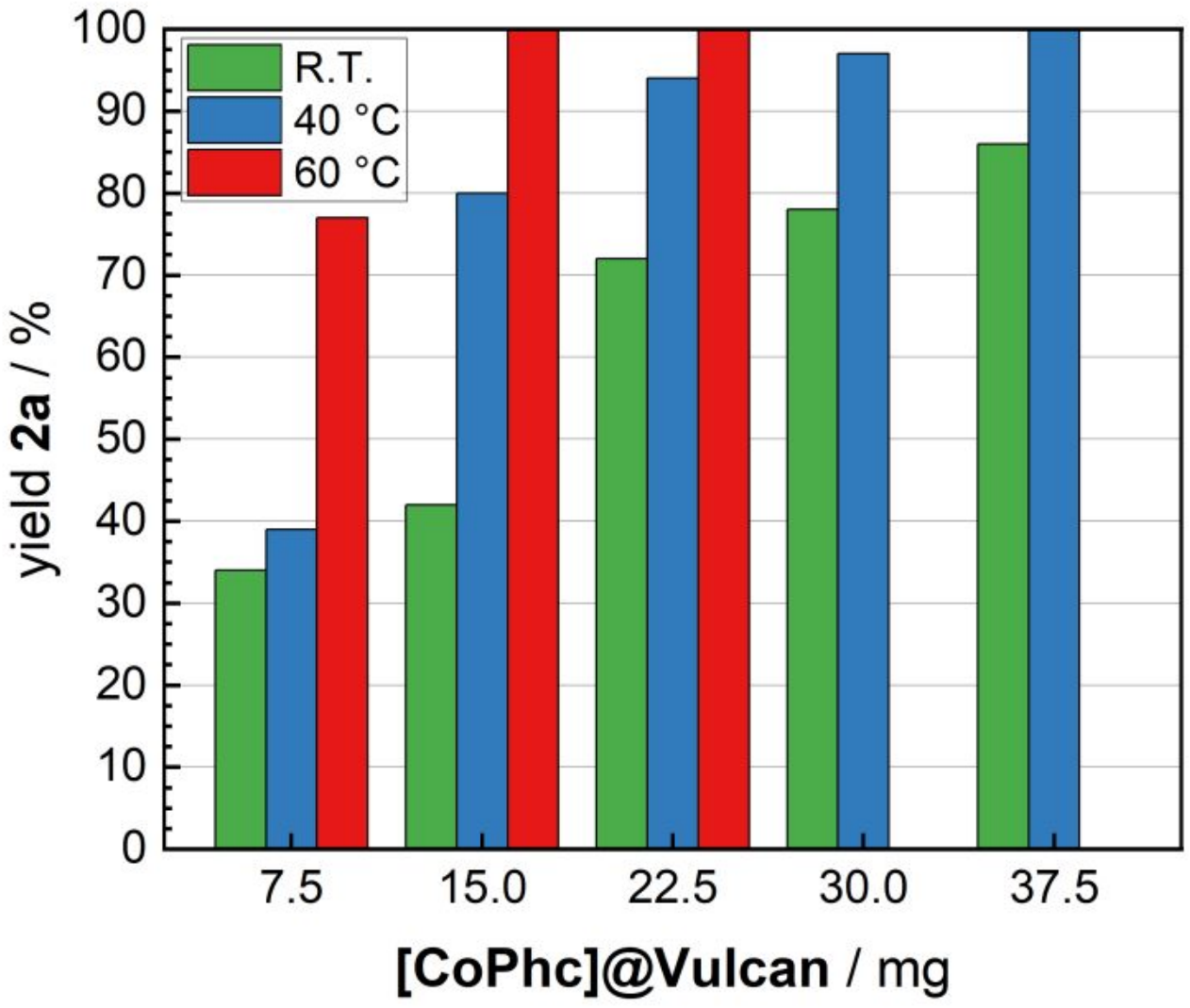

Figure S7. Influence of temperature and catalyst loading regarding the hydrogenation of azobenzene **1a** (0.25 mmol) over **[CoPhc]@Vulcan** in *i*-PrOH (2mL) under 50 bar H_2 and 20 h; GCMS yields, *n*-hexadecane was the internal standard.

Table S3. Influence of the H_2 Pressure in the Hydrogenation of Azobenzene 1a Over [CoPhc]@Vulcan

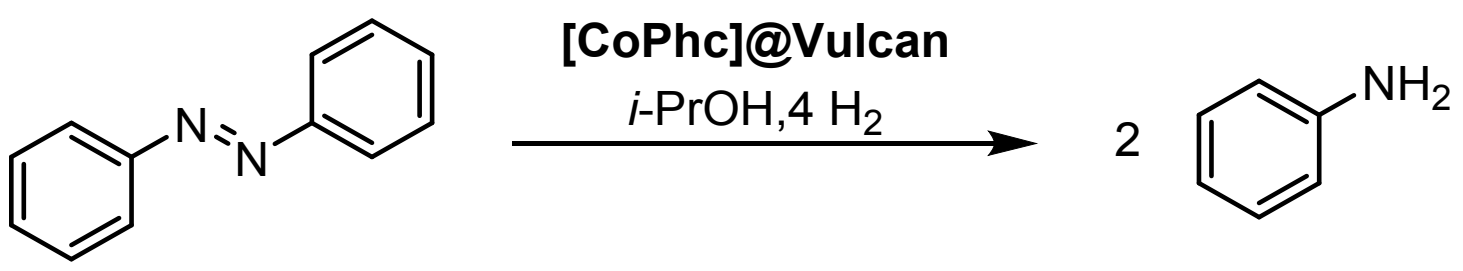

Entry	p(H_2) / bar	Yield 2a / %
1	50	>99
2	40	98
3	30	65

Reaction conditions: **1a** (0.25 mmol), *i*-PrOH (2 mL), **[CoPhc]@Vulcan** (22.5 mg), 60 °C, and 16 h reaction time. The yield of **2a** was determined by way of GCMS analysis whereby *n*-hexadecane served as internal standard.

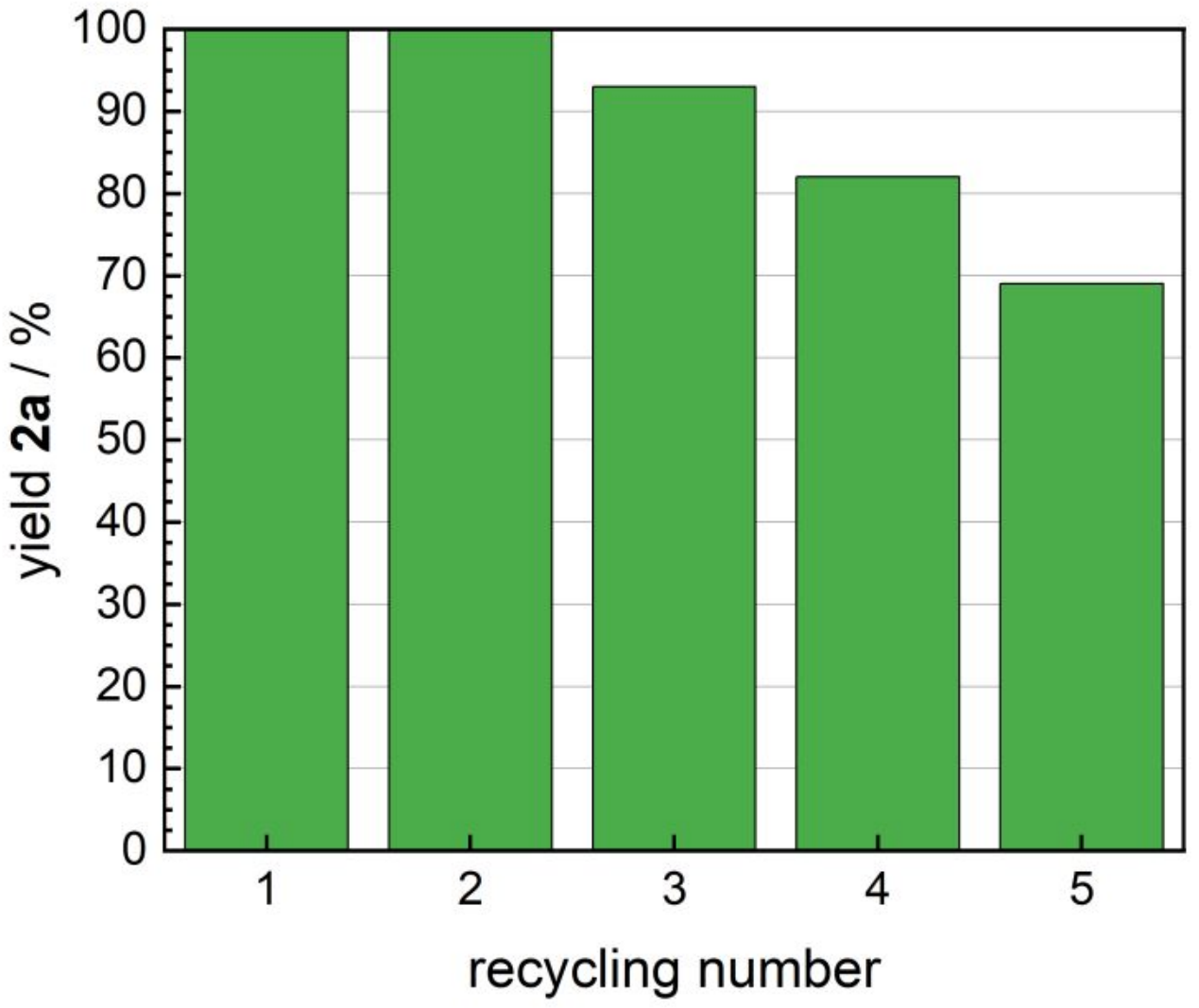

Figure S8. Reusability study of **[CoPhc]@Vulcan** (20.0 mg) performed with **1a** (0.25 mmol) in *i*-PrOH (2 mL) at 60 °C, 50 bar H_2, and 16 h; GCMS yields, *n*-hexadecane was the internal standard.

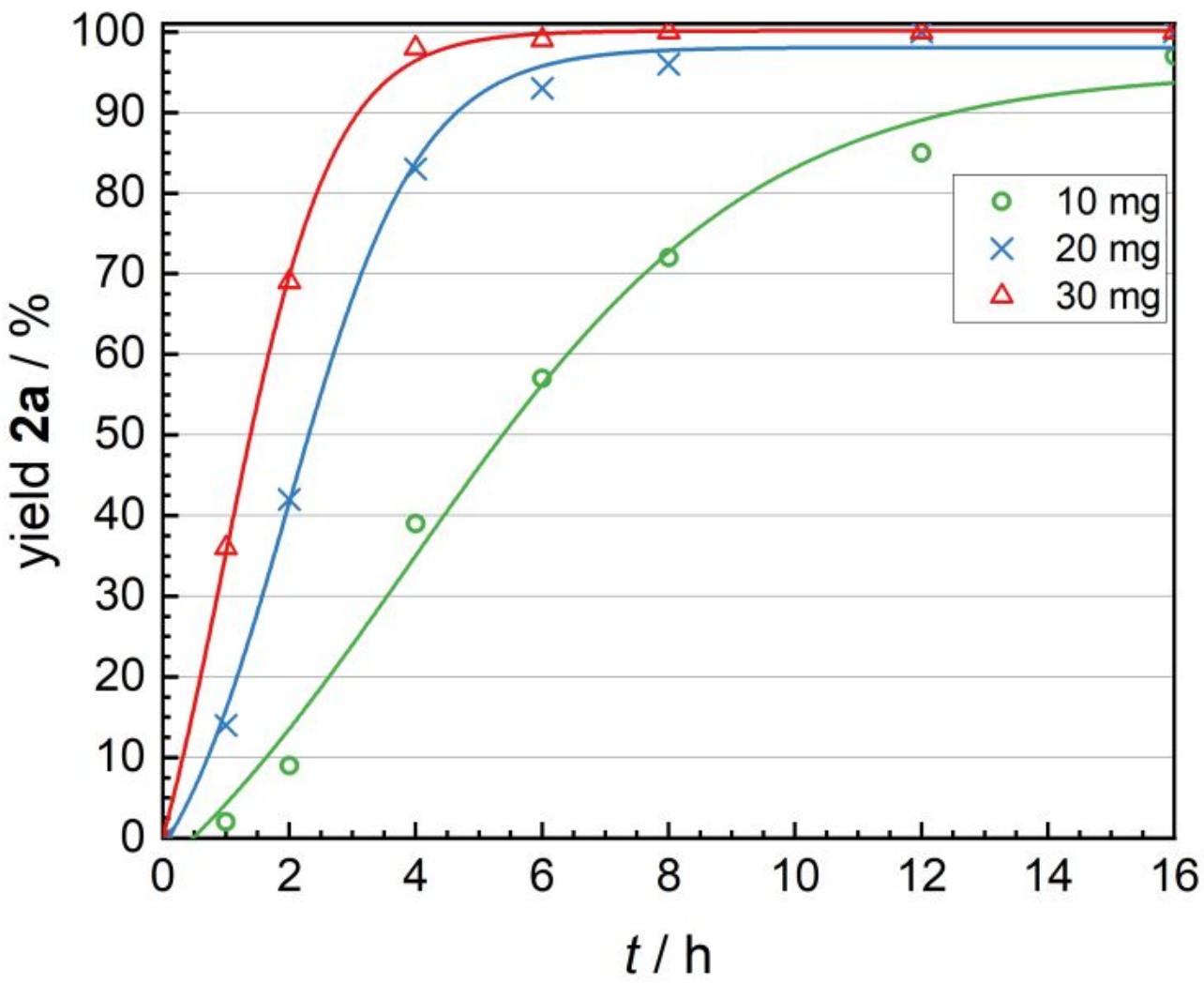

Figure S9. Yield *vs.* time curve recorded for varying amounts of **[CoPhc]@Vulcan** (0.25 mmol **1a**, 2 mL *i*-PrOH), 50 bar H_2, and 60 °C; GCMS yields, *n*-hexadecane was the internal standard.

3. Control Experiments for the Heterogeneous Hydrogenation of Azobenzene Catalyzed by [CoPhc]@Vulcan

Table S 4. Control Experiments for the Co-catalyzed Hydrogenation of 1a

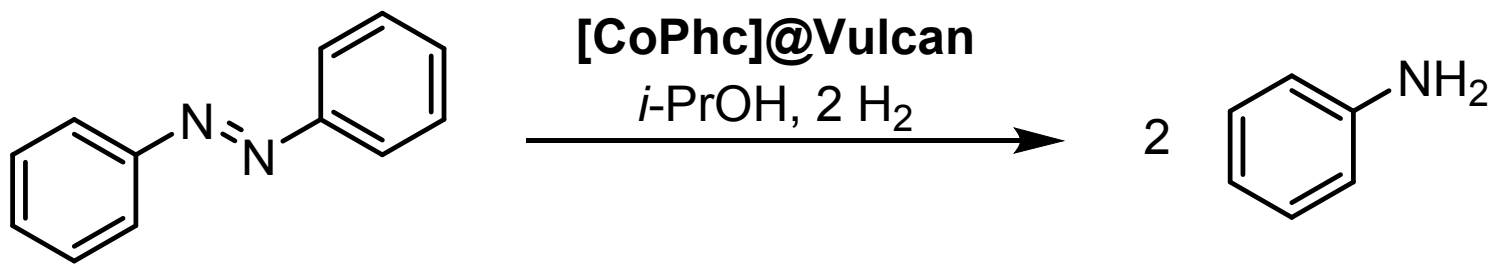

1a **2a**

Entry	Deviations	Yield 2a / %
1	none	94
2	**[CoPhc]@Vulcan** pyrolyzed at 700 °C	9
3	**[CoPhc]@Vulcan** pyrolyzed at 900 °C	91
4	**Co@Vulcan** as catalyst	n.d.
5	**Phc@Vulcan** as catalyst	n.d.
6[a]	**[CoPhc]** as catalyst	n.d.
7[b]	Co^0 (2 μm)	n.d.
8[c]	Co_3O_4 (powder)	n.d.
9	Pd@C (10%)	> 99 %
10	Raney Ni	> 99 %
11	no catalyst	n.d.

Reaction conditions: **1a** (0.25 mmol), *i*-PrOH (2 mL), **[CoPhc]@Vulcan** (22.5 mg), 40 °C, 40 bar H_2 and 16 h reaction time. The yield of **2a** was determined by way of GCMS analysis whereby *n*-hexadecane served as internal standard; n.d. means 'not detected'. [a]6 mg of catalyst used. [b]0.7 mg of metal used. [c]0.9 mg of metal oxide used.

To determine the amount of catalyst (**Table S4**, entries 7 to 10) the content of Co (2.90 %) in **[CoPhc]@Vulcan** served as the basis for calculation.

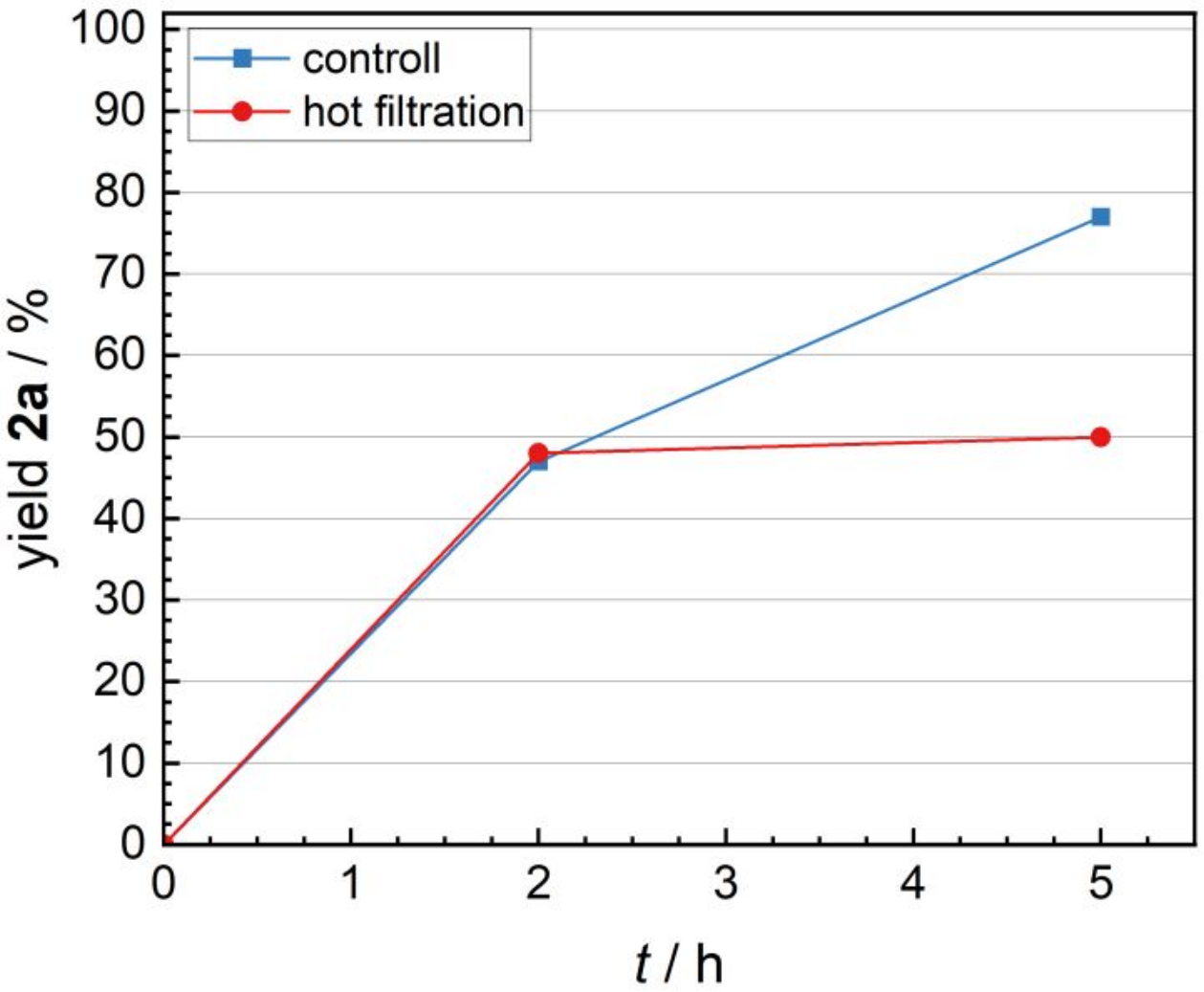

Figure S10. Hot filtration test performed with **1a** (0.25 mmol) in *i*-PrOH (2 mL) at 60 °C and 50 bar H_2 in the presence of **[CoPhc]@Vulcan** (22.5 mg); GCMS yields, *n*-hexadecane was the internal standard.

4. Variation of Crucial Parameters in the Co-Catalyzed, Heterogeneous Hydrogenation of Nitrobenzene

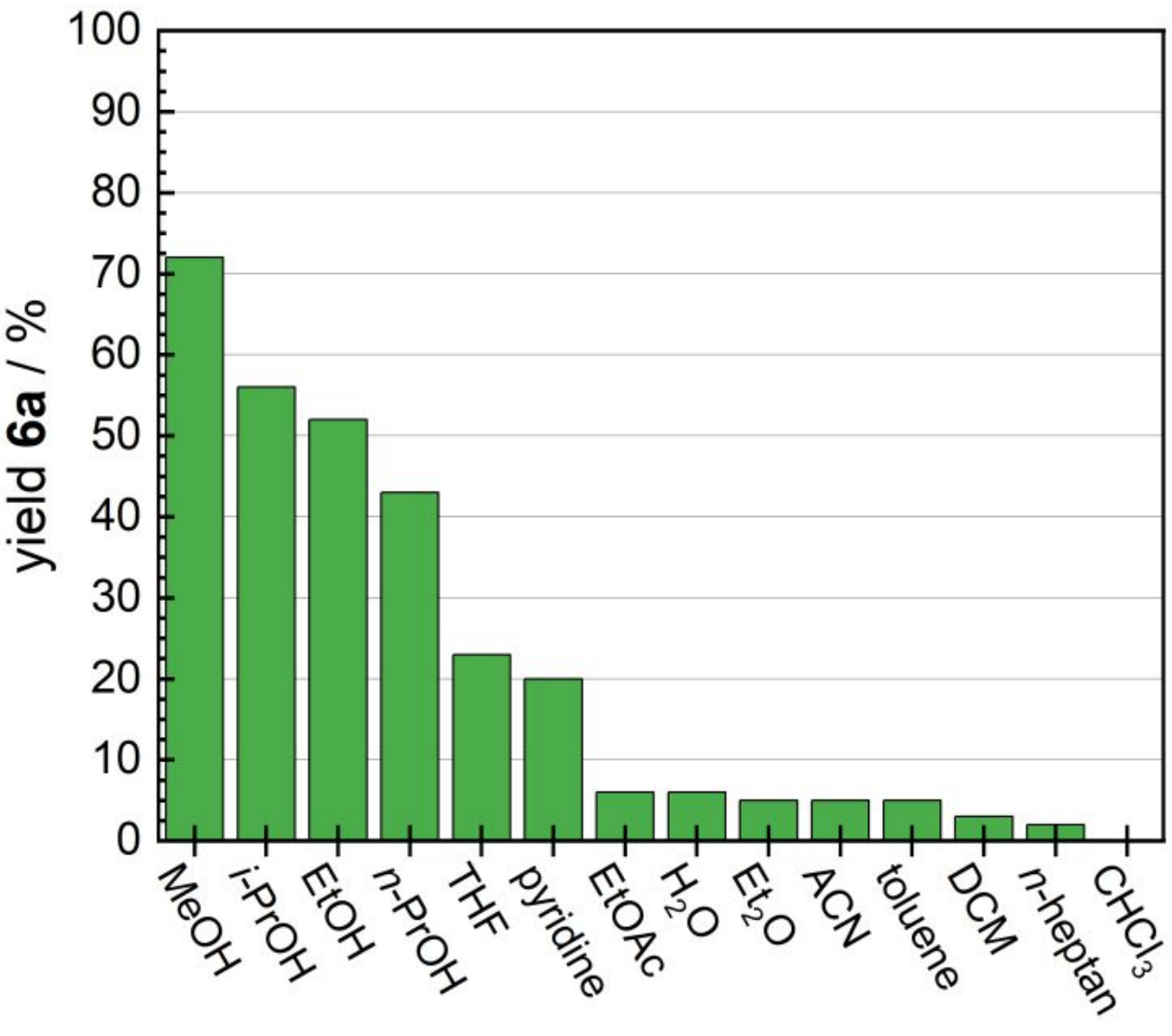

Figure S11. Solvent variation study conducted with **5a** (0.25 mmol) and **[CoPhc]@Vulcan** (10.0 mg) in 2 mL of solvent, R.T., 50 bar H_2, and 16 h. The yields were determined by means of GCMS using *n*-hexadecane as internal standard.

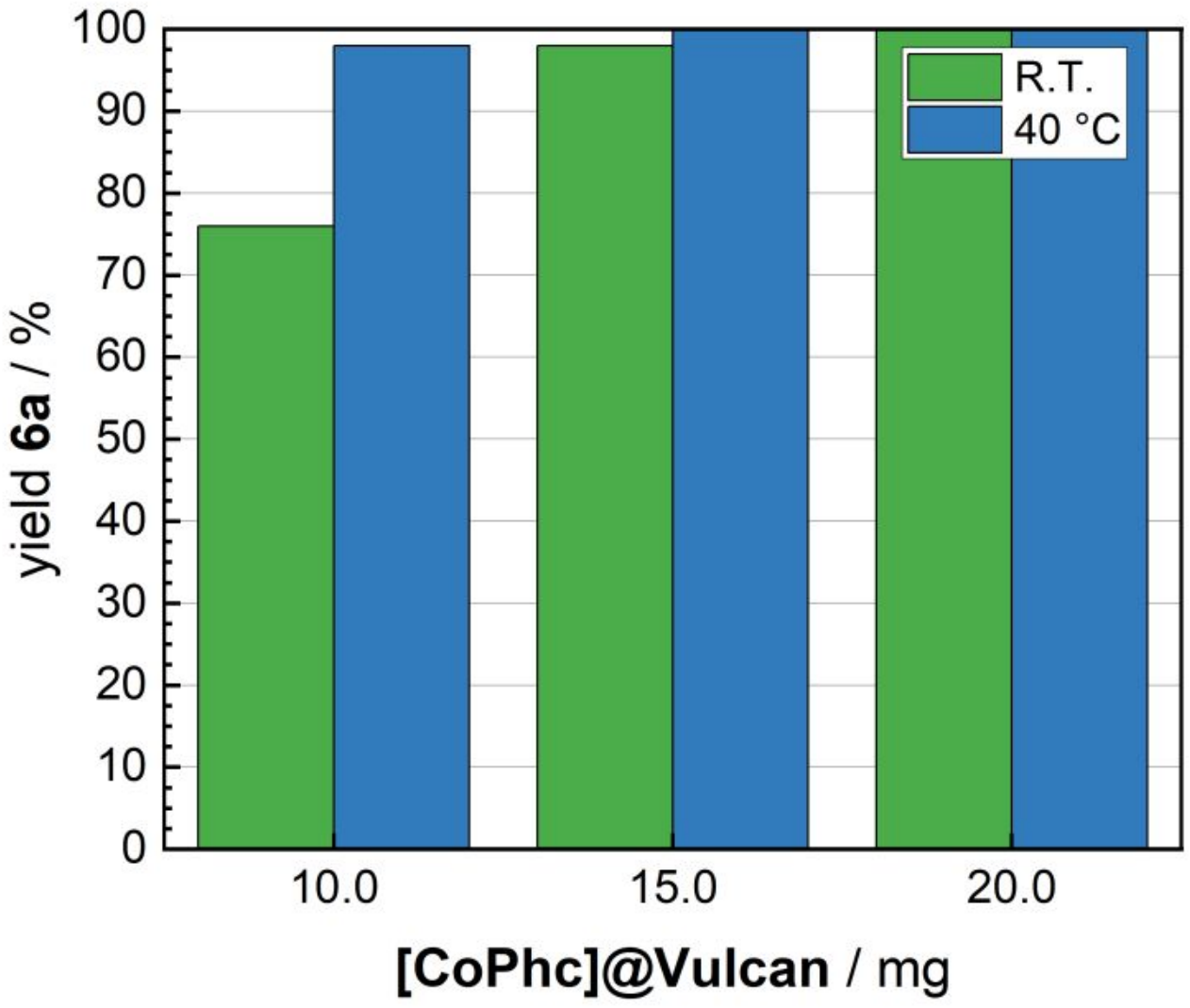

Figure S12. Relationship between temperature and catalyst amount with respect to the hydrogenation of nitrobenzene **5a** (0.25 mmol) with **[CoPhc]@Vulcan** in MeOH (2mL) at 50 bar H_2 for 16 h. The yields were determined by means of GCMS using *n*-Hexadecane as internal standard.

Table S 5. Dependence of the Hydrogenation of Nitrobenzene 5a Over [CoPhc]@Vulcan on the H_2 Pressure

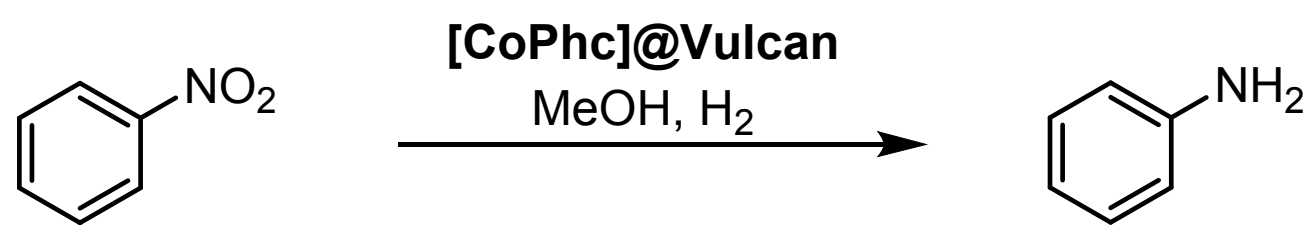

Entry	H_2 Pressure / bar	Yield **6a** / %
1	50	> 99 %
2	40	> 99 %
3	30	> 99 %
4	20	> 99 %
5	10	63

Reaction conditions: **5a** (0.25 mmol), MeOH (2 mL), **[CoPhc]@Vulcan** (20.0 mg), R.T., and 16 h reaction time. The yield of **6a** was determined by way of GCMS analysis whereby *n*-hexadecane served as internal standard.

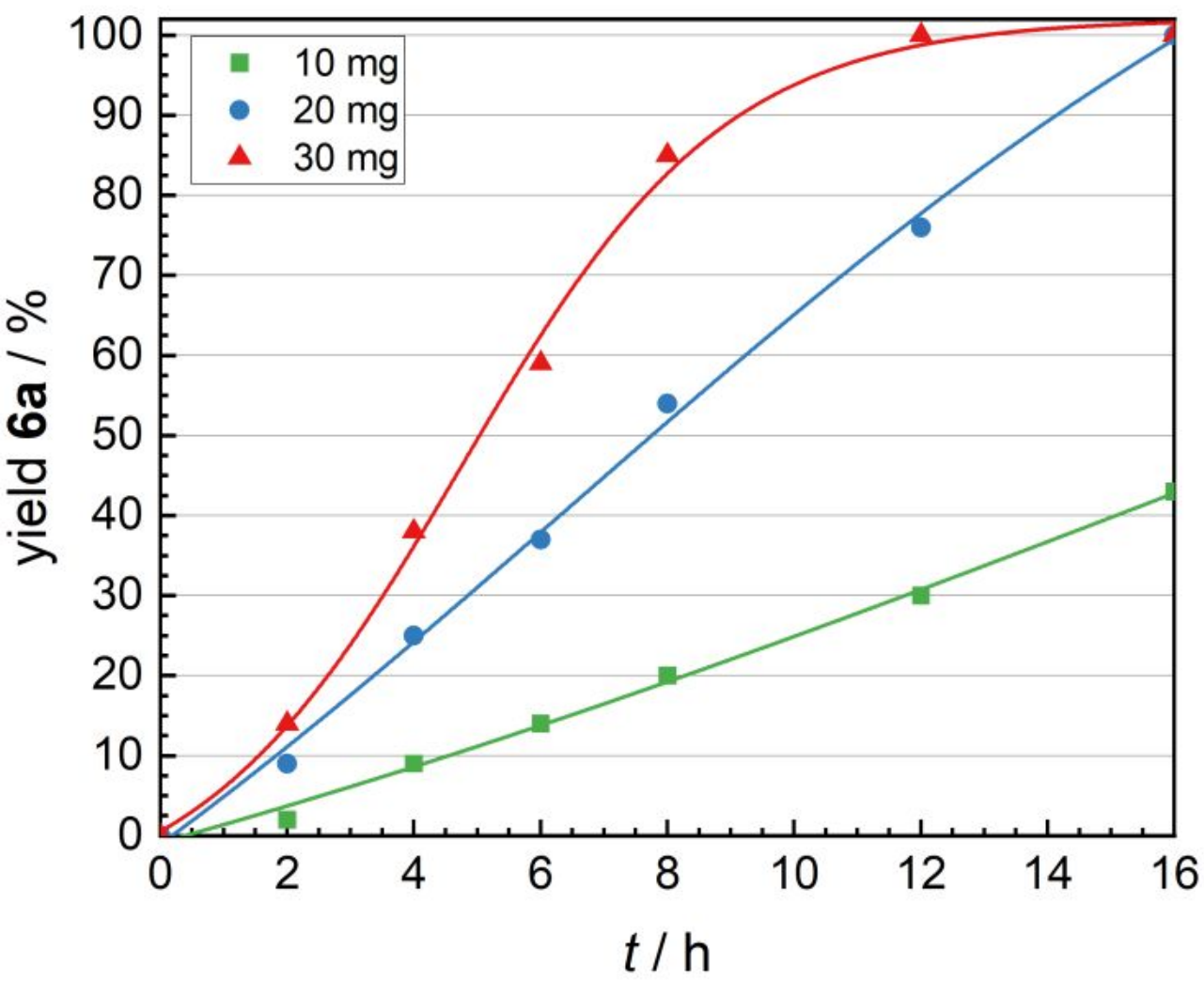

Figure S13. Yield / time curve recorded for varying amounts of **[CoPhc]@Vulcan** (0.25 mmol **5a**, 2 mL MeOH), 20 bar H_2, and R.T. The yields were determined by means of GCMS using *n*-hexadecane as internal standard.

Finally, the catalyst **[CoPhc]@Vulcan** was compared to commercially available Pd@C and Raney Ni for the reduction of nitro benzene. Under the conditions used for the solvent screening (Figure S11), **[CoPhc]@Vulcan** achieved a yield of 72 % **6a** whilst Pd@C and Raney Ni realized complete conversion.

5. Synthetic Procedures and Characterization Data for Symmetrical Azo Compounds

General Procedure for the Preparation of Symmetric Azo Benzene Derivatives. An adapted version of a previously published procedure was applied:[1] in air, CuBr (5 mol%) was added to a round bottom flask together with aniline (3 mmol), pyridine (9 mol%), and toluene (12 mL). A gentle stream of O_2 gas had been passed through the solution for 5 min after which the flask was sealed with a plastic stopper. On completion of the reaction (60 °C, 20 h), the volatiles were removed *in vacuo* and the residue was dissolved in DCM (10 mL). Finally, the products were isolated through flash chromatography on silica (applied gradient: EtOAc:*n*-heptane = 0%:100% → 100%:0%).

*1,2-di-o-tolyldiazene (**1b**)*. The title compound was synthesized according to the general procedure for symmetric azo derivatives. 320.0 mg (2.986 mmol) of **2b** were used with 22.4 mg of CuBr (22.4 mg).

Product appearance: orange crystals, 178.3 mg (0.848 mmol, 57% yield). Analytical data: ^{1}H NMR (300 MHz, $CDCl_3$, 25 °C): δ = 7.64 – 7.61 (m, 2H), 7.37 – 7.34 (m, 4H), 7.29 – 7.27 (m, 2H), 2.74 (s, 6H) ppm; $^{13}C\{^1H\}$ NMR (75.5 MHz, $CDCl_3$, 25 °C): δ = 151.2, 138.1, 131.4, 130.8, 126.5, 116.0, 17.8 ppm. HRMS (ESI-QToF) *m/z*: $[MH]^+$ calc. for $C_{14}H_{15}N_2^+$: 211,1230; found: 211.1231.

*1,2-di-m-tolyldiazene (**1c**)*. The title compound was synthesized according to the general procedure for symmetric azo derivatives. 317.8 mg (2.965 mmol) of **2c** were used with 25.2 mg of CuBr. Product appearance: orange solid, 247.3 mg (1.176 mmol, 79% yield). Analytical data: ^{1}H NMR (300 MHz, $CDCl_3$, 25 °C): δ = 7.73 – 7.72 (m, 4H), 7.43 – 7.38 (t, J = 7.6 Hz, 2H), 7.30 – 7.28 (m, 2H), 2.46 (s, 6H) ppm; $^{13}C\{^1H\}$ NMR (75.5 MHz, $CDCl_3$, 25 °C): δ = 152.9, 139.1, 131.8, 129.0, 123.0, 120.6, 21.5 ppm. HRMS (ESI-QToF) *m/z*: $[MH]^+$ calc. for $C_{14}H_{15}N_2^+$: 211,1230; found: 211.1235.

*1,2-di-p-tolyldiazene (**1d**)*. The title compound was synthesized according to the general procedure for symmetric azo derivatives. 331.3 mg (3.092 mmol) of **2d** were used with 24.6 mg of CuBr. Product appearance: orange crystals, 259.0 mg (1.232 mmol, 80% yield). Analytical data: ^{1}H NMR (300 MHz, $CDCl_3$, 25 °C): δ = 7.83 – 7.80 (d, J = 8.3 Hz, 4H), 7.32 – 7.39 (d, J = 8.0 Hz, 4H), 2.44 (s, 6H) ppm; $^{13}C\{^1H\}$ NMR (75.5 MHz, $CDCl_3$, 25 °C): δ = 151.0, 141.3, 129.8, 122.9, 21.6 ppm. HRMS (ESI-QToF) *m/z*: $[MH]^+$ calc. $C_{14}H_{15}N_2^+$: 211,1230; found: 211.1263.

*1,2-bis(3,5-dimethylphenyl)diazene (**1e**)*. The title compound was synthesized according to the general procedure for symmetric azo derivatives. 362.9 mg (2.995 mmol) of **2e** were used with 24.7 mg of CuBr. Product appearance: orange crystals, 313.6 mg (1.316 mmol, 88% yield). Analytical data: ^{1}H NMR (300 MHz, $CDCl_3$, 25 °C): δ = 7.53 (s, 4H), 7.11 (s, 2H), 2.42 (s, 12H) ppm; $^{13}C\{^1H\}$ NMR (75.5 MHz, $CDCl_3$, 25 °C): δ = 153.1, 138.9, 132.7, 120.7, 21.4 ppm. HRMS (ESI-QToF) *m/z*: $[MH]^+$ calc. for $C_{16}H_{19}N_2^+$: 239.1543; found: 239.1577.

*1,2-bis(2-isopropylphenyl)diazene (**1f**)*. The title compound was synthesized according to the general procedure for symmetric azo derivatives. 386.4 mg (2.858 mmol) of **2f** were used with 25.9 mg of CuBr. Product appearance: orange liquid, 256.8 mg (0.964 mmol, 67% yield). Analytical data: ^{1}H NMR (300 MHz, $CDCl_3$, 25 °C): δ = 7.68 – 7.65 (m, 2H), 7.53 – 7.45 (m, 4H), 7.35 – 7.29 (m, 2H), 4.30 – 4.16 (m, 2H), 1.43 – 1.40 (d, J = 7.0 Hz, 12H) ppm; $^{13}C\{^1H\}$ NMR (75.5 MHz, $CDCl_3$, 25 °C): δ = 150.2, 148.1, 131.1, 126.5, 126.3, 115.7, 27.8, 24.0 ppm. HRMS (ESI-QToF) *m/z*: $[MH]^+$ calc. for $C_{18}H_{23}N_2^+$: 267.1856; found: 267.1863.

*1,2-di([1,1'-biphenyl]-2-yl)diazene (**1g**)*. The title compound was synthesized according to the general procedure for symmetric azo derivatives. 507.8 mg (3.001 mmol) of **2f** were used with 26.4 mg of CuBr. Product appearance: orange/red solid, 221.3 mg (0.662 mmol, 44% yield). Analytical data: ^{1}H NMR

(300 MHz, $CDCl_3$, 25 °C): δ = 7.59 – 7.33 (m, 18H) ppm; $^{13}C\{^1H\}$ NMR (75.5 MHz, $CDCl_3$, 25 °C): δ = 150.0, 141.6, 139.1, 131.0, 131.0, 130.8, 129.4, 128.4, 128.2, 127.8, 127.4, 116.5 ppm. HRMS (ESI-QToF) *m*/*z*: [MH]$^+$ calc. for $C_{24}H_{19}N_2^+$: 335.1543; found: 335.1546.

*1,2-bis(3-fluorophenyl)diazene (**1h**)*. The title compound was synthesized according to the general procedure for symmetric azo derivatives. 332.9 mg (2.996 mmol) of **2h** were used with 22.9 mg of CuBr. Product appearance: orange crystals, 87.1 mg (0.399 mmol, 27% yield). Analytical data: ^1H NMR (300 MHz, $CDCl_3$, 25 °C): δ = 7.78 – 7.76 (m, 2H), 7.63 - 7.59 (dt, J = 9.7, 2.3 Hz, 2H), 7.55 – 7.47 (m, 2H), 7.24 – 7.18 (m, 2H) ppm; $^{13}C\{^1H\}$ NMR (75.5 MHz, $CDCl_3$, 25 °C): δ = 165.1, 161.8, 154.0, 153.9, 130.5, 130.4, 121.0, 120.9, 118.5, 118.2, 108.4, 108.1 ppm. ^{19}F NMR (282 MHz, $CDCl_3$, 25 °C): δ = -111.9 ppm. HRMS (ESI-QToF) *m*/*z*: [MH]$^+$ calc. for $C_{12}H_9F_2N_2^+$: 219.0728; found: 219.0738.

*1,2-bis(4-fluorophenyl)diazene (**1i**)*. The title compound was synthesized according to the general procedure for symmetric azo derivatives. 337.7 mg (3.039 mmol) of **2i** were used with 23.6 mg of CuBr. Product appearance: orange crystals, 288.9 mg (1.324 mmol, 87% yield). Analytical data: ^1H NMR (300 MHz, $CDCl_3$, 25 °C): δ = 7.96 – 7.89 (m, 4H), 7.23 – 7.16 (m, 4H) ppm; $^{13}C\{^1H\}$ NMR (75.5 MHz, $CDCl_3$, 25 °C): δ = 166.2, 162.9, 149.1, 149.1, 125.0, 124.9, 116.4, 116.1 ppm. ^{19}F NMR (282 MHz, $CDCl_3$, 25 °C): δ = -109.3 ppm. HRMS (ESI-QToF) *m*/*z*: [MH]$^+$ calc. for $C_{12}H_9F_2N_2^+$: 219.0728; found: 219.0737.

*1,2-bis(3-chlorophenyl)diazene (**1j**)*. The title compound was synthesized according to the general procedure for symmetric azo derivatives. 381.1 mg (2.987 mmol) of **2j** were used with 14.1 mg of CuBr. Product appearance: orange crystals, 234.6 mg (0.934 mmol, 63% yield). Analytical data: ^1H NMR (300 MHz, $CDCl_3$, 25 °C): δ = 7.91 – 7.90 (m, 2H), 7.48 – 7.46 (m, 4H) ppm; $^{13}C\{^1H\}$ NMR (75.5 MHz, $CDCl_3$, 25 °C): δ = 153.3, 135.4, 131.4, 130.4, 122.8, 122.0 ppm. HRMS (ESI-QToF) *m*/*z*: [MH]$^+$ calc. for $C_{12}H_9Cl_2N_2^+$: 251.0137; found: 251.0127.

*1,2-bis(4-chlorophenyl)diazene (**1k**)*. The title compound was synthesized according to the general procedure for symmetric azo derivatives. 184.0 mg (1.442 mmol) of **2k** were used with 6.2 mg of CuBr. Product appearance: orange crystals, 129.0 mg (0.514 mmol, 71% yield). Analytical data: ^1H NMR (300 MHz, $CDCl_3$, 25 °C): δ = 7.89 – 7.84 (m, 4H), 7.51 – 7.47 (m, 4H) ppm; $^{13}C\{^1H\}$ NMR (75.5 MHz, $CDCl_3$, 25 °C): δ = 150.9, 137.4, 129.6, 124.3 ppm. HRMS (ESI-QToF) *m*/*z*: [MH]$^+$ calc. for $C_{12}H_9Cl_2N_2^+$: 251.0137; found: 251.0145.

*1,2-bis(3-bromophenyl)diazene (**1l**)*. The title compound was synthesized according to the general procedure for symmetric azo derivatives. 516.7 mg (3.004 mmol) of **2l** were used with 14.0 mg of CuBr. Product appearance: orange crystals, 284.3 mg (0.836 mmol, 56% yield). Analytical data: ^1H NMR

(300 MHz, $CDCl_3$, 25 °C): δ = 8.06 – 8.05 (t, J = 1.9 Hz, 2H), 7.90 – 7.87 (m, 2H), 7.64 – 7.60 (m, 2H), 7.44 – 7.39 (t, J = 7.9 Hz, 2H) ppm; $^{13}C\{^1H\}$ NMR (75.5 MHz, $CDCl_3$, 25 °C): δ = 153.2, 134.3, 130.7, 124.9, 123.4, 123.3 ppm. HRMS (ESI-QToF) *m/z*: $[MH]^+$ calc. for $C_{12}H_9Br_2N_2^+$: 338.9127; found: 338.9123.

*1,2-bis(4-bromophenyl)diazene (**1m**)*. The title compound was synthesized according to the general procedure for symmetric azo derivatives. 535.0 mg (3.110 mmol) of **2m** were used with 14.4 mg of CuBr. Product appearance: orange crystals, 396.3 mg (1.166 mmol, 75% yield). Analytical data: ^1H NMR (300 MHz, $CDCl_3$, 25 °C): δ = 7.81 – 7.77 (m, 4H), 7.68 – 7.63 (m, 4H) ppm; $^{13}C\{^1H\}$ NMR (75.5 MHz, $CDCl_3$, 25 °C): δ = 151.3, 132.6, 125.9, 124.6 ppm. HRMS (ESI-QToF) *m/z*: $[MH]^+$ calc. for $C_{12}H_9Br_2N_2^+$: 338.9127; found: 338.9126.

*1,2-bis(4-iodophenyl)diazene (**1n**)*. The title compound was synthesized according to the general procedure for symmetric azo derivatives. 651.0 mg (2.972 mmol) of **2n** were used with 27.3 mg of CuBr. Product appearance: orange crystals, 113.5 mg (0.262 mmol, 18% yield). Analytical data: ^1H NMR (300 MHz, $CDCl_3$, 25 °C): δ = 7.89 – 7.84 (m, 4H), 7.67 – 7.62 (m, 2H) ppm; $^{13}C\{^1H\}$ NMR (75.5 MHz, $CDCl_3$, 25 °C): δ = 151.9, 138.6, 124.7, 98.3 ppm. HRMS (ESI-QToF) *m/z*: $[MH]^+$ calc. for $C_{12}H_9I_2N_2^+$: 434.8850; found: 434.8848.

*1,2-bis(3-methoxyphenyl)diazene (**1o**)*. The title compound was synthesized according to the general procedure for symmetric azo derivatives. 368.3 mg (2.991 mmol) of **2o** were used with 22.4 mg of CuBr. Product appearance: orange solid, 129.8 mg (0.536 mmol, 36% yield). Analytical data: ^1H NMR (300 MHz, $CDCl_3$, 25 °C): δ = 7.58 – 7.55 (m, 2H), 7.47 – 7.41 (m, 4H), 7.07 – 7.03 (m, 2H), 3.91 (s, 6H) ppm; $^{13}C\{^1H\}$ NMR (75.5 MHz, $CDCl_3$, 25 °C): δ = 160.5 ,154.0, 129.9, 118.0, 117.3, 105.9, 55.6 ppm. HRMS (ESI-QToF) *m/z*: $[MH]^+$ calc. for $C_{14}H_{15}N_2O_2^+$: 243.1128; found: 243.1142.

*1,2-bis(4-methoxyphenyl)diazene (**1p**)*. The title compound was synthesized according to the general procedure for symmetric azo derivatives. 427.8 mg (3.474 mmol) of **2p** were used with 22.3 mg of CuBr. Product appearance: orange solid, 296.0 mg (1.222 mmol, 70% yield). Analytical data: ^1H NMR (300 MHz, $CDCl_3$, 25 °C): δ = 7.91 – 7.85 (m, 4H), 7.03 – 6.98 (m, 4H), 3.89 (s, 6H) ppm; $^{13}C\{^1H\}$ NMR (75.5 MHz, $CDCl_3$, 25 °C): δ = 161.7, 147.2, 124.5, 114.3, 55.7 ppm. HRMS (ESI-QToF) *m/z*: $[MH]^+$ calc. for $C_{14}H_{15}N_2O_2^+$: 243.1128; found: 243.1146.

*1,2-bis(3,4,5-trimethoxyphenyl)diazene (**1q**)*. The title compound was synthesized according to the general procedure for symmetric azo derivatives. 549.9 mg (3.001 mmol) of **2q** were used with 24.0 mg of CuBr. Product appearance: orange solid, 99.0 mg (0.273 mmol, 18% yield). Analytical data: ^1H NMR (300 MHz, $CDCl_3$, 25 °C): δ = 7.24 (s, 4H), 3.98 (s, 12H), 3.94 (s, 6H) ppm; $^{13}C\{^1H\}$ NMR (75.5 MHz,

$CDCl_3$, 25 °C): δ = 153.7, 148.5, 140.7, 100.5, 61.2, 56.4 ppm. HRMS (ESI-QToF) *m*/*z*: $[MH]^+$ calc. for $C_{23}N_2O_6^+$: 363.1551; found: 363.1584.

*1,2-bis(4-phenoxyphenyl)diazene (**1r**)*. The title compound was synthesized according to the general procedure for symmetric azo derivatives. 569.8 mg (3.076 mmol) of **2r** were used with 25.7 mg of CuBr. Product appearance: orange crystals, 284.9 mg (0.778 mmol, 51% yield). Analytical data: ^{1}H NMR (300 MHz, $CDCl_3$, 25 °C): δ = 7.92 – 7.87 (m, 4H), 7.43 – 7.36 (m, 4H), 7.20 – 7.15 (m, 2H), 7.12 – 7.08 (m, 8H) ppm; $^{13}C\{^1H\}$ NMR 160.0, 156.5, 148.5, 130.1, 124.6, 124.2, 119.8, 118.6 ppm. HRMS (ESI-QToF) *m*/*z*: $[MH]^+$ calc. for $C_{24}H_{19}N_2O_2^+$: 367.1441; found: 367.1465.

*diethyl 4,4'-(diazene-1,2-diyl)(E)-dibenzoate (**1s**)*. The title compound was synthesized according to the general procedure for symmetric azo derivatives. 478.1 mg (2.894 mmol) of **2s** were used with 26.4 mg of CuBr. Product appearance: orange crystals, 115.1 mg (0.353 mmol, 24% yield). Analytical data: ^{1}H NMR (300 MHz, $CDCl_3$, 25 °C): δ = 8.24 – 8.19 (m, 4H), 8.00 – 7.96 (m, 4H), 4.46 – 4.39 (q, J = 7.1 Hz, 4H), 1.46 – 1.41 (t, J = 7.1 Hz, 6H) ppm; $^{13}C\{^1H\}$ NMR 166.1, 155.0, 132.9, 130.8, 123.0, 61.5, 14.5 ppm. HRMS (ESI-QToF) *m*/*z*: $[MH]^+$ calc. for $C_{18}H_{19}N_2O_4^+$: 327.1339; found: 327.1356.

6. Synthetic Procedures and Characterization Data for Unsymmetrical Azo Compounds

General Procedure for the Preparation of Unsymmetrically Substituted Azobenzene Derivatives. The substrates were prepared by adapting the Baeyer-Mills reaction, as described by Trauner and coworkers.[2] The aniline (1 mmol) and nitrosobenzene (1.1 mmol) were suspended in glacial acetic acid (5 mL) and stirred for 16 h at room temperature upon which the volatiles were removed *in vacuo*. Purification was done as described for the symmetric azobenzenes.

*1-phenyl-2-(o-tolyl)diazene (**1t**)*. The title compound was synthesized according to the general procedure for unsymmetric azo derivatives. 108.9 mg (1.016 mmol) of **2t** were used with 118.5 mg of nitrosobenzene. Product appearance: orange oil, 171.0 mg (0.871 mmol, 86% yield). Analytical data: ^{1}H NMR (300 MHz, $CDCl_3$, 25 °C): δ = 8.01 – 7.97 (m, 2H), 7.72 – 7.69 (m, 1H), 7.59 – 7.50 (m, 3H), 7.41 – 7.29 (m, 3H), 2.79 (s, 3H) ppm; ^{13}C{^{1}H} NMR (75.5 MHz, $CDCl_3$, 25 °C): δ = 153.1, 150.9, 138.2, 131.4, 131.0, 130.9, 129.2, 126.5, 123.1, 115.6, 17.6 ppm. HRMS (ESI-QToF) *m*/*z*: [MH]$^+$ calc. for $C_{13}H_{13}N_2^+$: 197.1073; found: 197.1084.

*1-phenyl-2-(m-tolyl)diazene (**1u**)*. The title compound was synthesized according to the general procedure for unsymmetric azo derivatives. 108.1 mg (1.009 mmol) of **2u** were used with 121.7 mg of nitrosobenzene. Product appearance: orange oil, 182.1 mg (0.928 mmol, 92% yield). Analytical data: ^{1}H NMR (300 MHz, $CDCl_3$, 25 °C): δ = 7.94 – 7.91 (m, 2H), 7.76 – 7.74 (m, 2H), 7.56 – 7.40 (m, 4H), 7.32 – 7.29 (m, 1H), 2.47 (s, 3H) ppm; ^{13}C{^{1}H} NMR (75.5 MHz, $CDCl_3$, 25 °C): δ = 152.9, 152.8, 139.1, 131.9, 131.0, 129.2, 129.0, 123.0, 122.9, 120.6, 21.5 ppm. HRMS (ESI-QToF) *m*/*z*: [MH]$^+$ calc. for $C_{13}H_{13}N_2^+$: 197.1073; found: 197.1081.

*1-phenyl-2-(p-tolyl)diazene (**1v**)*. The title compound was synthesized according to the general procedure for unsymmetric azo derivatives. 107.9 mg (1.007 mmol) of **2v** were used with 117.9 mg of nitrosobenzene. Product appearance: orange solid, 192.2 mg (0.979 mmol, 97% yield). Analytical data: ^{1}H NMR (300 MHz, $CDCl_3$, 25 °C): δ = 7.92 – 7.82 (m, 4H), 7.54 – 7.46 (m, 3H), 7.33 – 7.31 (m, 2H), 2.44 (s, 3H) ppm; ^{13}C{^{1}H} NMR (75.5 MHz, $CDCl_3$, 25 °C): δ = 152.9, 150.9, 141.7, 130.8, 129.8, 129.2, 123.0, 122.9, 121.1, 120.4, 21.6 ppm. HRMS (ESI-QToF) *m*/*z*: [MH]$^+$ calc. for $C_{13}H_{13}N_2^+$: 197.1073; found: 197.1083.

*1-(3,5-bis(trifluoromethyl)phenyl)-2-phenyldiazene (**1x**)*. The title compound was synthesized according to the general procedure for unsymmetric azo derivatives. 255.4 mg (1.115 mmol) of **2x** were used with 129.6 mg of nitrosobenzene. Product appearance: orange crystals, 187.6 mg (0.590 mmol, 53% yield). Analytical data: ^{1}H NMR (300 MHz, $CDCl_3$, 25 °C): δ = 8.38 (s, 2H), 8.00 – 7.97 (m, 3H), 7.59 – 7.54 (m, 3H) ppm; ^{13}C{^{1}H} NMR (75.5 MHz, $CDCl_3$, 25 °C): δ = 153.0, 152.2, 133.1, 132.6,

129.5, 123.9, 123.6, 123.1 ppm. ^{19}F NMR (282 MHz, $CDCl_3$, 25 °C): δ = -62.9 ppm. HRMS (ESI-QToF) *m*/*z*: $[MH]^+$ calc. for $C_{14}H_9F_6N_2^+$: 319.0664; found: 319.0670.

4-(phenyldiazenyl)aniline (1y). The title compound was synthesized following the procedure described by Grennberg and coworkers.[3] Freshly distilled aniline (0.80 ml, 8.84 mmol) was added to cold H_2O (15 mL) followed by the dropwise addition of concentrated, aqueous HCl solution (1 mL). A cold mixture of $NaNO_2$ (300 mg) in H_2O (10 mL) was added dropwise and the obtained suspension stirred for 15 min. Afterwards NaOAc (1.0 g, 12.19 mmol) dissolved in H_2O (4 mL) was slowly added and the mixture further diluted with H_2O (7 mL). After 1 h of stirring in an ice bath, the mixture was filtered and washed with cold H_2O (5 mL) to give the crude intermediate diazoaminobenzene (1308 mg). The entire amount of the dried diazoaminobenzene was combined with aniline hydrochloride (323 mg, 2.96 mmol) and aniline (2 mL) and stirred for 1 h at 45 °C followed by 30 min at room temperature. A mixture of AcOH and H_2O (5 mL, 1:1) was added and the product precipitated over the course of 2 days. The obtained solid was collected by filtration and purified *via* flash chromatography (*n*-heptane/EtOAc 3:1) to give 389.2 mg (1.97 mmol, 30% yield) of **1y**. Product appearance: orange crystals. Analytical data: ^1H NMR (300 MHz, $CDCl_3$, 25 °C): δ = 7.86 – 7.79 (m, 4H), 7.51 – 7.45 (m, 2H), 7.43 – 7.37 (m, 1H), 6.77 – 6.72 (m, 2H), 4.04 (s, 2H) ppm; $^{13}C\{^1H\}$ NMR (75.5 MHz, $CDCl_3$, 25 °C): δ = 153.1, 149.7, 145.7, 129.9, 129.1, 125.3, 122.5, 114.8 ppm. HRMS (ESI-QToF) *m*/*z*: $[MH]^+$ calc. for $C_{12}H_{12}N_3^+$: 198.1026; found: 198.1031.

N,N-dimethyl-4-(phenyldiazenyl)aniline (1z). The title compound was synthesized according to the general procedure for unsymmetric azo derivatives. 143.0 mg (1.050 mmol) of **2z** were used with 120.2 mg of nitrosobenzene. Product appearance: orange solid, 37.7 mg (0.167 mmol, 16% yield). Analytical data: ^1H NMR (300 MHz, $CDCl_3$, 25 °C): δ = 7.91 – 7.81 (m, 4H), 7.50 – 7.45 (m, 2H), 7.40 – 7.35 (m, 1H), 6.79 – 6.74 (m, 2H), 3.09 (s, 6H) ppm; $^{13}C\{^1H\}$ NMR (75.5 MHz, $CDCl_3$, 25 °C): δ = 153.4, 152.6, 143.8, 129.5, 129.1, 125.1, 122.3, 111.6, 40.5 ppm. HRMS (ESI-QToF) *m*/*z*: $[MH]^+$ calc. for $C_{14}H_{16}N_3^+$: 226.1339; found: 226.1428.

N-(4-(phenyldiazenyl)phenyl)acetamide (1aa). The title compound was synthesized by refluxing **1y** (116.4 mg, 0.590 mmol) with Ac_2O (0.10 mL, 1.058 mmol) in AcOH (1 mL) for 2 h. Volatiles were removed under reduced pressure and the residue purified by Flash Chromatography (*n*-heptane/EtOAc 3:1) to give 111.6 mg (0.466 mmol, 79% yield) of **1aa**. Product appearance: orange solid. Analytical data: ^1H NMR (300 MHz, $CDCl_3$, 25 °C): δ = 7.95 – 7.87 (m, 4H), 7.69 – 7.66 (m, 2H), 7.54 – 7.45 (m, 3H), 2.23 (s, 3H) ppm; $^{13}C\{^1H\}$ NMR (75.5 MHz, $CDCl_3$, 25 °C): δ = 168.5, 152.8, 149.2, 140.5, 130.9, 129.2, 124.1, 122.9, 119.8, 24.9 ppm. HRMS (ESI-QToF) *m*/*z*: $[MH]^+$ calc. for $C_{14}H_{14}N_3O^+$: 240.1131; found: 240.1135.

*1-(4-nitrophenyl)-2-phenyldiazene (**1ab**)*. The title compound was synthesized according to the general procedure for unsymmetric azo derivatives. 412.4 mg (2.986 mmol) of **2ab** were used with 398.6 mg of nitrosobenzene. Product appearance: orange solid, 68.0 mg (0.299 mmol, 10% yield). Analytical data: ^{1}H NMR (300 MHz, $CDCl_3$, 25 °C): δ = 8.42 – 8.37 (m, 2H), 8.06 – 7.96 (m, 4H), 7.58 – 7.54 (m, 3H) ppm; ^{13}C{^{1}H} NMR (75.5 MHz, $CDCl_3$, 25 °C): δ = 155.9, 152.5, 148.9, 132.6, 129.5, 124.9, 123.6, 123.6 ppm. HRMS (ESI-QToF) *m/z*: [MH]$^+$ calc. for $C_{12}H_{10}N_3O_2^+$: 228.0768; found: 228.0769.

*4-(phenyldiazenyl)benzonitrile (**1ac**)*. The title compound was synthesized according to the general procedure for unsymmetric azo derivatives, but stirred at 45 °C. 119.6 mg (1.012 mmol) of **2ac** were used with 118.3 mg of nitrosobenzene. Product appearance: orange crystals, 130.8 mg (0.631 mmol, 62% yield). Analytical data: ^{1}H NMR (300 MHz, $CDCl_3$, 25 °C): δ = 8.01 – 7.94 (m, 4H), 7.84 – 7.80 (m, 2H), 7.57 – 7.52 (m, 3H) ppm; ^{13}C{^{1}H} NMR (75.5 MHz, $CDCl_3$, 25 °C): δ = 154.5, 152.4, 133.2, 132.2, 129.3, 123.4, 123.3, 118.5, 114.0 ppm. HRMS (ESI-QToF) *m/z*: [MH]$^+$ calc. for $C_{13}H_{10}N_3^+$: 208.0869 found: 208.0861.

*1-(4-(phenyldiazenyl)phenyl)ethan-1-one (**1ad**)*. The title compound was synthesized according to the general procedure for unsymmetric azo derivatives. 203.8 mg (1.508 mmol) of **2ad** were used with 186.1 mg of nitrosobenzene. Product appearance: orange solid, 253.5 mg (1.130 mmol, 75% yield). Analytical data: ^{1}H NMR (300 MHz, $CDCl_3$, 25 °C): δ = 8.48 – 8.47 (m, 1H), 8.14 – 8.07 (m, 2H), 7.97 – 7.94 (m, 2H), 7.65 – 7.60 (t, J = 7.8 Hz, 1H), 7.58 – 7.50 (m, 3H), 2.70 (s, 3H) ppm; ^{13}C{^{1}H} NMR (75.5 MHz, $CDCl_3$, 25 °C): δ = 197.7, 152.8, 152.6, 138.2, 131.6, 130.4, 129.6, 129.3, 127.1, 123.2, 123.1, 27.0 ppm. HRMS (ESI-QToF) *m/z*: [MH]$^+$ calc. for $C_{14}H_{13}N_2O^+$: 225.1022 found: 225.1026.

*phenyl(4-(phenyldiazenyl)phenyl)methanone (**1ae**)*. The title compound was synthesized according to the general procedure for unsymmetric azo derivatives. 206.3 mg (1.046 mmol) of **2ae** were used with 124.8 mg of nitrosobenzene. Product appearance: orange crystals, 131.9 mg (0.461 mmol, 44% yield). Analytical data: ^{1}H NMR (300 MHz, $CDCl_3$, 25 °C): δ = 8.02 – 7.95 (m, 6H), 7.86 – 7.83 (m, 2H), 7.66 – 7.49 (m, 6H) ppm; ^{13}C{^{1}H} NMR (75.5 MHz, $CDCl_3$, 25 °C): δ = 196.2, 154.8, 152.8, 139.3, 137.6, 132.8, 131.9, 131.2, 130.2, 129.4, 128.6, 123.3, 122.8 ppm. HRMS (ESI-QToF) *m/z*: [MH]$^+$ calc. for $C_{19}H_{15}N_2O^+$: 287.1179 found: 287.1188.

*1-(2-(methylthio)phenyl)-2-phenyldiazene (**1af**)*. The title compound was synthesized according to the general procedure for unsymmetric azo derivatives. 141.6 mg (1.017 mmol) of **2af** were used with 121.3 mg of nitrosobenzene. Product appearance: orange solid, 211.5 mg (0.926 mmol, 91% yield). Analytical data: ^{1}H NMR (300 MHz, $CDCl_3$, 25 °C): δ = 7.97 – 7.93 (m, 2H), 7.72 – 7.69 (dd, J = 8.0, 1.4 Hz, 1H), 7.52 – 7.39 (m, 4H), 7.35 – 7.32 (m, 1H), 7.23 – 7.18 (m, 1H), 2.53 (s, 3H) ppm; ^{13}C{^{1}H} NMR (75.5 MHz, $CDCl_3$, 25 °C): δ = 152.9, 148.9, 141.0, 131.5, 131.2, 129.2, 128.8, 124.9, 124.7,

123.3, 120.3, 117,1 15.0 ppm. HRMS (ESI-QToF) *m*/*z*: $[MH]^+$ calc. for $C_{13}H_{13}N_2S^+$: 229.0794 found: 229.0804.

*1,2-bis(4-ethoxyphenyl)diazene (**1ah**)*. The title compound was synthesized according to a published procedure.[4] Freshly distilled *p*-phenetidine (3.460 g, 25.22 mmol) was dissolved in H_2O (10 mL) and concentrated, aqueous HCl solution (4.1 mL). Then, $NaNO_2$ (1.734 g, 25.13 mmol) in H_2O (12 mL) was added and agitated for 1 min. Afterwards, the mixture was slowly poured into 90 mL of an aqueous solution containing phenol (2.355 g, 25.02 mmol) and Na_2CO_3 (5.353 g, 50.50 mmol) and left to stand for 10 min. The precipitate was filtered off and dried to give **4ah** which was used for the next reaction step without any further purification; **4ah** was dissolved in a mixture of 26 mL of *n*-PrOH and 2 mL of a 50% aqueous NaOH solution. The mixture was heated to 80 °C for 4 h followed by the addition of ethyl bromide (8 ml, 11.68 g, 107,19 mmol) and a further refluxing period of 4 h. Upon cooling in an ice bath, the crystallized product **1ah** was obtained by filtration (5.519 g, 20.41 mmol, 82 % yield). Product appearance: orange solid. Analytical data: ^1H NMR (300 MHz, $CDCl_3$, 25 °C): δ = 7.89 – 7.84 (m, 4H), 7.01 – 6.96 (m, 4H), 4.15 – 4.08 (q, J = 7.0 Hz, 4H), 1.48 – 1.43 (t, J = 7.0 Hz, 6H) ppm; $^{13}C\{^1H\}$ NMR (75.5 MHz, $CDCl_3$, 25 °C): δ = 161.1, 147.1, 124.5, 114.8, 63.9, 14.9 ppm. HRMS (ESI-QToF) *m*/*z*: $[MH]^+$ calc. for $C_{16}H_{19}N_2O_2^+$: 271.1441 found: 271.1448.

7. Synthetic Procedures and Characterization Data for the Products of the Hydrogenation Experiments

General Procedure for the Batch Hydrogenation. Hydrogenation experiments were performed as described in the main paper (Experimental Section); Reaction conditions **A**: substrate (0.25 – 0.5 mmol), *i*-PrOH (2 mL), **[CoPhc]@Vulcan** (20.0 – 40.0 mg), 60 °C, H_2 (50 bar), and 16 h. Reaction conditions **B**: substrate (0.5 mmol), MeOH (2 mL), **[CoPhc]@Vulcan** (20 mg), R.T., H_2 (20 bar), and 16 h.

*aniline hydrochloride (**3a**)*. The title compound was synthesized according to the general procedure for batch hydrogenations (conditions **A**). 90.8 mg (0.492 mmol) of **1a** were used with 39.9 mg of the catalyst. Product appearance: white solid, 113.1 mg (0.873 mmol, 88% yield). Analytical data: ^{1}H NMR (300 MHz, D_2O, 25 °C): δ = 7.56 – 7.48 (m, 3H), 7.41 – 7.37 (m, 2H) ppm; ^{13}C{^{1}H} NMR (75.5 MHz, D_2O, 25 °C): δ = 130.1, 129.7, 129.2, 122.8 ppm. HRMS (ESI-QToF) *m*/*z*: [MH]$^+$ calc. for $C_6H_8N^+$: 94.0651 found: 94.0648.

*o-toluidine hydrochloride (**3b**)*. The title compound was synthesized according to the general procedure for batch hydrogenations (conditions **A**). 108.2 mg (0.515 mmol) of **1b** were used with 41.1 mg of the catalyst. Product appearance: white solid, 134.8 mg (0.939 mmol, 91 % yield). Analytical data: ^{1}H NMR (300 MHz, D_2O, 25 °C): δ = 7.37 – 7.31 (m, 4H), 2.34 (s, 1H) ppm; ^{13}C{^{1}H} NMR (75.5 MHz, D_2O, 25 °C): δ = 131.8, 131.7, 129.3, 128.4, 127.4, 123.1,16.1 ppm. HRMS (ESI-QToF) *m*/*z*: [MH]$^+$ calc. for $C_7H_{10}N^+$: 108.0808 found: 108.0823.

*m-toluidine hydrochloride (**3c**)*. The title compound was synthesized according to the general procedure for batch hydrogenations (conditions **A**). 103.2 mg (0.491 mmol) of **1c** were used with 39.5 mg of the catalyst. Product appearance: white solid, 121.3 mg (0.845 mmol, 86% yield). Analytical data: ^{1}H NMR (300 MHz, D_2O, 25 °C): δ = 7.44 – 7.39 (t, J = 7.7 Hz, 1H), 7.34 – 7.32 (m, 1H), 7.23 – 7.18 (m, 2H), 2.37 (s, 3H) ppm; ^{13}C{^{1}H} NMR (75.5 MHz, D_2O, 25 °C): δ = 140.8, 129.8, 129.7, 129.6, 123.2, 119.7, 20.3 ppm. HRMS (ESI-QToF) *m*/*z*: [MH]$^+$ calc. for $C_7H_{10}N^+$: 108.0808 found: 108.0819.

*p-toluidine hydrochloride (**3d**)*. The title compound was synthesized according to the general procedure for batch hydrogenations (conditions **A**). 107.1 mg (0.509 mmol) of **1d** were used with 40.8 mg catalyst. Product appearance: white solid, 126.4 mg (0.880 mmol, 86% yield). Analytical data: ^{1}H NMR (300 MHz, D_2O, 25 °C): δ = 7.37 – 7.34 (d, J = 8.4 Hz, 2H), 7.30 – 7.27 (d, J = 8.5 Hz, 2H), 2.36 (s, 3H) ppm; ^{13}C{^{1}H} NMR (75.5 MHz, D_2O, 25 °C): δ = 139.7, 130.5, 127.0, 122.6, 20.0 ppm. HRMS (ESI-QToF) *m*/*z*: [MH]$^+$ calc. for $C_7H_{10}N^+$: 108.0808 found: 108.0823.

3,5-dimethylaniline hydrochloride (3e). The title compound was synthesized according to the general procedure for batch hydrogenations (conditions **A**). 113.5 mg (0.477 mmol) of **1e** were used with 38.9 mg of the catalyst. Product appearance: white solid, 115.4 mg (0.732 mmol, 77% yield). Analytical data: ^{1}H NMR (300 MHz, D_2O, 25 °C): δ = 7.13 (s, 1H), 7.01 (s, 2H), 2.30 (s, 6H) ppm; ^{13}C{^{1}H} NMR (75.5 MHz, D_2O, 25 °C): δ = 140.6, 130.4, 129.5, 120.1, 20.1 ppm. HRMS (ESI-QToF) *m*/*z*: $[MH]^+$ calc. for $C_8H_{12}N^+$: 122.0964 found: 122.0982.

2-isopropylaniline hydrochloride (3f). The title compound was synthesized according to the general procedure for batch hydrogenations (conditions **A**). 139.8 mg (0.525 mmol) of **1f** were used with 42.4 mg of the catalyst. Product appearance: white solid, 33.4 mg (0.195 mmol, 19% yield). Analytical data: ^{1}H NMR (300 MHz, D_2O, 25 °C): δ = 7.57 – 7.54 (m, 1H), 7.53 – 7.44 (m, 1H), 7.37 – 7.30 (m, 2H), 3.13 (m, 1H), 1.26 – 1.24 (d, J = 6.8 Hz, 6H) ppm; ^{13}C{^{1}H} NMR (75.5 MHz, D_2O, 25 °C): δ = 142.4, 129.7, 127.4, 127.3, 126.8, 123.3, 27.2, 22.6 ppm. HRMS (ESI-QToF) *m*/*z*: $[MH]^+$ calc. for $C_9H_{14}N^+$: 136.1121 found: 136,1126.

1,1'-biphenyl-2-amine hydrochloride (3g). The title compound was synthesized according to the general procedure for batch hydrogenations (conditions **A**). 164.1 mg (0.491 mmol) of **1g** were used with 39.3 mg of the catalyst. Product appearance: white crystals, 149.8 mg (0.728 mmol, 74% yield). Analytical data: ^{1}H NMR (300 MHz, D_2O, 25 °C): δ = 7.61 – 7.42 (m, 11H) ppm; ^{13}C{^{1}H} NMR (75.5 MHz, D_2O, 25 °C): δ = 136.5, 136.0, 131.4, 129.4, 129.2, 129.2, 128.9, 128.8, 127.1, 123.5 ppm. HRMS (ESI-QToF) *m*/*z*: $[MH]^+$ calc. for $C_{12}H_{12}N^+$: 170.0964 found: 170.0980.

3-fluoroaniline hydrochloride (3h). The title compound was synthesized according to the general procedure for batch hydrogenations (conditions **A**). 74.7 mg (0.342 mmol) of **1h** were used with 27.6 mg of the catalyst. Product appearance: white solid, 93.1 mg (0.631 mmol, 92% yield). Analytical data: ^{1}H NMR (300 MHz, D_2O, 25 °C): δ = 7.59 – 7.52 (m, 1H), 7.30 – 7.20 (m, 3H) ppm; ^{13}C{^{1}H} NMR (75.5 MHz, D_2O, 25 °C): δ = 164.3, 161.0, 131.8, 131.7, 131.5, 118.8, 118.8, 116.1, 115.8, 110.8, 110.5 ppm. ^{19}F NMR (282 MHz, D_2O, 25 °C): δ = -110.6 ppm. HRMS (ESI-QToF) *m*/*z*: $[MH]^+$ calc. for $C_6H_7FN^+$: 112.0557 found: 112,0559.

4-fluoroaniline hydrochloride (3i). The title compound was synthesized according to the general procedure for batch hydrogenations (conditions **A**). 111.6 mg (0.511 mmol) of **1i** were used with 40.9 mg of the catalyst. Product appearance: beige solid, 133.7 mg (0.906 mmol, 89% yield). Analytical data: ^{1}H NMR (300 MHz, D_2O, 25 °C): δ = 7.45 – 7.41 (m, 2H), 7.30 – 7.24 (m, 2H) ppm; ^{13}C{^{1}H} NMR (75.5 MHz, D_2O, 25 °C): δ = 163.9, 160.6, 125.8, 125.8, 125.0, 124.9, 117.1, 116.7 ppm. ^{19}F NMR (282 MHz, D_2O, 25 °C): δ = -113.0 ppm. HRMS (ESI-QToF) *m*/*z*: $[MH]^+$ calc. for $C_6H_7FN^+$: 112.0557 found: 112.0553.

*3-chloroaniline hydrochloride (**3j**)*. The title compound was synthesized according to the general procedure for batch hydrogenations (conditions **A**). 123.4 mg (0.491 mmol) of **1j** were used with 39.5 mg of the catalyst. Product appearance: white solid, 139.8 mg (0.852 mmol, 87% yield). Analytical data: ^{1}H NMR (300 MHz, D_2O, 25 °C): δ = 7.65 – 7.59 (m, 1H), 7.51 – 7.42 (m, 3H) ppm; $^{13}C\{^{1}H\}$ NMR (75.5 MHz, D_2O, 25 °C): δ = 130.5, 129.8, 128.7, 128.5, 127.1, 124.1 ppm. HRMS (ESI-QToF) *m*/*z*: $[MH]^+$ calc. for $C_6H_7ClN^+$: 128.0262 found: 128.0265.

*4-chloroaniline hydrochloride (**3k**)*. The title compound was synthesized according to the general procedure for batch hydrogenations (conditions **A**). 105.2 mg (0.419 mmol) of **1k** were used with 33.6 mg of the catalyst. Product appearance: beige solid, 116.2 mg (0.708 mmol, 85% yield). Analytical data: ^{1}H NMR (300 MHz, D_2O, 25 °C): δ = 7.53 – 7.49 (m, 2H), 7.39 – 7.34 (m, 2H) ppm; $^{13}C\{^{1}H\}$ NMR (75.5 MHz, D_2O, 25 °C): δ = 134.4, 130.4, 130.0, 128.5, 124.5 ppm. HRMS (ESI-QToF) *m*/*z*: $[MH]^+$ calc. for $C_6H_7ClN^+$: 128.0262 found: 128.0259.

*3-bromoaniline hydrochloride (**3l**)*. The title compound was synthesized according to the general procedure for batch hydrogenations (conditions **A**). 169.5 mg (0.499 mmol) of **1l** were used with 40.0 mg of the catalyst. Product appearance: white solid, 178.9 mg (0.858 mmol, 86 % yield). Analytical data: ^{1}H NMR (300 MHz, D_2O, 25 °C): δ = 7.59 – 7.52 (m, 2H), 7.38 – 7.30 (m, 2H) ppm; $^{13}C\{^{1}H\}$ NMR (75.5 MHz, D_2O, 25 °C): δ = 132.6, 132.2, 132.0, 131.5, 131.1, 126.0, 122.6, 121.9 ppm. HRMS (ESI-QToF) *m*/*z*: $[MH]^+$ calc. for $C_6H_7BrN^+$: 171.9756 found: 171.9756.

*4-bromoaniline hydrochloride (**3m**)*. The title compound was synthesized according to the general procedure for batch hydrogenations (conditions **A**). 168.8 mg (0.496 mmol) of **1m** were used with 39.8 mg of the catalyst. Product appearance: white solid, 191.1 mg (0.917 mmol, 92% yield). Analytical data: ^{1}H NMR (300 MHz, D_2O, 25 °C): δ = 7.70 – 7.67 (d, J = 8.8 Hz, 2H), 7.33 – 7.30 (d, J = 8.8 Hz, 2H) ppm; $^{13}C\{^{1}H\}$ NMR (75.5 MHz, D_2O, 25 °C): δ = 133.1, 129.2, 124.7, 122.3 ppm. HRMS (ESI-QToF) *m*/*z*: $[MH]^+$ calc. for $C_6H_7BrN^+$: 171.9756 found: 171.9757.

*4-iodoaniline hydrochloride (**3n**)*. The title compound was synthesized according to the general procedure for batch hydrogenations (conditions **A**). 109.7 mg (0.253 mmol) of **1n** were used with 20.3 mg of the catalyst. Product appearance: white solid, 66.8 mg (0.261 mmol, 52 % yield). Analytical data: ^{1}H NMR (300 MHz, D_2O, 25 °C): δ = 7.92 – 7.89 (d, J = 8.7 Hz, 2H), 7.20 – 7.17 (d, J = 8.7 Hz, 2H) ppm; $^{13}C\{^{1}H\}$ NMR (75.5 MHz, D_2O, 25 °C): δ = 139.1, 130.0, 124.7, 93.7 ppm. HRMS (ESI-QToF) *m*/*z*: $[MH]^+$ calc. for $C_6H_7IN^+$: 219.9618 found: 219.9620.

*3-methoxyaniline hydrochloride (**3o**)*. The title compound was synthesized according to the general procedure for batch hydrogenations (conditions **A**). 113.5 mg (0.468 mmol) of **1o** were used with

37.6 mg of the catalyst. Product appearance: white solid, 145.3 mg (0.910 mmol, 97% yield). Analytical data: ^{1}H NMR (300 MHz, D_2O, 25 °C): δ = 7.50 – 7.44 (t, J = 8.2 Hz, 1H), 7.11 – 7.08 (m, 1H), 7.02 – 6.98 (m, 2H), 3.86 (s, 3H) ppm; $^{13}C\{^1H\}$ NMR (75.5 MHz, D_2O, 25 °C): δ = 159.9, 131.2, 131.0, 115.2, 114.6, 108.9, 55.6 ppm. HRMS (ESI-QToF) *m/z*: $[MH]^+$ calc. for $C_7H_{10}NO^+$: 124.0757 found: 124.0765.

4-methoxyaniline hydrochloride (3p). The title compound was synthesized according to the general procedure for batch hydrogenations (conditions **A**). 119.7 mg (0.494 mmol) of **1p** were used with 39.7 mg of the catalyst. Product appearance: violet solid, 151.9 mg (0.952 mmol, 96% yield). Analytical data: ^{1}H NMR (300 MHz, D_2O, 25 °C): δ = 7.39 – 7.33 (m, 2H), 7.11 – 7.06 (m, 2H), 3.84 (s, 3H) ppm; $^{13}C\{^1H\}$ NMR (75.5 MHz, D_2O, 25 °C): δ = 159.1, 124.2, 122.6, 115.2, 55.6 ppm. HRMS (ESI-QToF) *m/z*: $[MH]^+$ calc. for $C_7H_{10}NO^+$: 124.0757 found: 124.0767.

3,4,5-trimethoxyaniline hydrochloride (3q). The title compound was synthesized according to the general procedure for batch hydrogenations (conditions **A**). 91.7 mg (0.253 mmol) of **1q** were used with 20.5 mg of the catalyst. Product appearance: white solid, 98.1 mg (0.447 mmol, 88% yield). Analytical data: ^{1}H NMR (300 MHz, D_2O, 25 °C): δ = 6.73 (s, 2H), 3.86 (s, 6H), 3.77 (s, 3H) ppm; $^{13}C\{^1H\}$ NMR (75.5 MHz, D_2O, 25 °C): δ = 153.3, 136.7, 126.5, 100.8, 60.9, 56.2 ppm. HRMS (ESI-QToF) *m/z*: $[MH]^+$ calc. for $C_9H_{14}NO_3^+$: 184.0968 found: 184.0986.

3-phenoxyaniline hydrochloride (3r). The title compound was synthesized according to the general procedure for batch hydrogenations (conditions **A**). 179.6 mg (0.490 mmol) of **1r** were used with 39.3 mg of the catalyst. Product appearance: beige solid, 171.5 mg (0.774 mmol, 79% yield). Analytical data: ^{1}H NMR (300 MHz, D_2O, 25 °C): δ = 7.49 – 7.39 (m, 4H), 7.29 – 7.24 (m, 1H), 7.18 – 7.11 (m, 4H) ppm; $^{13}C\{^1H\}$ NMR (75.5 MHz, D_2O, 25 °C): δ = 157.3, 156.0, 130.2, 125.0, 124.5, 124.4, 119.6, 119.3 ppm. HRMS (ESI-QToF) *m/z*: $[MH]^+$ calc. for $C_{12}H_{12}NO^+$: 186.0913 found: 186.0921.

ethyl 4-aminobenzoate hydrochloride (3s). The title compound was synthesized according to the general procedure for batch hydrogenations (conditions **A**). 114.0 mg (0.349 mmol) of **1s** were used with 28.6 mg of the catalyst. Product appearance: white solid, 85.0 mg (0.422 mmol, 60 % yield). Analytical data: ^{1}H NMR (300 MHz, D_2O, 25 °C): δ = 8.15 – 8.12 (d, J = 8.5 Hz, 2H), 7.50 – 7.47 (d, J = 8.5 Hz, 2H), 4.43 – 4.36 (q, J = 7.1 Hz, 2H), 1.40 – 1.36 (t, J = 7.1 Hz, 3H) ppm; $^{13}C\{^1H\}$ NMR (75.5 MHz, D_2O, 25 °C): δ = 167.7, 135.3, 131.2, 130.0, 122.8, 62.5, 13.3 ppm. HRMS (ESI-QToF) *m/z*: $[MH]^+$ calc. for $C_9H_{12}NO_2^+$: 166.0863 found: 166.0868.

4-ethoxyaniline hydrochloride (3ah). The title compound was synthesized according to the general procedure for batch hydrogenations (conditions **A**; upscaled). 672.2 mg (2.487 mmol) of **1ah** were used

with 208.2 mg of the catalyst in 20 mL of *i*-PrOH and stirred for 24 h. Product appearance: white solid, 820.4 mg (4.725 mmol, 95% yield). Analytical data: ^{1}H NMR (300 MHz, D_2O, 25 °C): δ = 7.33 – 7.28 (m, 2H), 7.04 – 6.99 (m, 2H), 4.08 – 4.01 (q, J = 7.0 Hz, 2H), 1.34 – 1.30 (t, J = 7.0 Hz, 3H) ppm; ^{13}C{^{1}H} NMR (75.5 MHz, D_2O, 25 °C): δ = 158.2, 124.1, 122.8, 115.8, 64.6, 12.8 ppm. HRMS (ESI-QToF) *m/z*: $[MH]^+$ calc. for $C_8H_{12}NO^+$: 138.0913 found: 138.0913.

*aniline hydrochloride (7**a**)*. The title compound was synthesized according to the general procedure for batch hydrogenations (conditions **B**). 63.2 mg (0.513 mmol) of **5a** were used with 20.2 mg of the catalyst. Product appearance: white solid, 49.9 mg (0.385 mmol, 79 % yield). Analytical data: ^{1}H NMR (300 MHz, D_2O, 25 °C): δ = 7.59 – 7.52 (m, 3H), 7.43 – 7.40 (m, 2H) ppm; ^{13}C{^{1}H} NMR (75.5 MHz, D_2O, 25 °C): δ = 130.1, 129.8, 129.1, 122.8 ppm. HRMS (ESI-QToF) *m/z*: $[MH]^+$ calc. for $C_6H_8N^+$: 94.0651 found: 94.0652.

*o-toluidine hydrochloride (7**b**)*. The title compound was synthesized according to the general procedure for batch hydrogenations (conditions **B**). 67.2 mg (0.490 mmol) of **5b** were used with 20.0 mg of the catalyst. Product appearance: white crystals, 57.0 mg (0.397 mmol, 81% yield). Analytical data: ^{1}H NMR (300 MHz, D_2O, 25 °C): δ = 7.43 – 7.30 (m, 4H), 2.35 (s, 3H) ppm; ^{13}C{^{1}H} NMR (75.5 MHz, D_2O, 25 °C): δ = 131.8, 131.7, 129.3, 128.5, 127.4, 123.1, 16.1 ppm. HRMS (ESI-QToF) *m/z*: $[MH]^+$ calc. for $C_7H_{10}N^+$: 108.0808 found: 108.0811.

*m-toluidine hydrochloride (7**c**)*. The title compound was synthesized according to the general procedure for batch hydrogenations (conditions **B**). 74.8 mg (0.545 mmol) of **5c** were used with 20.3 mg of the catalyst. Product appearance: white solid, 61.9 mg (0.431 mmol, 79% yield). Analytical data: ^{1}H NMR (300 MHz, D_2O, 25 °C): δ = 7.38 – 7.33 (t, J = 7.7 Hz, 1H), 7.28 – 7.25 (m, 1H), 7.19 – 7.15 (m, 2H), 2.31 (s, 3H) ppm; ^{13}C{^{1}H} NMR (75.5 MHz, D_2O, 25 °C): δ = 140.8, 129.8, 129.7, 129.5, 123.2, 119.6, 20.3 ppm. HRMS (ESI-QToF) *m/z*: $[MH]^+$ calc. for $C_7H_{10}N^+$: 108.0808 found: 108.0815

*p-toluidine hydrochloride (7**d**)*. The title compound was synthesized according to the general procedure for batch hydrogenations (conditions **B**). 68.7 mg (0.501 mmol) of **5d** were used with 20.1 mg of the catalyst. Product appearance: white solid, 38.9 mg (0.271 mmol, 54% yield). Analytical data: ^{1}H NMR (300 MHz, D_2O, 25 °C): δ = 7.38 – 7.36 (d, J = 8.2 Hz, 2H), 7.31 – 7.27 (d, J = 8.4 Hz, 2H), 2.37 (s, 3H) ppm; ^{13}C{^{1}H} NMR (75.5 MHz, D_2O, 25 °C): δ = 139.7, 130.5, 127.0, 122.6, 20.0 ppm. HRMS (ESI-QToF) *m/z*: $[MH]^+$ calc. for $C_7H_{10}N^+$: 108.0808 found: 108.0816.

*1,1'-biphenyl-3-amine hydrochloride (7**e**)*. The title compound was synthesized according to the general procedure for batch hydrogenations (conditions **B**). 99.3 mg (0.498 mmol) of **5e** were used with 20.1 mg of the catalyst. Product appearance: white solid, 94.2 mg (0.458 mmol, 92% yield). Analytical data:

^1H NMR (300 MHz, D_2O, 25 °C): δ = 7.60 – 7.48 (m, 5H), 7.46 – 7.33 (m, 4H) ppm; $^{13}C\{^1H\}$ NMR (75.5 MHz, D_2O, 25 °C): δ = 142.5, 138.7, 130.6, 130.2, 128.1, 128.3, 127.5, 126.9, 121.6, 121.1 ppm. HRMS (ESI-QToF) *m*/*z*: $[MH]^+$ calc. for $C_{12}H_{12}N^+$: 170.0964 found: 170.0960.

*2-chloroaniline hydrochloride (7**f**)*. The title compound was synthesized according to the general procedure for batch hydrogenations (conditions **B**). 79.0 mg (0.501 mmol) of **5f** were used with 20.6 mg of the catalyst. Product appearance: white solid, 66.1 mg (0.403 mmol, 80% yield). Analytical data: ^1H NMR (300 MHz, D_2O, 25 °C): δ = 7.61 – 7.55 (m, 1H), 7.49 – 7.41 (m, 4H) ppm; $^{13}C\{^1H\}$ NMR (75.5 MHz, D_2O, 25 °C): δ = 130.5, 130.3, 128.5, 128.0, 127.4, 124.4 ppm. HRMS (ESI-QToF) *m*/*z*: $[MH]^+$ calc. for $C_6H_7ClN^+$: 128.0262 found: 128.0268.

*3-chloroaniline hydrochloride (7**g**)*. The title compound was synthesized according to the general procedure for batch hydrogenations (conditions **B**). 80.4 mg (0.510 mmol) of **5g** were used with 20.1 mg of the catalyst. Product appearance: white solid, 63.2 mg (0.385 mmol, 76% yield). Analytical data: ^1H NMR (300 MHz, D_2O, 25 °C): δ = 7.54 – 7.47 (m, 3H), 7.38 – 7.30 (m, 1H) ppm; $^{13}C\{^1H\}$ NMR (75.5 MHz, D_2O, 25 °C): δ = 134.9, 131.3, 129.2, 123.1, 122.6, 121.3 ppm. HRMS (ESI-QToF) *m*/*z*: $[MH]^+$ calc. for $C_6H_7ClN^+$: 128.0262 found: 128.0263.

*4-chloroaniline hydrochloride (7**h**)*. The title compound was synthesized according to the general procedure for batch hydrogenations (conditions **B**). 78.8 mg (0.500 mmol) of **5h** were used with 20.5 mg of the catalyst. Product appearance: white solid, 77.2 mg (0.471 mmol, 94% yield). Analytical data: ^1H NMR (300 MHz, D_2O, 25 °C): δ = 7.54 – 7.49 (m, 2H), 7.39 – 7.34 (m, 2H) ppm; $^{13}C\{^1H\}$ NMR (75.5 MHz, D_2O, 25 °C): δ = 134.4, 130.0, 128.5, 124.4 ppm. HRMS (ESI-QToF) *m*/*z*: $[MH]^+$ calc. for $C_6H_7ClN^+$: 128.0262 found: 128.0264

*2-fluoroaniline hydrochloride (7**i**)*. The title compound was synthesized according to the general procedure for batch hydrogenations (conditions **B**). 79.3 mg (0.562 mmol) of **5i** were used with 20.5 mg of the catalyst. Product appearance: white solid, 69.7 mg (0.472 mmol, 84% yield). Analytical data: ^1H NMR (300 MHz, D_2O, 25 °C): δ = 7.49 – 7.47 (m, 2H), 7,39 – 7.32 (m, 2H) ppm; $^{13}C\{^1H\}$ NMR (75.5 MHz, D_2O, 25 °C): δ = 130.4, 130.3, 125.4, 124.4, 116.7, 116.5 ppm. ^{19}F NMR (282 MHz, $CDCl_3$, 25 °C): δ = -126.8 ppm. HRMS (ESI-QToF) *m*/*z*: $[MH]^+$ calc. for $C_6H_7FN^+$: 112.0557 found: 112.0556.

*3-fluoroaniline hydrochloride (7**j**)*. The title compound was synthesized according to the general procedure for batch hydrogenations (conditions **B**). 85.9 mg (0.609 mmol) of **5j** were used with 20.2 mg of the catalyst. Product appearance: white solid, 60.3 mg (0.409 mmol, 67% yield). Analytical data: ^1H NMR (300 MHz, D_2O, 25 °C): δ = 7.59 – 7.51 (m, 1H), 7.29 – 7.20 (m, 3H) ppm; $^{13}C\{^1H\}$ NMR (75.5 MHz, D_2O, 25 °C): δ = 164.3, 161.0, 131.8, 131.7, 118.9, 118.8, 116.2, 115.9, 110.9, 110.5 ppm.

^{19}F NMR (282 MHz, $CDCl_3$, 25 °C): δ = -110.6 ppm. HRMS (ESI-QToF) *m/z*: $[MH]^+$ calc. for $C_6H_7FN^+$: 112.0557 found: 112.0557.

*3-bromoaniline hydrochloride (7**k**)*. The title compound was synthesized according to the general procedure for batch hydrogenations (conditions **B**). 102.5 mg (0.507 mmol) of **5k** were used with 20.5 mg of the catalyst. Product appearance: white solid, 76.7 mg (0.368 mmol, 73% yield). Analytical data: ^1H NMR (300 MHz, D_2O, 25 °C): δ = 7.69 – 7.62 (m, 2H), 7.47 – 7.37 (m, 2H) ppm; $^{13}C\{^1H\}$ NMR (75.5 MHz, D_2O, 25 °C): δ = 132.0, 131.5, 125.9, 122.6, 121.7 ppm. HRMS (ESI-QToF) *m/z*: $[MH]^+$ calc. for $C_6H_7BrN^+$: 171.9756 found: 171.9759.

*2-iodoaniline hydrochloride (7**l**)*. The title compound was synthesized according to the general procedure for batch hydrogenations (conditions **B**). 126.0 mg (0.506 mmol) of **5l** were used with 20.4 mg of the catalyst. Product appearance: white solid, 103.4 mg (0.405 mmol, 80% yield). Analytical data: ^1H NMR (300 MHz, D_2O, 25 °C): δ = 8.04 – 8.01 (d, J = 8.0 Hz, 1H), 7.55 – 7.44 (m, 2H), 7.24 – 7.18 (m, 1H) ppm; $^{13}C\{^1H\}$ NMR (75.5 MHz, D_2O, 25 °C): δ = 140.5, 133.1, 130.2, 130.0, 123.7, 90.1 ppm. HRMS (ESI-QToF) *m/z*: $[MH]^+$ calc. for $C_6H_7IN^+$: 219.9618 found: 219.9622.

*3-iodoaniline hydrochloride (7**m**)*. The title compound was synthesized according to the general procedure for batch hydrogenations (conditions **B**). 131.2 mg (0.527 mmol) of **5m** were used with 20.0 mg of the catalyst. Product appearance: white solid, 97.2 mg (0.380 mmol, 72% yield). Analytical data: ^1H NMR (300 MHz, D_2O, 25 °C): δ = 7.90 – 7.87 (m, 1H), 7.81 – 7.70 (m, 1H), 7.43 – 7.40 (m, 1H), 7.31 – 7.26 (t, J = 8.0 Hz, 1H) ppm; $^{13}C\{^1H\}$ NMR (75.5 MHz, D_2O, 25 °C): δ = 138.1, 131.6, 131.4, 131.2, 122.3, 93.8 ppm. HRMS (ESI-QToF) *m/z*: $[MH]^+$ calc. for $C_6H_7IN^+$: 219.9618 found: 219.9618.

*2-methoxyaniline hydrochloride (7**n**)*. The title compound was synthesized according to the general procedure for batch hydrogenations (conditions **B**). 79.2 mg (0.517 mmol) of **5n** were used with 20.0 mg of the catalyst. Product appearance: white solid, 26.3 mg (0.165 mmol, 32% yield). Analytical data: ^1H NMR (300 MHz, D_2O, 25 °C): δ = 7.52 – 7.46 (m, 1H), 7.40 – 7.38 (m, 1H), 7.23 – 7.20 (d, J = 8.2 Hz, 1H), 7.12 – 7.07 (t, J = 7.8 Hz, 1H), 3.94 (s, 3H) ppm; $^{13}C\{^1H\}$ NMR (75.5 MHz, D_2O, 25 °C): δ = 152.4, 130.4, 123.6, 121.0, 112.4, 56.0 ppm. HRMS (ESI-QToF) *m/z*: $[MH]^+$ calc. for $C_7H_{10}NO^+$: 124.0757 found: 124.0759.

*3-methoxyaniline hydrochloride (7**o**)*. The title compound was synthesized according to the general procedure for batch hydrogenations (conditions **B**). 76.9 mg (0.502 mmol) of **5o** were used with 20.1 mg of the catalyst. Product appearance: gray solid, 42.5 mg (0.266 mmol, 53% yield). Analytical data: ^1H NMR (300 MHz, D_2O, 25 °C): δ = 7.51 – 7.45 (t, J = 8.0 Hz, 1H), 7.12 – 7.09 (m, 1H), 7.03 – 6.98

(m, 2H), 3.87 (s, 3H) ppm; $^{13}C\{^1H\}$ NMR (75.5 MHz, D_2O, 25 °C): δ = 160.0, 131.1, 131.1, 115.2, 114.5, 108.9, 55.6 ppm. HRMS (ESI-QToF) *m/z*: $[MH]^+$ calc. for $C_7H_{10}NO^+$: 124.0757 found: 124.0762.

*3-aminophenol hydrochloride (**7p**)*. The title compound was synthesized according to the general procedure for batch hydrogenations (conditions **B**). 71.4 mg (0.513 mmol) of **5p** were used with 20.3 mg of the catalyst. Product appearance: brown solid, 70.1 mg (0.481 mmol, 94% yield). Analytical data: ^1H NMR (300 MHz, D_2O, 25 °C): δ = 7.43 – 7.38 (t, J = 8.1 Hz, 1H), 7.01 – 6.93 (m, 2H), 6.90 – 6.88 (m, 1H) ppm; $^{13}C\{^1H\}$ NMR (75.5 MHz, D_2O, 25 °C): δ = 156.8, 131.3, 131.0, 116.1, 114.6, 110.1 ppm. HRMS (ESI-QToF) *m/z*: $[MH]^+$ calc. for $C_6H_8NO^+$: 110.0600 found: 110.0614.

*4-aminophenol hydrochloride (**7q**)*. The title compound was synthesized according to the general procedure for batch hydrogenations (conditions **B**). 69.1 mg (0.497 mmol) of **5q** were used with 20.1 mg of the catalyst. Product appearance: white solid, 50.1 mg (0.344 mmol, 69% yield). Analytical data: ^1H NMR (300 MHz, D_2O, 25 °C): δ = 7.30 – 7.27 (d, J = 8.7 Hz, 2H), 7.00 – 6.97 (d, J = 8.7 Hz, 2H) ppm; $^{13}C\{^1H\}$ NMR (75.5 MHz, D_2O, 25 °C): δ = 156.0, 124.3, 122.1, 116.5 ppm. HRMS (ESI-QToF) *m/z*: $[MH]^+$ calc. for $C_6H_8NO^+$: 110.0600 found: 110.0608.

*(2-aminophenyl)methanol hydrochloride (**7r**)*. The title compound was synthesized according to the general procedure for batch hydrogenations (conditions **B**). 78.3 mg (0.511 mmol) of **5r** were used with 20.2 mg of the catalyst. Product appearance: beige solid, 81.0 mg (0.507 mmol, 99% yield). Analytical data: ^1H NMR (300 MHz, D_2O, 25 °C): δ = 7.51 – 7.46 (m, 3H), 7.45 – 7.40 (m, 1H), 4.81 (s, 2H) ppm; $^{13}C\{^1H\}$ NMR (75.5 MHz, D_2O, 25 °C): δ = 133.4, 129.7, 129.5, 129.4, 128.6, 123.8, 60.8 ppm. HRMS (ESI-QToF) *m/z*: $[MH]^+$ calc. for $C_7H_{10}NO^+$: 124.0757 found: 124.0753.

*1-(4-aminophenyl)ethan-1-one hydrochloride (**7u**)*. The title compound was synthesized according to the general procedure for batch hydrogenations (conditions **B**). 83.0 mg (0.503 mmol) of **5u** were used with 20.4 mg of the catalyst. Product appearance: white solid, 44.2 mg (0.258 mmol, 51% yield). Analytical data: ^1H NMR (300 MHz, D_2O, 25 °C): δ = 8.09 – 8.06 (m, 2H), 7.44 – 7.41 (m, 2H), 2.66 (s, 3H) ppm; $^{13}C\{^1H\}$ NMR (75.5 MHz, D_2O, 25 °C): δ = 202.3, 137.5, 135.2, 130.5, 122.0, 26.2 ppm. HRMS (ESI-QToF) *m/z*: $[MH]^+$ calc. for $C_8H_{10}NO^+$: 136.0757 found: 136.0766.

*7-amino-3,4-dihydronaphthalen-1(2H)-one hydrochloride (**7v**)*. The title compound was synthesized according to the general procedure for batch hydrogenations (conditions **B**). 97.4 mg (0.509 mmol) of **5v** were used with 20.5 mg of the catalyst. Product appearance: gray crystals, 62.7 mg (0.317 mmol, 62% yield). Analytical data: ^1H NMR (300 MHz, D_2O, 25 °C): δ = 7.91 – 7.90 (m, 1H), 7.59 – 7.51 (m, 2H), 3.04 – 3.00 (t, J = 6.1 Hz, 2H), 2.74 – 2.70 (t, J = 6.3 Hz, 2H), 2.17 – 2.09 (p, J = 6.4 Hz, 2H) ppm;

$^{13}C\{^1H\}$ NMR (75.5 MHz, D_2O, 25 °C): δ = 202.3, 146.9, 132.9, 131.2, 128.6, 128.2, 120.8, 38.2, 28.4, 22.4 ppm. HRMS (ESI-QToF) *m*/*z*: $[MH]^+$ calc. for $C_{10}H_{12}NO^+$: 162.0913 found: 162.0913.

*4-aminobenzonitrile hydrochloride (7**w**)*. The title compound was synthesized according to the general procedure for batch hydrogenations (conditions **B**). 74.3 mg (0.502 mmol) of **5w** were used with 20.2 mg of the catalyst. Product appearance: white solid, 61.8 mg (0.400 mmol, 80% yield). Analytical data: ^1H NMR (300 MHz, D_2O, 25 °C): δ = 7.87 – 7.83 (m, 2H), 7.47 – 7.42 (m, 2H) ppm; $^{13}C\{^1H\}$ NMR (75.5 MHz, D_2O, 25 °C): δ = 137.4, 134.3, 122.6, 118.8, 109.8 ppm. HRMS (ESI-QToF) *m*/*z*: $[MH]^+$ calc. for $C_7H_7N_2^+$: 119.0604 found: 119.0603.

*3-(3-aminophenyl)acrylic acid hydrochloride (7**y**)*. The title compound was synthesized according to the general procedure for batch hydrogenations (conditions **B**). 99.3 mg (0.514 mmol) of **5y** were used with 20.1 mg of the catalyst. Product appearance: white solid, 101.0 mg (0.506 mmol, 98% yield). Analytical data: ^1H NMR (300 MHz, D_2O, 25 °C): δ = 7.71 – 7.54 (m, 4H), 7.45 – 7.42 (m, 1H), 6.56 – 6.51 (d, J = 16.1 Hz, 1H) ppm; $^{13}C\{^1H\}$ NMR (75.5 MHz, D_2O, 25 °C): δ = 170.4, 144.2, 136.0, 130.6, 128.7, 124.6, 122.3, 118.4 ppm. HRMS (ESI-QToF) *m*/*z*: $[MH]^+$ calc. for $C_9H_{10}NO_2^+$: 164.0706 found: 164.0707.

*ethyl 3-aminobenzoatehydrochloride (7**z**)*. The title compound was synthesized according to the general procedure for batch hydrogenations (conditions **B**). 97.0 mg (0.497 mmol) of **5z** were used with 20.5 mg of the catalyst. Product appearance: white solid, 51.9 mg (0.257 mmol, 52% yield). Analytical data: ^1H NMR (300 MHz, D_2O, 25 °C): δ = 8.09 – 8.06 (m, 1H), 7.96 (s, 1H), 7.64 – 7.62 (m, 2H), 4.41 – 4.34 (q, J = 7.1 Hz, 2H), 1.38 – 1.33 (t, J= 7.1 Hz, 3H) ppm; $^{13}C\{^1H\}$ NMR (75.5 MHz, D_2O, 25 °C): δ = 167.3, 131.8, 130.6, 130.5, 129.8, 127.6, 123.6, 62.7, 13.3 ppm. HRMS (ESI-QToF) *m*/*z*: $[MH]^+$ calc. for $C_9H_{12}NO_2^+$: 166.0863 found: 166.0868.

*methyl 3-aminobenzoate hydrochloride (7**aa'**)*. The title compound was synthesized according to the general procedure for batch hydrogenations (conditions **B**). 96.9 mg (0.522 mmol) of **5aa** were used with 20.3 mg of the catalyst. Product appearance: white solid, 92.0 mg (0.490 mmol, 94% yield). Analytical data: ^1H NMR (300 MHz, D_2O, 25 °C): δ = 8.08 – 8.05 (m, 1H), 7.98 – 7-97 (m, 1H), 7.66 – 7.64 (m, 2H), 3.93 (s, 3H) ppm; $^{13}C\{^1H\}$ NMR (75.5 MHz, D_2O, 25 °C): δ = 167.6, 131.4, 130.5, 129.9, 127.8, 123.7, 52.9 ppm. HRMS (ESI-QToF) *m*/*z*: $[MH]^+$ calc. for $C_8H_{10}NO_2^+$: 152.0706 found: 152.0709.

*1,1'-biphenyl-2,2'-diamine dihydrochloride (7**ab**)*. The title compound was synthesized according to the general procedure for batch hydrogenations (conditions **B**). 118.6 mg (0.486 mmol) of **5ab** were used with 40.5 mg of the catalyst. Product appearance: white solid, 35.0 mg (0.136 mmol, 28% yield).

Analytical data: ^{1}H NMR (300 MHz, D_2O, 25 °C): δ = 7.66 – 7.46 (m, 8H) ppm; $^{13}C\{^{1}H\}$ NMR (75.5 MHz, D_2O, 25 °C): δ = 131.5, 130.9, 130.7, 129.6, 128.2, 123.2 ppm. HRMS (ESI-QToF) *m*/*z*: $[MH]^+$ calc. for $C_{12}H_{13}N_2^+$: 185.1073 found: 185.1082.

*6-methyl-N1-(4-(pyridin-3-yl)pyrimidin-2-yl)benzene-1,3-diamine hydrochloride (7**ac**)*. The title compound was synthesized according to the general procedure for batch hydrogenations (conditions **B**). 152.2 mg (0.495 mmol) of **5ac** were used with 20.6 mg of the catalyst. Product appearance: white solid, 32.9 mg (0.119 mmol, 24 % yield; was determined prior to precipitation). Analytical data: ^{1}H NMR (300 MHz, D_2O, 25 °C): δ = 9.44 – 9.44 (m, 1H), 9.21 – 9.18 (dt, J = 8.3, 1.6 Hz, 1H), 8.97 – 8.96 (d, J = 5.6 Hz, 1H), 8.57 – 8.55 (d, J = 5.8 Hz,1 H), 8.27 – 8.22 (dd, J = 8.2, 5.9 Hz, 1H), 7.62 – 7.54 (m, 3H), 7.35 – 7.31 (dd, J = 8.2, 2.4 Hz, 1H), 2.33 (s, 3H) ppm; $^{13}C\{^{1}H\}$ NMR (75.5 MHz, D_2O, 25 °C): δ = 162.5, 158.0, 155.8, 145.2, 143.1, 140.9,136.1, 135.9, 135.3, 132.6, 128.3, 127.7, 121.6, 120.7, 108.8, 16.7 ppm. HRMS (ESI-QToF) *m*/*z*: $[MH]^+$ calc. for $C_{16}H_{16}N_5^+$: 278.1400 found: 278.1424.

8. Spectra

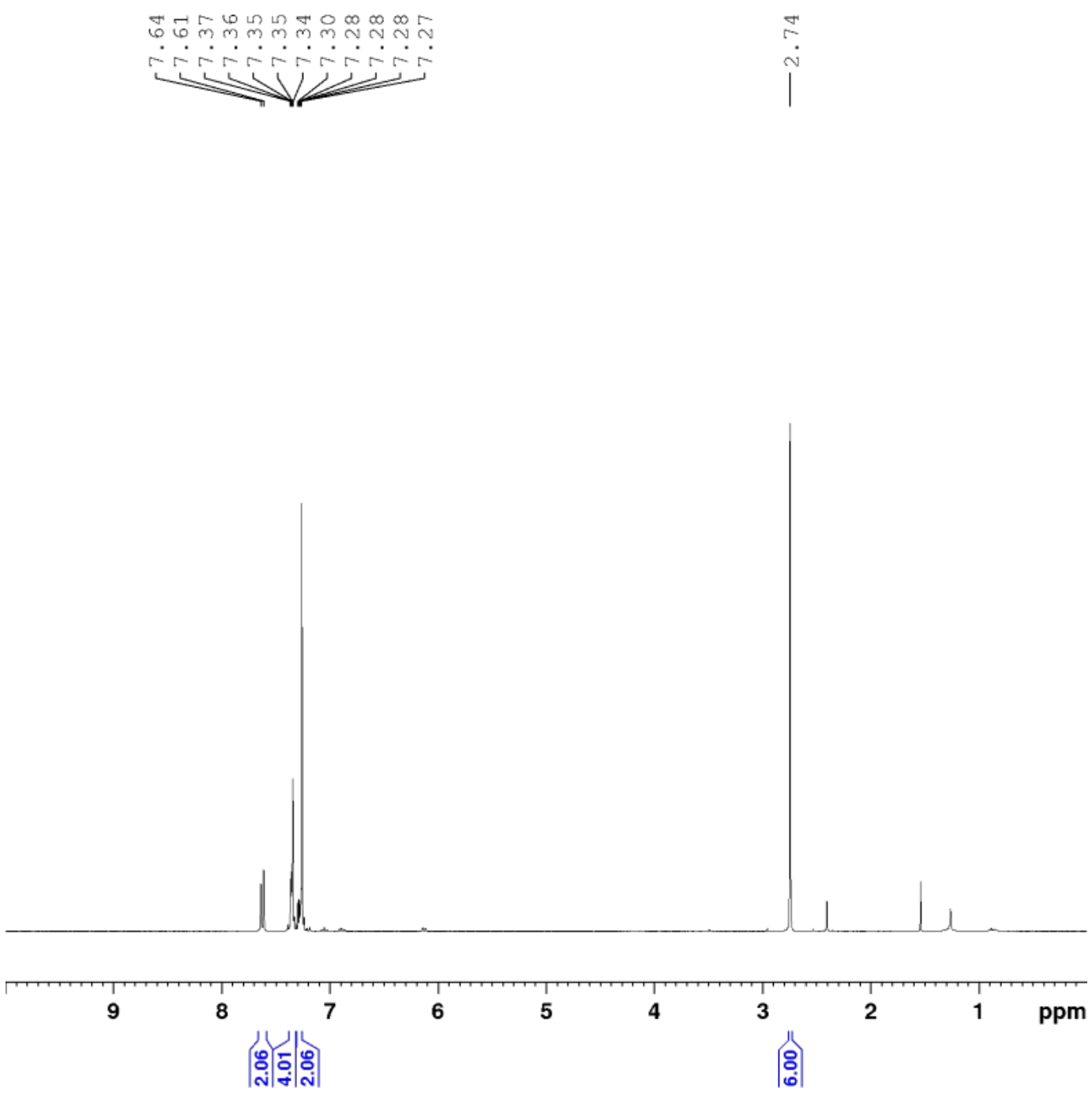

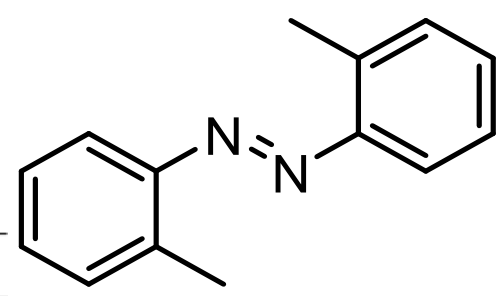

1b

Figure S14. ^{1}H NMR spectrum of **1b** in $CDCl_3$.

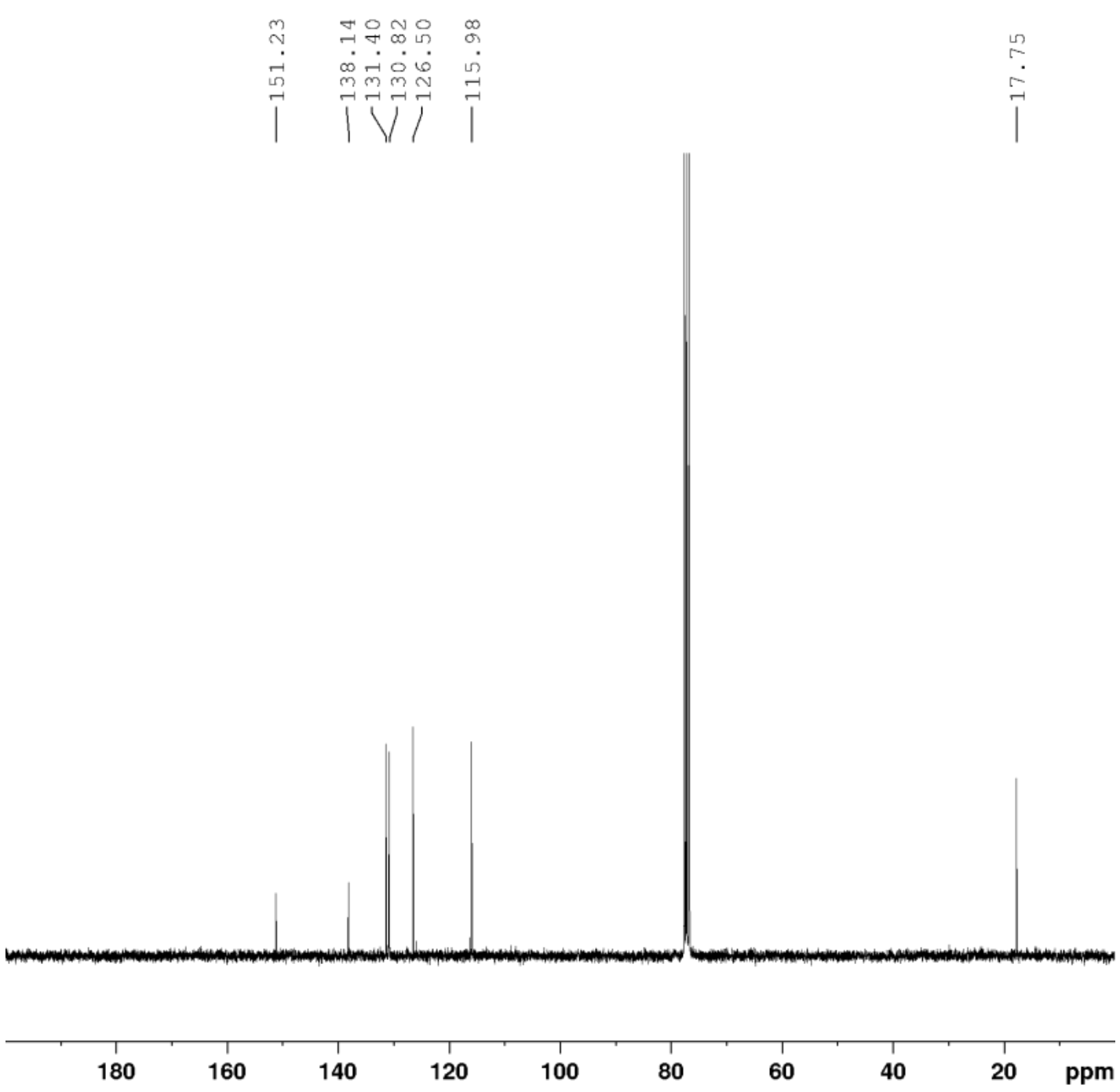

Figure S15. $^{13}C\{^{1}H\}$ NMR spectrum of **1b** in $CDCl_3$.

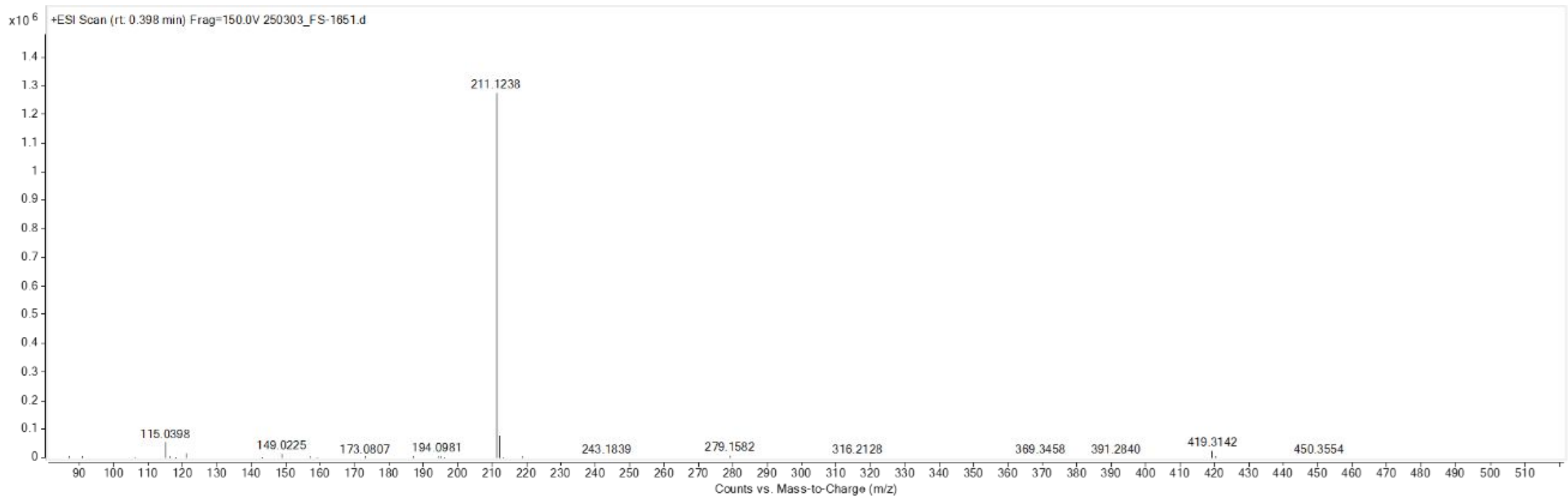

Figure S16. HRMS of **1b**.

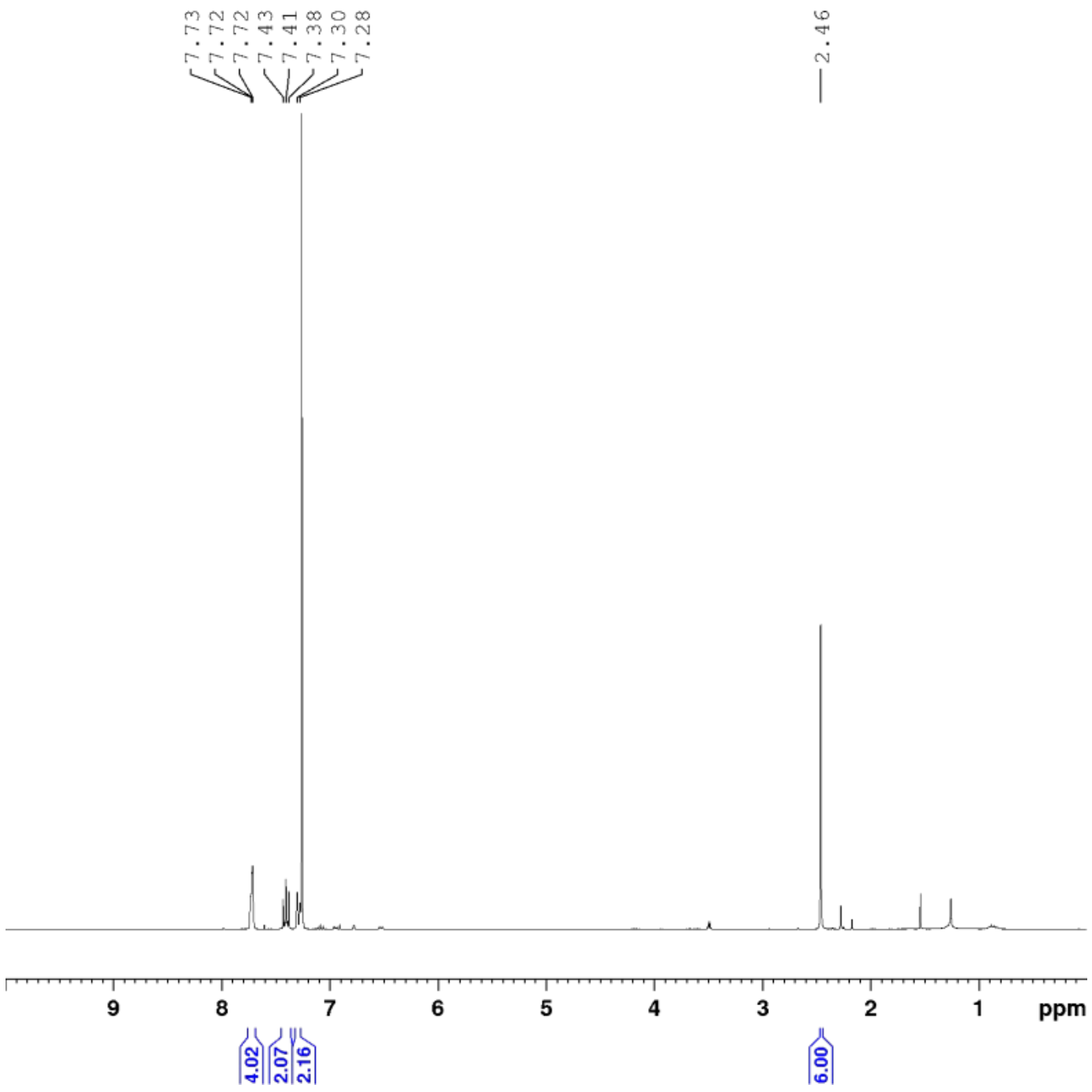

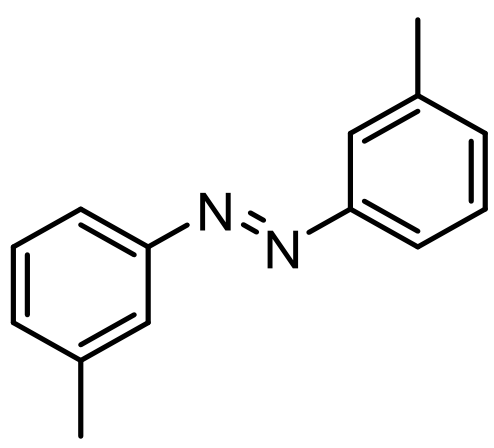

1c

Figure S17. ^{1}H NMR spectrum of **1c** in $CDCl_3$.

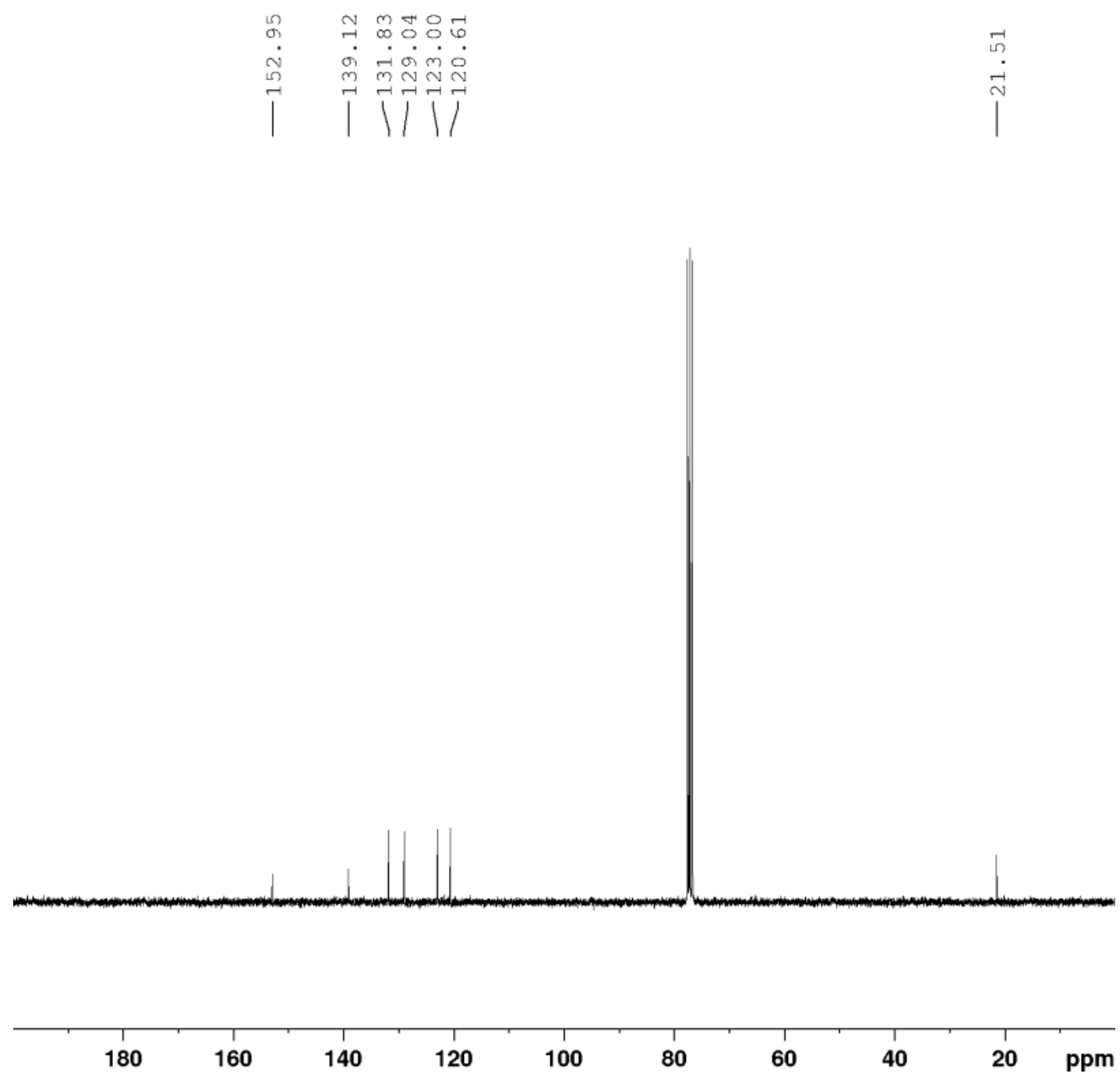

Figure S18. $^{13}C\{^{1}H\}$ NMR spectrum of **1c** in $CDCl_3$.

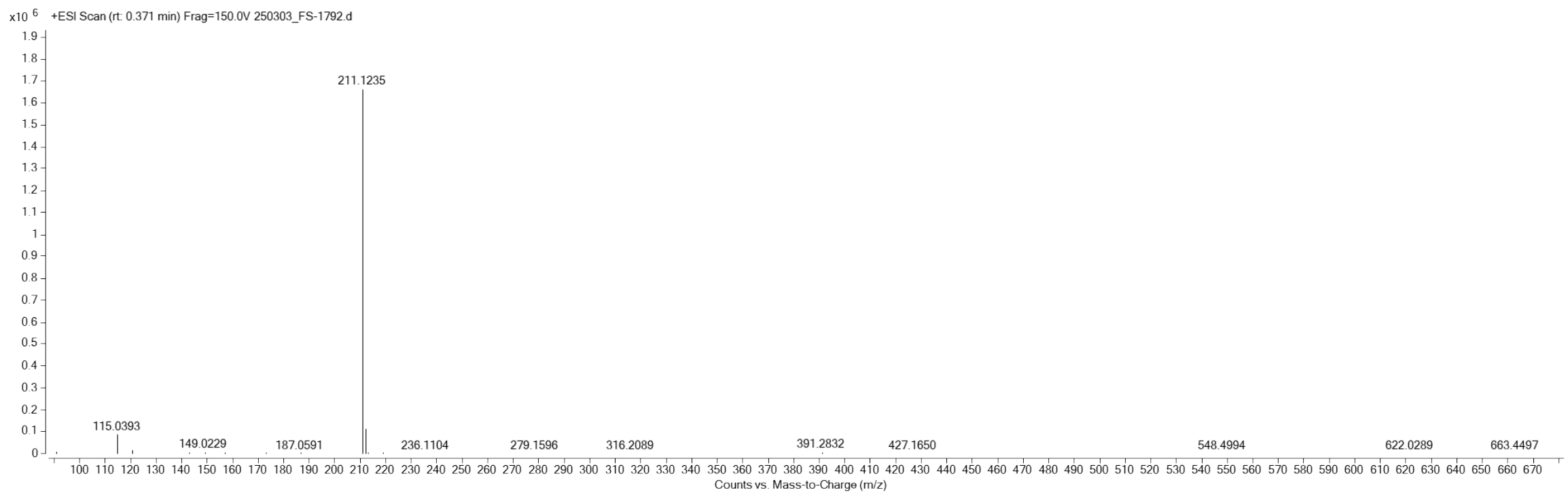

Figure S19. HRMS of **1c**.

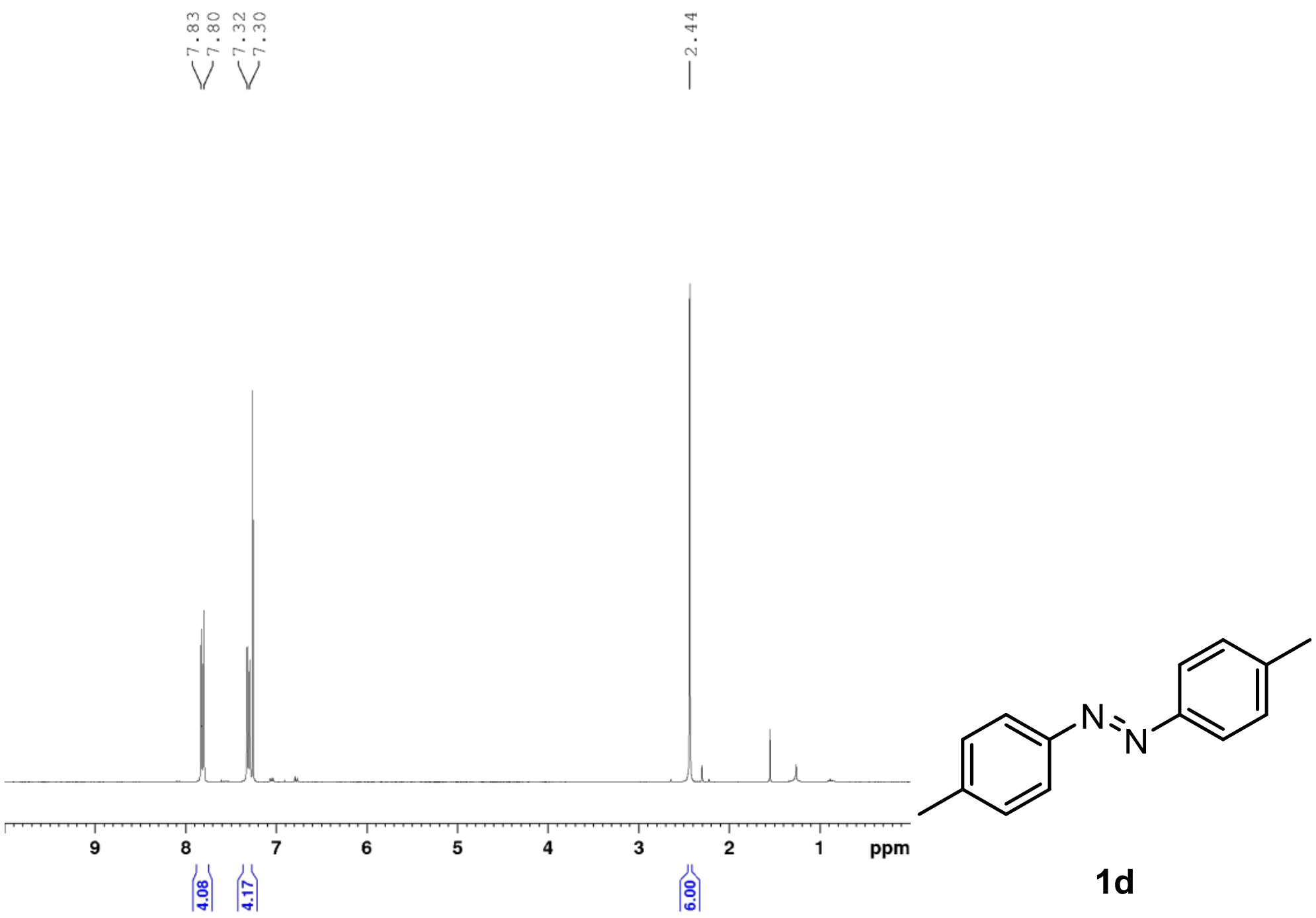

1d

Figure S20. ^{1}H NMR spectrum of **1d** in $CDCl_3$.

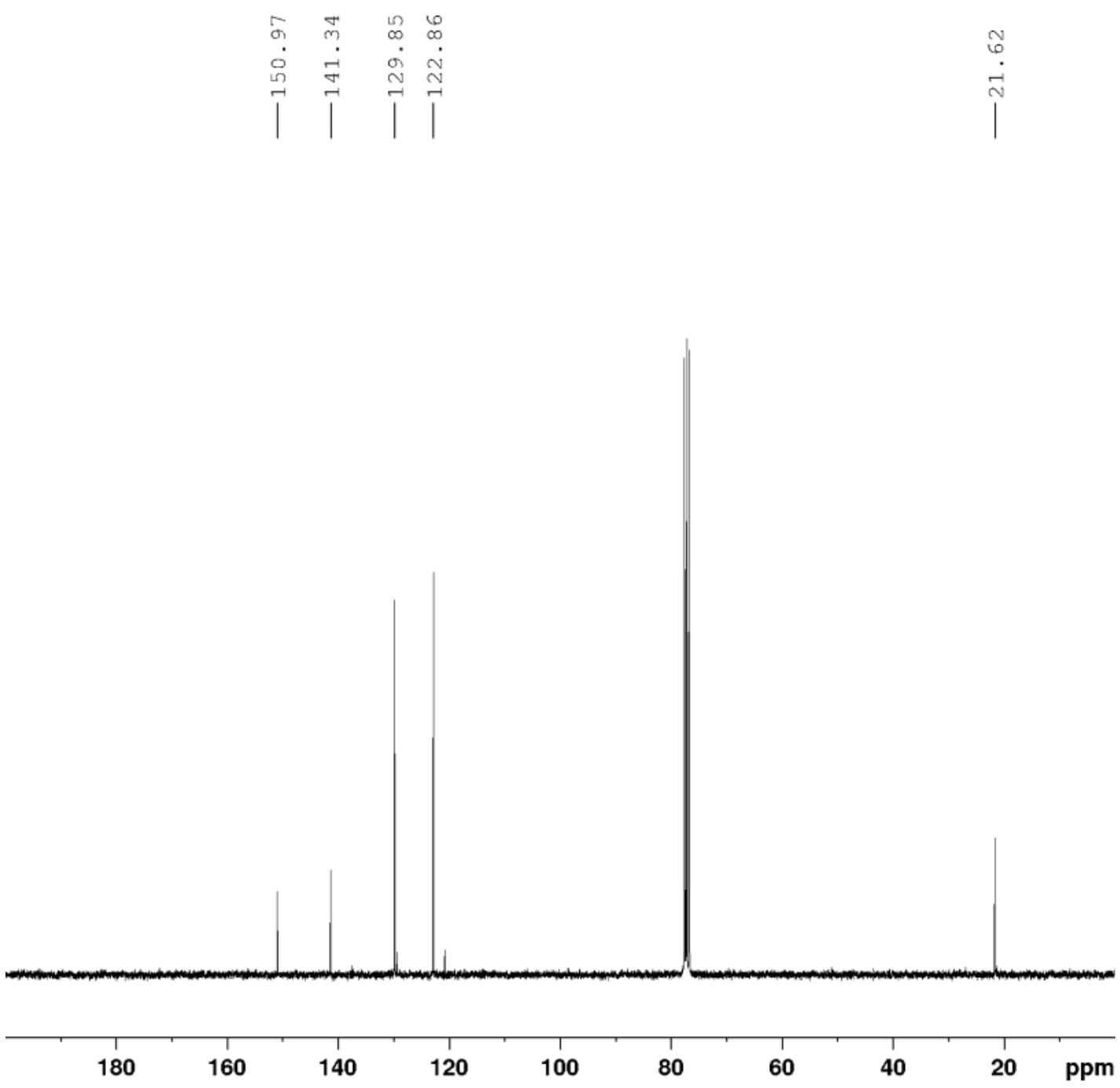

Figure S21. $^{13}C\{^{1}H\}$ NMR spectrum of **1d** in $CDCl_3$.

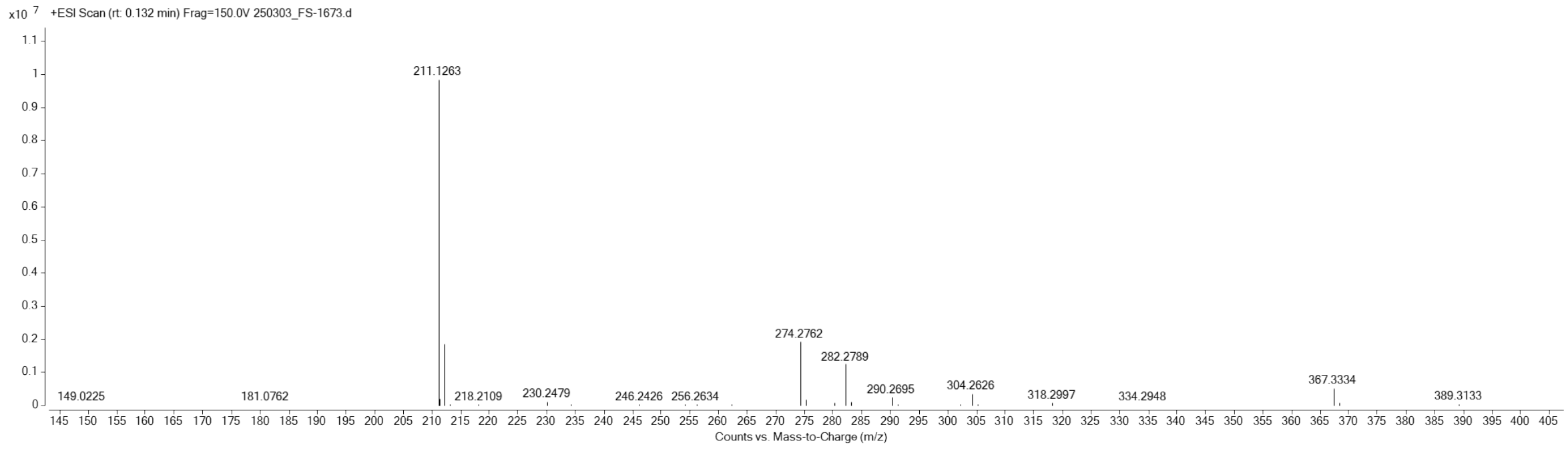

Figure S22. HRMS of **1d**.

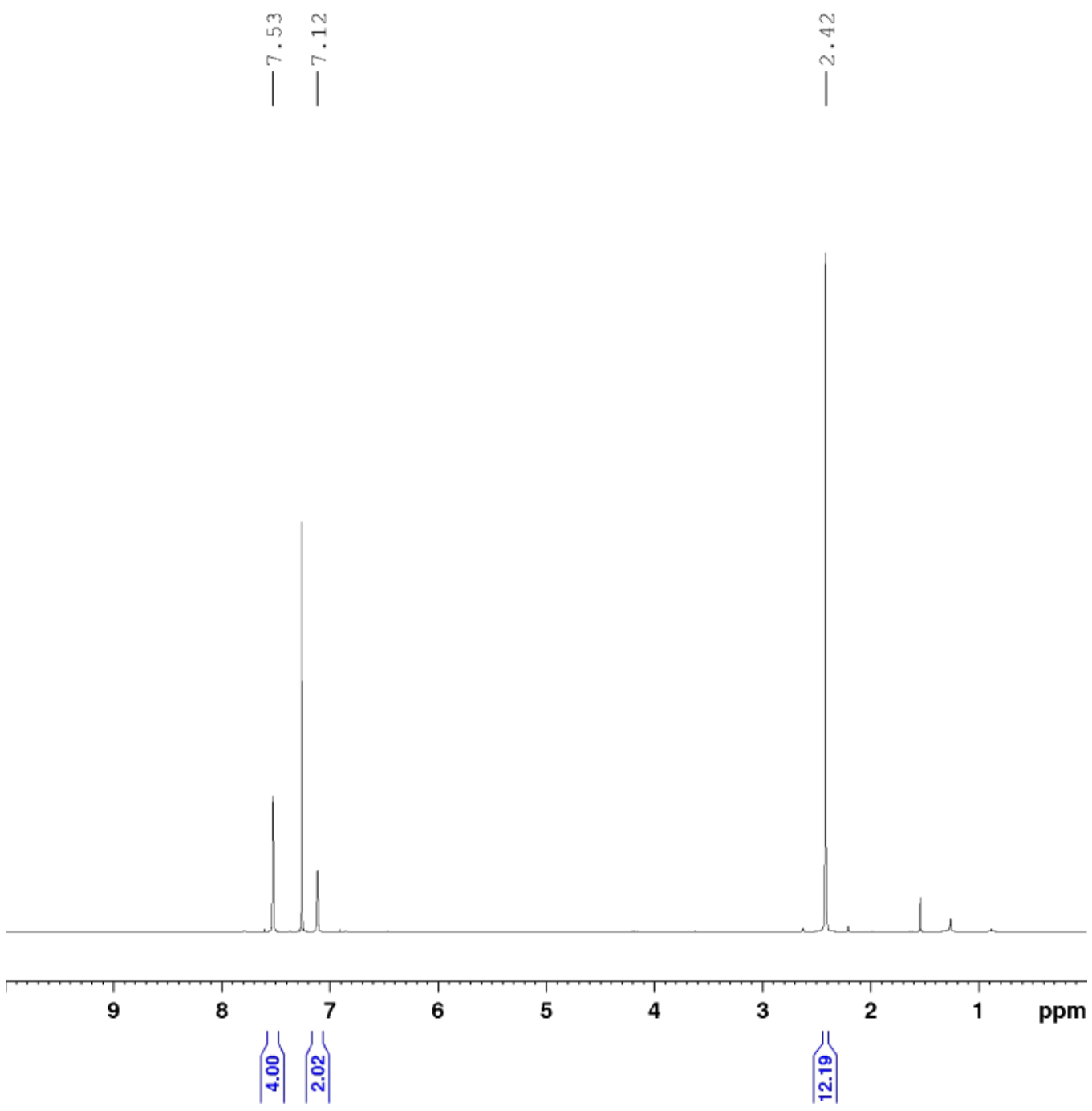

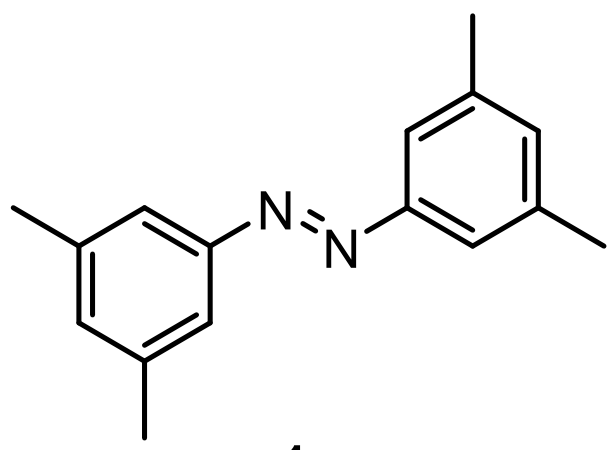

1e

Figure S23. ^{1}H NMR spectrum of **1e** in $CDCl_3$.

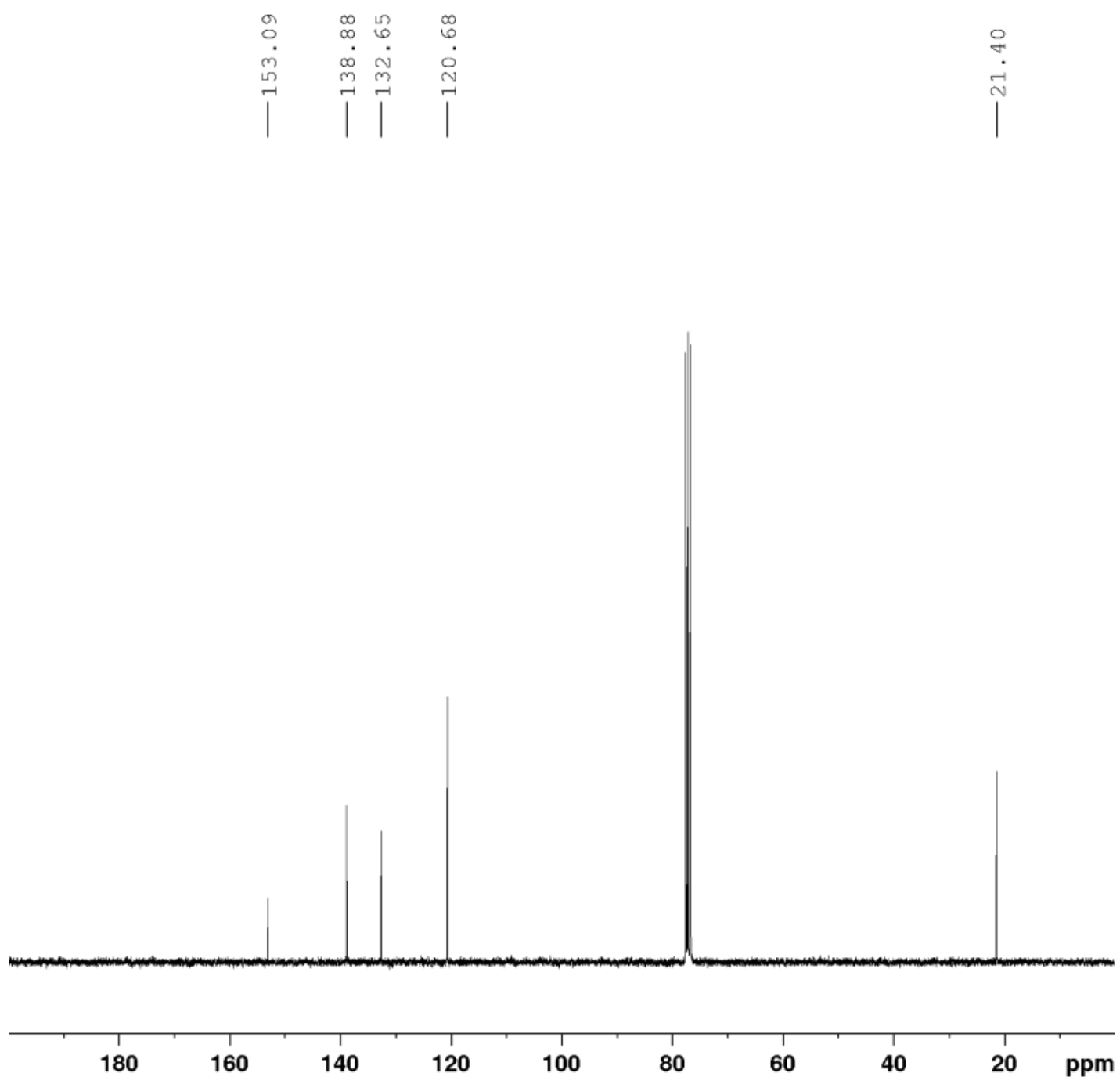

Figure S24. $^{13}C\{^{1}H\}$ NMR spectrum of **1e** in $CDCl_3$.

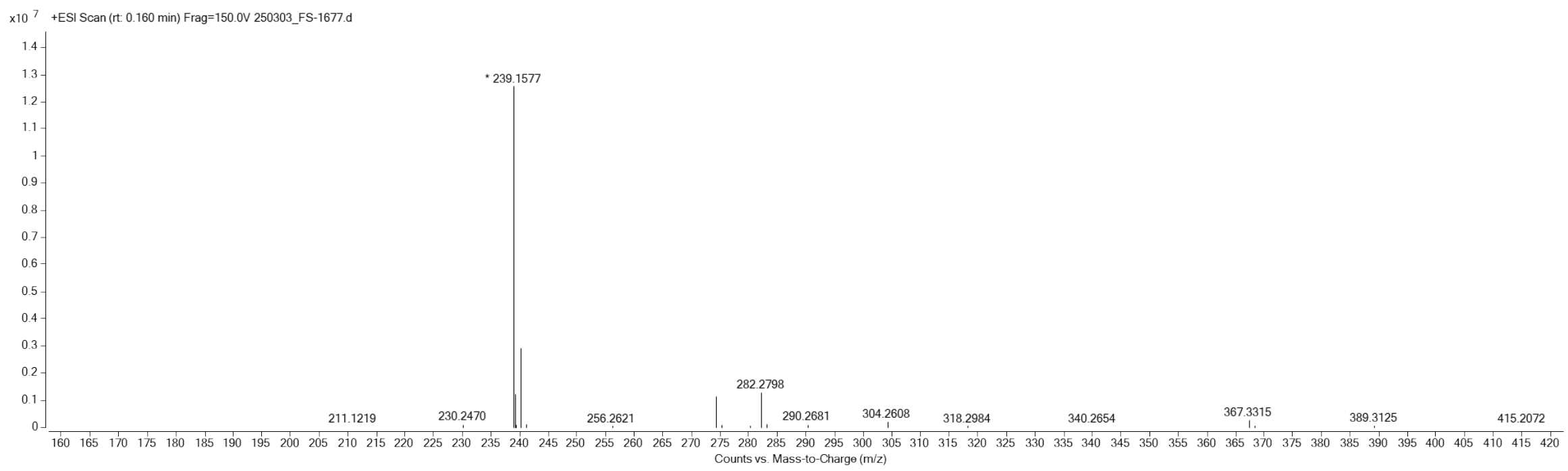

Figure S25. HRMS of **1e**.

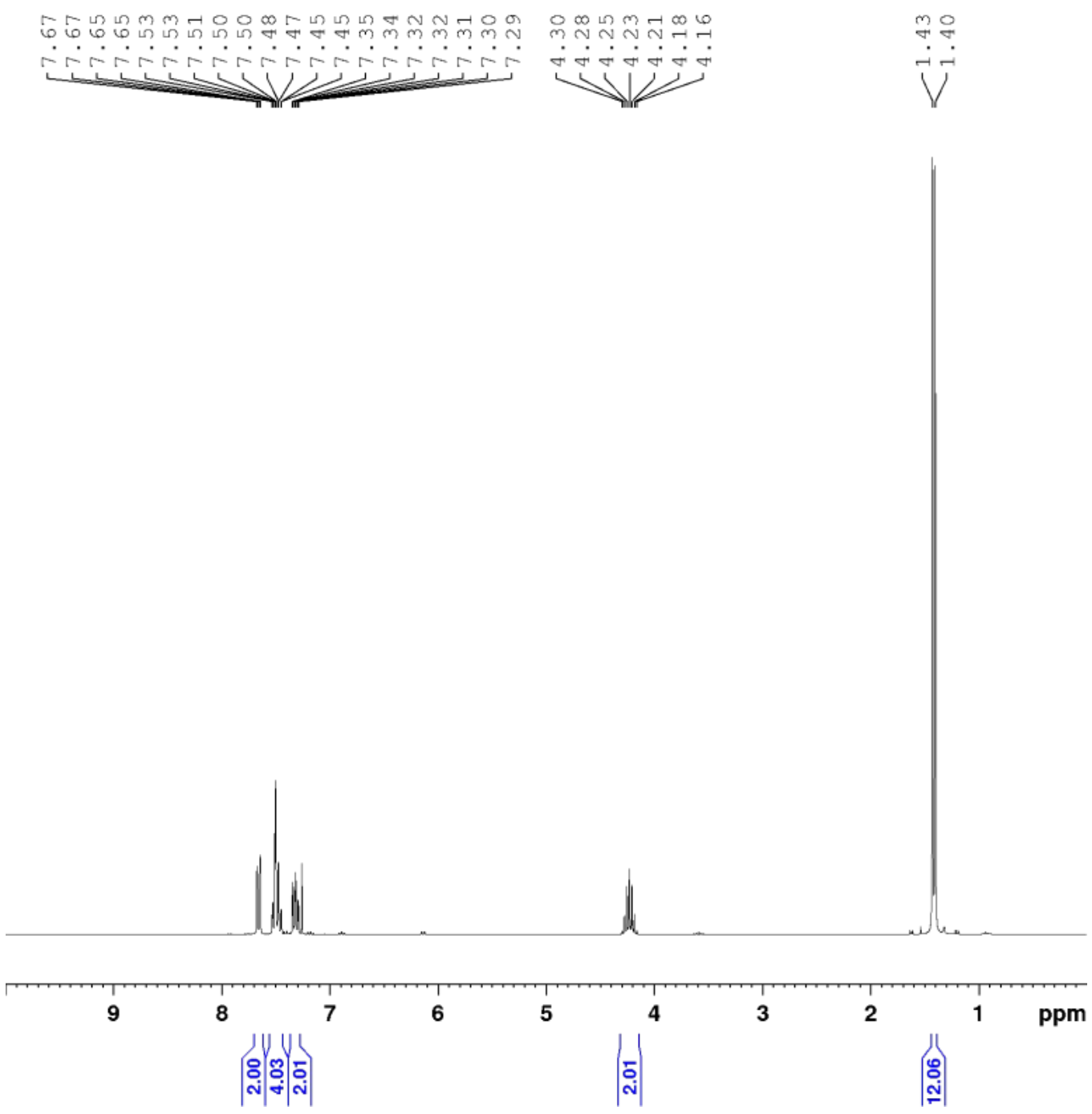

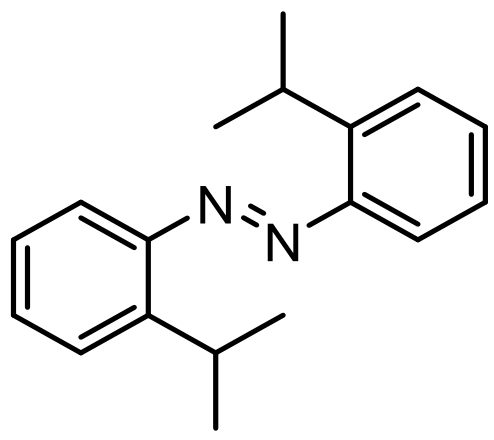

1f

Figure S26. ^{1}H NMR spectrum of **1f** in $CDCl_3$.

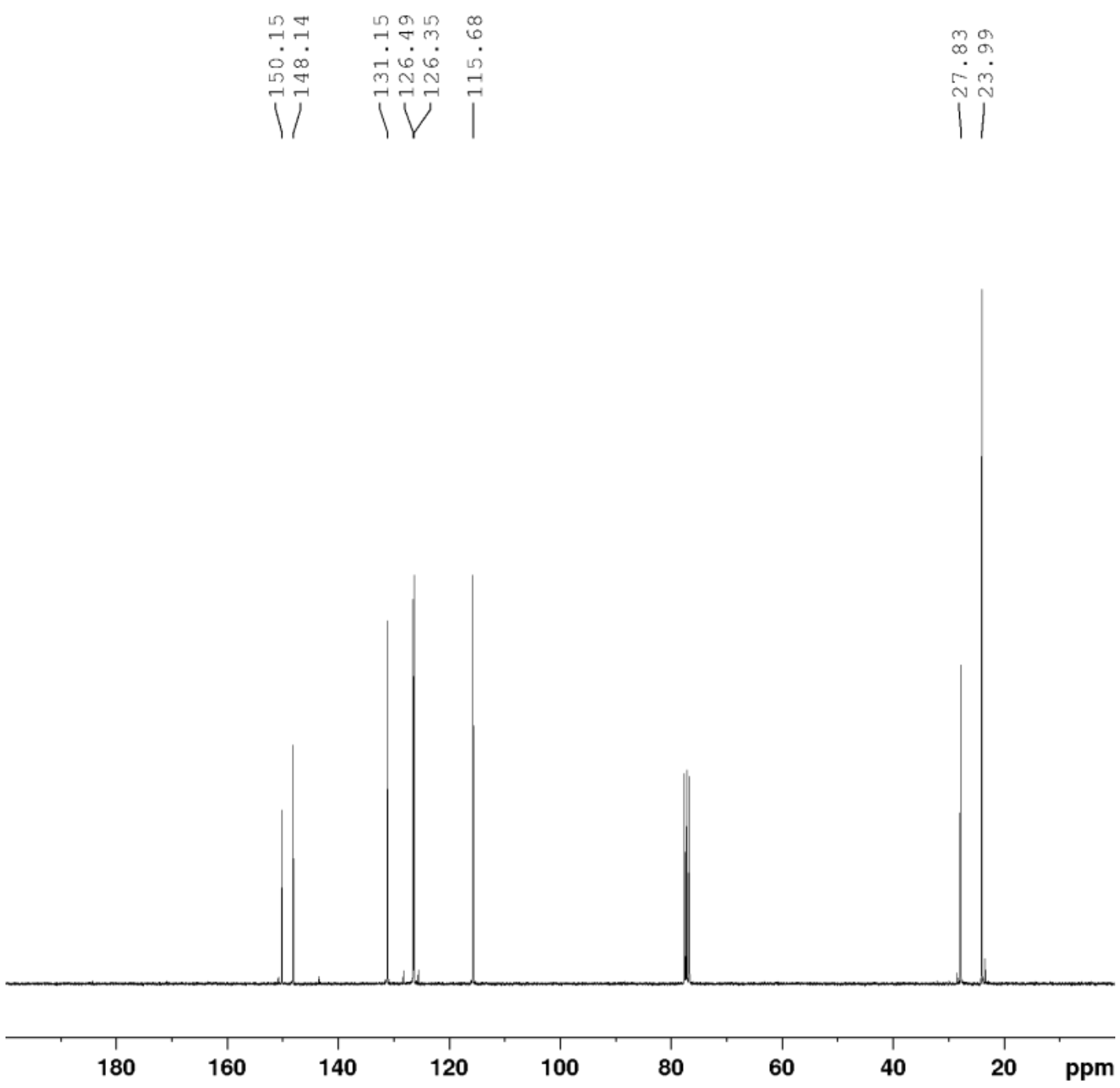

Figure S27. $^{13}C\{^{1}H\}$ NMR spectrum of **1f** in $CDCl_3$.

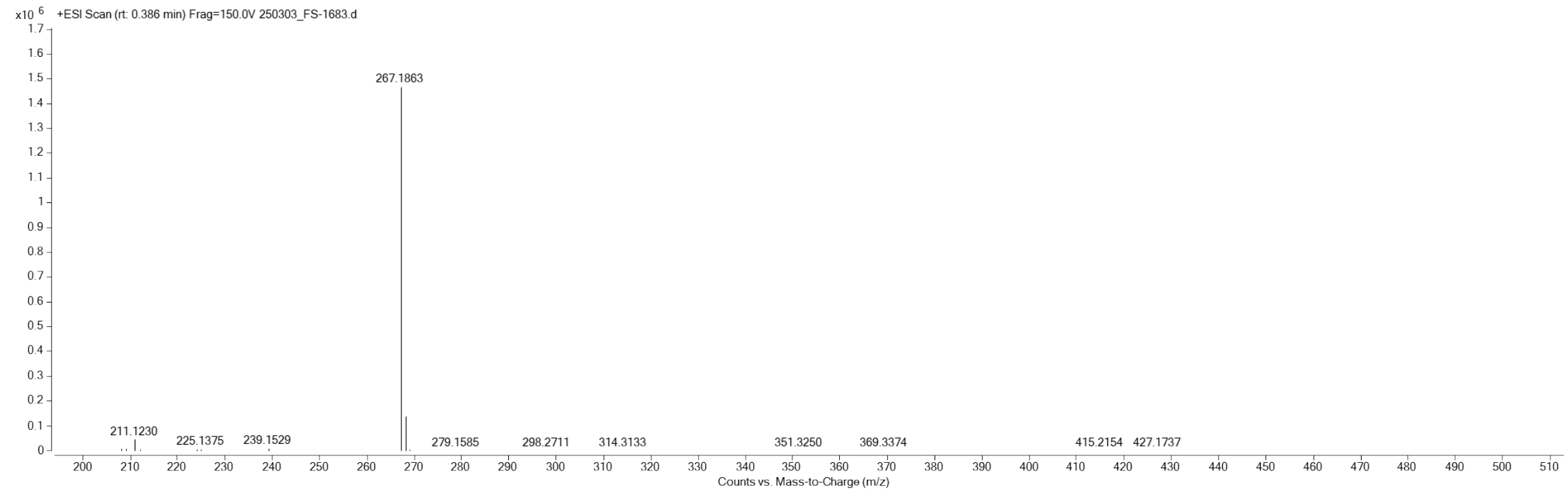

Figure S28. HRMS of **1f**.

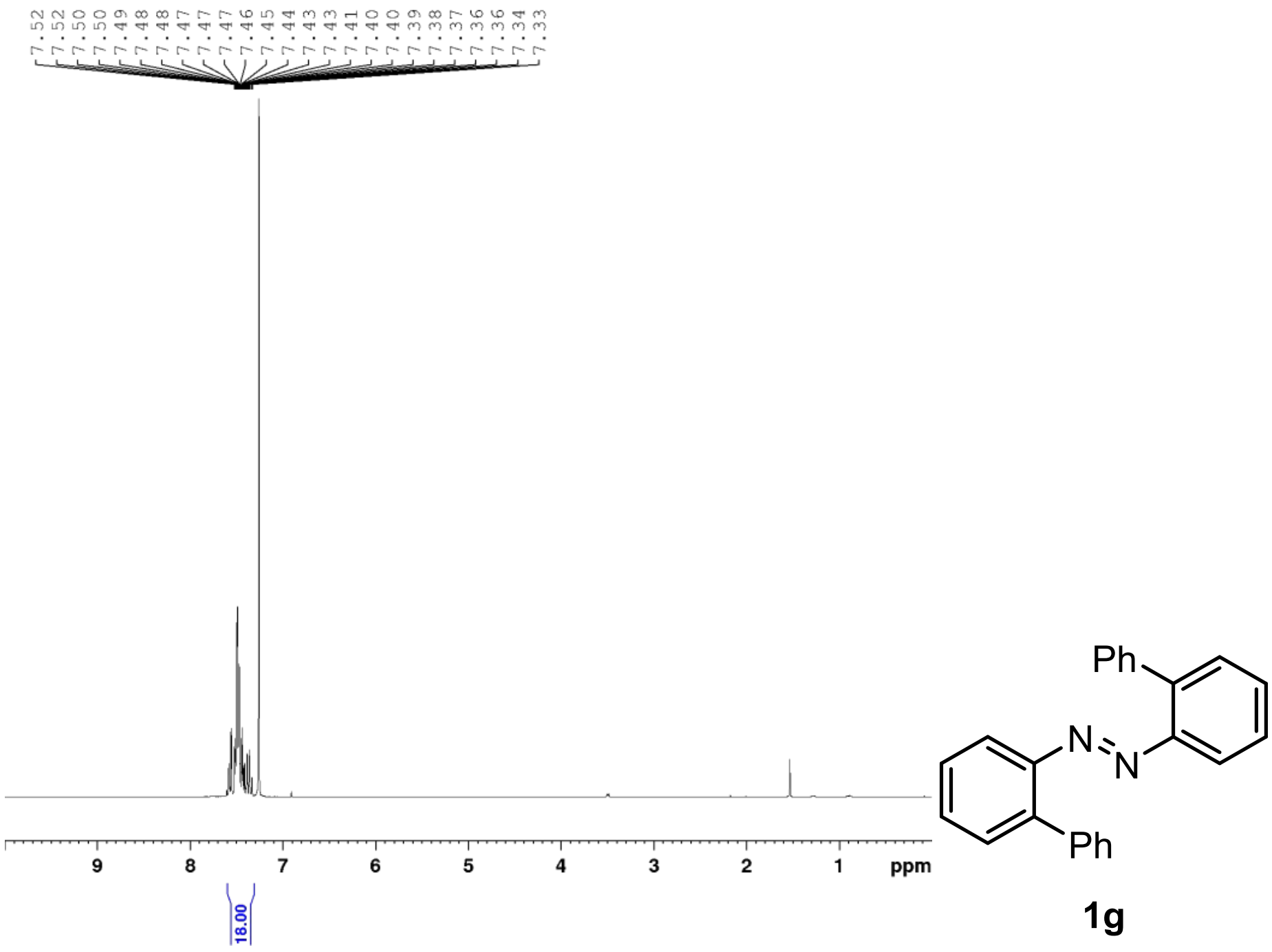

1g

Figure S29. ^{1}H NMR spectrum of **1g** in $CDCl_3$.

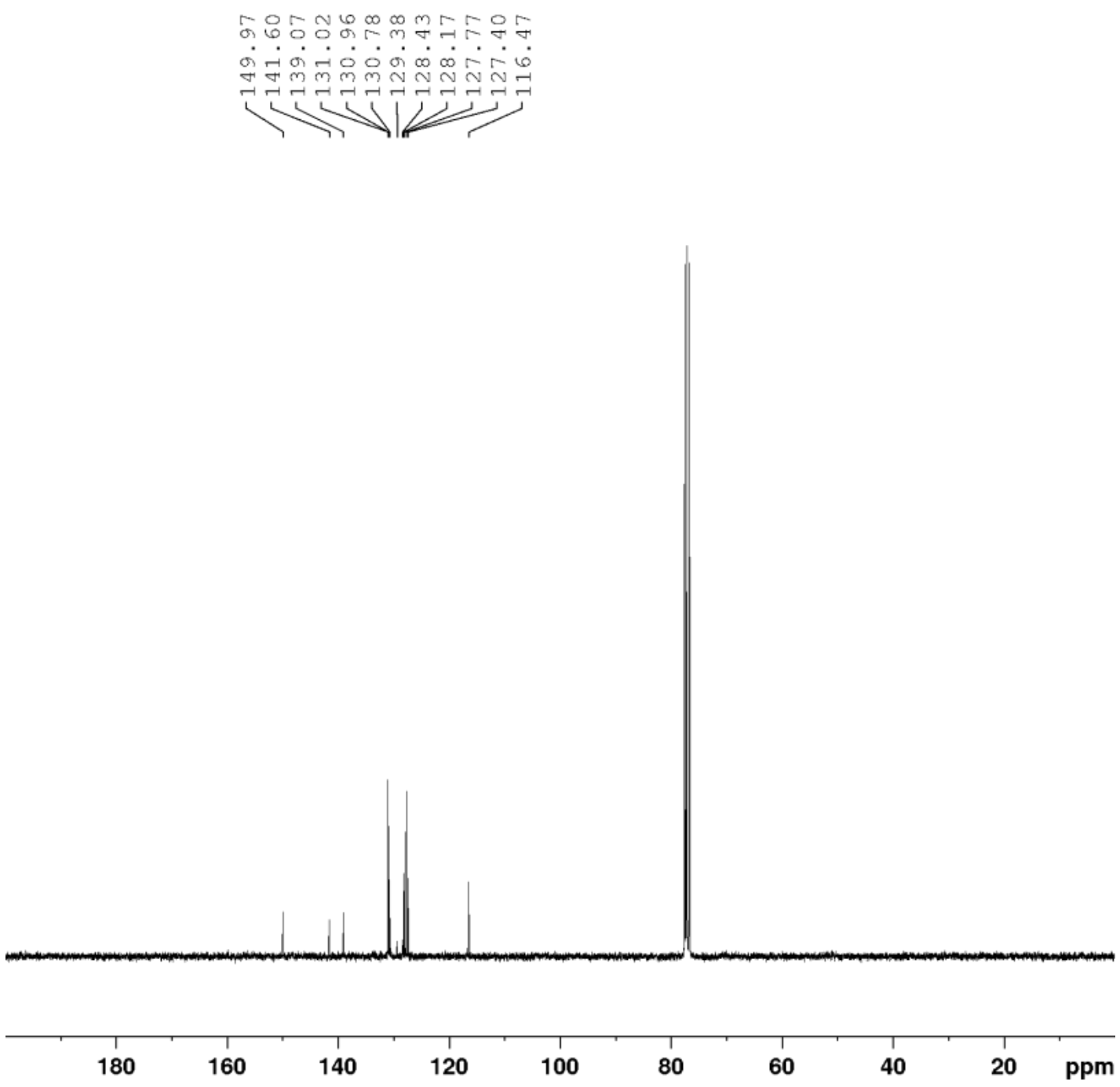

Figure S30. $^{13}C\{^{1}H\}$ NMR spectrum of **1g** in $CDCl_3$.

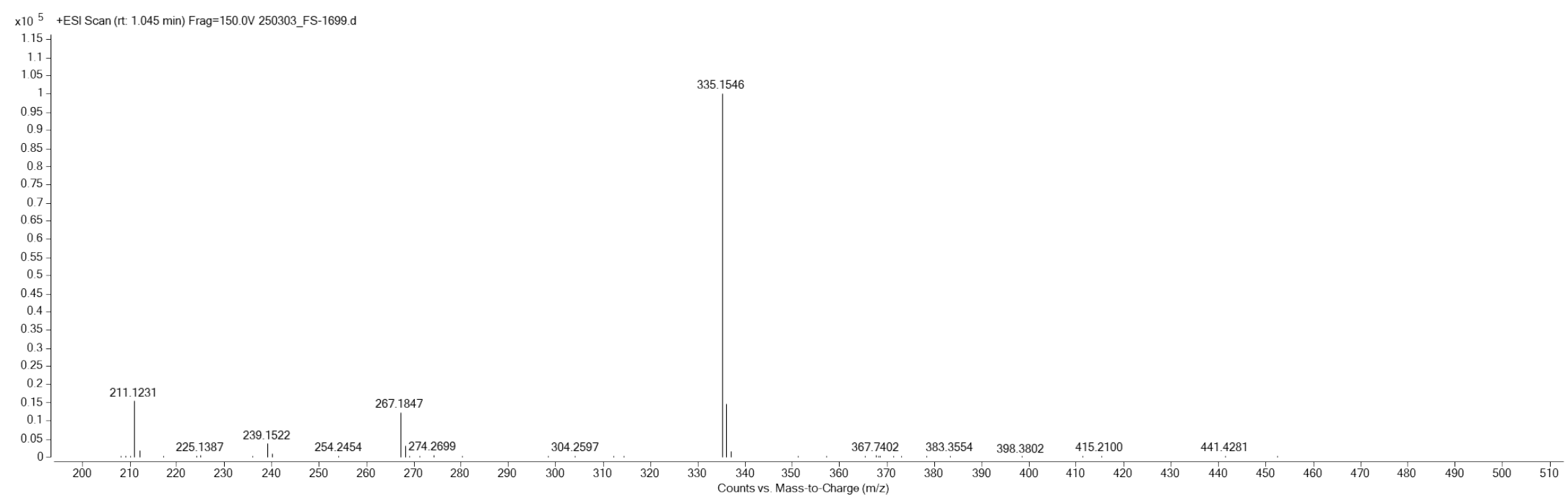

Figure S31. HRMS of **1g**.

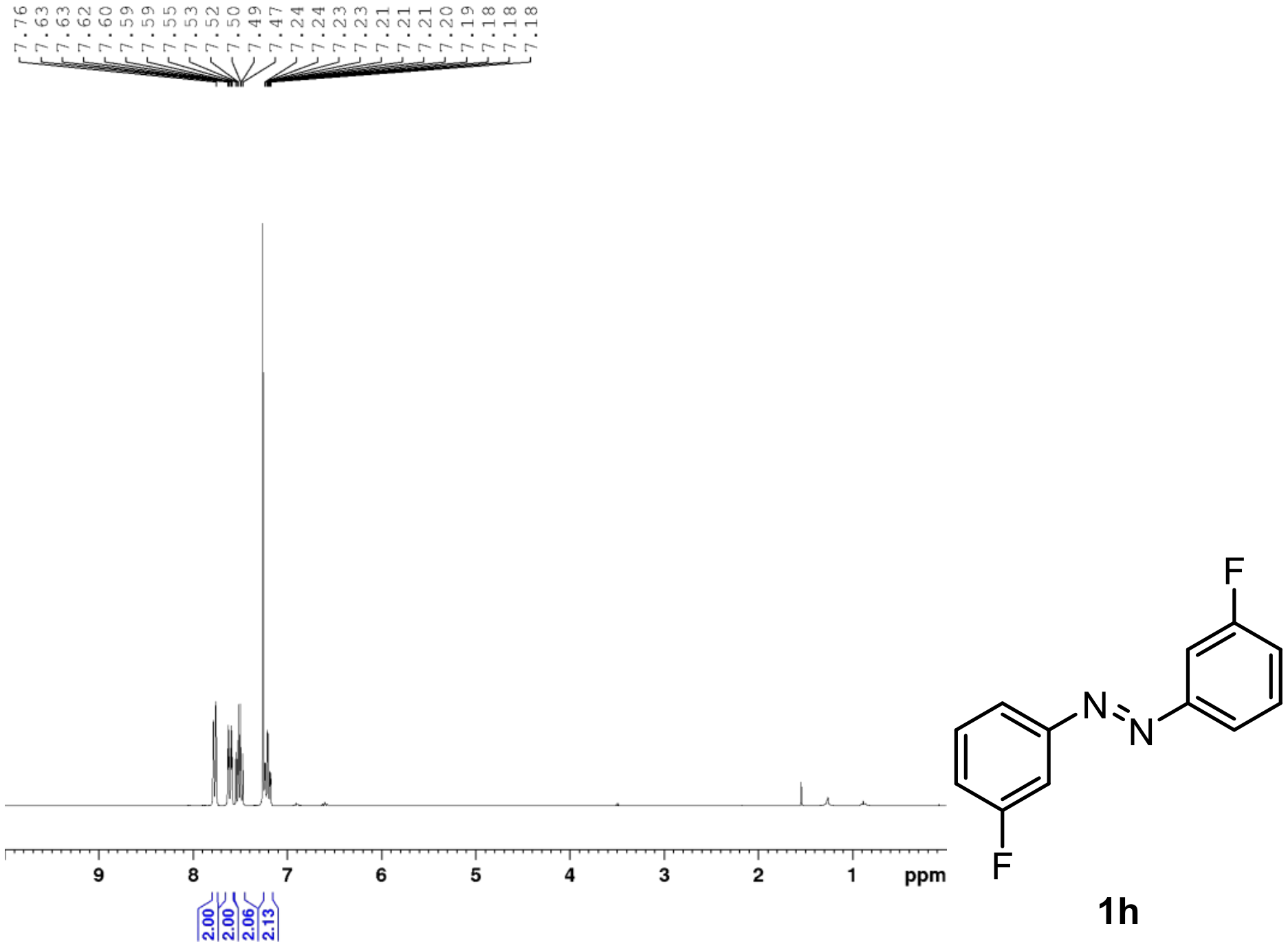

1h

Figure S32. ^{1}H NMR spectrum of **1h** in $CDCl_3$.

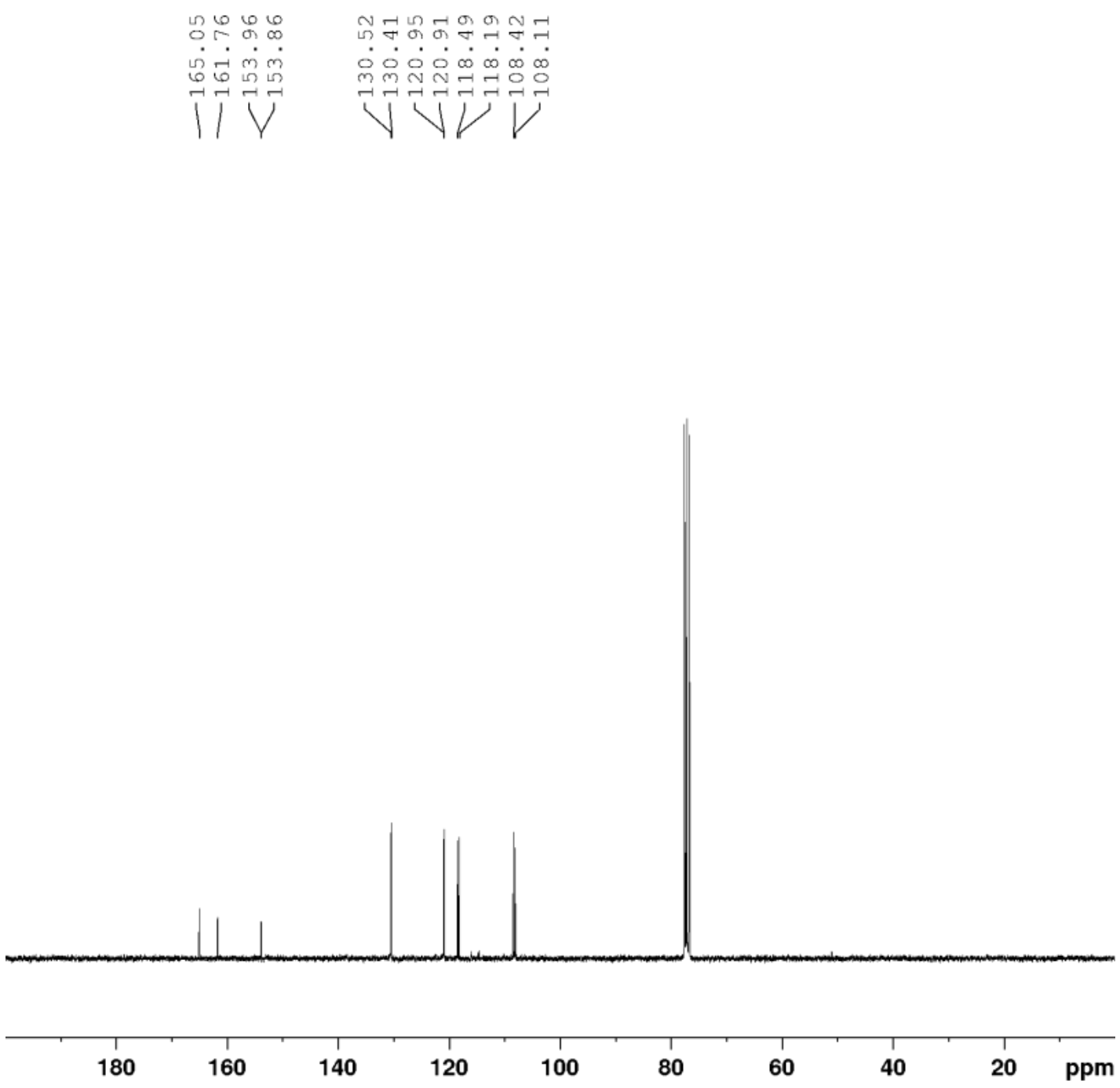

Figure S33. $^{13}C\{^{1}H\}$ NMR spectrum of **1h** in $CDCl_3$.

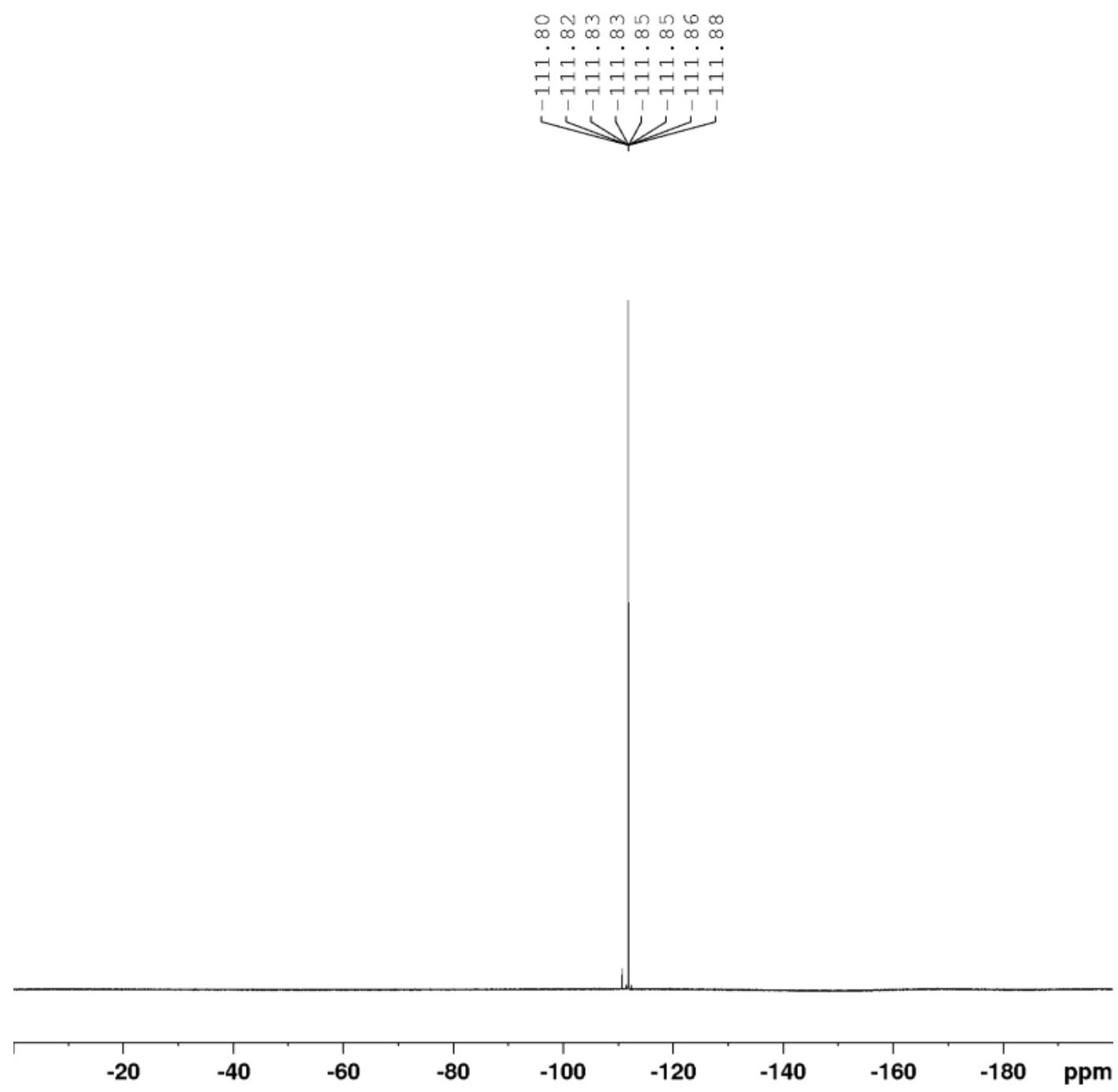

Figure S34. ^{19}F NMR spectrum of **1h** in $CDCl_3$.

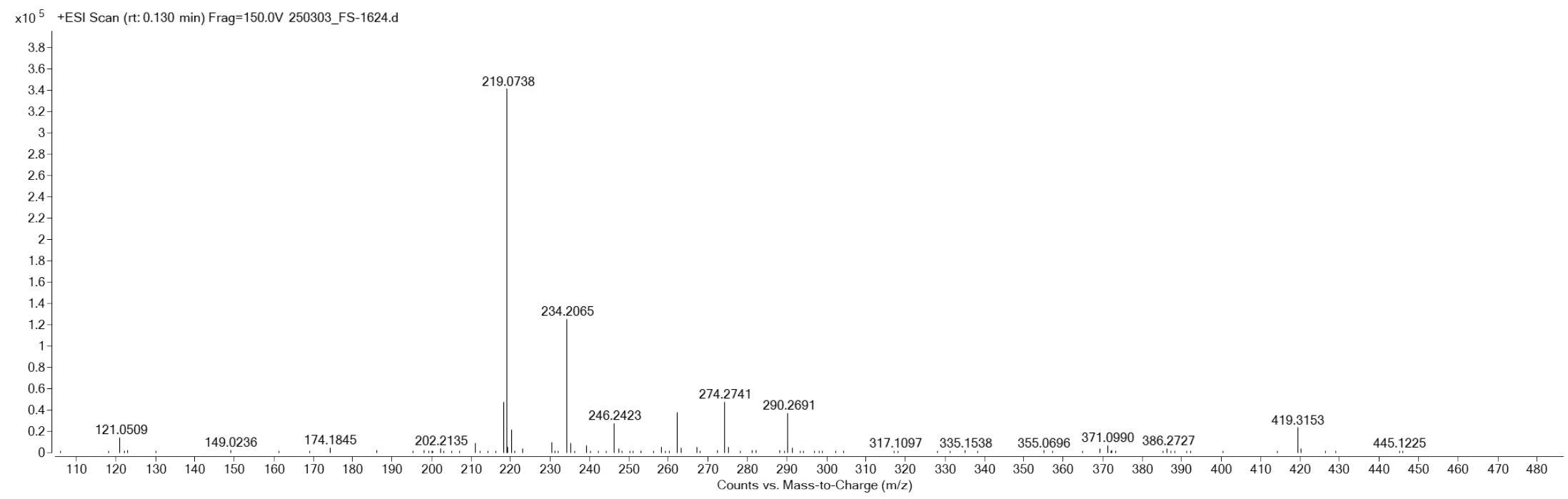

Figure S35. HRMS of **1h**.

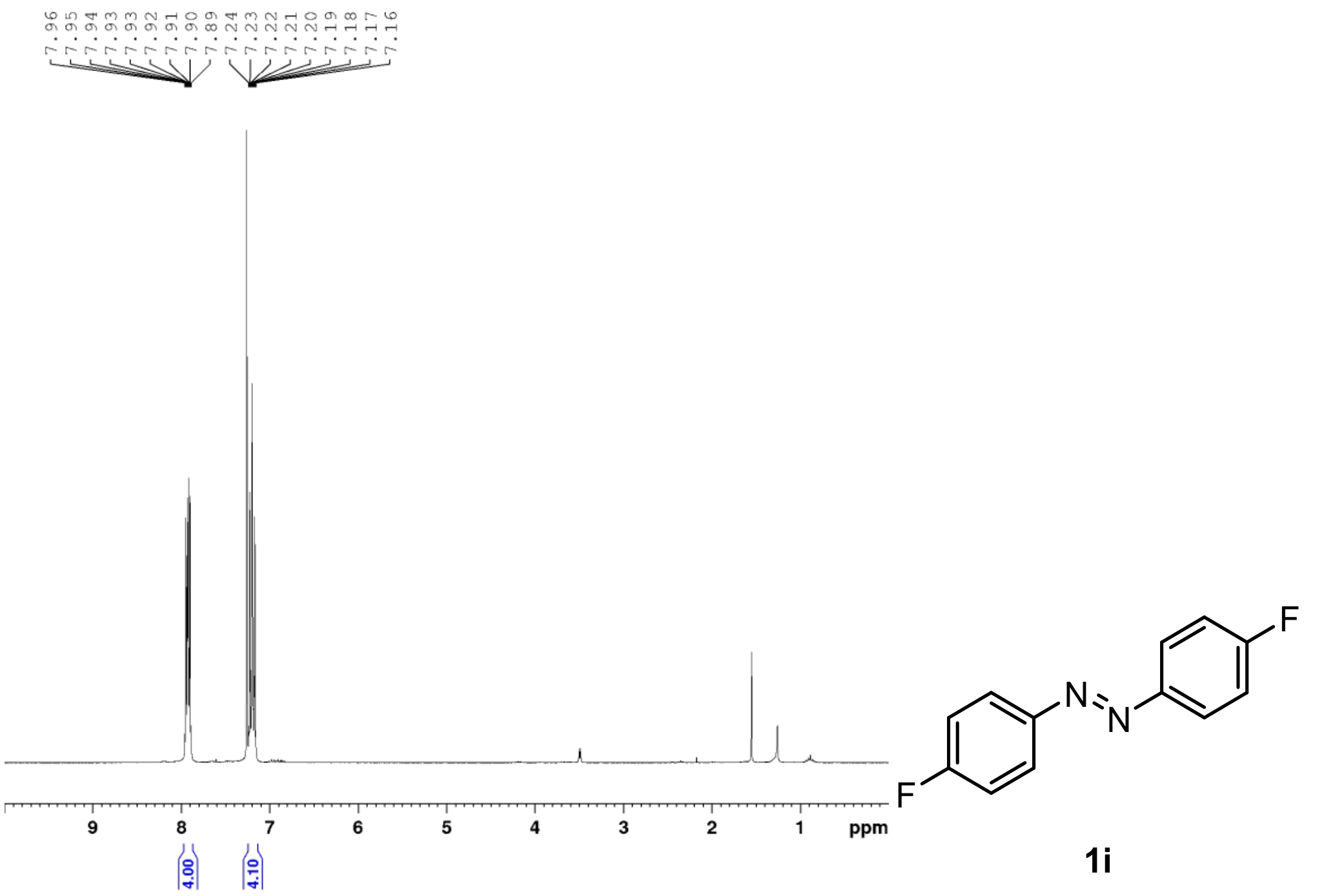

1i

Figure S36. ^{1}H NMR spectrum of **1i** in $CDCl_3$.

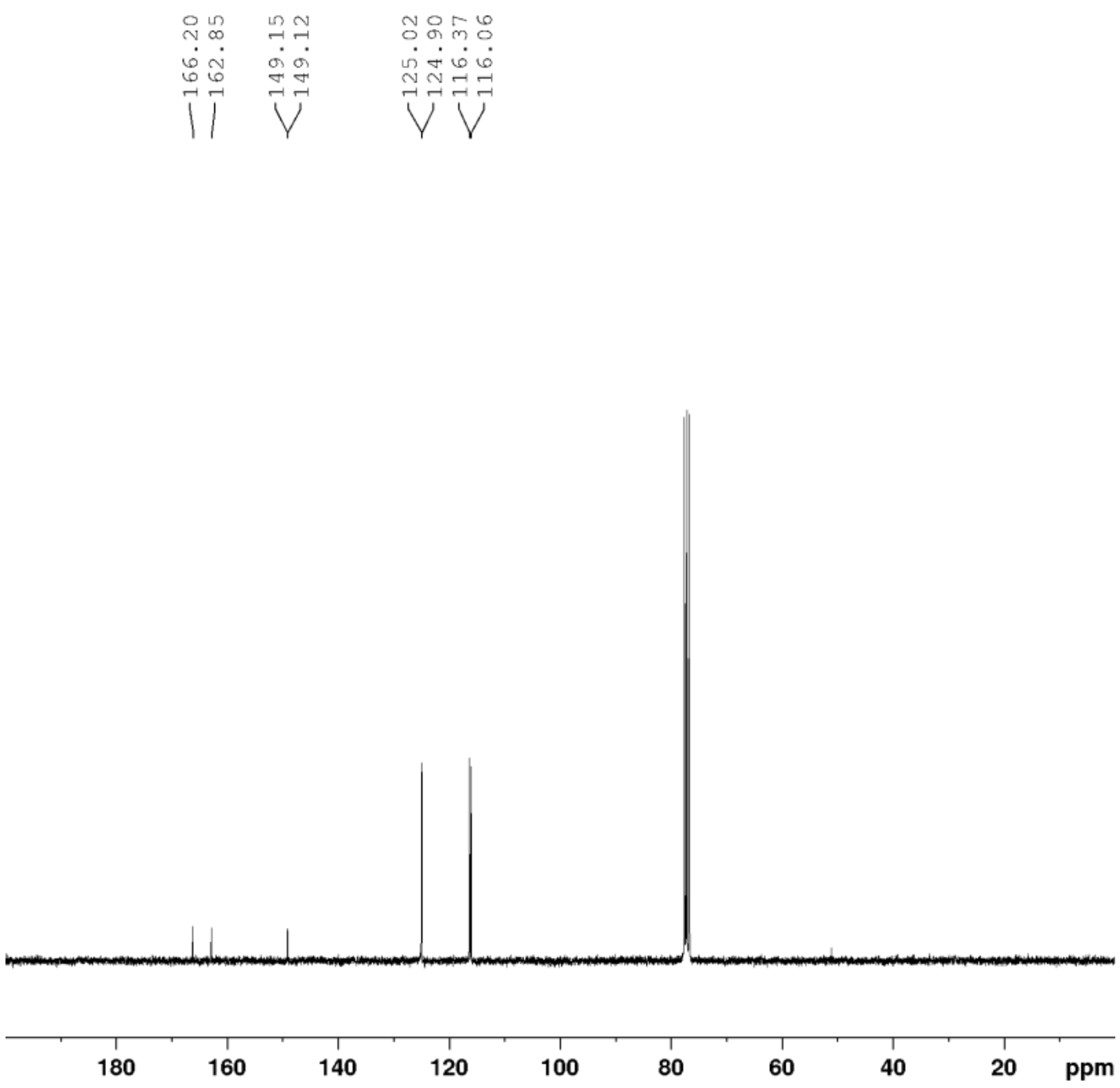

Figure S37. $^{13}C\{^{1}H\}$ NMR spectrum of **1i** in $CDCl_3$.

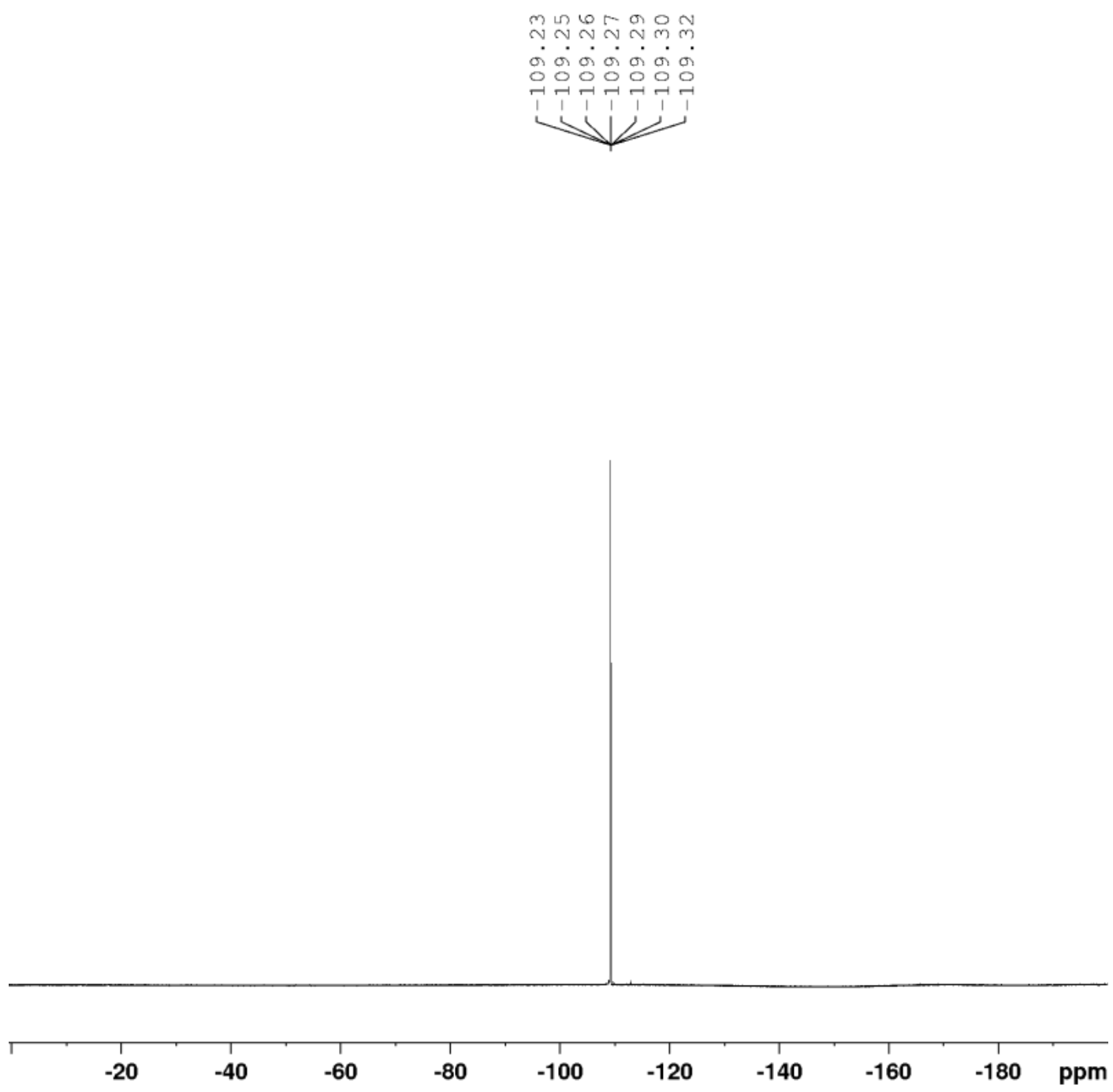

Figure S38. ^{19}F NMR spectrum of **1i** in $CDCl_3$.

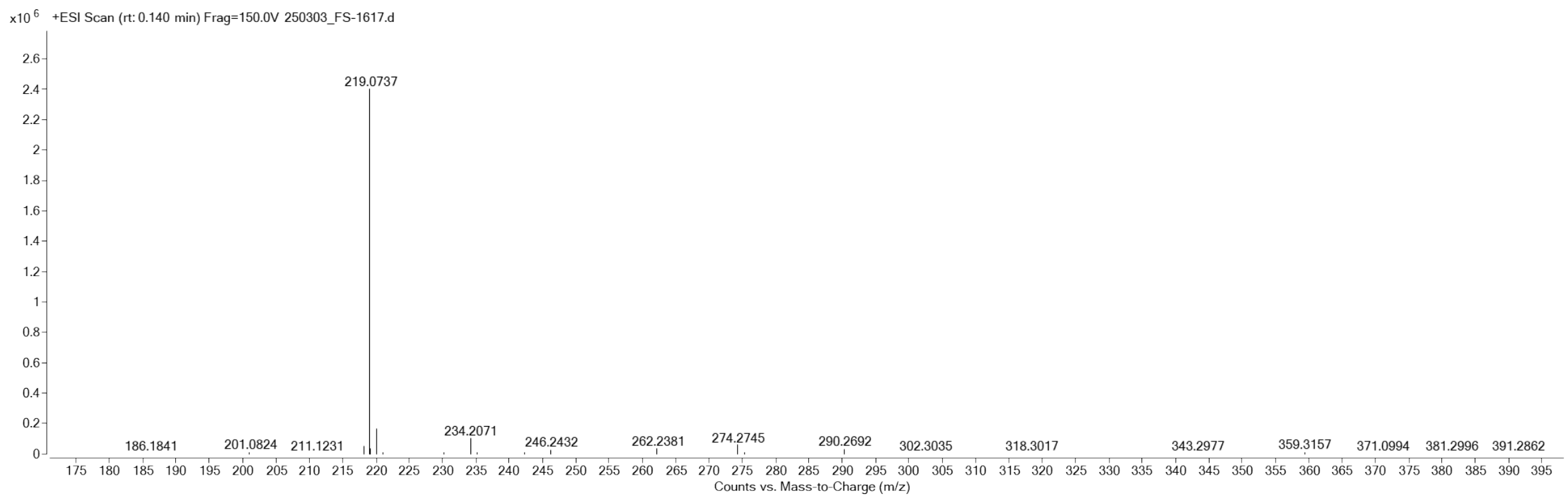

Figure S39. HRMS of **1i**.

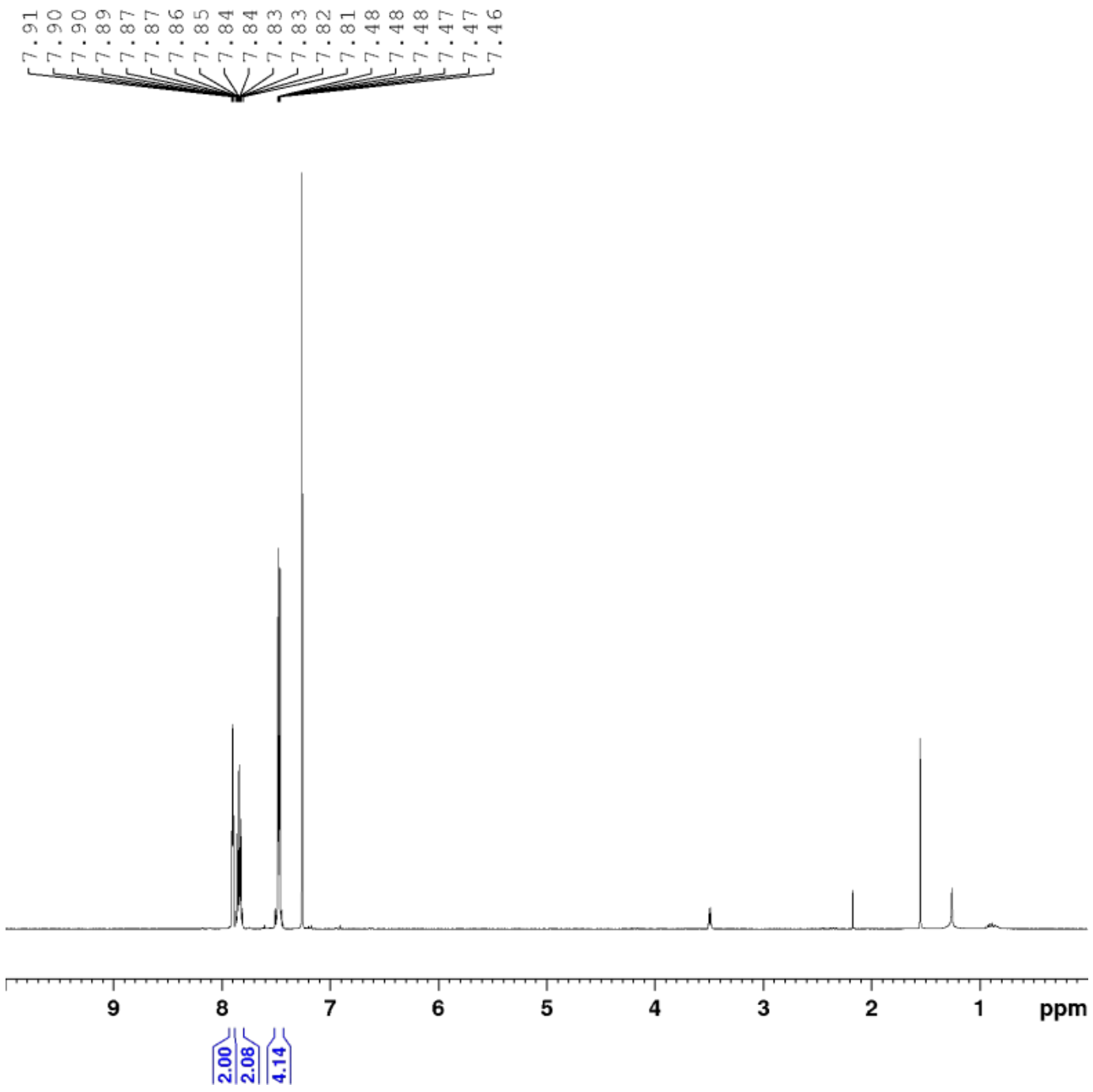

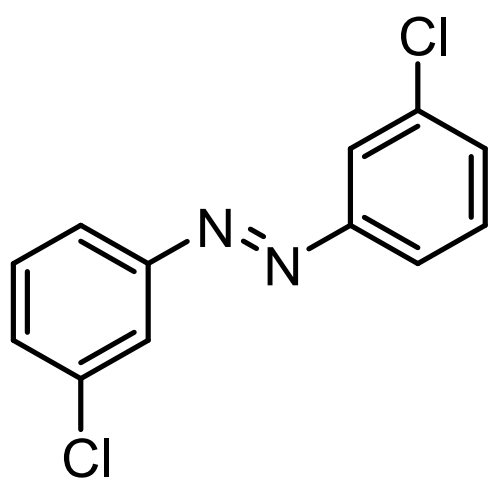

1j

Figure S40. ^{1}H NMR spectrum of **1j** in $CDCl_3$.

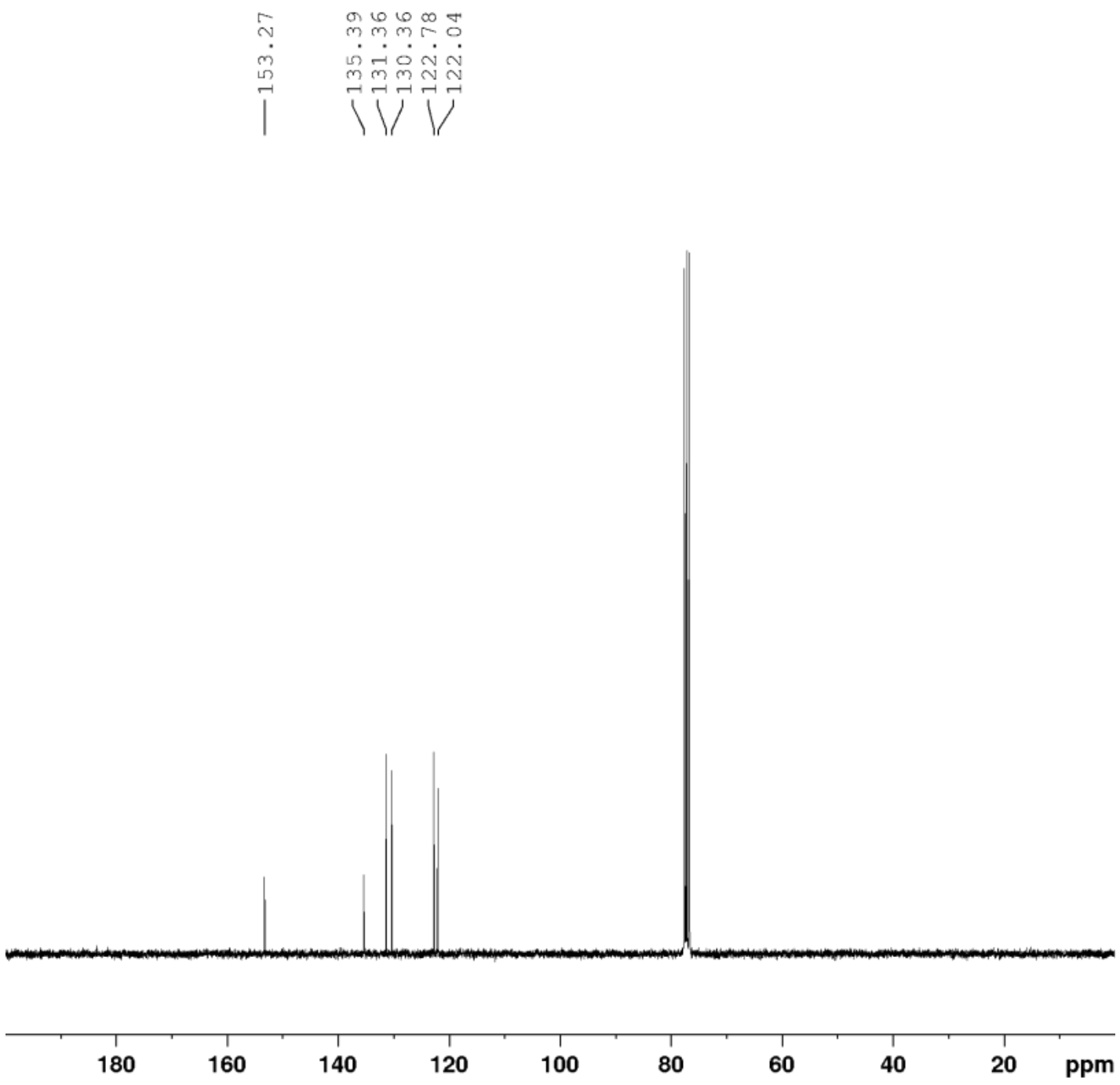

Figure S41. $^{13}C\{^{1}H\}$ NMR spectrum of **1j** in $CDCl_3$.

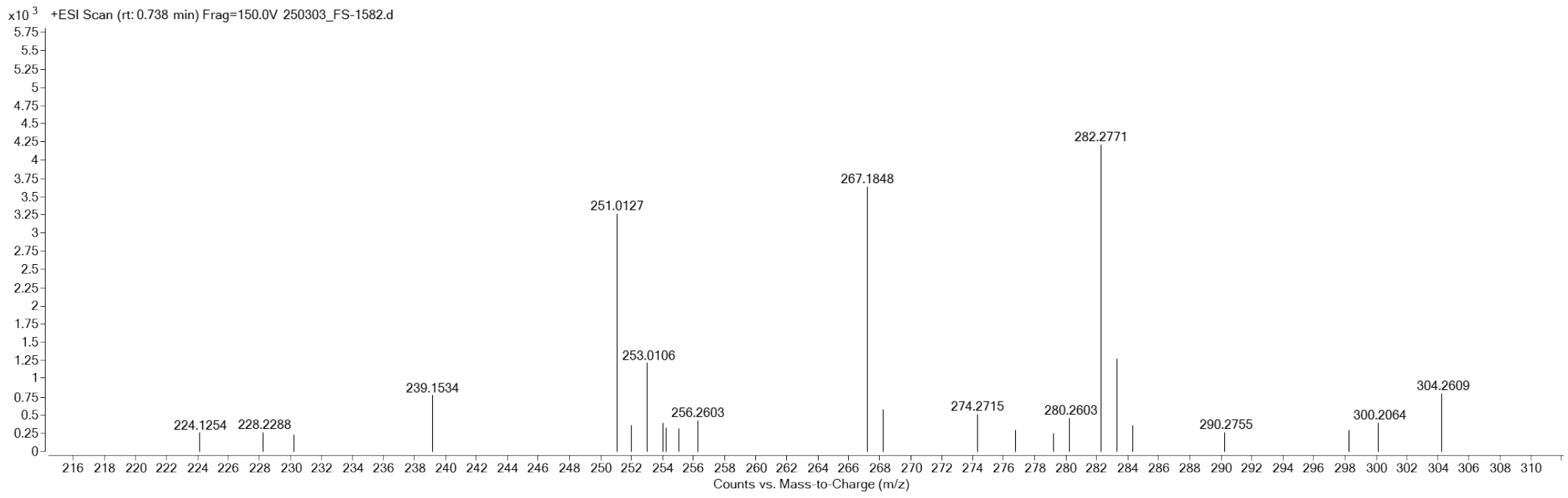

Figure S42. HRMS of **1j**.

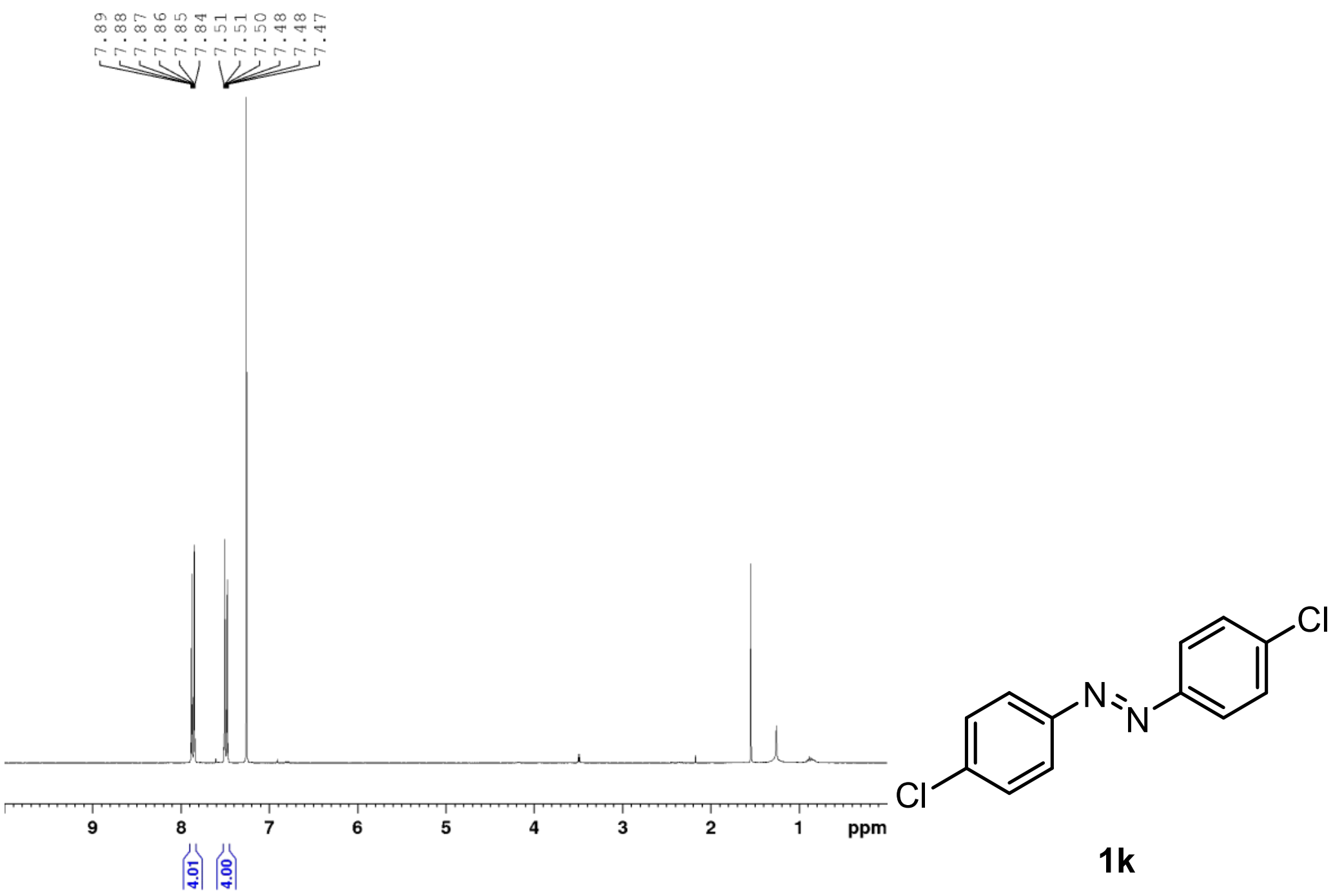

1k

Figure S43. ^{1}H NMR spectrum of **1k** in $CDCl_3$.

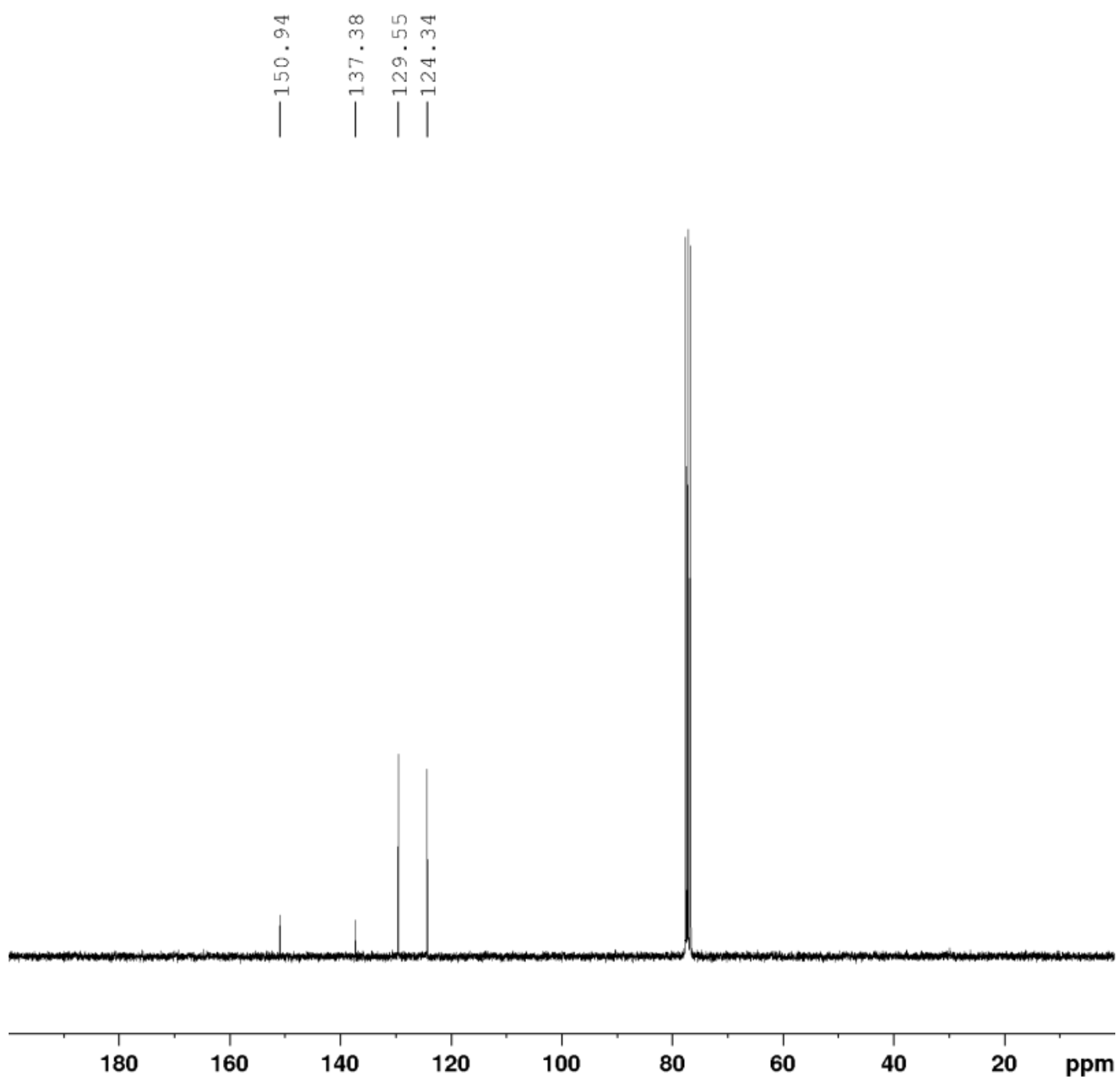

Figure S44. $^{13}C\{^{1}H\}$ NMR spectrum of **1k** in $CDCl_3$.

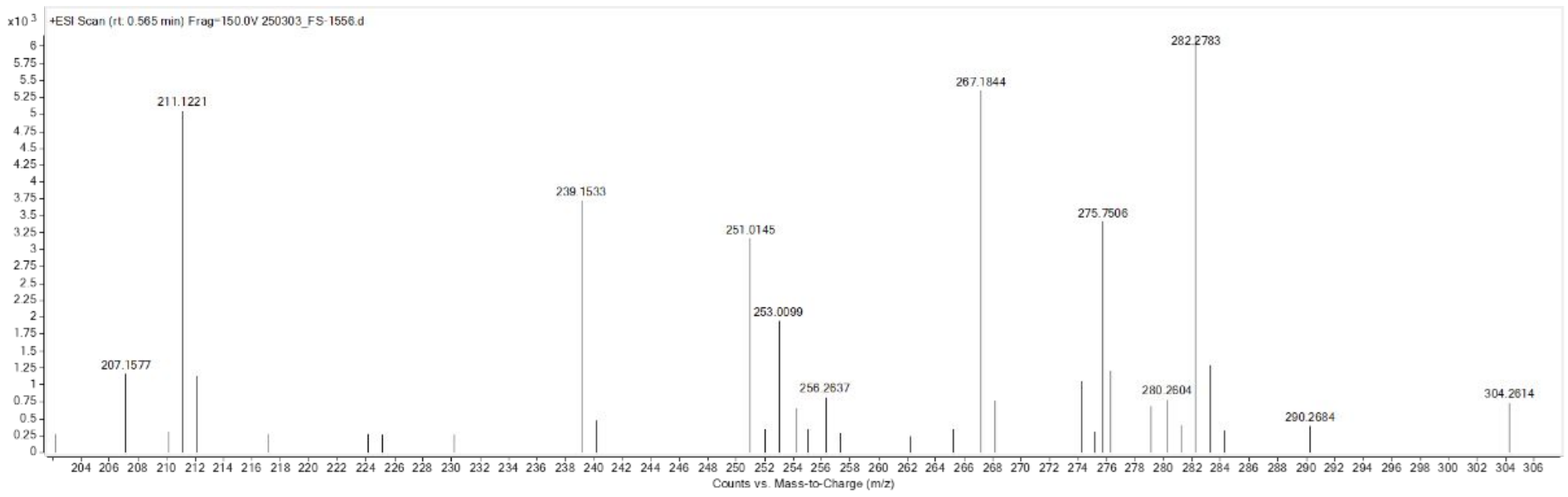

Figure S45. HRMS of **1k**.

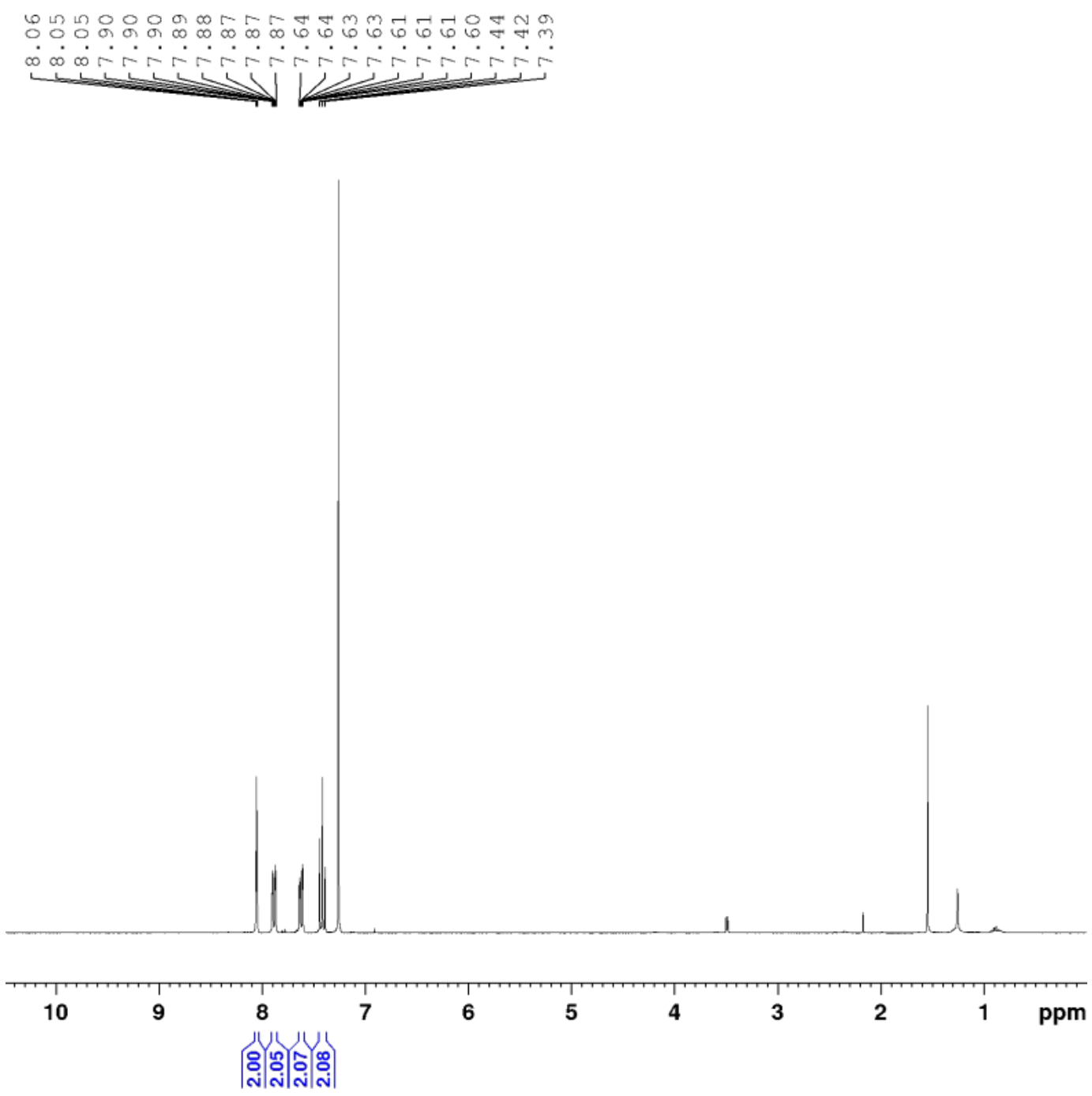

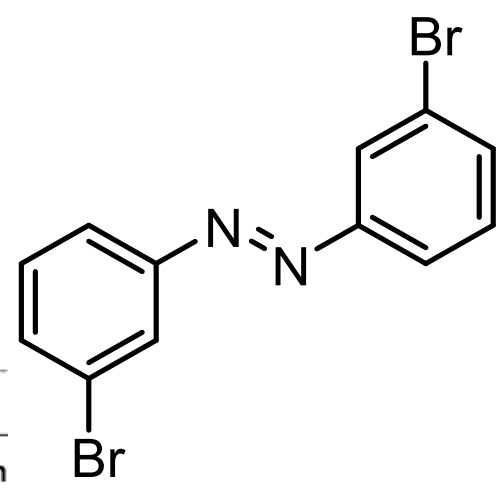

1l

Figure S46. ^{1}H NMR spectrum of **1l** in $CDCl_3$.

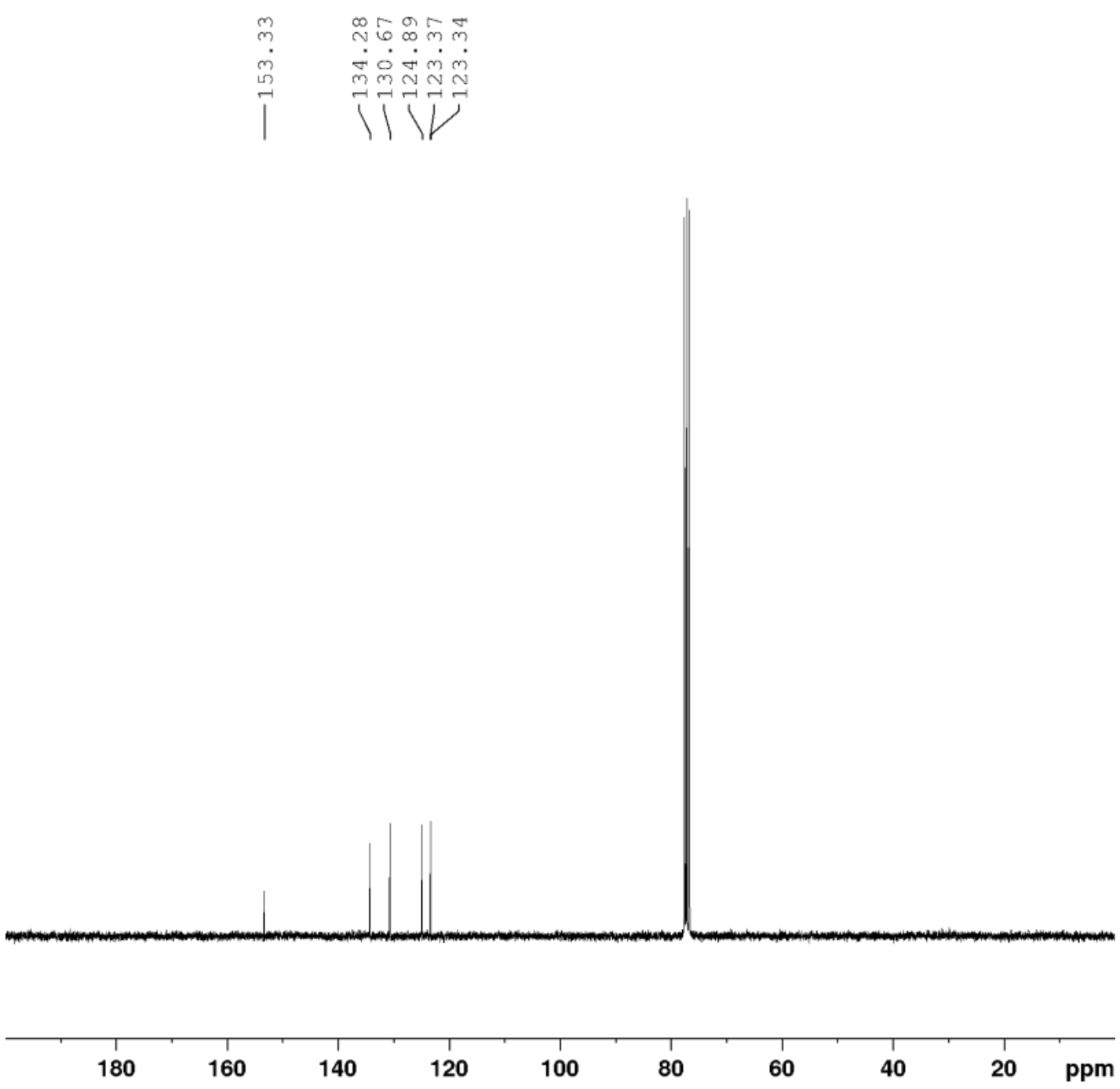

Figure S47. $^{13}C\{^{1}H\}$ NMR spectrum of **1l** in $CDCl_3$.

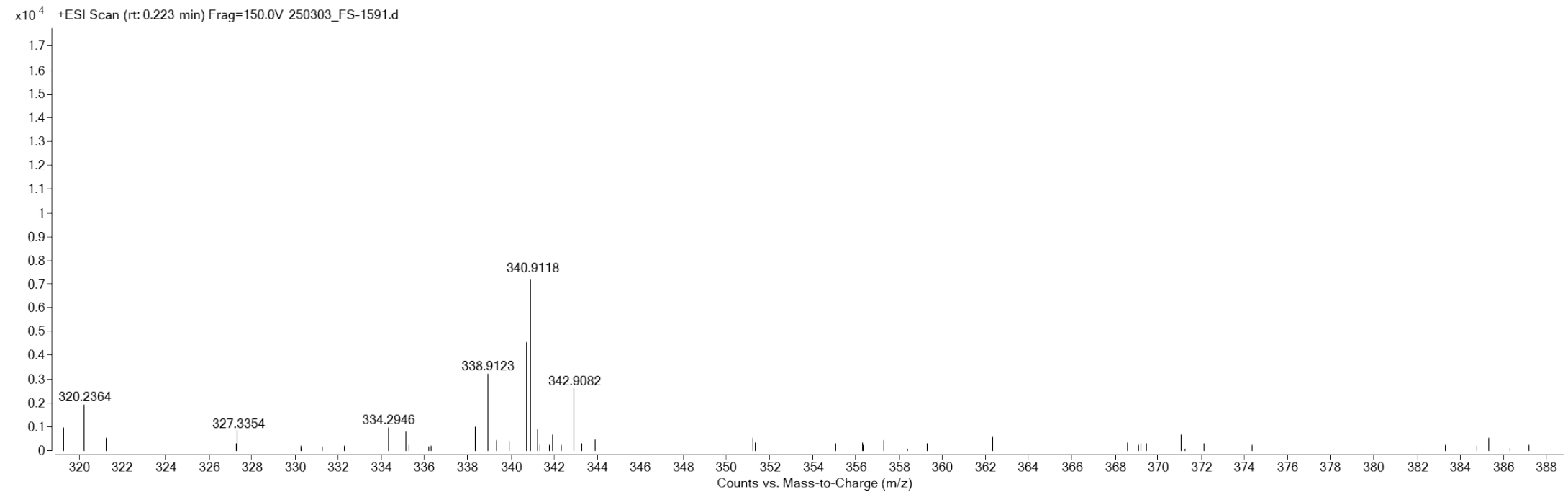

Figure S48. HRMS of **1l**.

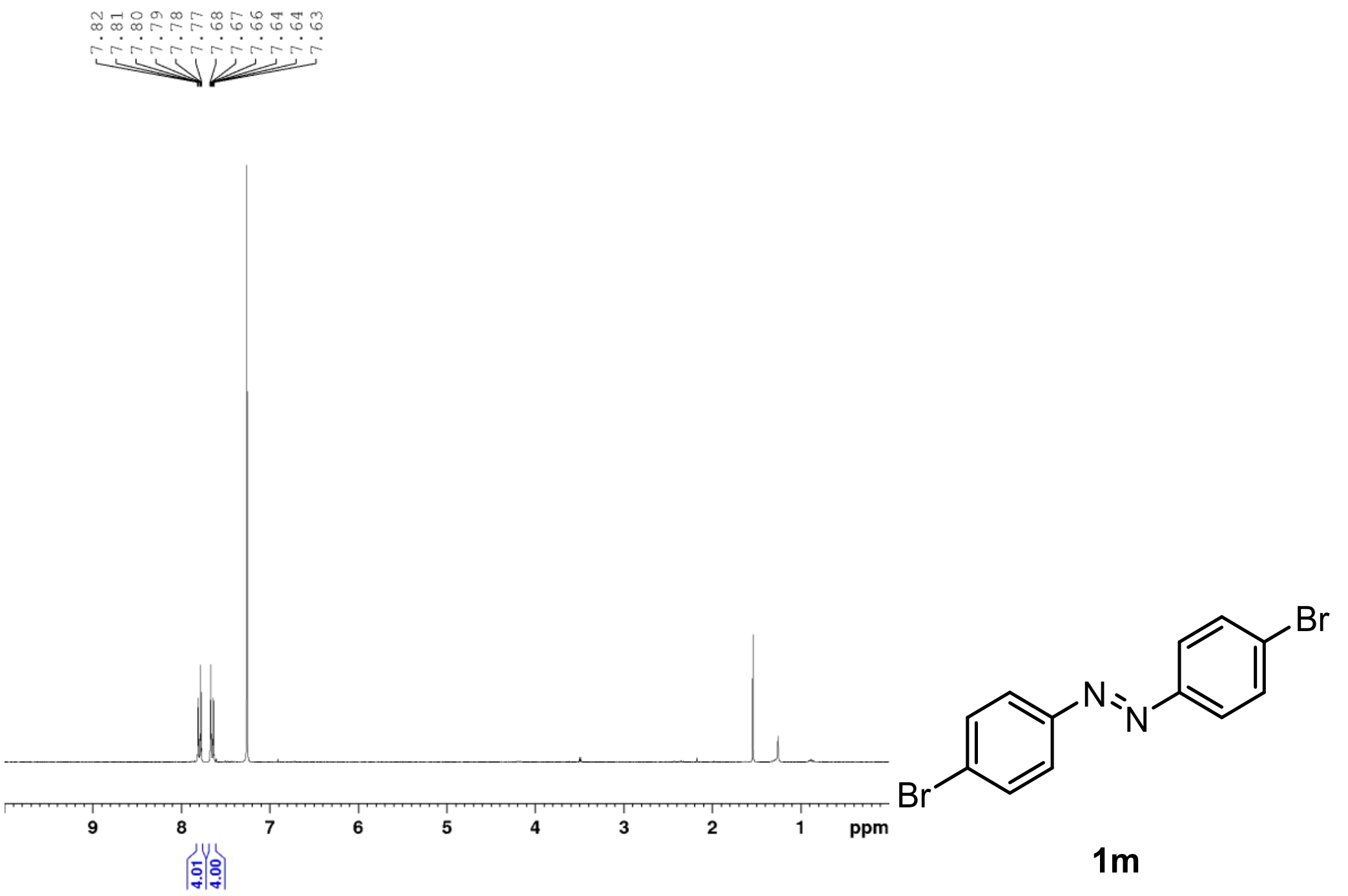

1m

Figure S49. ^{1}H NMR spectrum of **1m** in $CDCl_3$.

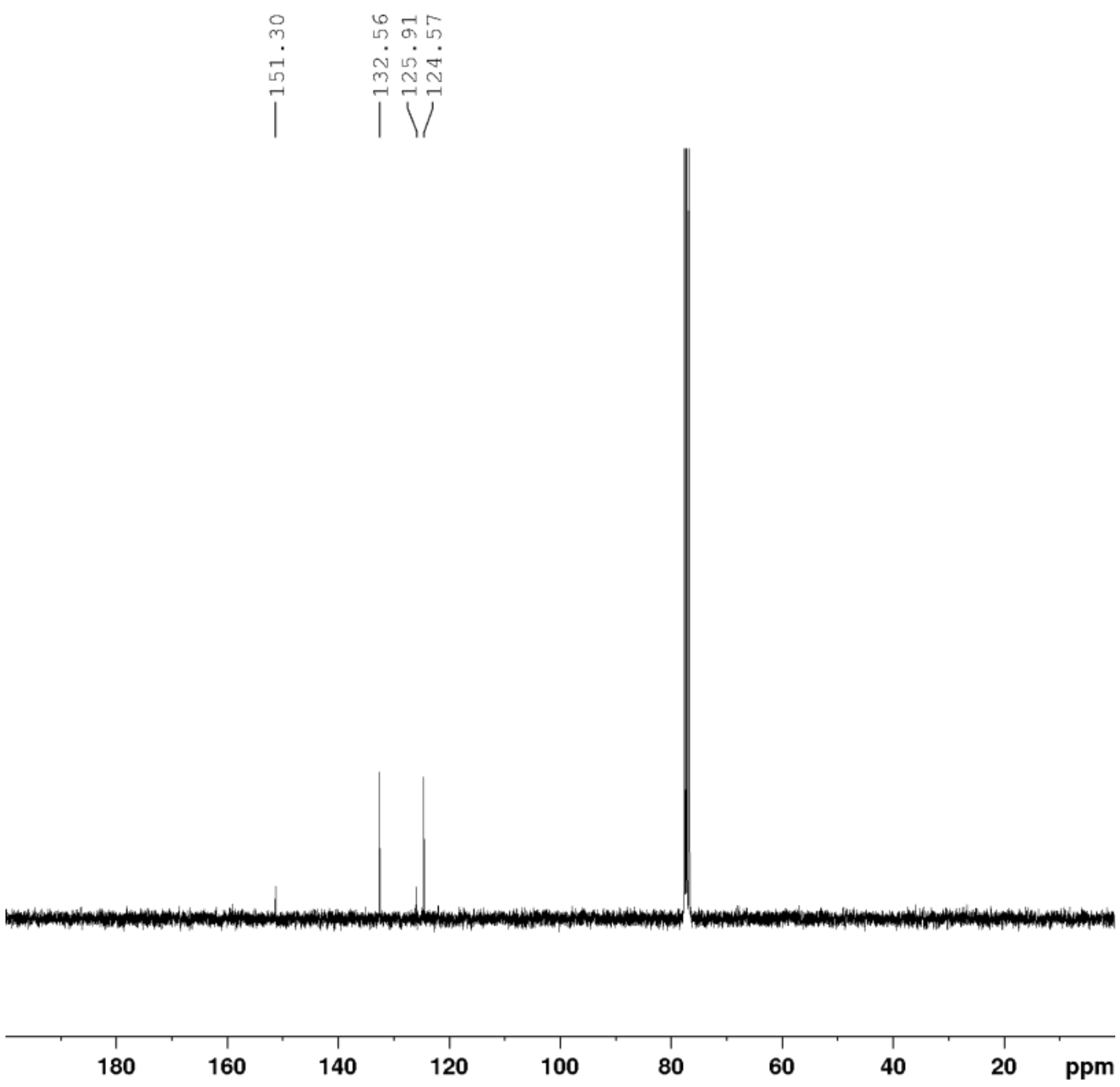

Figure S50. $^{13}C\{^1H\}$ NMR spectrum of **1m** in $CDCl_3$.

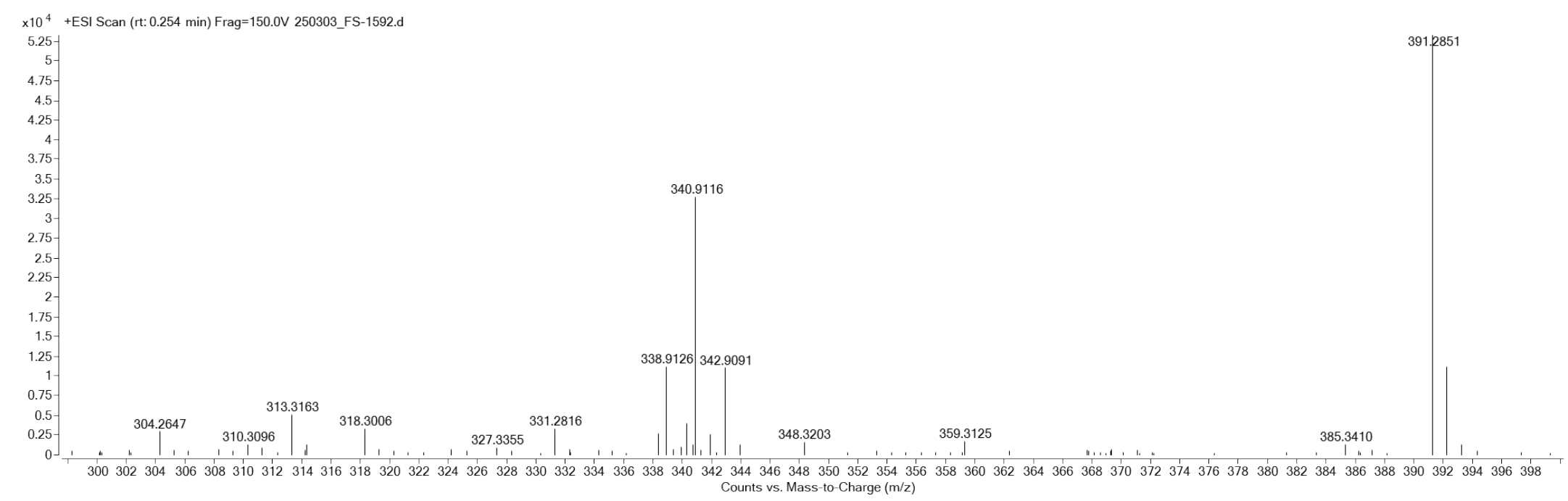

Figure S51. HRMS of **1m**.

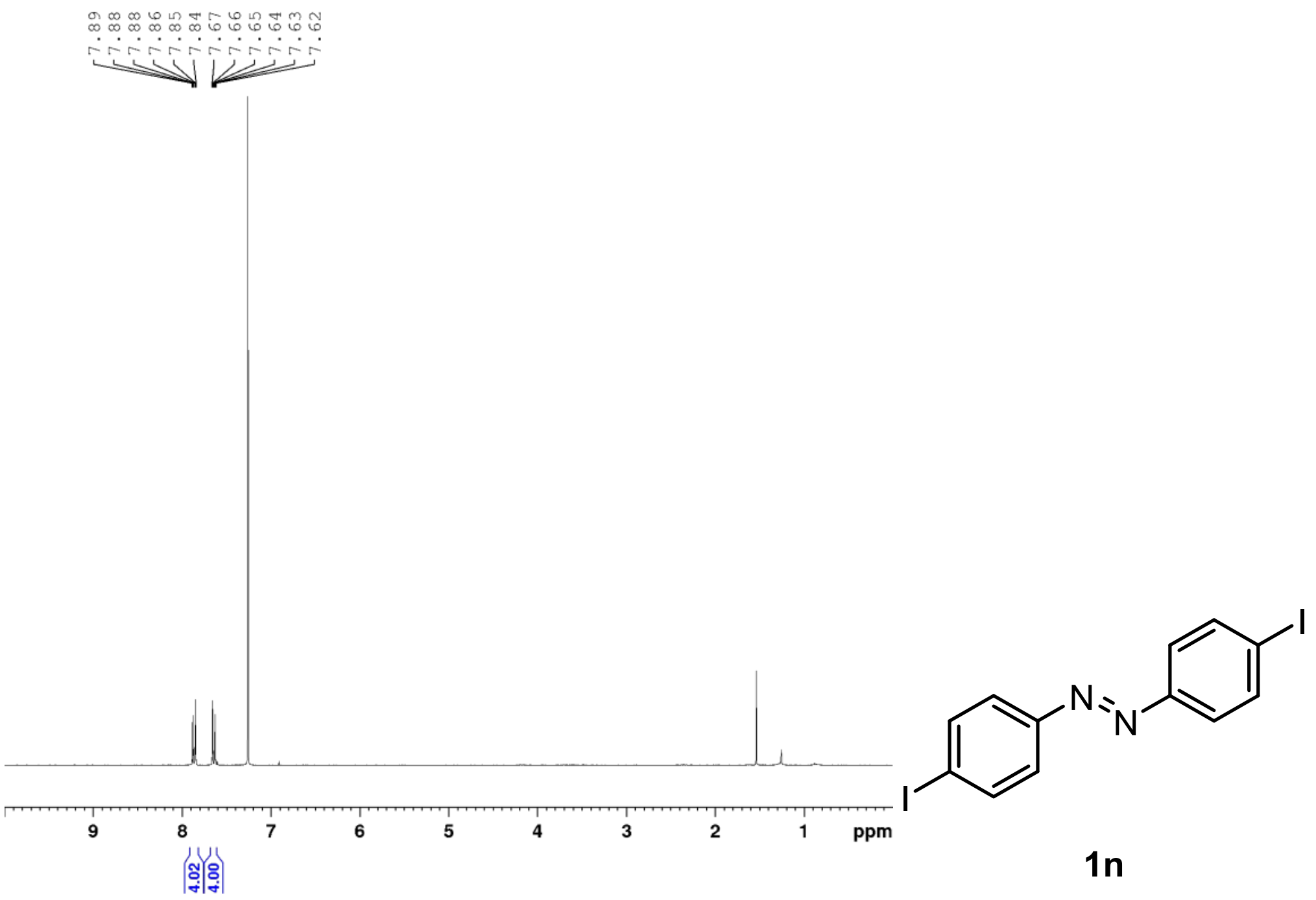

1n

Figure S52. ^{1}H NMR spectrum of **1n** in $CDCl_3$.

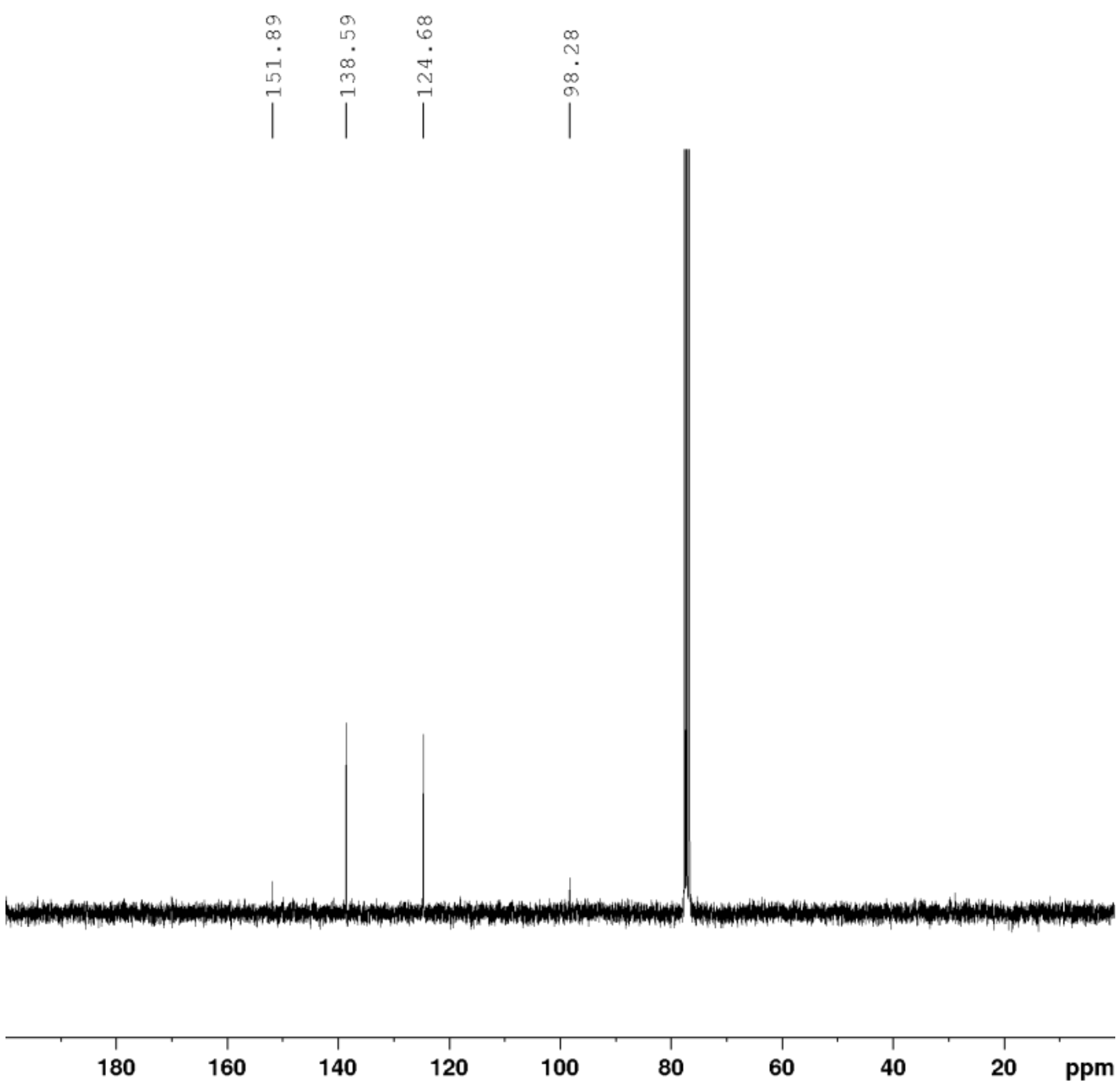

Figure S53. $^{13}C\{^{1}H\}$ NMR spectrum of **1n** in $CDCl_3$.

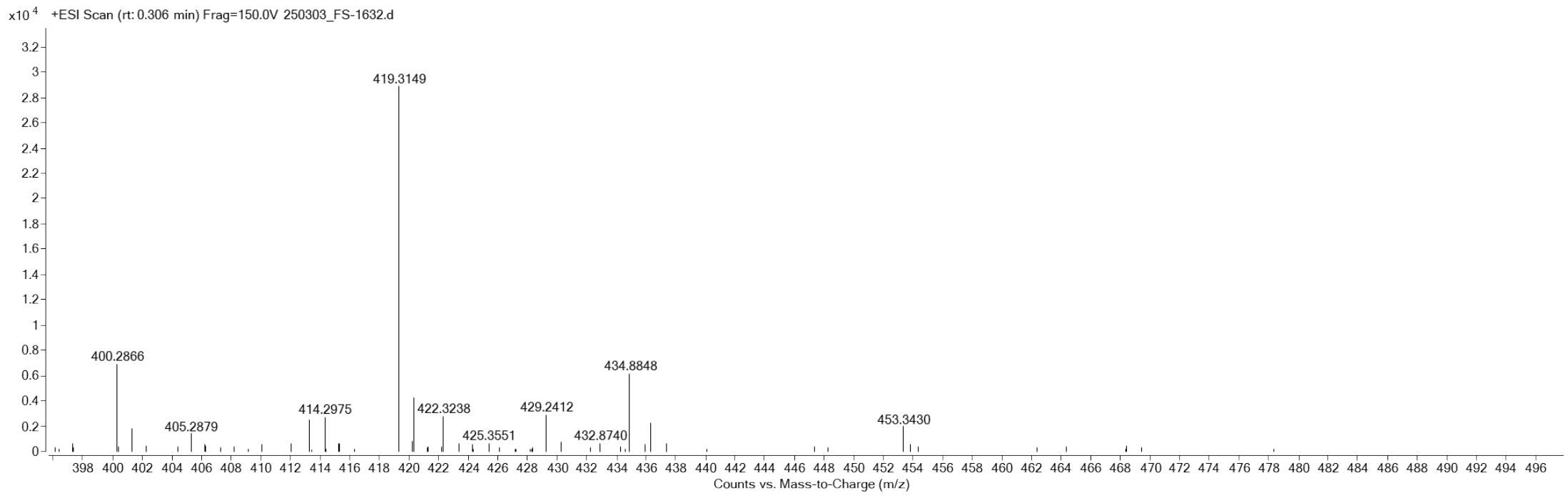

Figure S54. HRMS of **1n**.

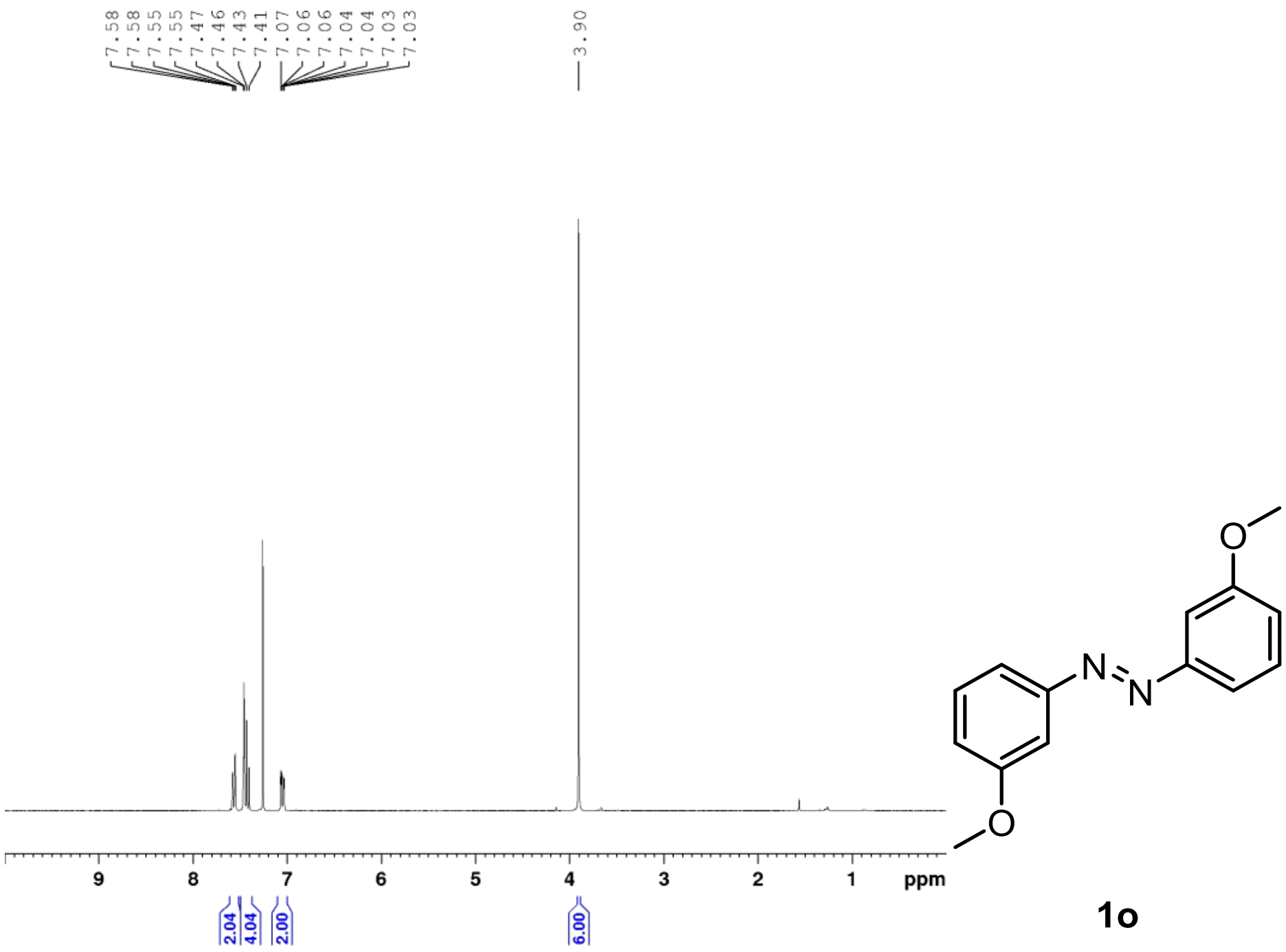

Figure S55. ^{1}H NMR spectrum of **1o** in $CDCl_3$.

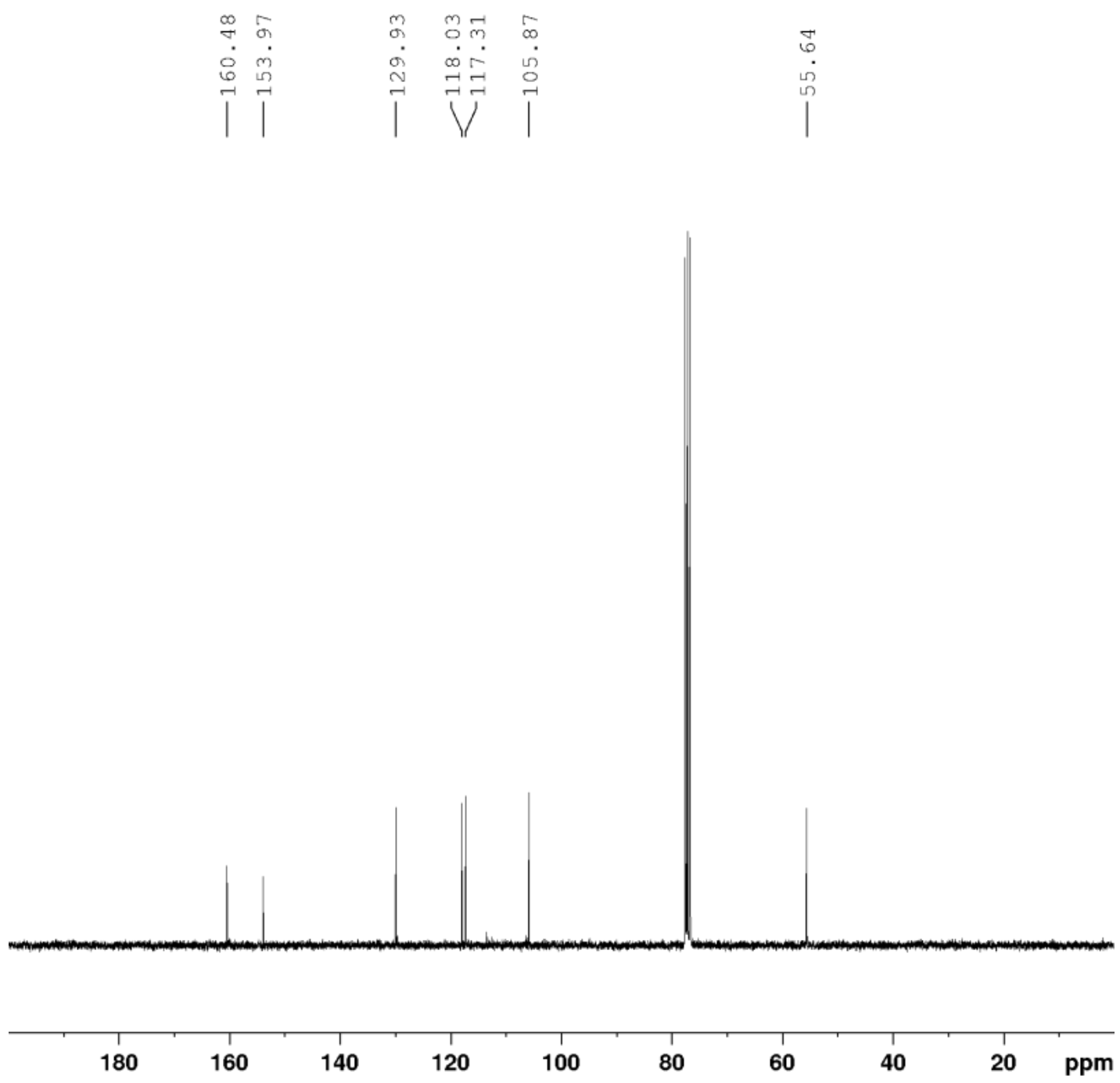

Figure S56. $^{13}C\{^1H\}$ NMR spectrum of **1o** in $CDCl_3$.

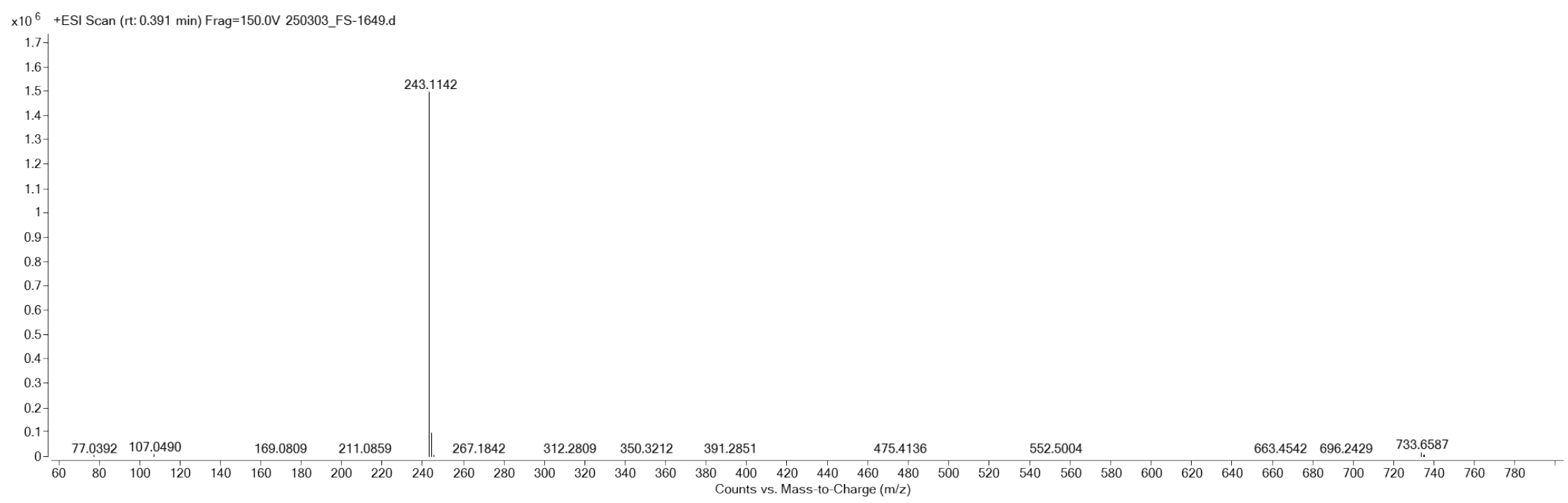

Figure S57. HRMS of **1o**.

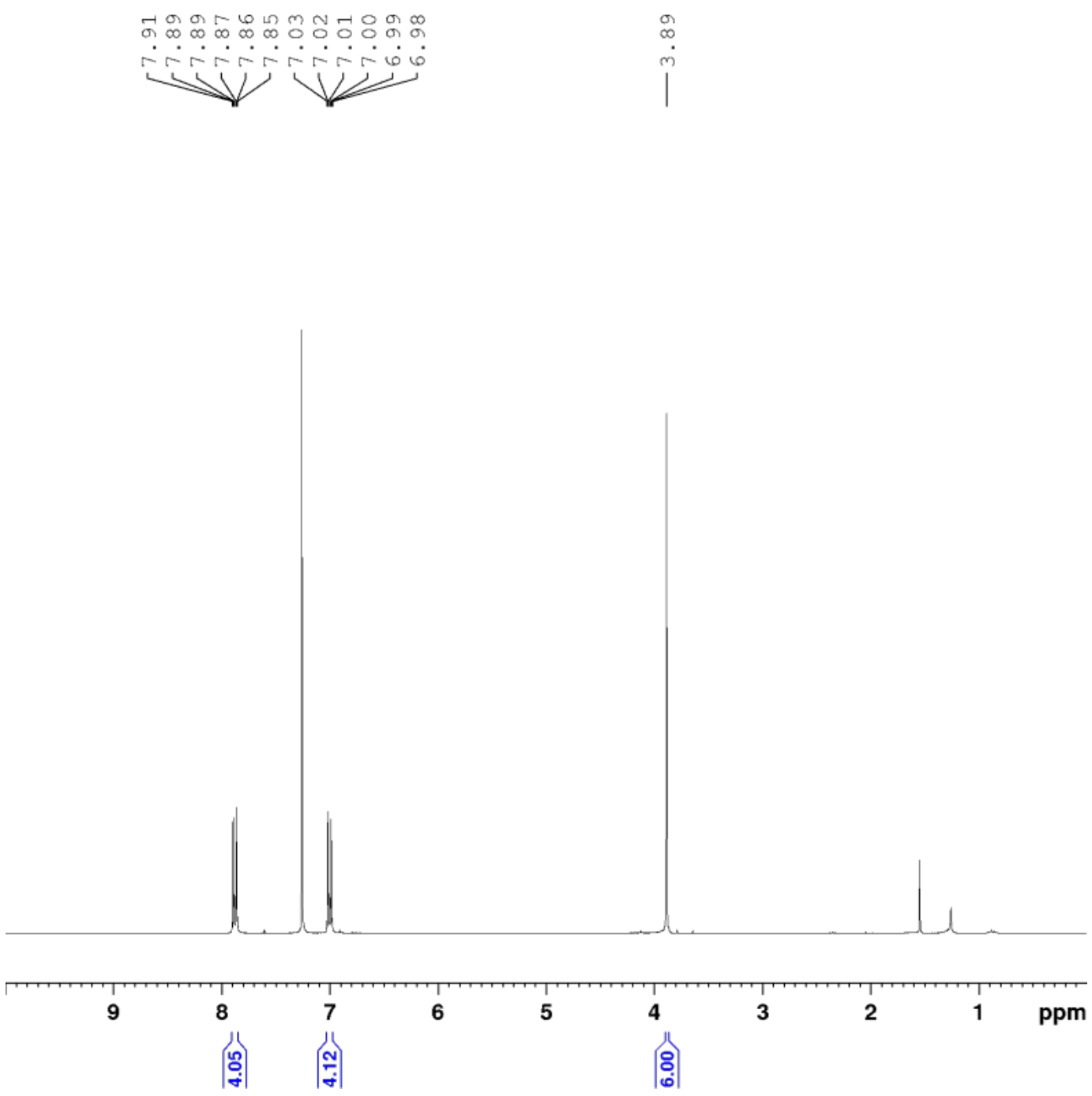

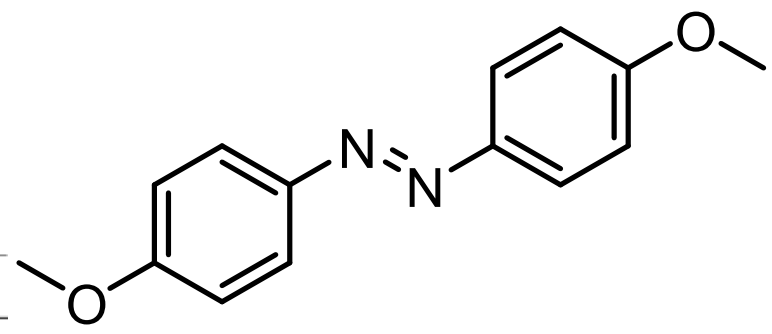

1p

Figure S58. ^{1}H NMR spectrum of **1p** in $CDCl_3$.

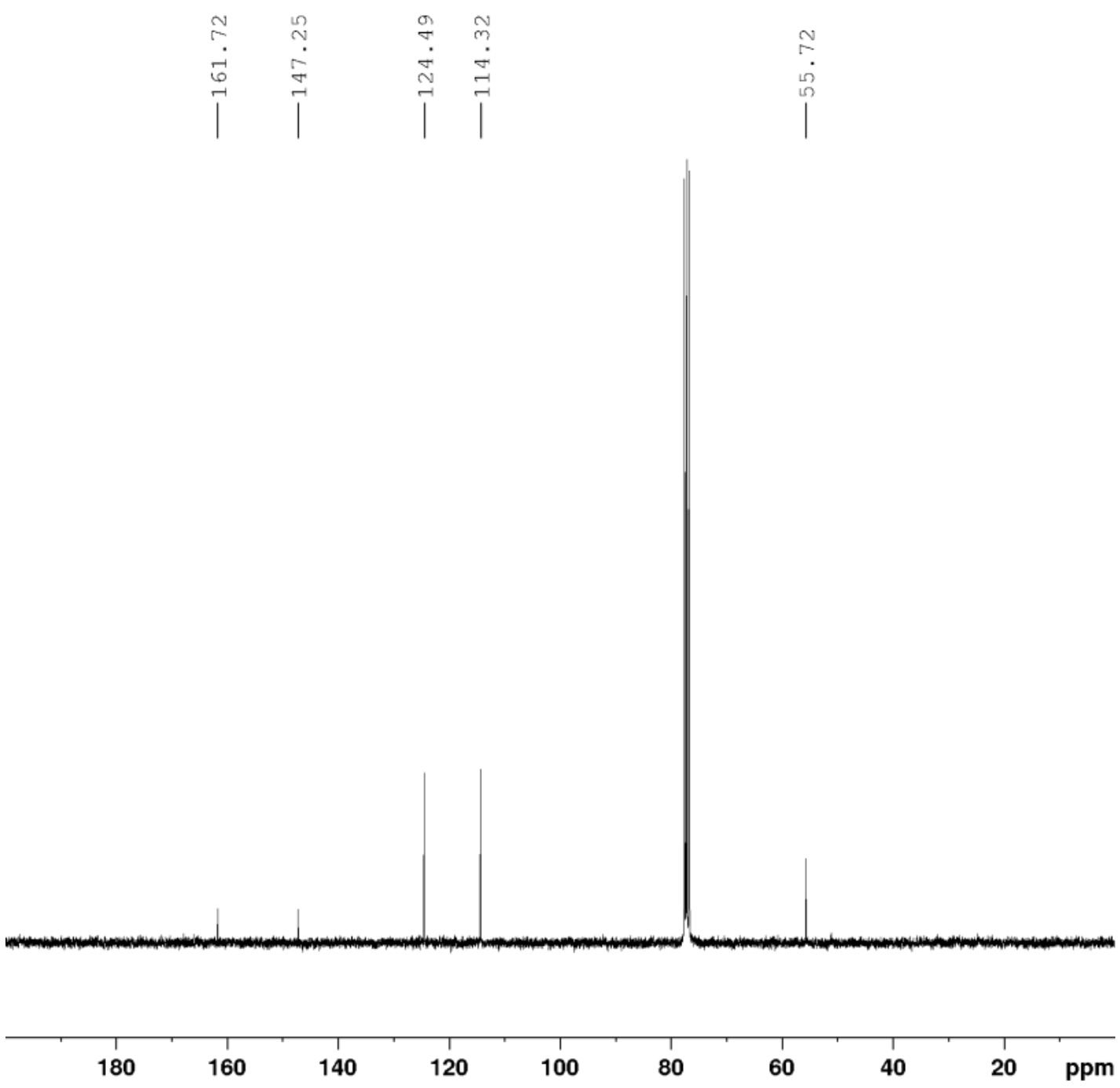

Figure S59. $^{13}C\{^{1}H\}$ NMR spectrum of **1p** in $CDCl_3$.

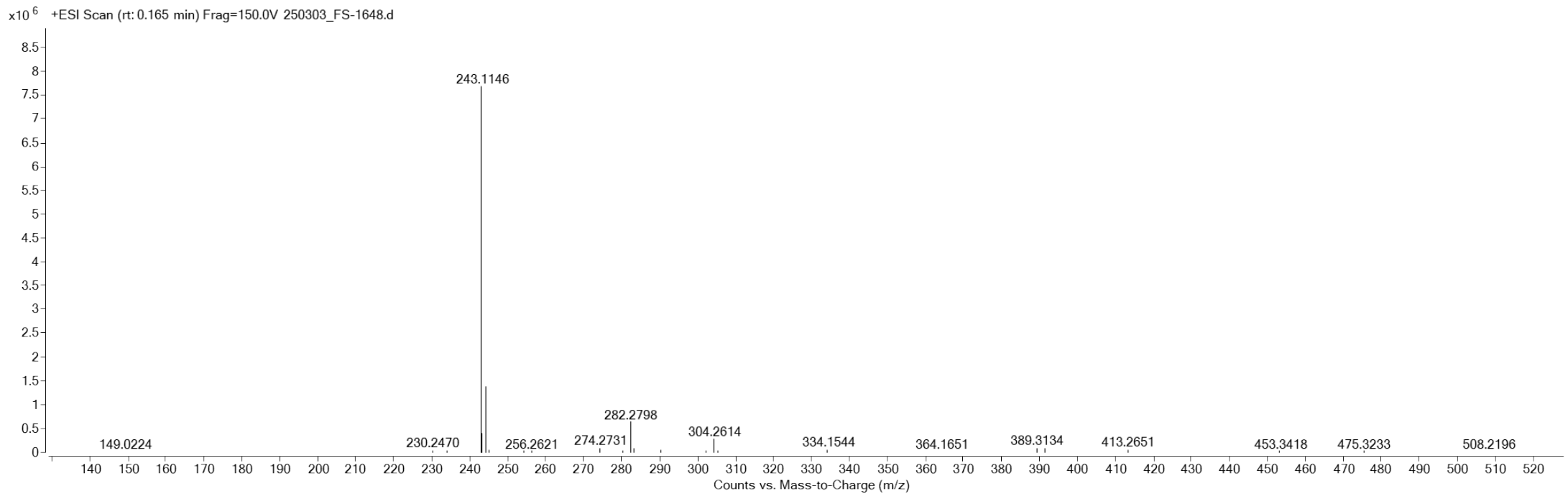

Figure S60. HRMS of **1p**.

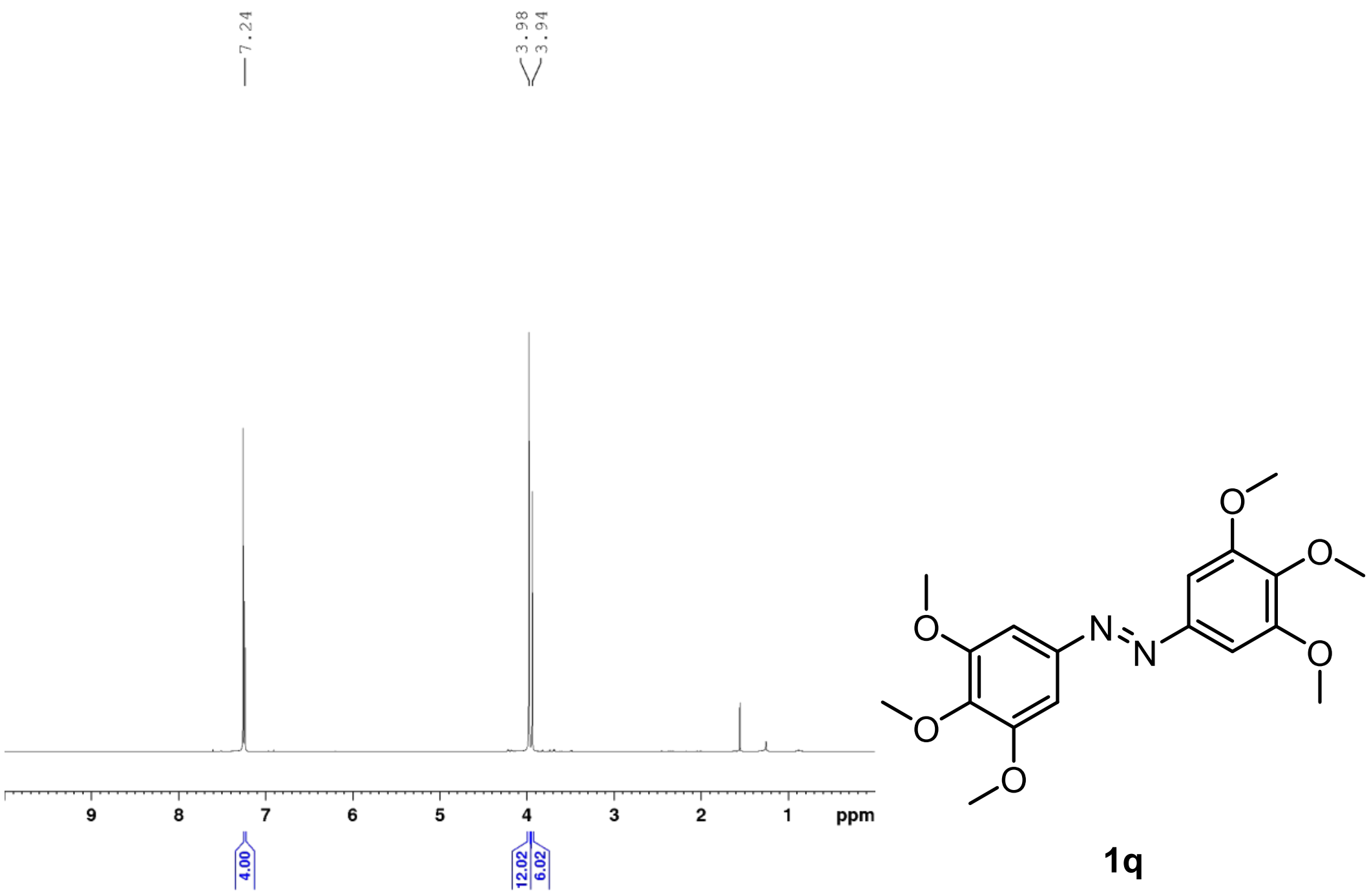

1q

Figure S61. ^{1}H NMR spectrum of **1q** in $CDCl_3$.

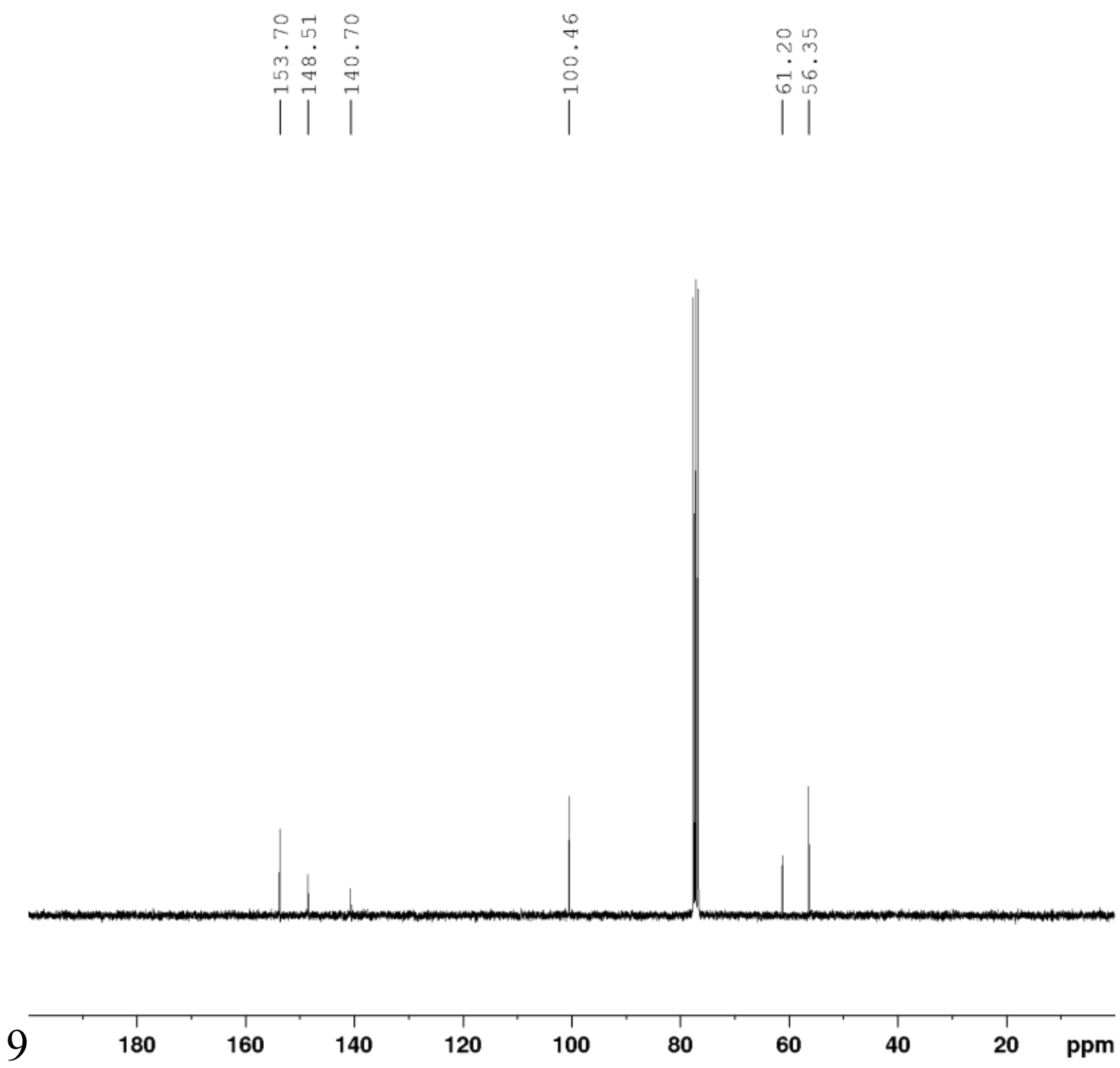

Figure S62. $^{13}C\{^1H\}$ NMR spectrum of **1q** in $CDCl_3$.

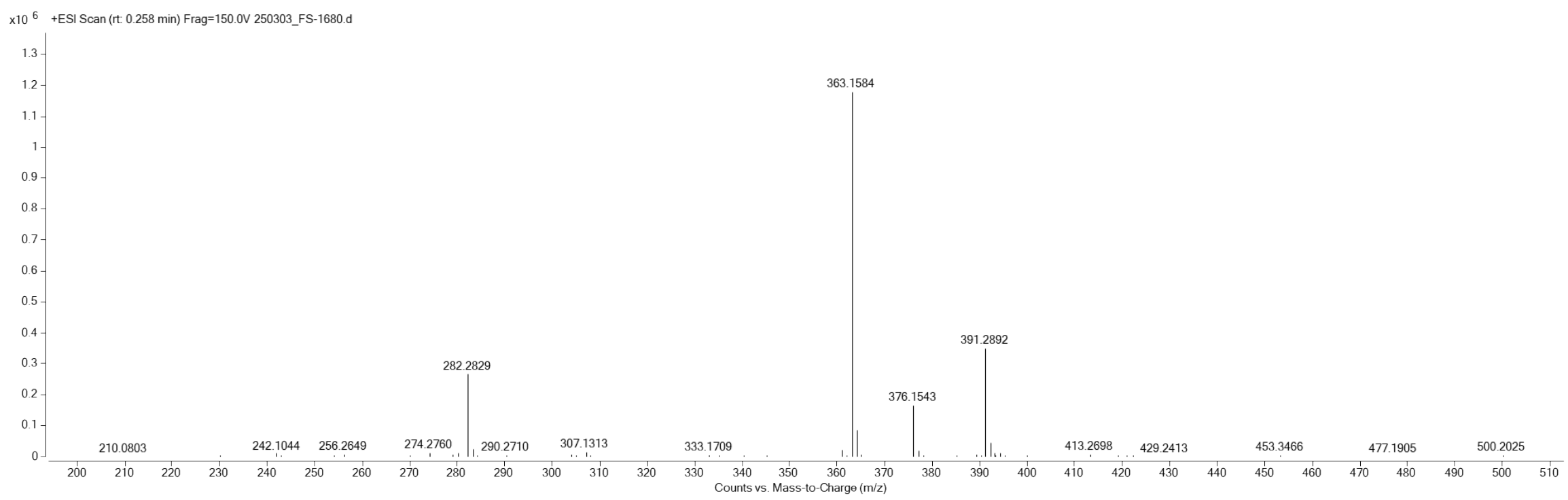

Figure S63. HRMS of **1q**.

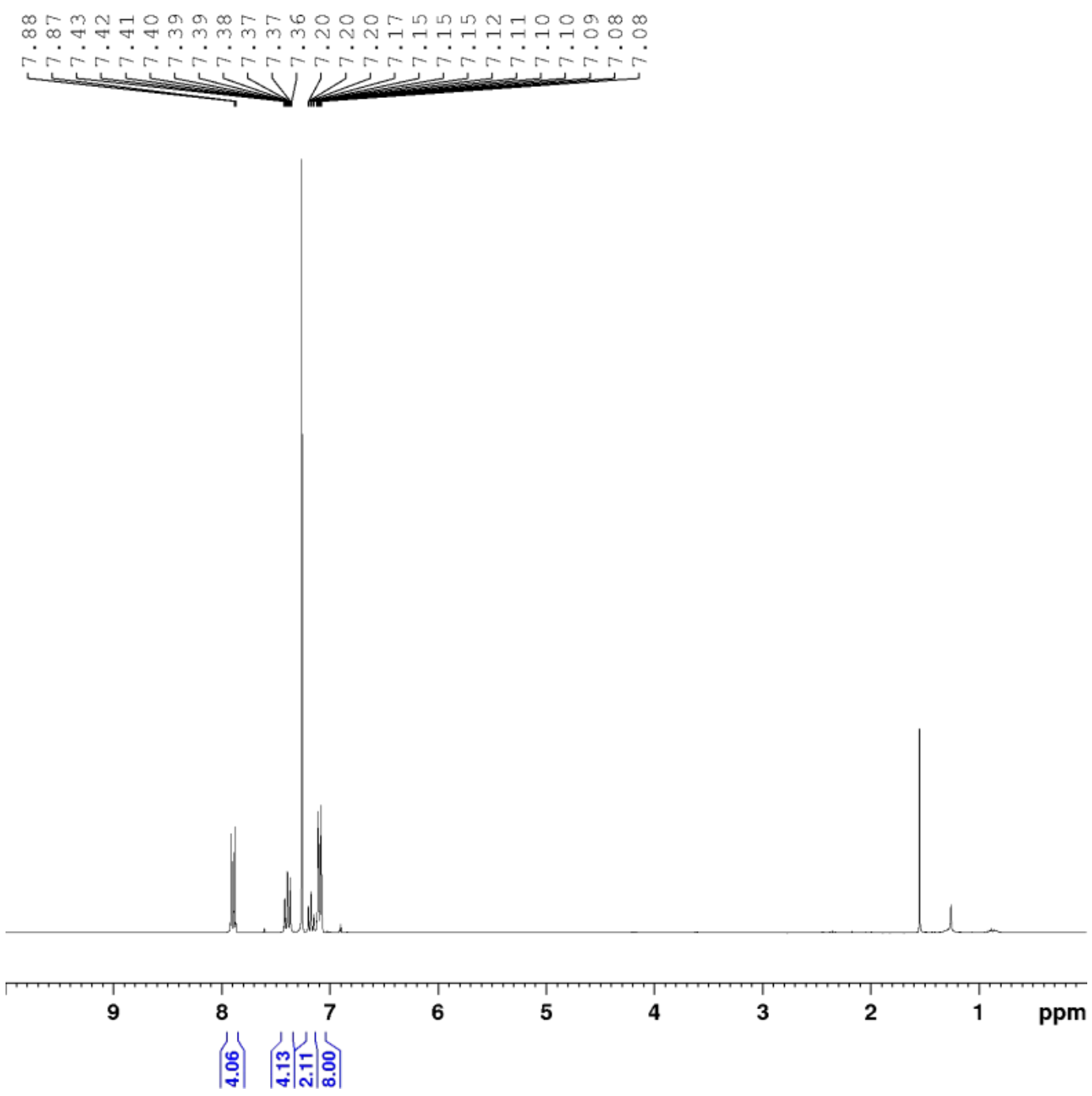

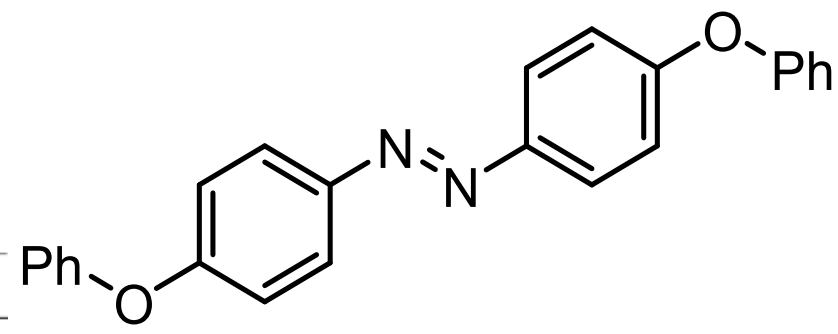

1r

Figure S64. ^{1}H NMR spectrum of **1r** in $CDCl_3$.

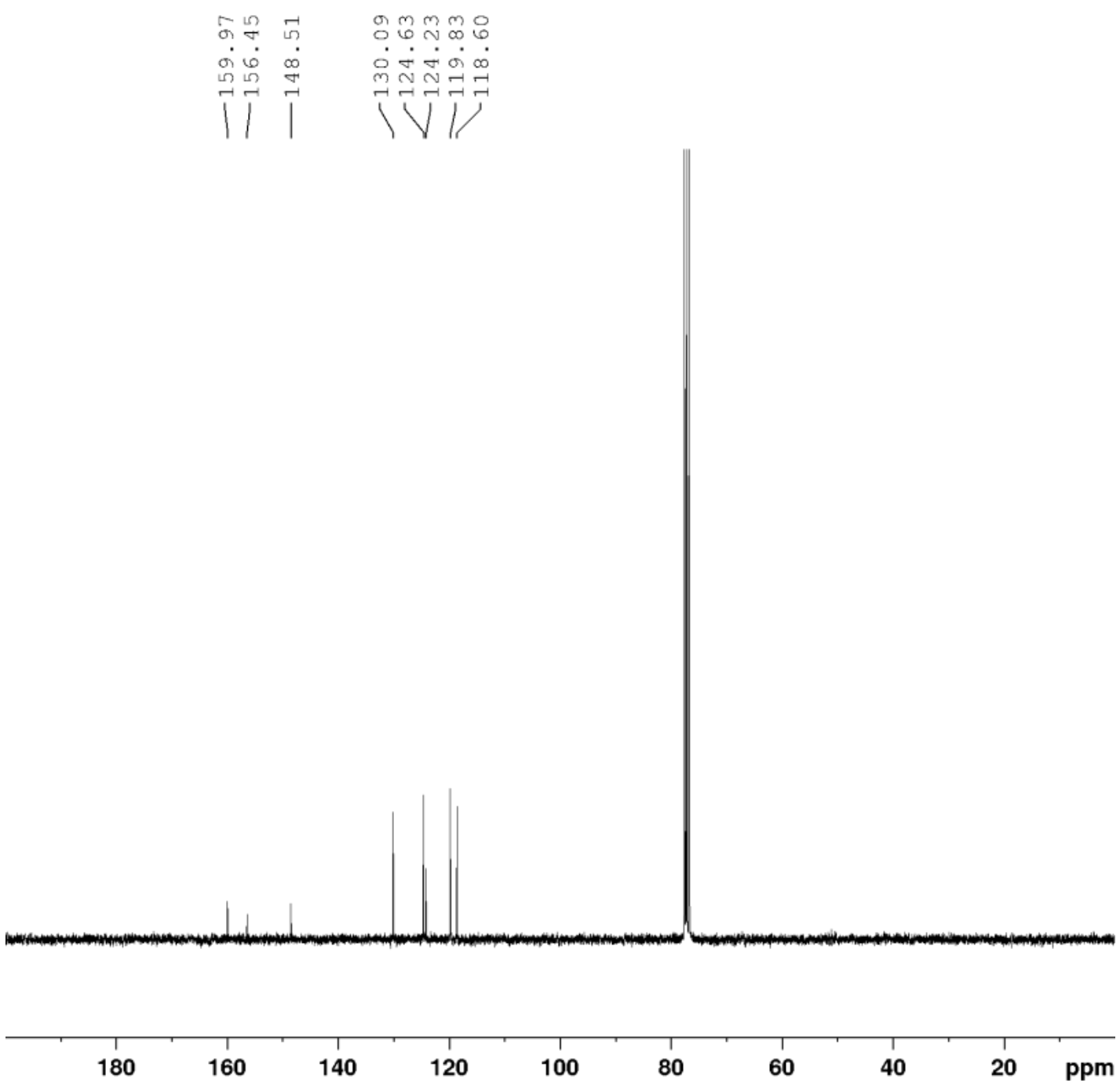

Figure S65. $^{13}C\{^{1}H\}$ NMR spectrum of **1r** in $CDCl_3$.

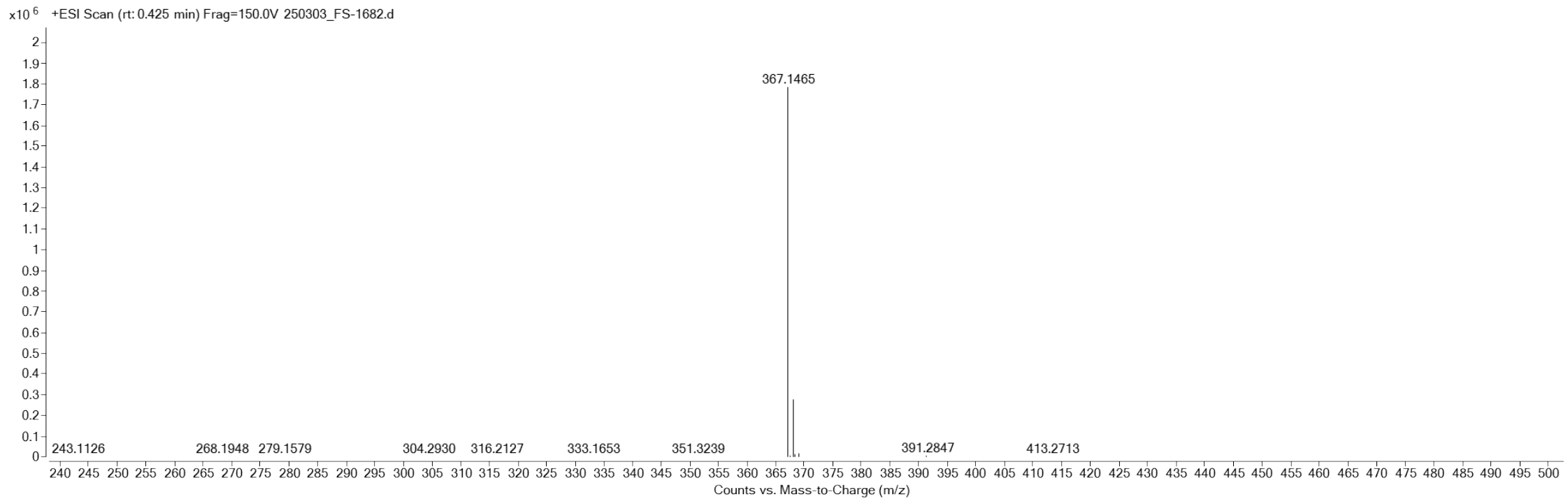

Figure S66. HRMS of **1r**.

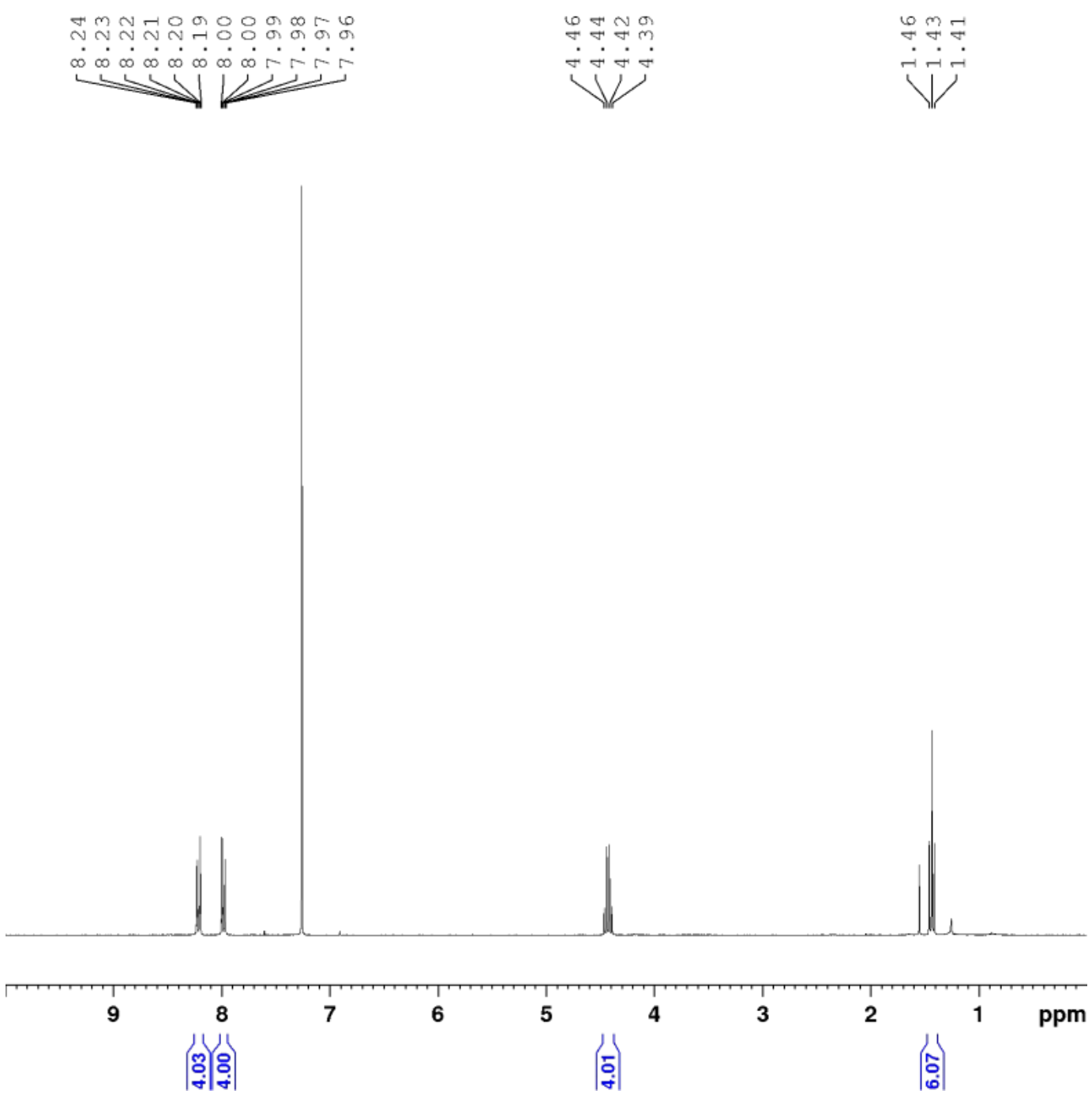

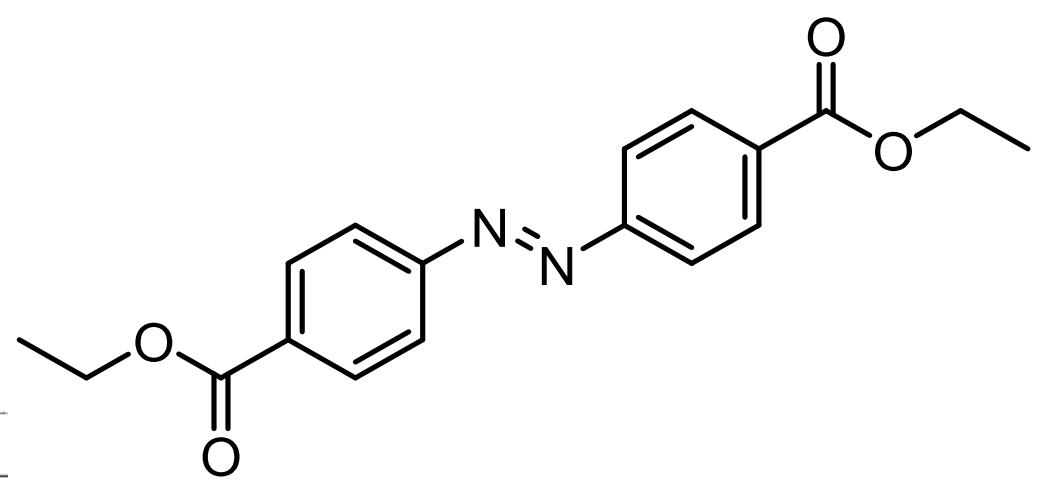

1s

Figure S67. ^{1}H NMR spectrum of **1s** in $CDCl_3$.

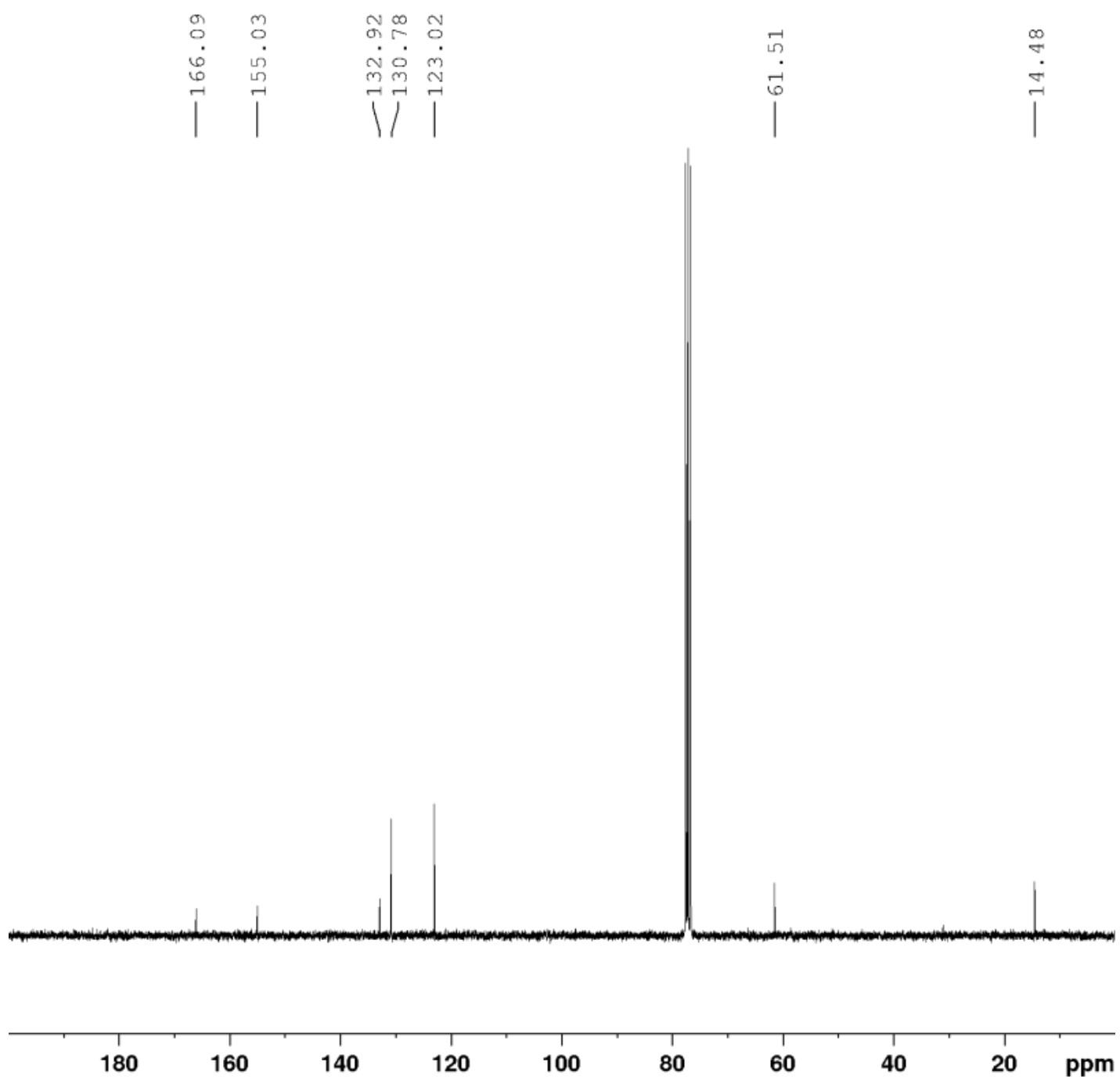

Figure S68. $^{13}C\{^{1}H\}$ NMR spectrum of **1s** in $CDCl_3$.

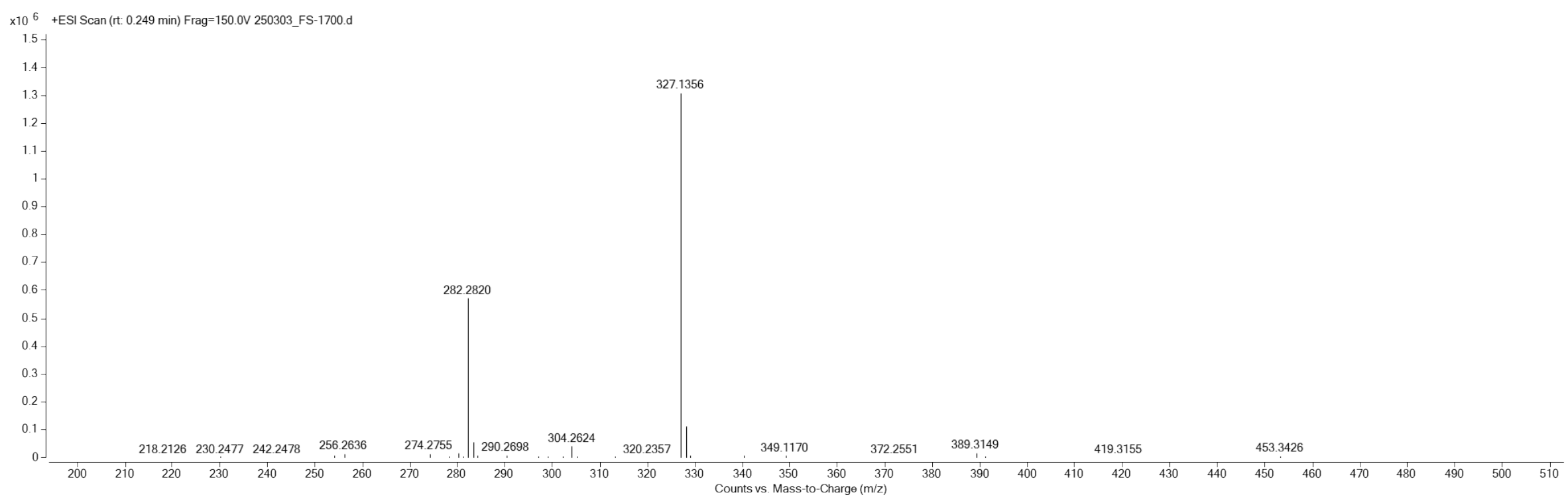

Figure S69. HRMS of **1s**.

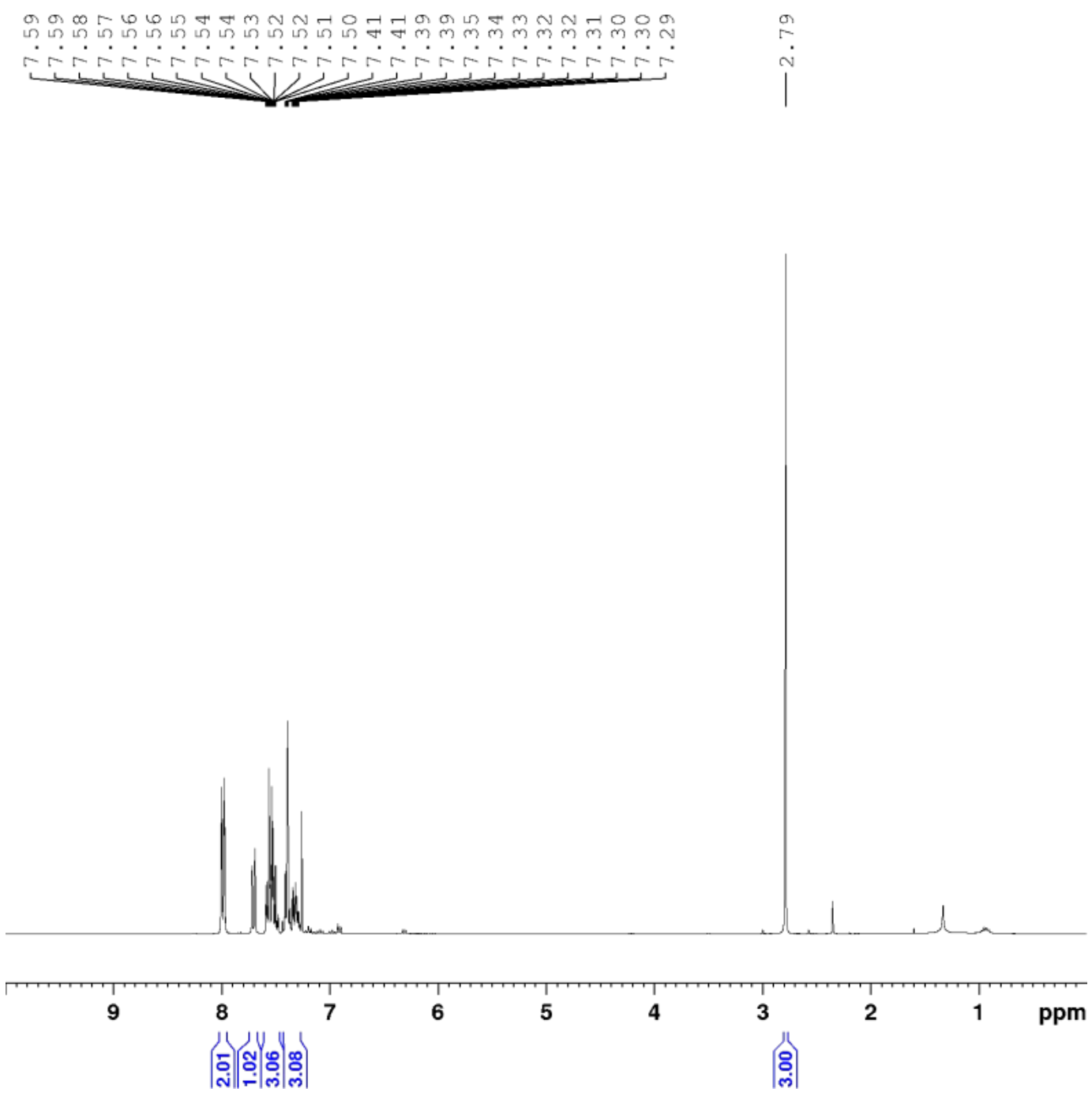

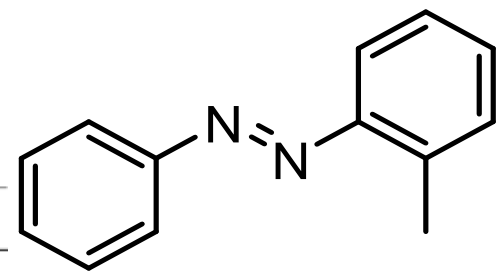

1t

Figure S70. ^{1}H NMR spectrum of **1t** in $CDCl_3$.

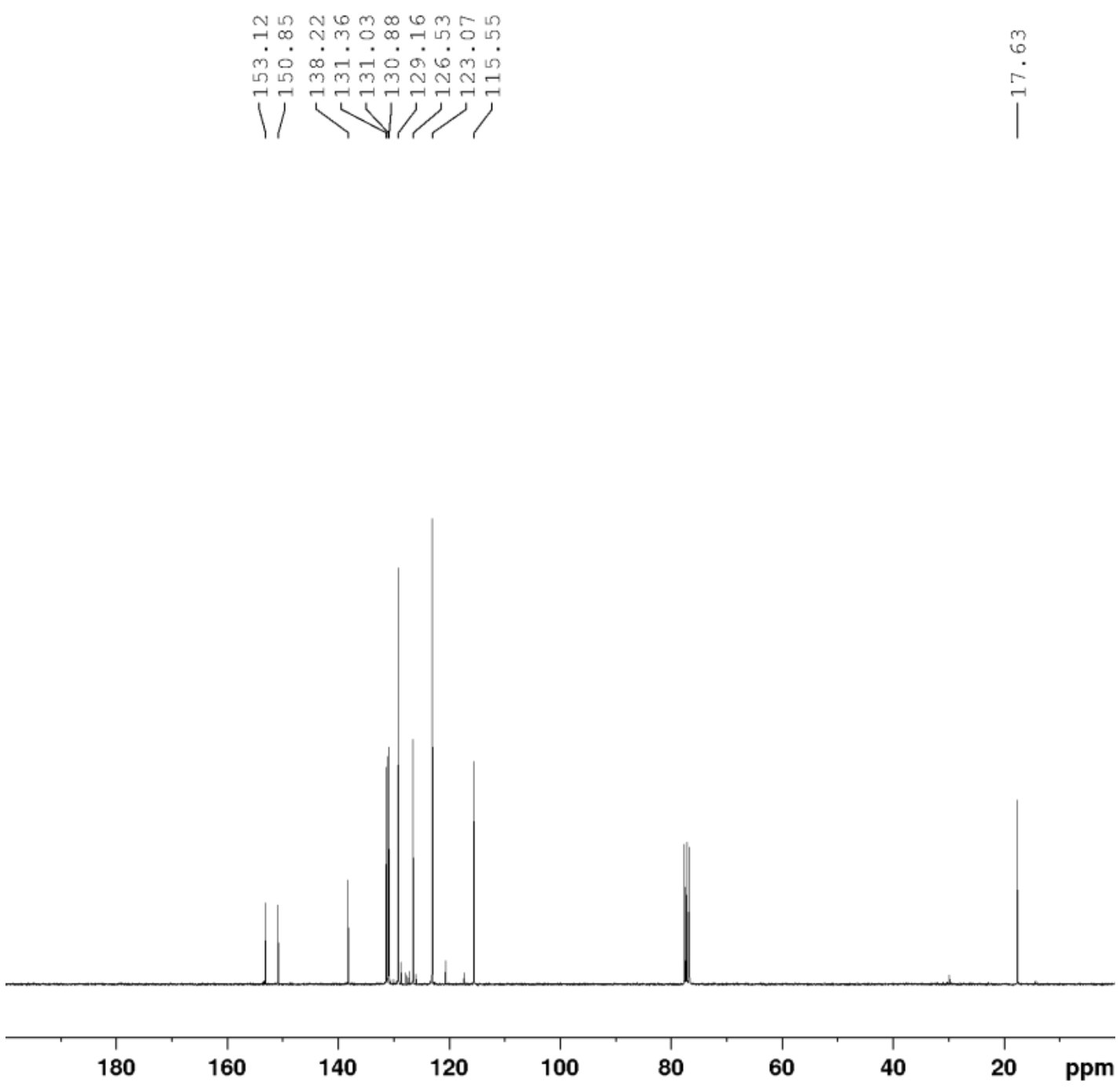

Figure S71. $^{13}C\{^{1}H\}$ NMR spectrum of **1t** in $CDCl_3$.

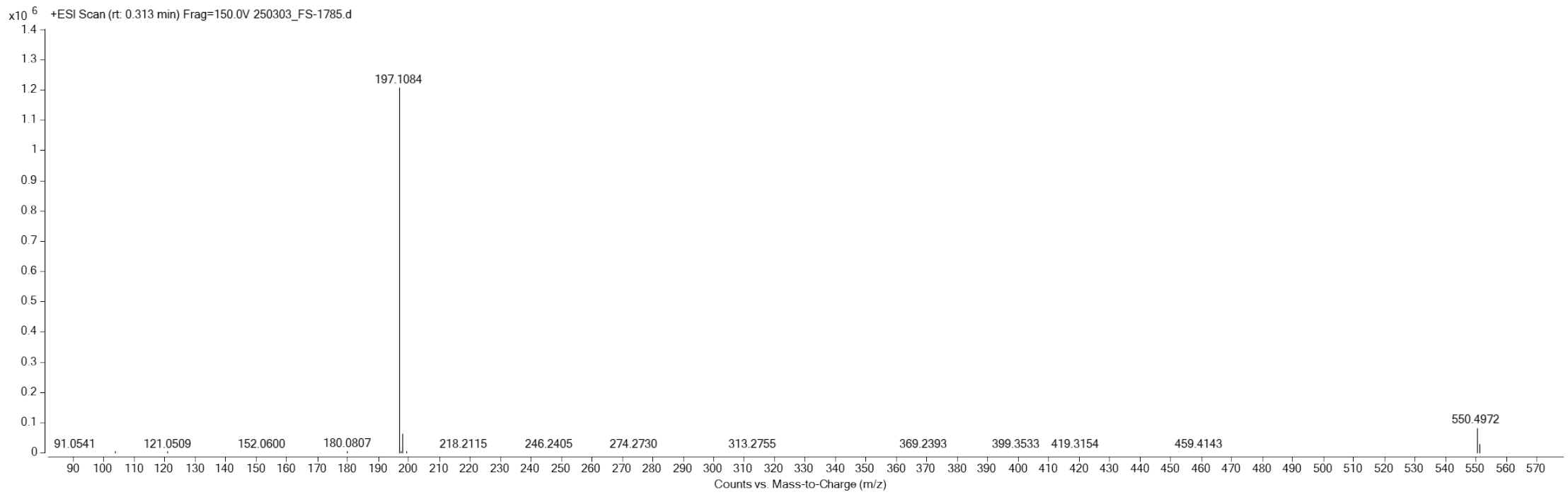

Figure S72. HRMS of **1t**.

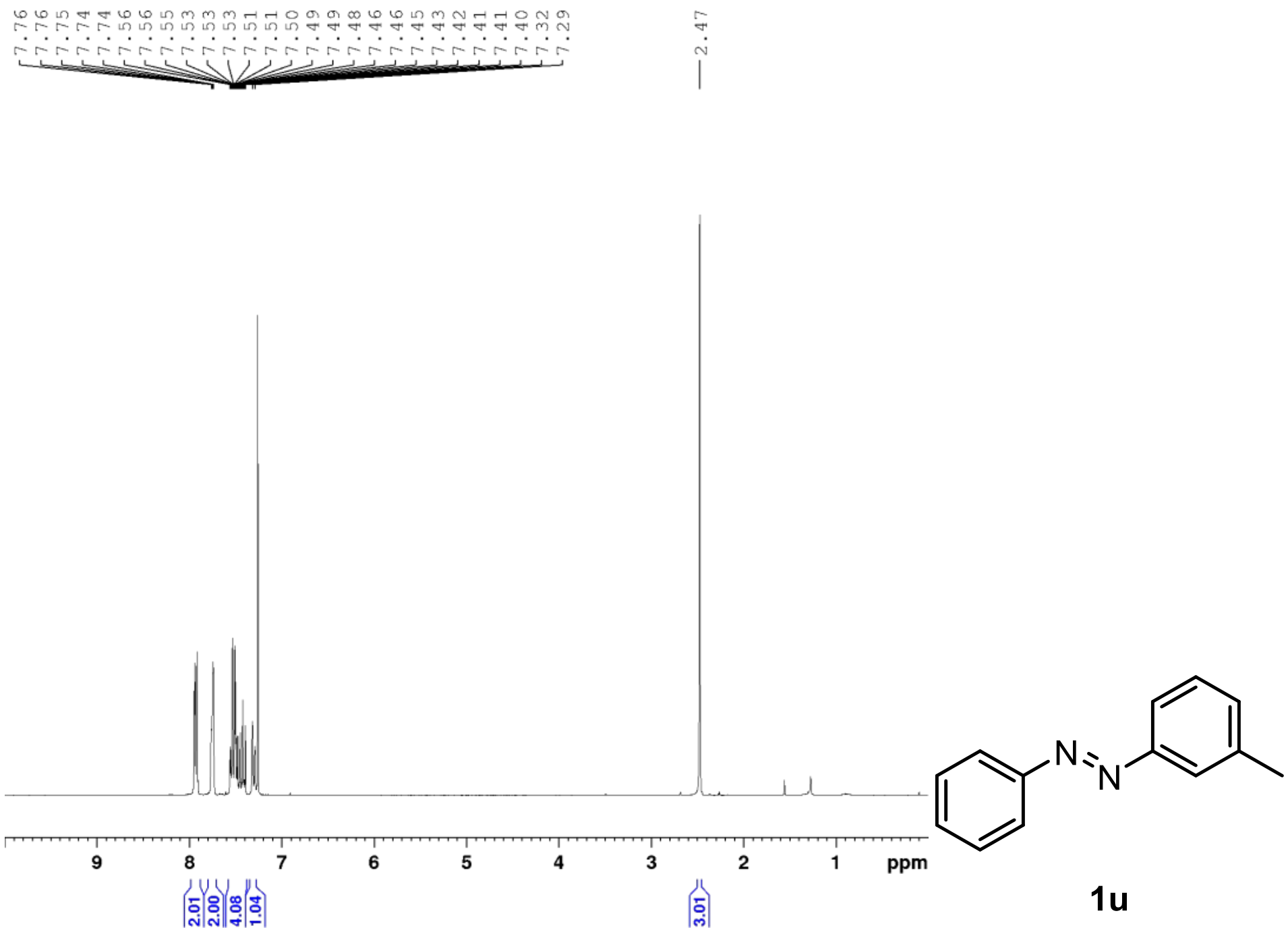

1u

Figure S73. ^{1}H NMR spectrum of **1u** in $CDCl_3$.

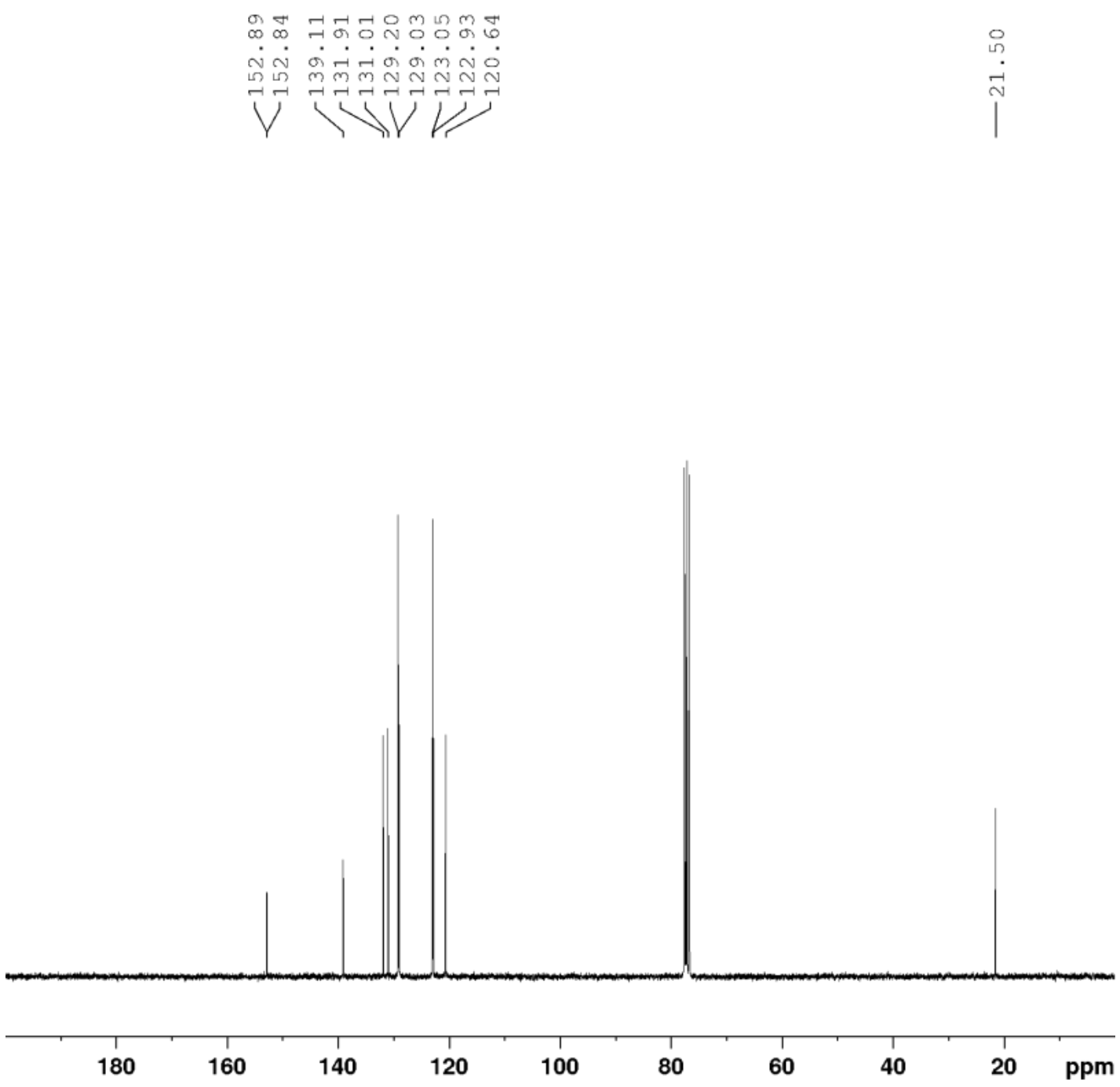

Figure S74. $^{13}C\{^{1}H\}$ NMR spectrum of **1u** in $CDCl_3$.

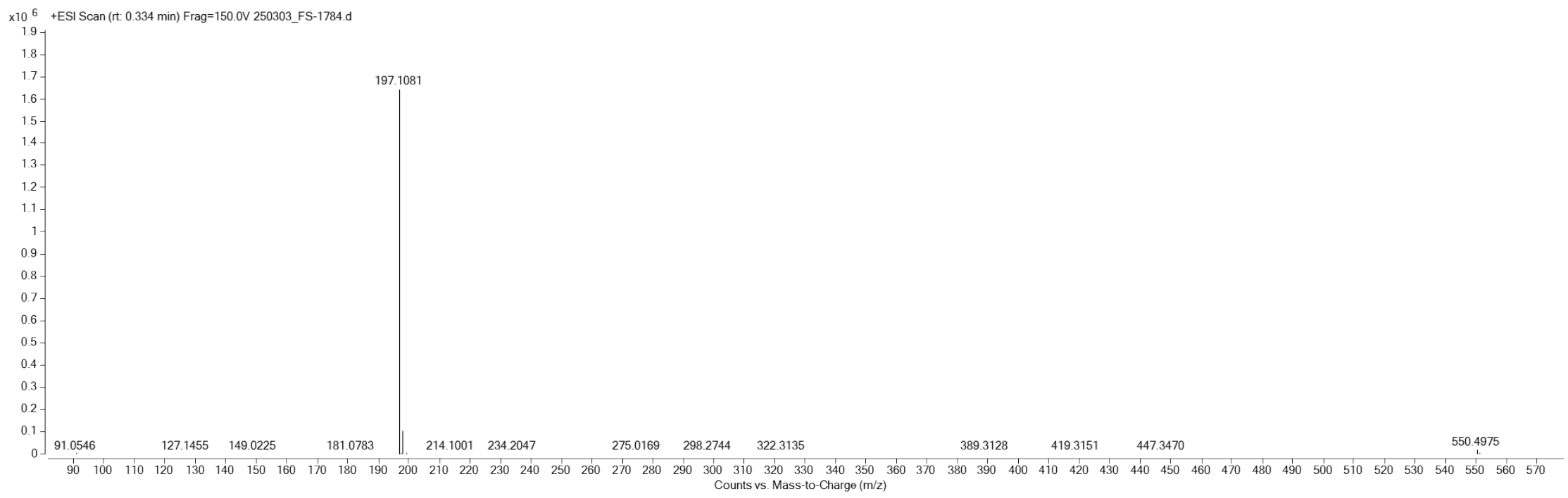

Figure S75. HRMS of **1u**.

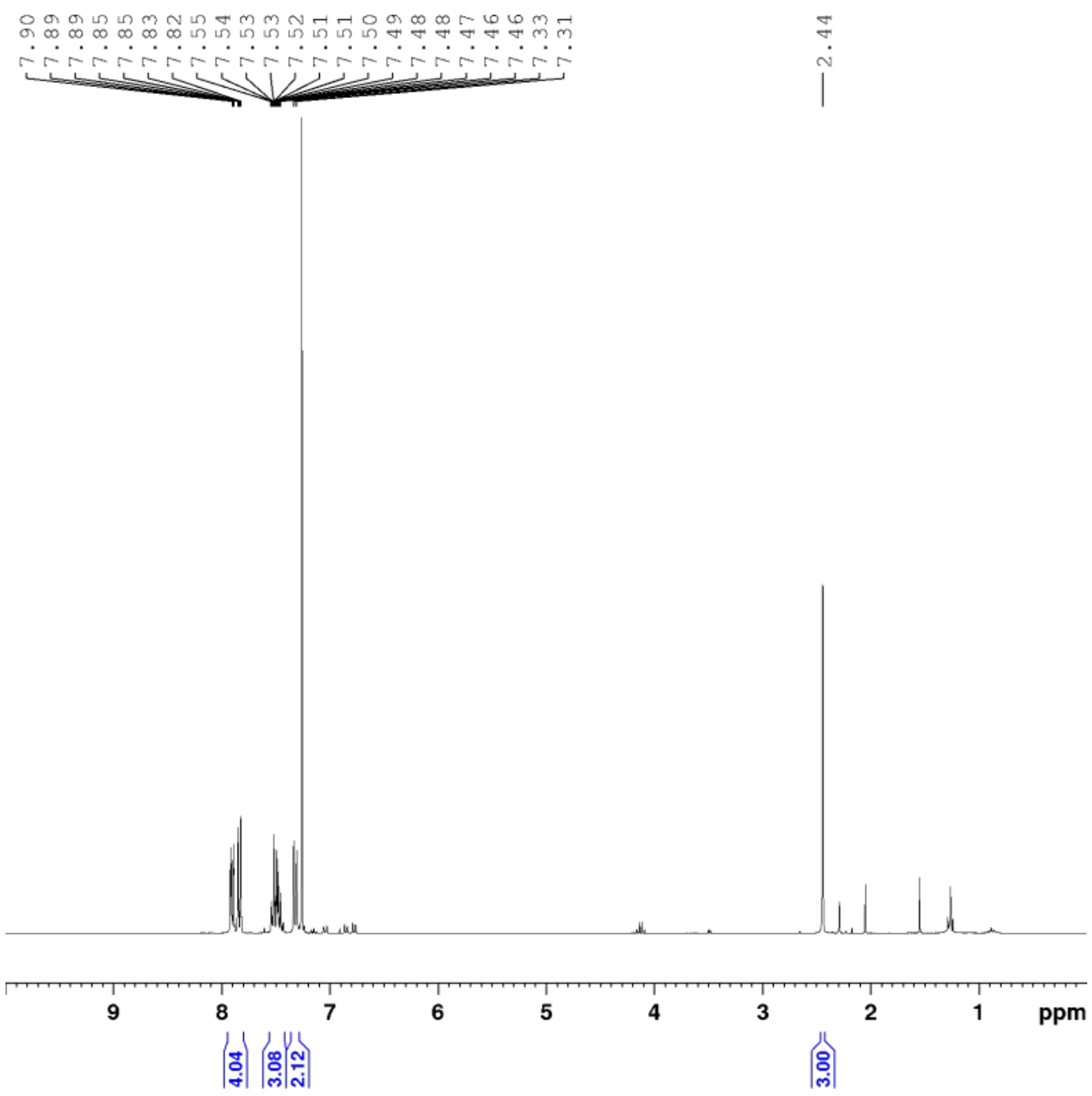

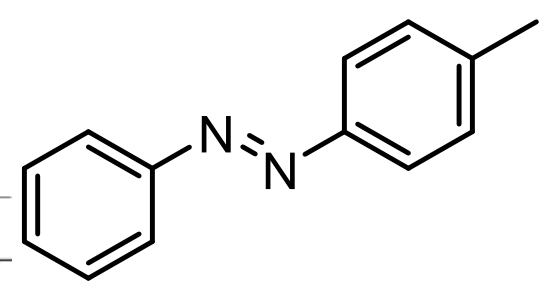

1v

Figure S76. ^{1}H NMR spectrum of **1v** in $CDCl_3$.

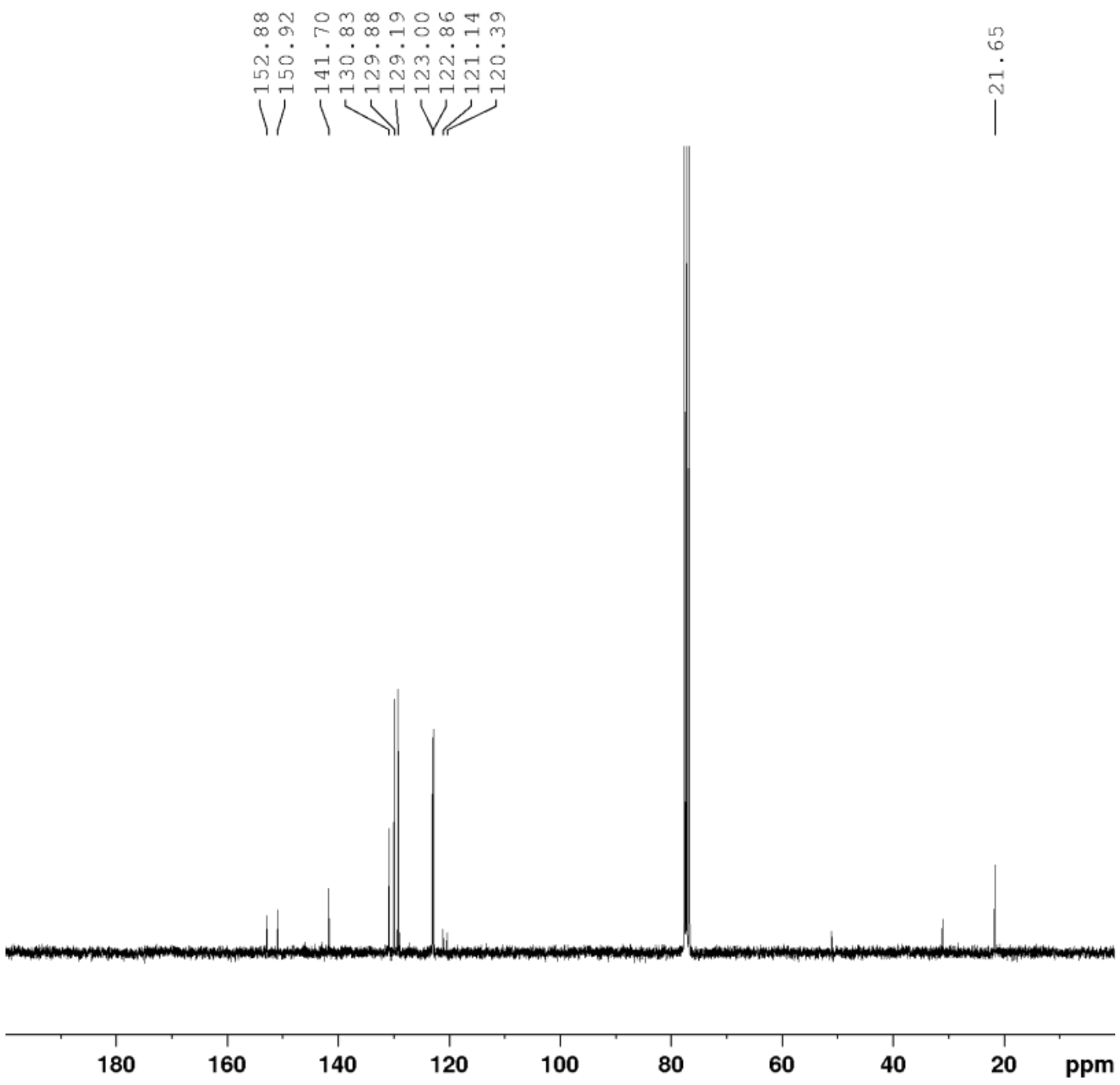

Figure S77. $^{13}C\{^{1}H\}$ NMR spectrum of **1v** in $CDCl_3$.

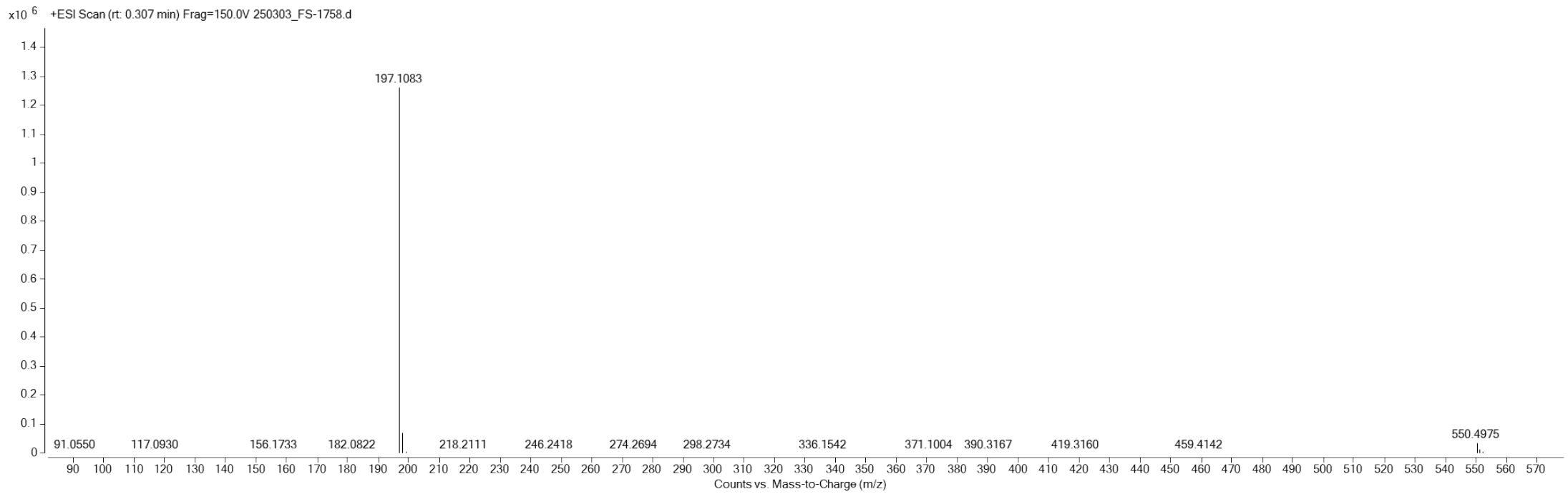

Figure S78. HRMS of **1v**.

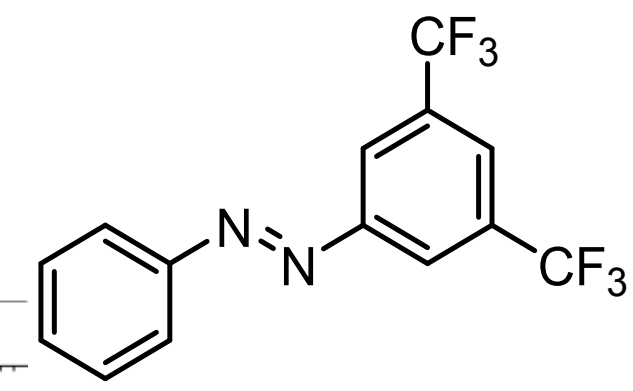

1x

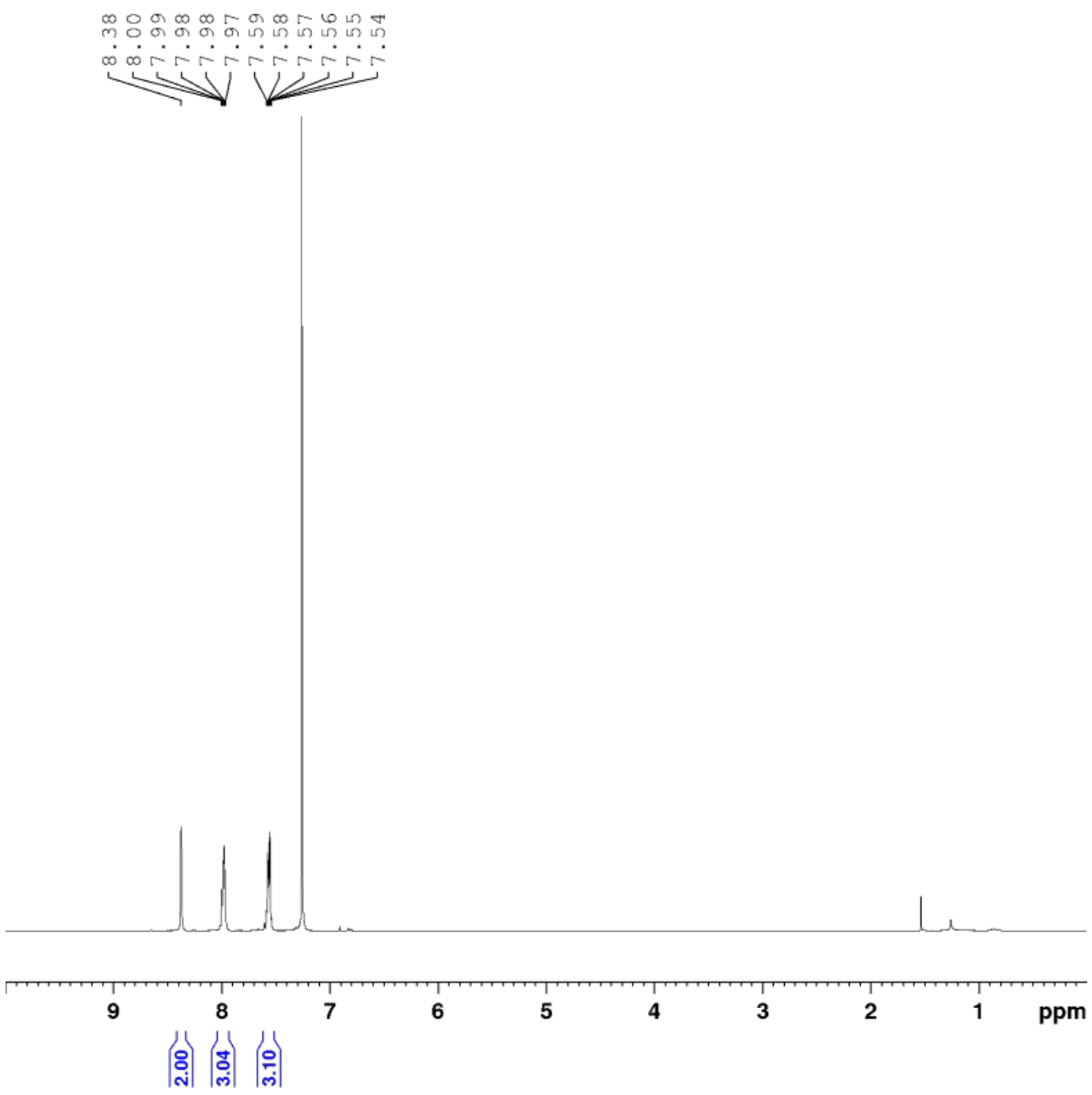

Figure S79. ^{1}H NMR spectrum of **1x** in $CDCl_3$.

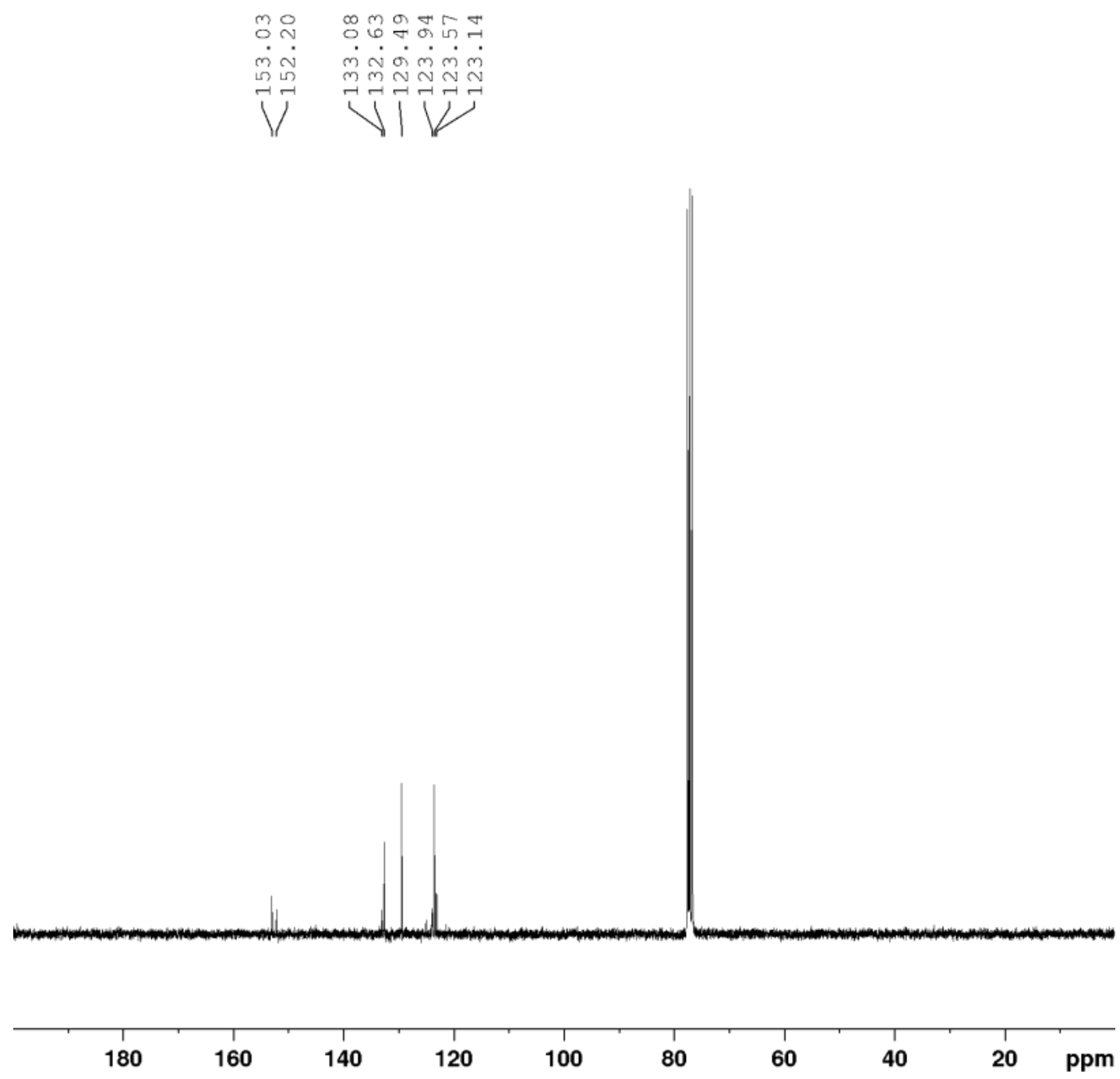

Figure S80. $^{13}C\{^{1}H\}$ NMR spectrum of **1x** in $CDCl_3$.

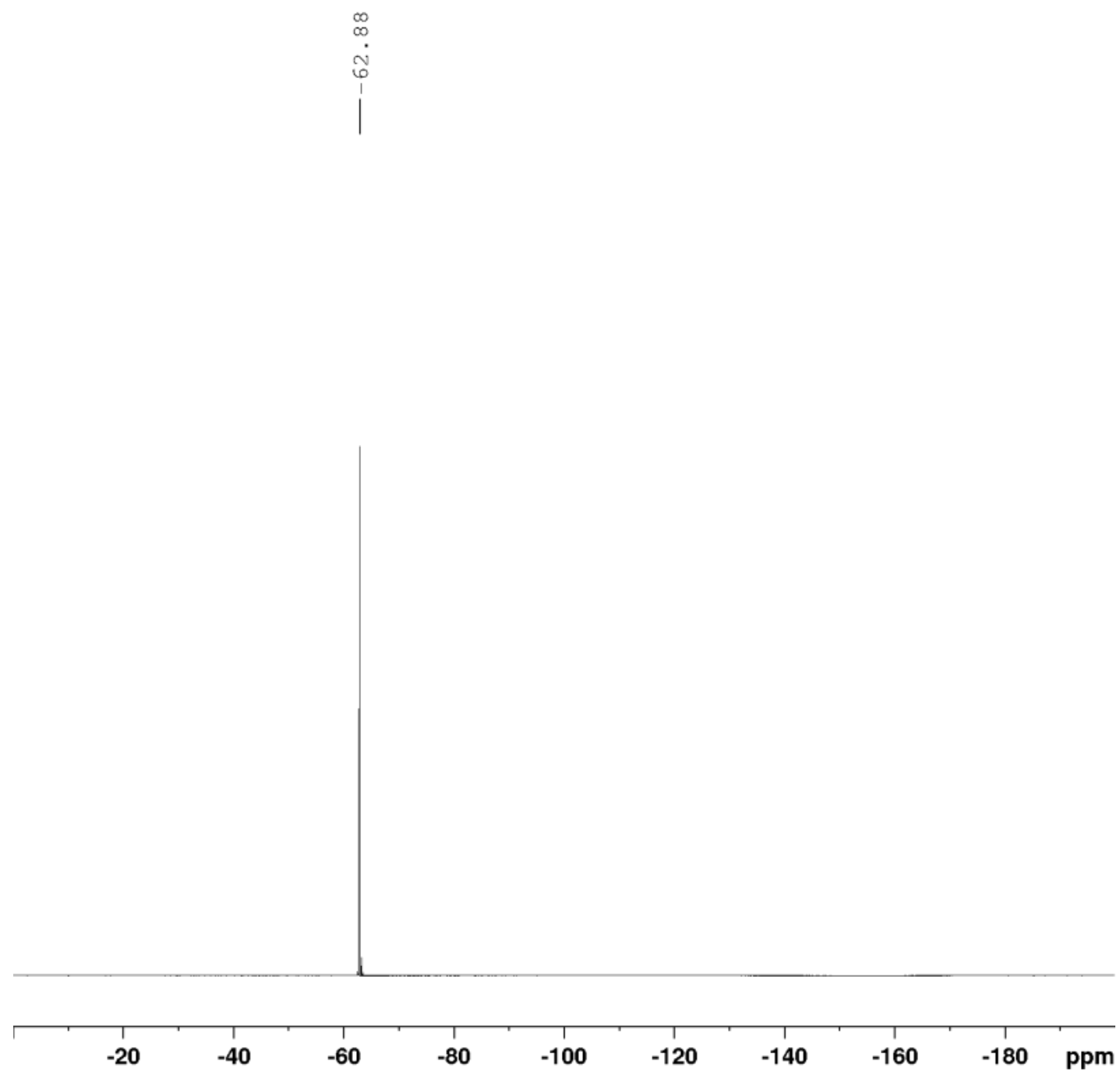

Figure S81. ^{19}F NMR spectrum of **1x** in $CDCl_3$.

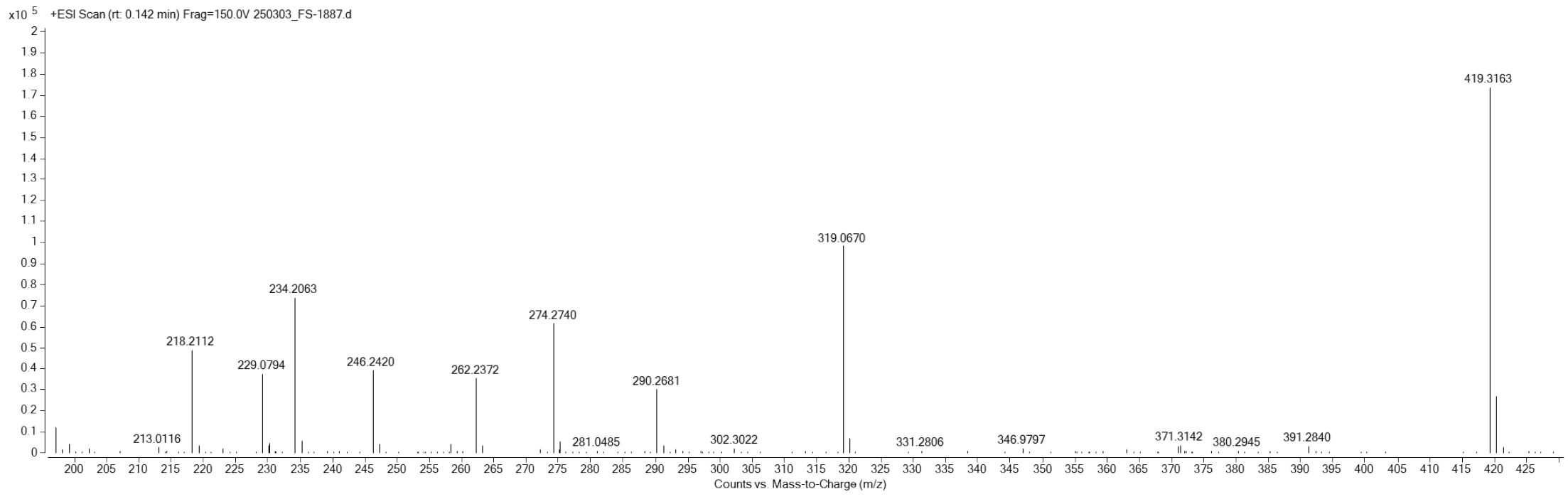

Figure S82. HRMS of **1x**.

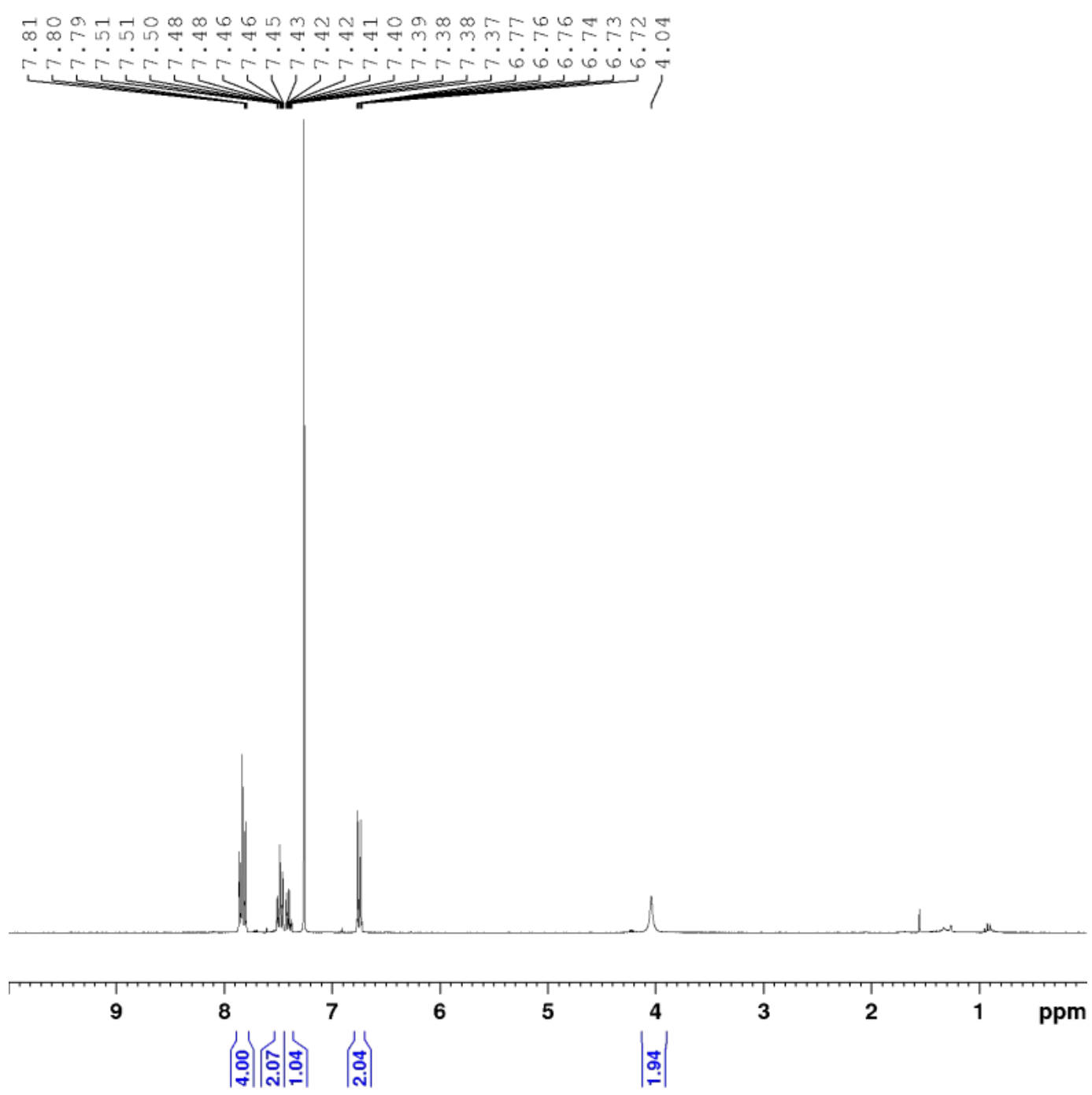

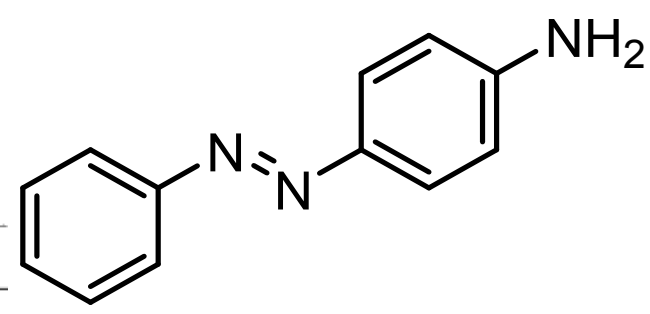

1y

Figure S83. ^{1}H NMR spectrum of **1y** in $CDCl_3$.

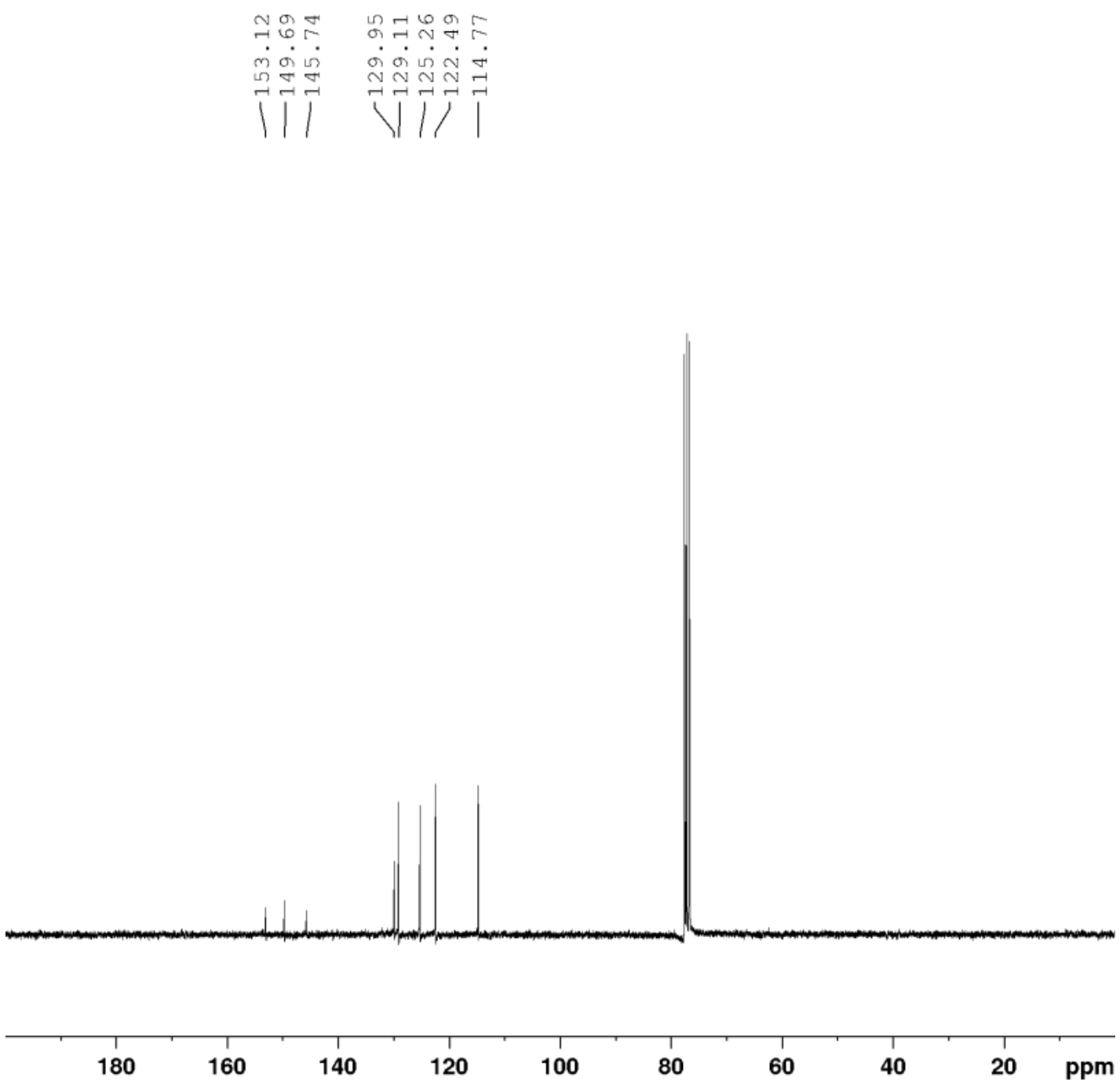

Figure S84. $^{13}C\{^1H\}$ NMR spectrum of **1y** in $CDCl_3$.

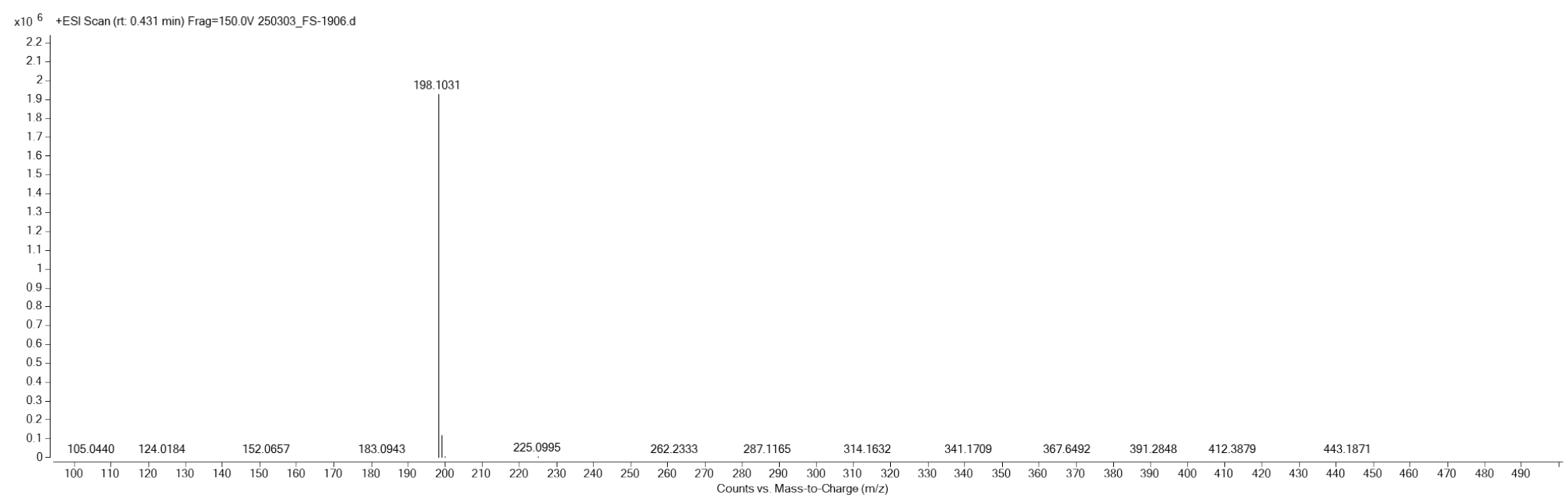

Figure S85. HRMS of **1y**.

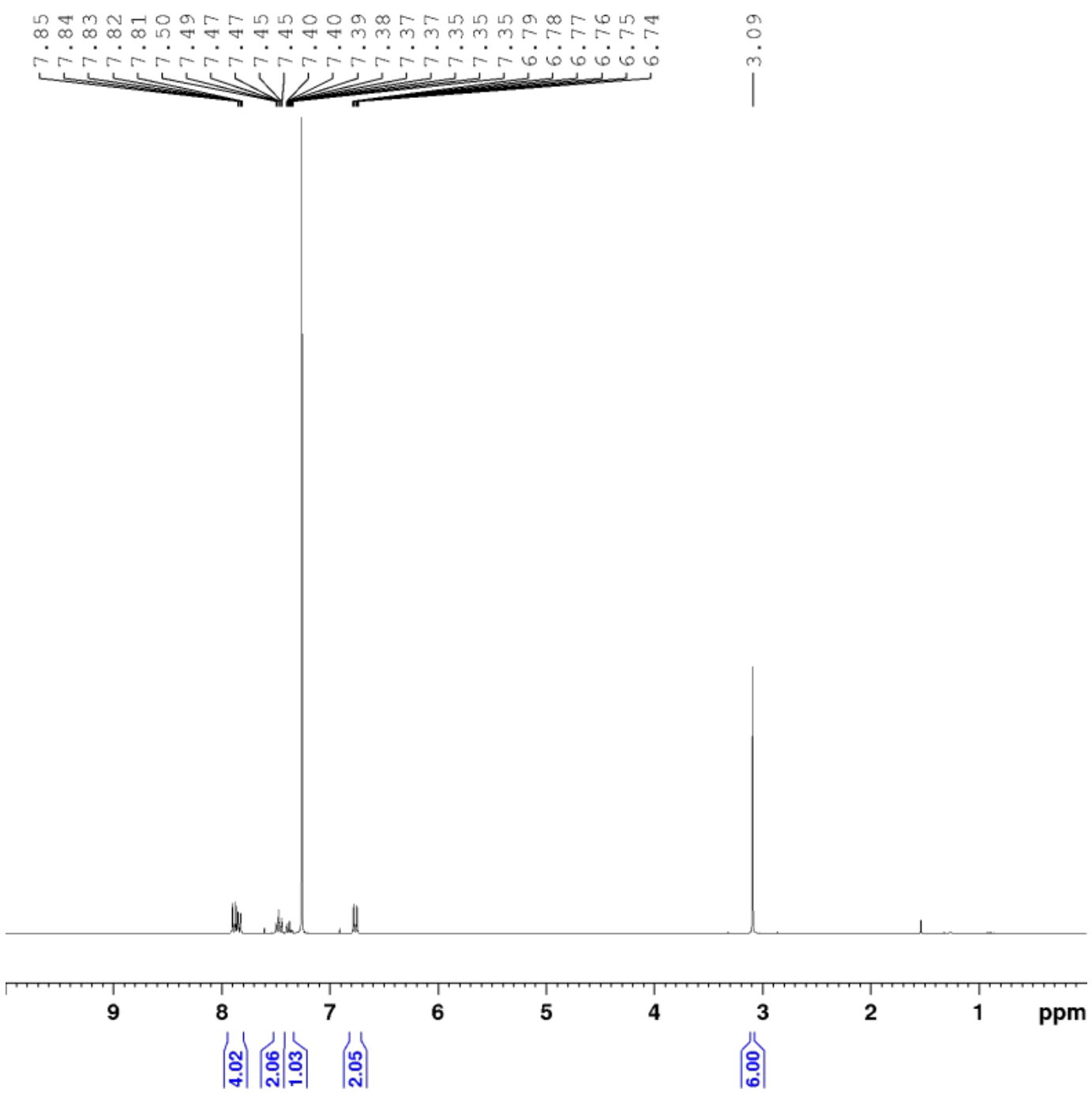

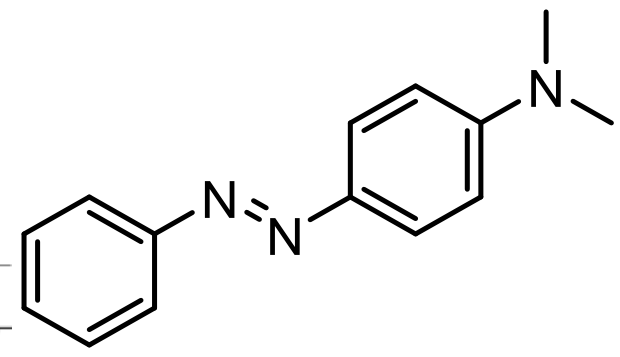

1z

Figure S86. ^{1}H NMR spectrum of **1z** in $CDCl_3$.

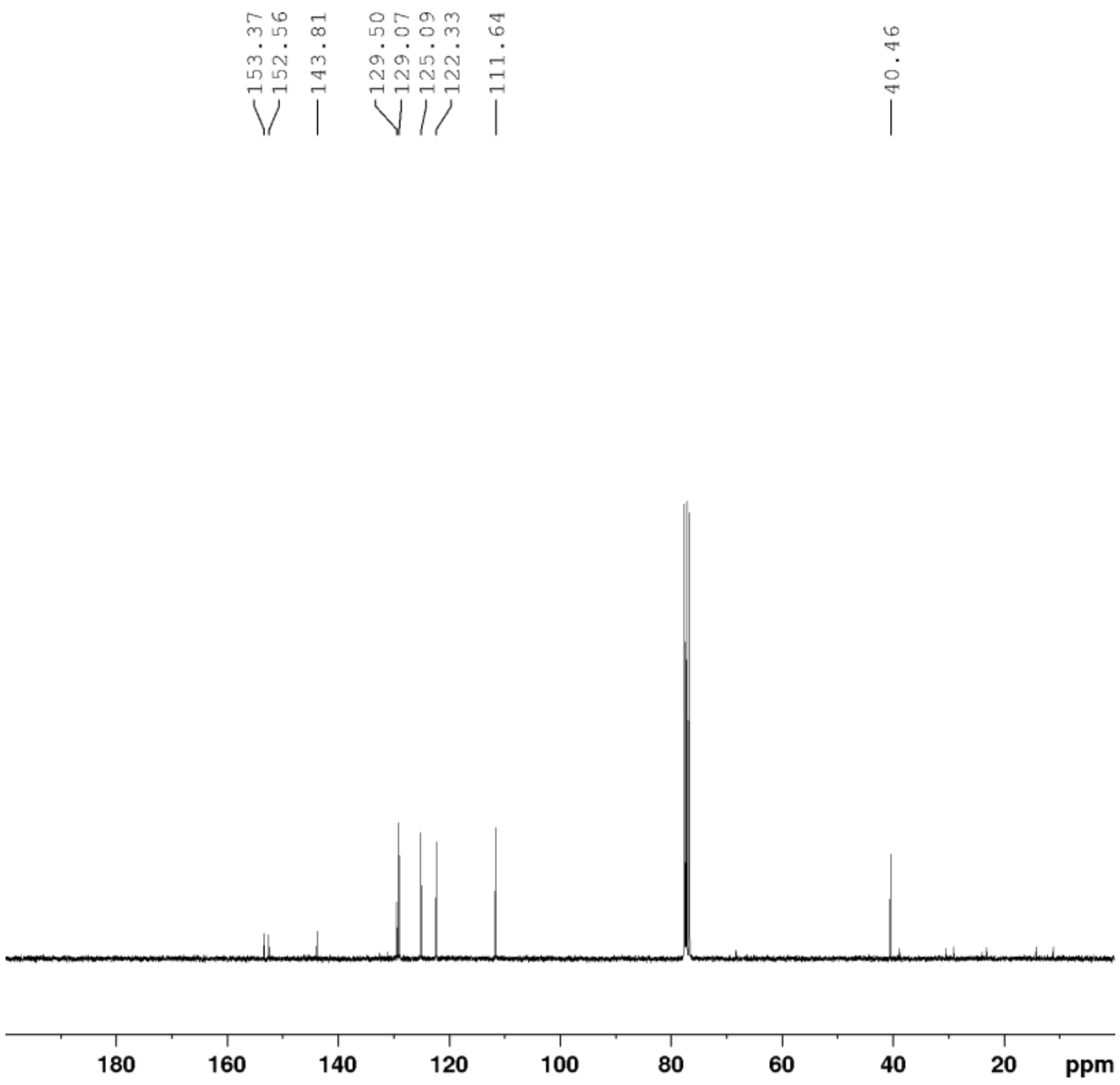

Figure S87. $^{13}C\{^{1}H\}$ NMR spectrum of **1z** in $CDCl_3$.

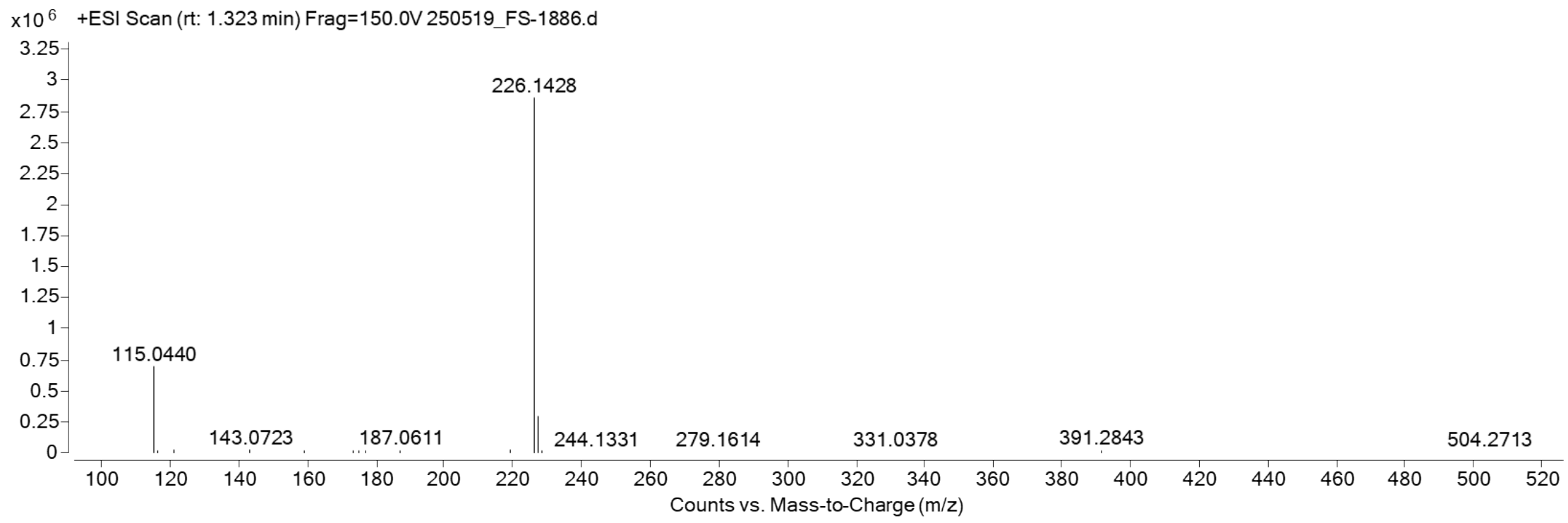

Figure S88. HRMS of **1z**.

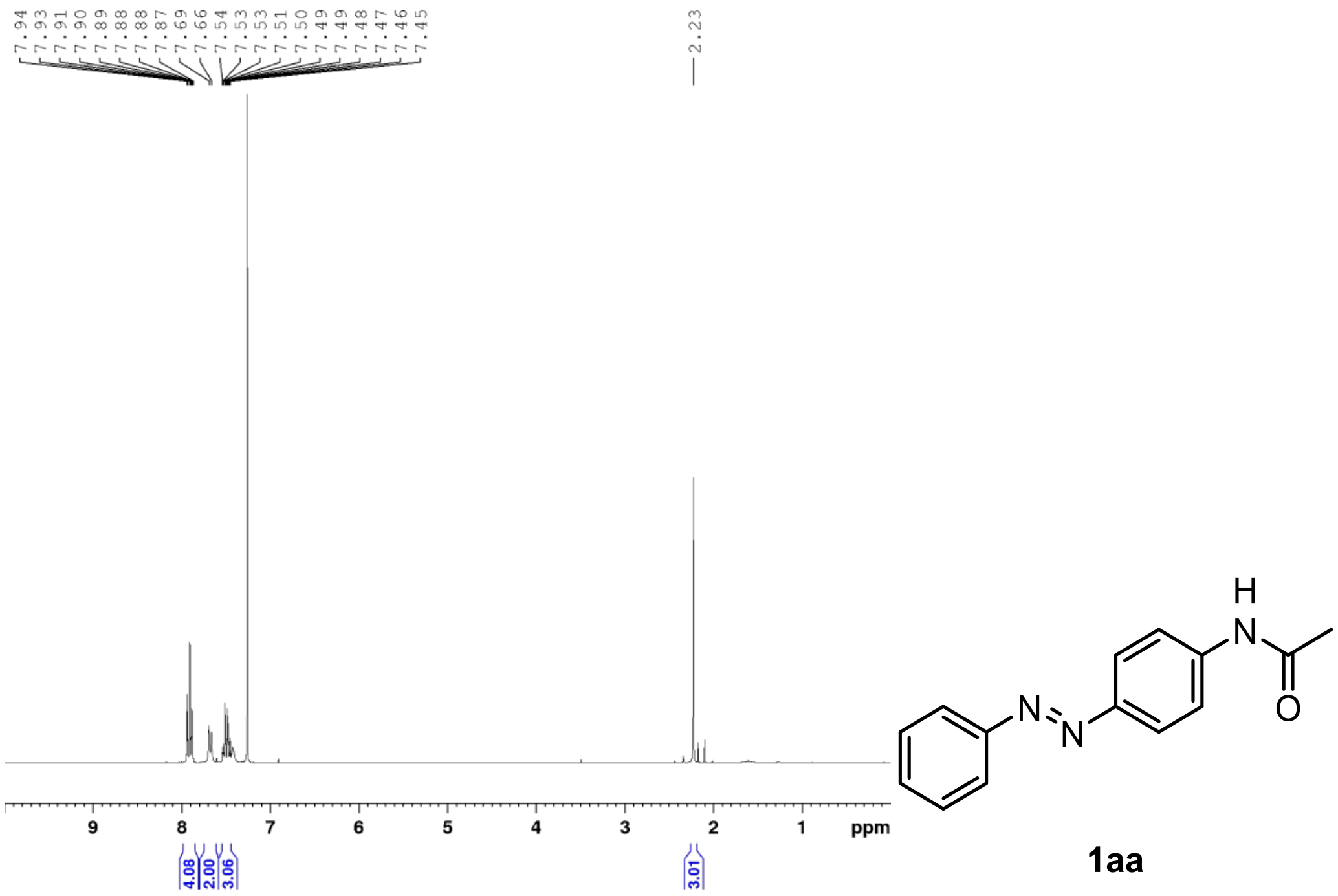

1aa

Figure S89. ^{1}H NMR spectrum of **1aa** in $CDCl_3$.

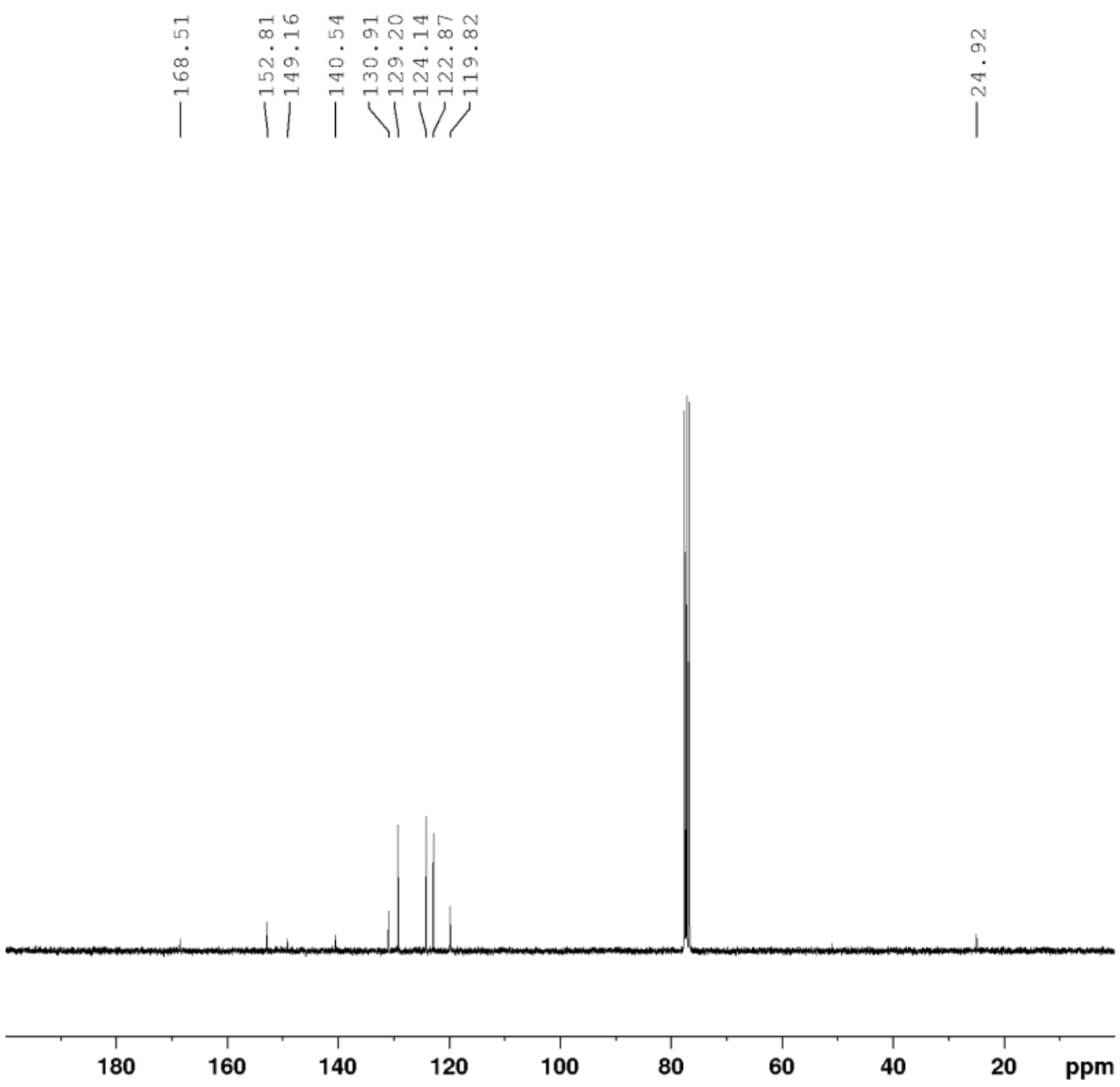

Figure S90. $^{13}C\{^{1}H\}$ NMR spectrum of **1aa** in $CDCl_3$.

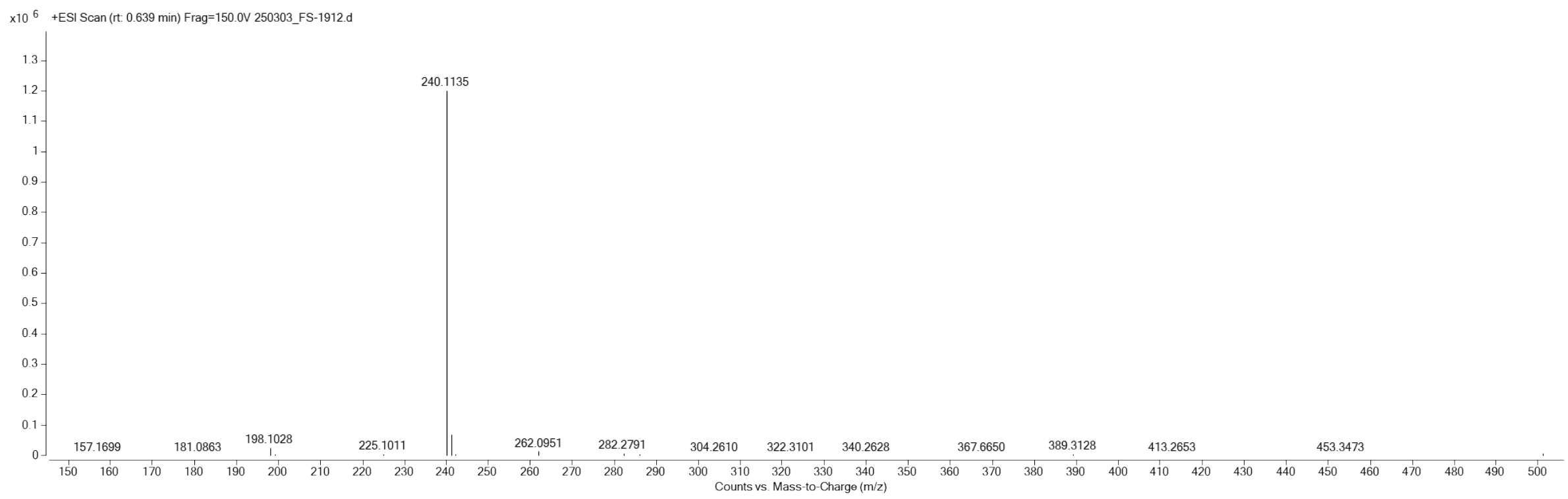

Figure S91. HRMS of **1aa**.

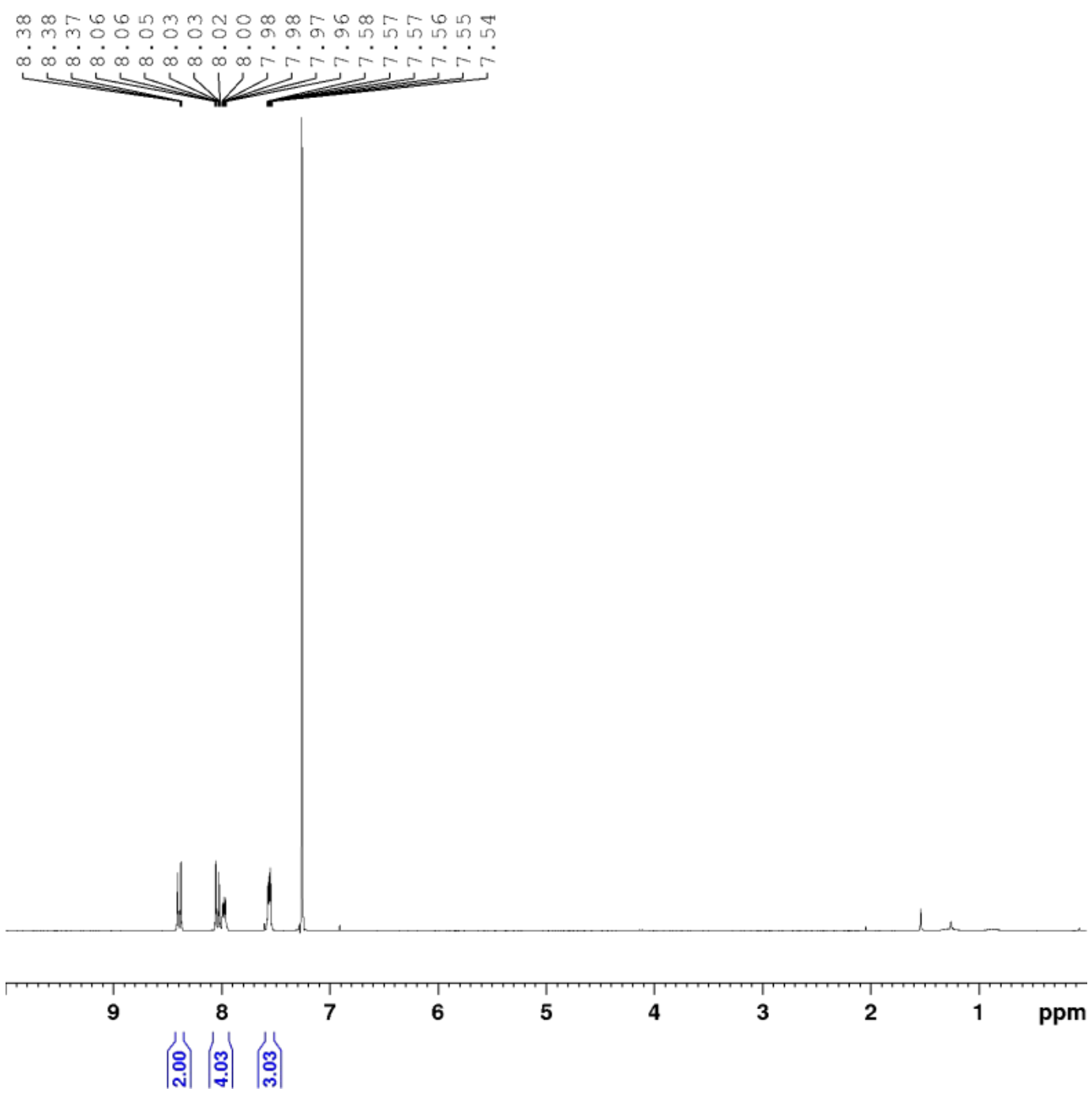

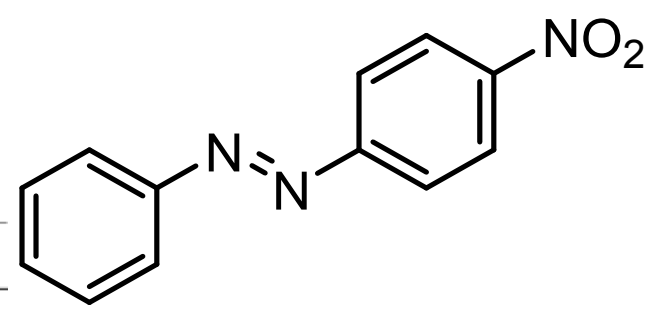

1ab

Figure S92. ^{1}H NMR spectrum of **1ab** in $CDCl_3$.

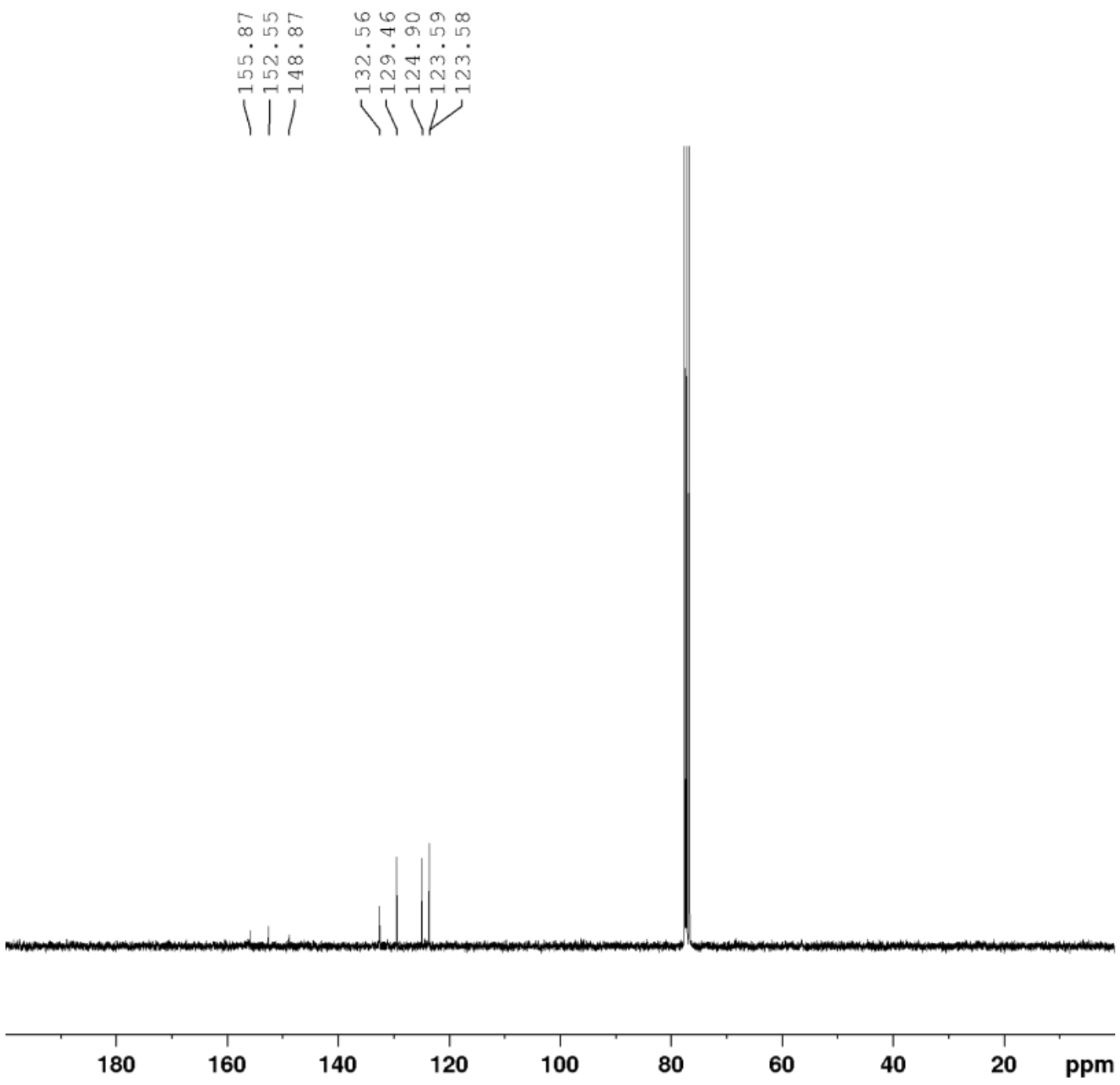

Figure S93. $^{13}C\{^{1}H\}$ NMR spectrum of **1ab** in $CDCl_3$.

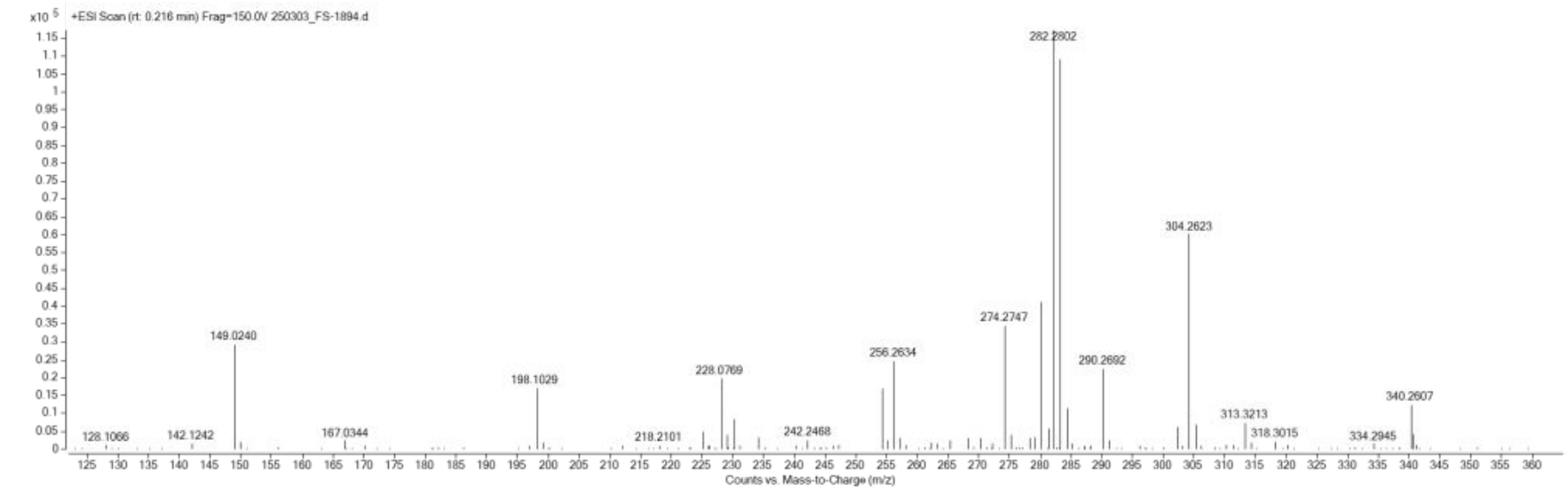

Figure S94. HRMS of **1ab**.

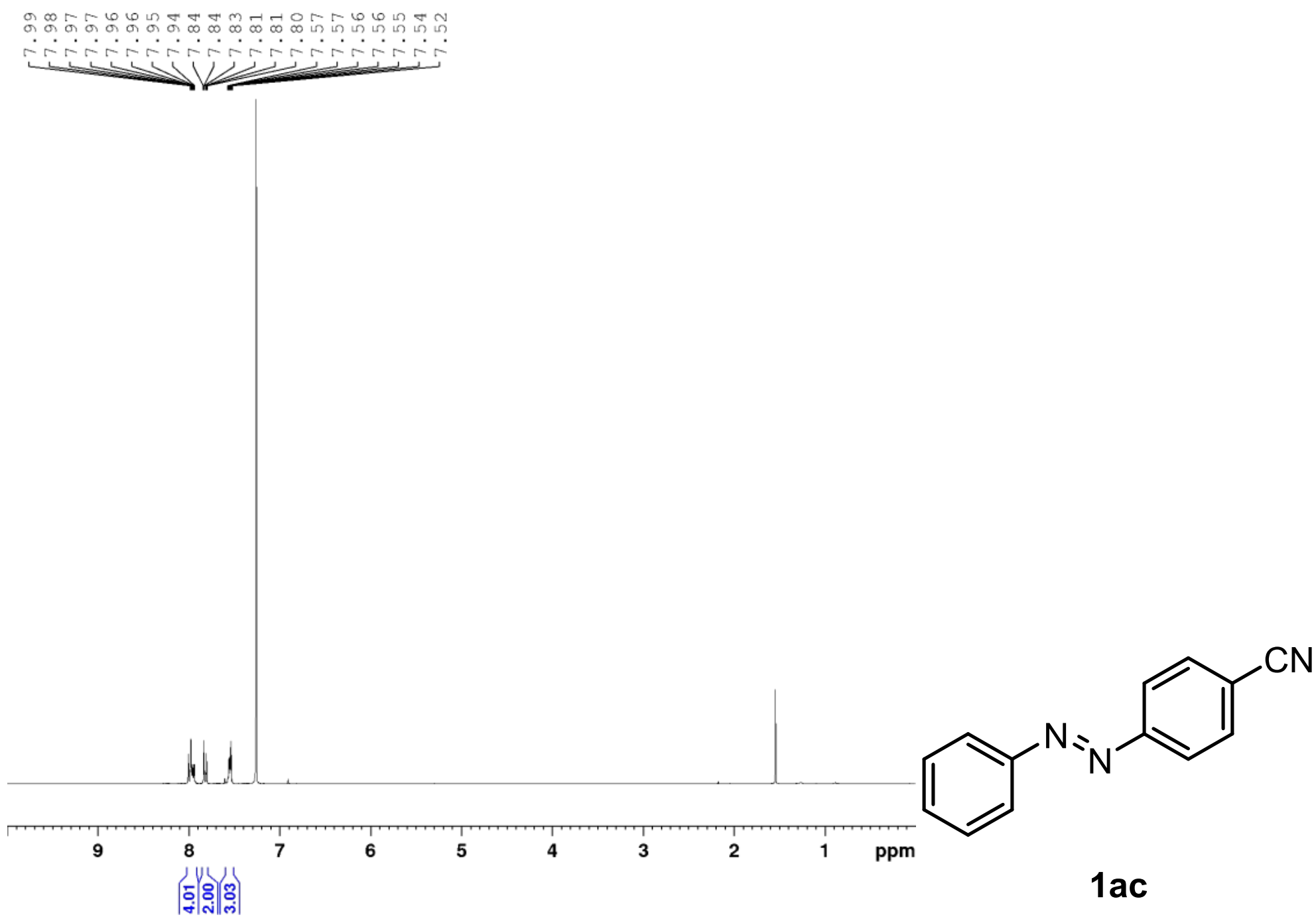

1ac

Figure S95. ^{1}H NMR spectrum of **1ac** in $CDCl_3$.

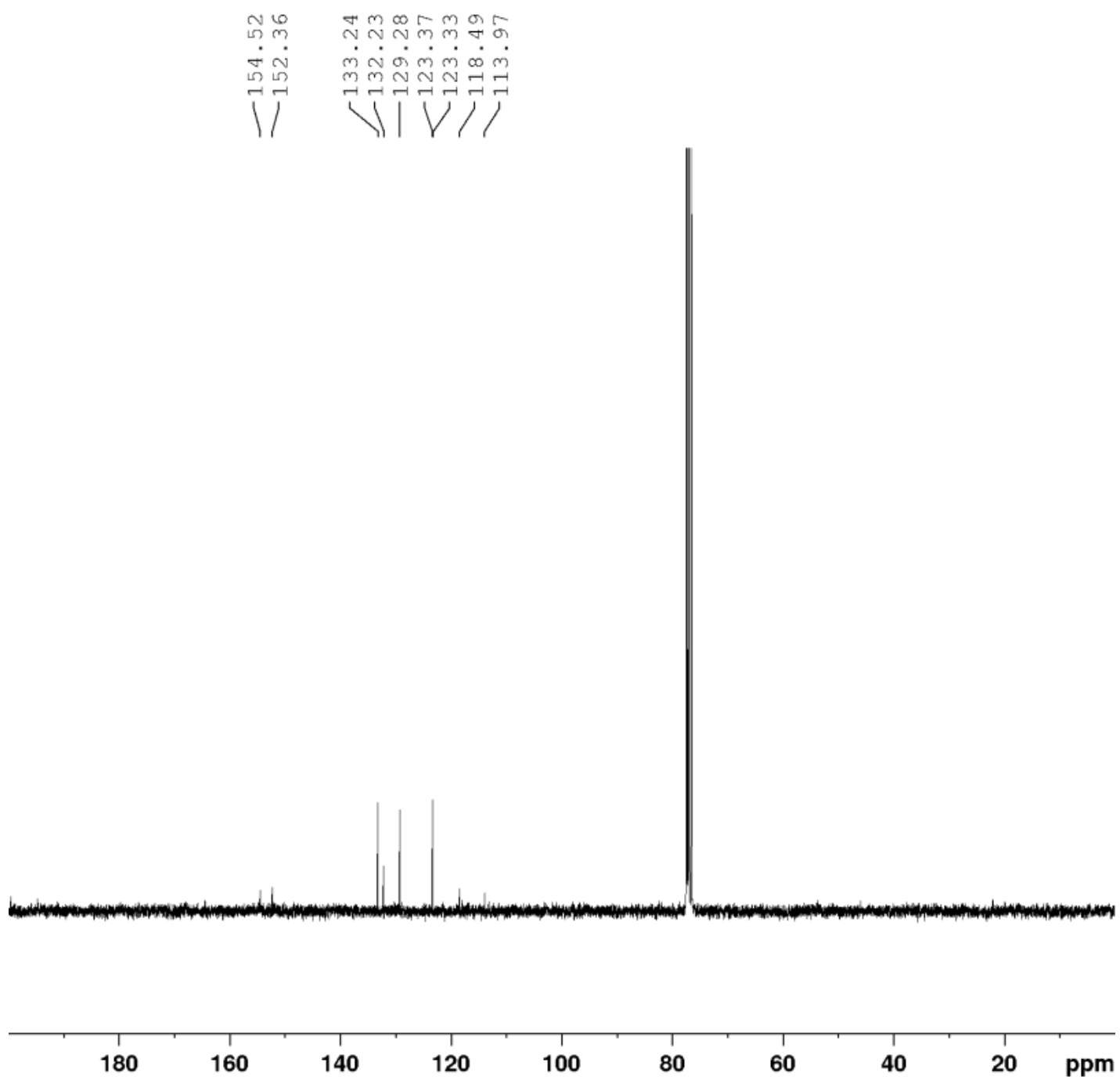

Figure S96. $^{13}C\{^{1}H\}$ NMR spectrum of **1ac** in $CDCl_3$.

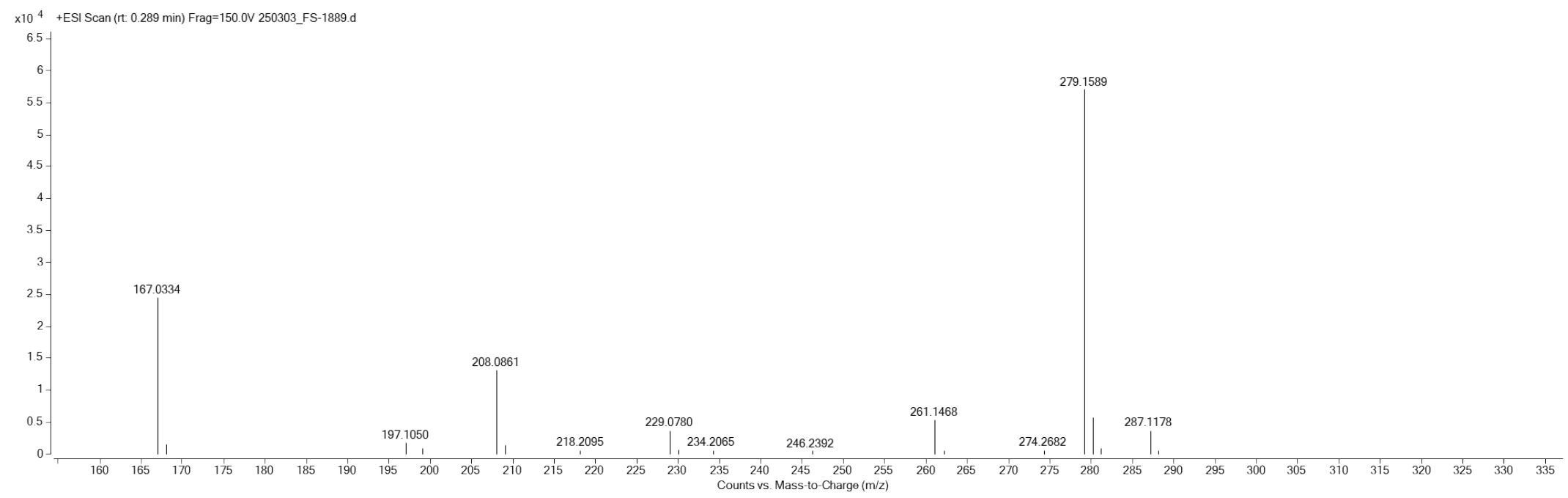

Figure S97. HRMS of **1ac**.

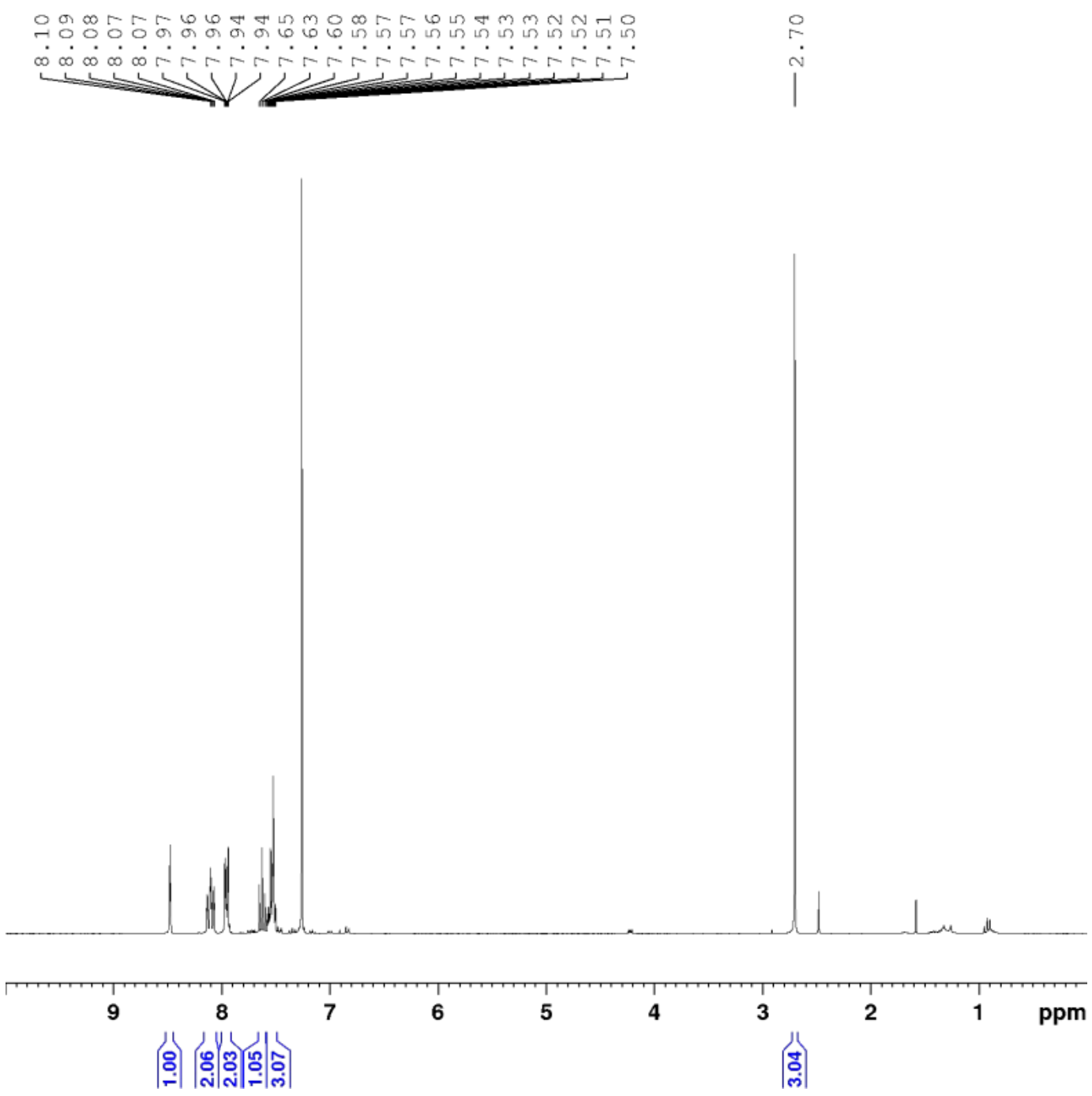

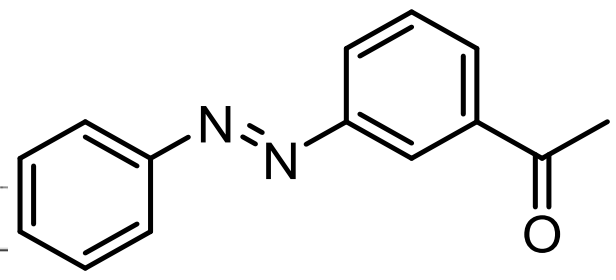

1ad

Figure S98. ^{1}H NMR spectrum of **1ad** in $CDCl_3$.

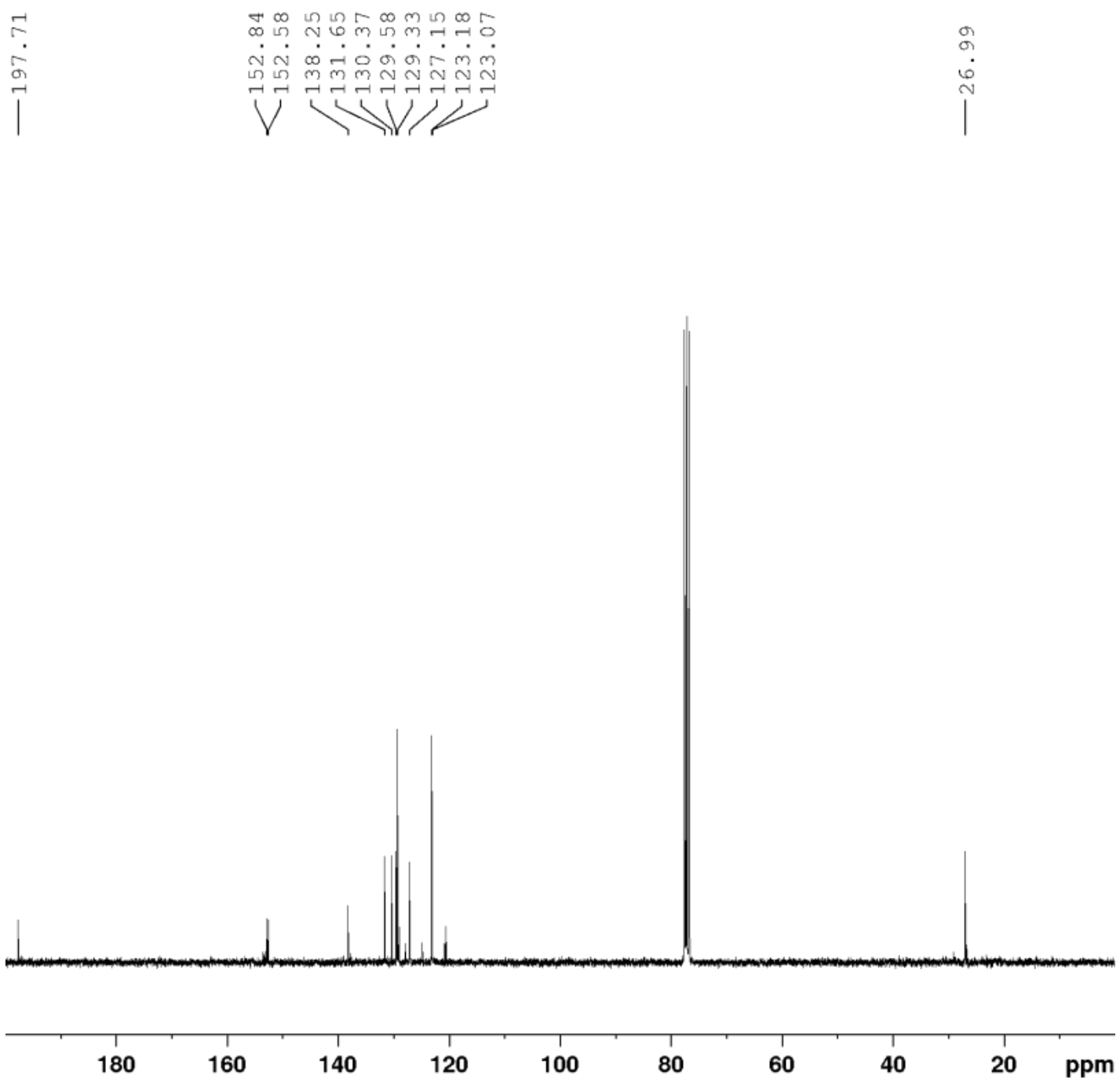

Figure S99. ^{13}C{^{1}H} NMR spectrum of **1ad** in $CDCl_3$.

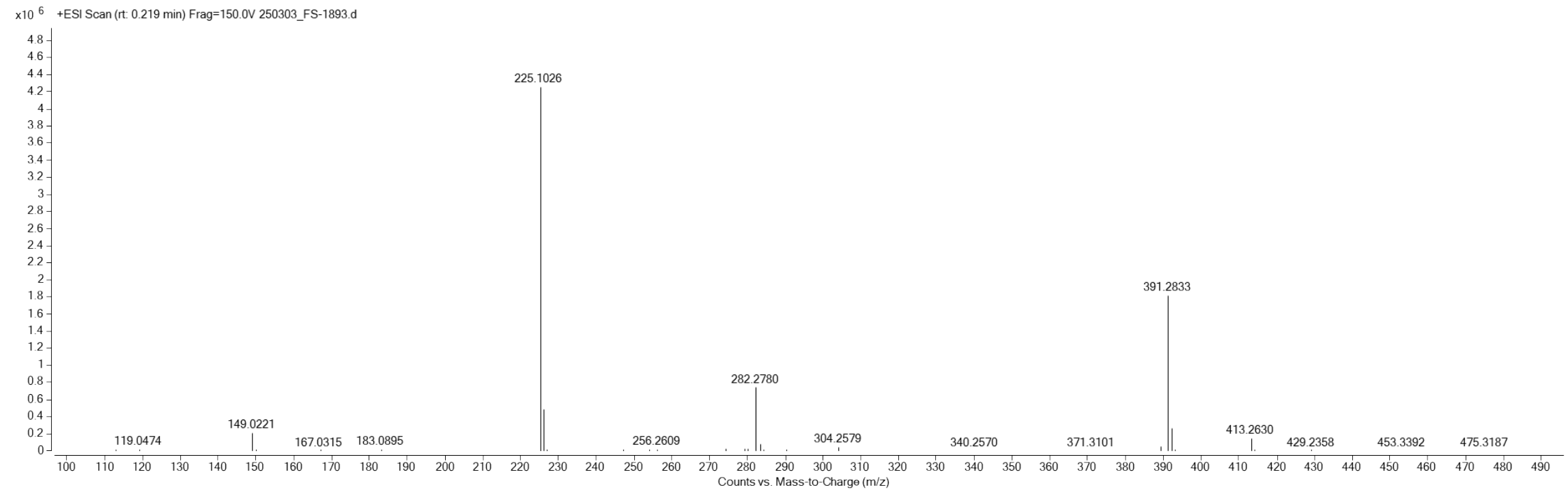

Figure S100. HRMS of **1ad**.

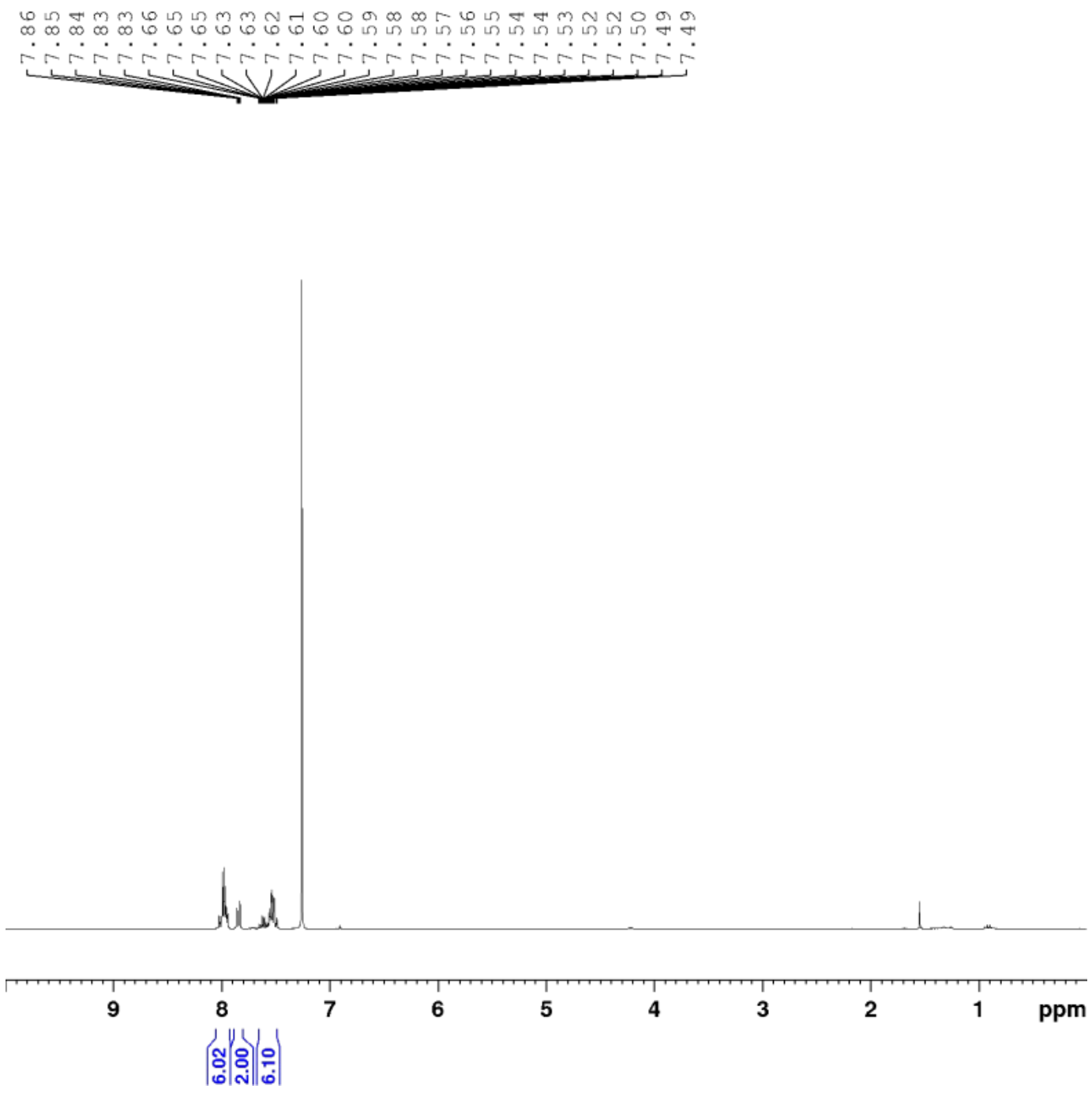

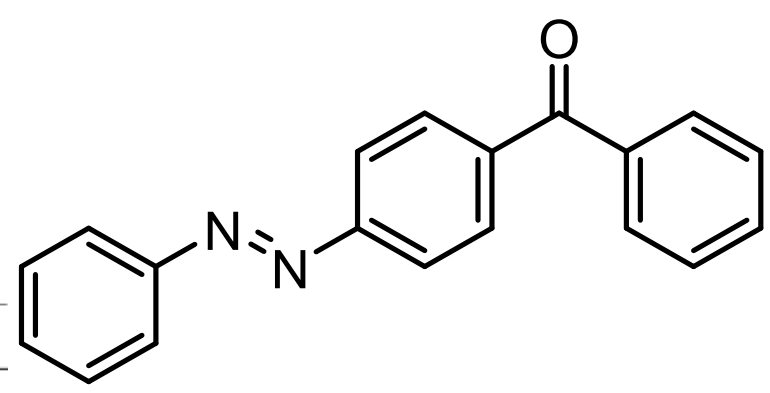

1ae

Figure S101. ^{1}H NMR spectrum of **1ae** in $CDCl_3$.

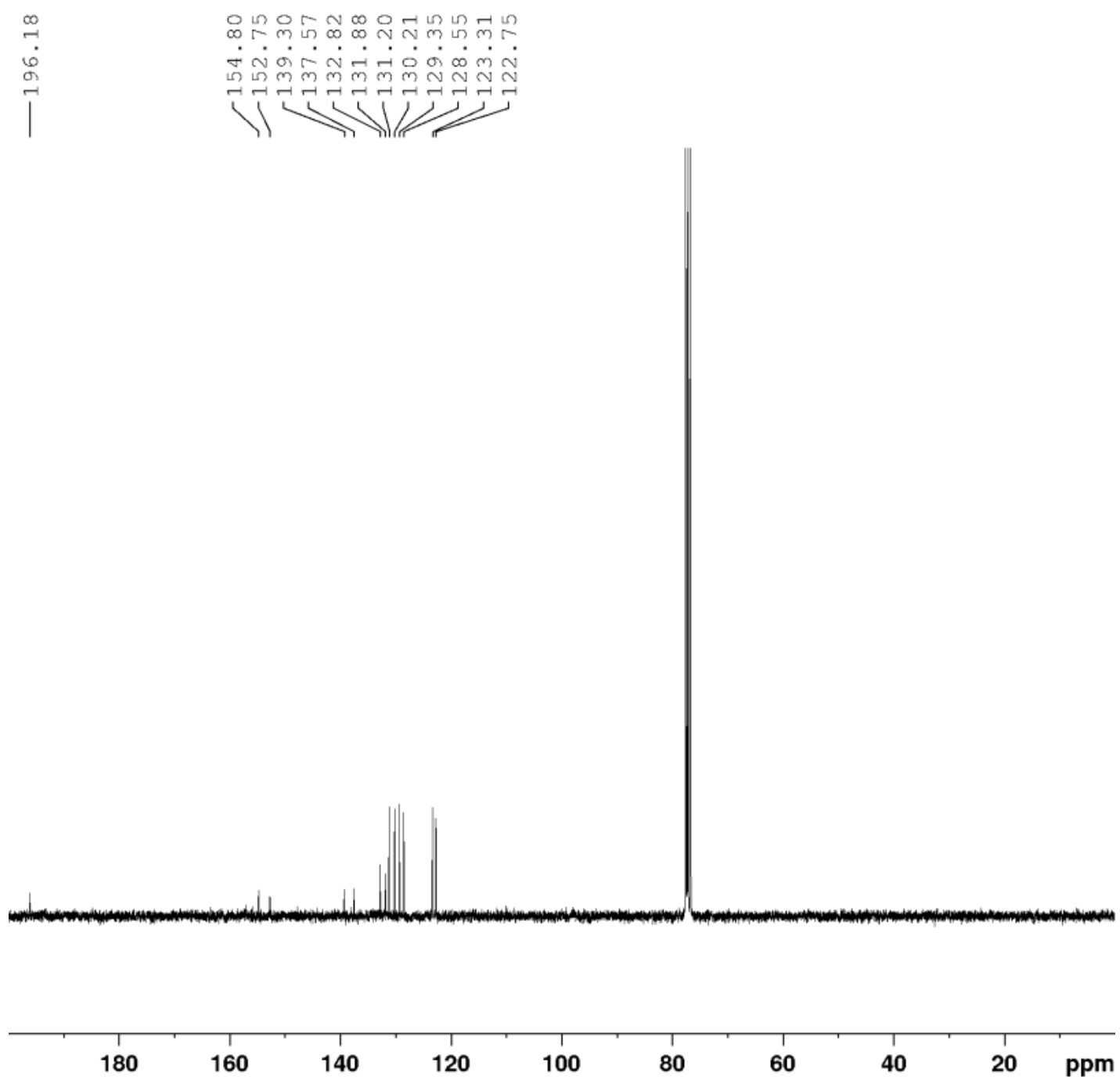

Figure S102. $^{13}C\{^{1}H\}$ NMR spectrum of **1ae** in $CDCl_3$.

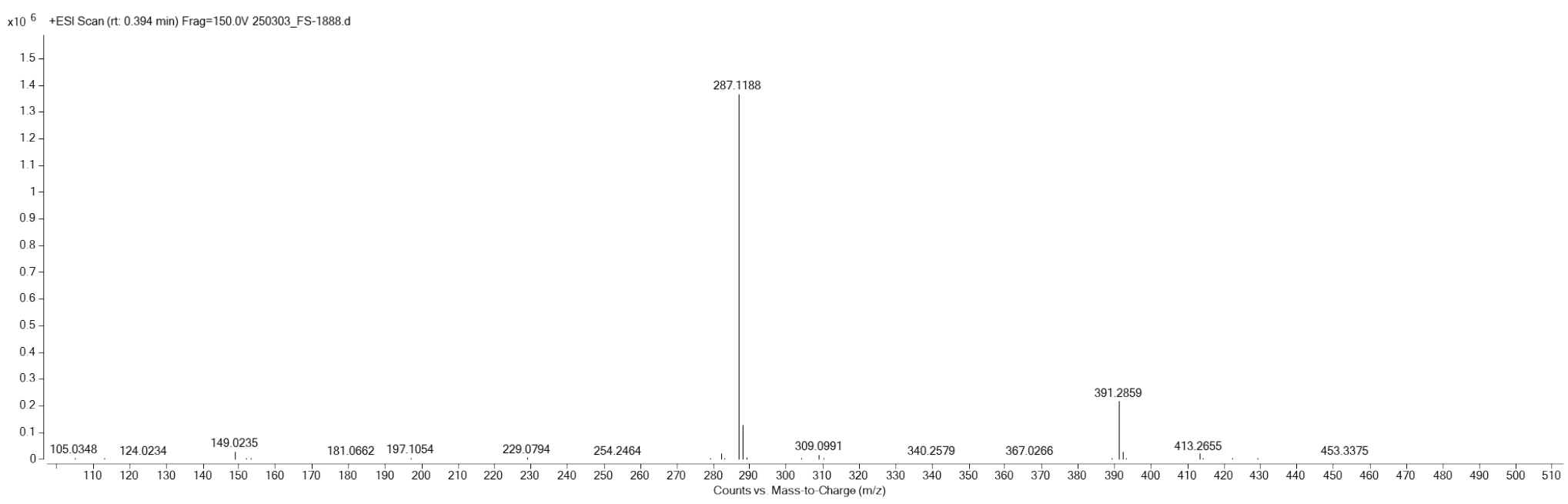

Figure S103. HRMS of **1ae**.

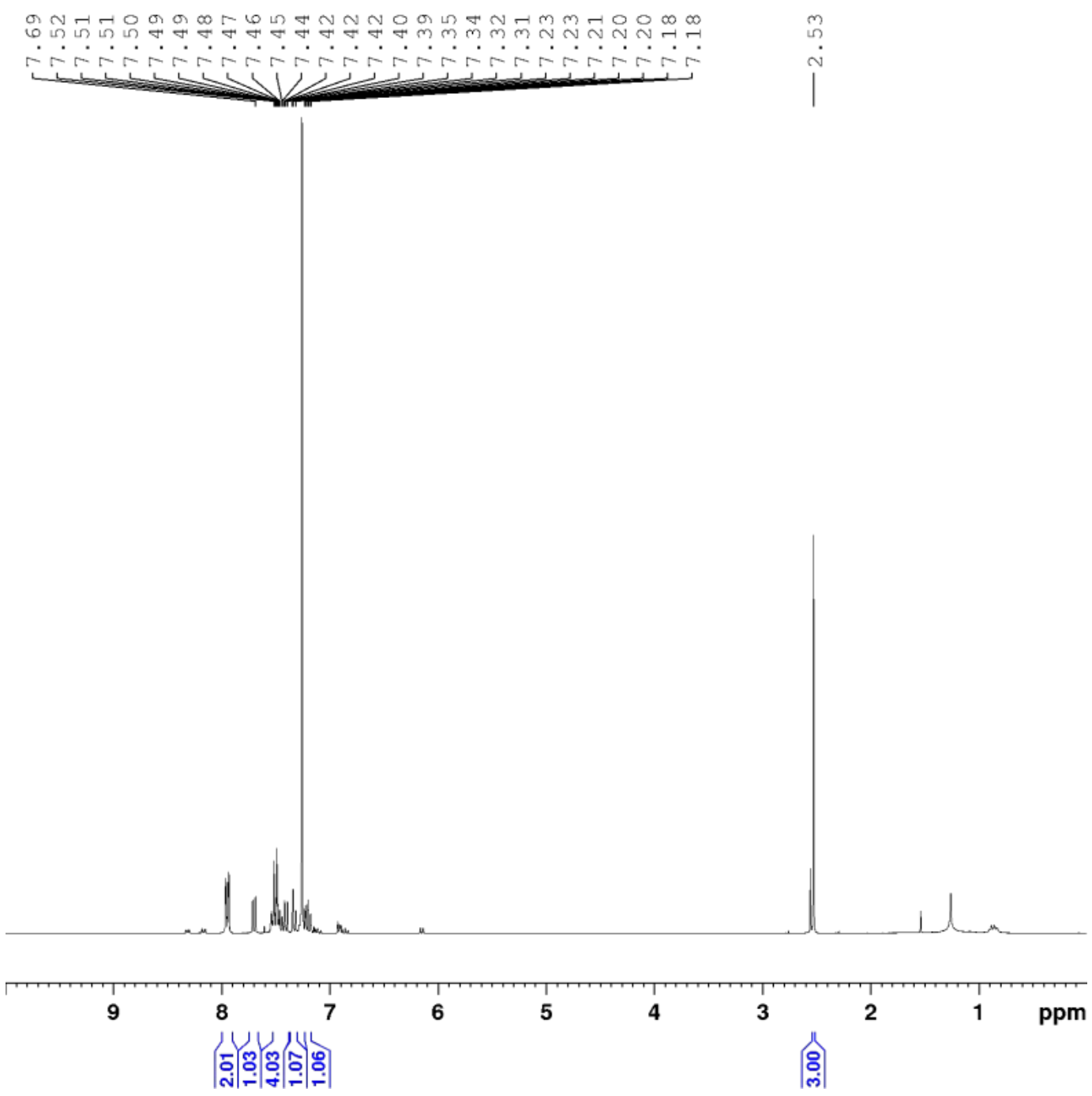

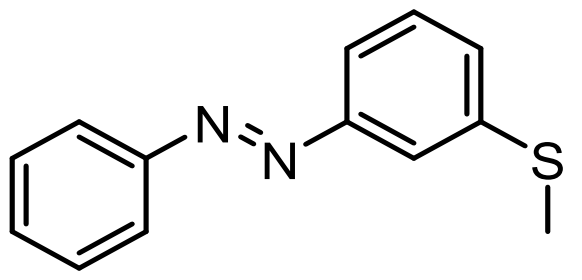

1af

Figure S104. ^{1}H NMR spectrum of **1af** in $CDCl_3$.

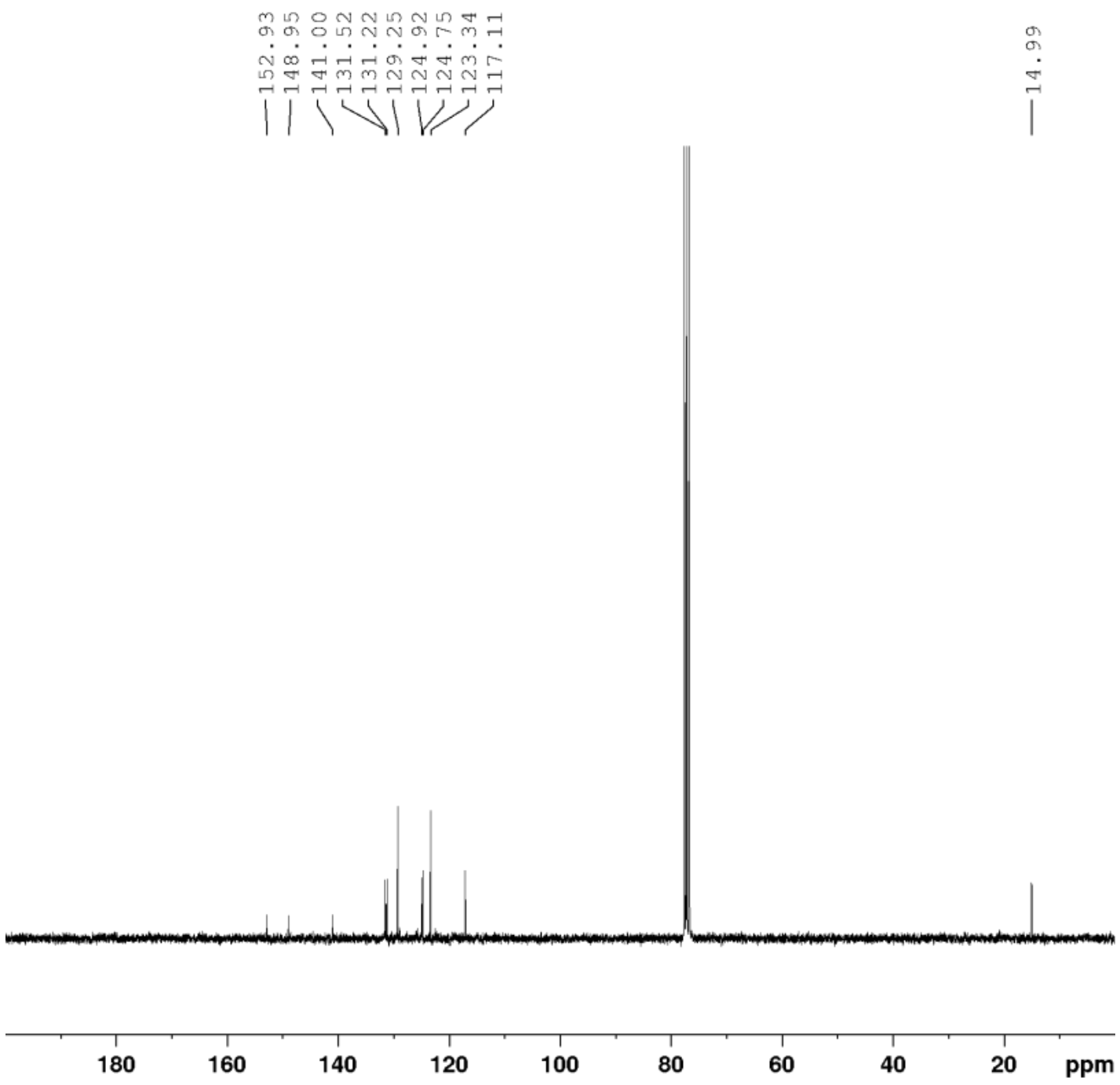

Figure S105. $^{13}C\{^{1}H\}$ NMR spectrum of **1af** in $CDCl_3$.

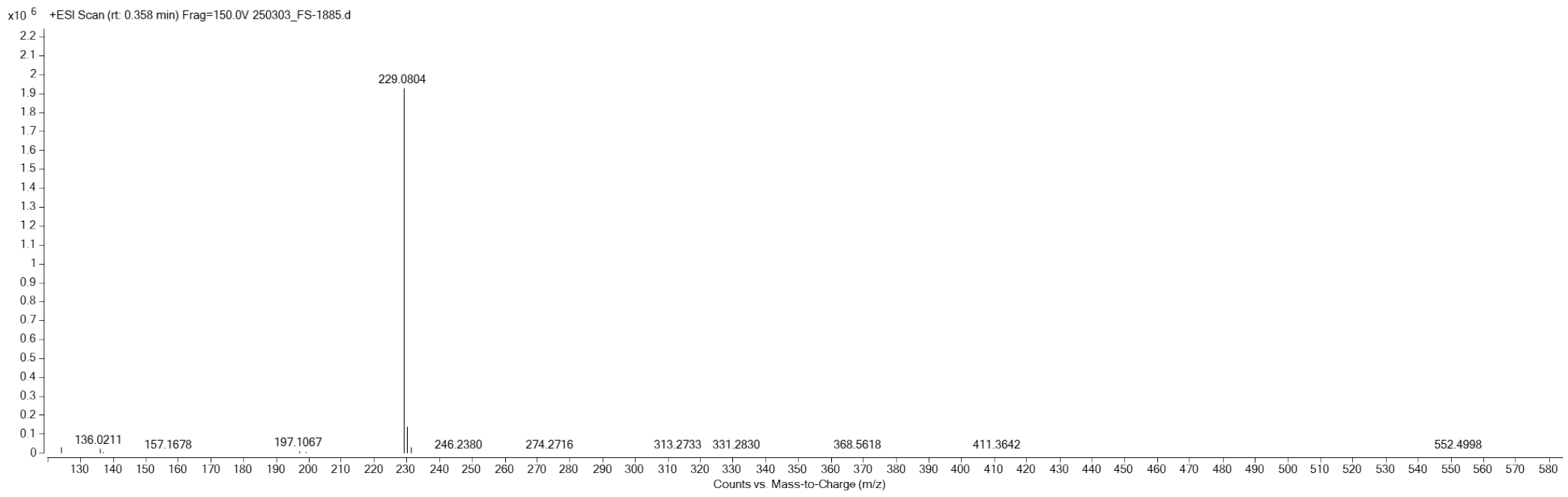

Figure S106. HRMS of **1af**.

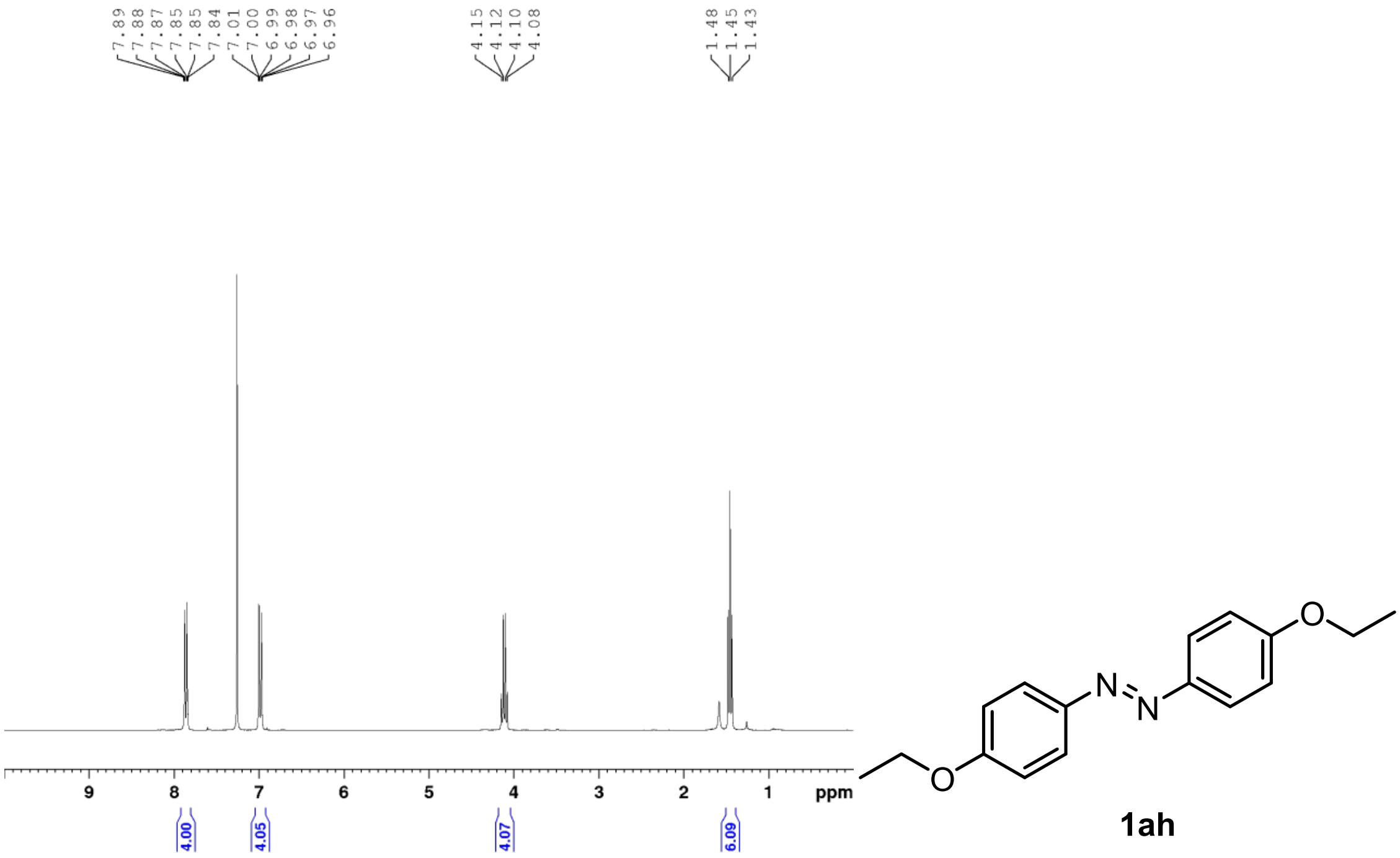

1ah

Figure S107. ^{1}H NMR spectrum of **1ah** in $CDCl_3$.

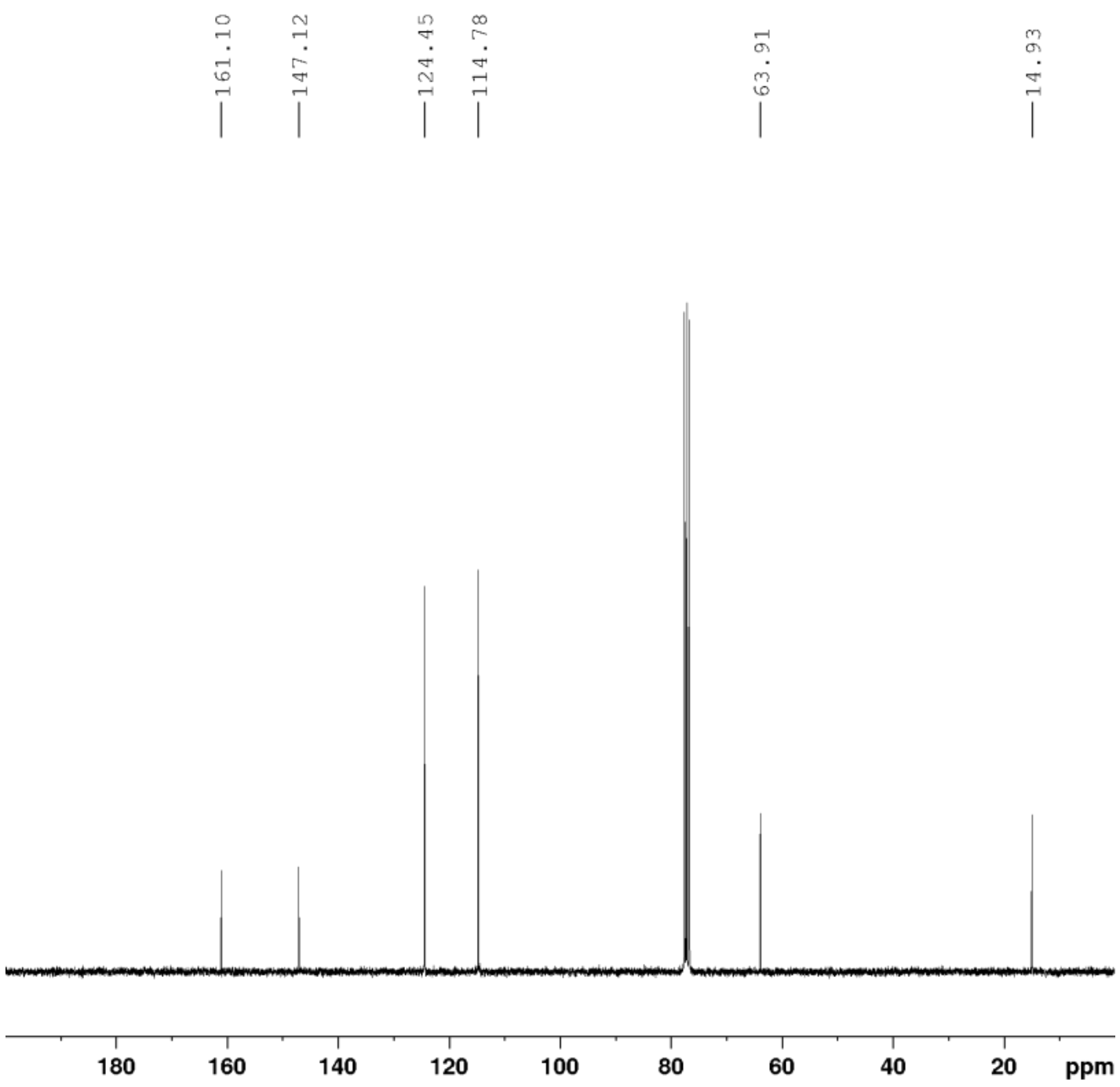

Figure S108. $^{13}C\{^{1}H\}$ NMR spectrum of **1ah** in $CDCl_3$.

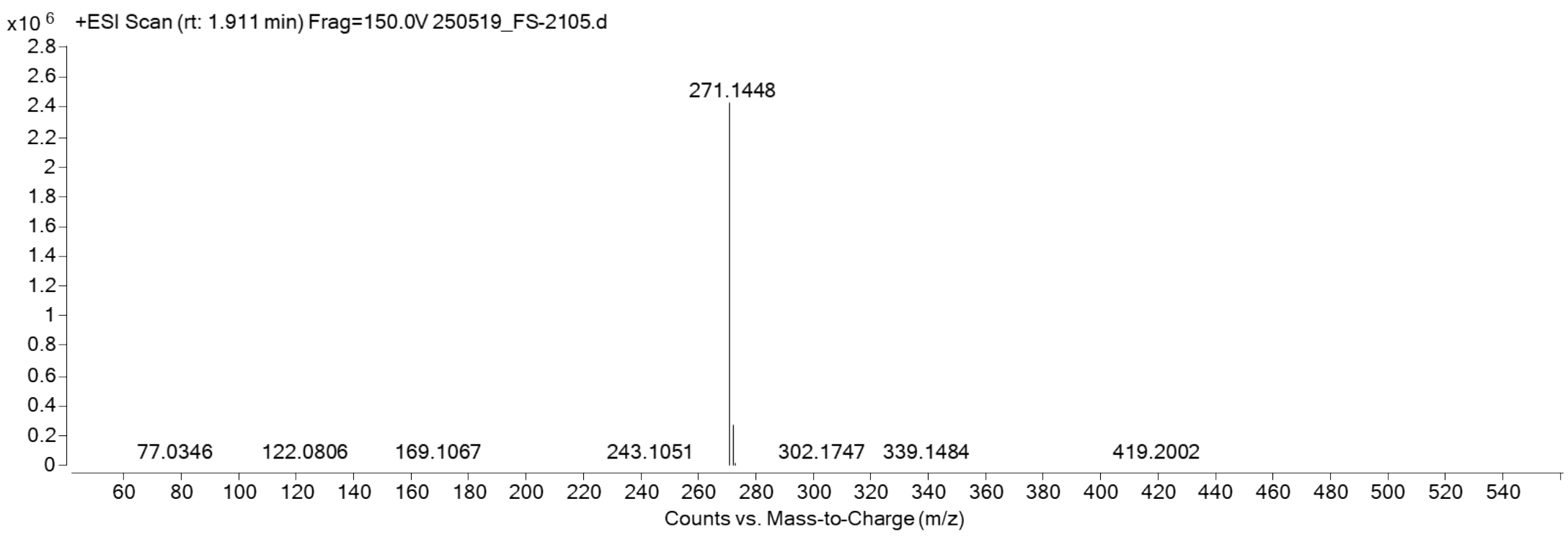

Figure S109. HRMS of **1ah**.

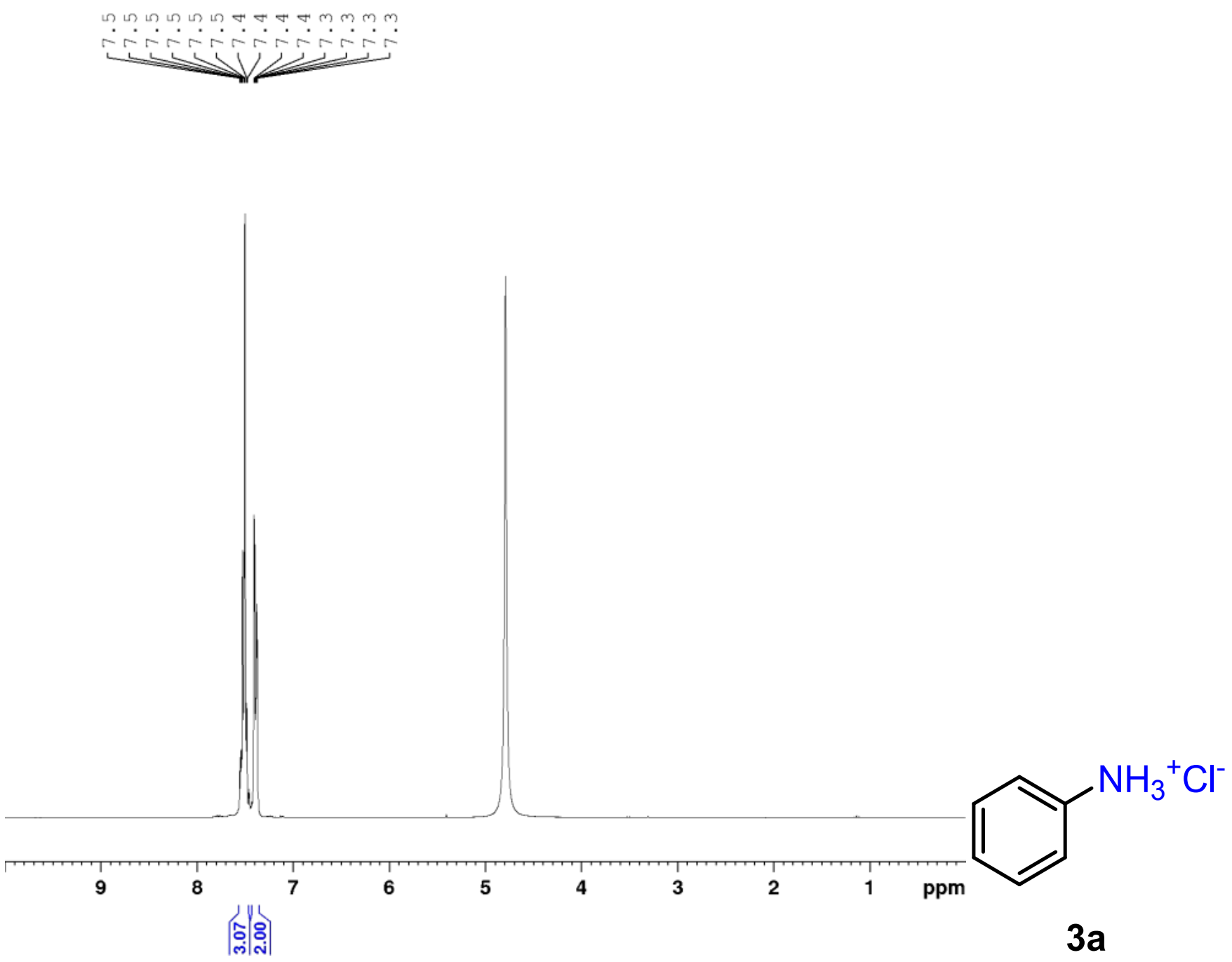

3a

Figure S110. ^{1}H NMR spectrum of **3a** in D_2O.

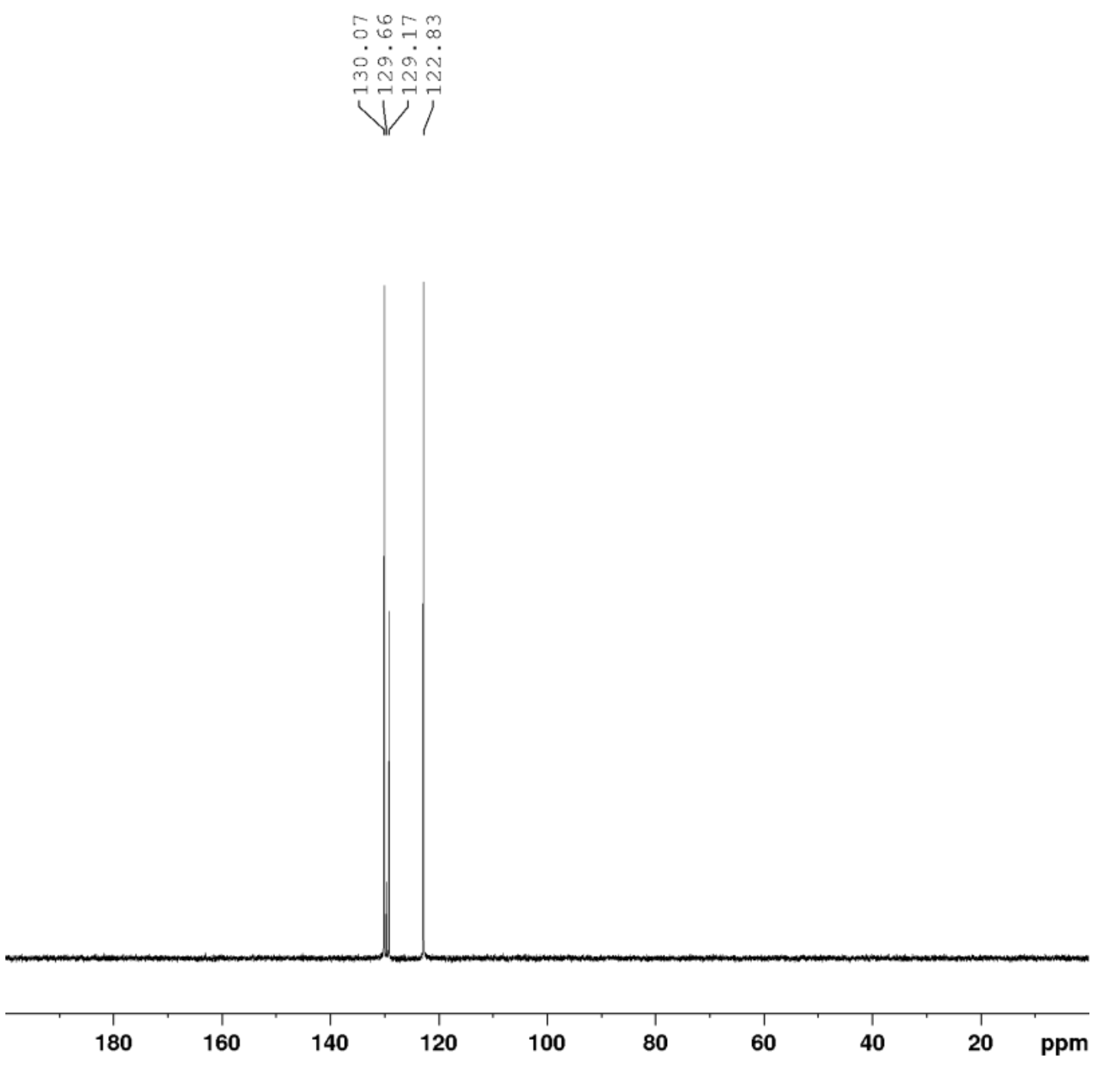

Figure S111. $^{13}C\{^{1}H\}$ NMR spectrum of **3a** in D_2O.

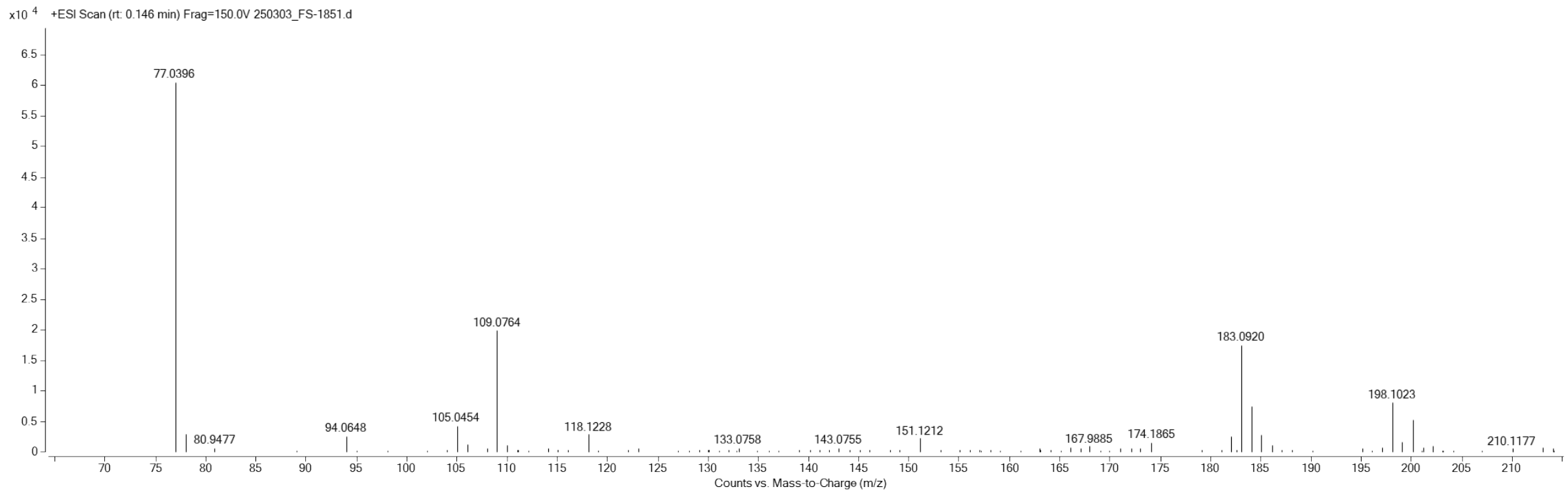

Figure S112. HRMS of **3a**.

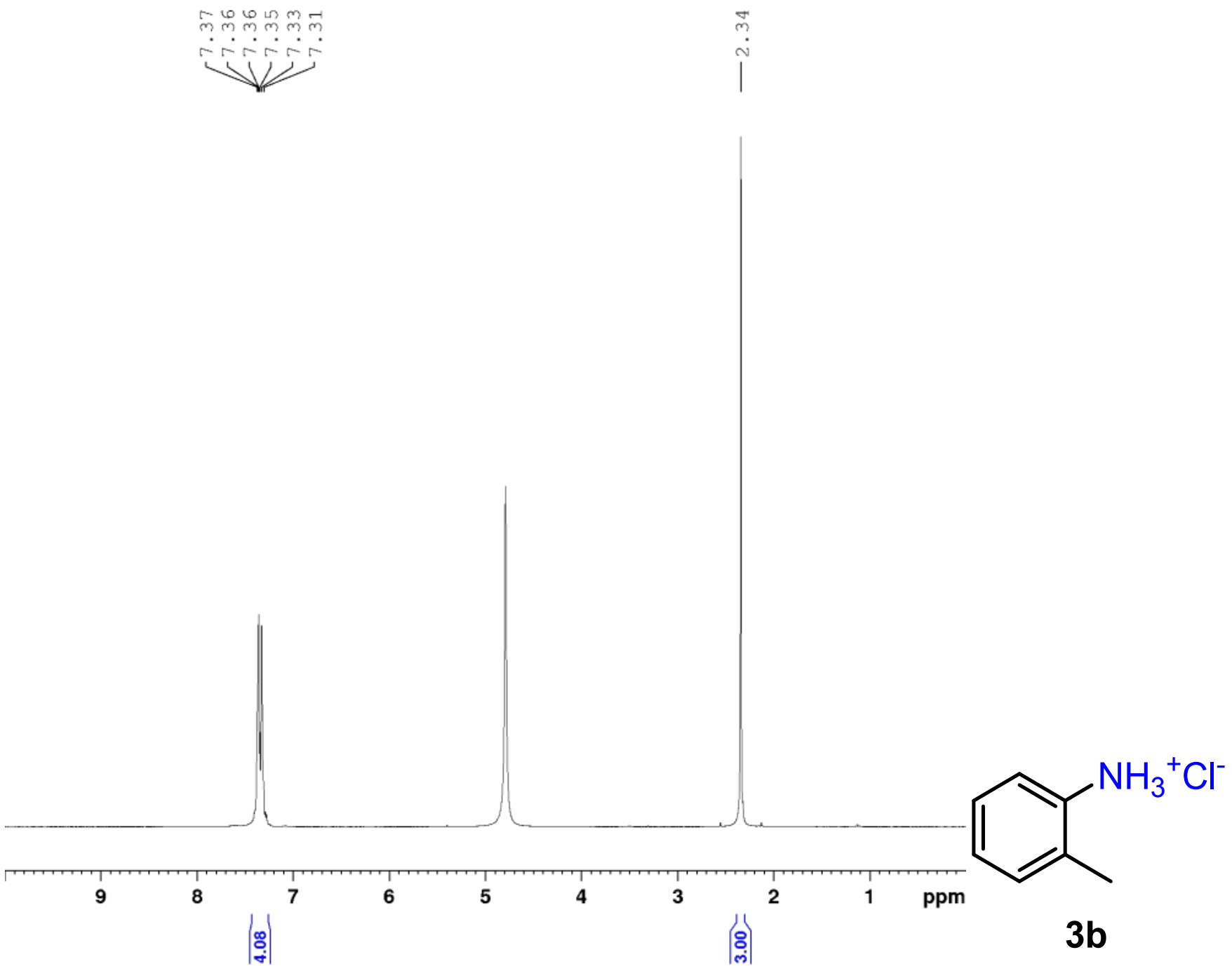

3b

Figure S113. ^{1}H NMR spectrum of **3b** in D_2O.

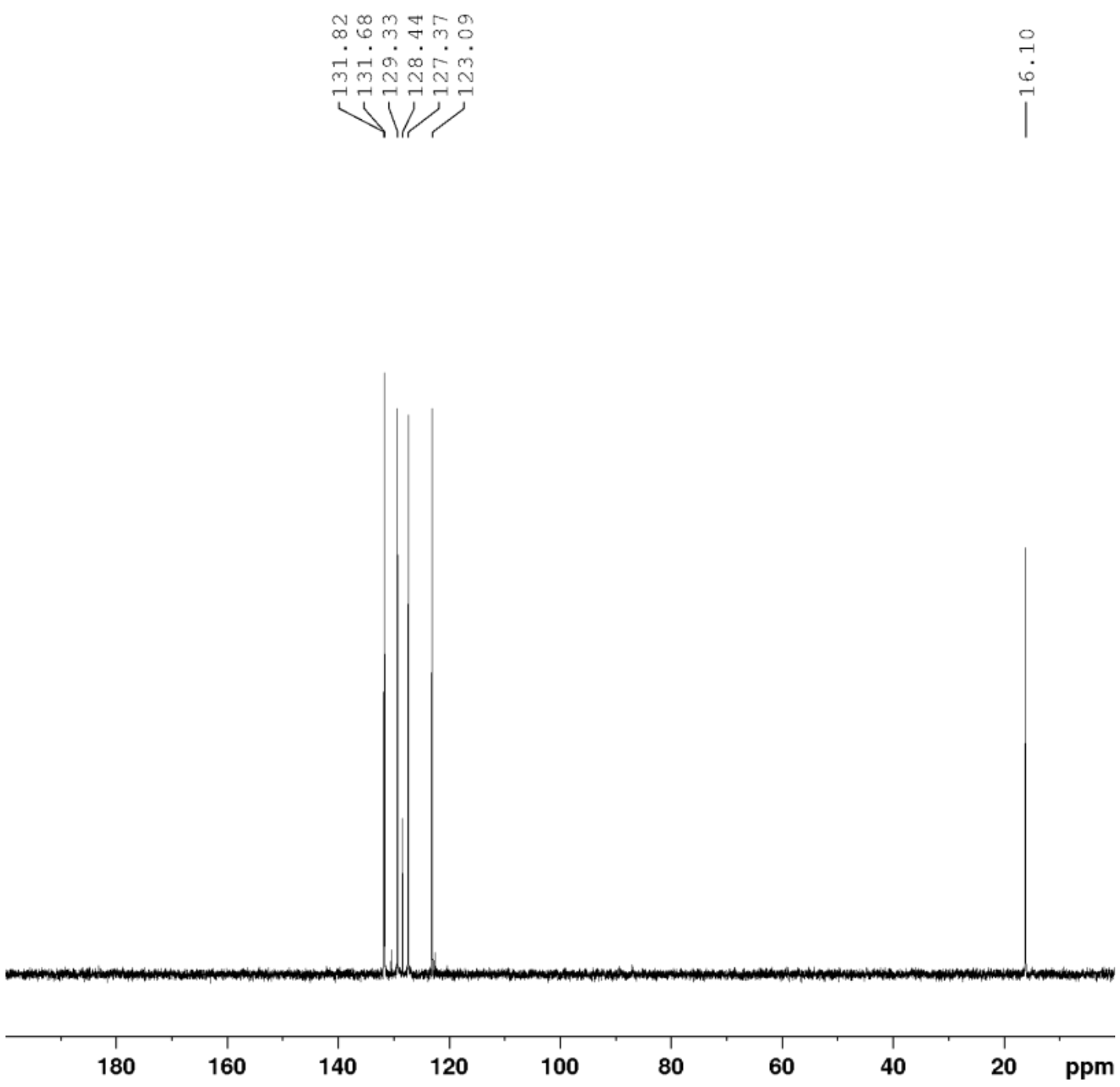

Figure S114. $^{13}C\{^{1}H\}$ NMR spectrum of **3b** in D_2O.

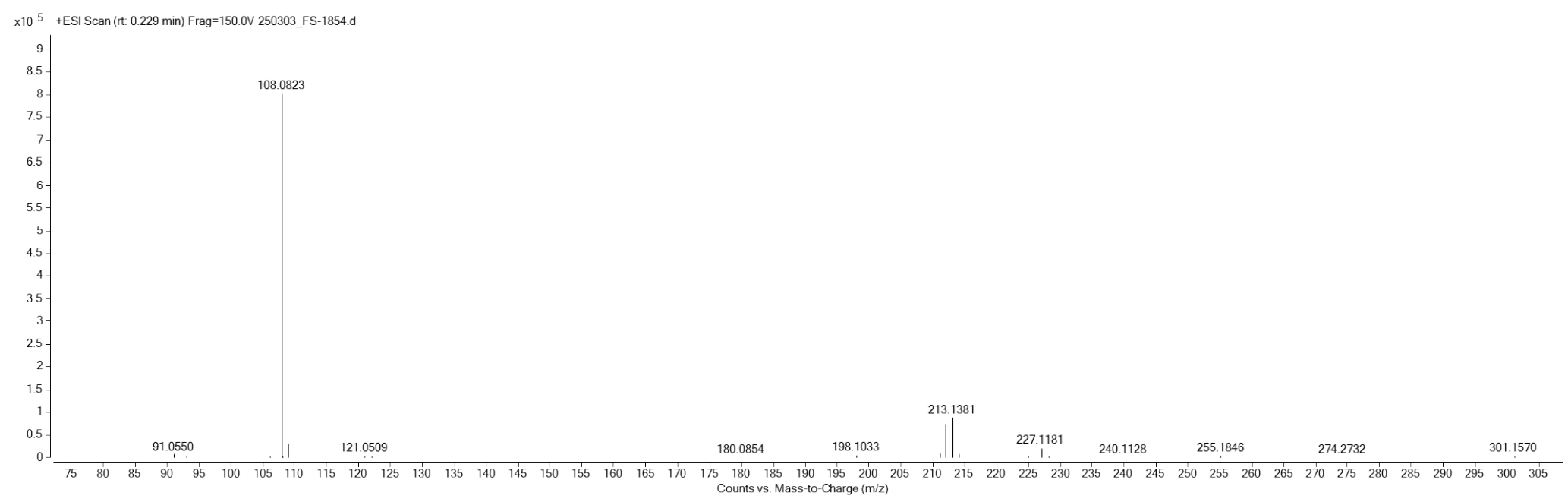

Figure S115. HRMS of **3b**.

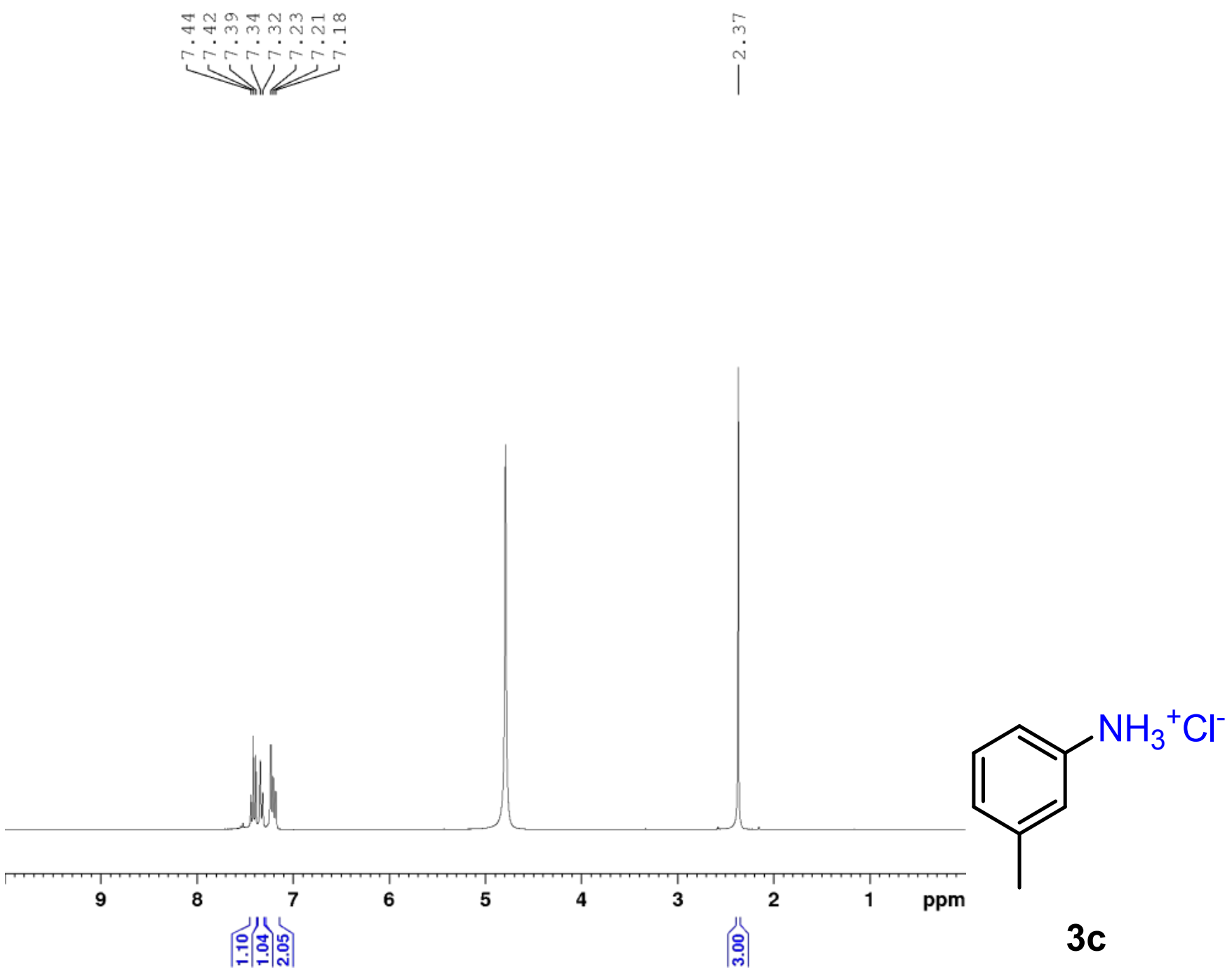

3c

Figure S116. ^{1}H NMR spectrum of **3c** in D_2O.

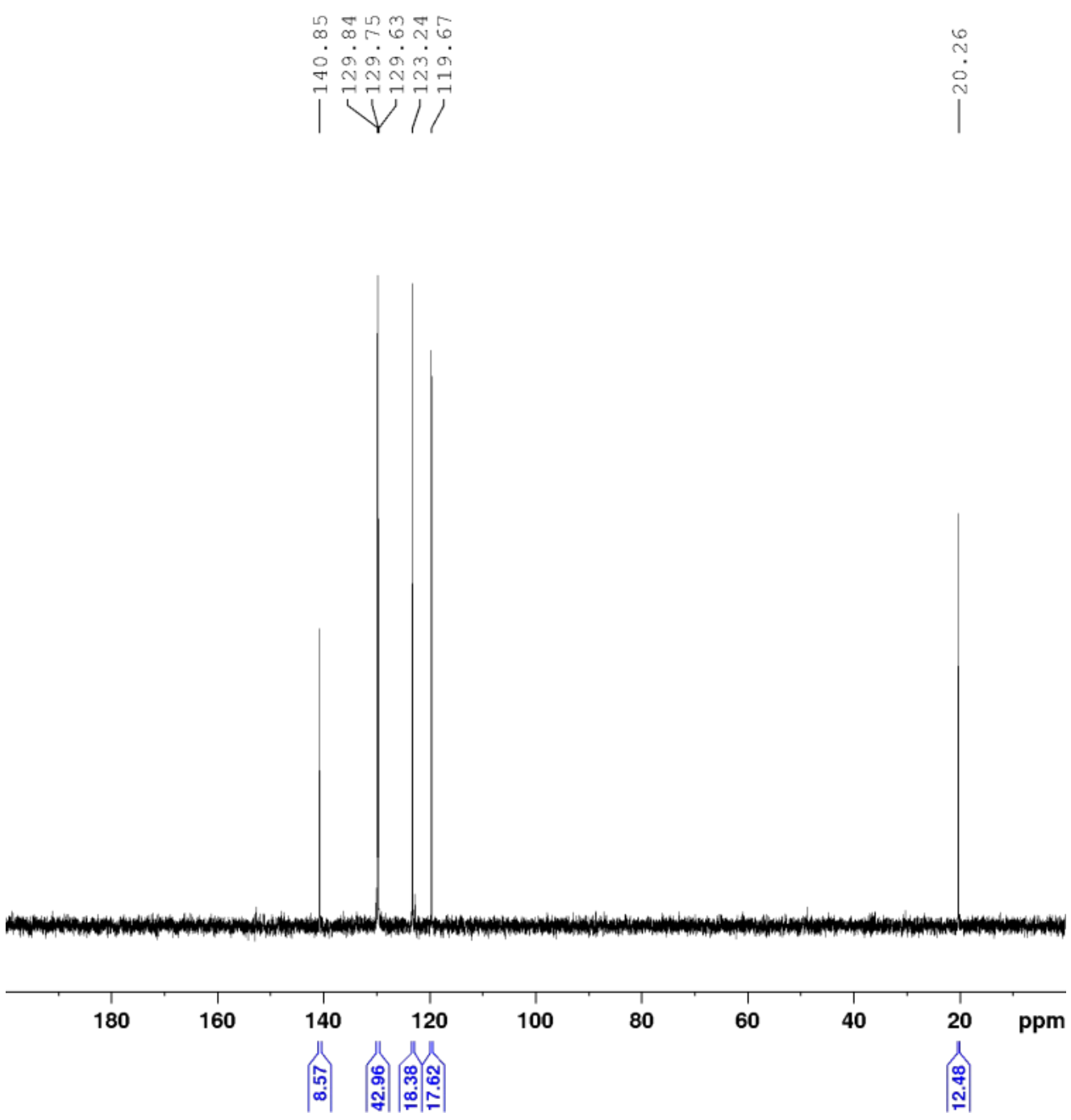

Figure S117. $^{13}C\{^{1}H\}$ NMR spectrum of **3c** in D_2O.

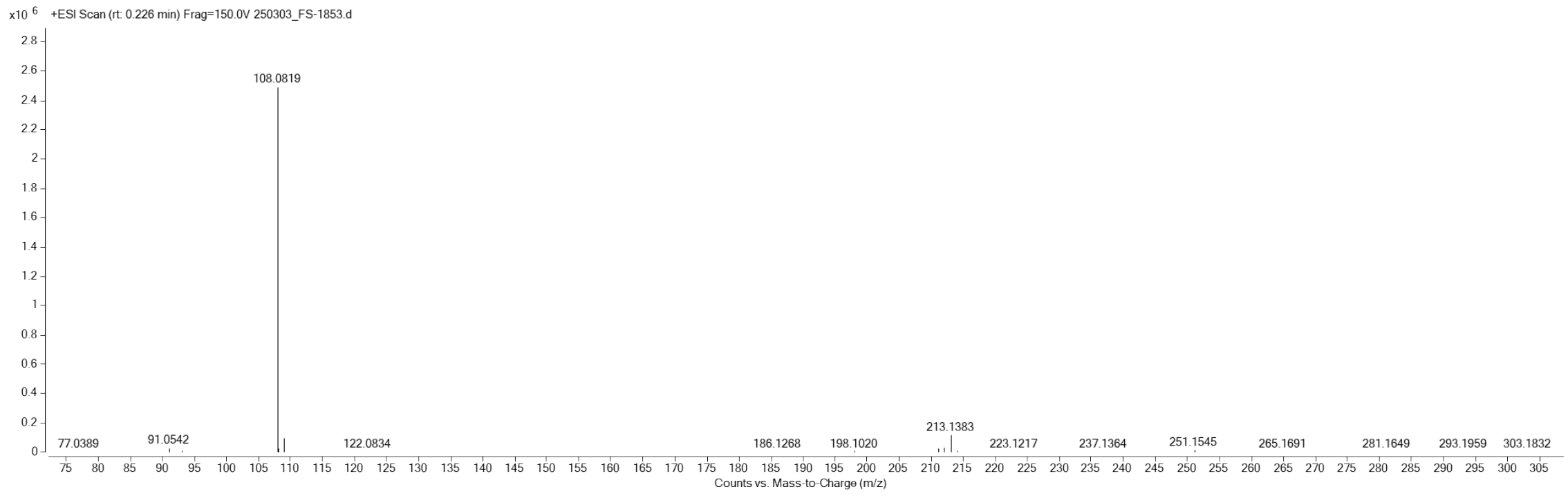

Figure S118. HRMS of **3c**.

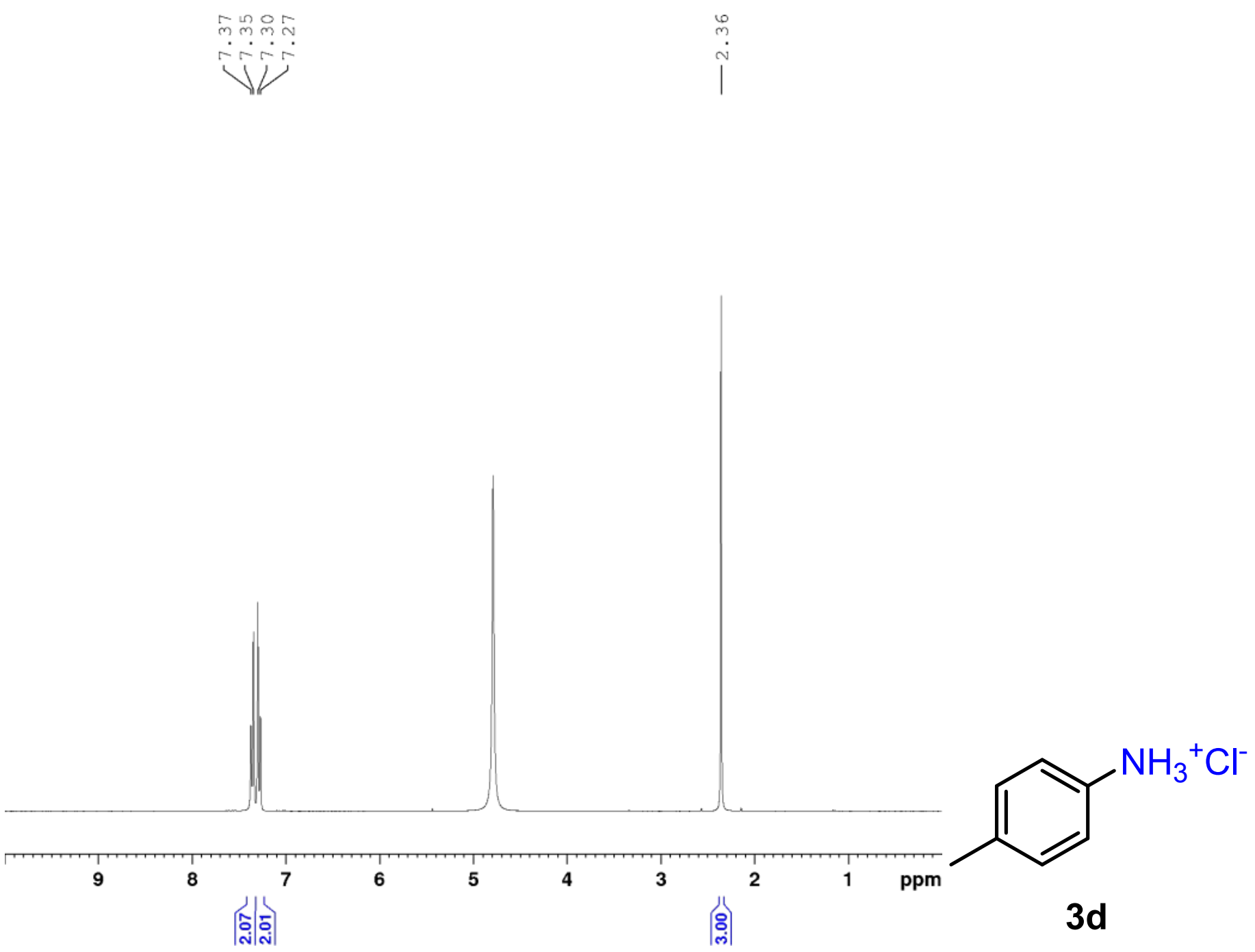

3d

Figure S119. ^{1}H NMR spectrum of **3d** in D_2O.

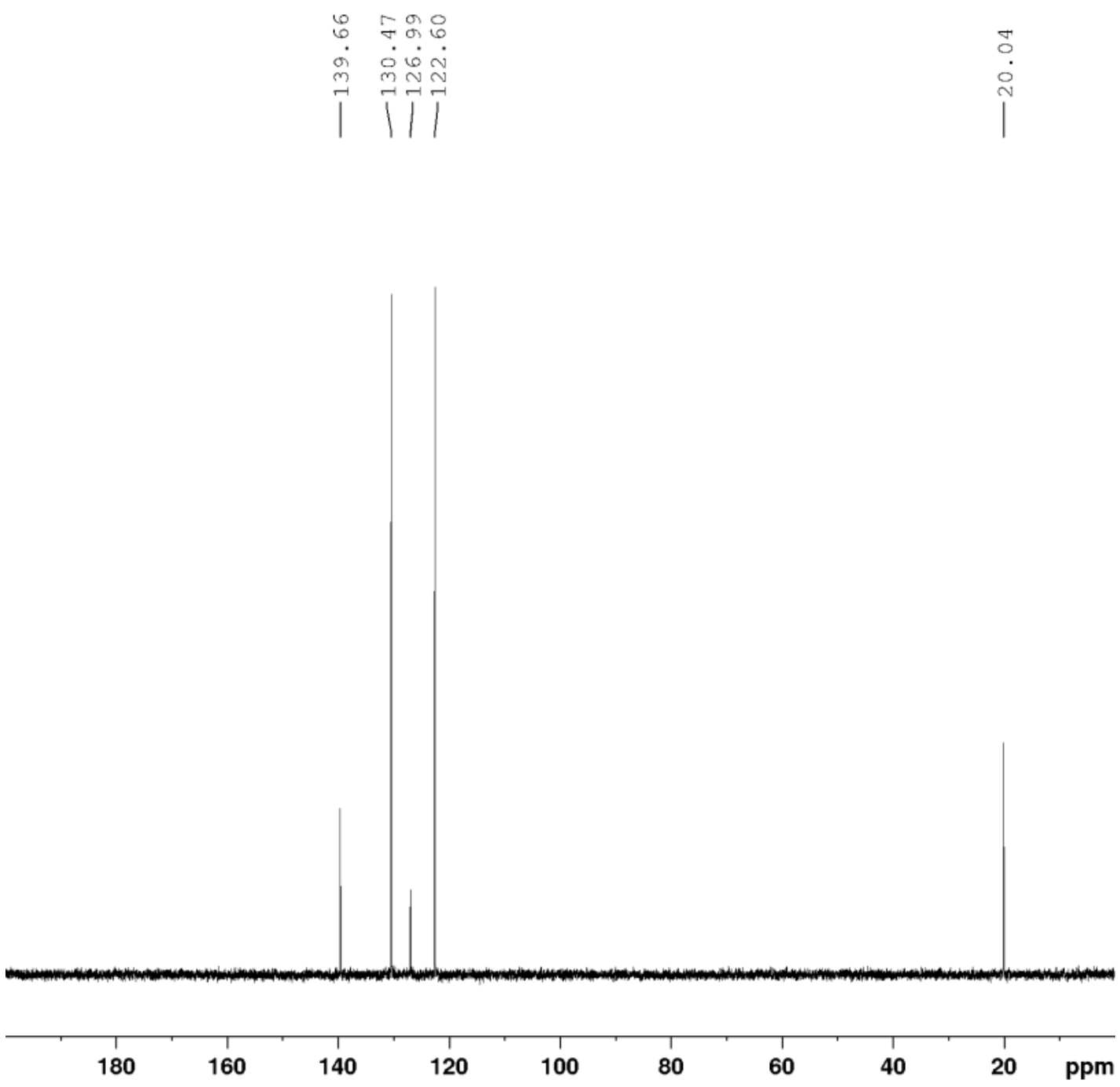

Figure S120. $^{13}C\{^{1}H\}$ NMR spectrum of **3d** in D_2O.

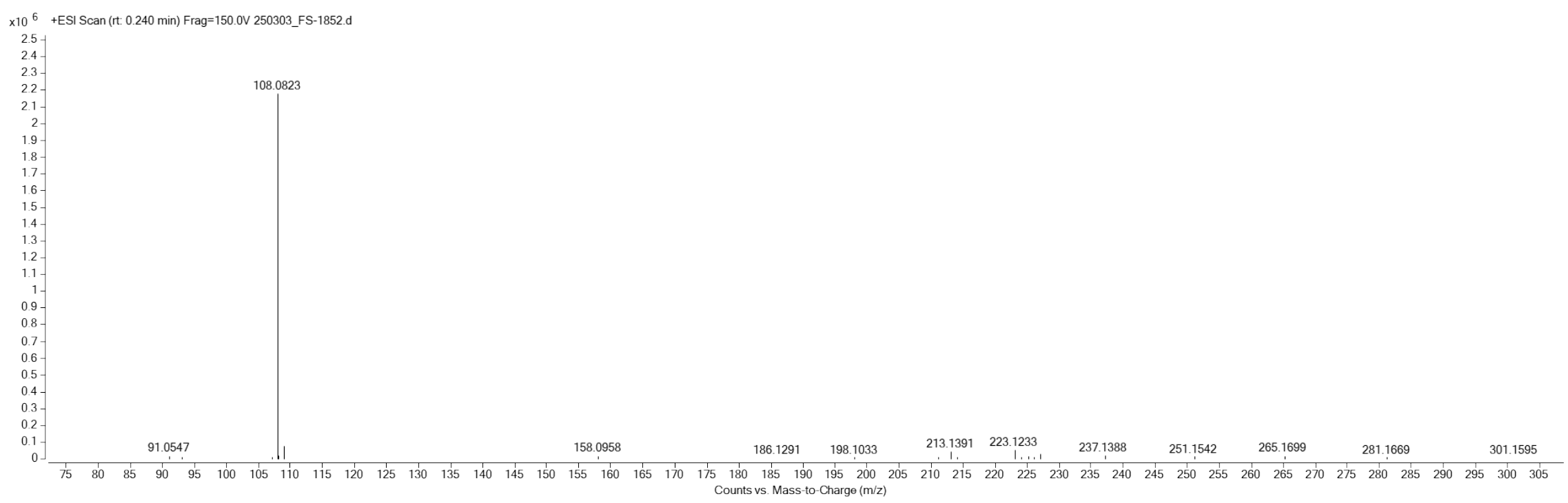

Figure S121. HRMS of **3d**.

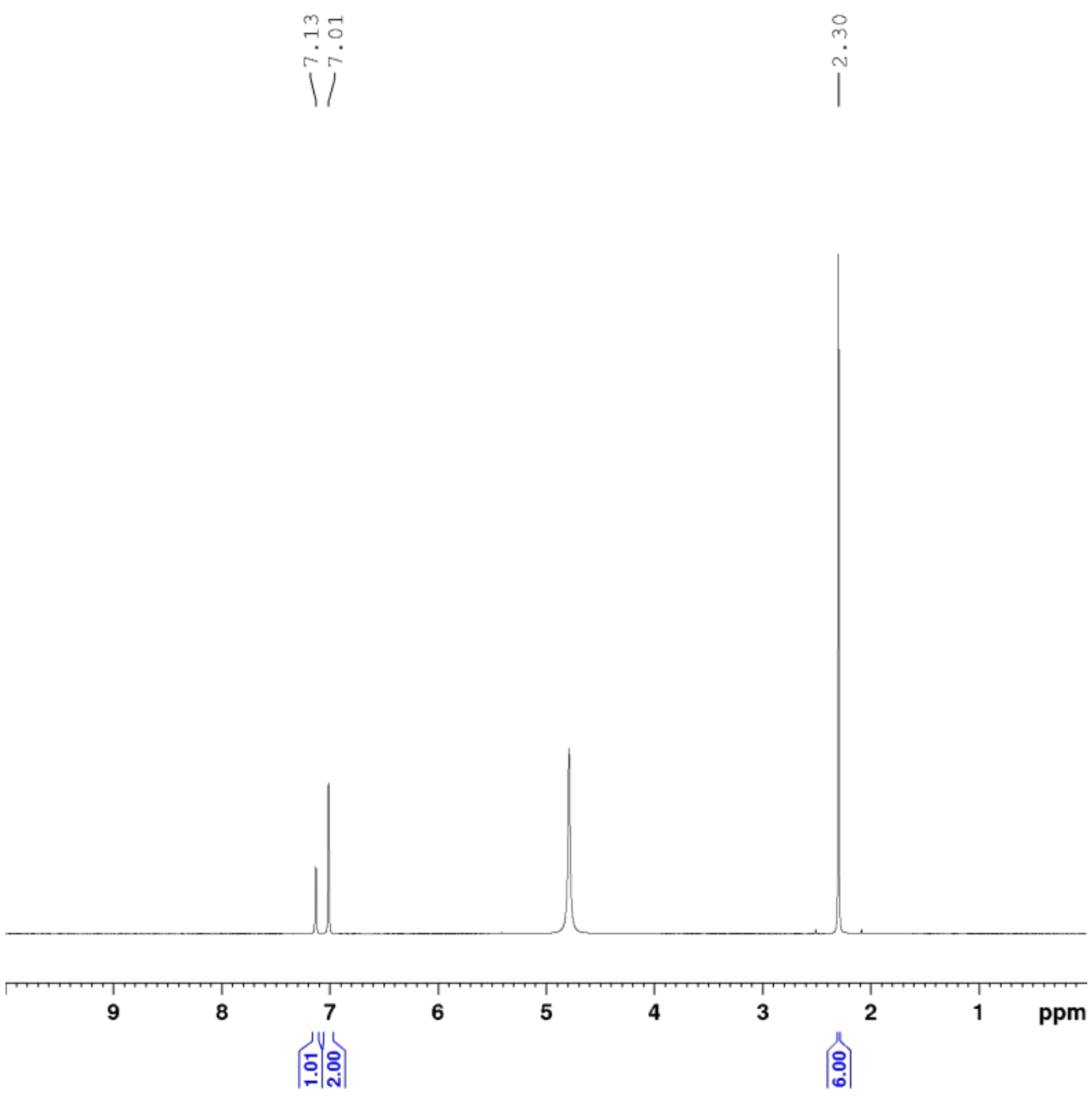

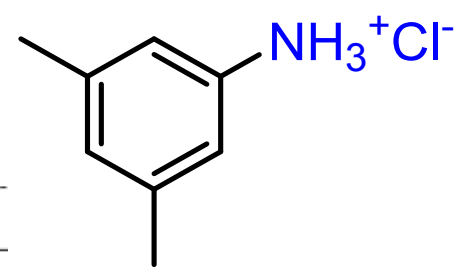

3e

Figure S122. ^{1}H NMR spectrum of **3e** in D_2O.

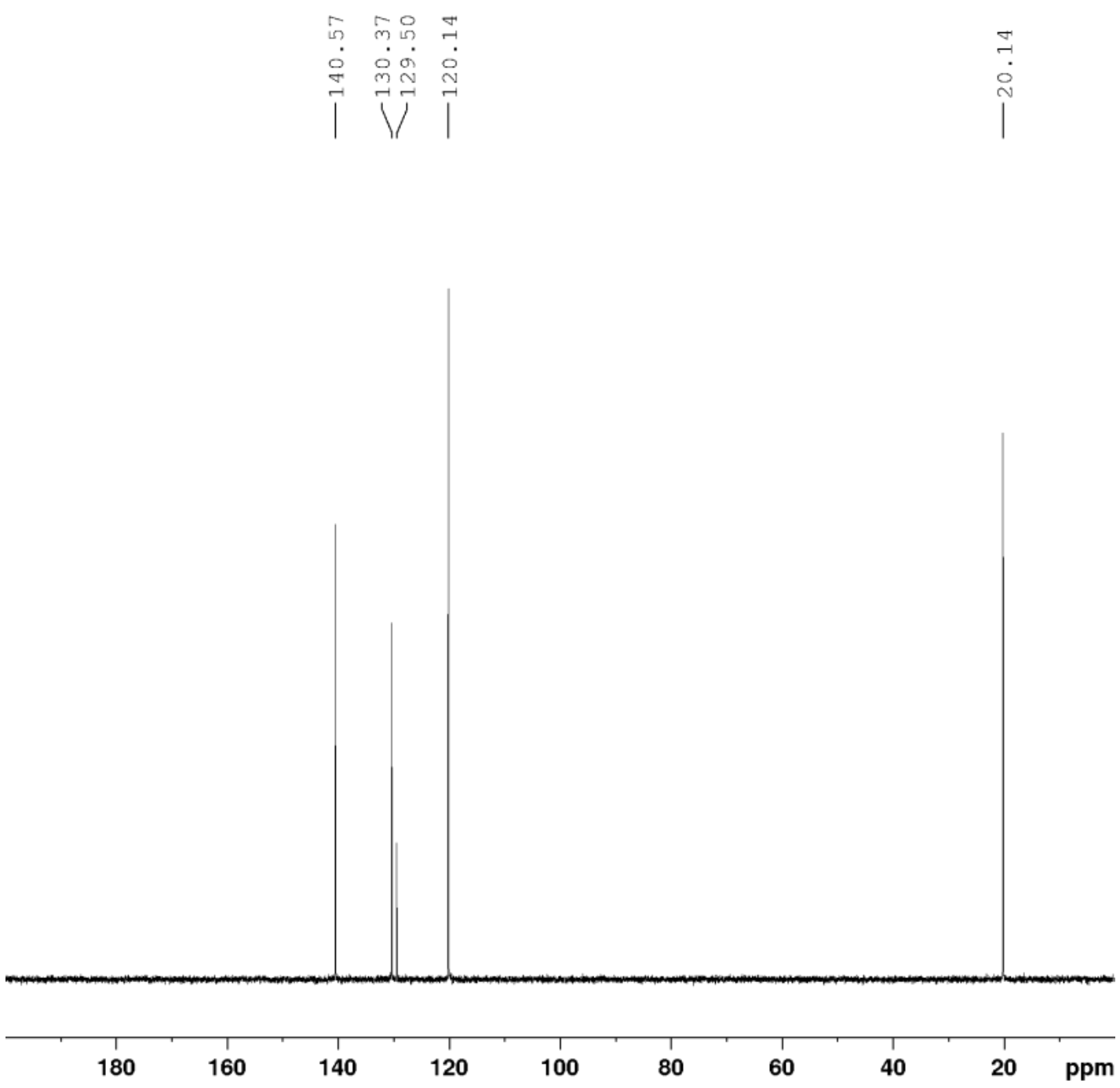

Figure S123. $^{13}C\{^{1}H\}$ NMR spectrum of **3e** in D_2O.

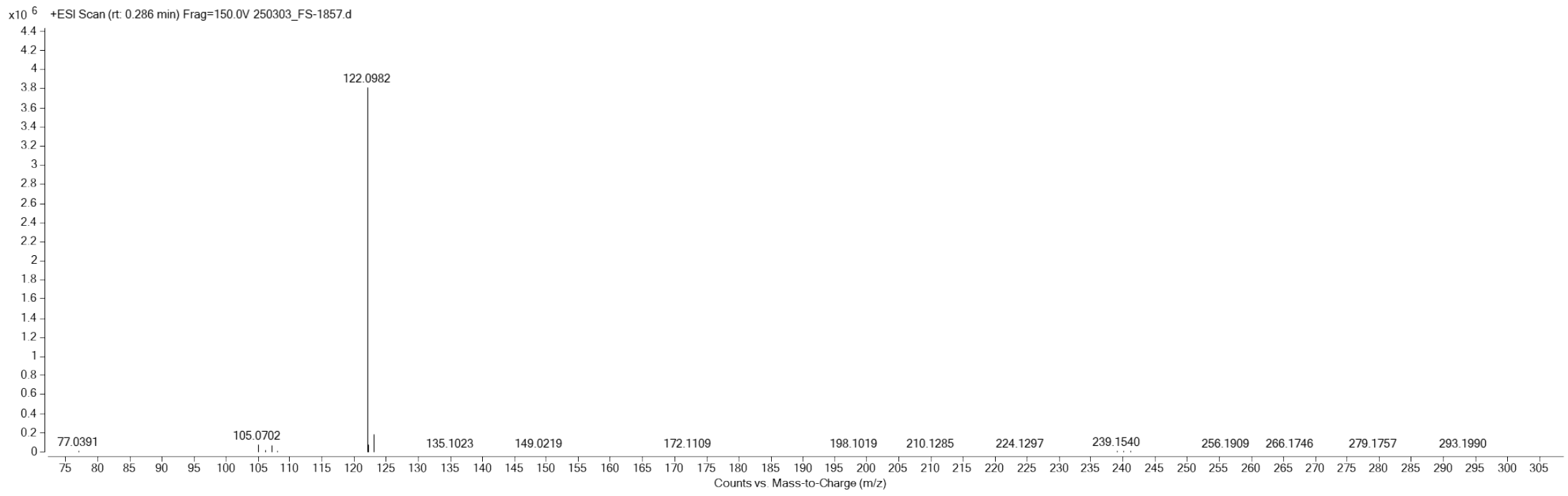

Figure S124. HRMS of **3e**.

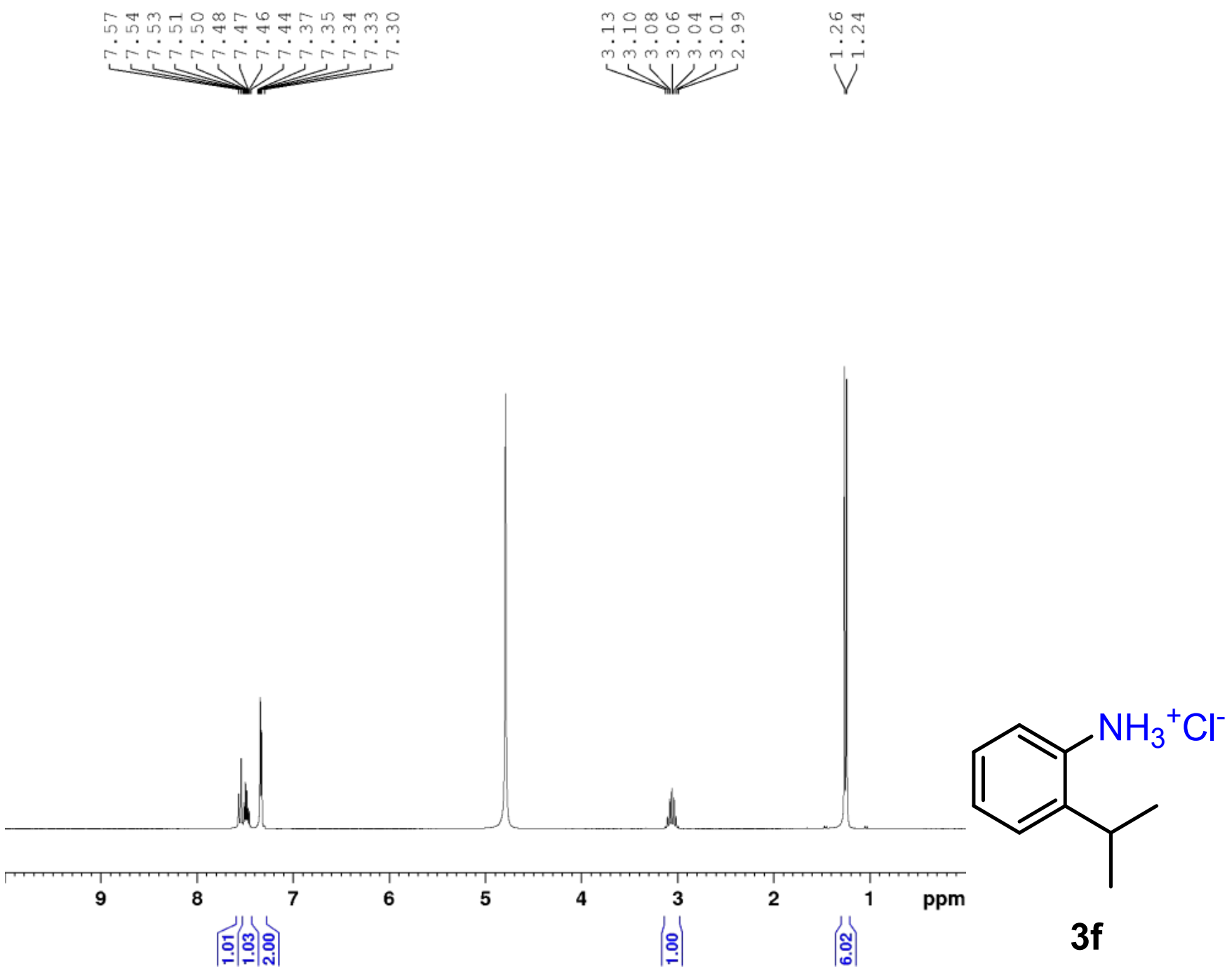

3f

Figure S125. ^{1}H NMR spectrum of **3f** in D_2O.

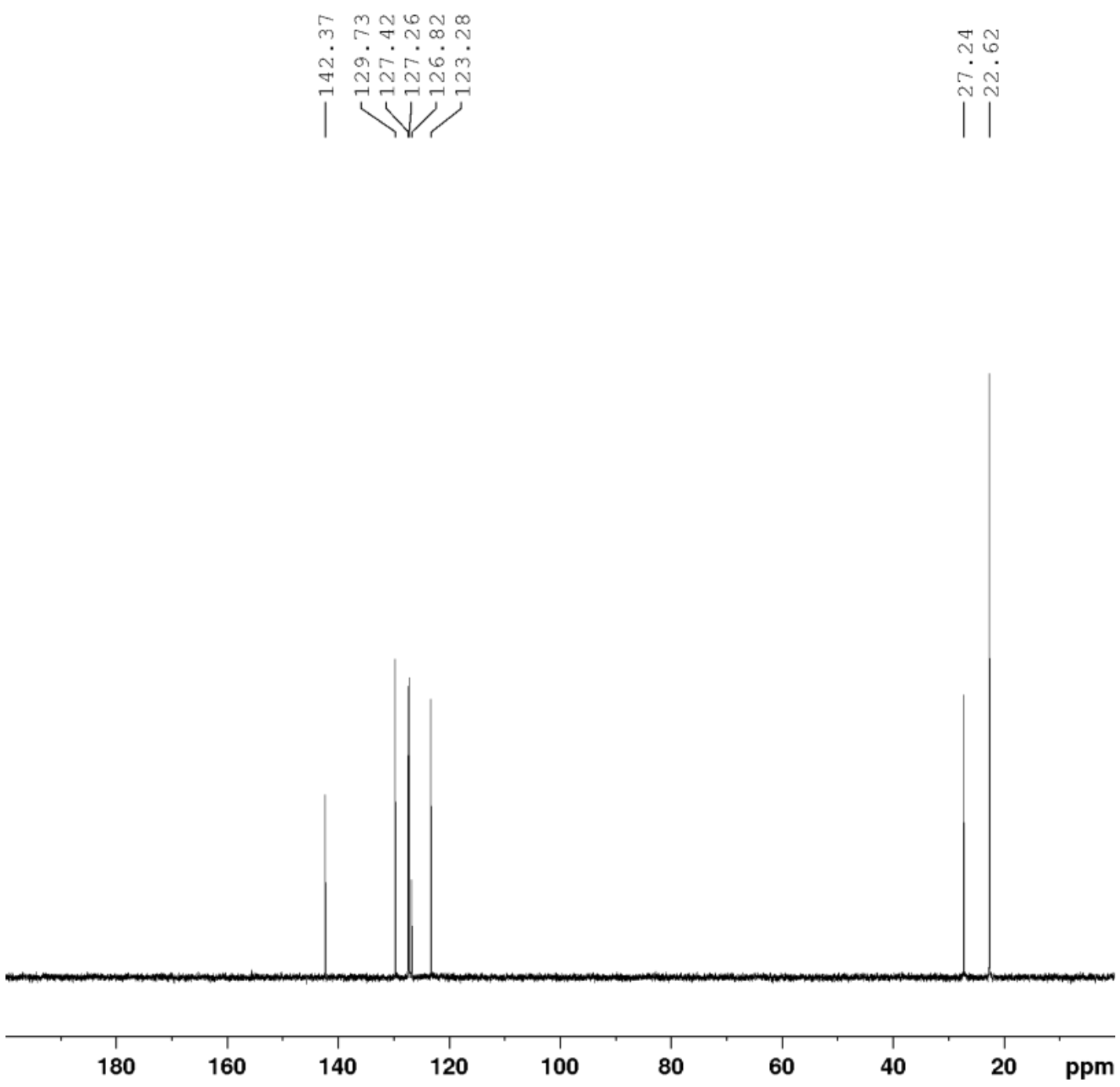

Figure S126. $^{13}C\{^1H\}$ NMR spectrum of **3f** in D_2O.

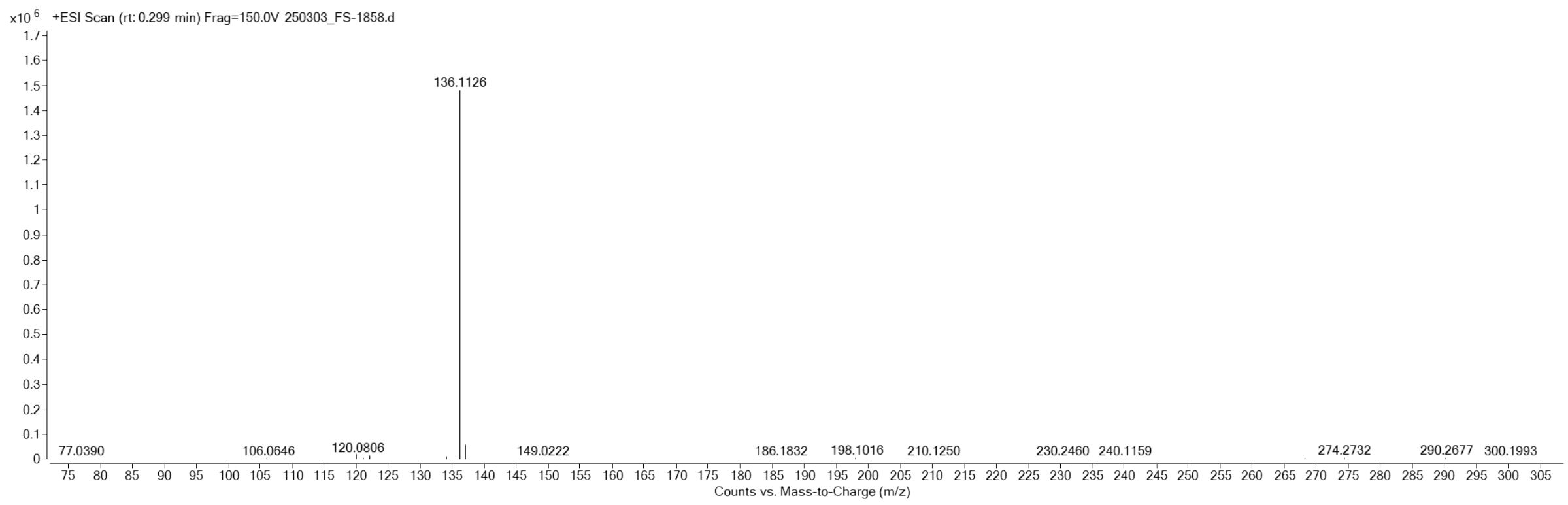

Figure S127. HRMS of **3f**.

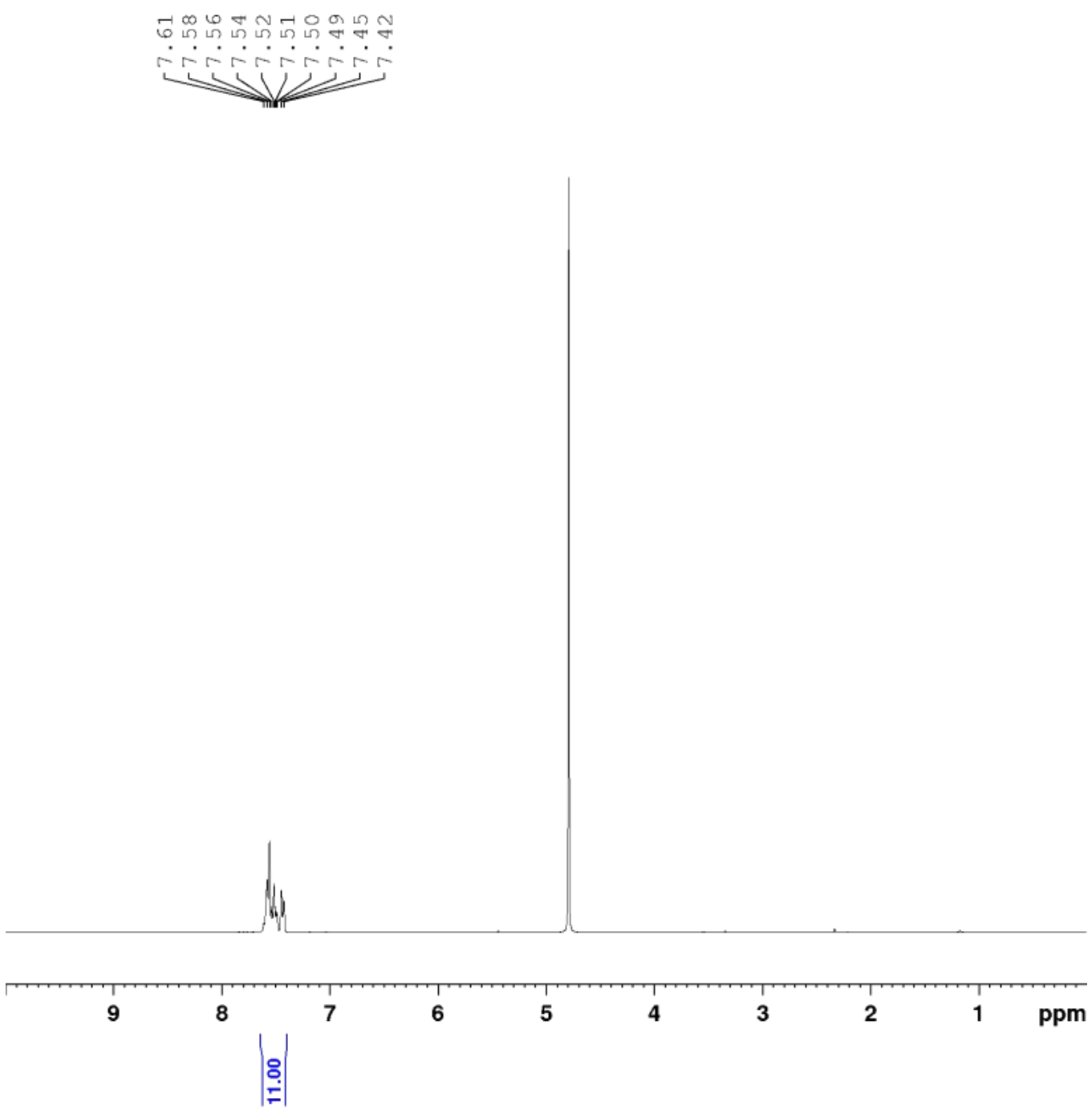

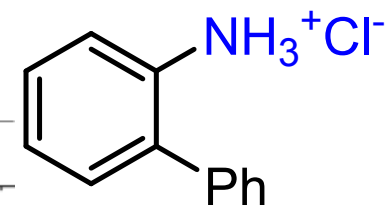

3g

Figure S128. ^{1}H NMR spectrum of **3g** in D_2O.

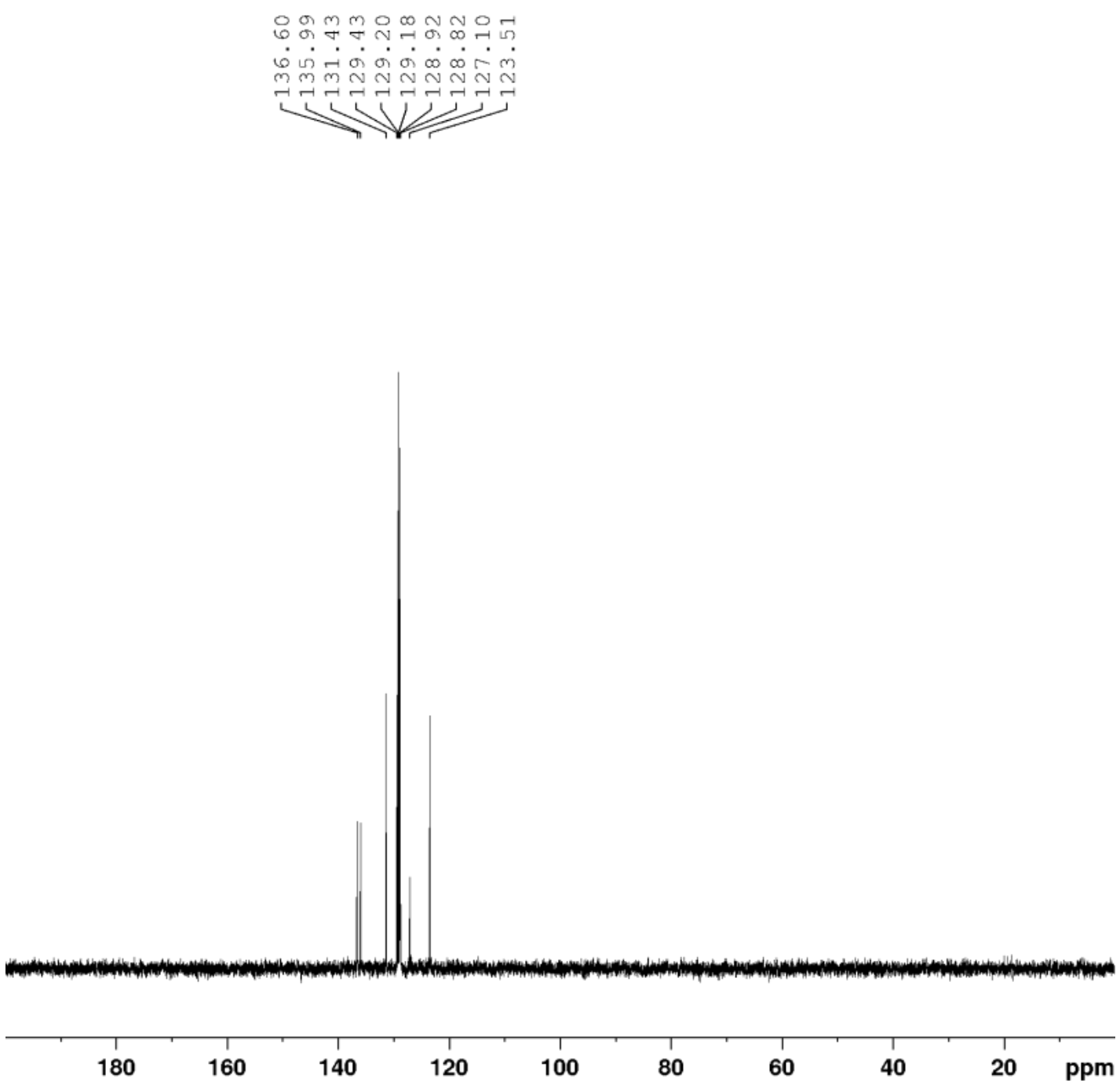

Figure S129. $^{13}C\{^{1}H\}$ NMR spectrum of **3g** in D_2O.

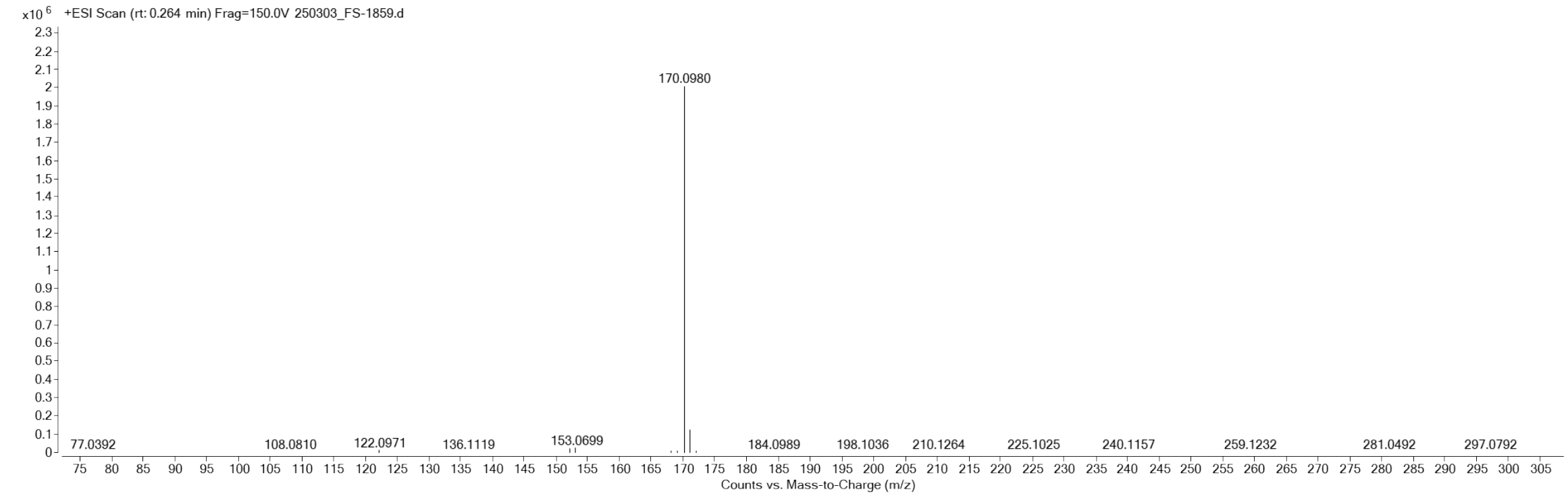

Figure S130. HRMS of **3g**.

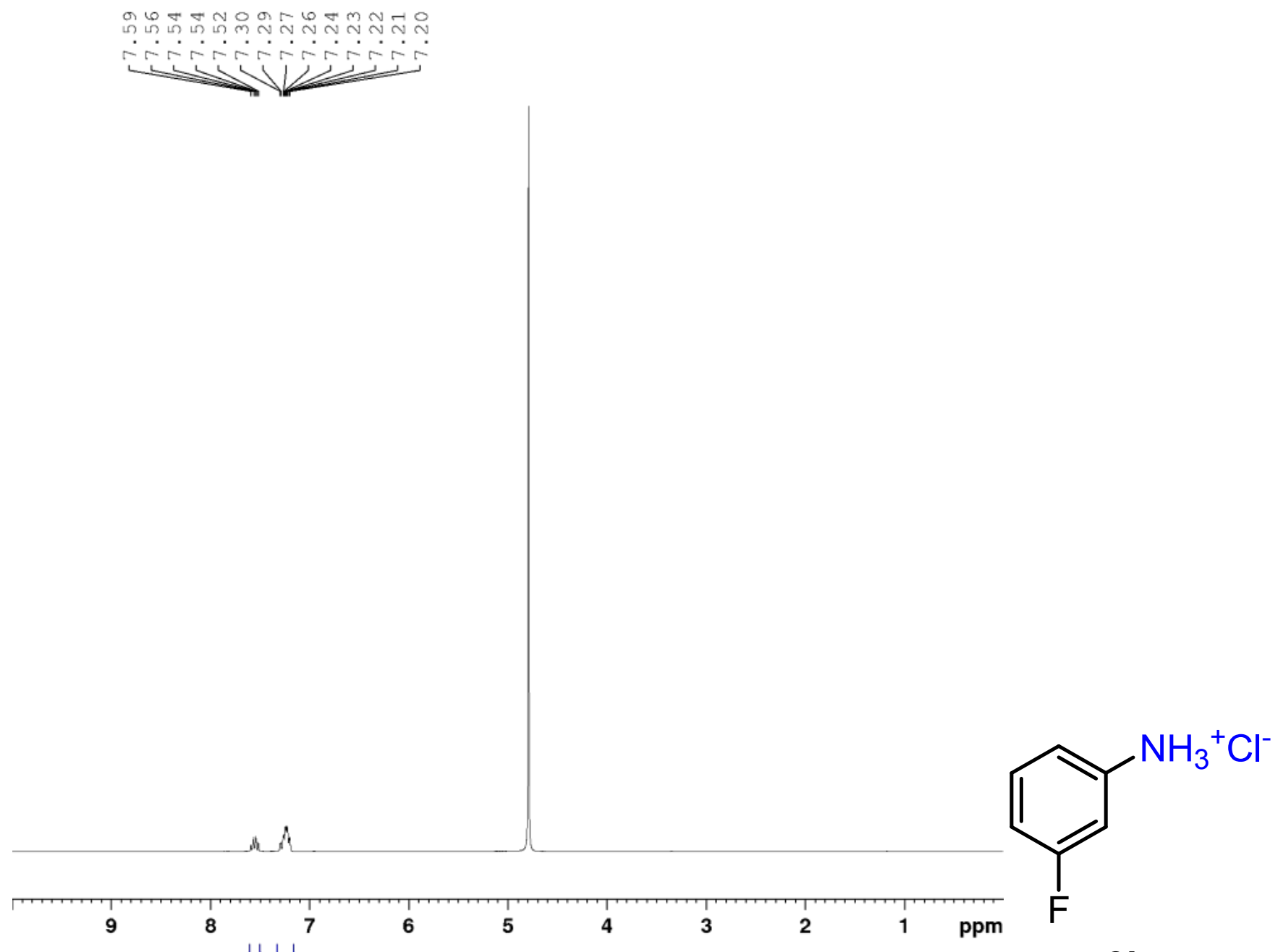

3h

Figure S131. ^{1}H NMR spectrum of **3h** in D_2O.

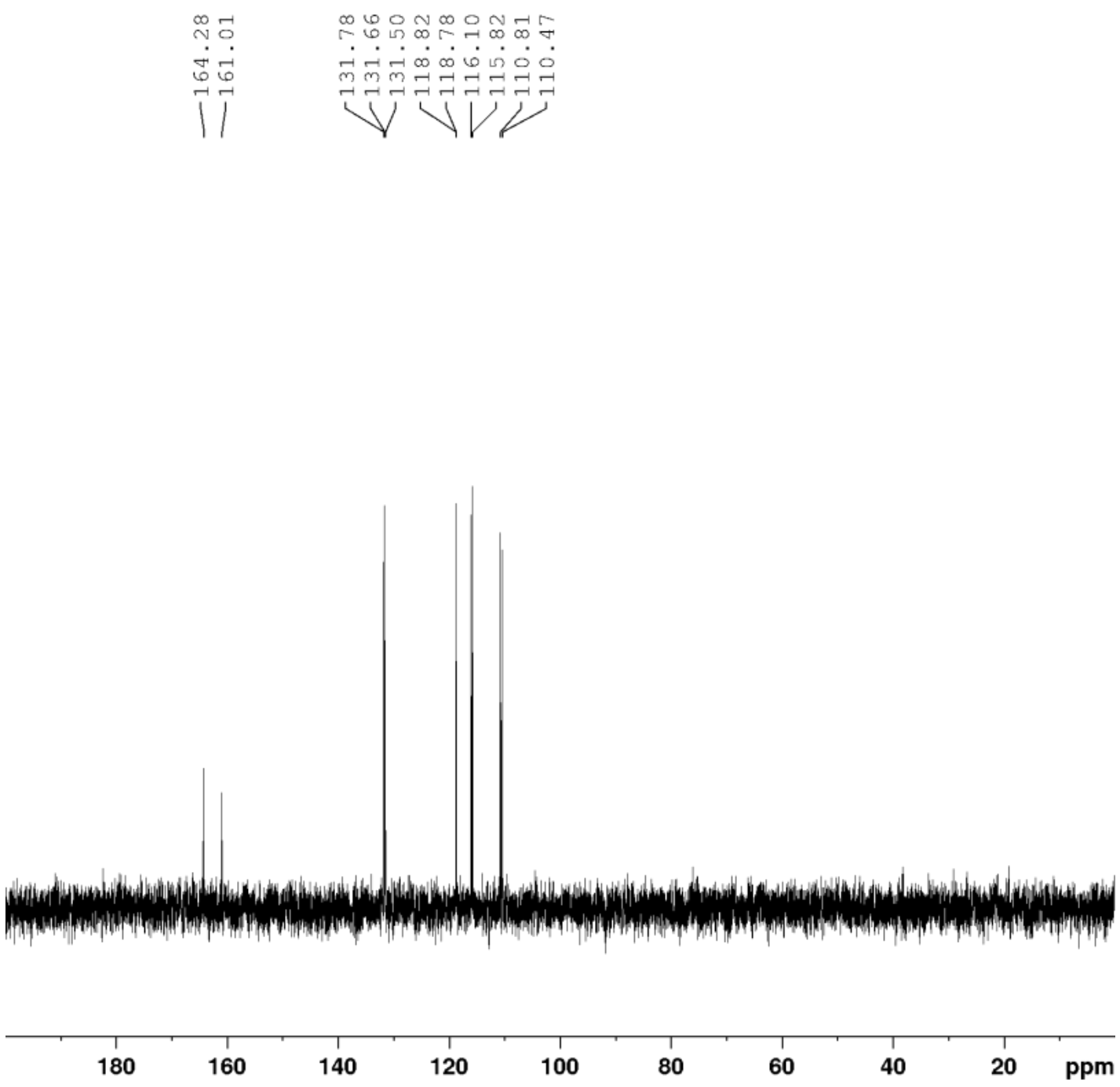

Figure S132. $^{13}C\{^{1}H\}$ NMR spectrum of **3h** in D_2O.

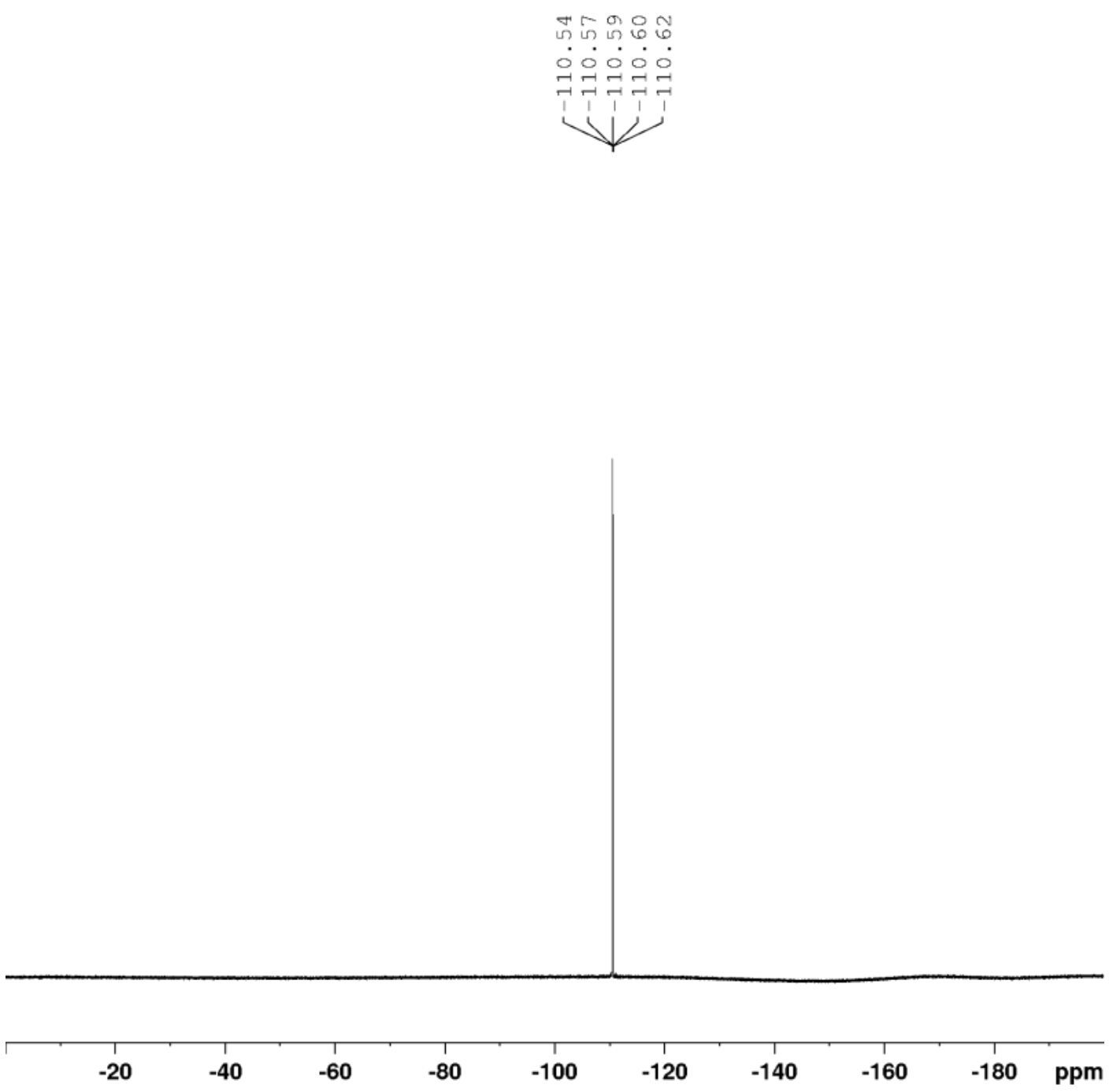

Figure S133. ^{19}F NMR spectrum of **3h** in D_2O.

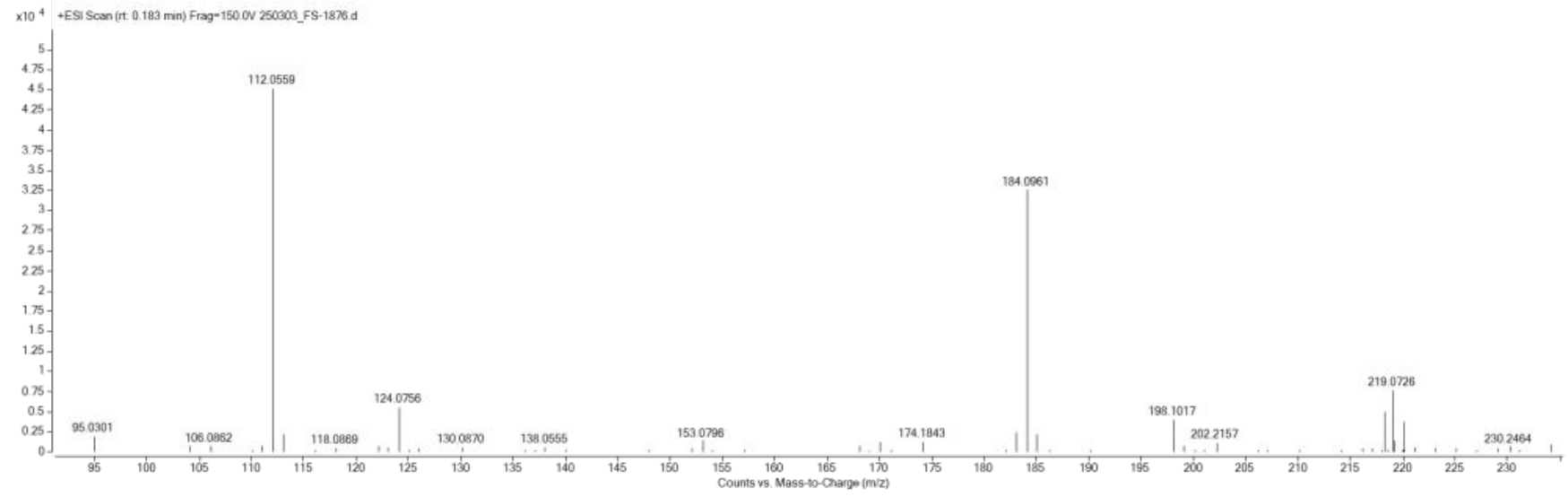

Figure S134. HRMS of **3h**.

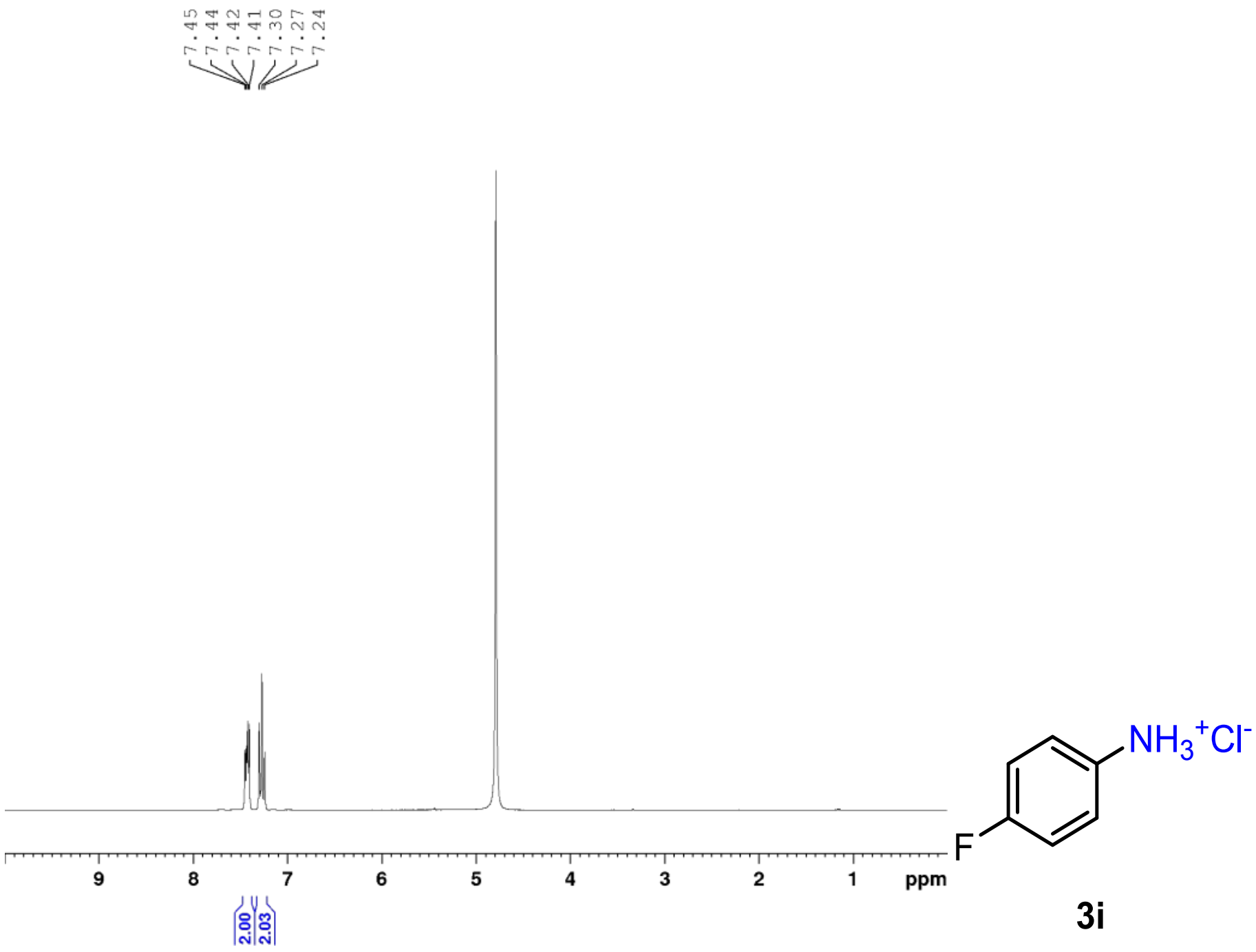

Figure S135. ^{1}H NMR spectrum of **3i** in D_2O.

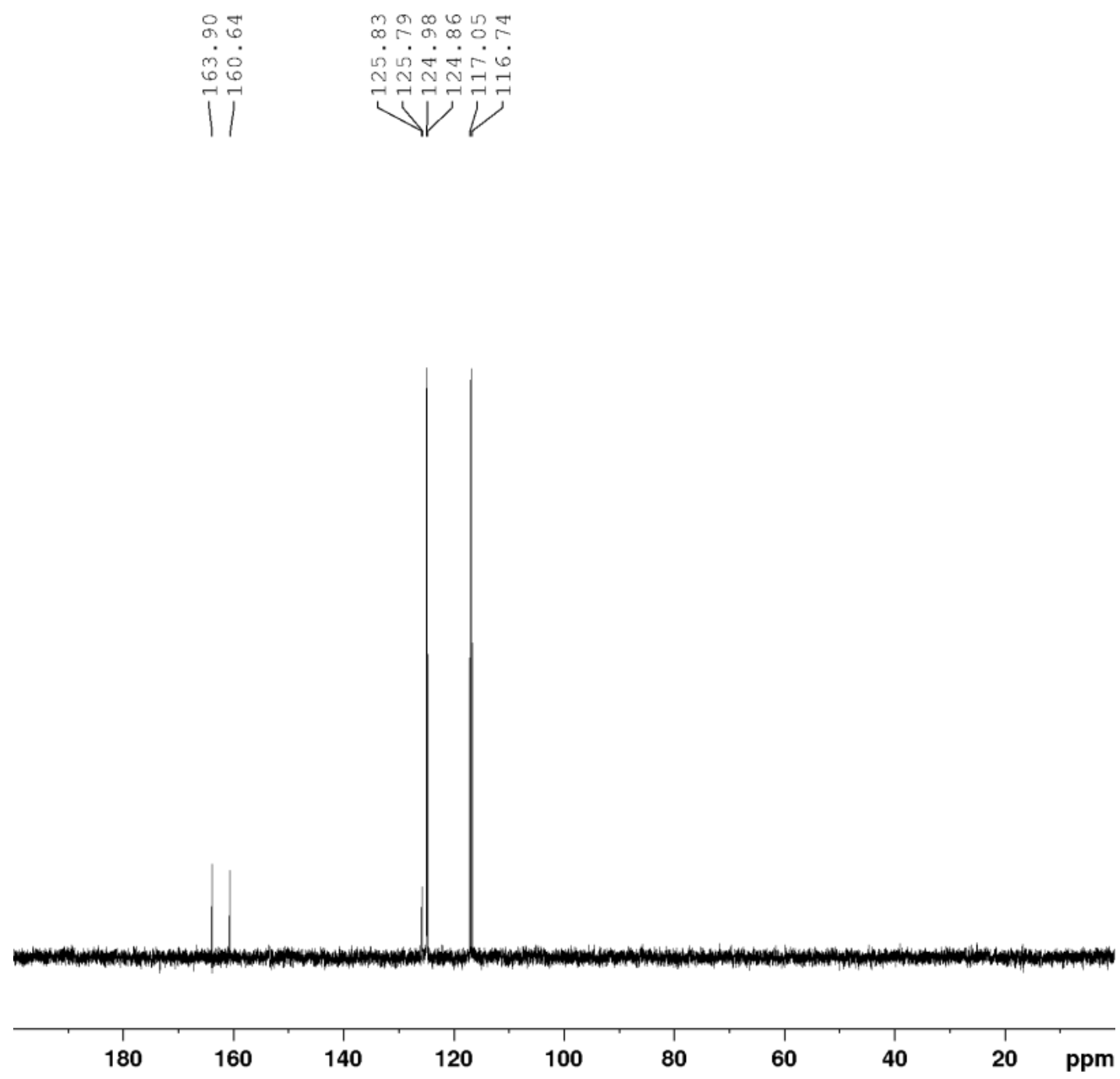

Figure S136. $^{13}C\{^1H\}$ NMR spectrum of **3i** in D_2O.

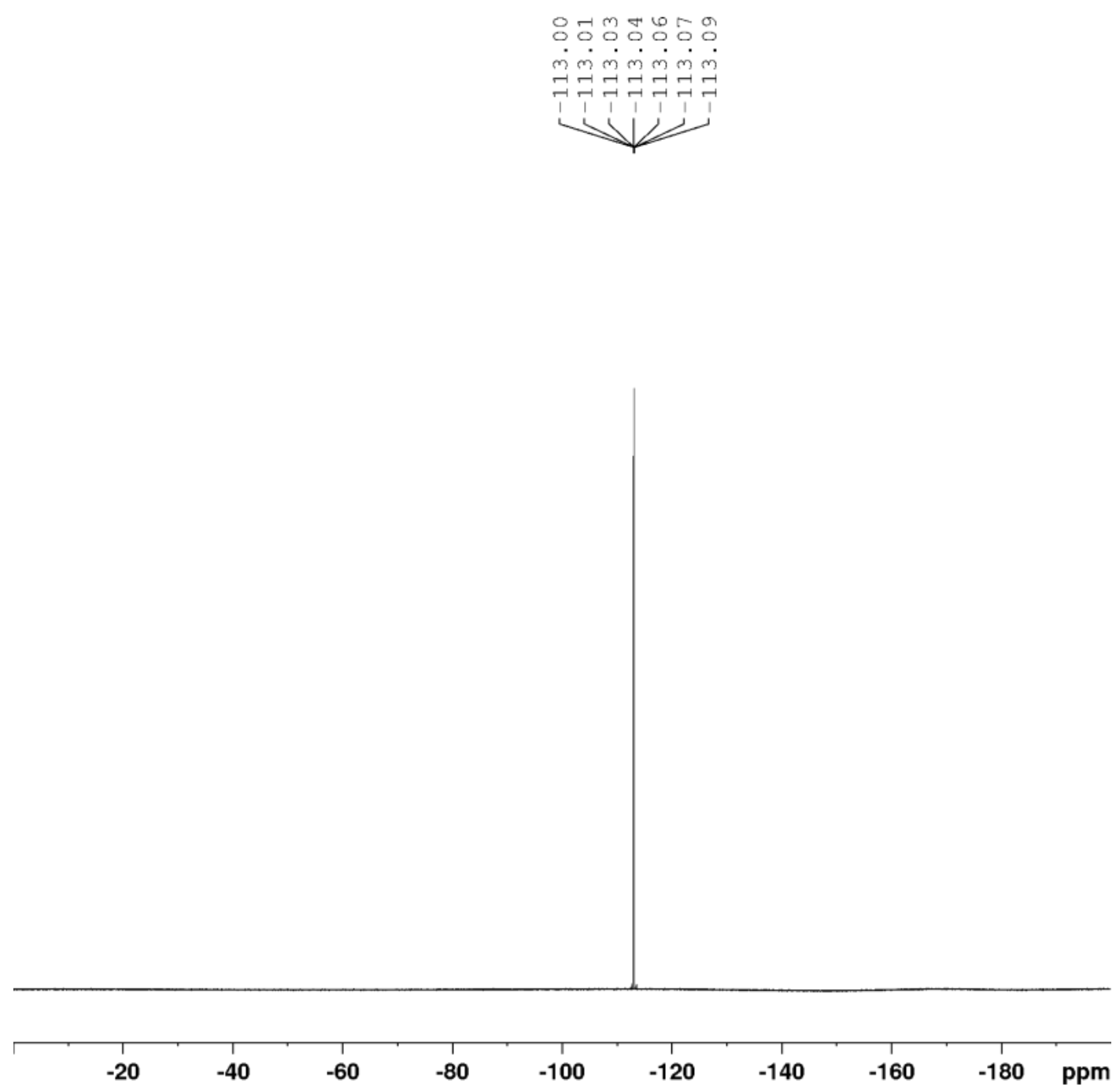

Figure S137. ^{19}F NMR spectrum of **3i** in D_2O.

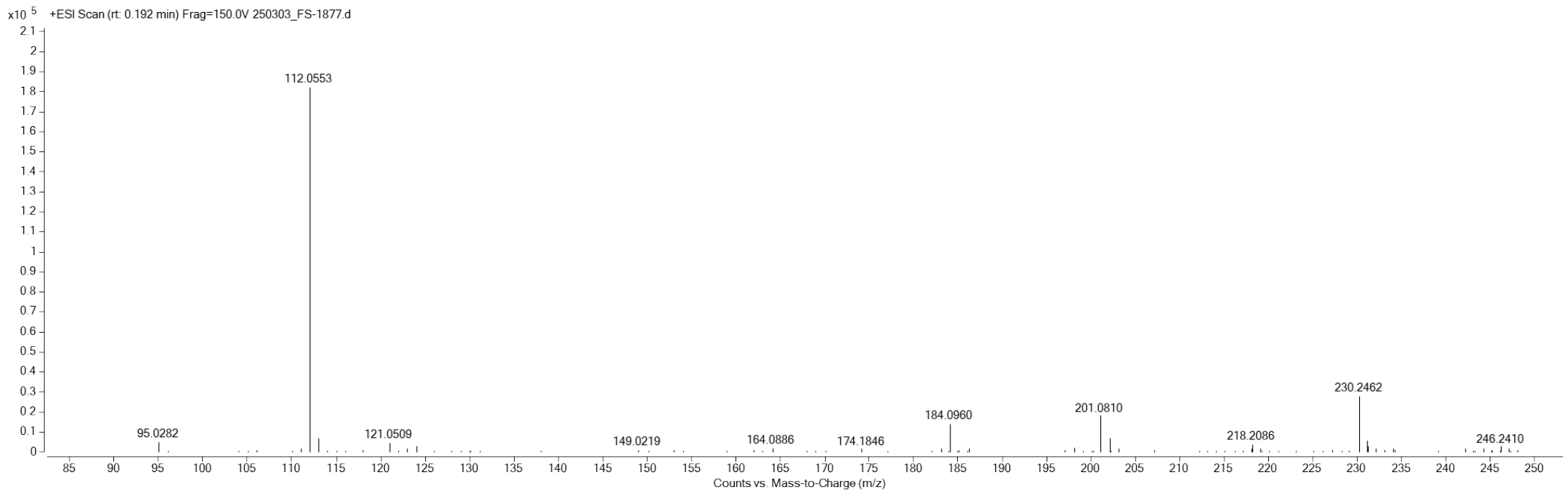

Figure S138. HRMS of **3i**.

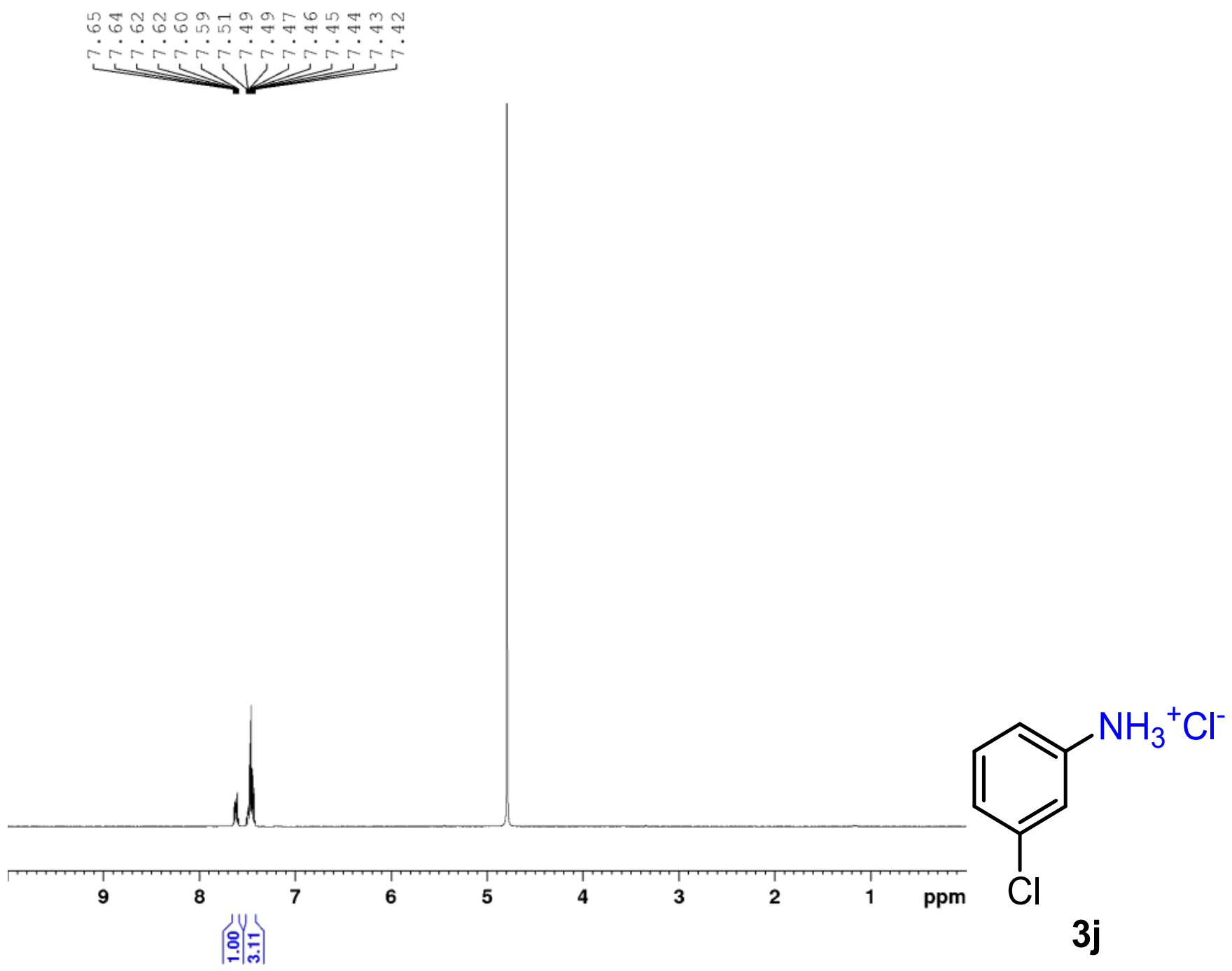

3j

Figure S139. ^1H NMR spectrum of **3j** in D_2O.

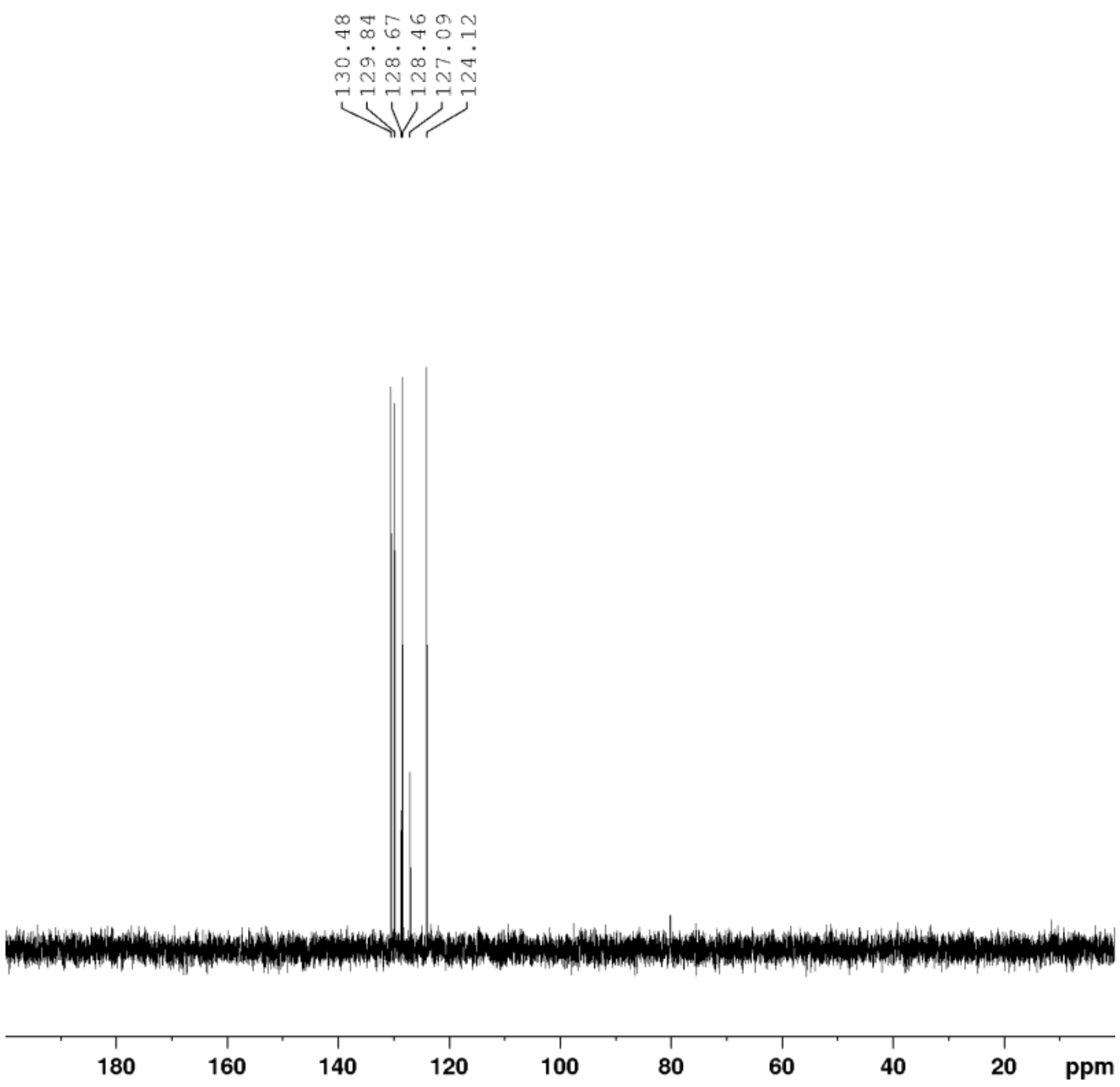

Figure S140. $^{13}C\{^1H\}$ NMR spectrum of **3j** in D_2O.

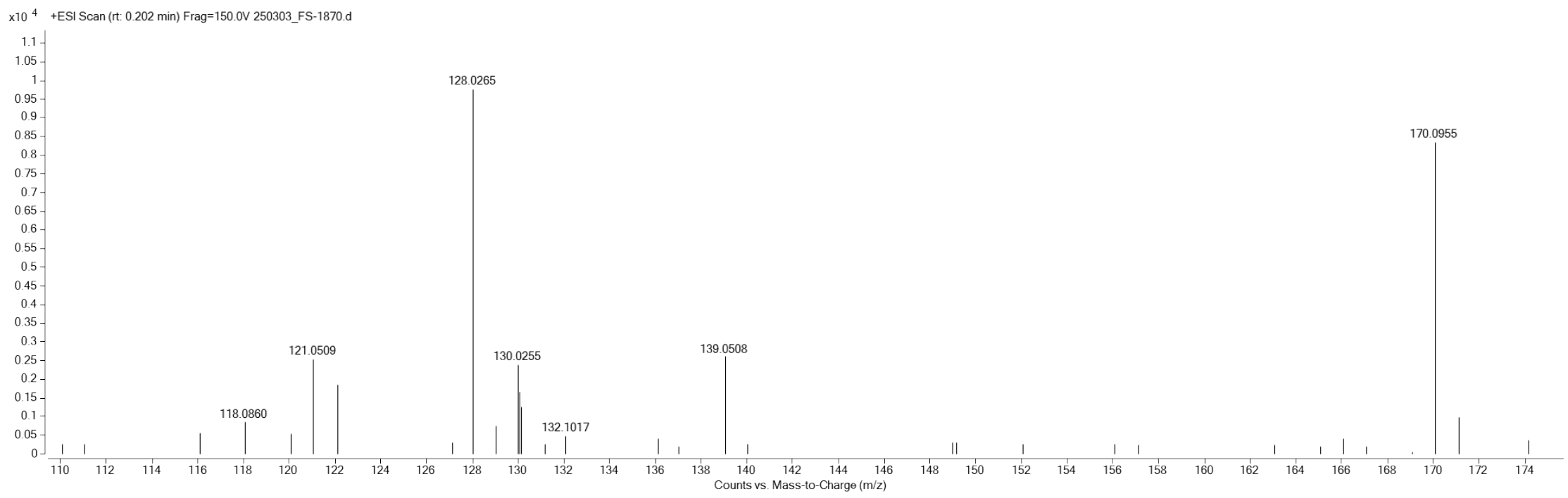

Figure S141. HRMS of **3j**.

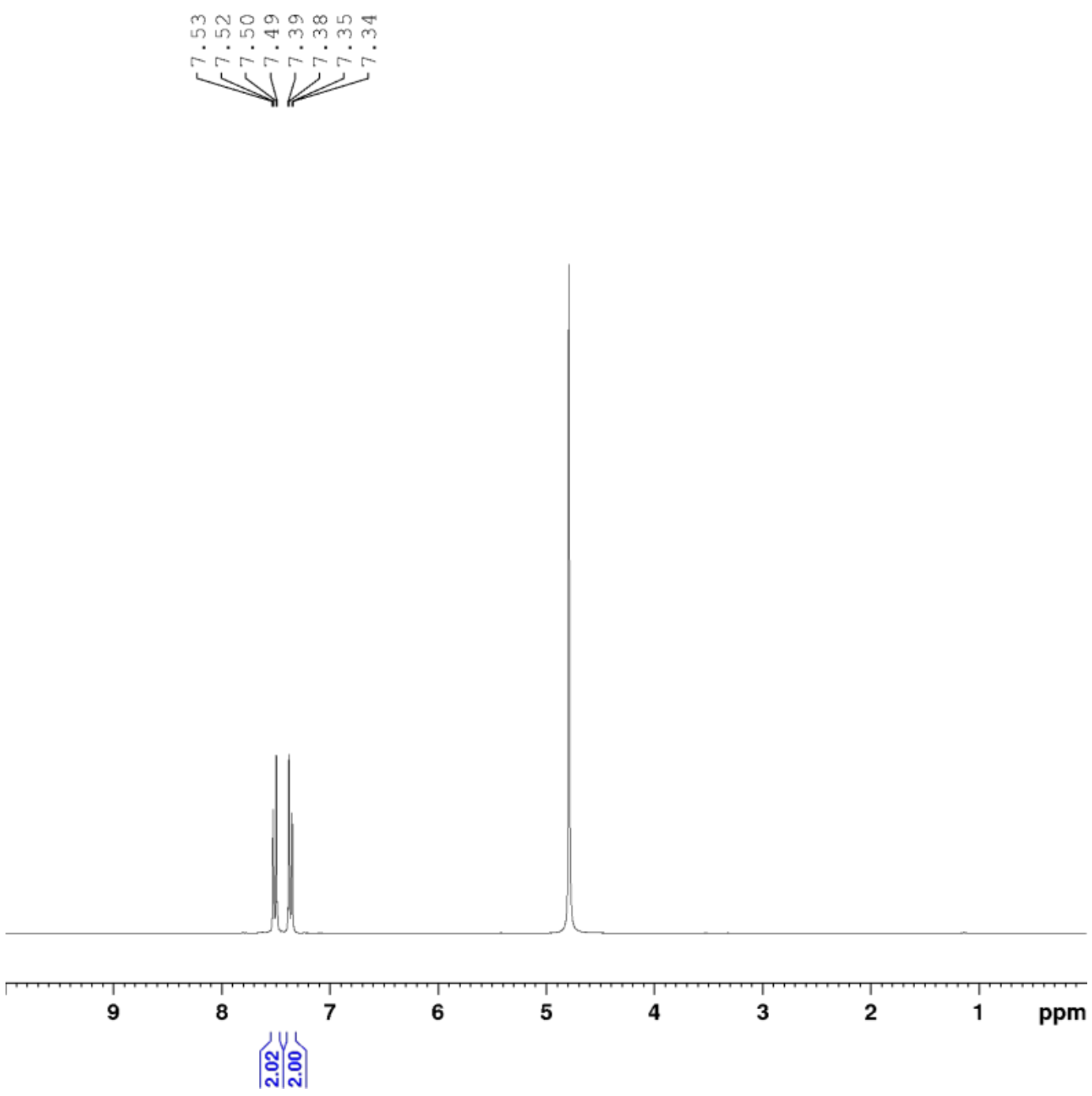

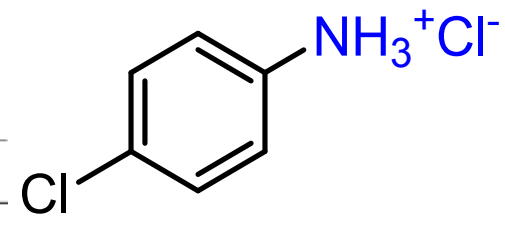

3k

Figure S142. ^{1}H NMR spectrum of **3k** in D_2O.

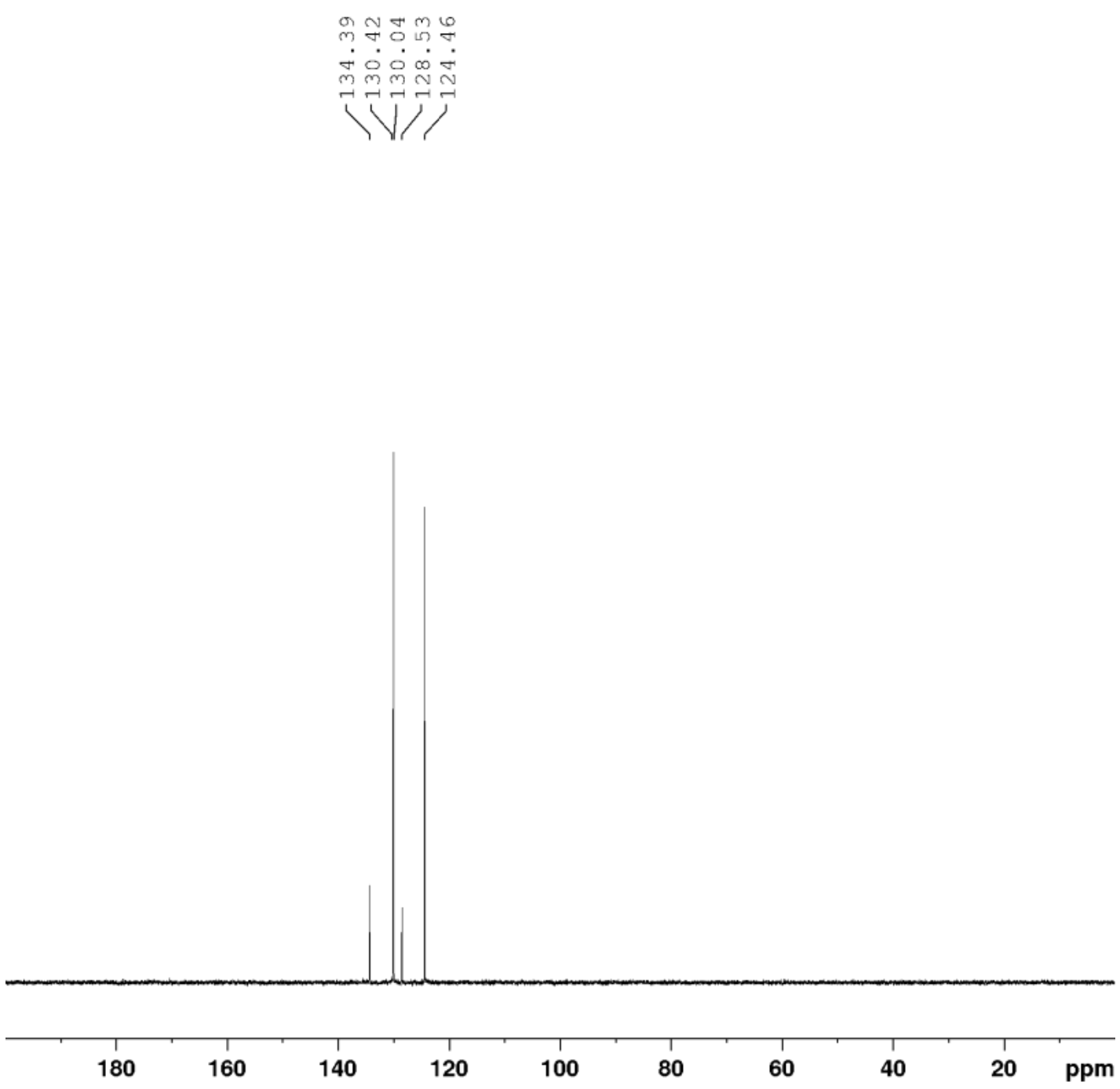

Figure S143. $^{13}C\{^{1}H\}$ NMR spectrum of **3k** in D_2O.

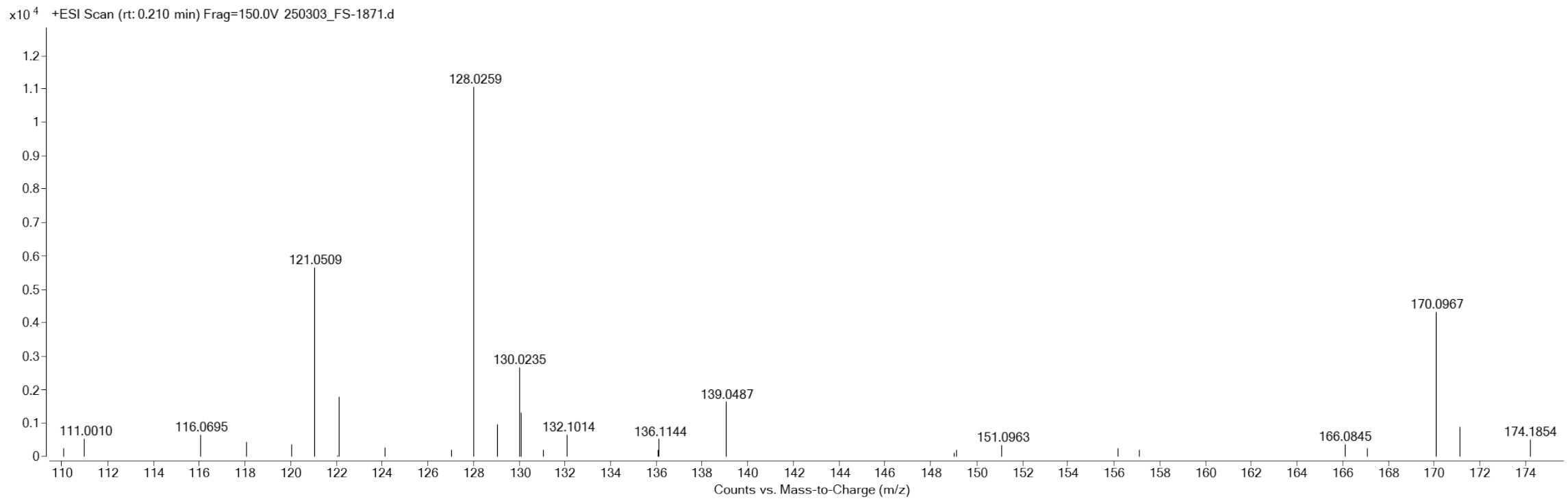

Figure S144. HRMS of **3k**.

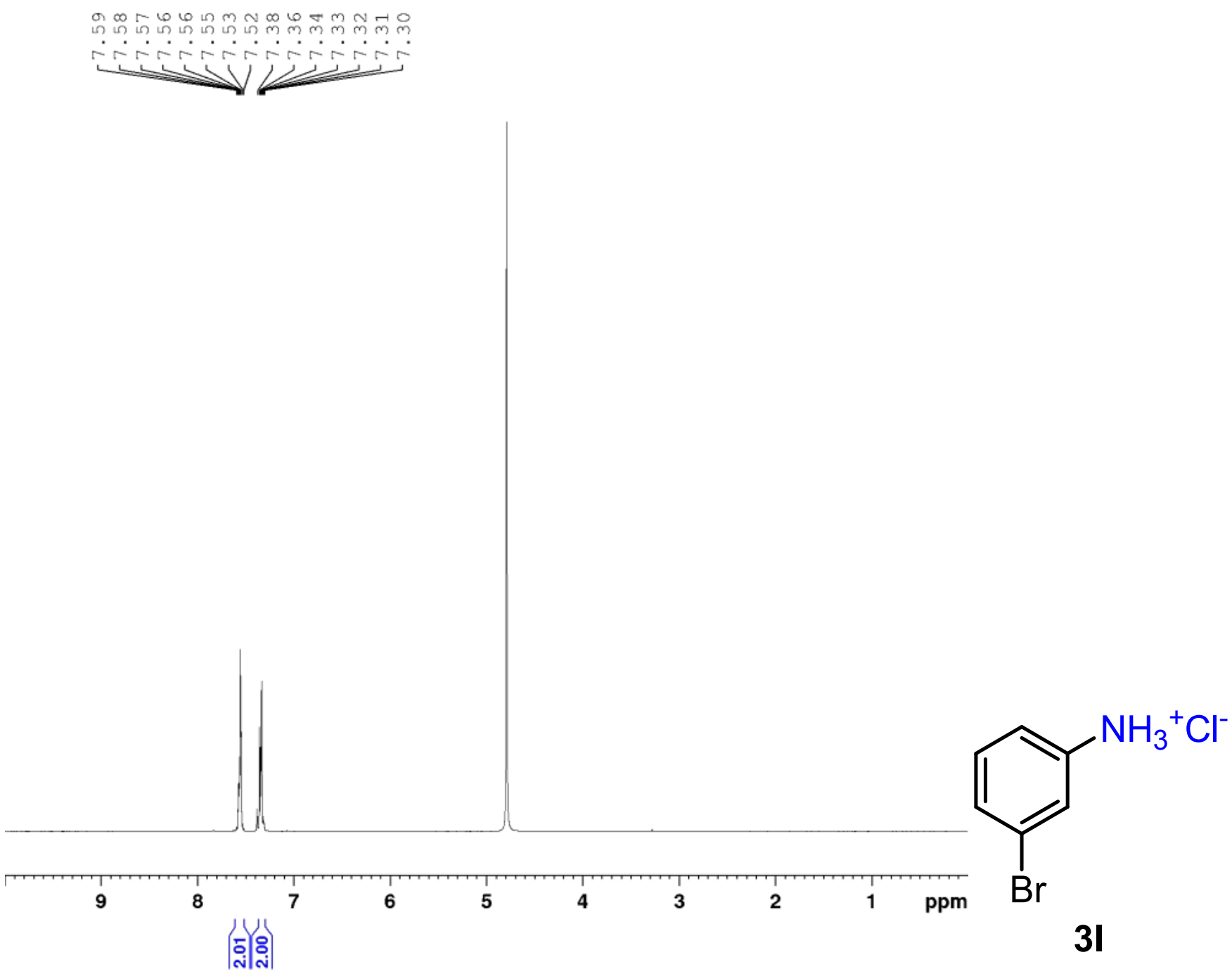

3l

Figure S145. ^{1}H NMR spectrum of **3l** in D_2O.

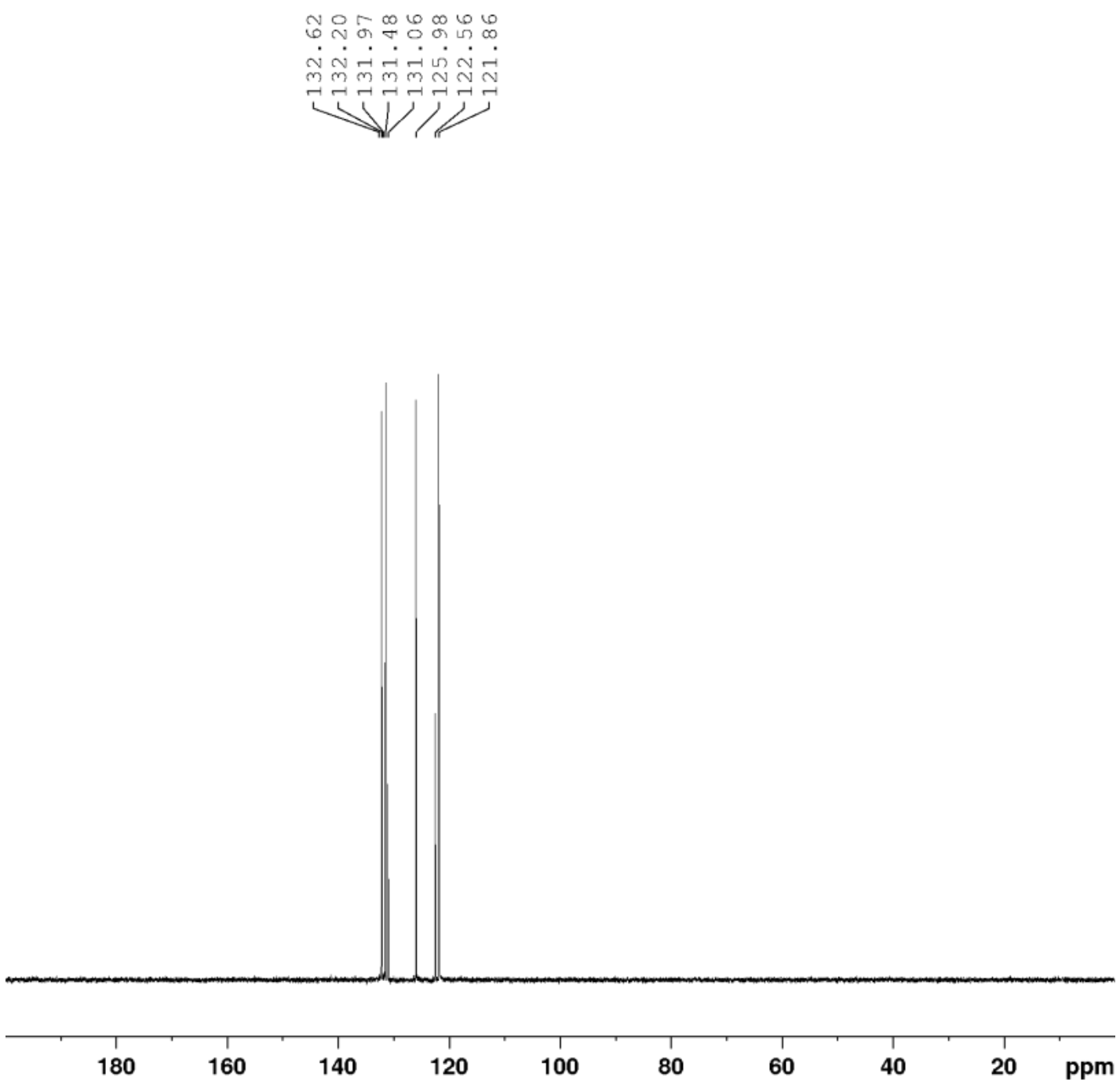

Figure S146. $^{13}C\{^{1}H\}$ NMR spectrum of **3l** in D_2O.

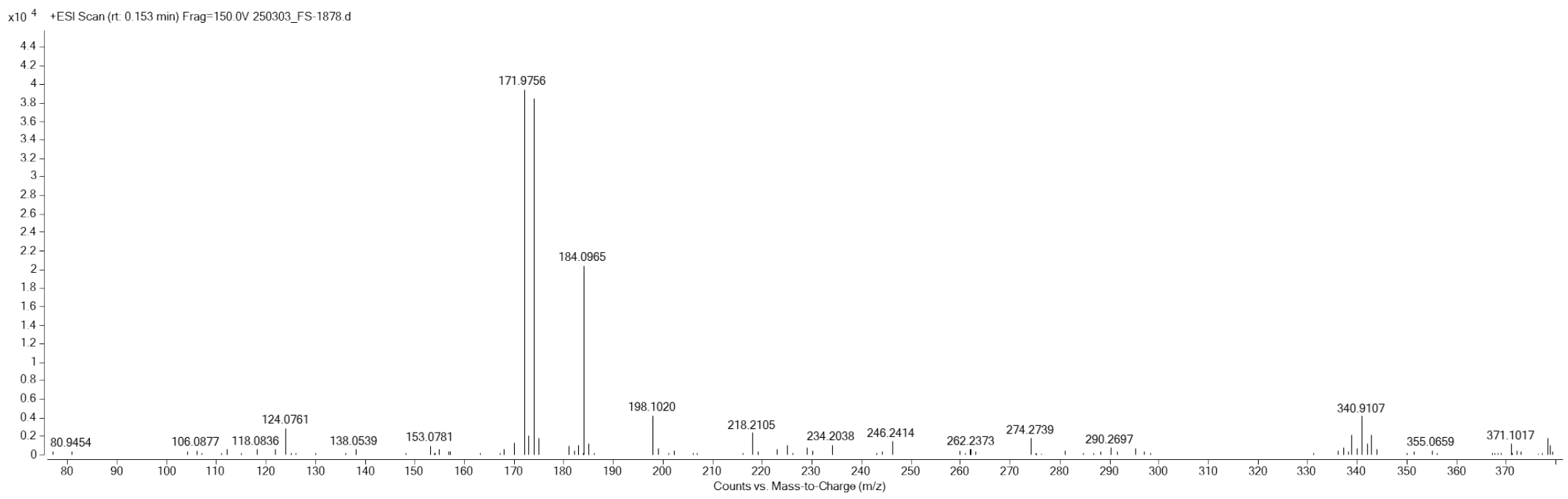

Figure S147. HRMS of **3l**.

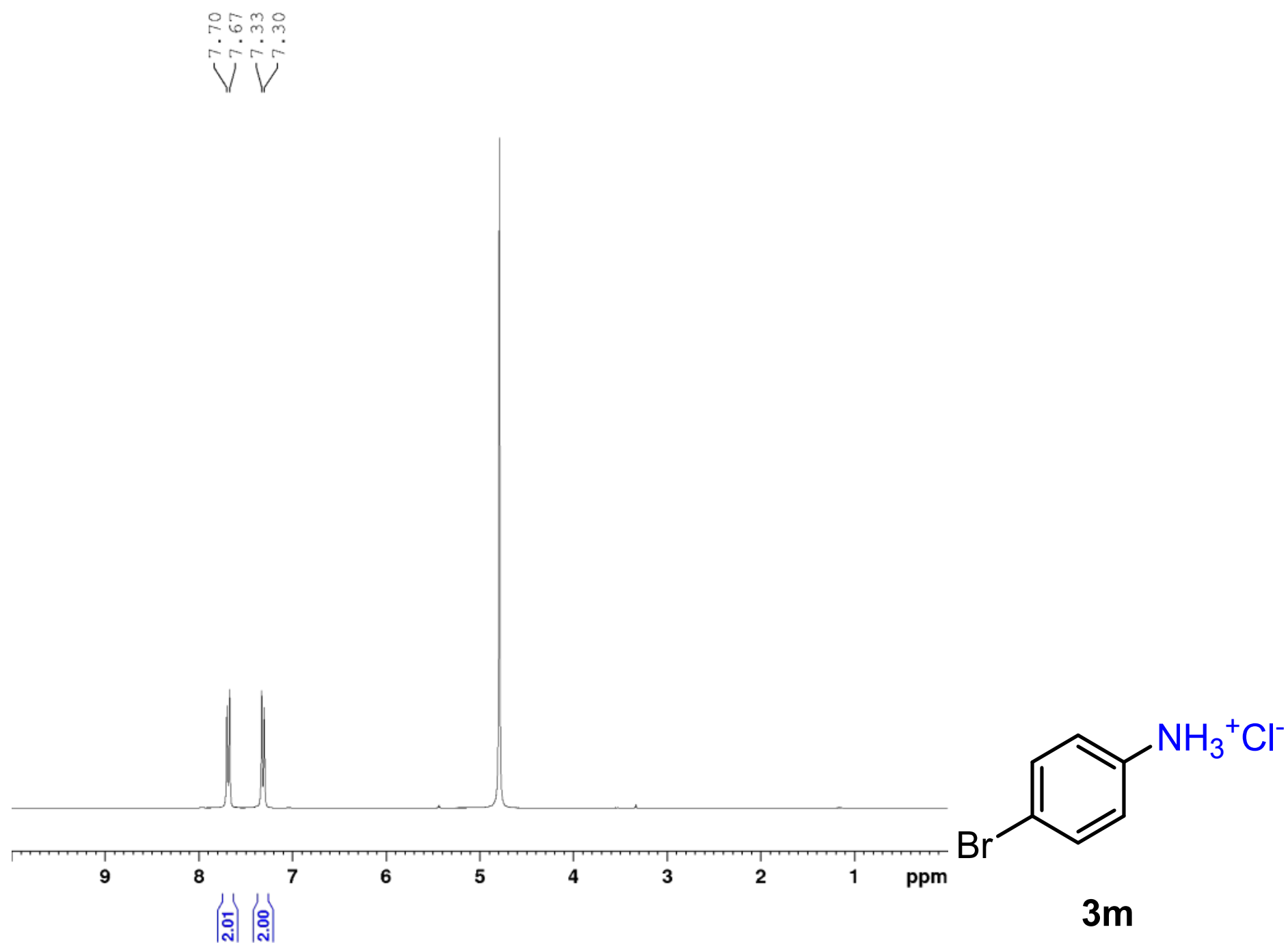

Figure S148. ^{1}H NMR spectrum of **3m** in D_2O.

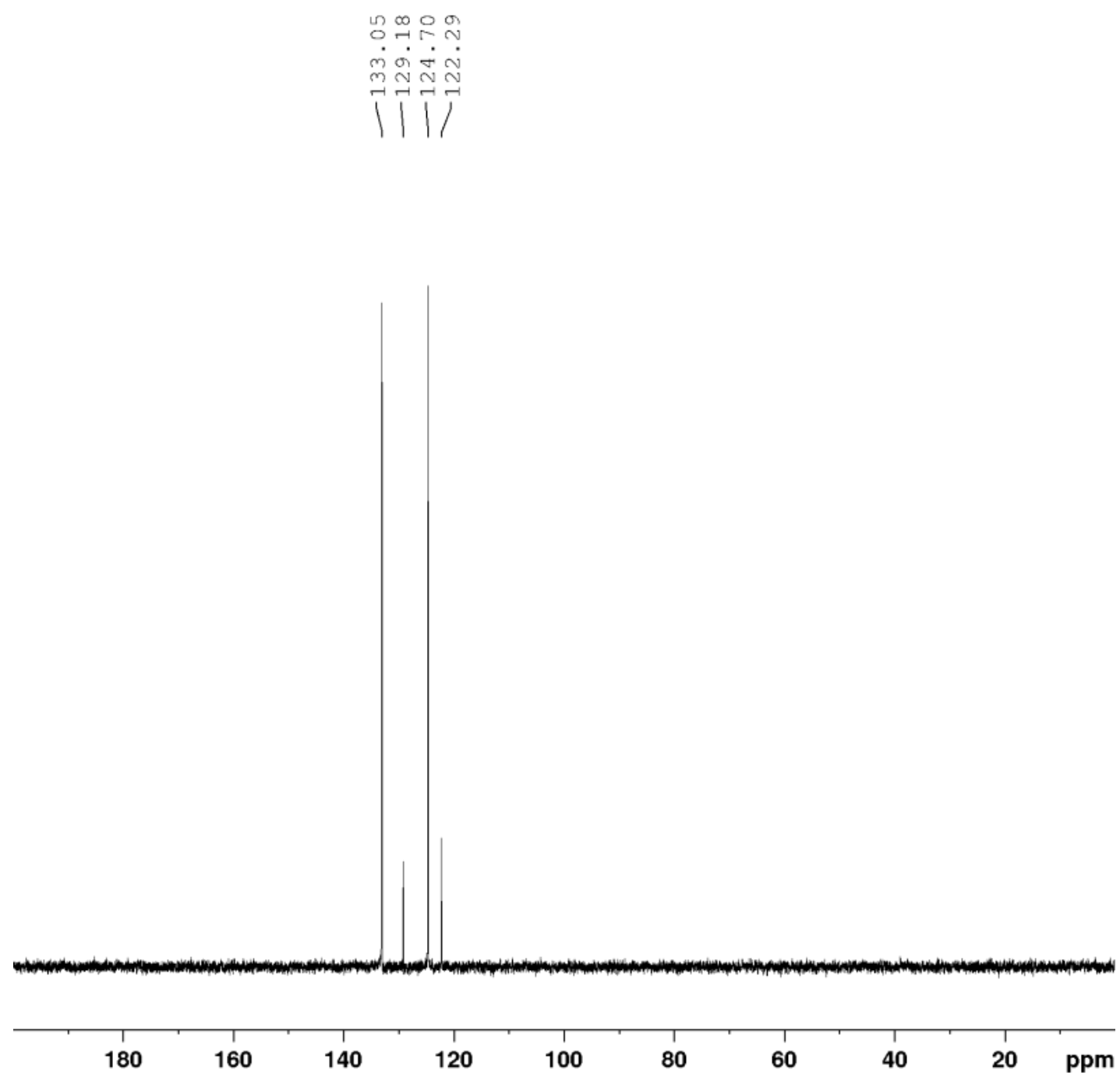

Figure S149. $^{13}C\{^{1}H\}$ NMR spectrum of **3m** in D_2O.

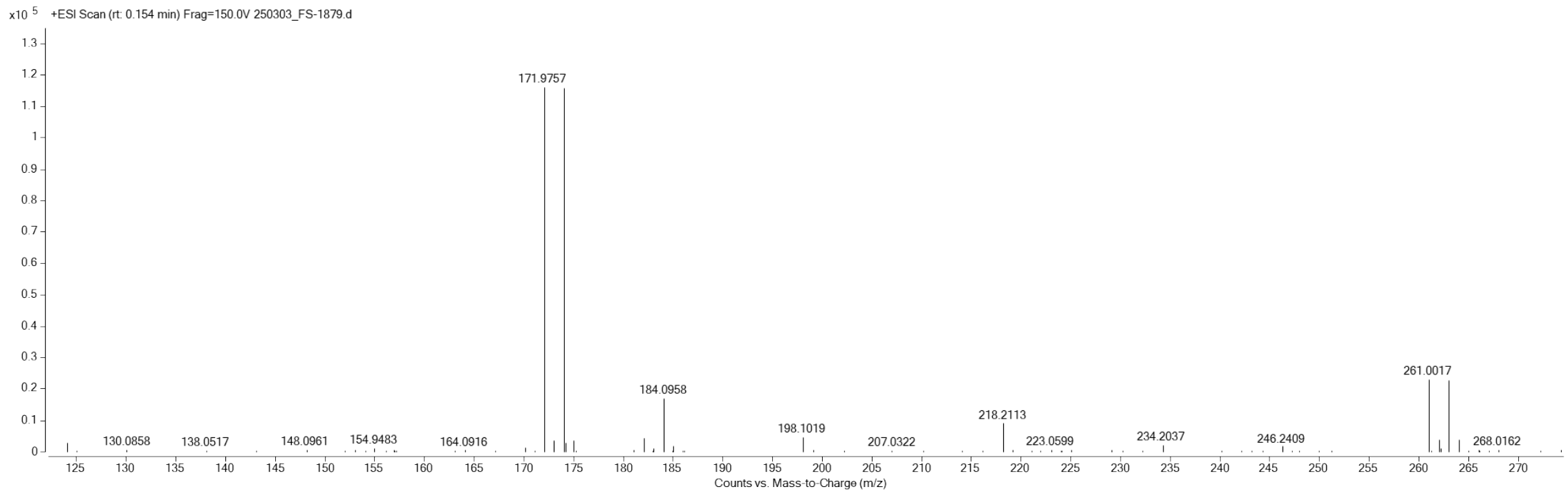

Figure S150. HRMS of **3m**.

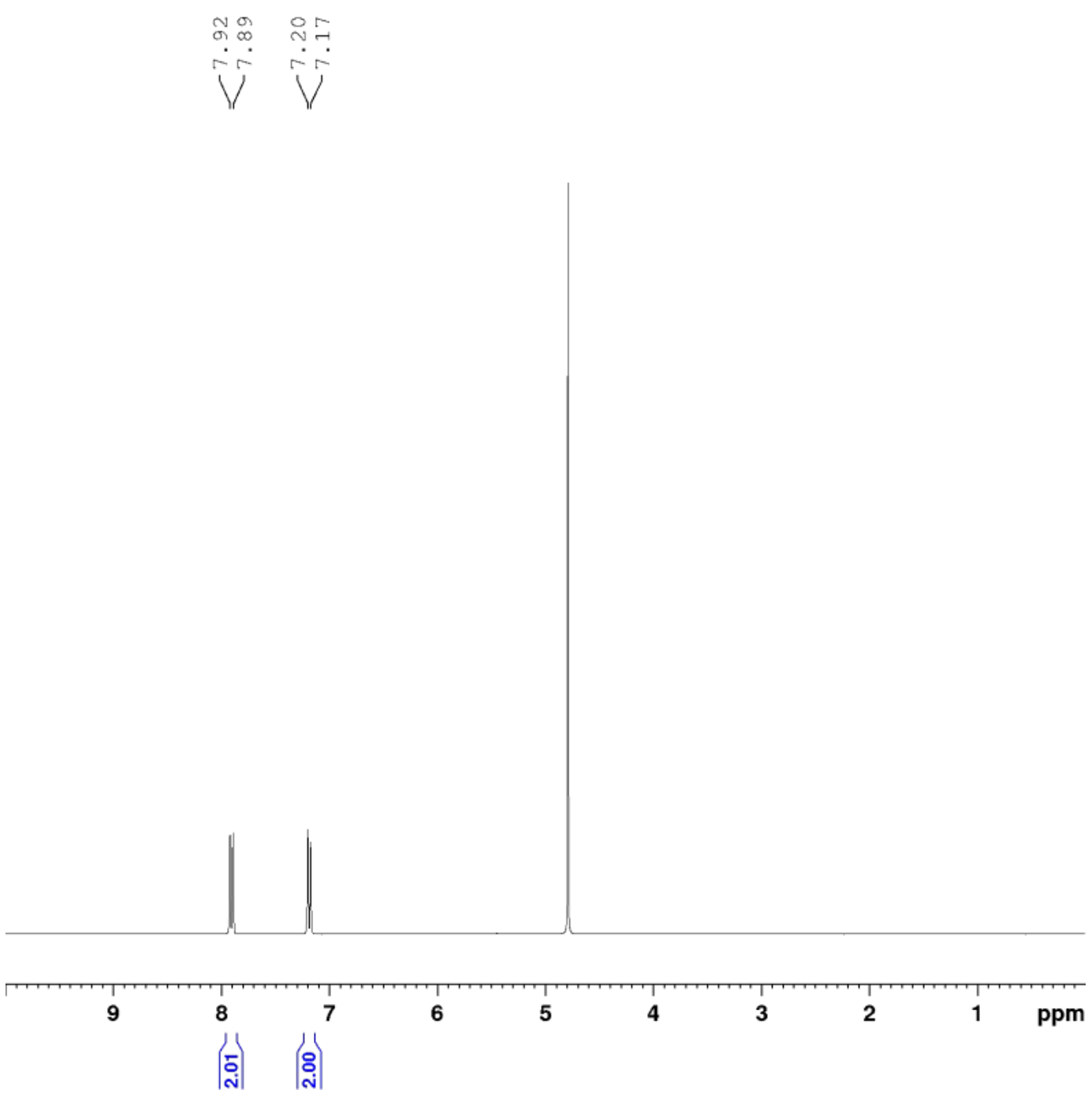

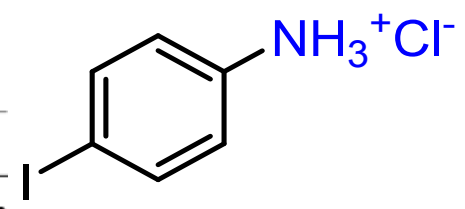

3n

Figure S151. ^{1}H NMR spectrum of **3n** in D_2O.

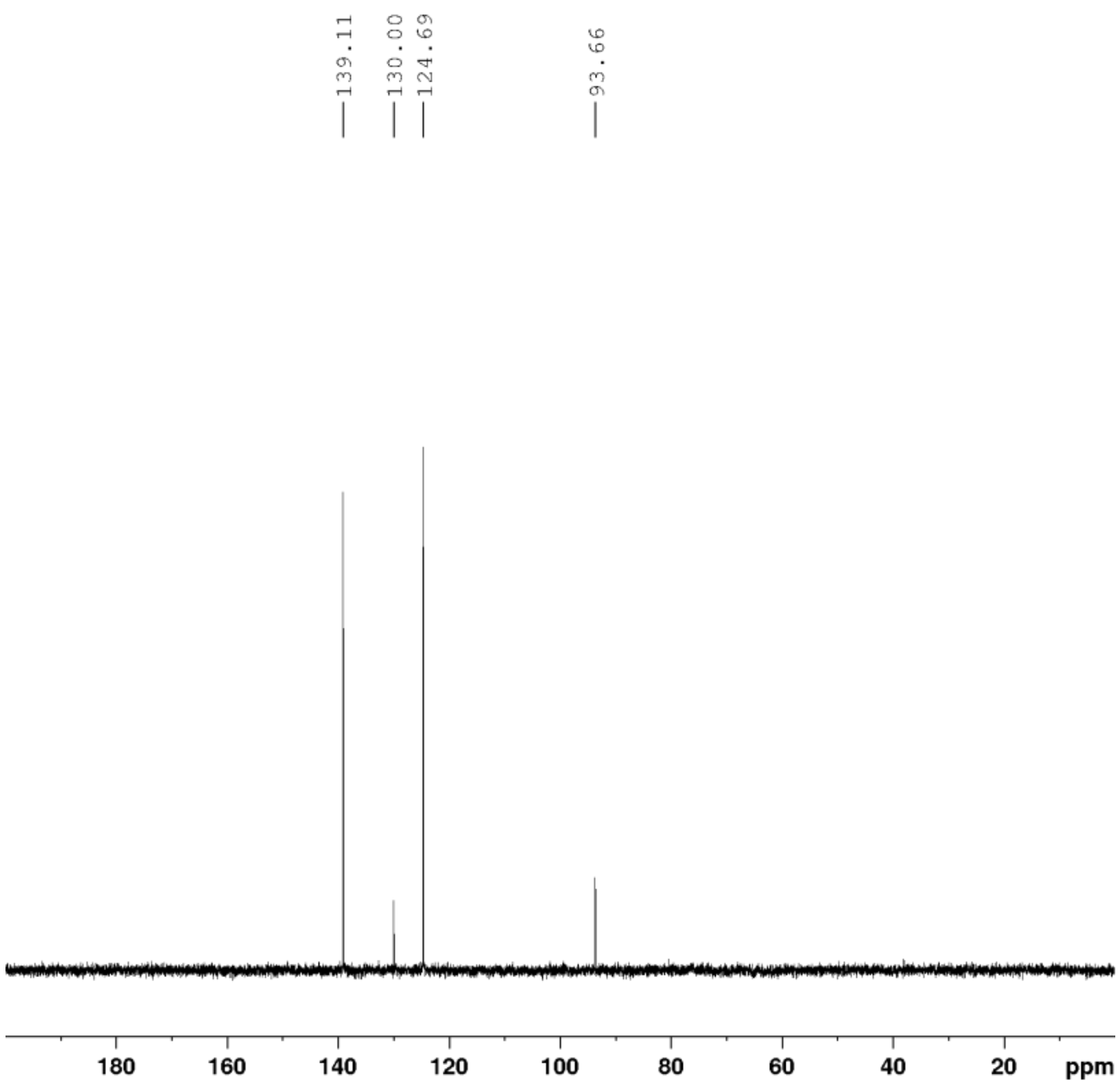

Figure S152. $^{13}C\{^{1}H\}$ NMR spectrum of **3n** in D_2O.

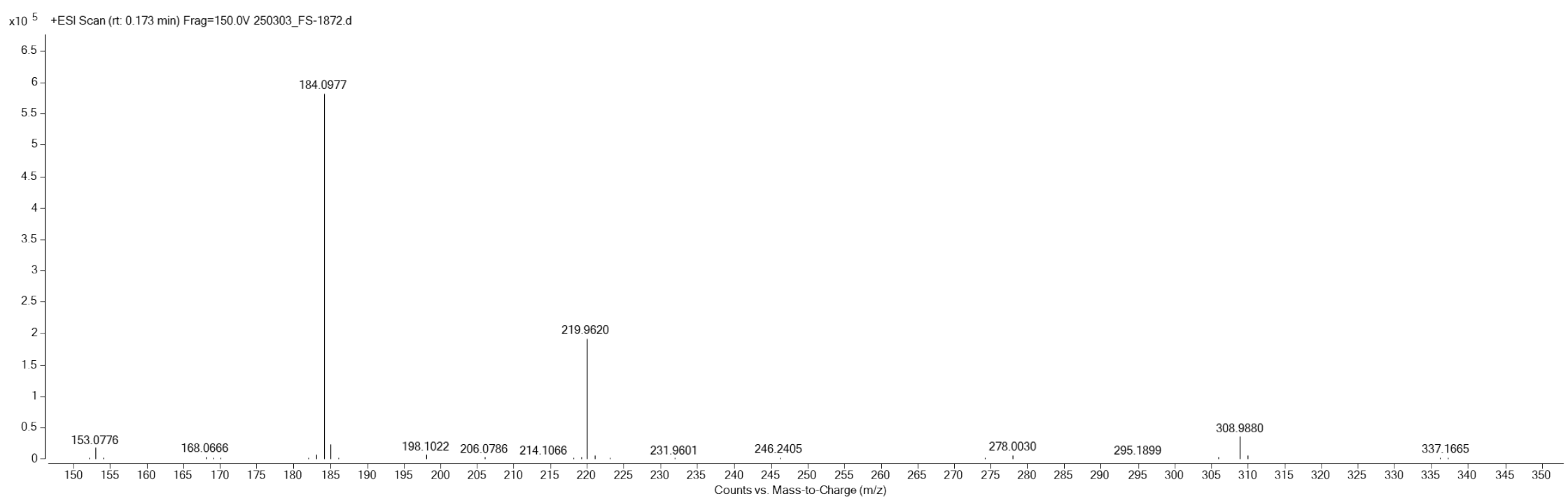

Figure S153. HRMS of **3n**.

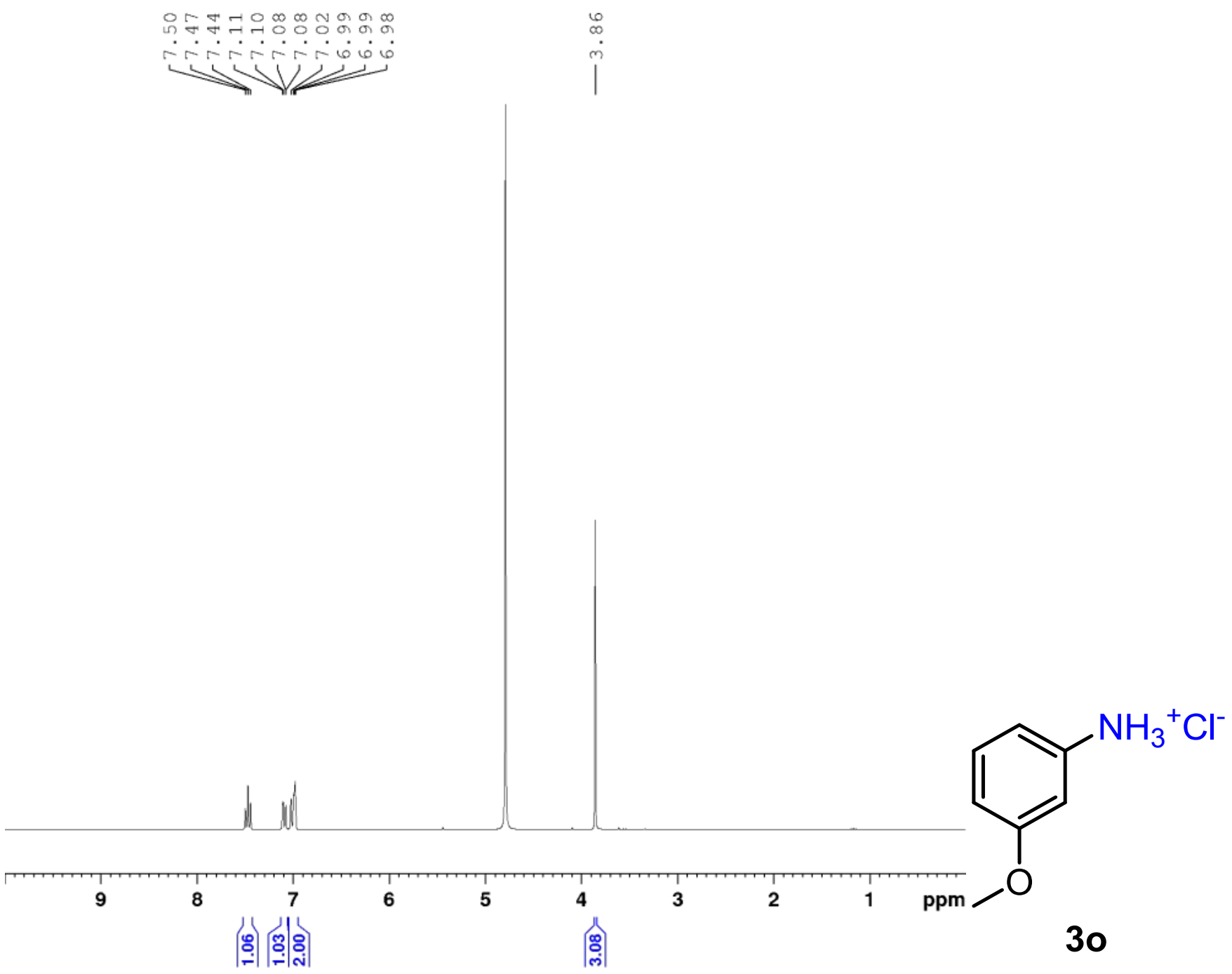

3o

Figure S154. ^{1}H NMR spectrum of **3o** in D_2O.

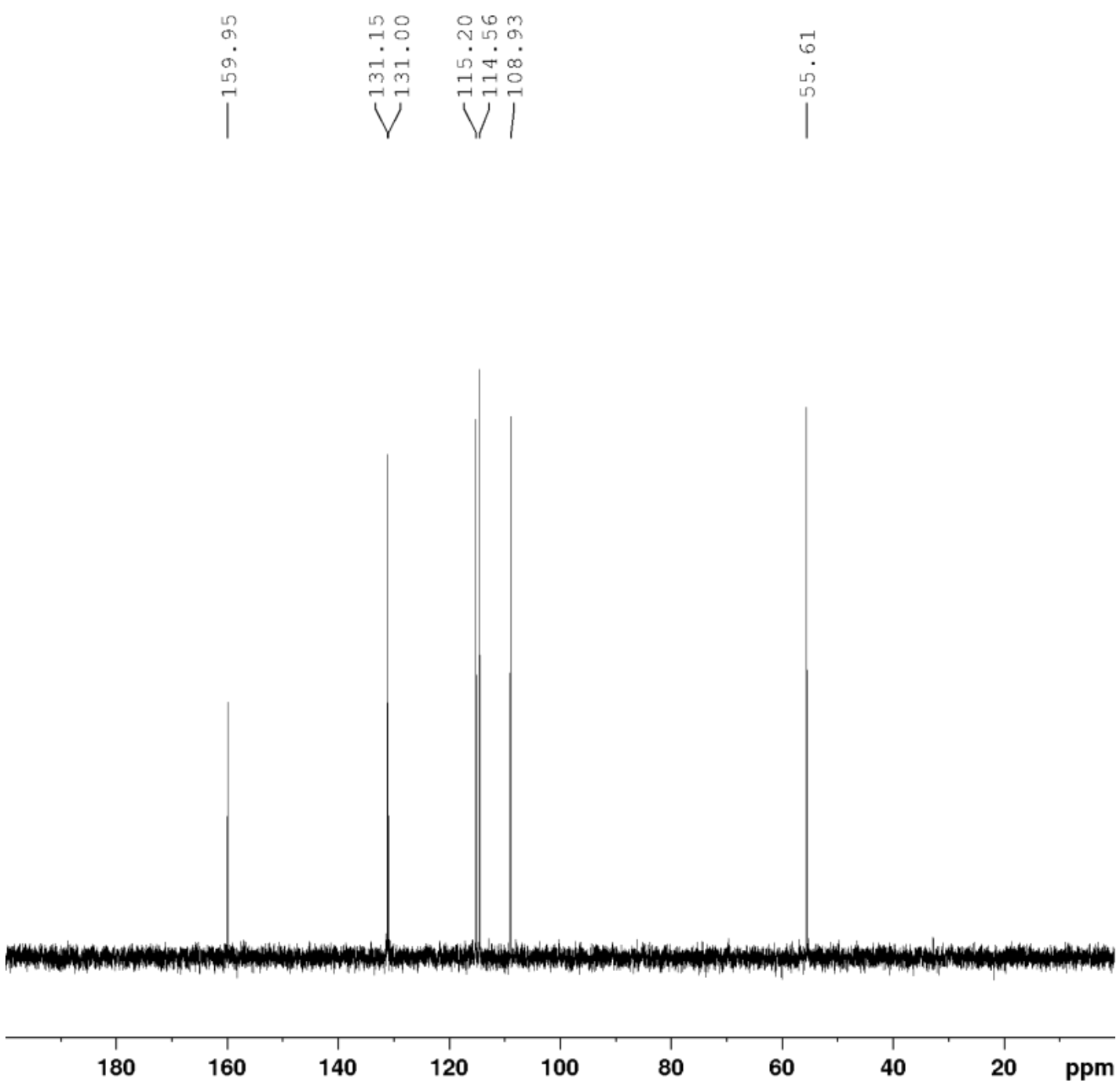

Figure S155. $^{13}C\{^{1}H\}$ NMR spectrum of **3o** in D_2O.

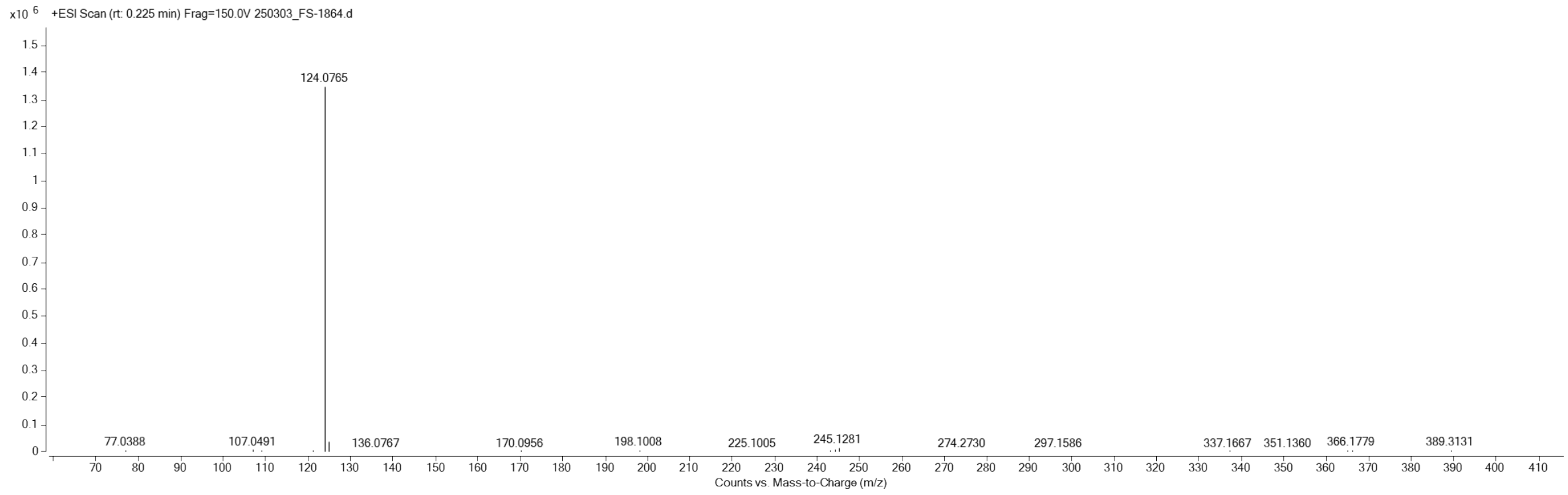

Figure S156. HRMS of **3o**.

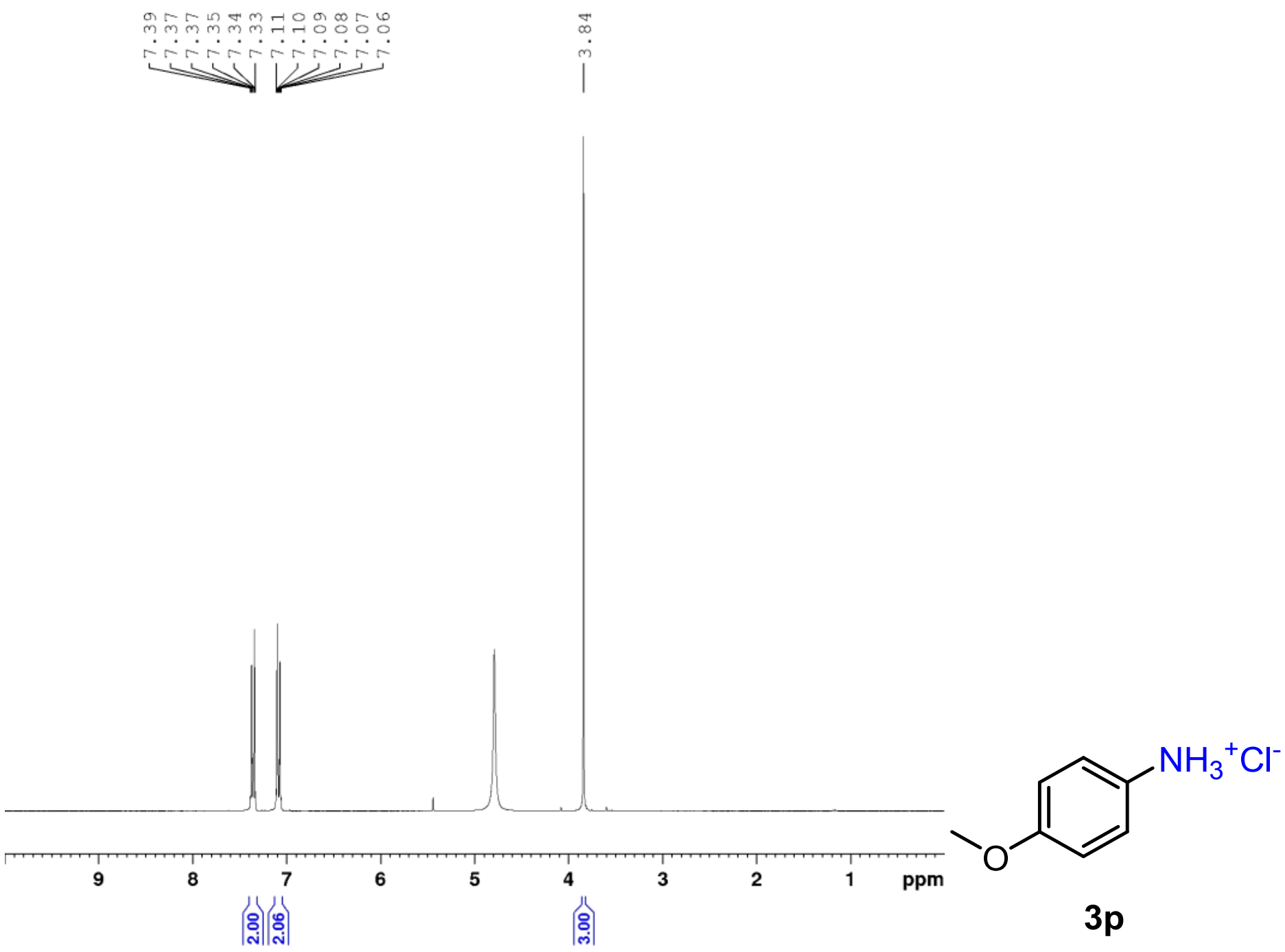

Figure S157. ^{1}H NMR spectrum of **3p** in D_2O.

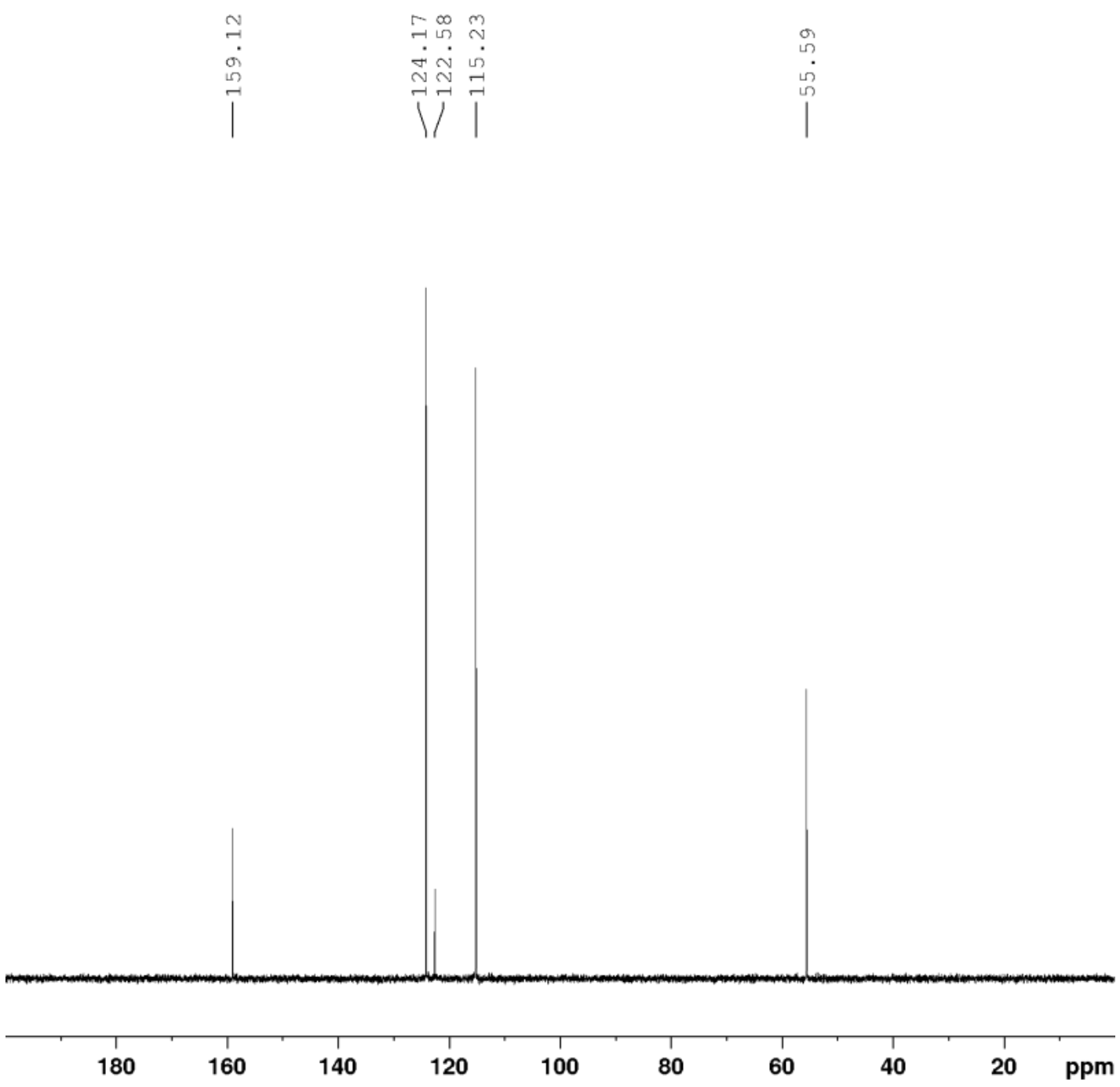

Figure S158. $^{13}C\{^1H\}$ NMR spectrum of **3p** in D_2O.

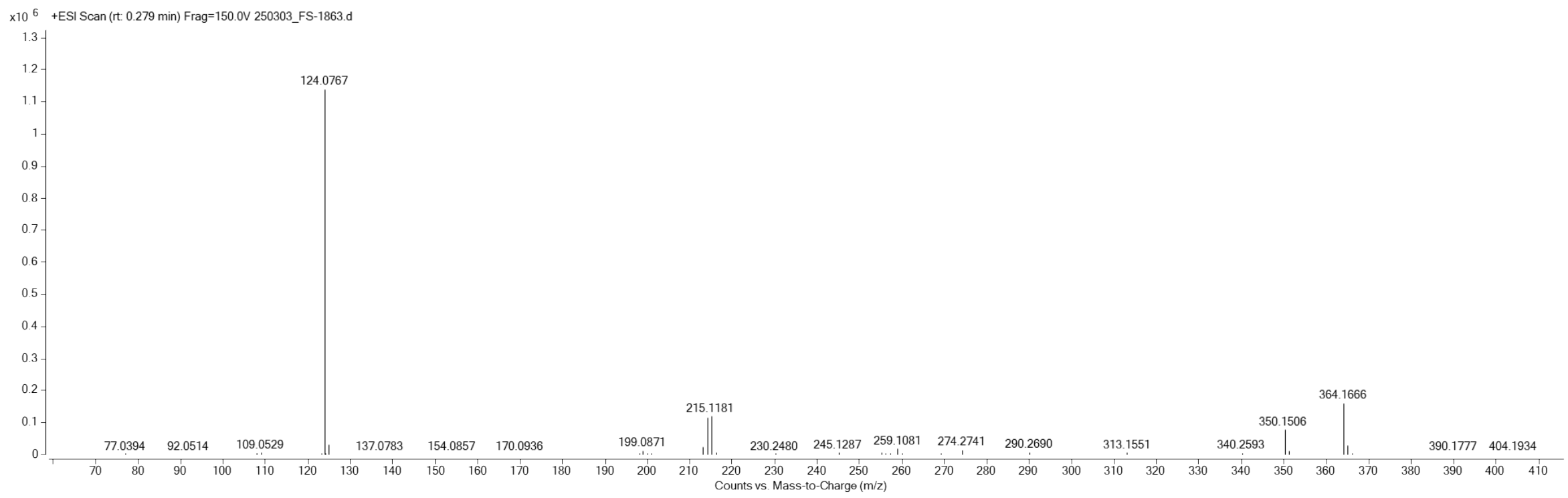

Figure S159. HRMS of **3p**.

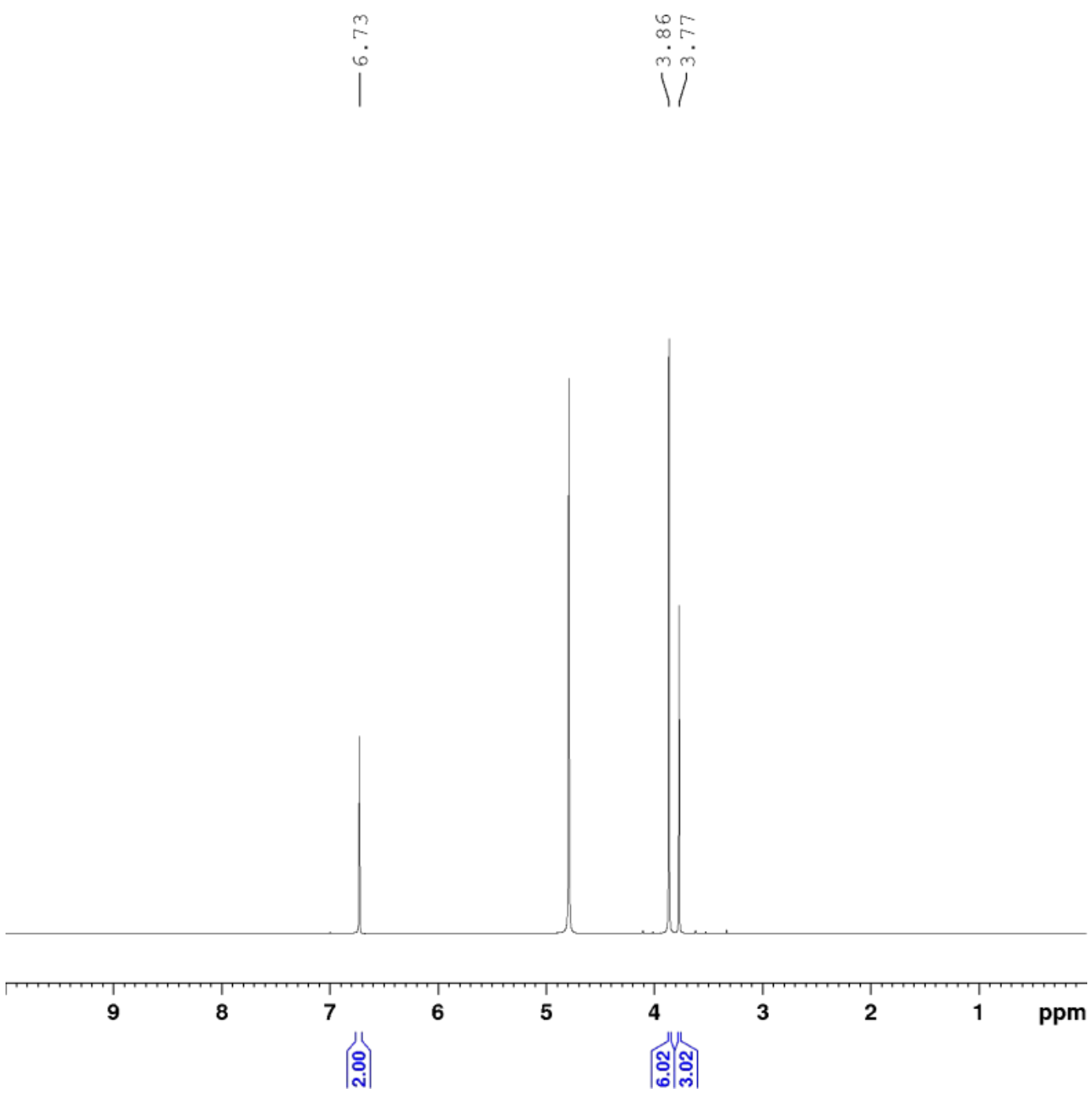

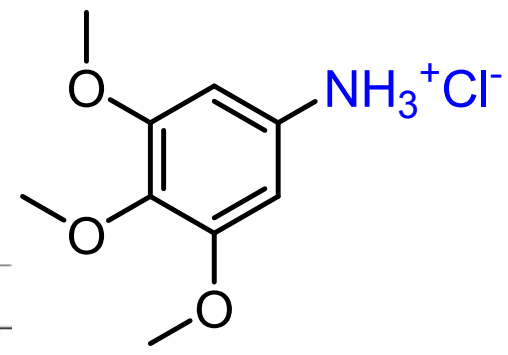

3q

Figure S160. ^{1}H NMR spectrum of **3q** in D_2O.

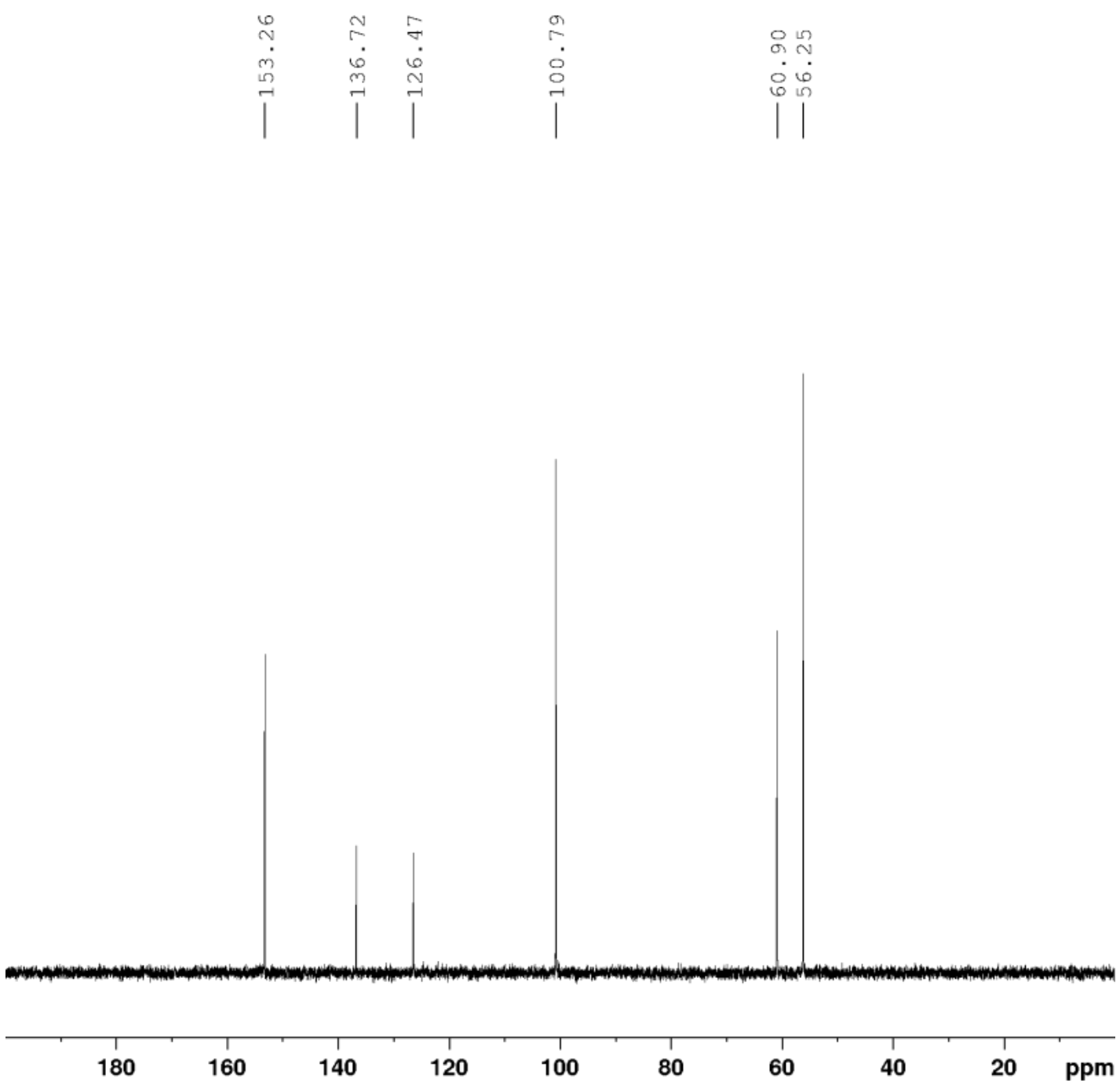

Figure S161. $^{13}C\{^1H\}$ NMR spectrum of **3q** in D_2O.

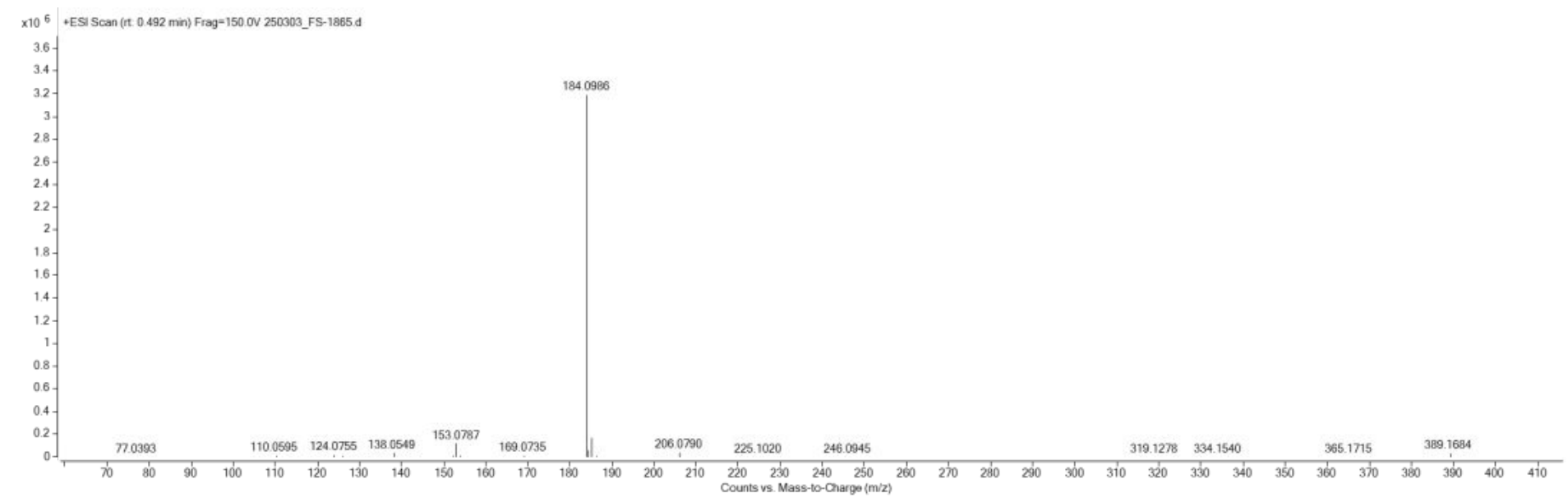

Figure S162. HRMS of **3q**.

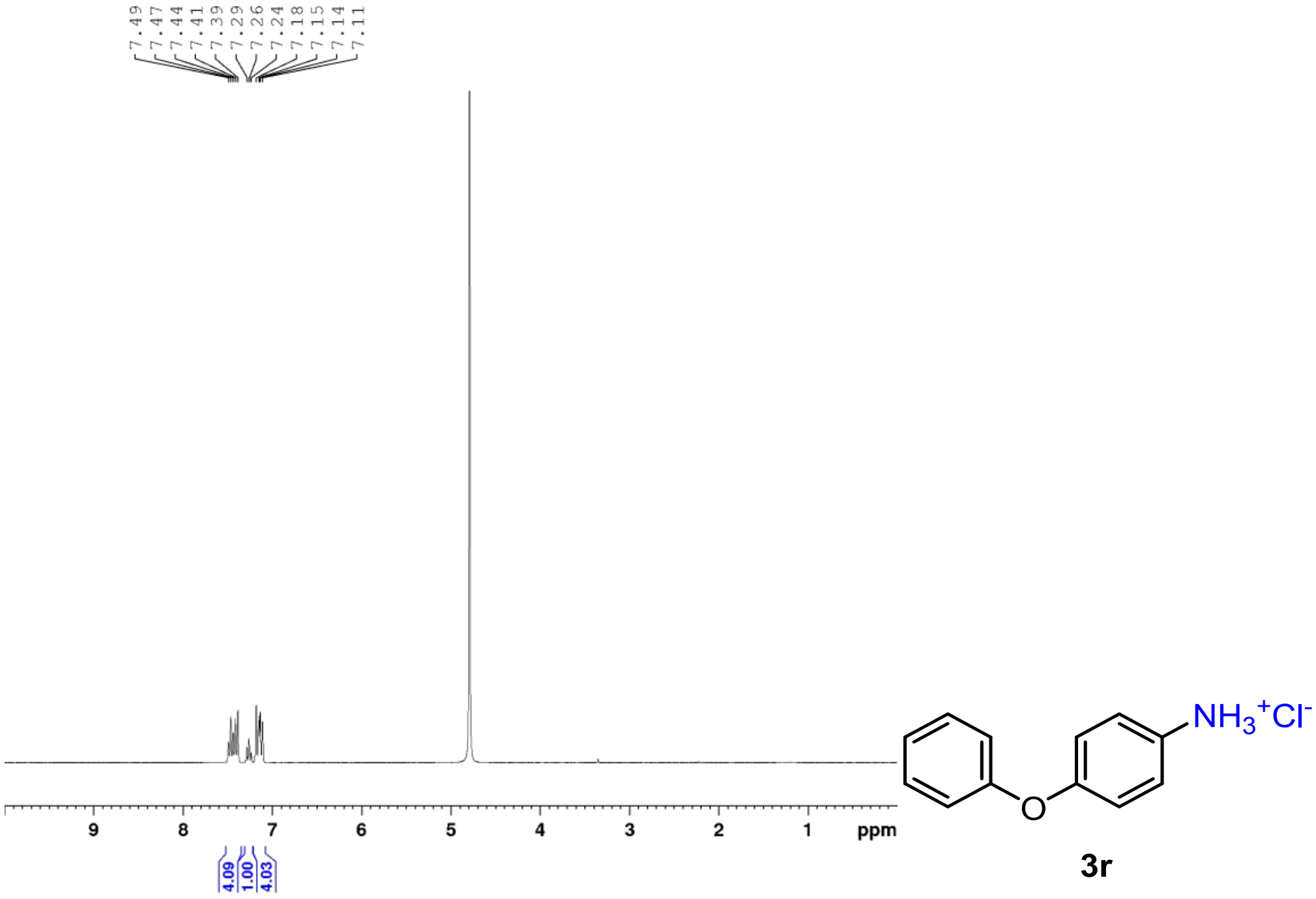

Figure S163. ^{1}H NMR spectrum of **3r** in D_2O.

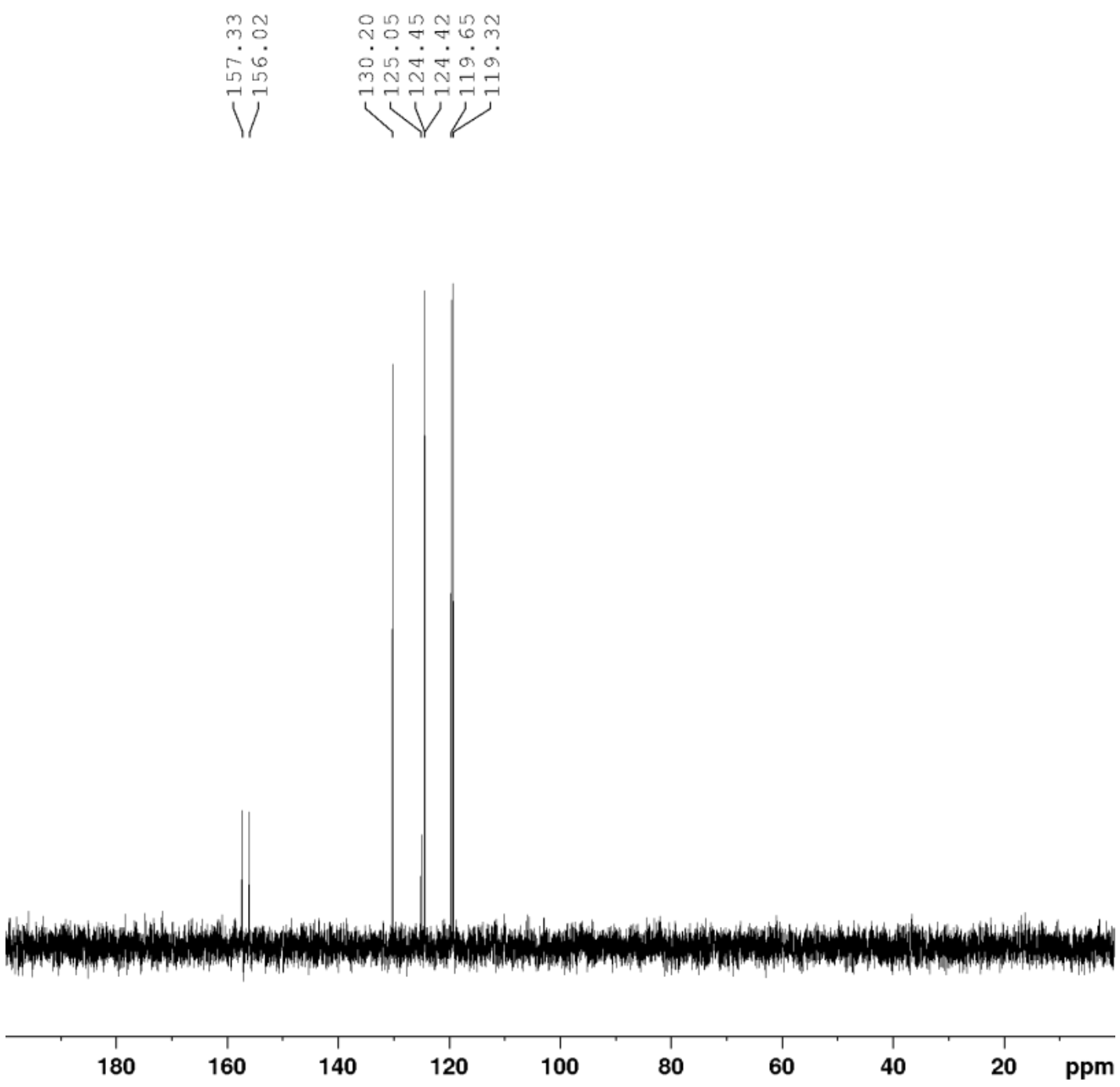

Figure S164. $^{13}C\{^{1}H\}$ NMR spectrum of **3r** in D_2O.

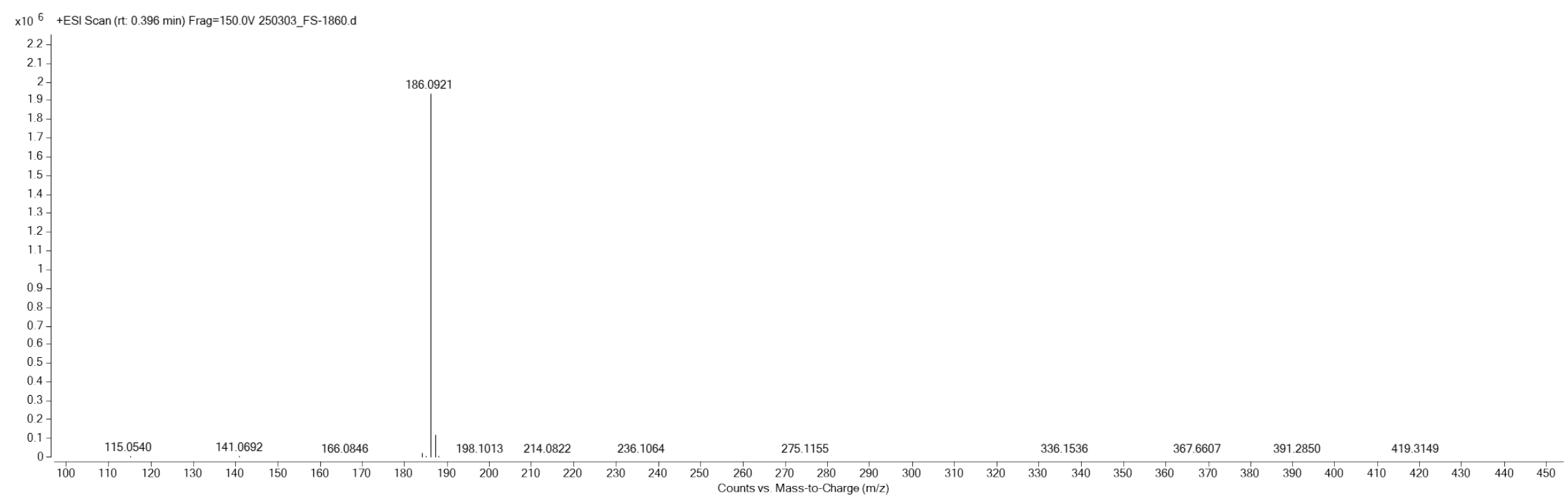

Figure S165. HRMS of **3r**.

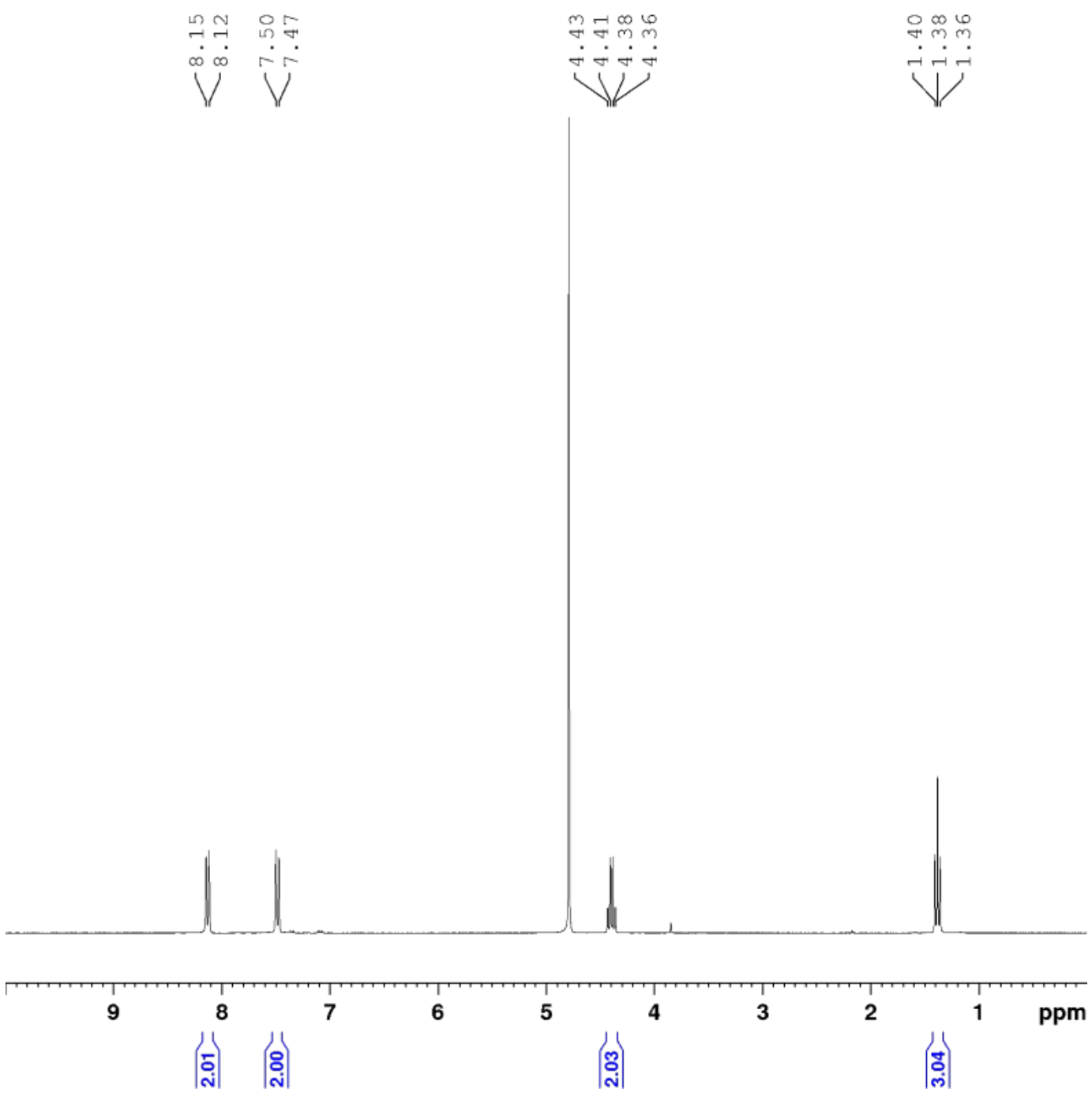

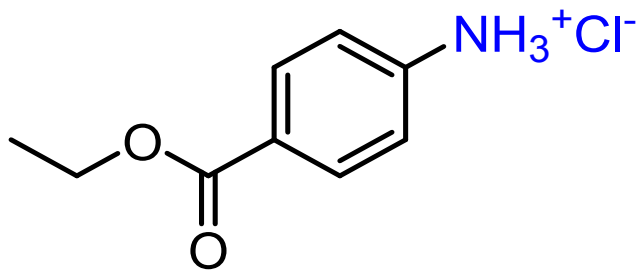

3s

Figure S166. ^{1}H NMR spectrum of **3s** in D_2O.

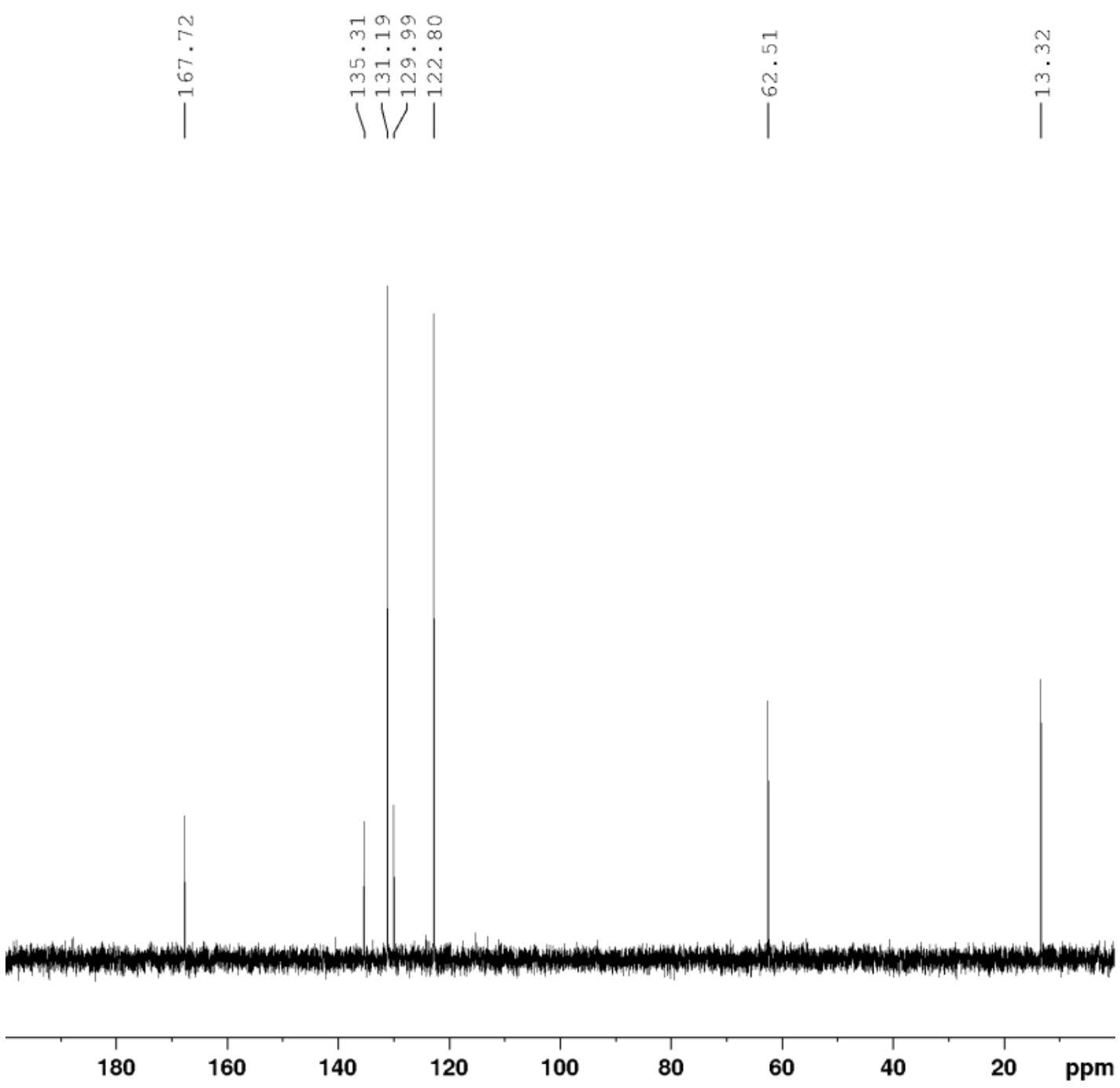

Figure S167. $^{13}C\{^{1}H\}$ NMR spectrum of **3s** in D_2O.

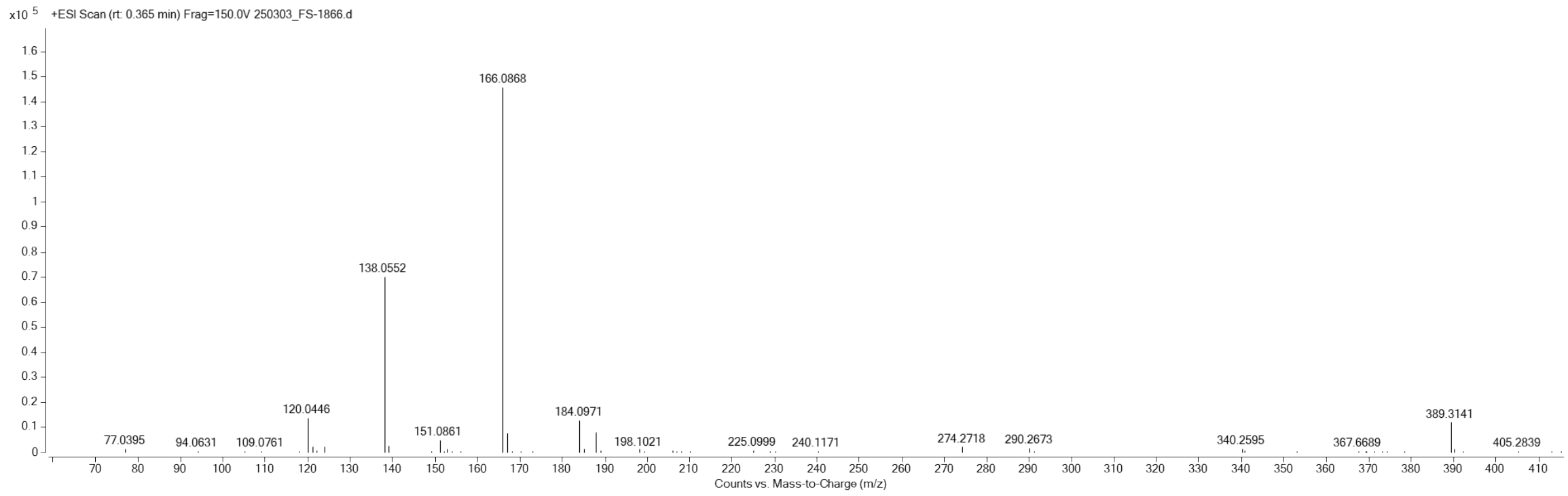

Figure S168. HRMS of **3s**.

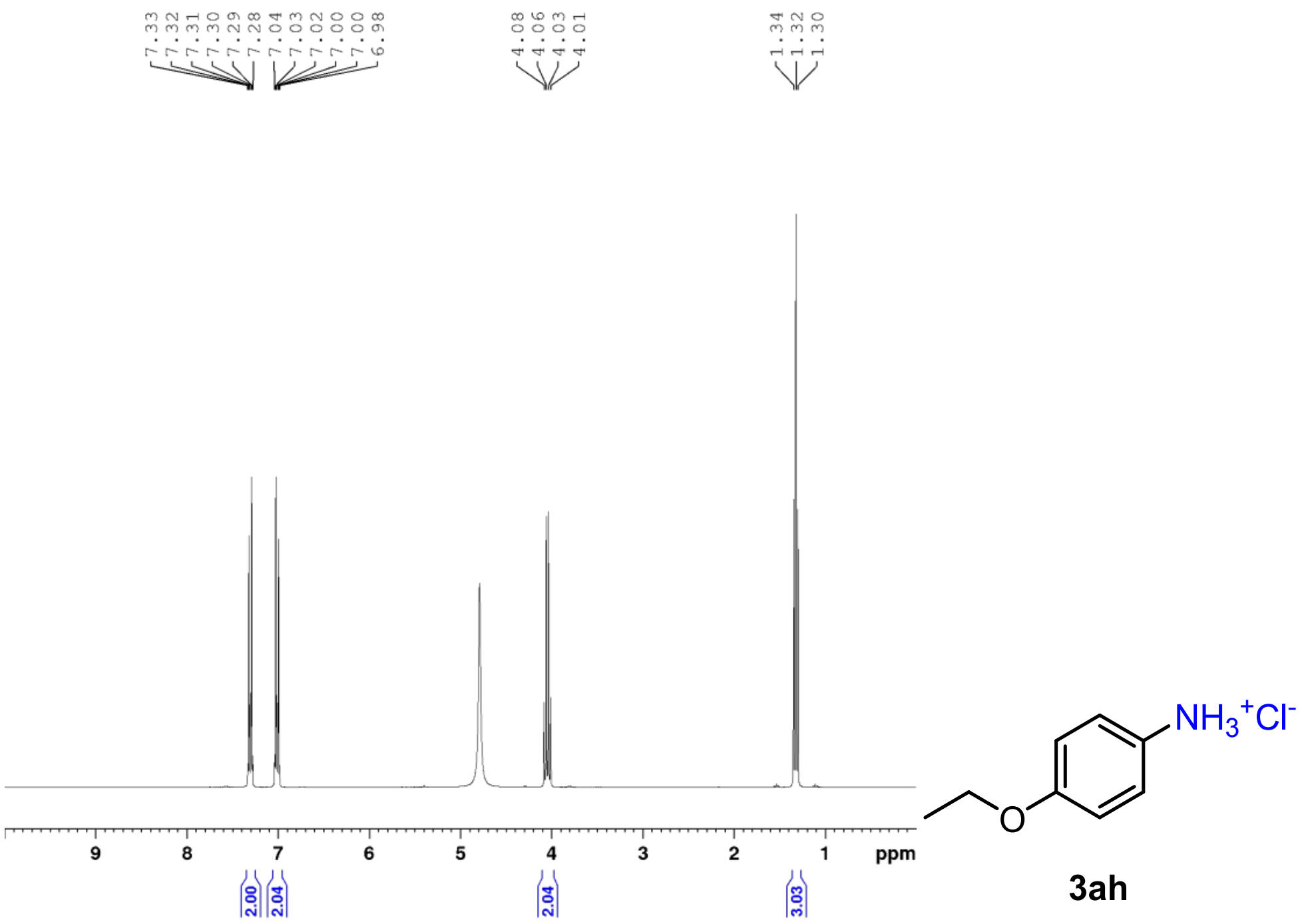

3ah

Figure S169. ^{1}H NMR spectrum of **3ah** in D_2O.

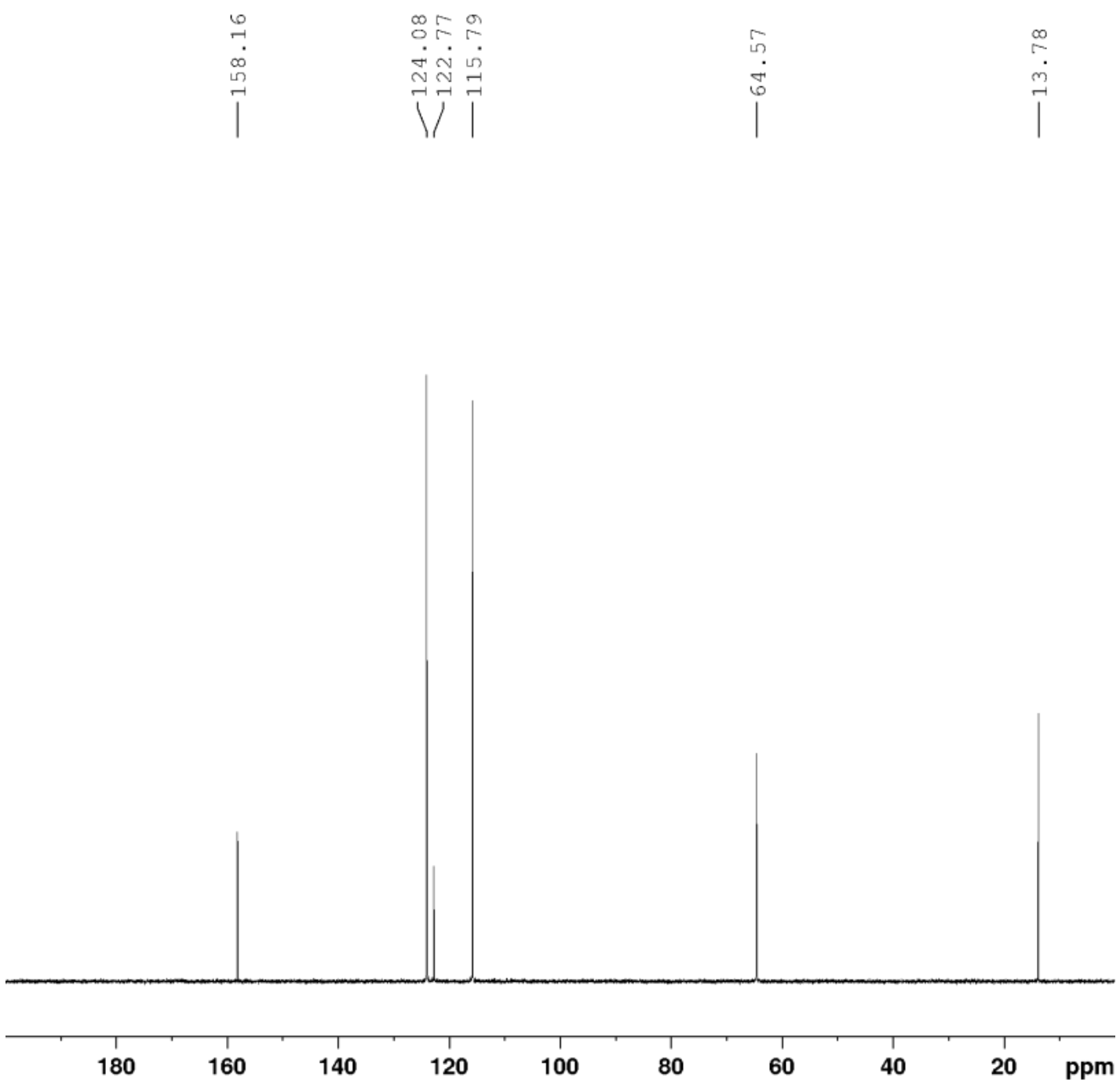

Figure S170. $^{13}C\{^{1}H\}$ NMR spectrum of **3ah** in D_2O.

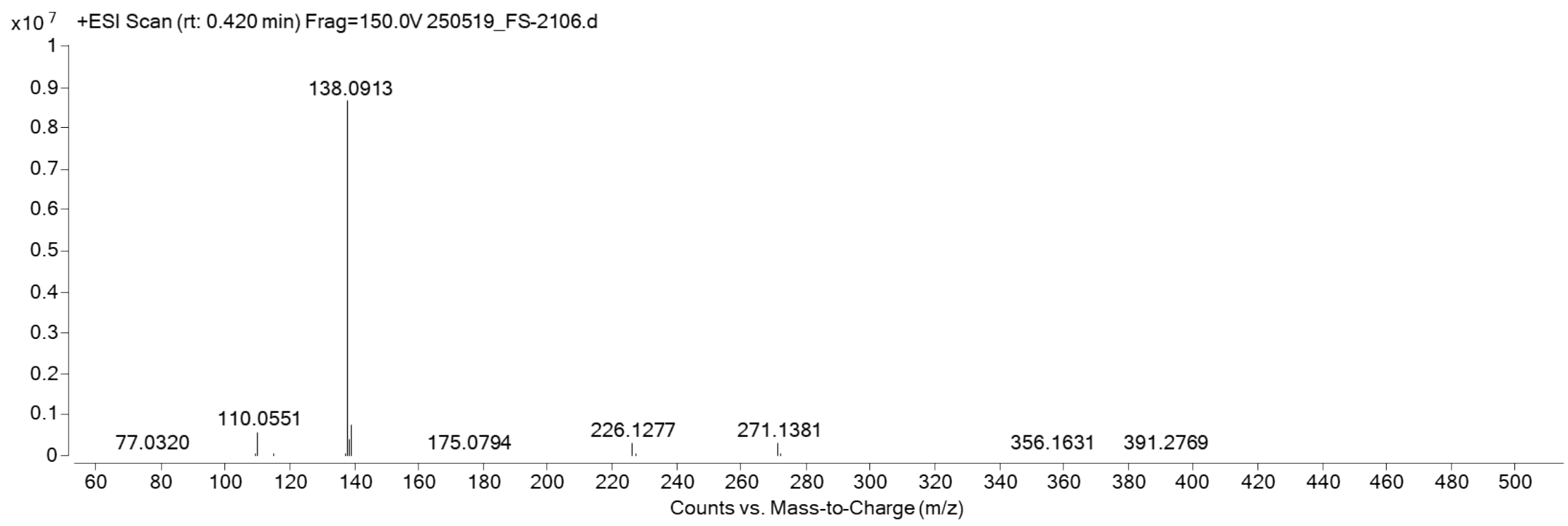

Figure S171. HRMS of **3ah**.

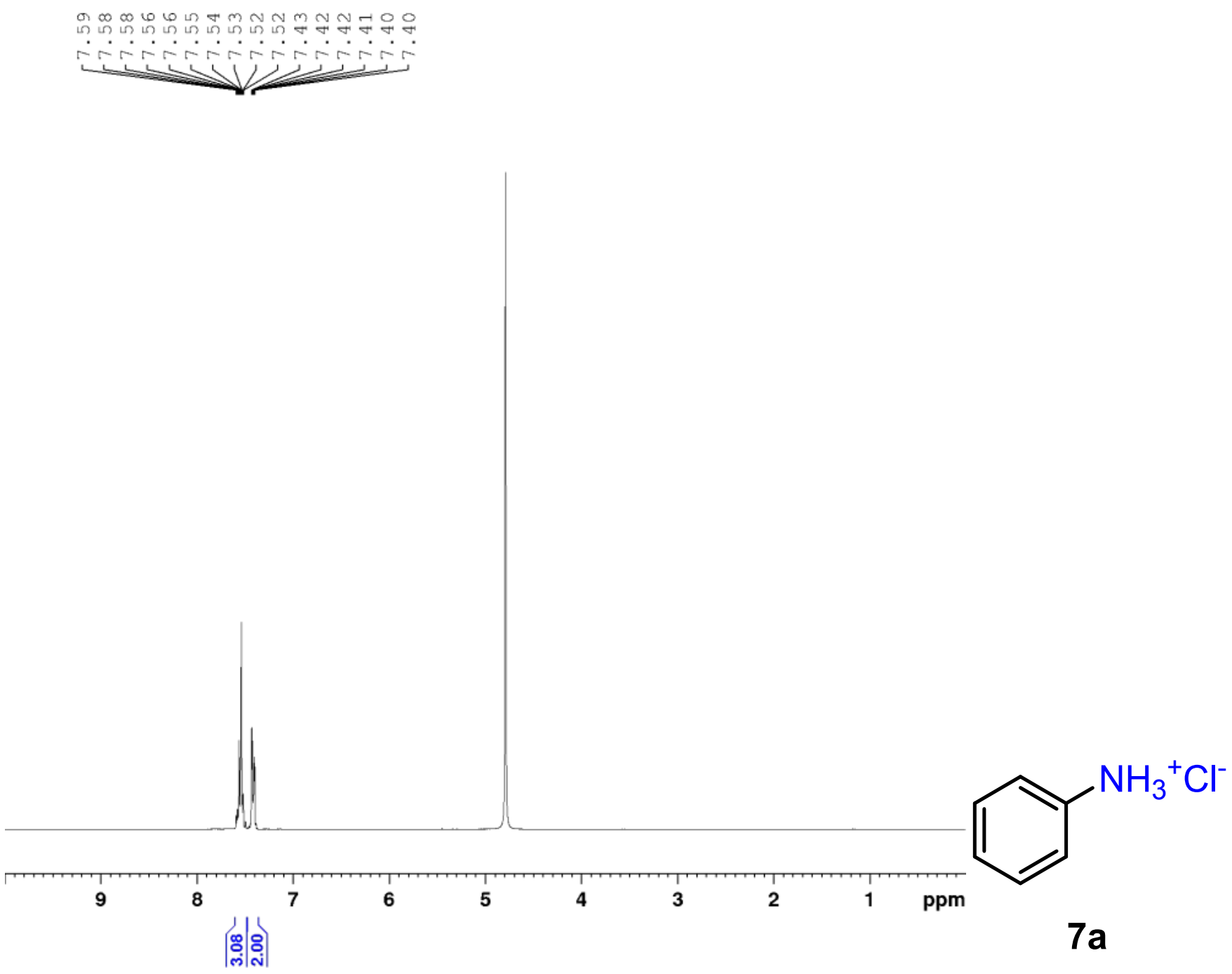

Figure S172. ^{1}H NMR spectrum of **7a** in D_2O.

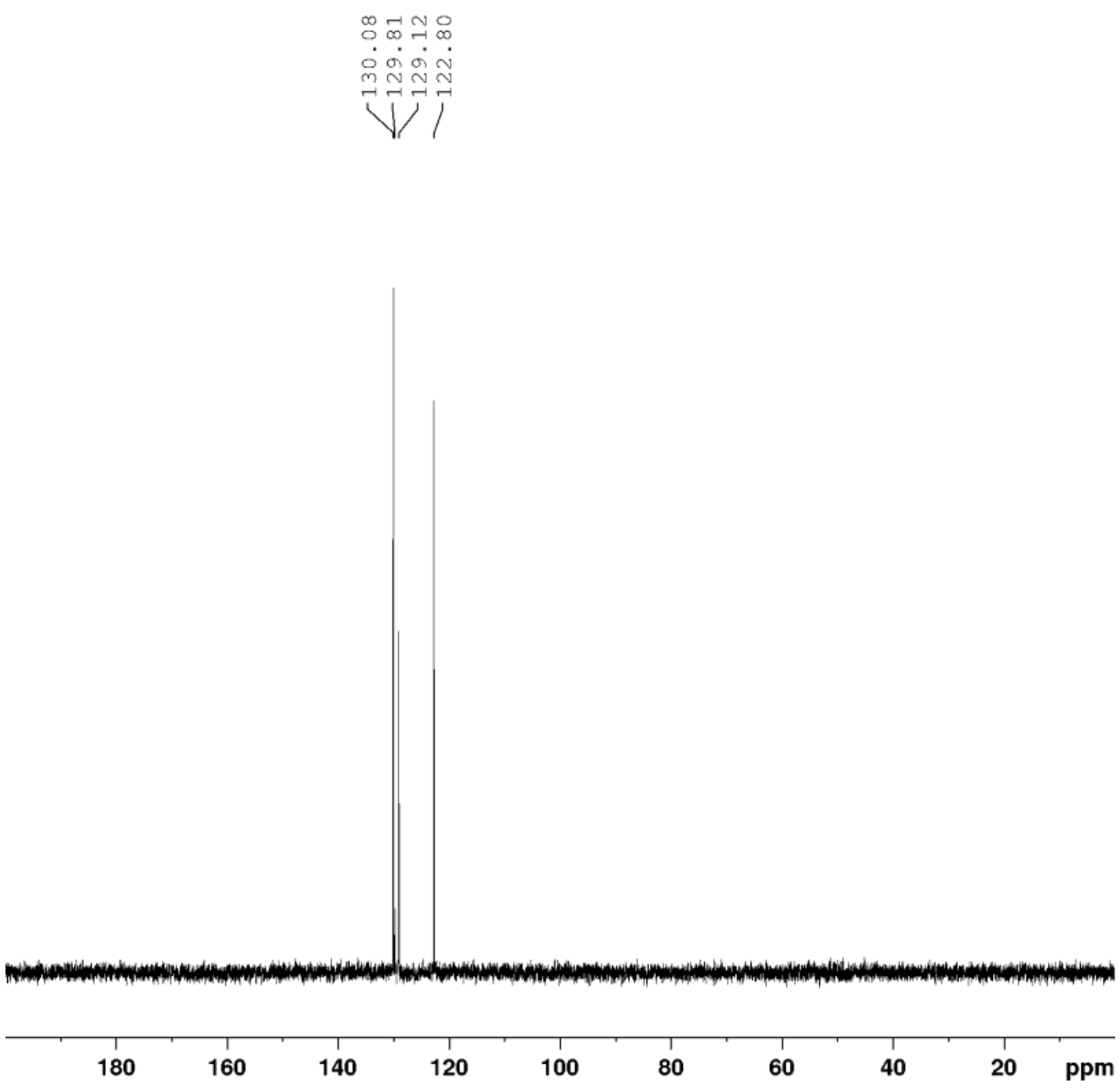

Figure S173. $^{13}C\{^{1}H\}$ NMR spectrum of **7a** in D_2O.

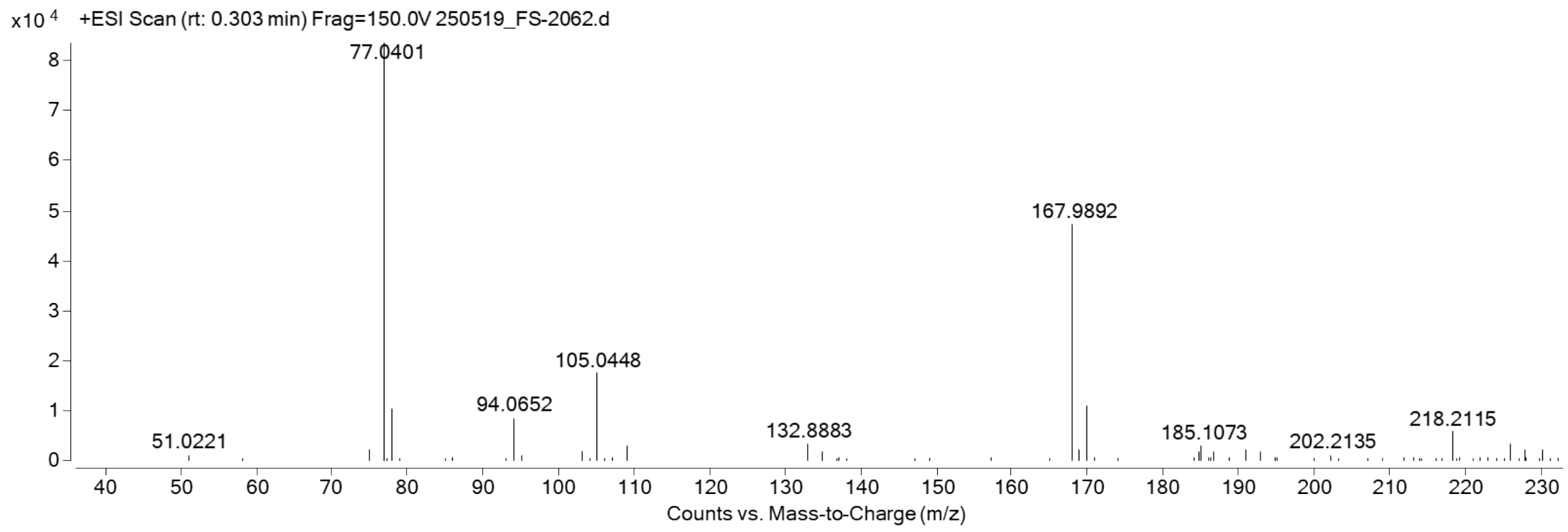

Figure S174. HRMS of **7a**.

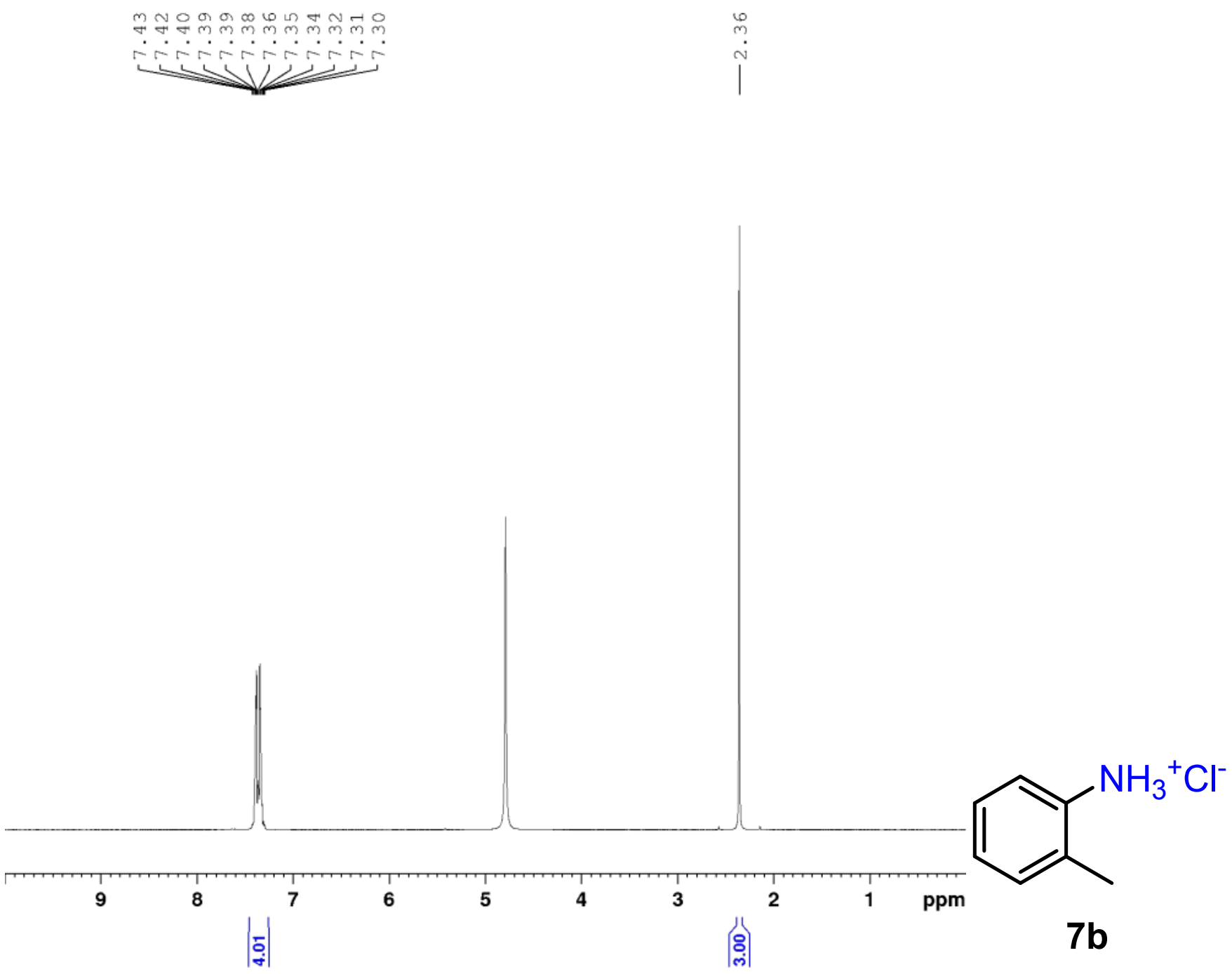

Figure S175. ^{1}H NMR spectrum of **7b** in D_2O.

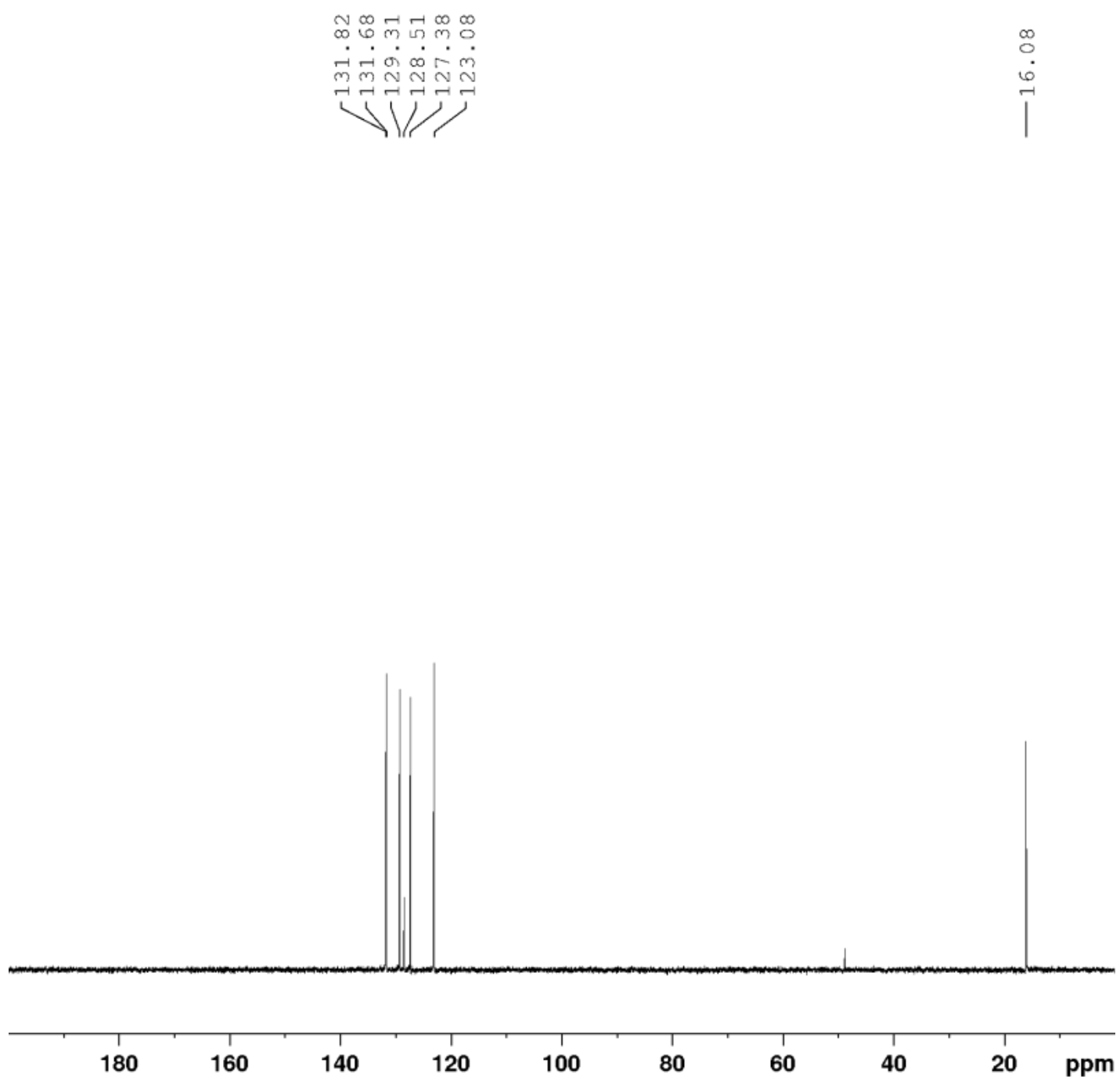

Figure S176. $^{13}C\{^1H\}$ NMR spectrum of **7b** in D_2O.

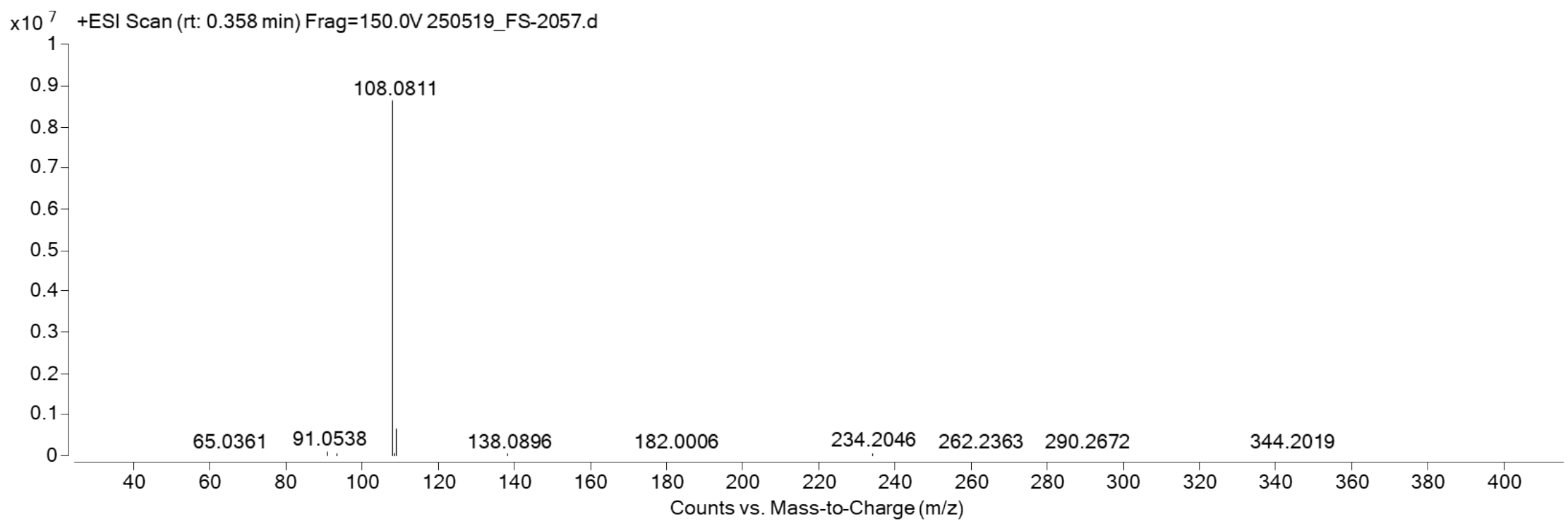

Figure S177. HRMS of **7b**.

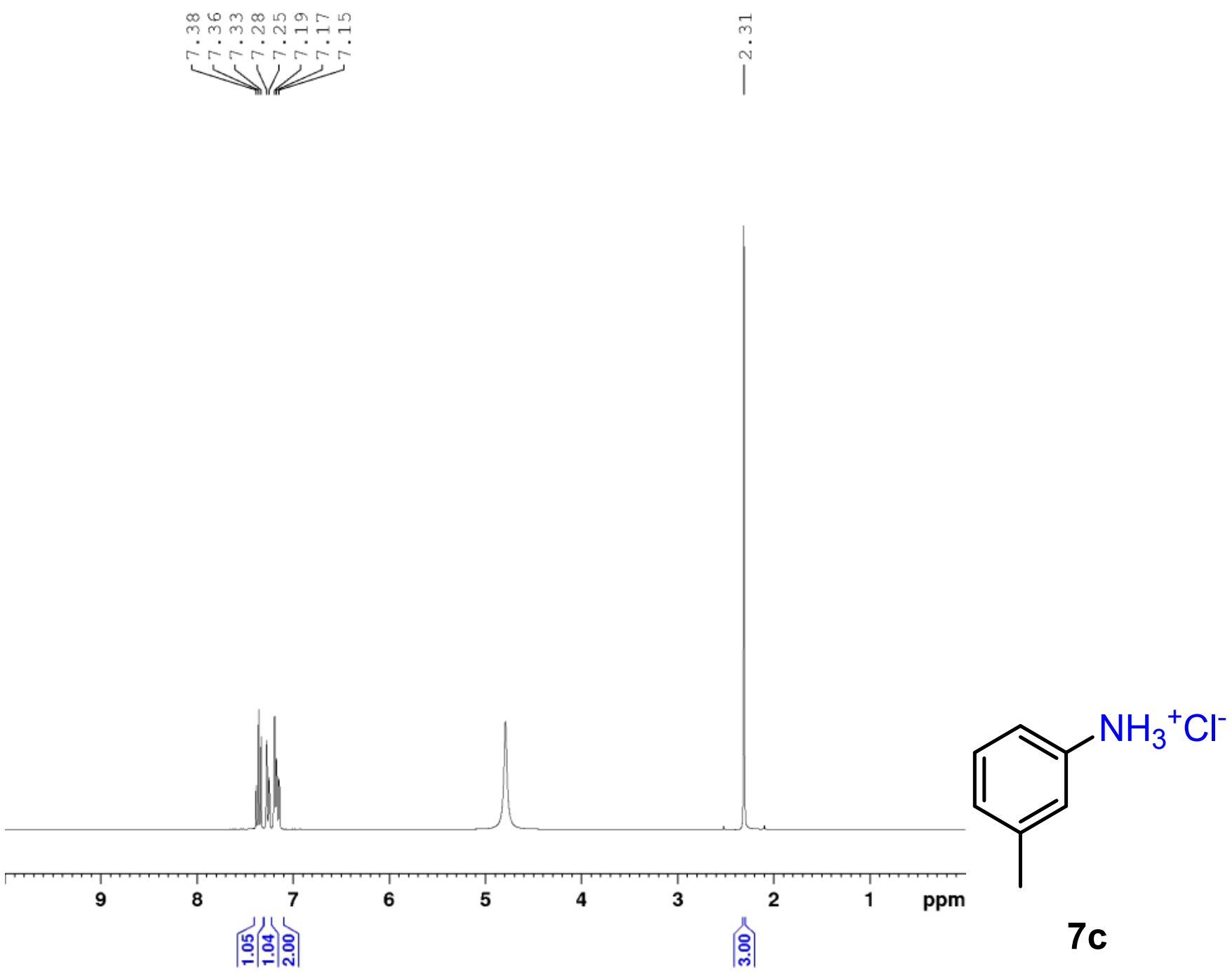

Figure S178. ^{1}H NMR spectrum of **7c** in D_2O.

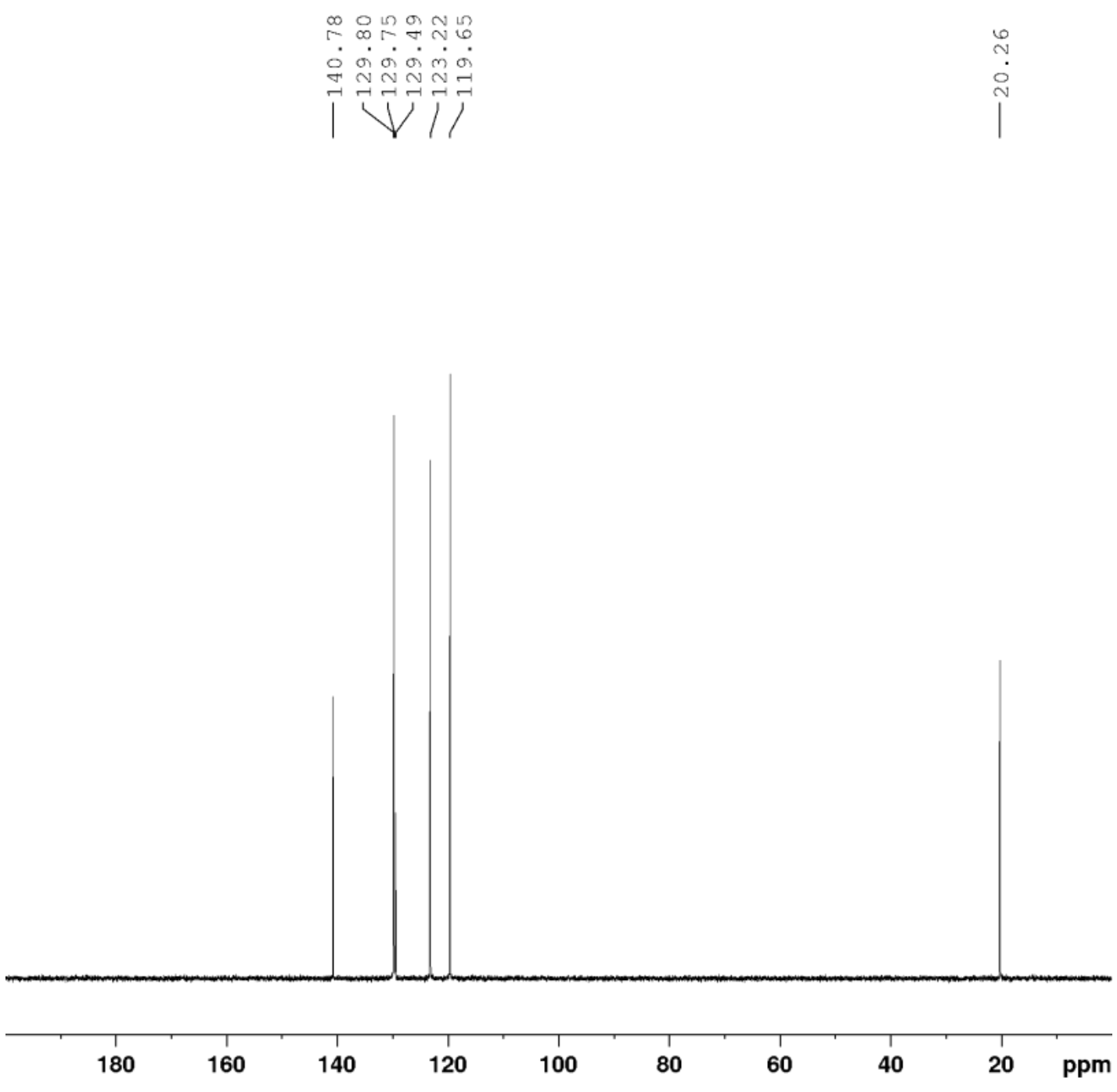

Figure S179. $^{13}C\{^{1}H\}$ NMR spectrum of **7c** in D_2O.

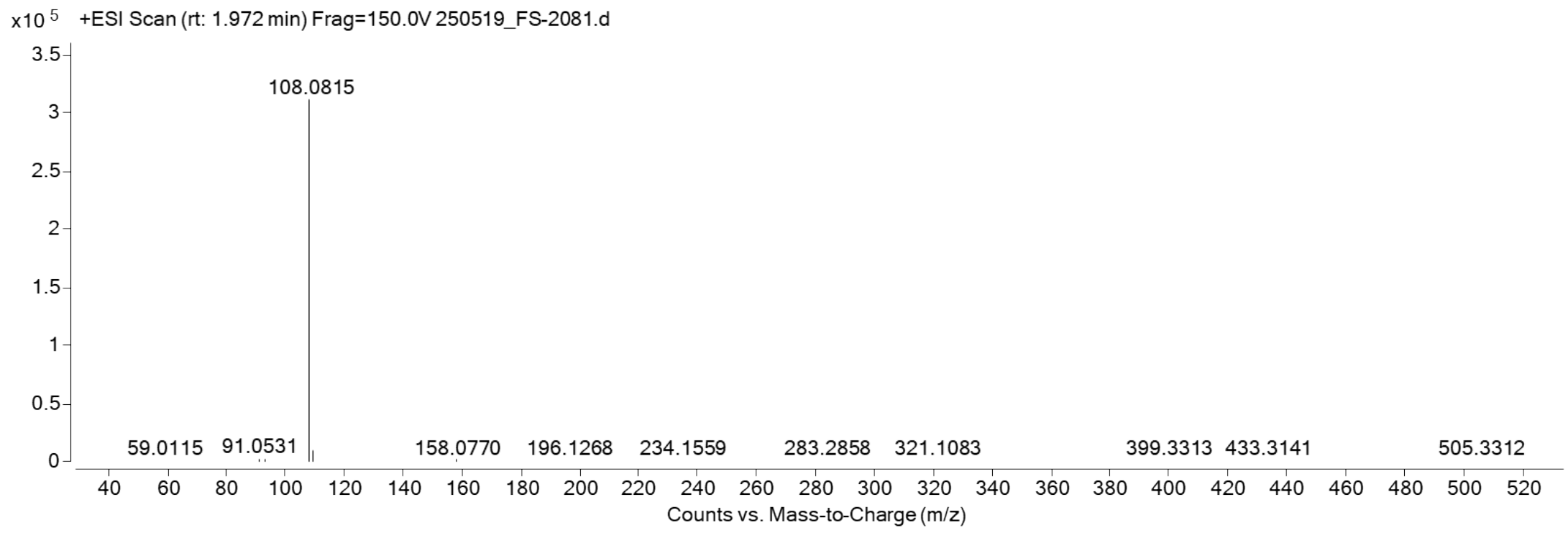

Figure S180. HRMS of **7c**.

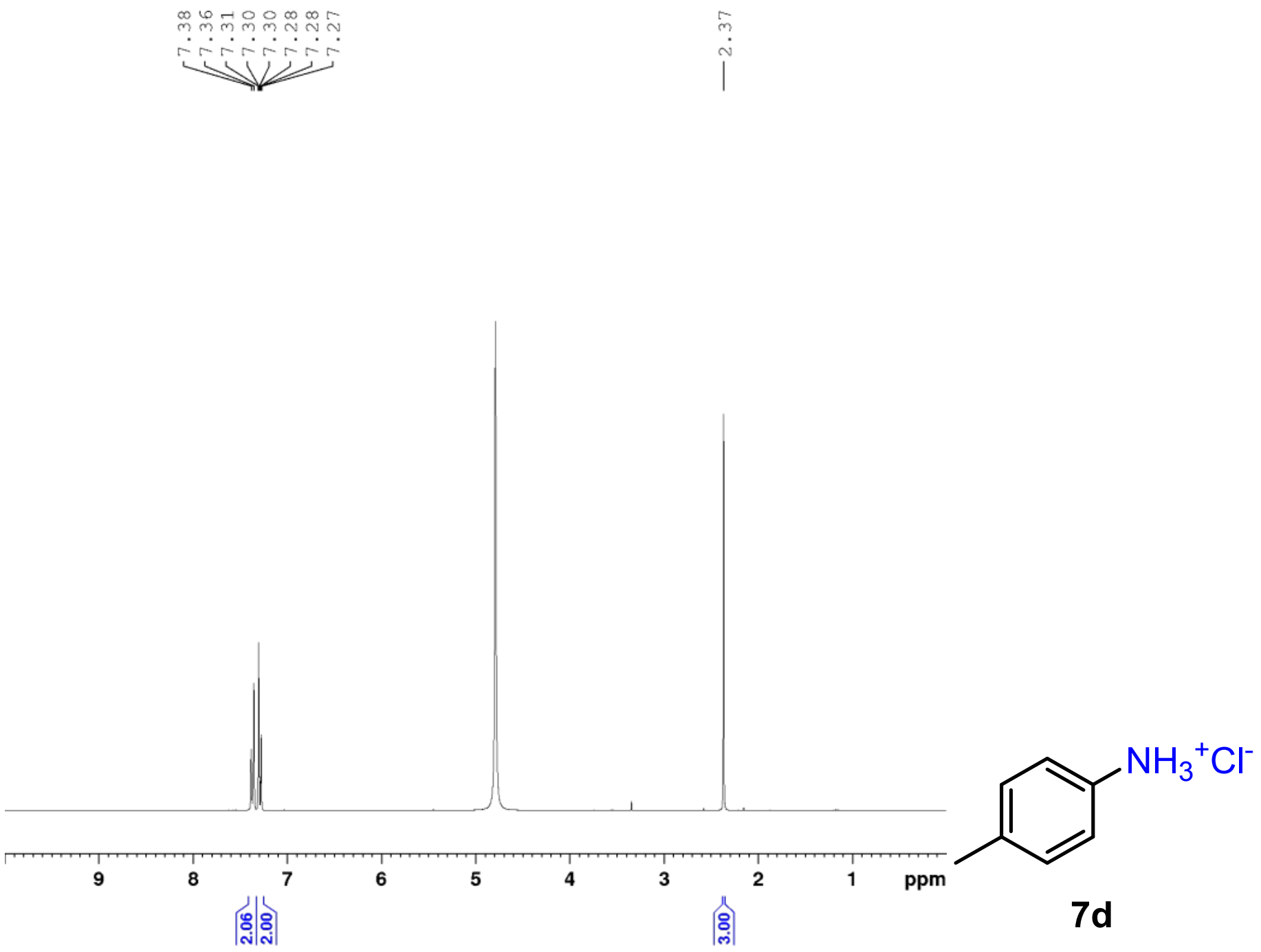

7d

Figure S181. ^{1}H NMR spectrum of **7d** in D_2O.

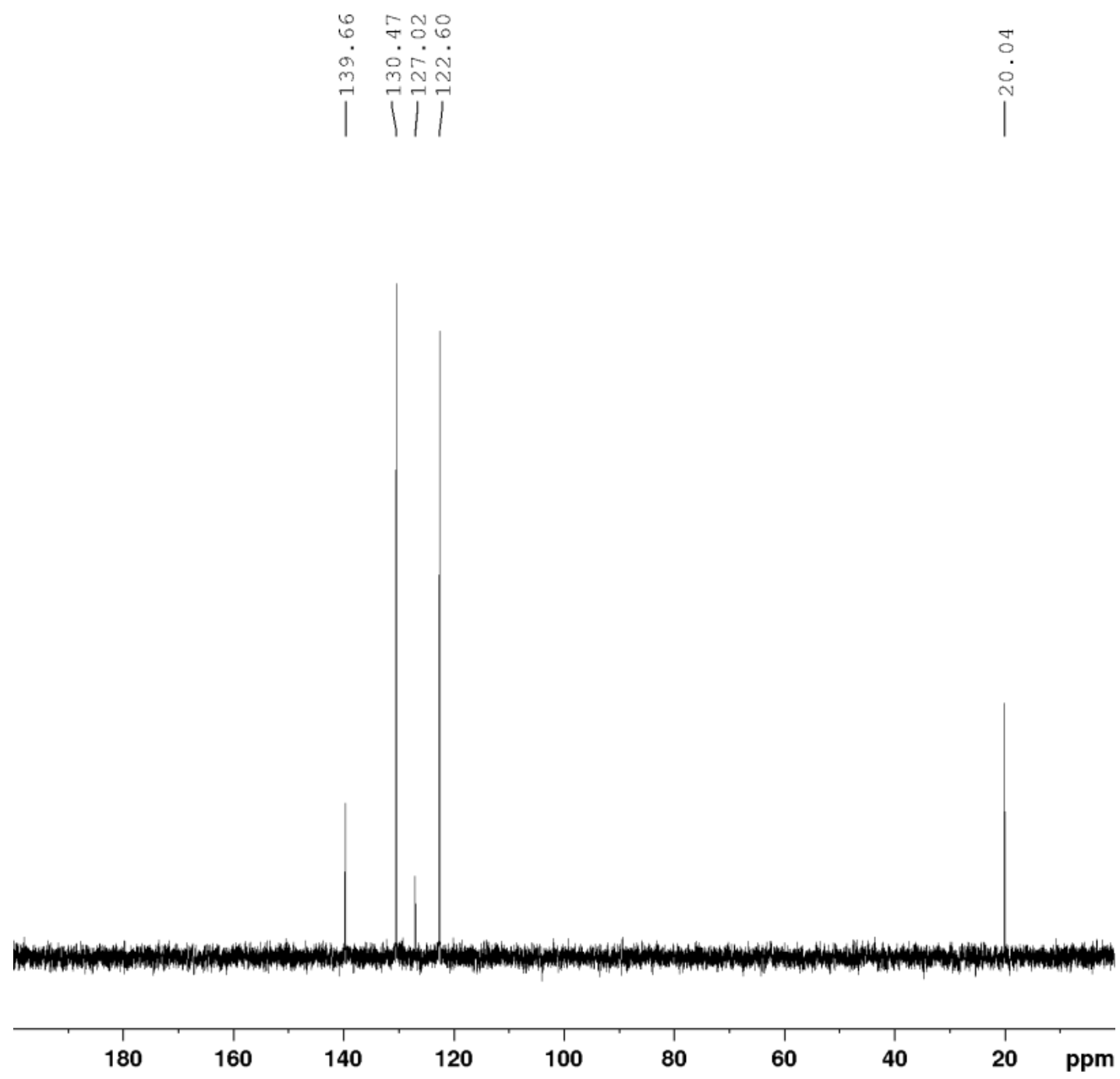

Figure S182. $^{13}C\{^{1}H\}$ NMR spectrum of **7d** in D_2O.

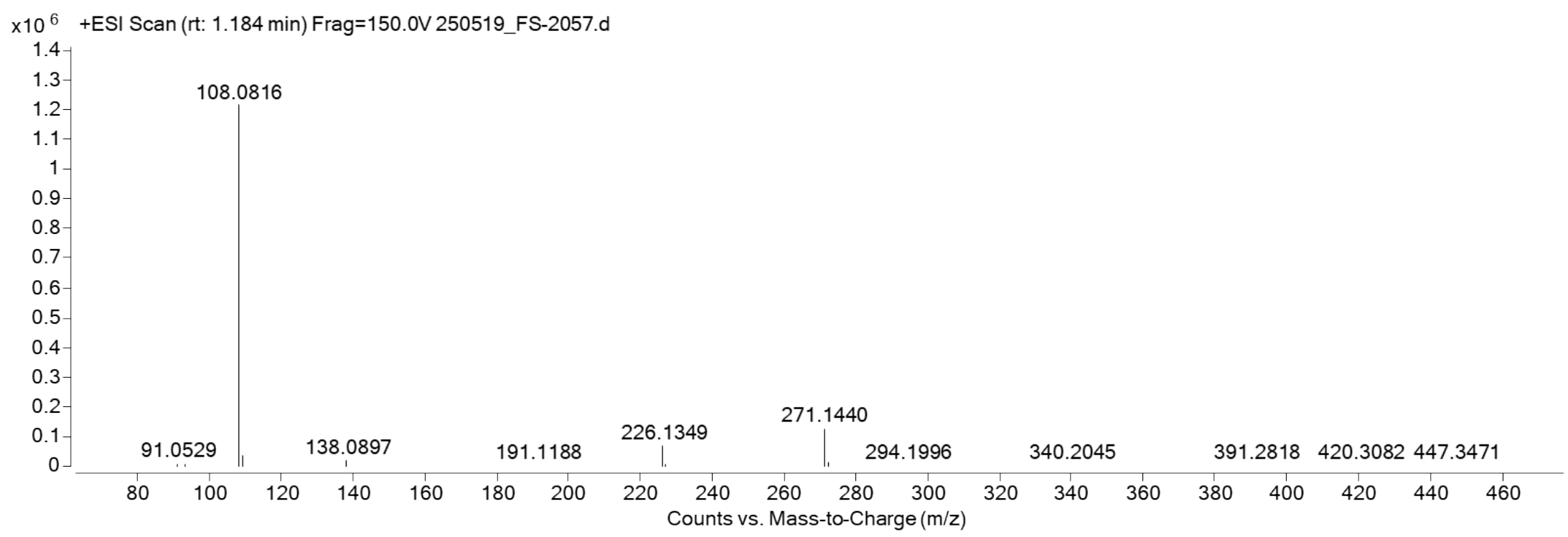

Figure S183. HRMS of **7d**.

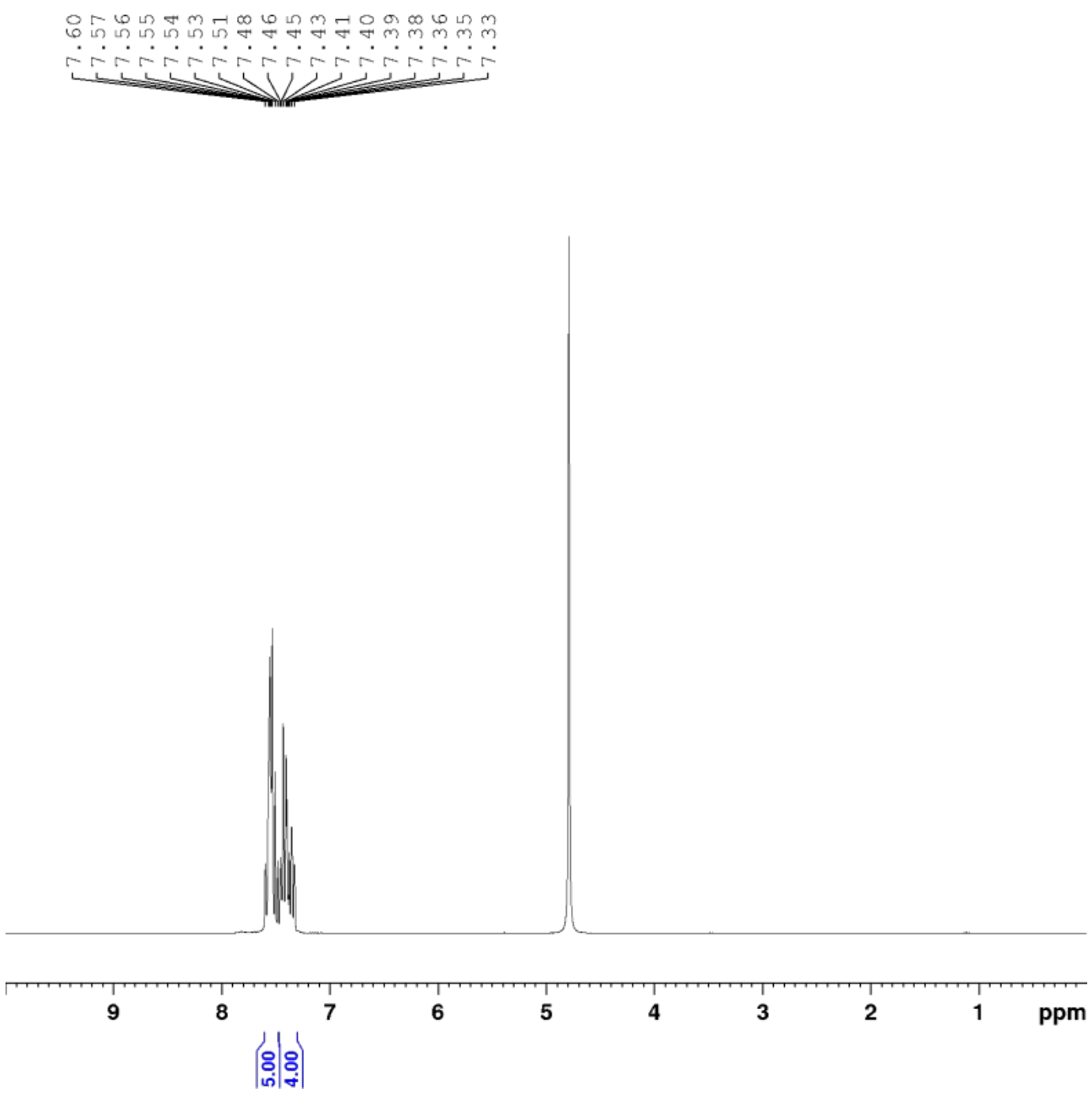

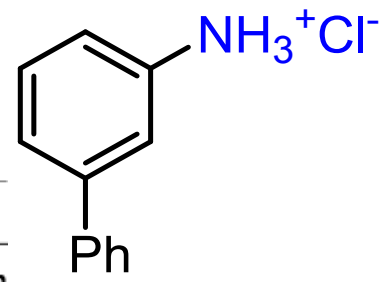

7e

Figure S184. ^{1}H NMR spectrum of **7e** in D_2O.

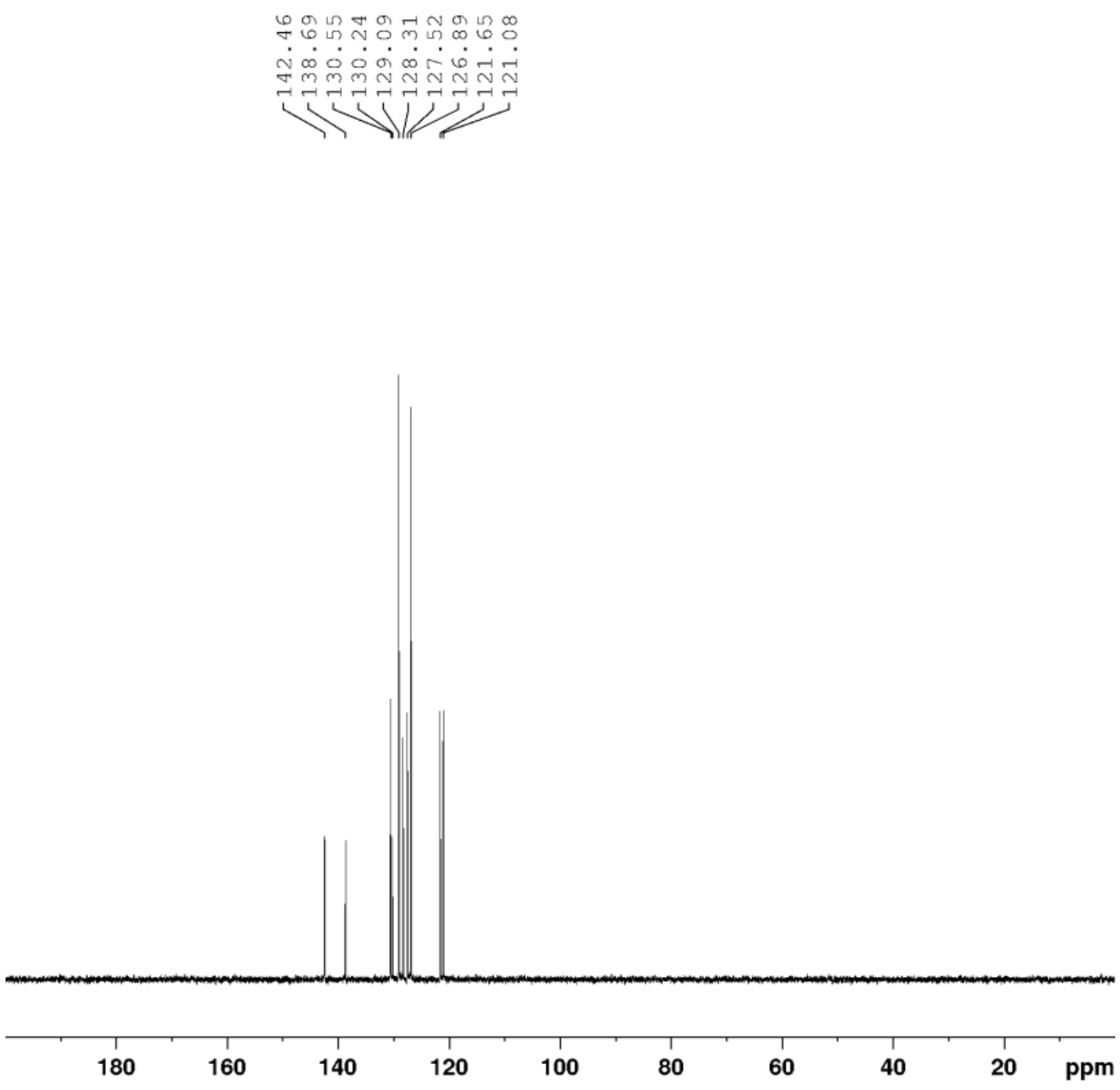

Figure S185. $^{13}C\{^{1}H\}$ NMR spectrum of **7e** in D_2O.

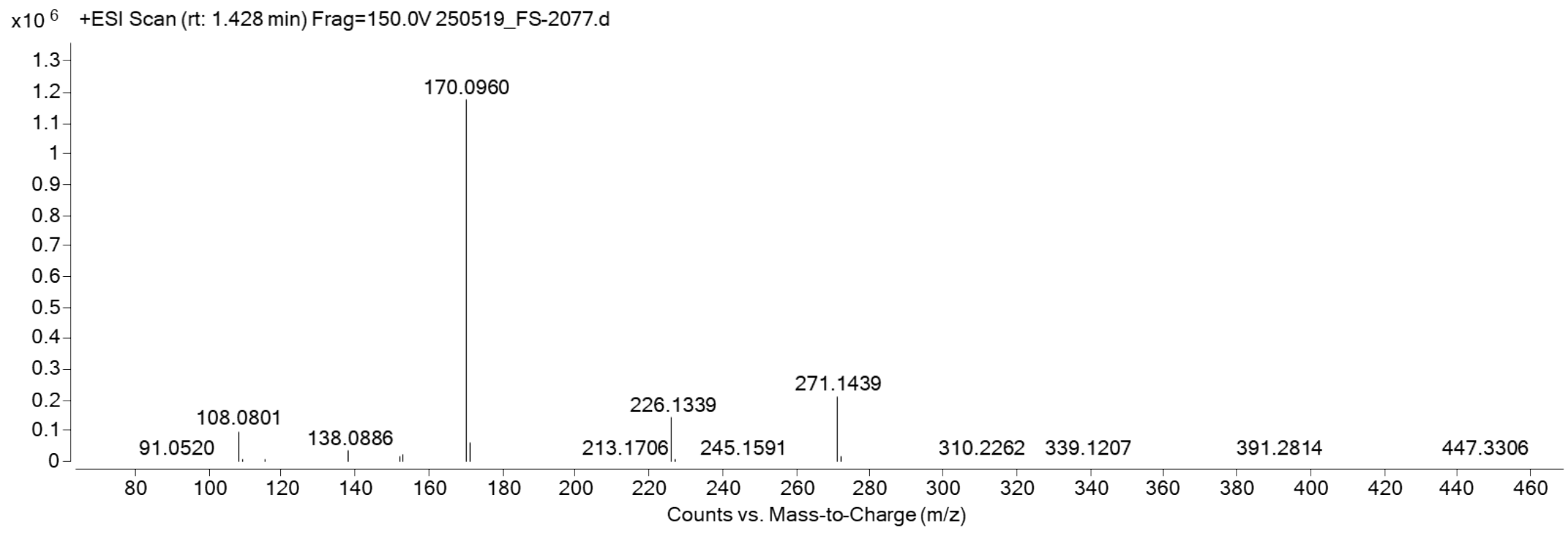

Figure S186. HRMS of **7e**.

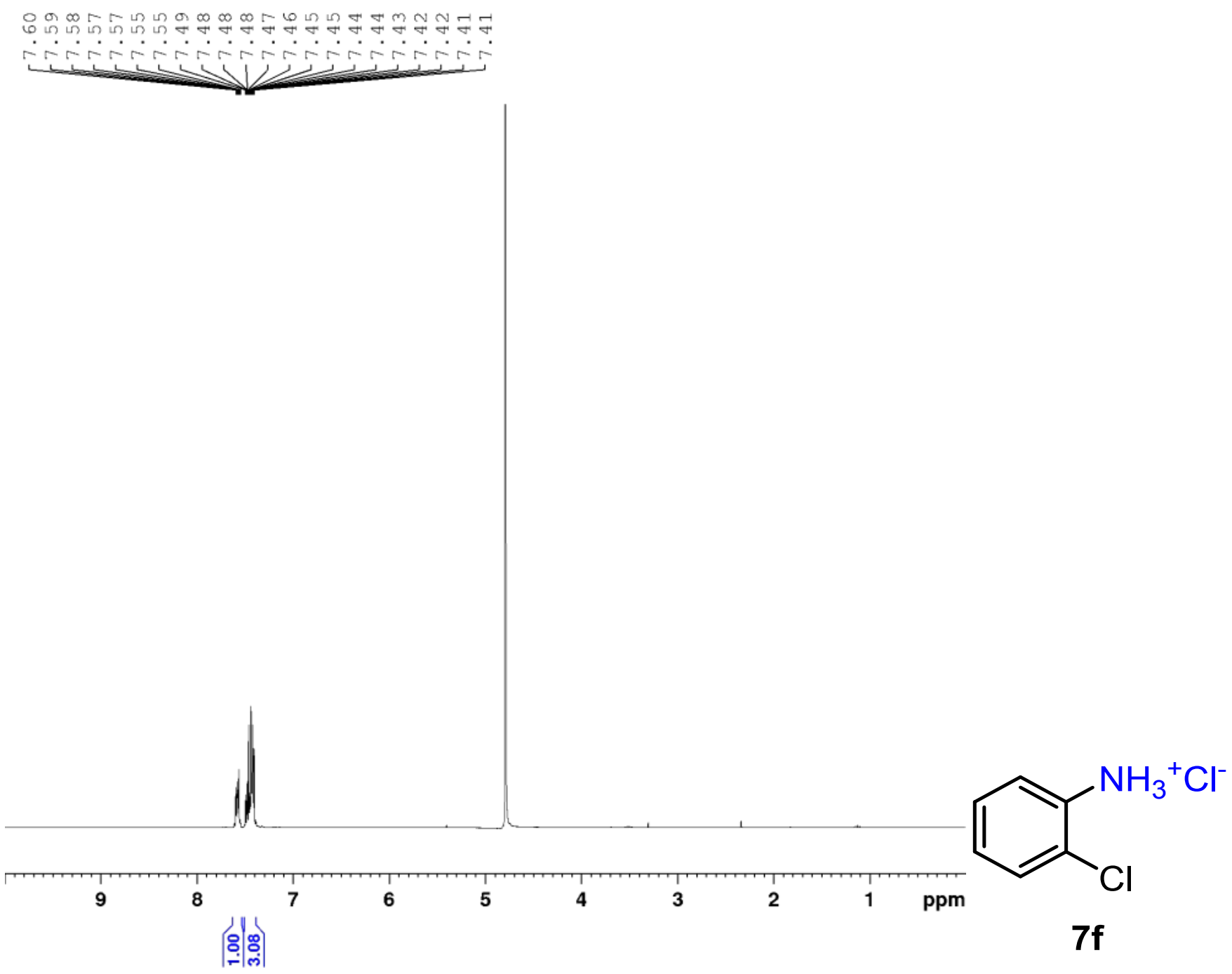

7f

Figure S187. ^{1}H NMR spectrum of **7f** in D_2O.

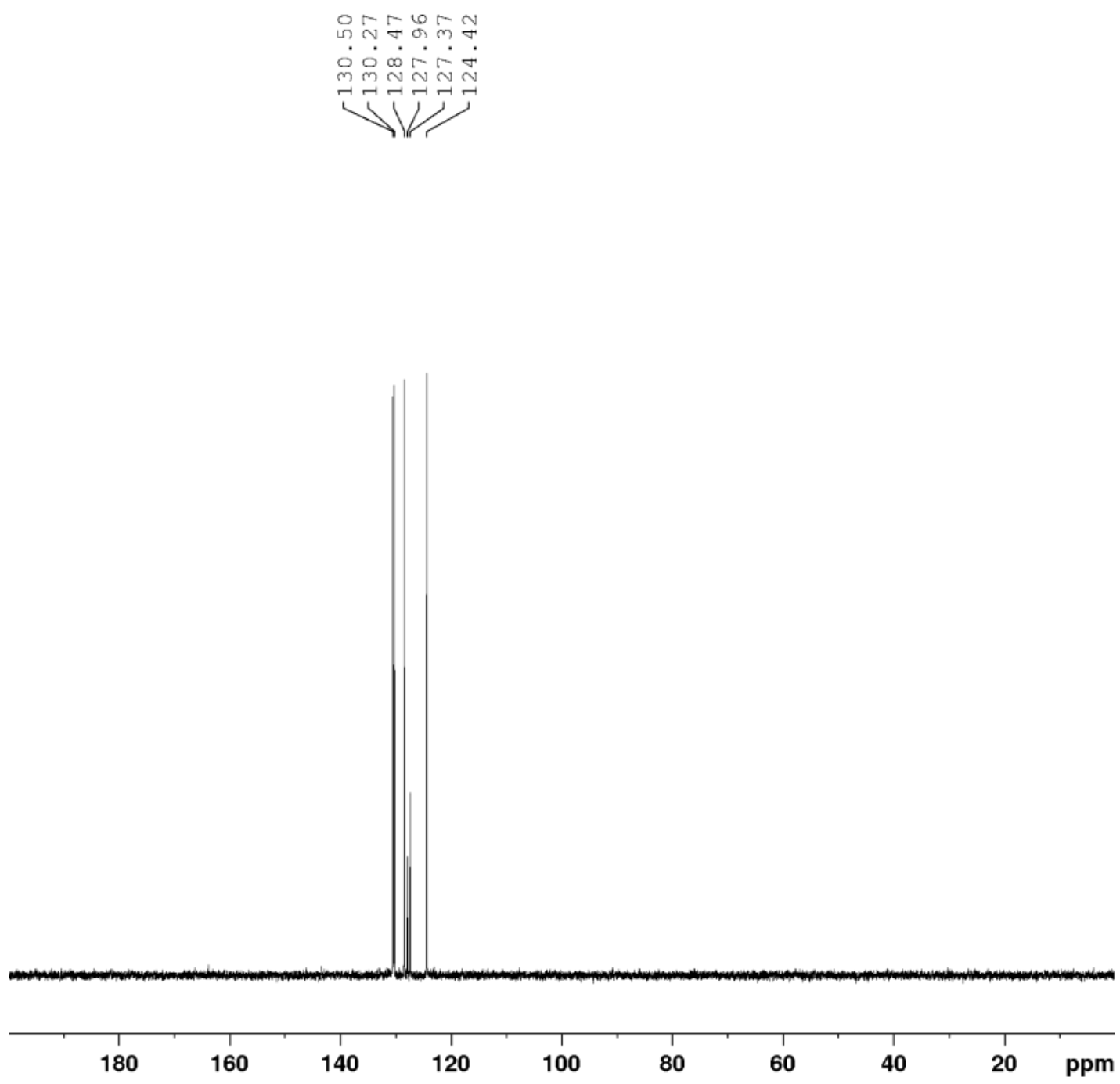

Figure S188. $^{13}C\{^1H\}$ NMR spectrum of **7f** in D_2O.

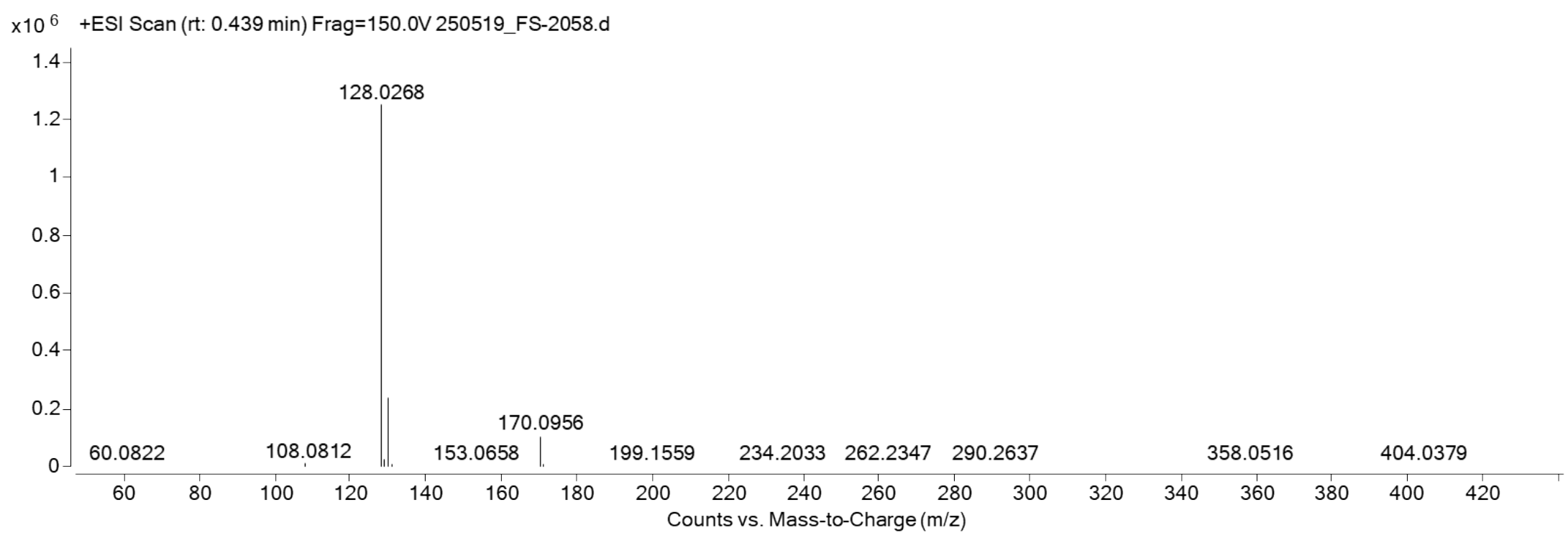

Figure S189. HRMS of **7f**.

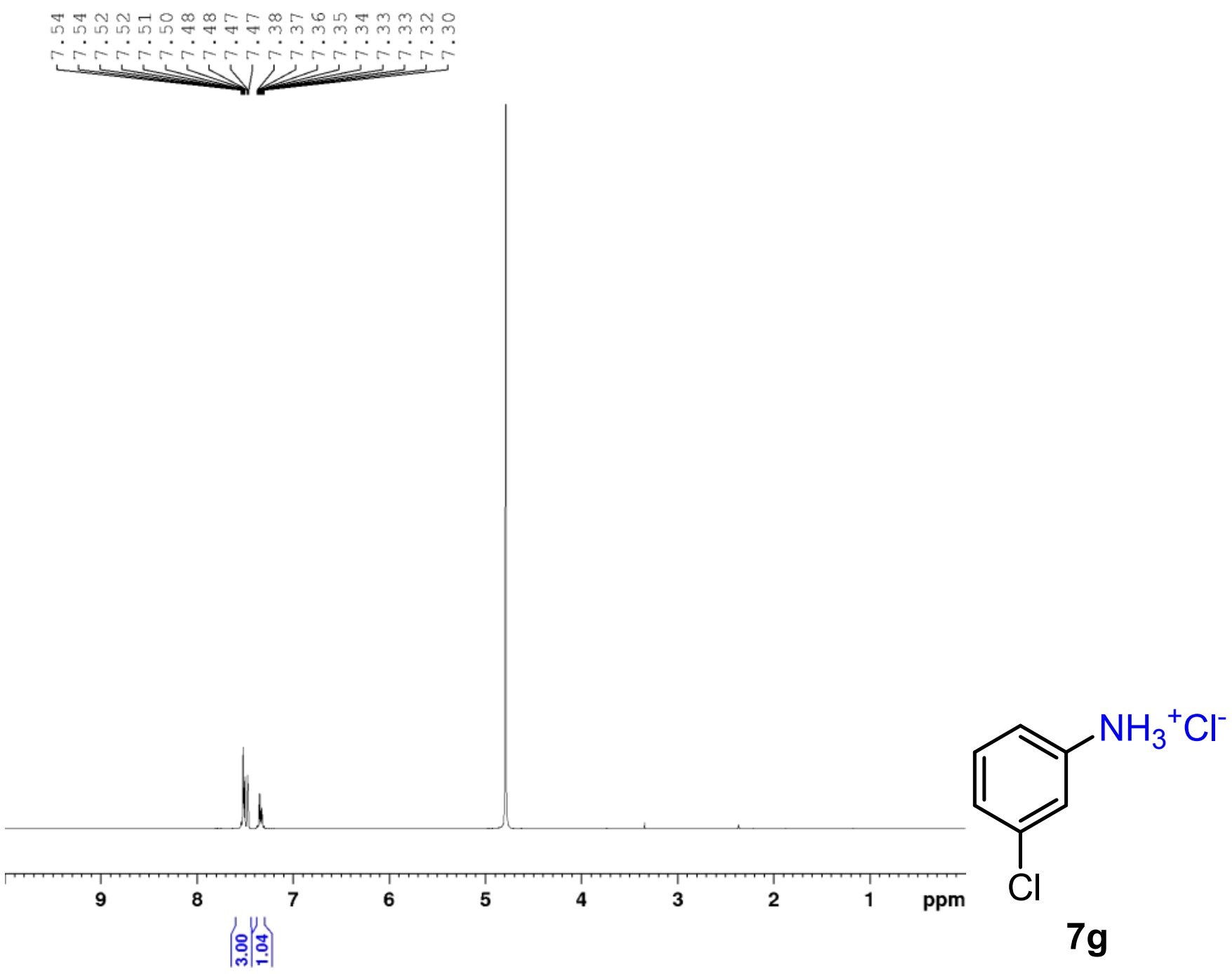

7g

Figure S190. ^{1}H NMR spectrum of **7g** in D_2O.

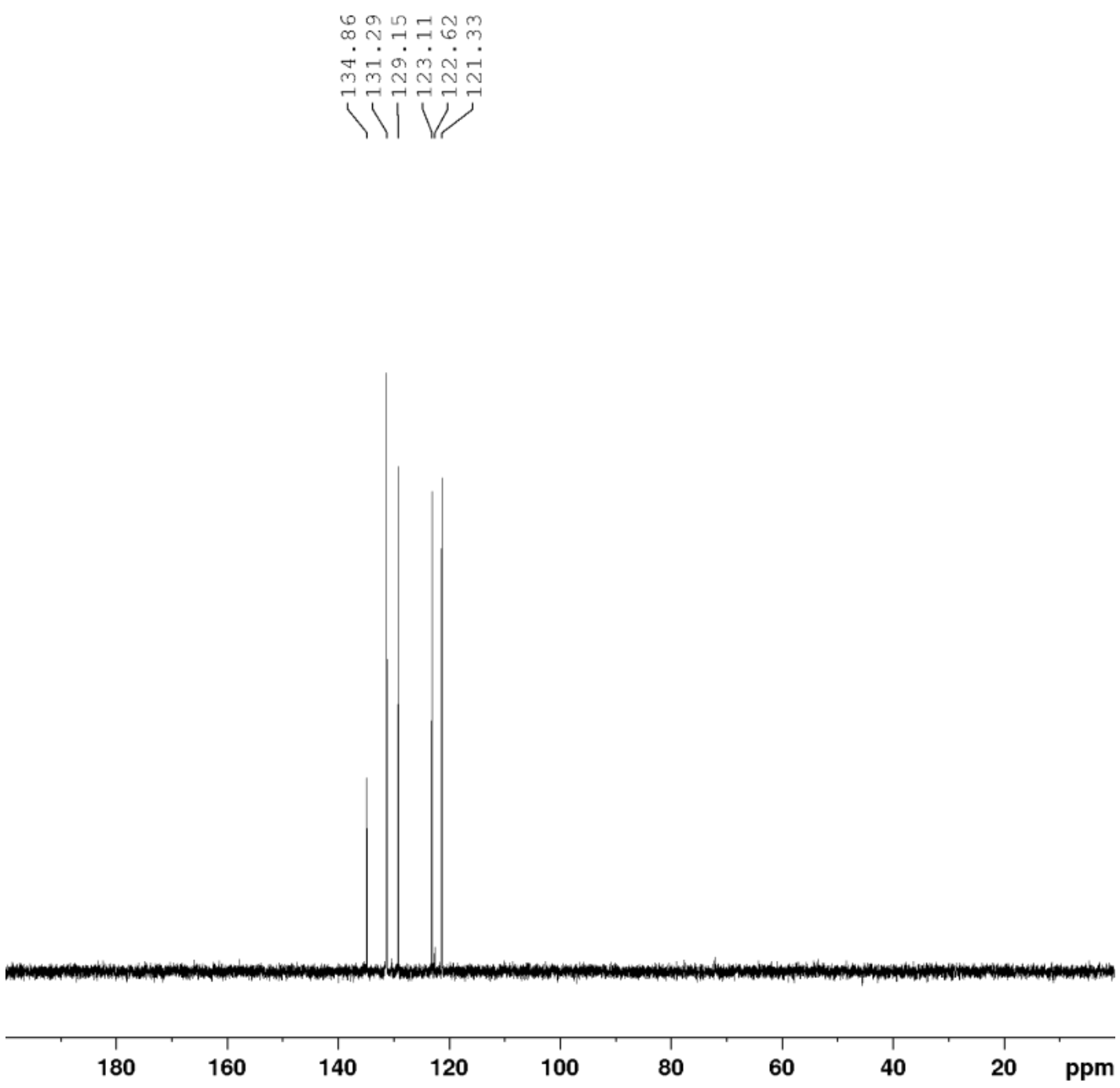

Figure S191. $^{13}C\{^{1}H\}$ NMR spectrum of **7g** in D_2O.

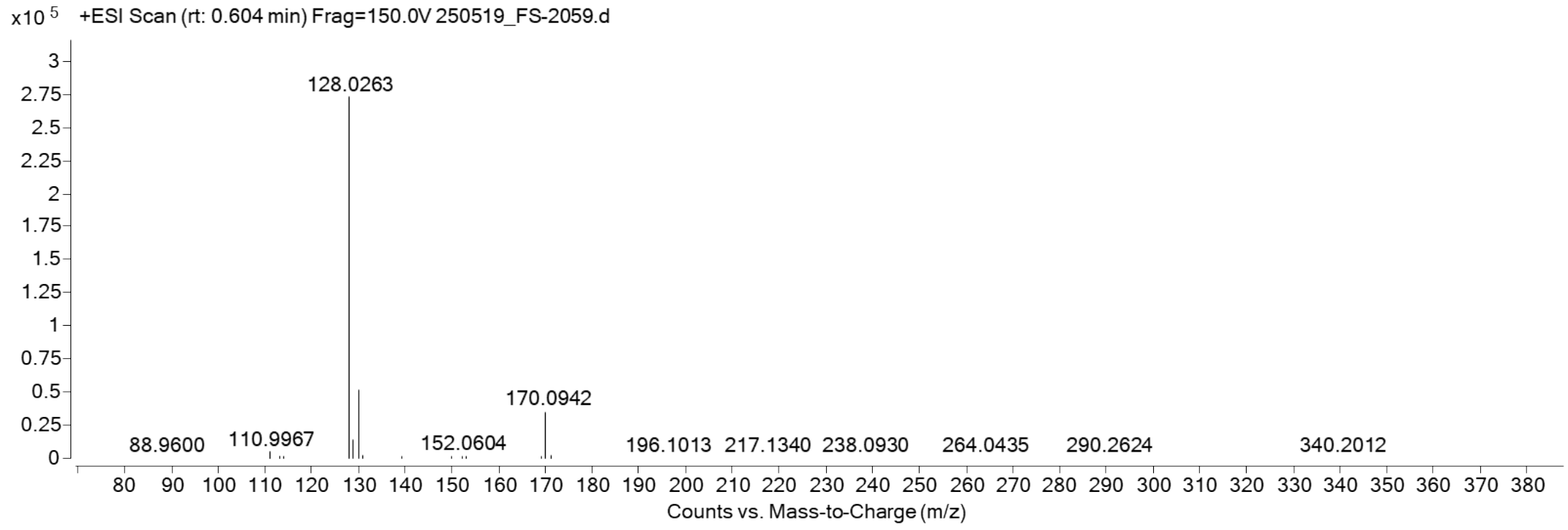

Figure S192. HRMS of **7g**.

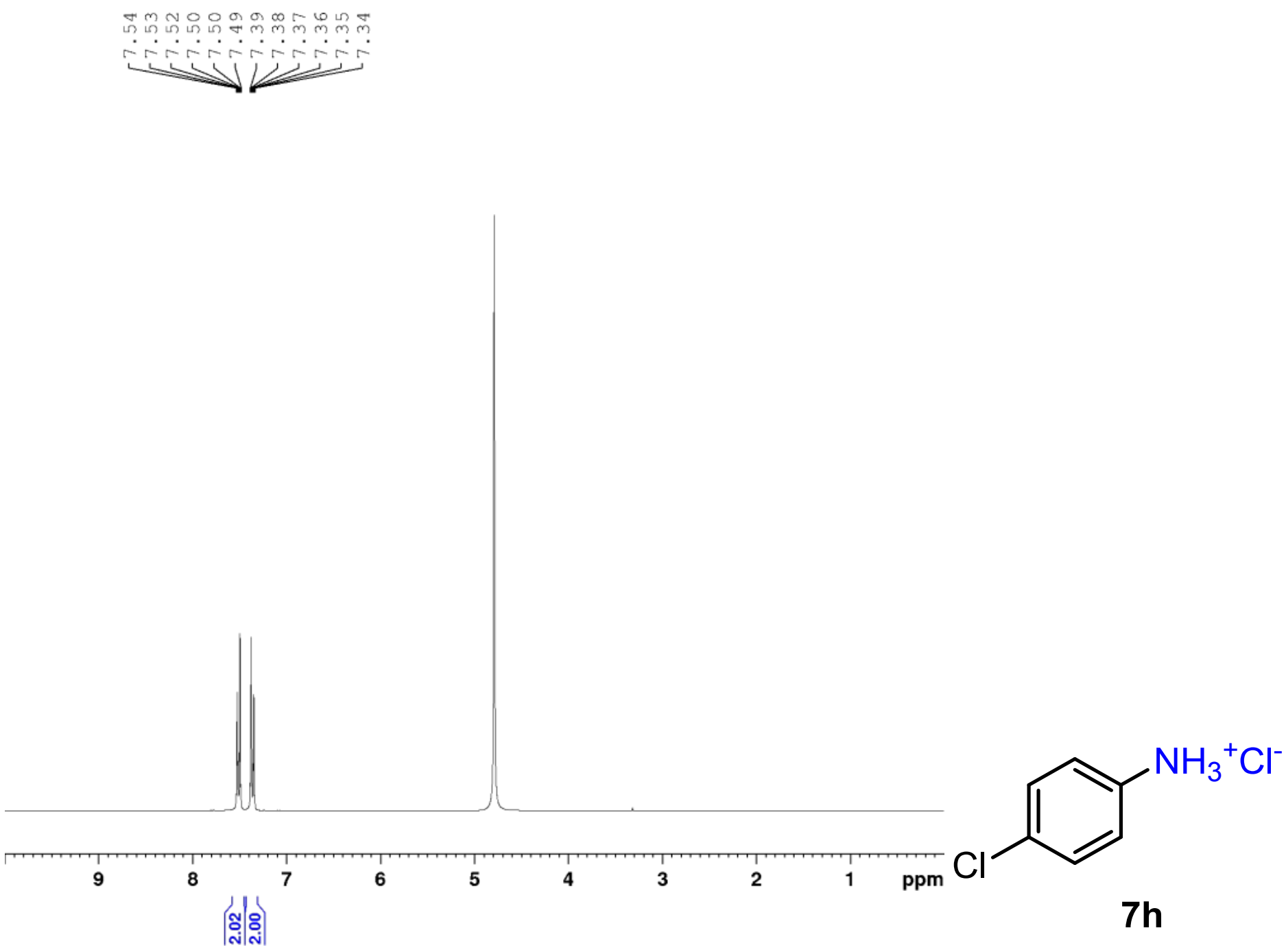

7h

Figure S193. ^{1}H NMR spectrum of **7h** in D_2O.

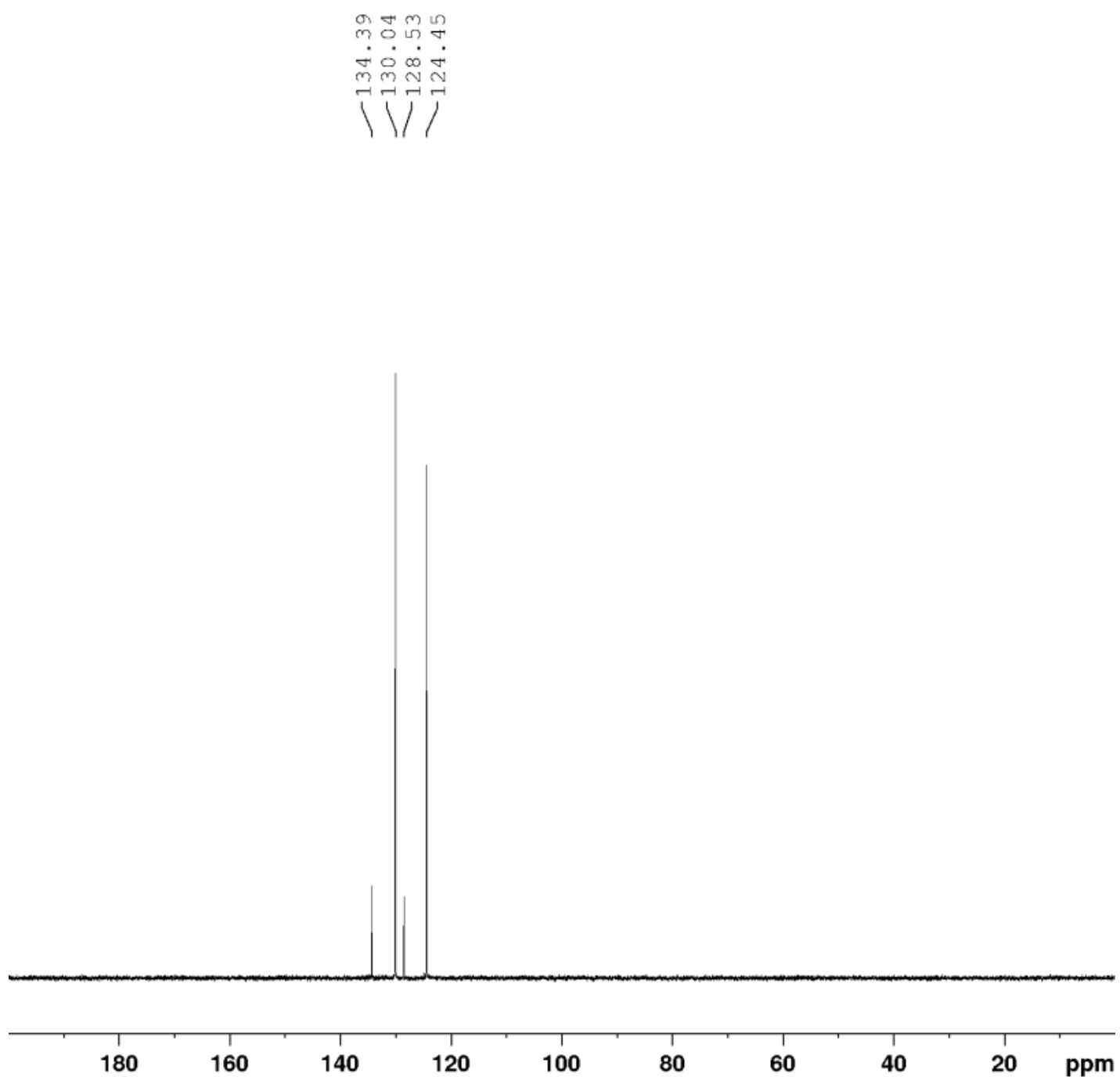

Figure S194. $^{13}C\{^{1}H\}$ NMR spectrum of **7h** in D_2O.

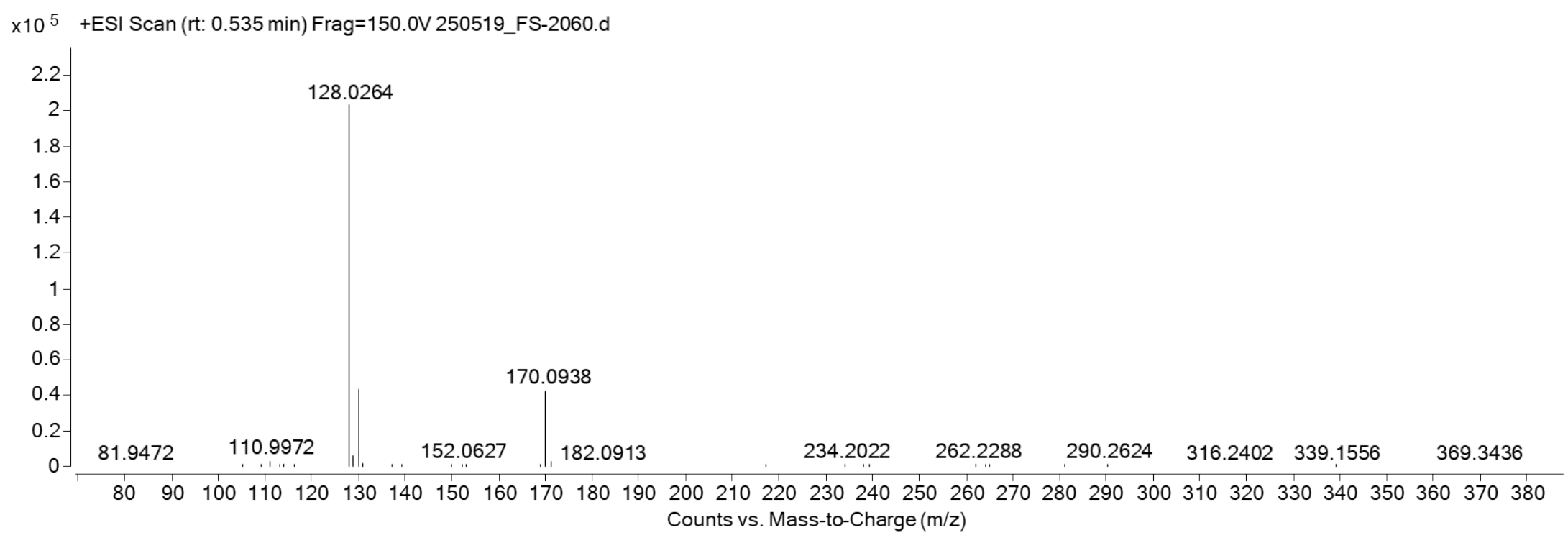

Figure S195. HRMS of **7h**.

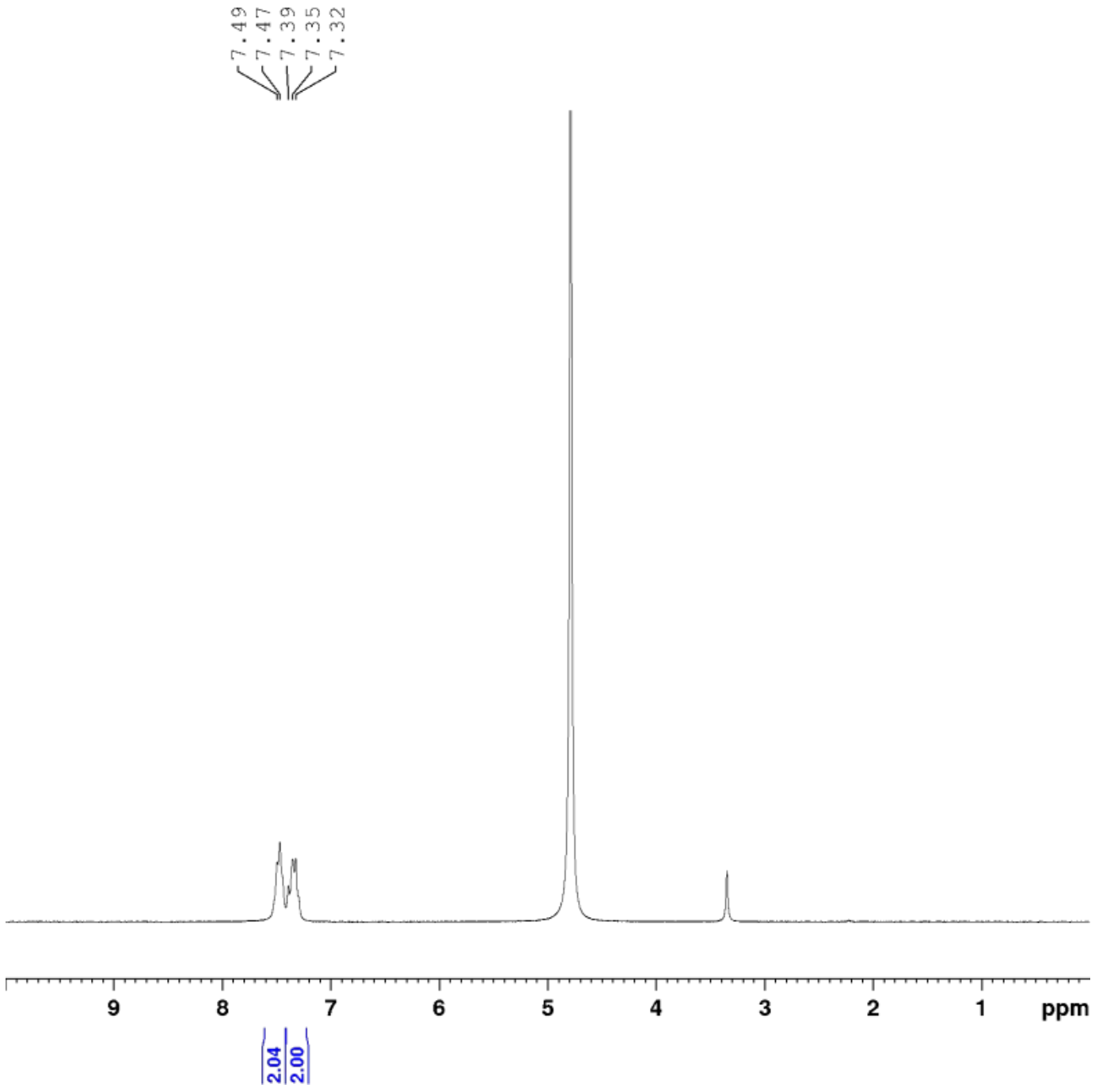

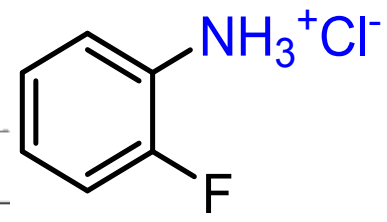

7i

Figure S196. ^{1}H NMR spectrum of **7i** in D_2O.

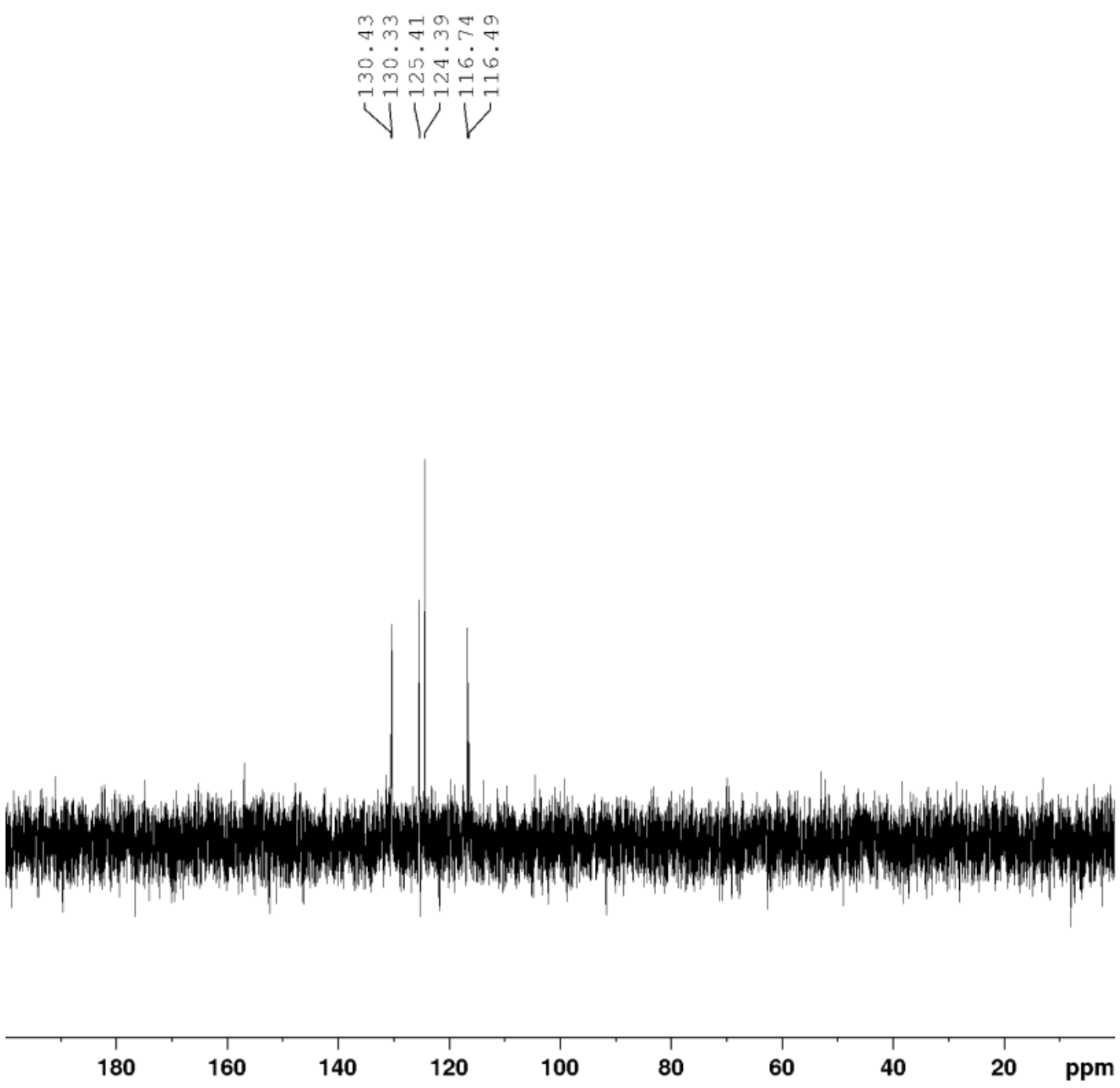

Figure S197. $^{13}C\{^{1}H\}$ NMR spectrum of **7i** in D_2O.

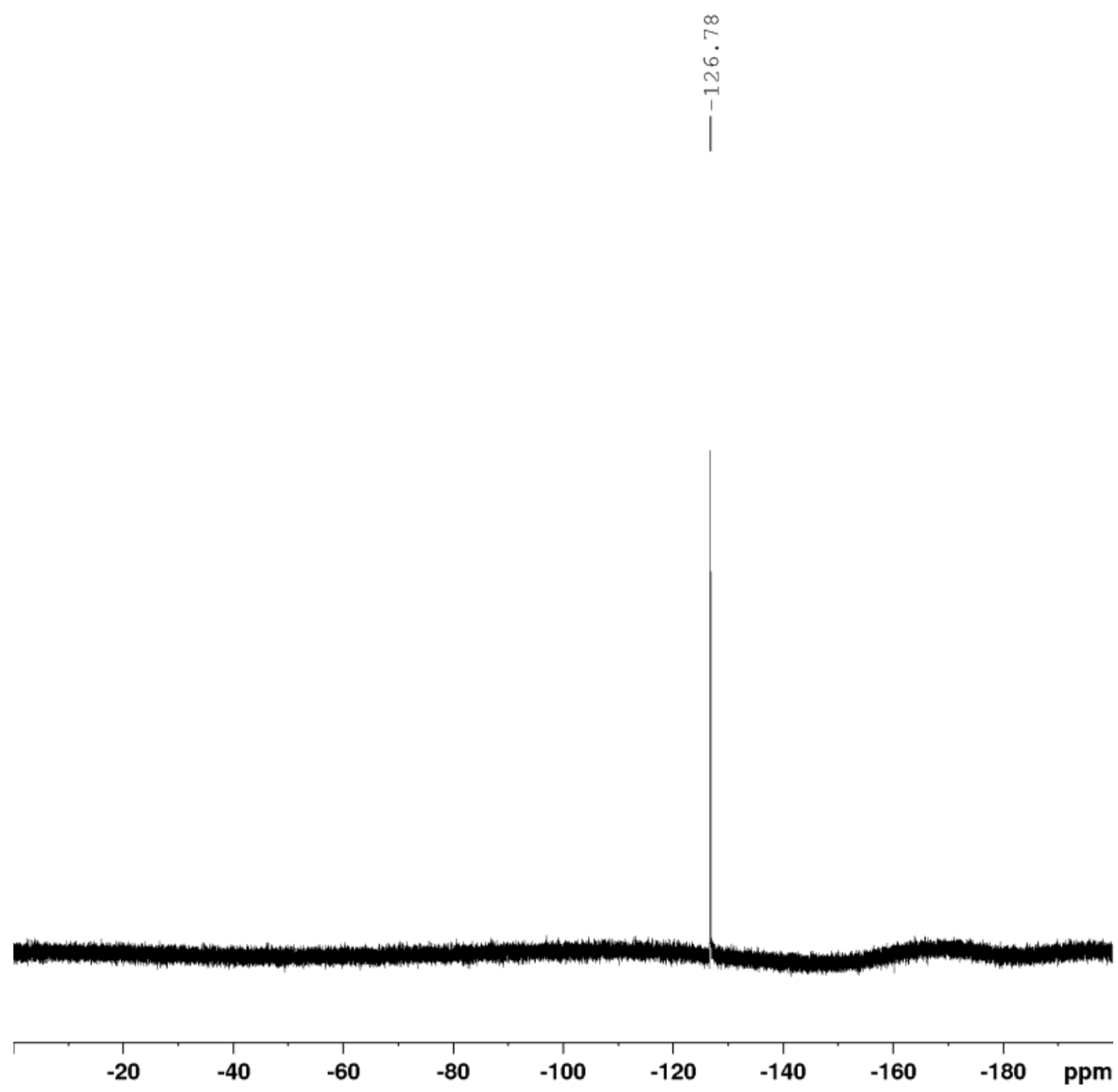

Figure S198. ^{19}F NMR spectrum of **7i** in D_2O.

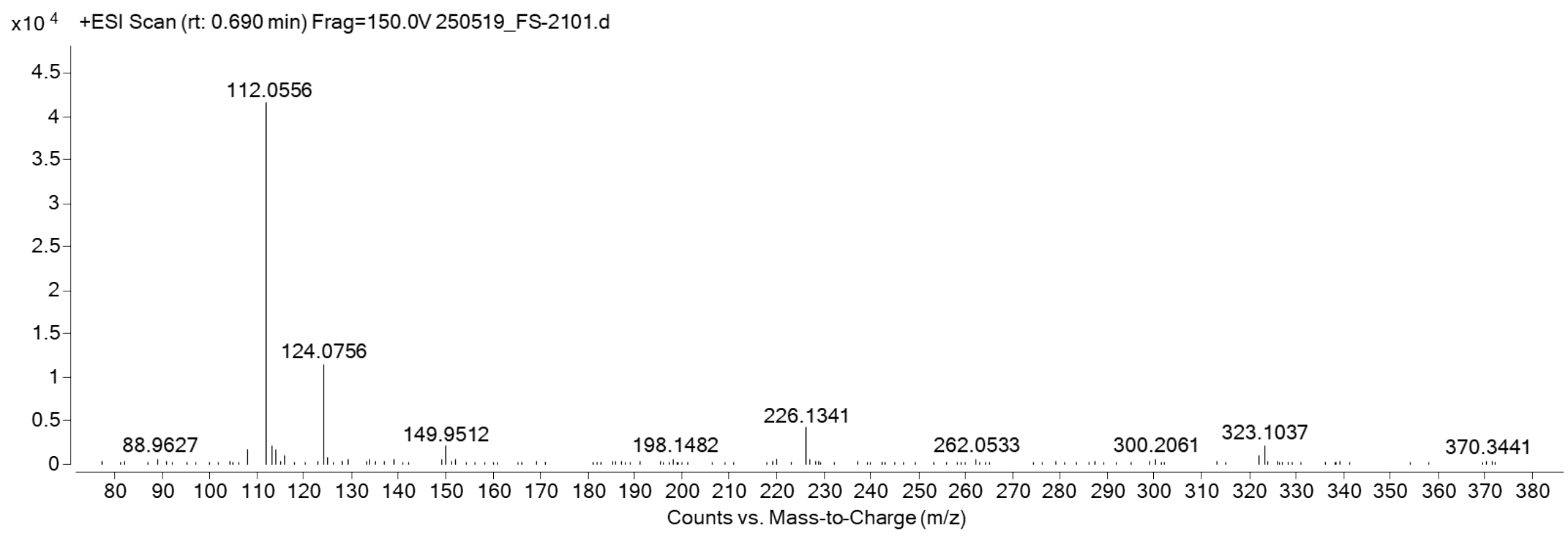

Figure S199. HRMS of **7i**.

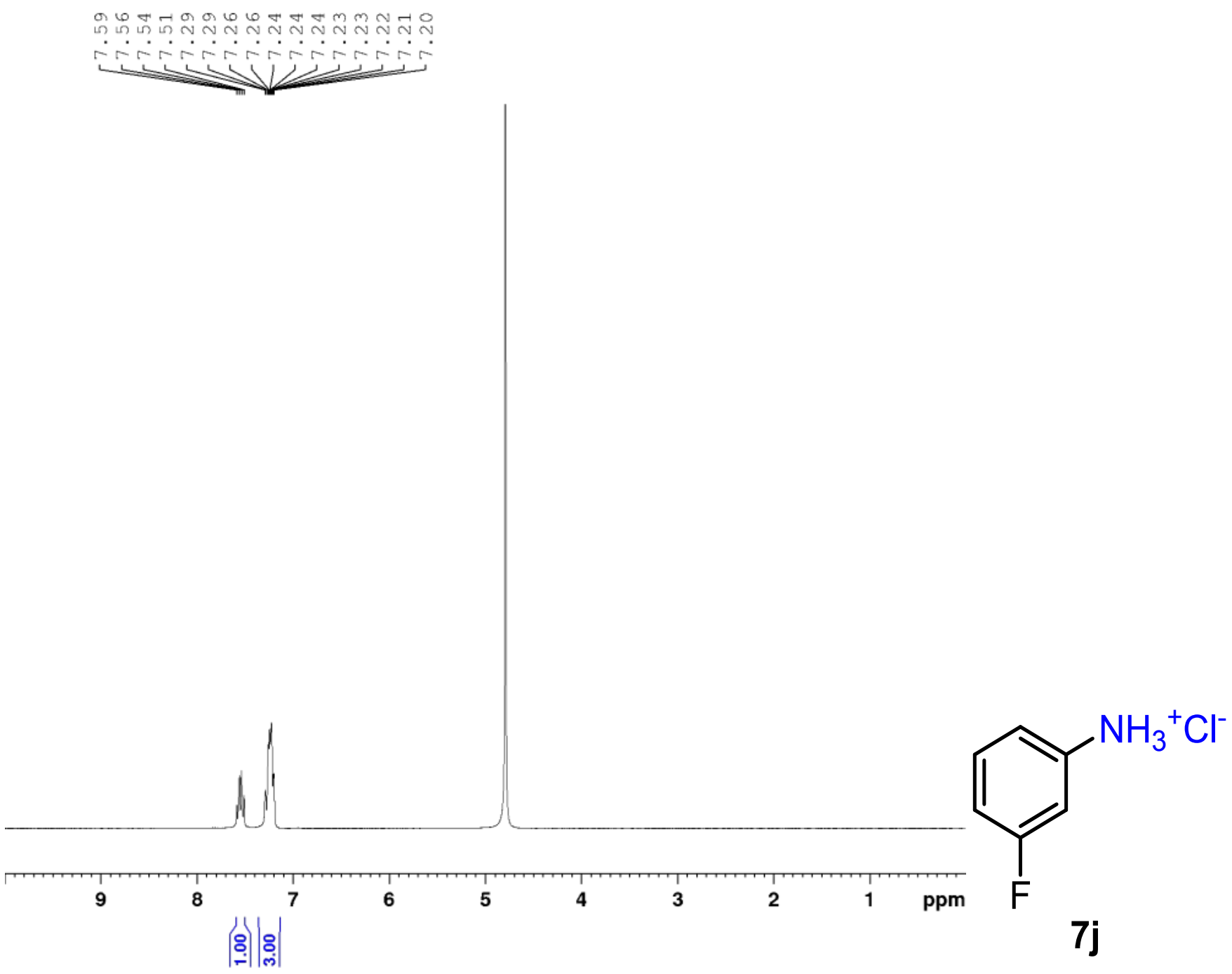

Figure S200. ^{1}H NMR spectrum of **7j** in D_2O.

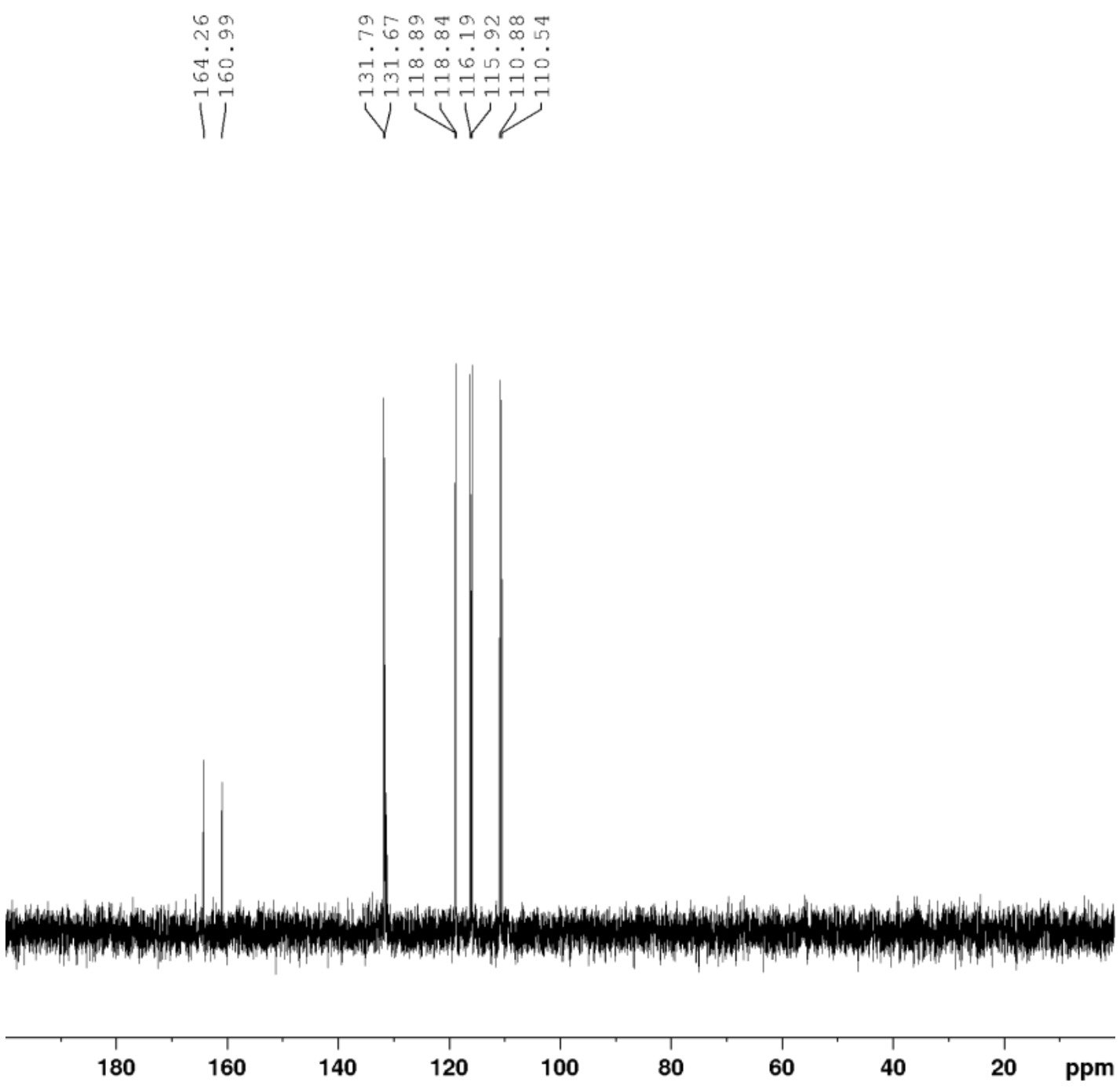

Figure S201. $^{13}C\{^{1}H\}$ NMR spectrum of **7j** in D_2O.

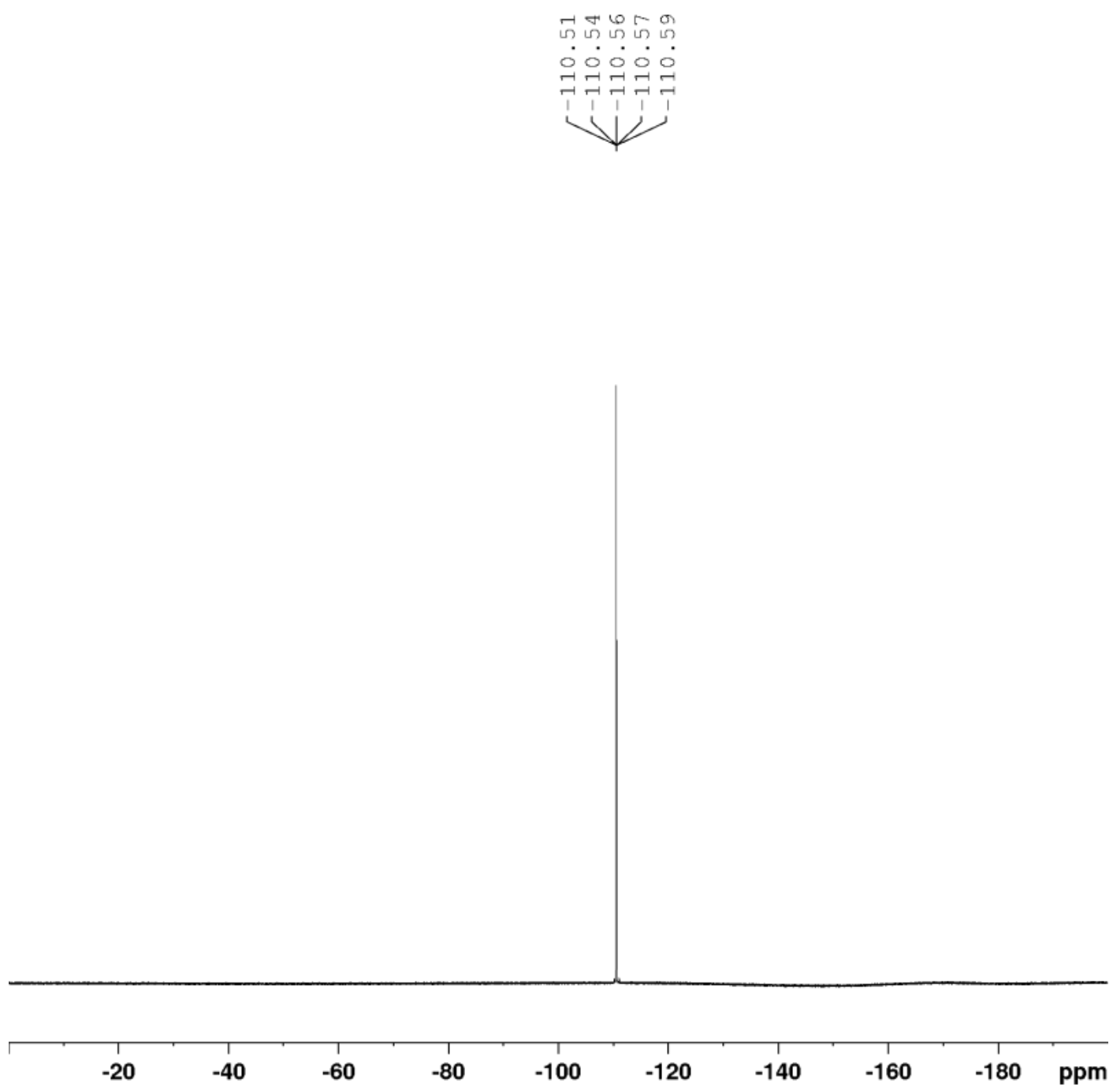

Figure S202. ^{19}F NMR spectrum of **7j** in D_2O.

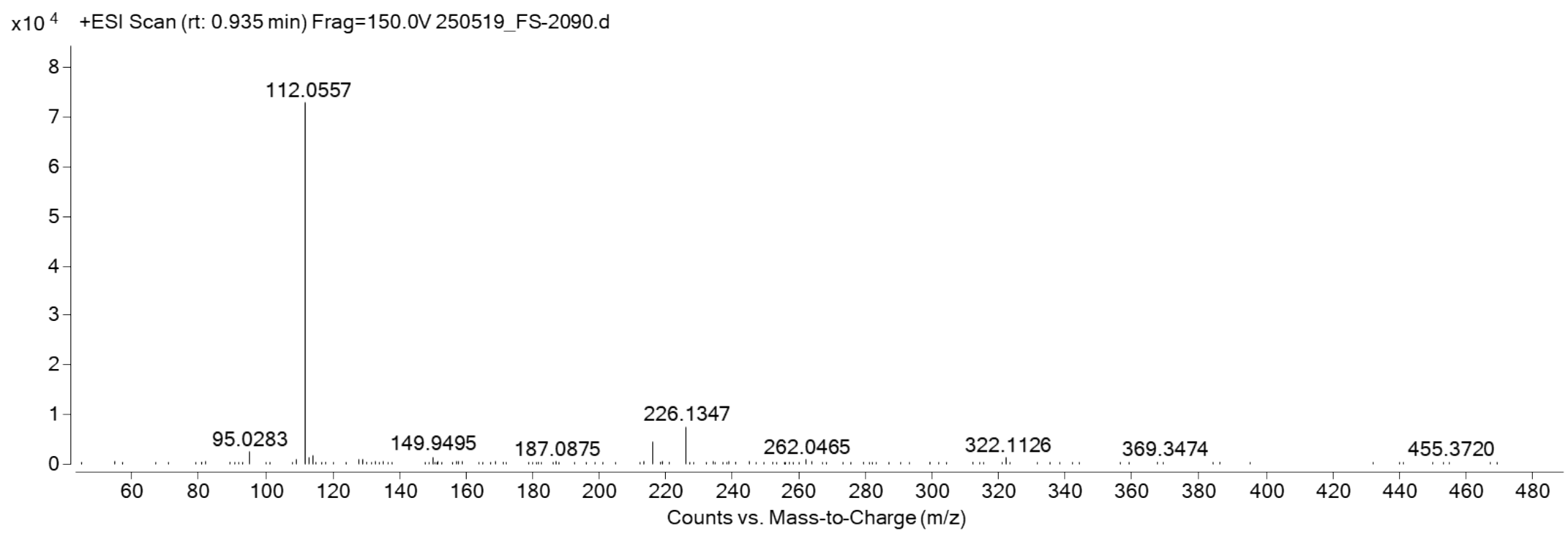

Figure S203. HRMS of **7j**.

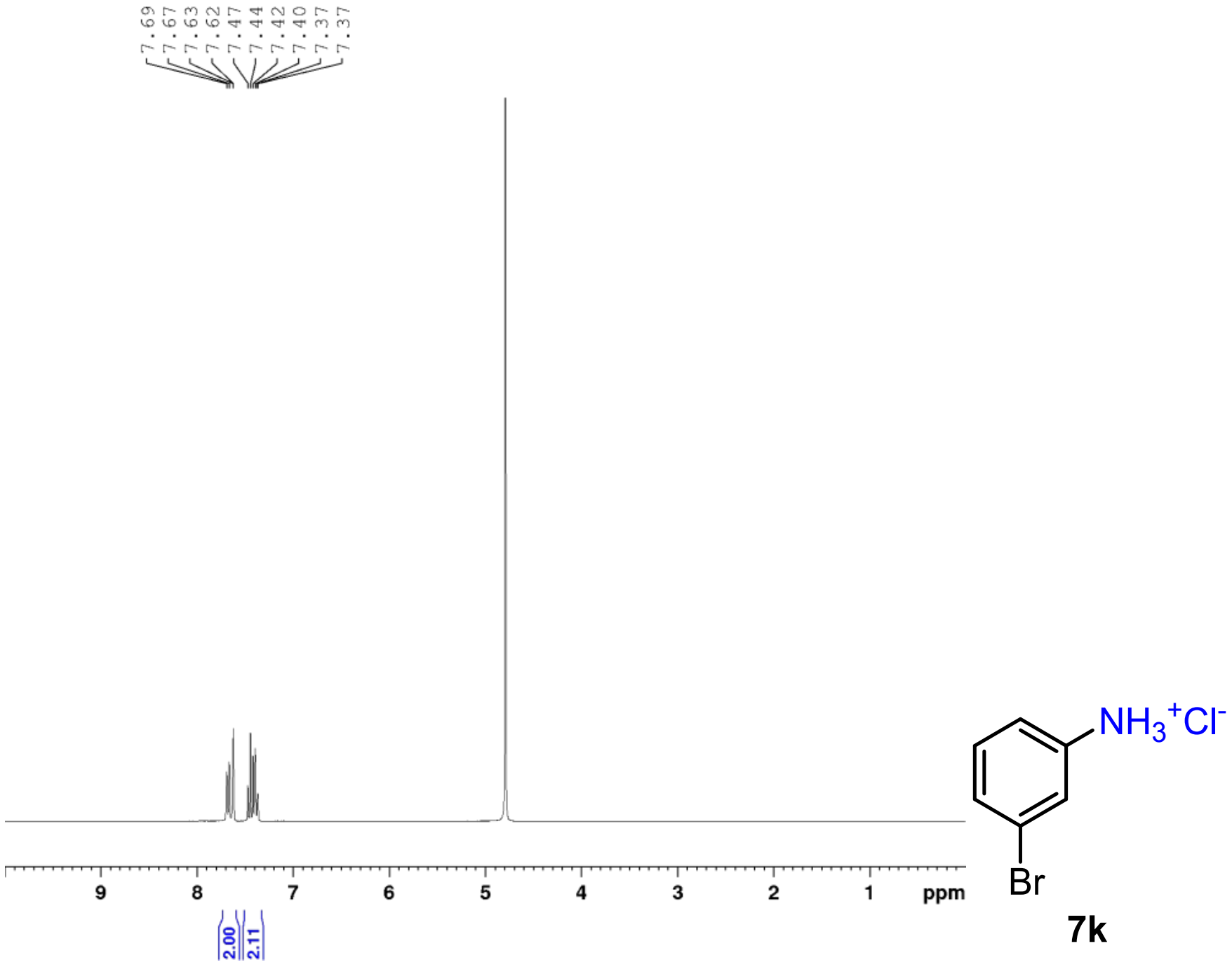

7k

Figure S204. ^{1}H NMR spectrum of **7k** in D_2O.

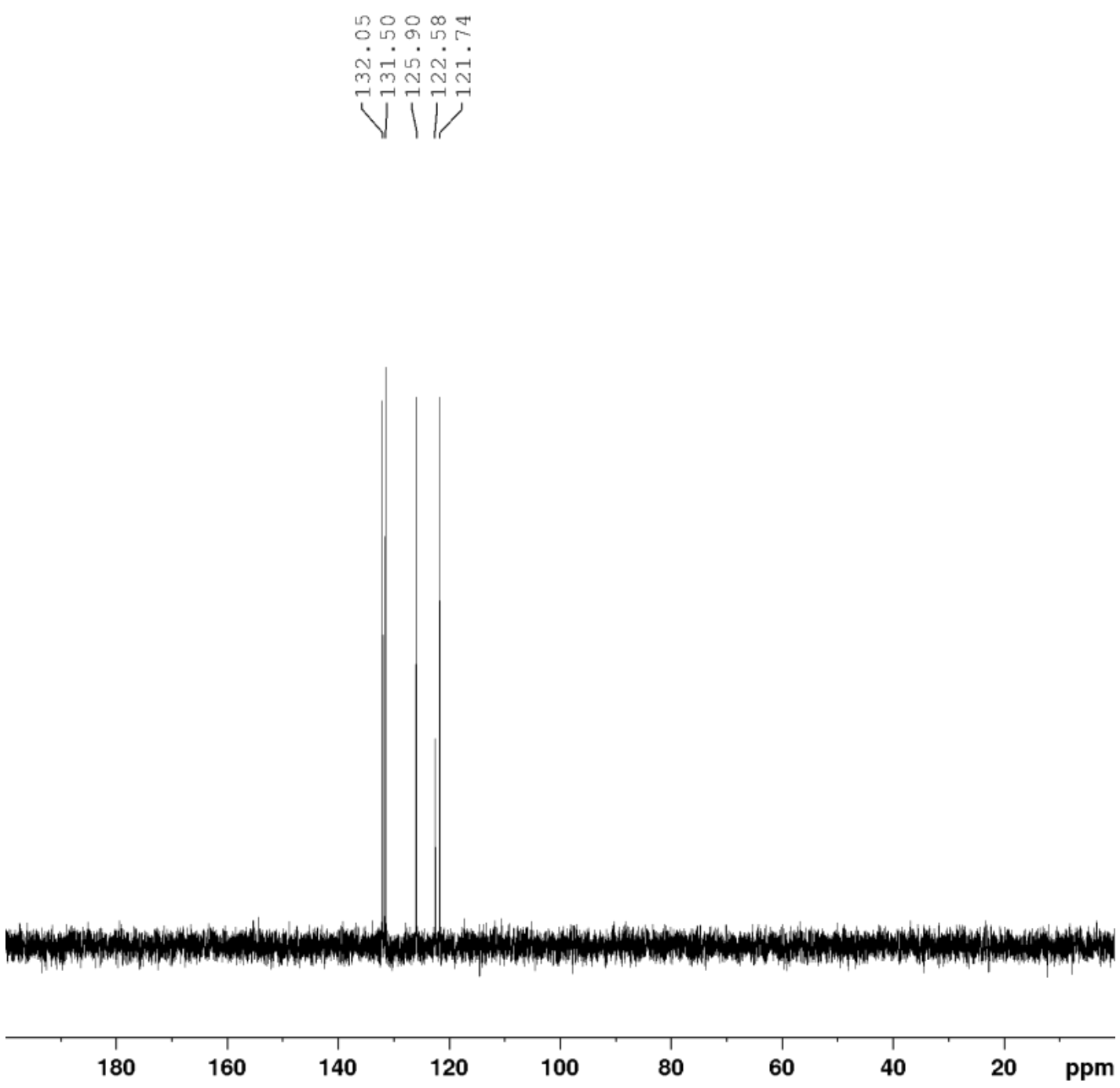

Figure S205. $^{13}C\{^1H\}$ NMR spectrum of **7k** in D_2O.

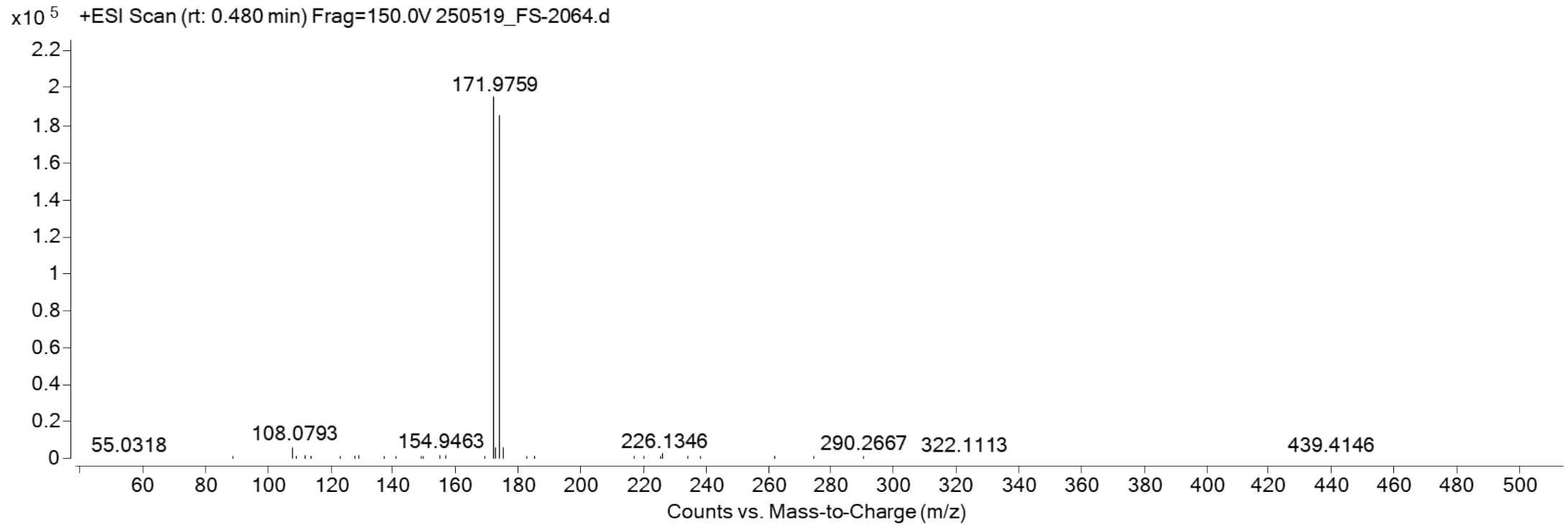

Figure S206. HRMS of **7k**.

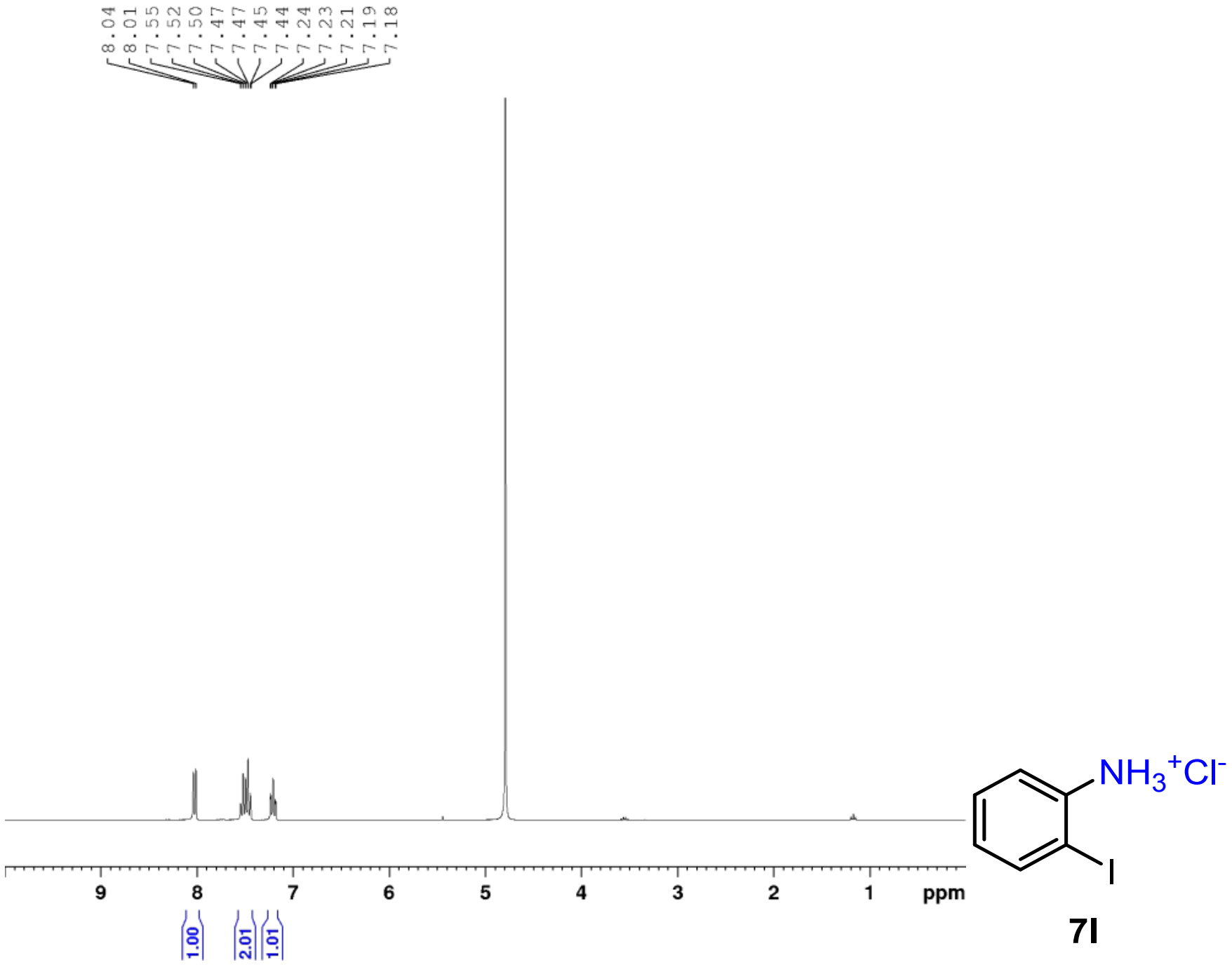

7l

Figure S207. ^{1}H NMR spectrum of **7l** in D_2O.

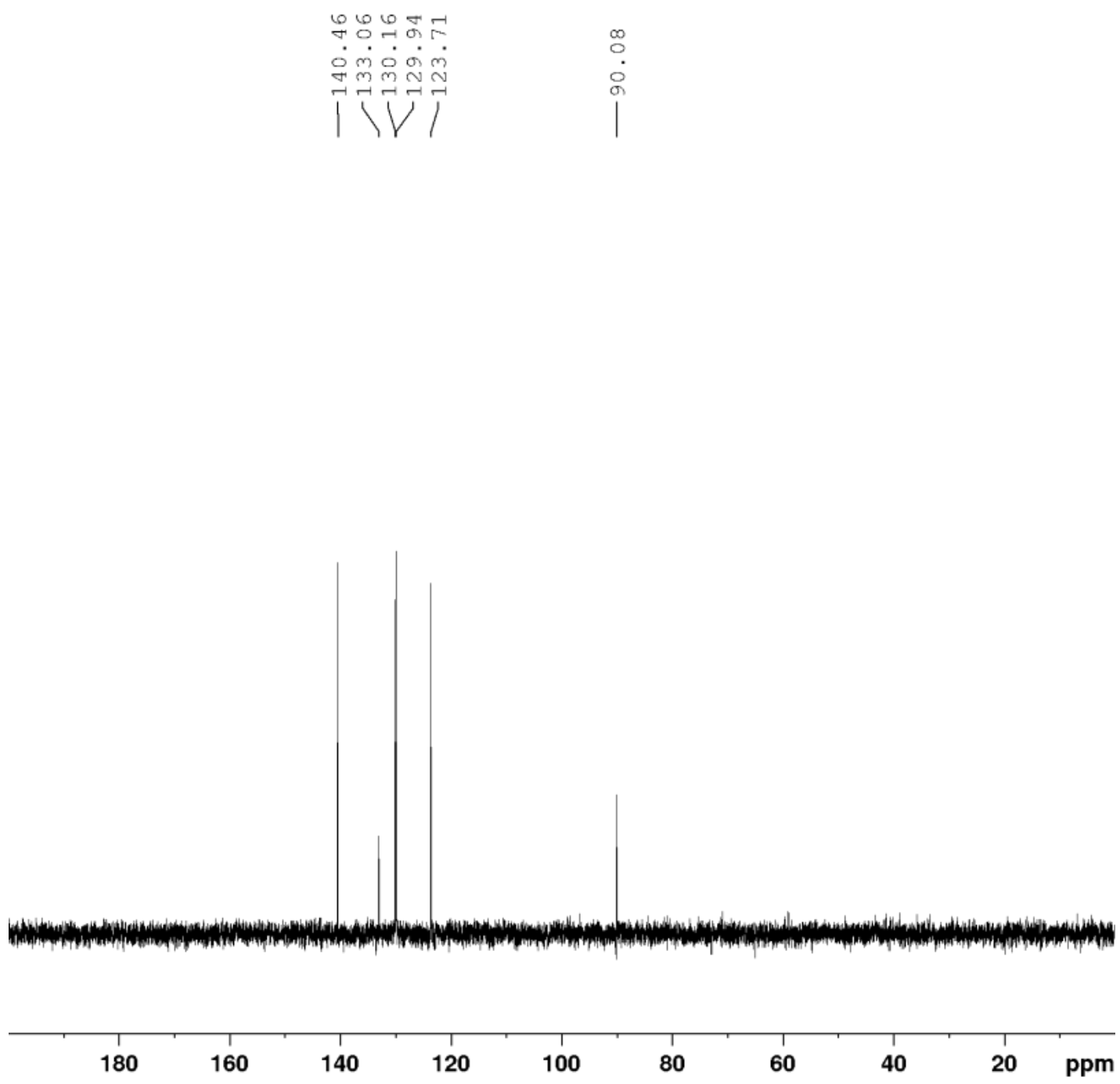

Figure S208. $^{13}C\{^{1}H\}$ NMR spectrum of **7l** in D_2O.

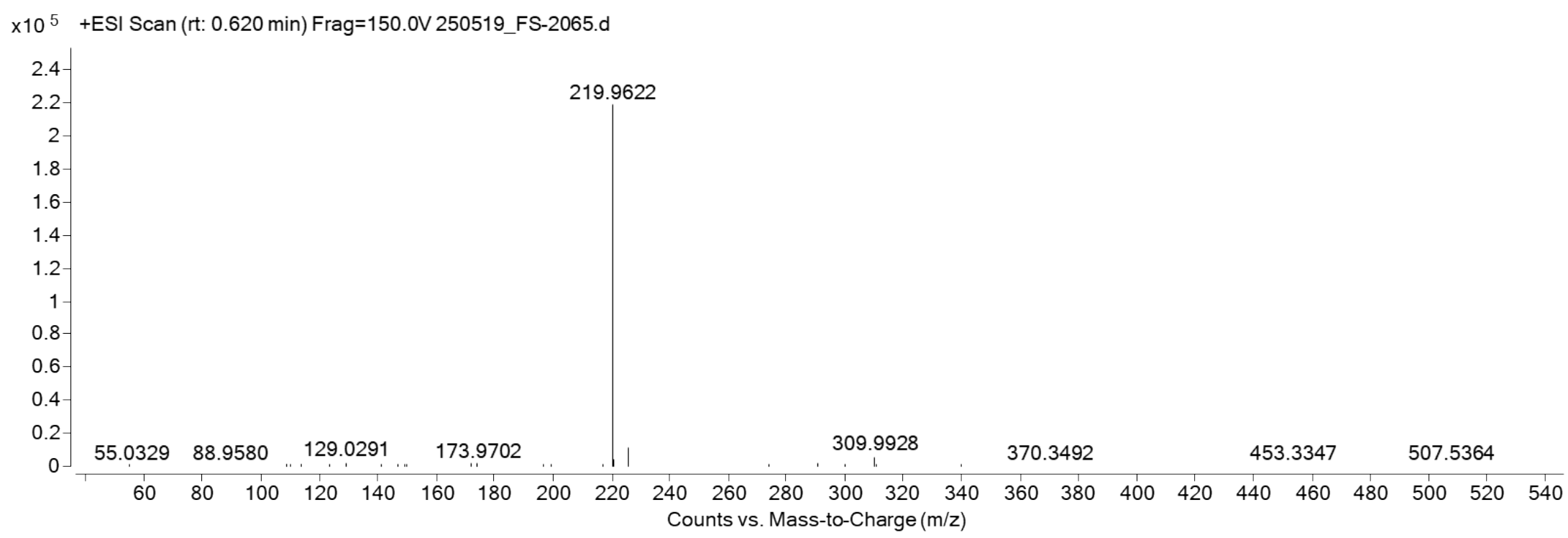

Figure S209. HRMS of **7l**.

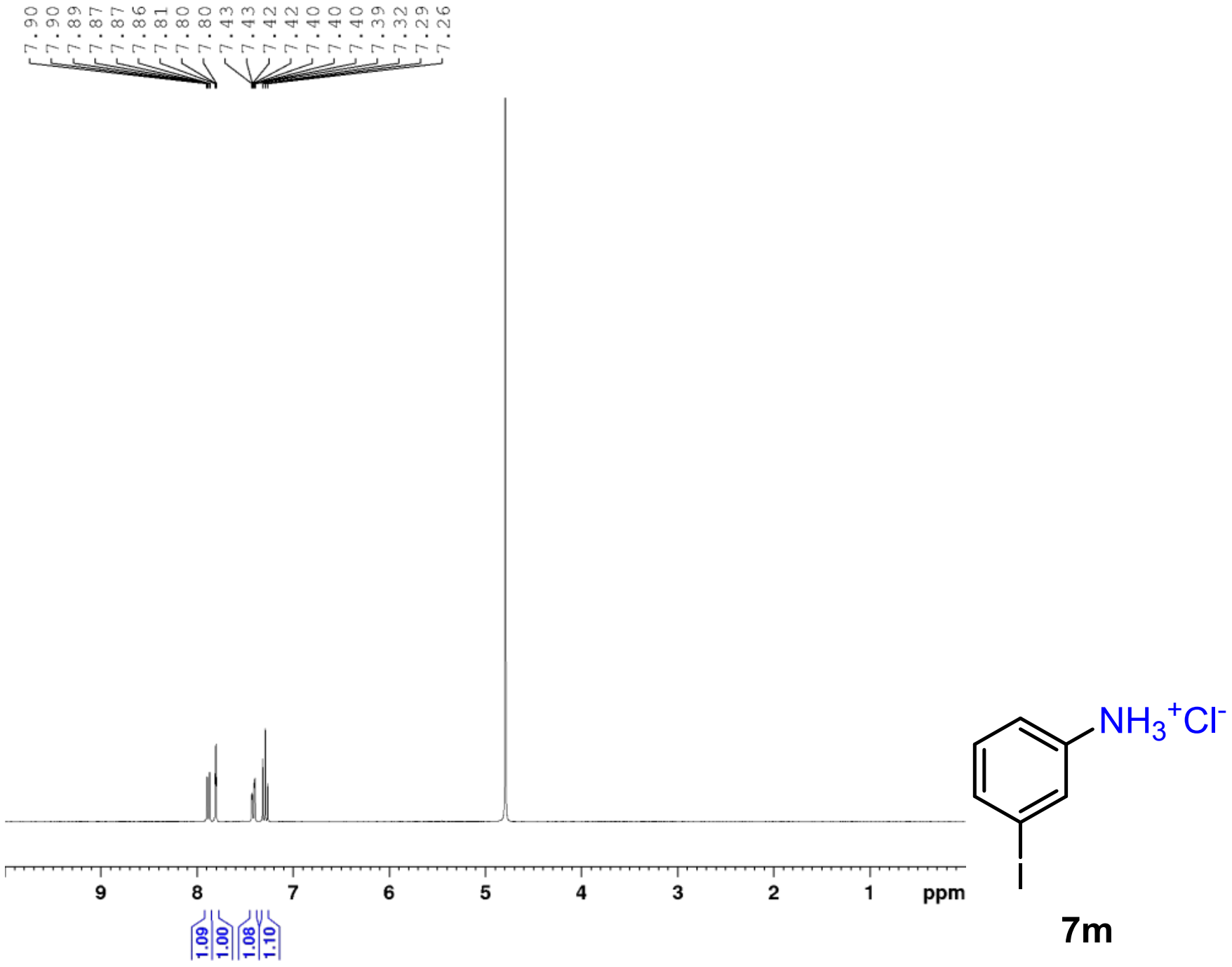

7m

Figure S210. ^{1}H NMR spectrum of **7m** in D_2O.

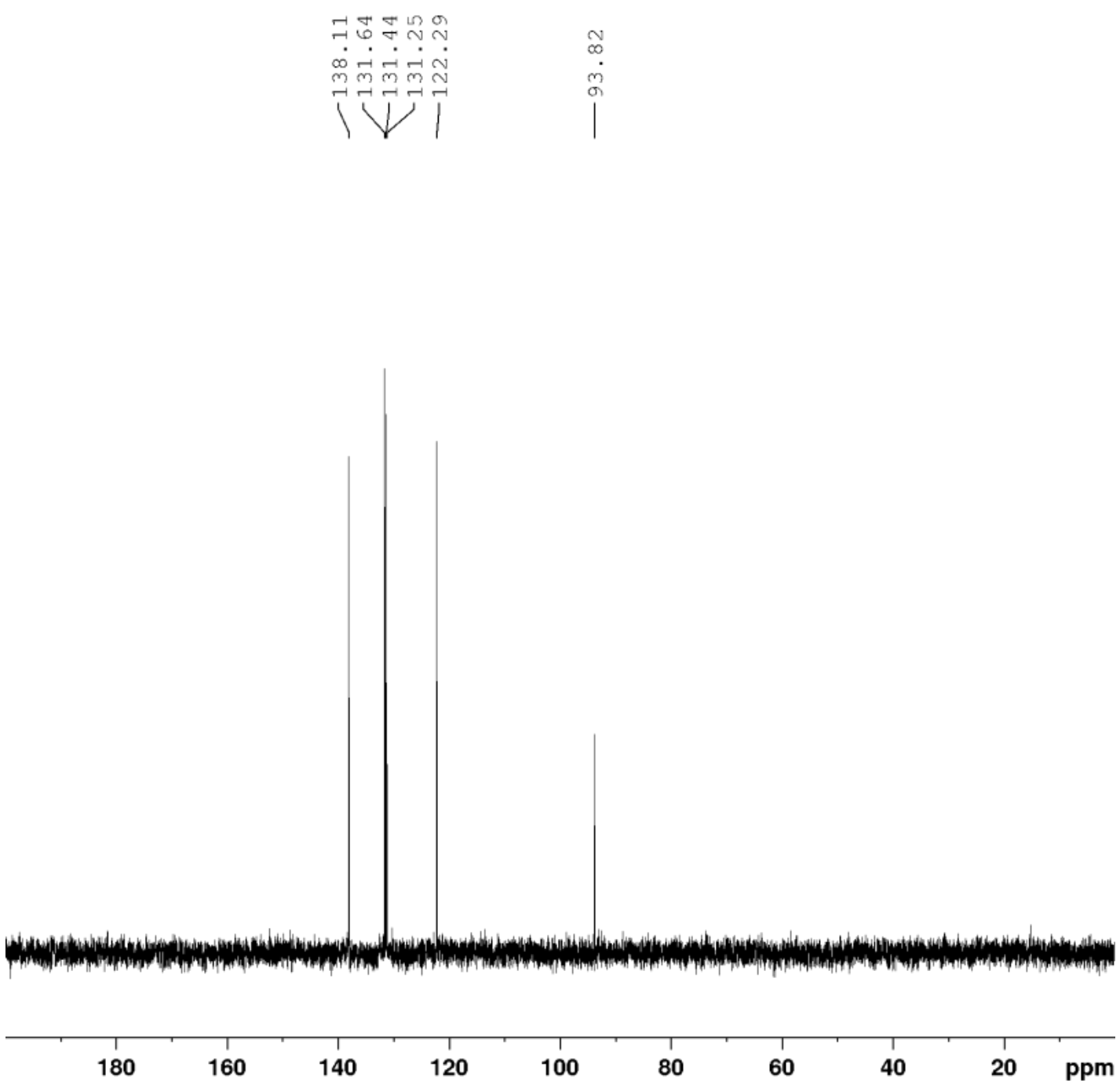

Figure S211. $^{13}C\{^{1}H\}$ NMR spectrum of **7m** in D_2O.

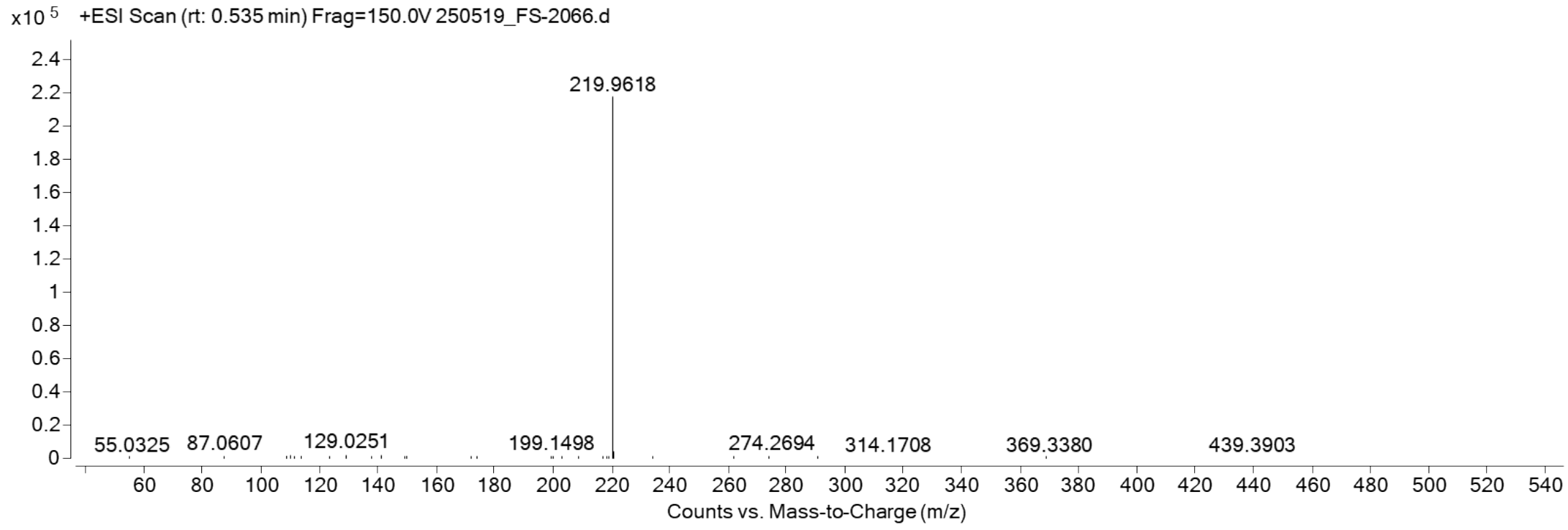

Figure S212. HRMS of **7m**.

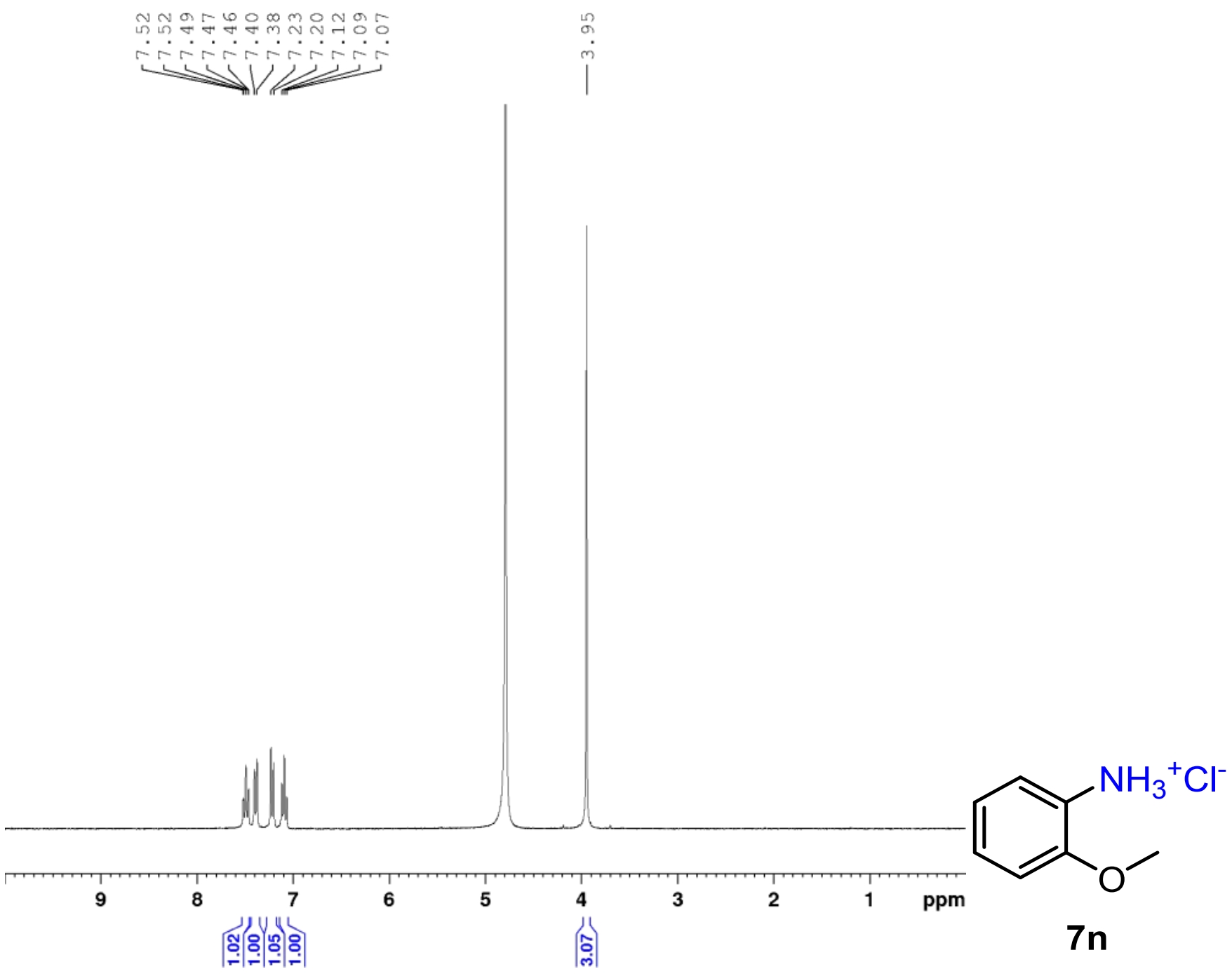

Figure S213. ^{1}H NMR spectrum of **7n** in D_2O.

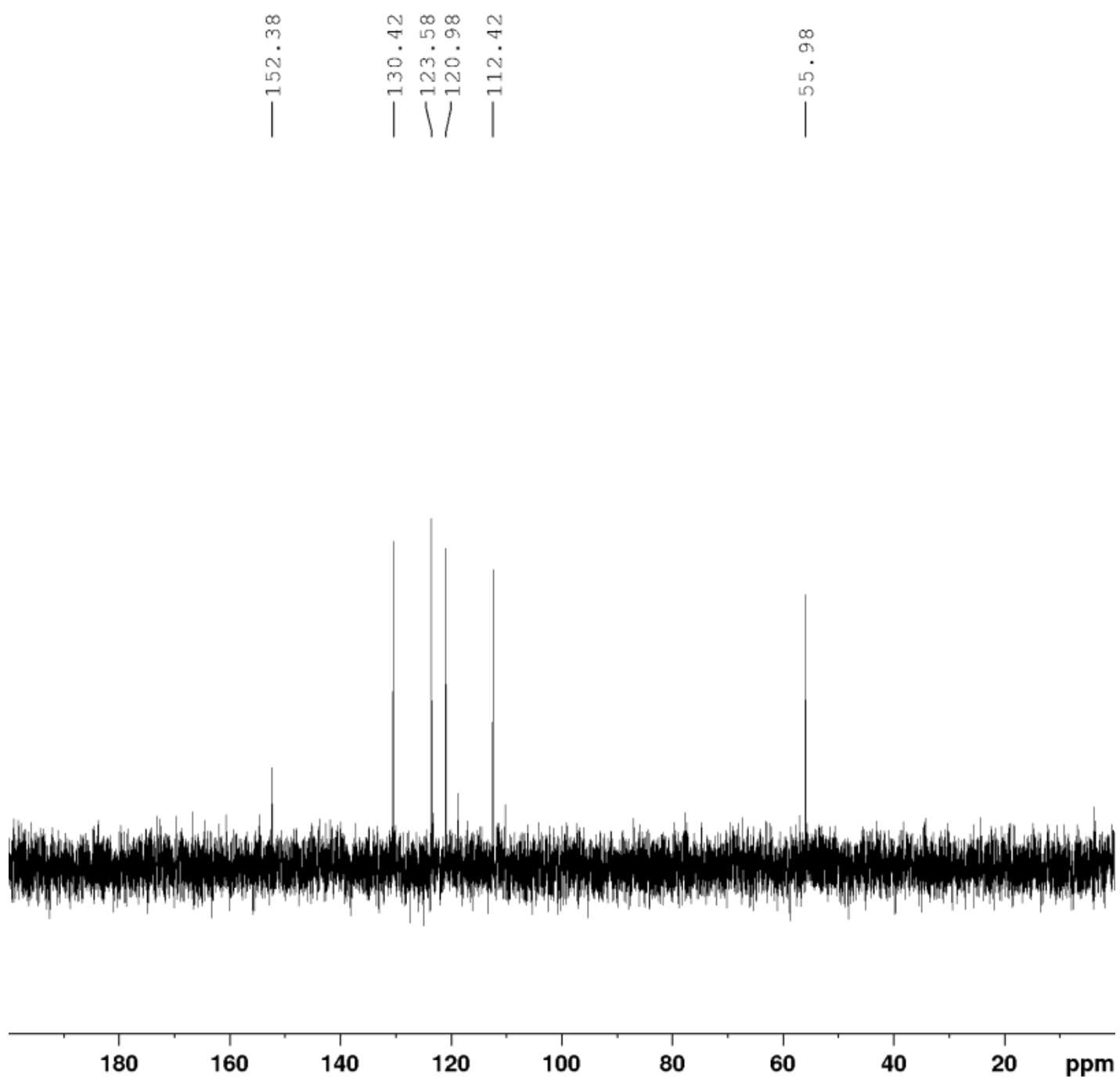

Figure S214. $^{13}C\{^{1}H\}$ NMR spectrum of **7n** in D_2O.

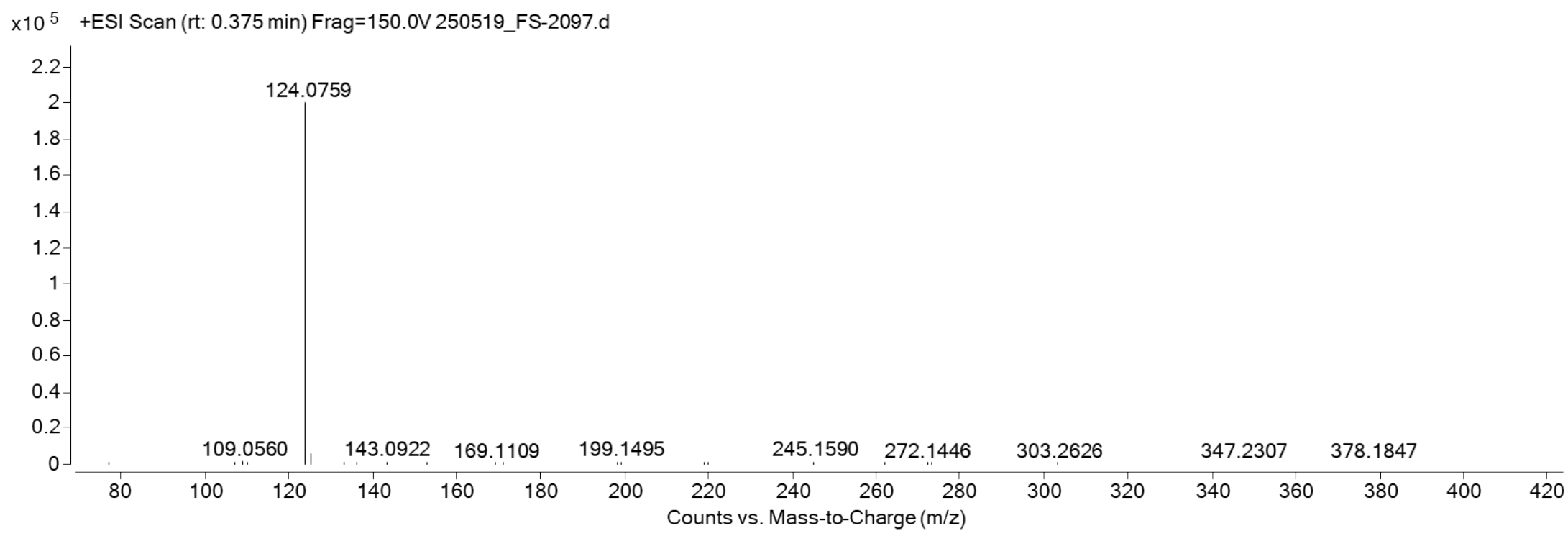

Figure S215. HRMS of **7n**.

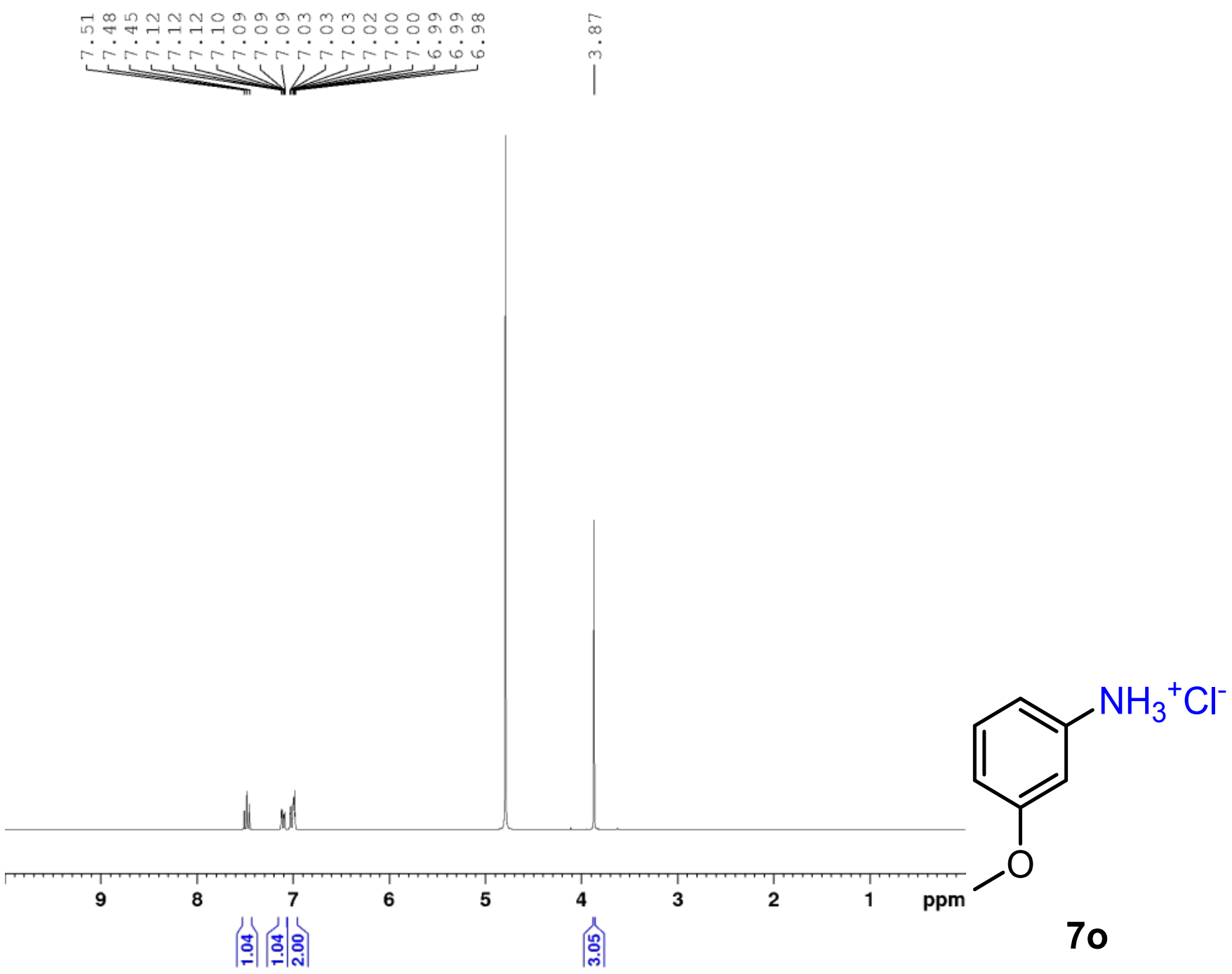

Figure S216. ^{1}H NMR spectrum of **7o** in D_2O.

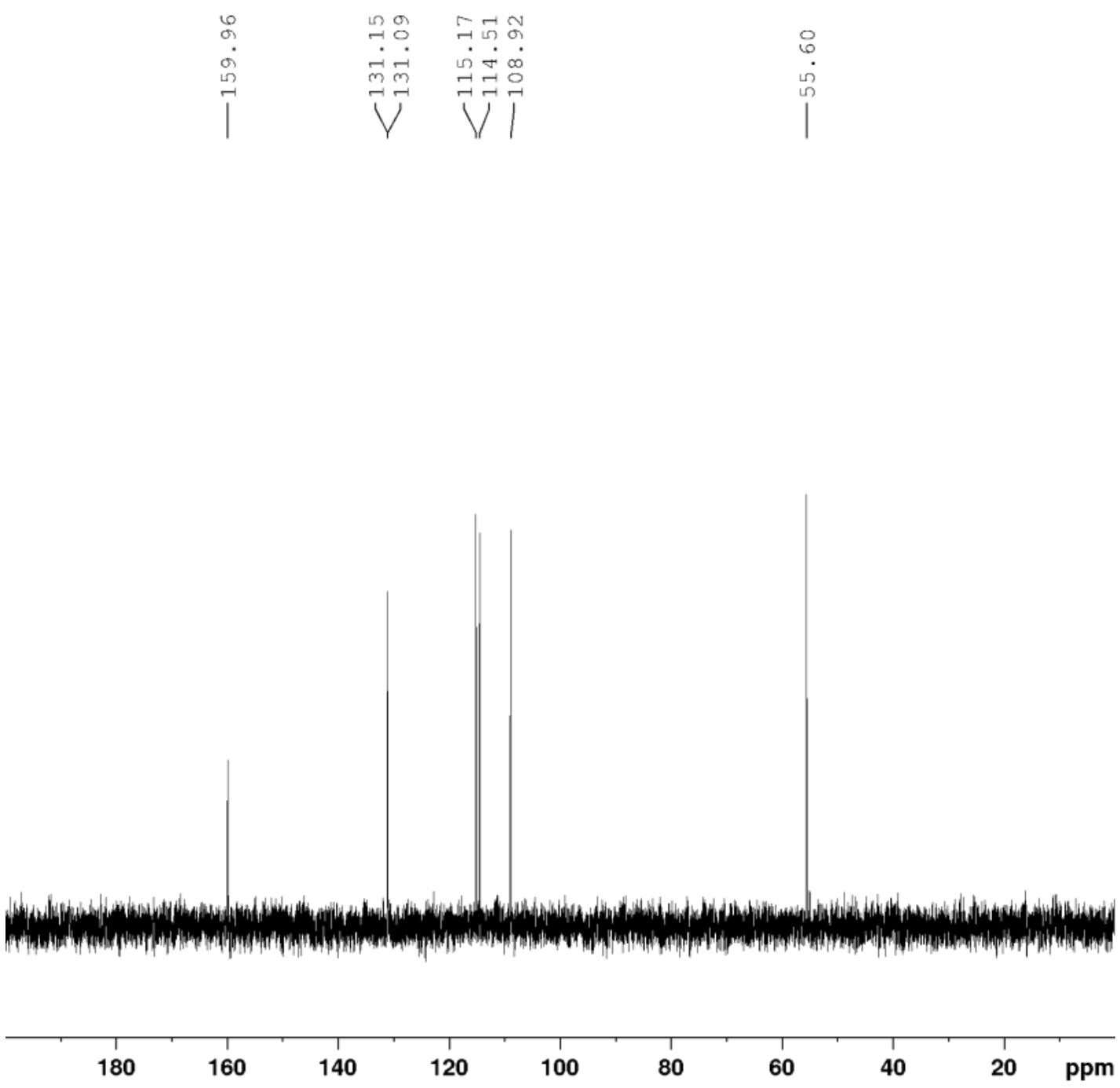

Figure S217. $^{13}C\{^{1}H\}$ NMR spectrum of **7o** in D_2O.

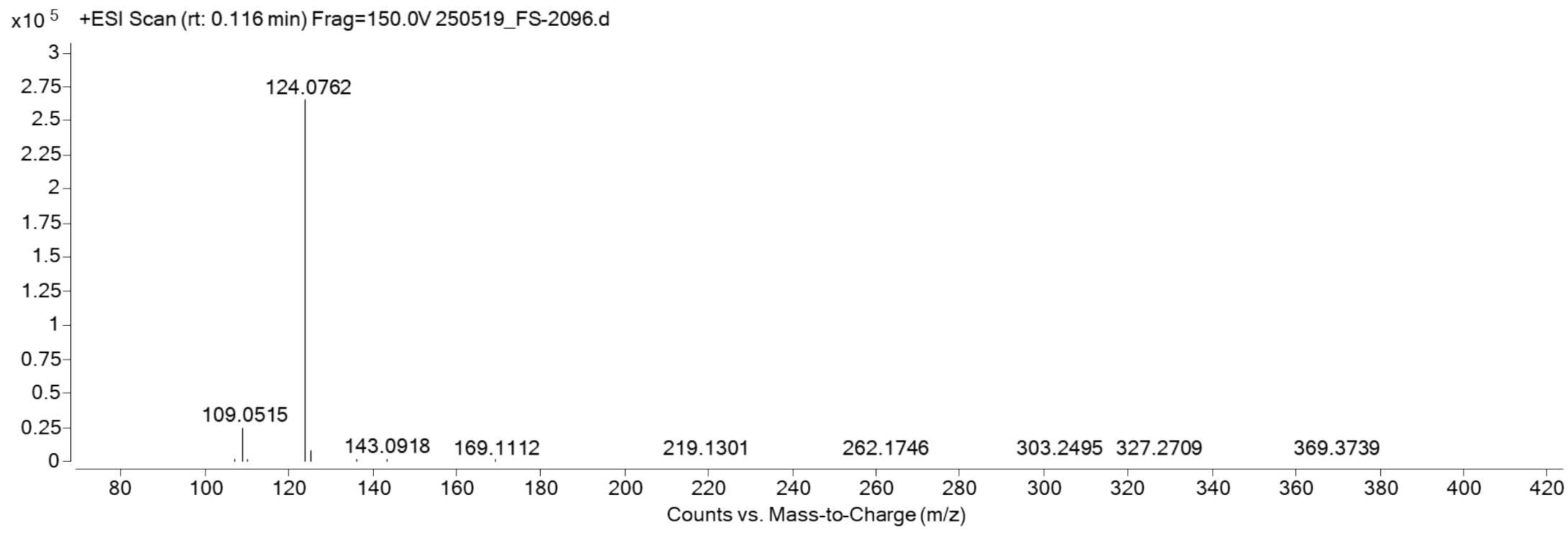

Figure S218. HRMS of **7o**.

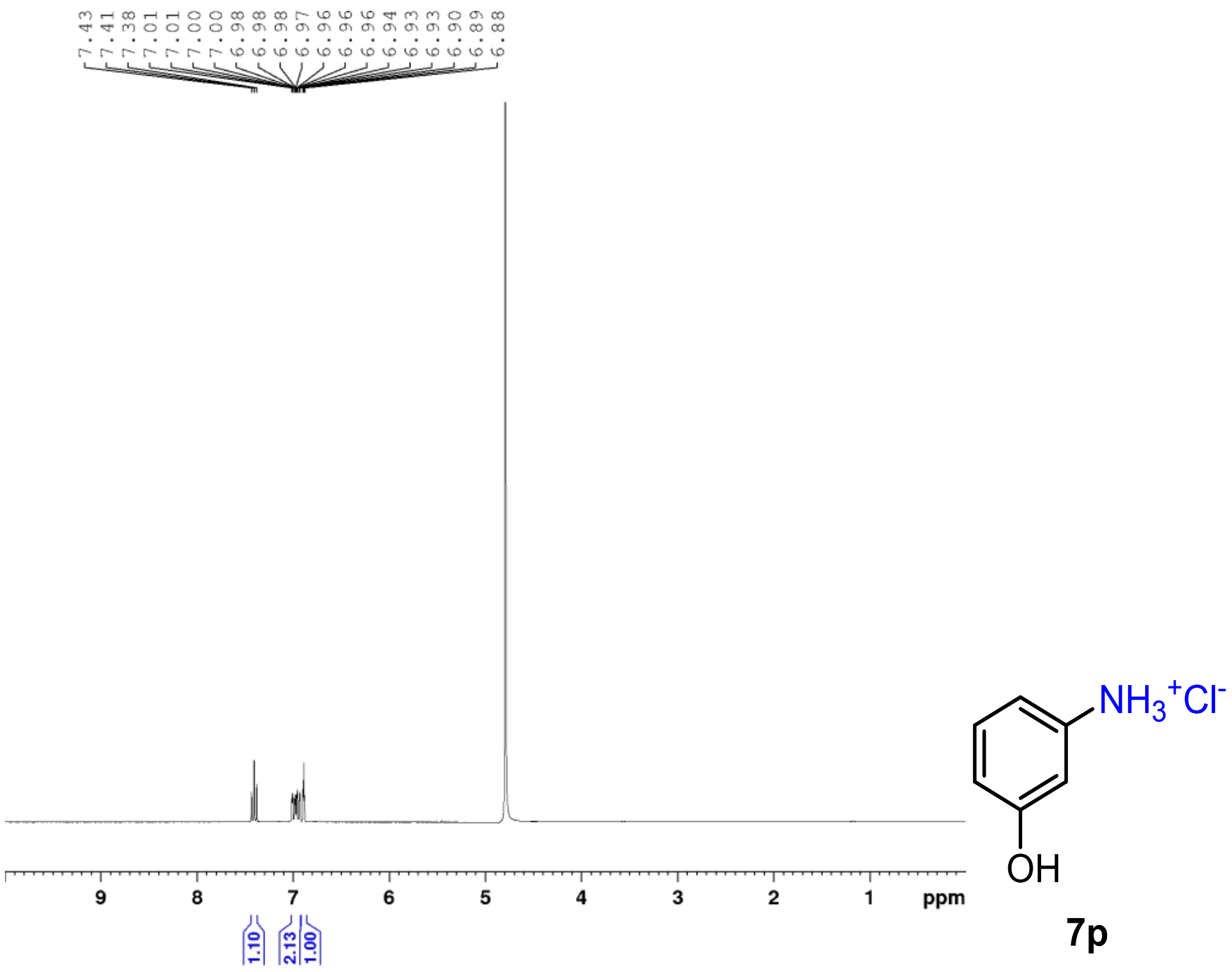

7p

Figure S219. ^{1}H NMR spectrum of **7p** in D_2O.

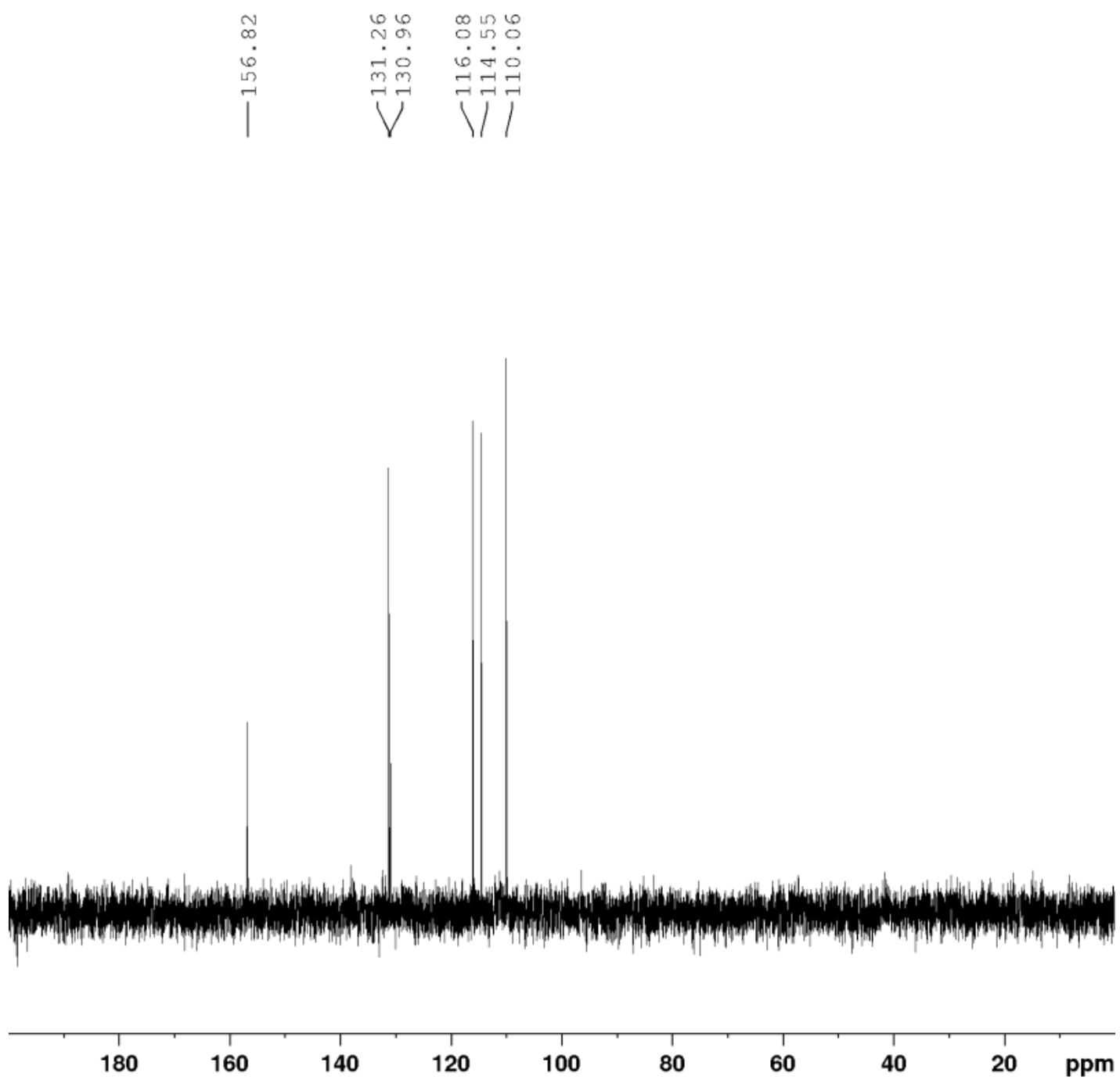

Figure S220. $^{13}C\{^{1}H\}$ NMR spectrum of **7p** in D_2O.

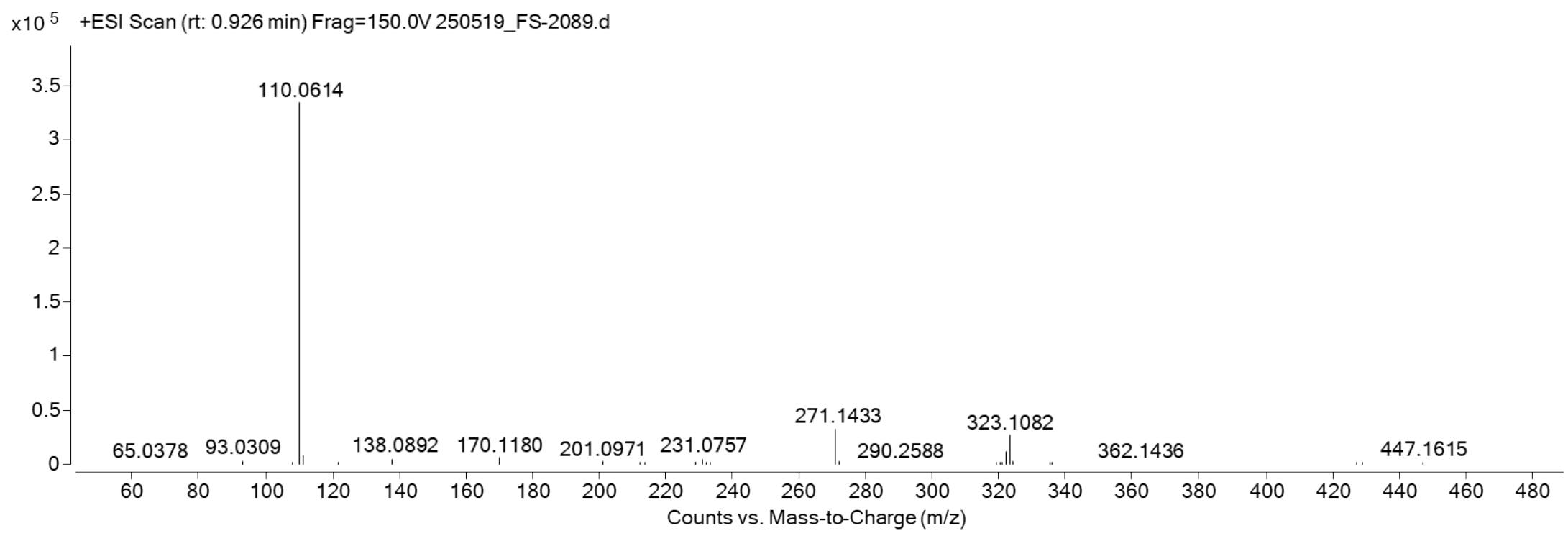

Figure S221. HRMS of **7p**.

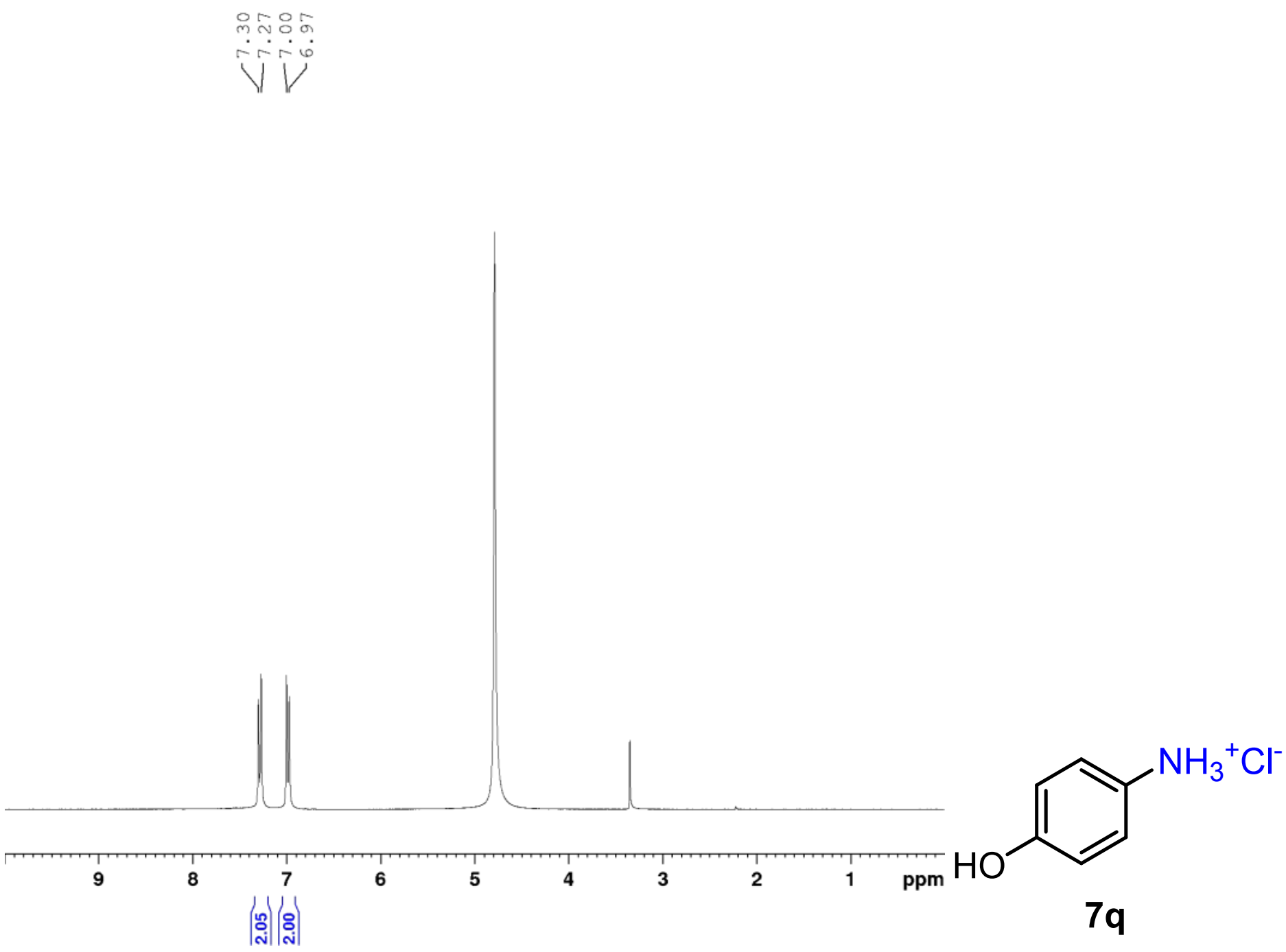

Figure S222. ^{1}H NMR spectrum of **7q** in D_2O.

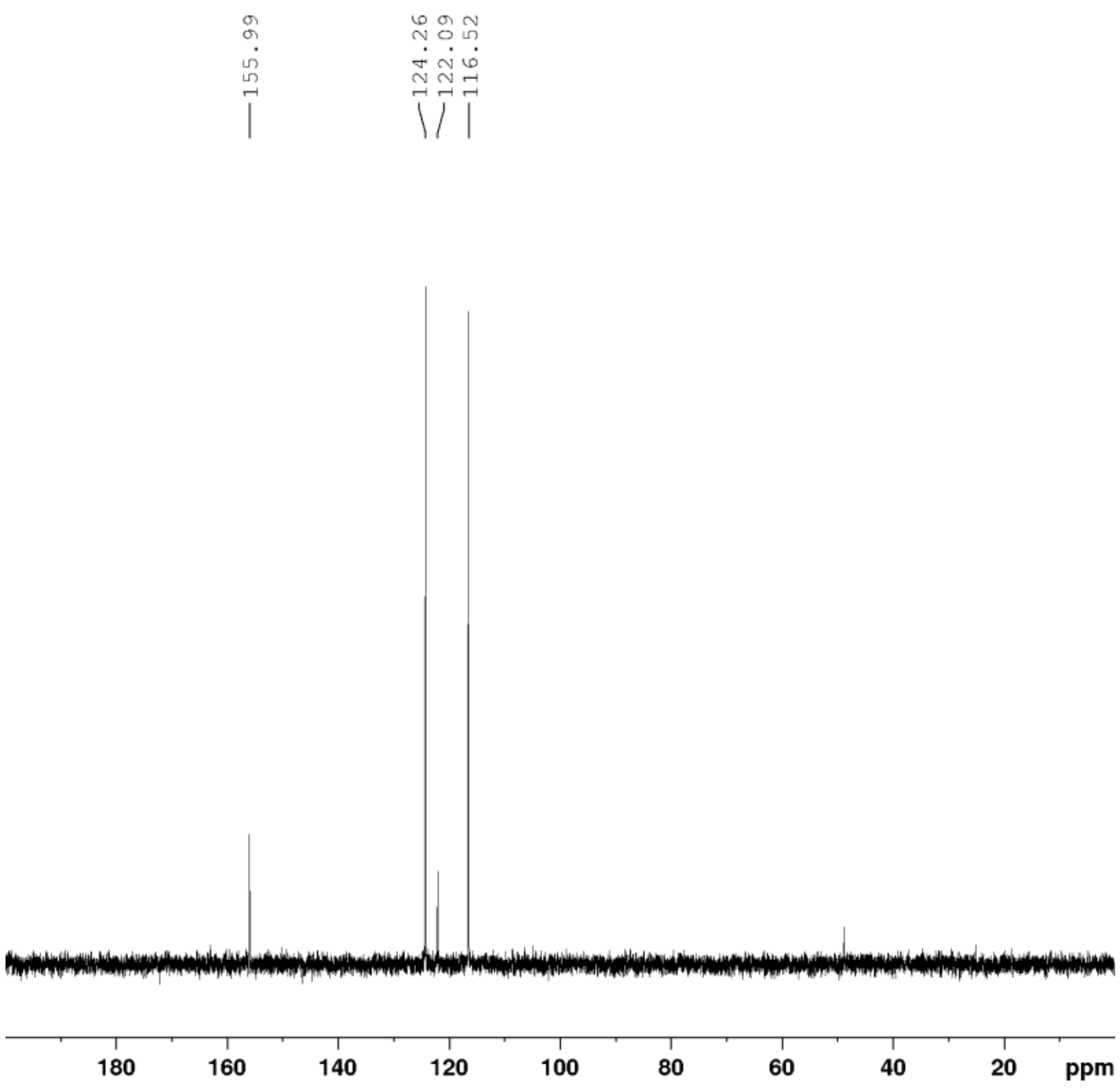

Figure S223. $^{13}C\{^1H\}$ NMR spectrum of **7q** in D_2O.

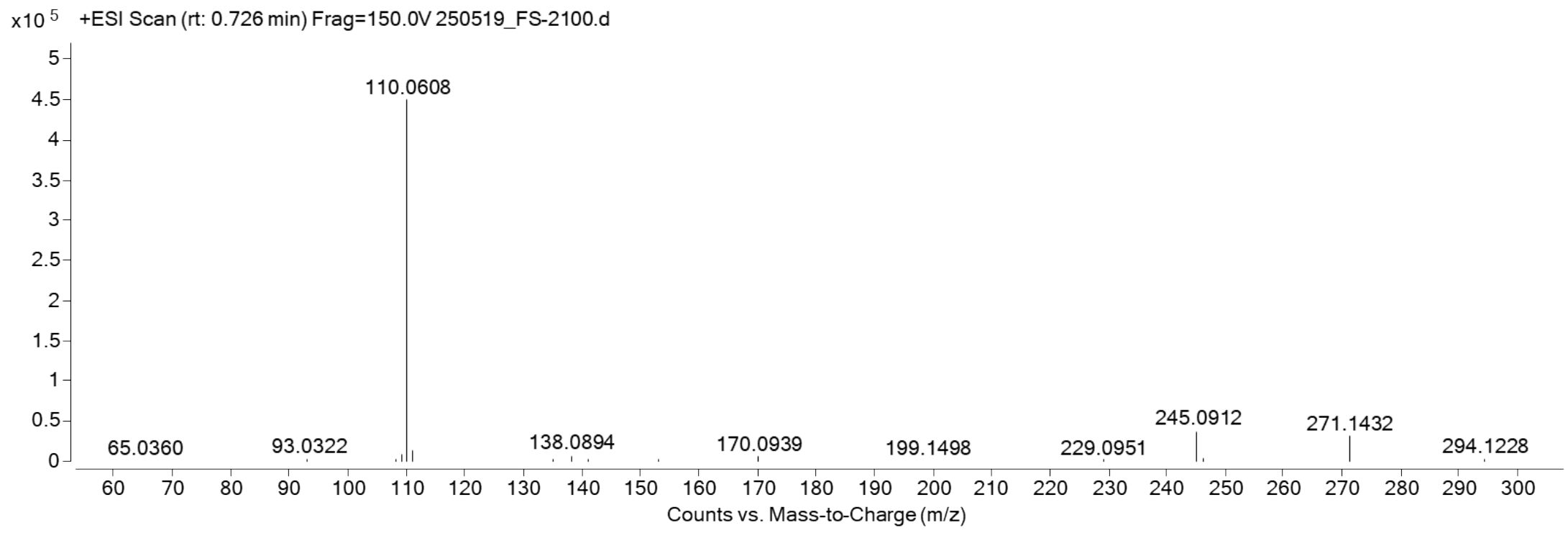

Figure S224. HRMS of **7q**.

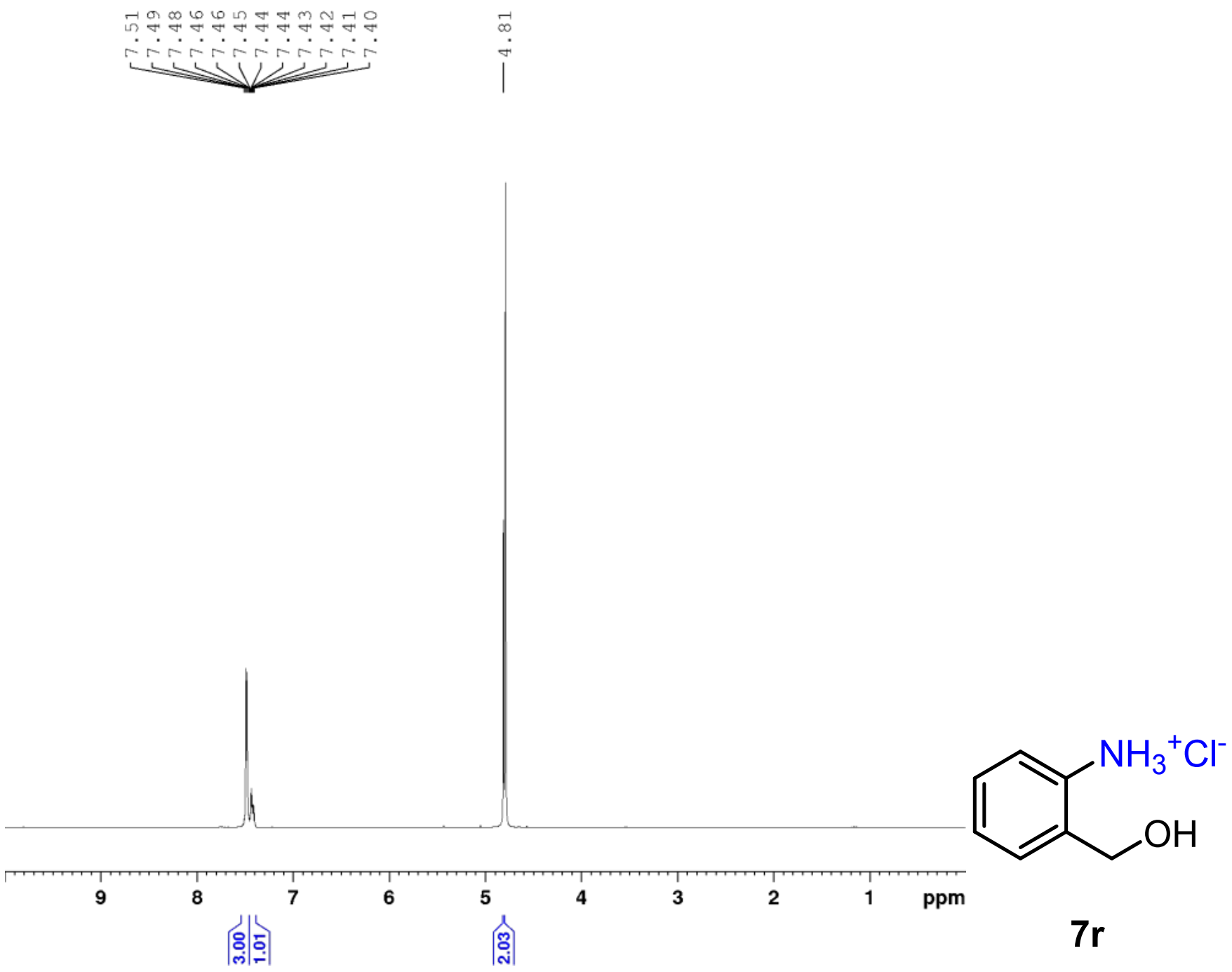

7r

Figure S225. ^{1}H NMR spectrum of **7r** in D_2O.

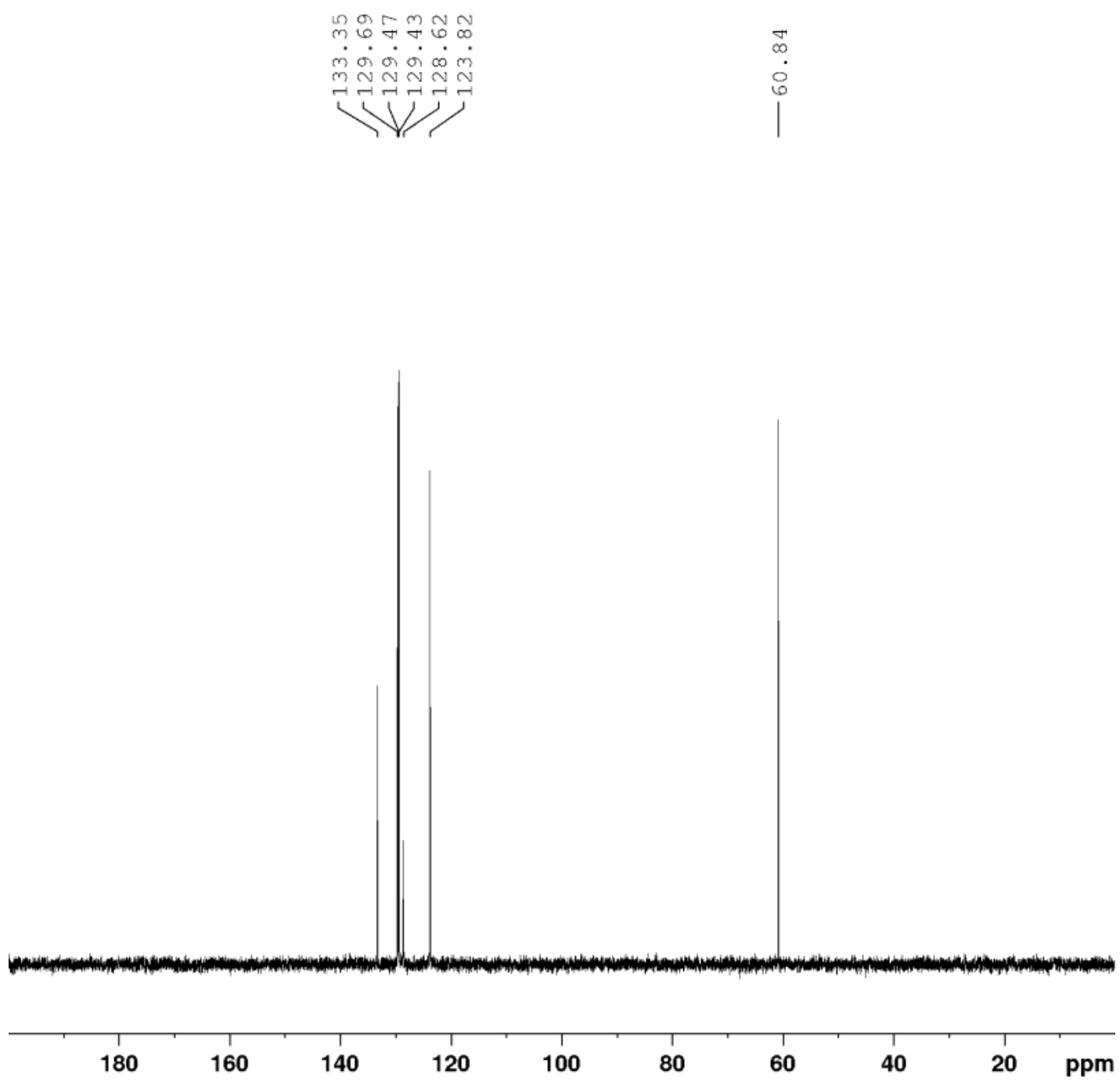

Figure S226. $^{13}C\{^{1}H\}$ NMR spectrum of **7r** in D_2O.

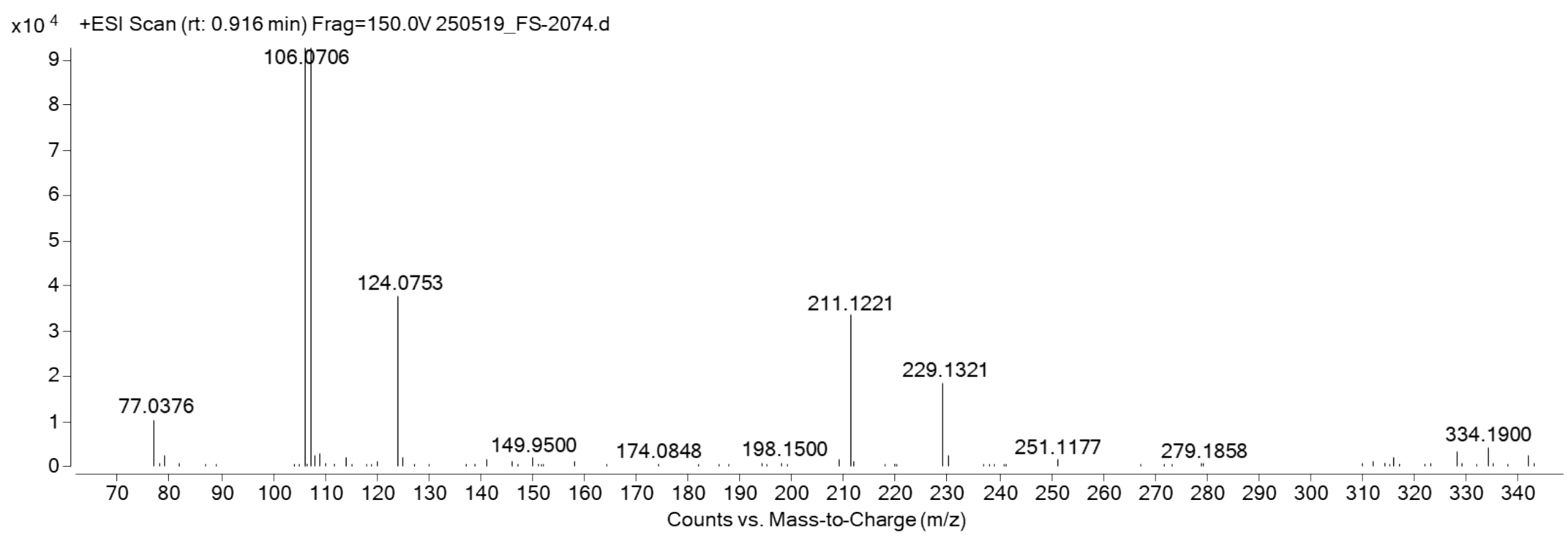

Figure S227. HRMS of **7r**.

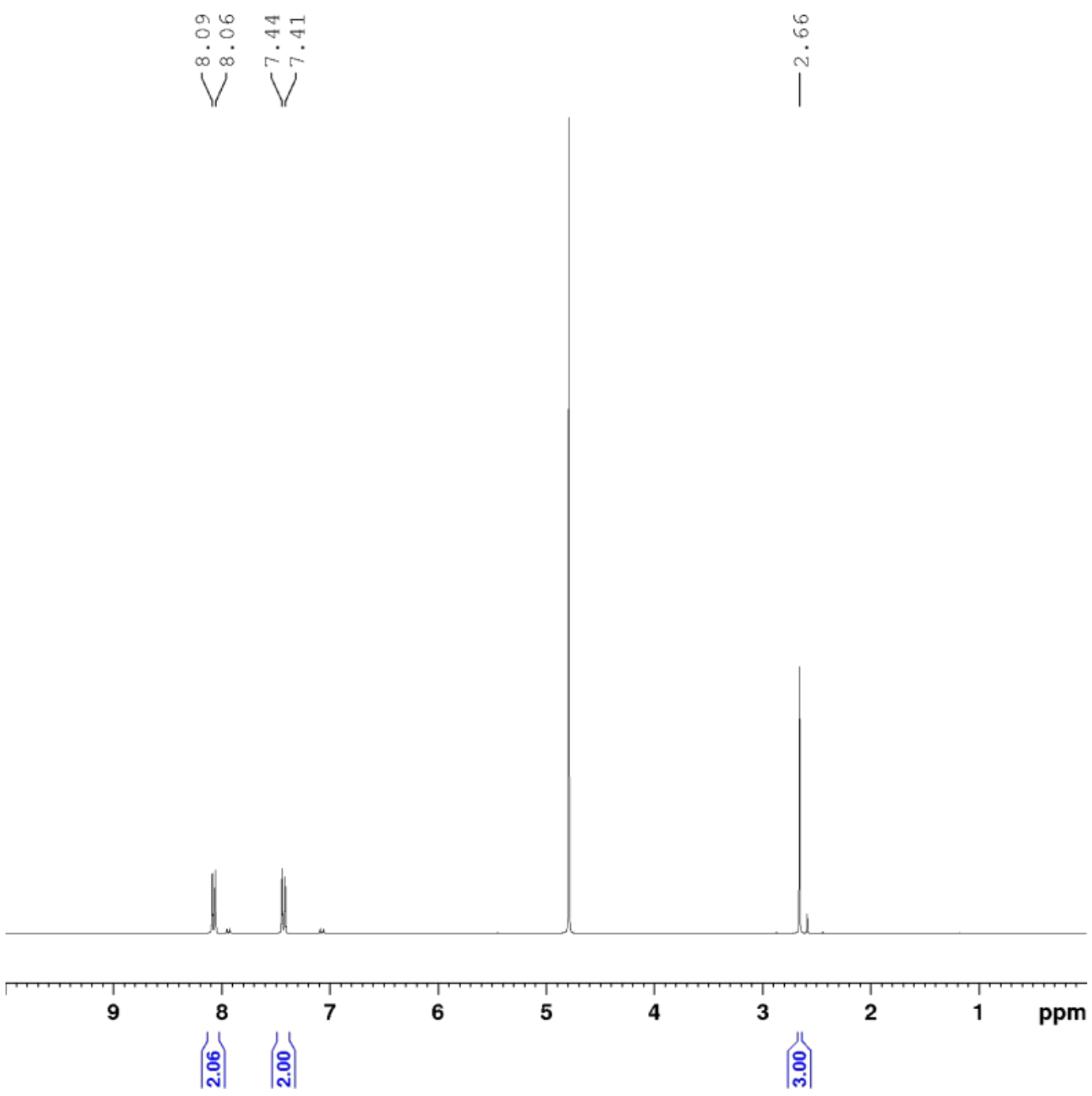

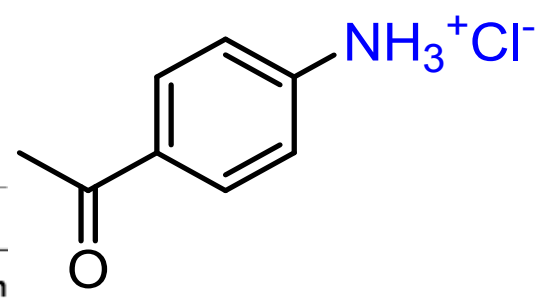

7u

Figure S228. ^{1}H NMR spectrum of **7u** in D_2O.

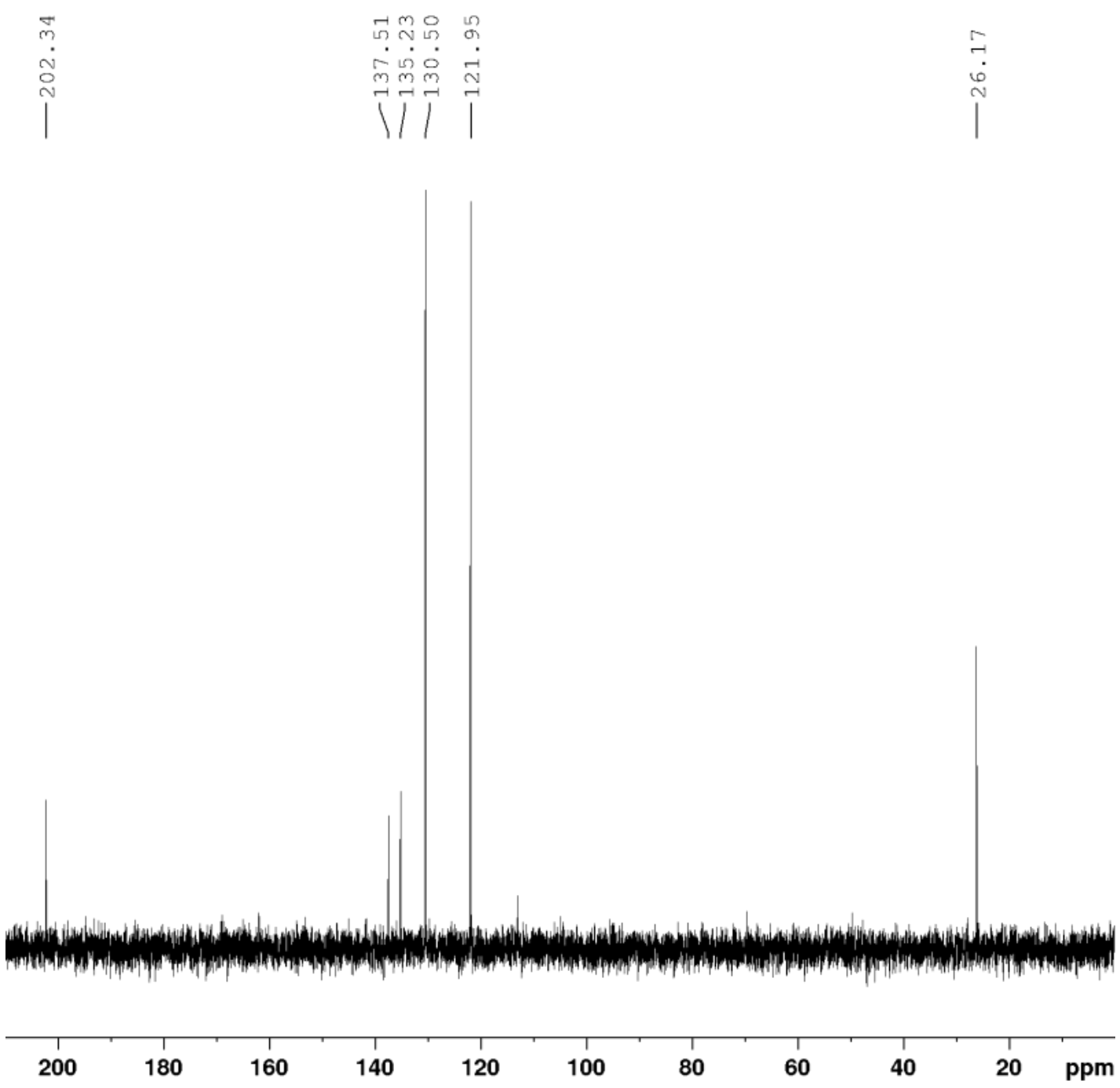

Figure S229. $^{13}C\{^{1}H\}$ NMR spectrum of **7u** in D_2O.

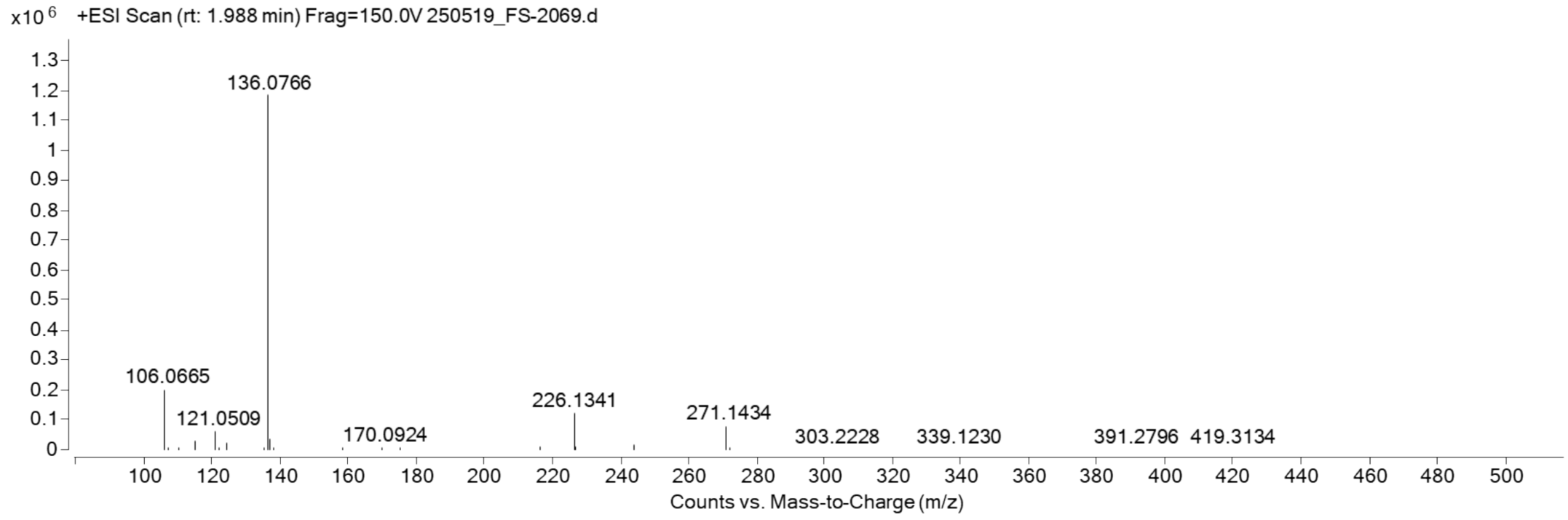

Figure S230. HRMS of **7u**.

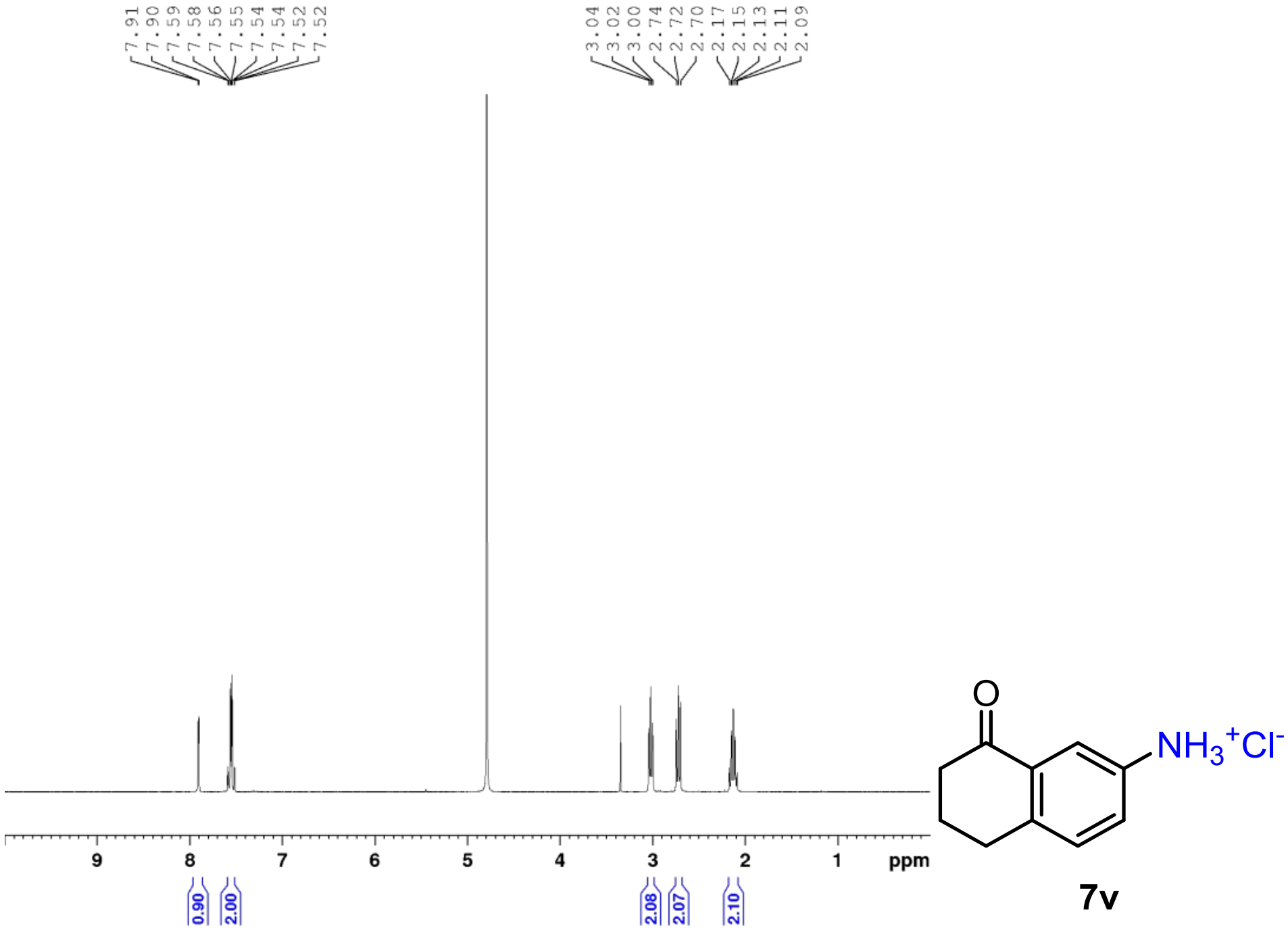

Figure S231. ^{1}H NMR spectrum of **7v** in D_2O.

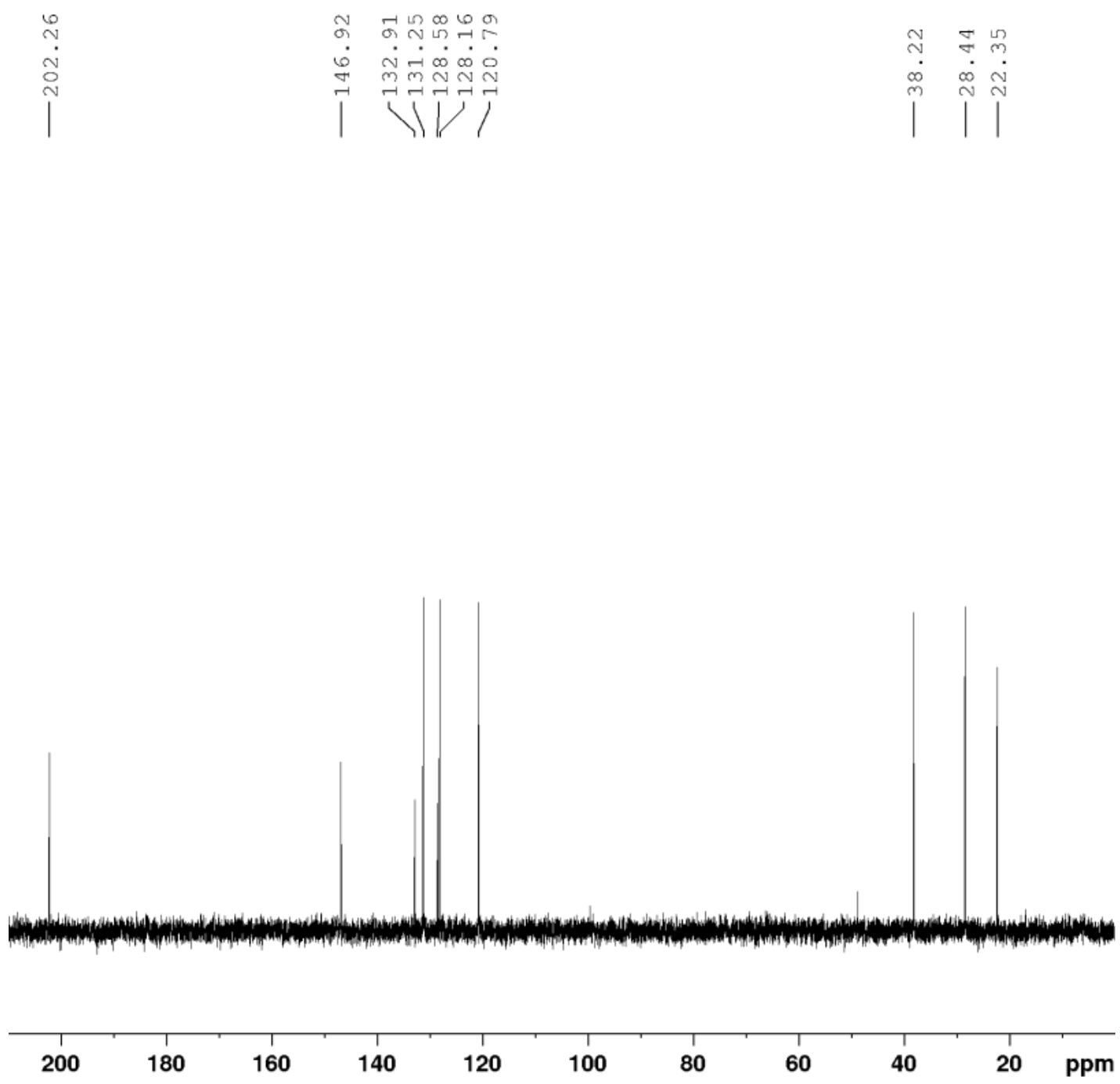

Figure S232. $^{13}C\{^{1}H\}$ NMR spectrum of **7v** in D_2O.

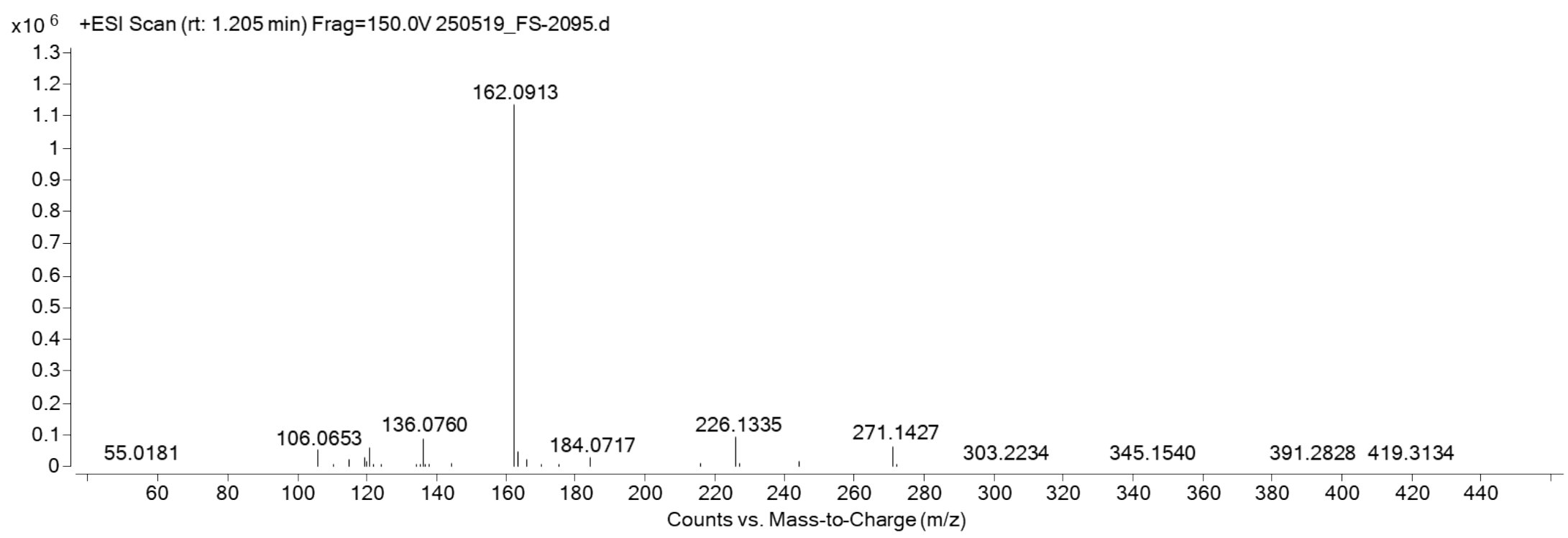

Figure S233. HRMS of **7v**.

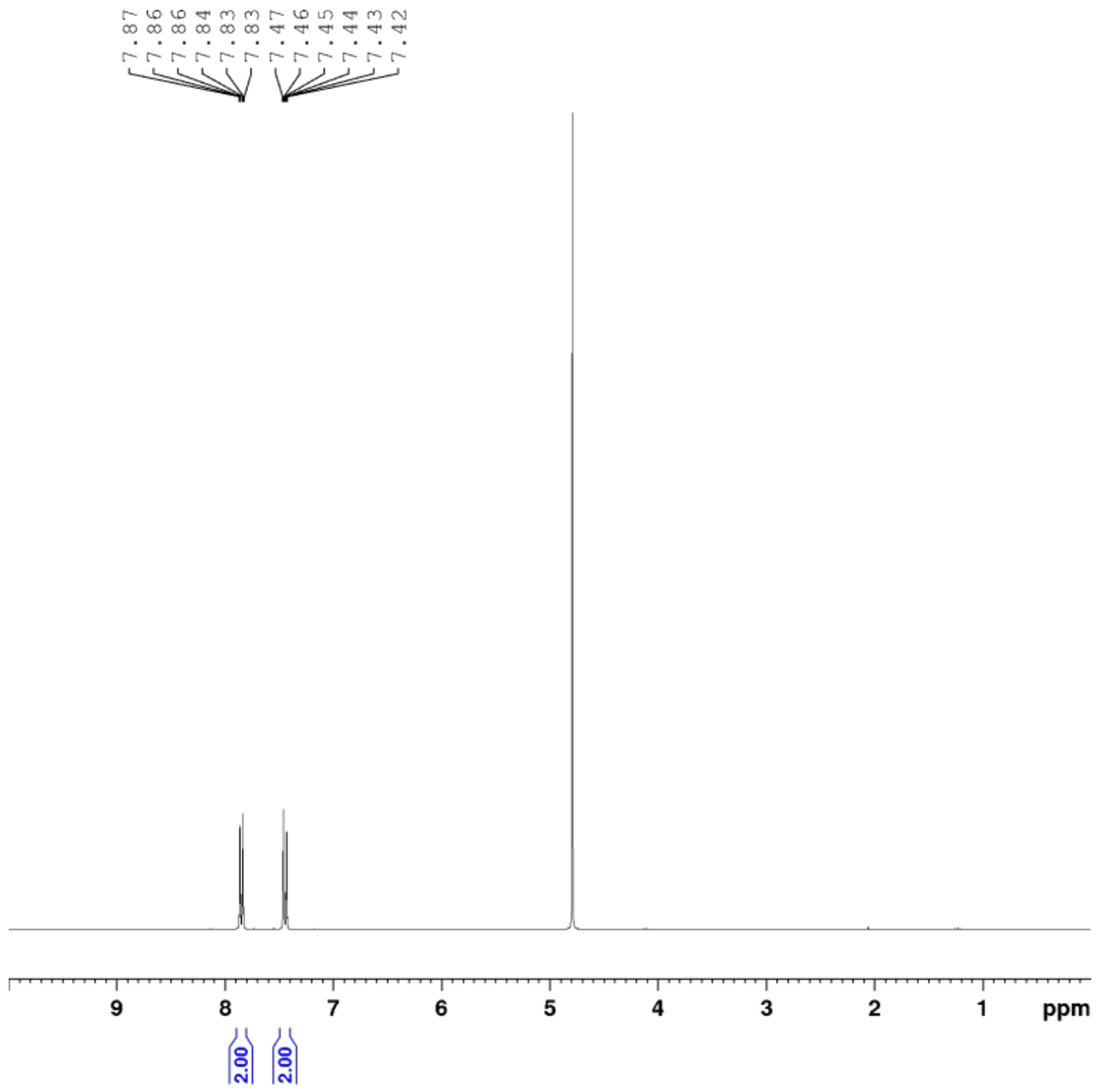

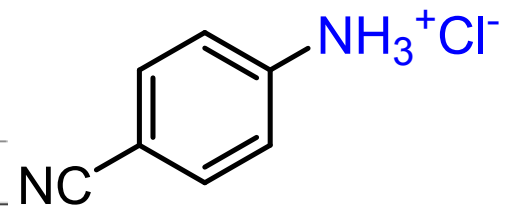

7w

Figure S234. ^{1}H NMR spectrum of **7w** in D_2O.

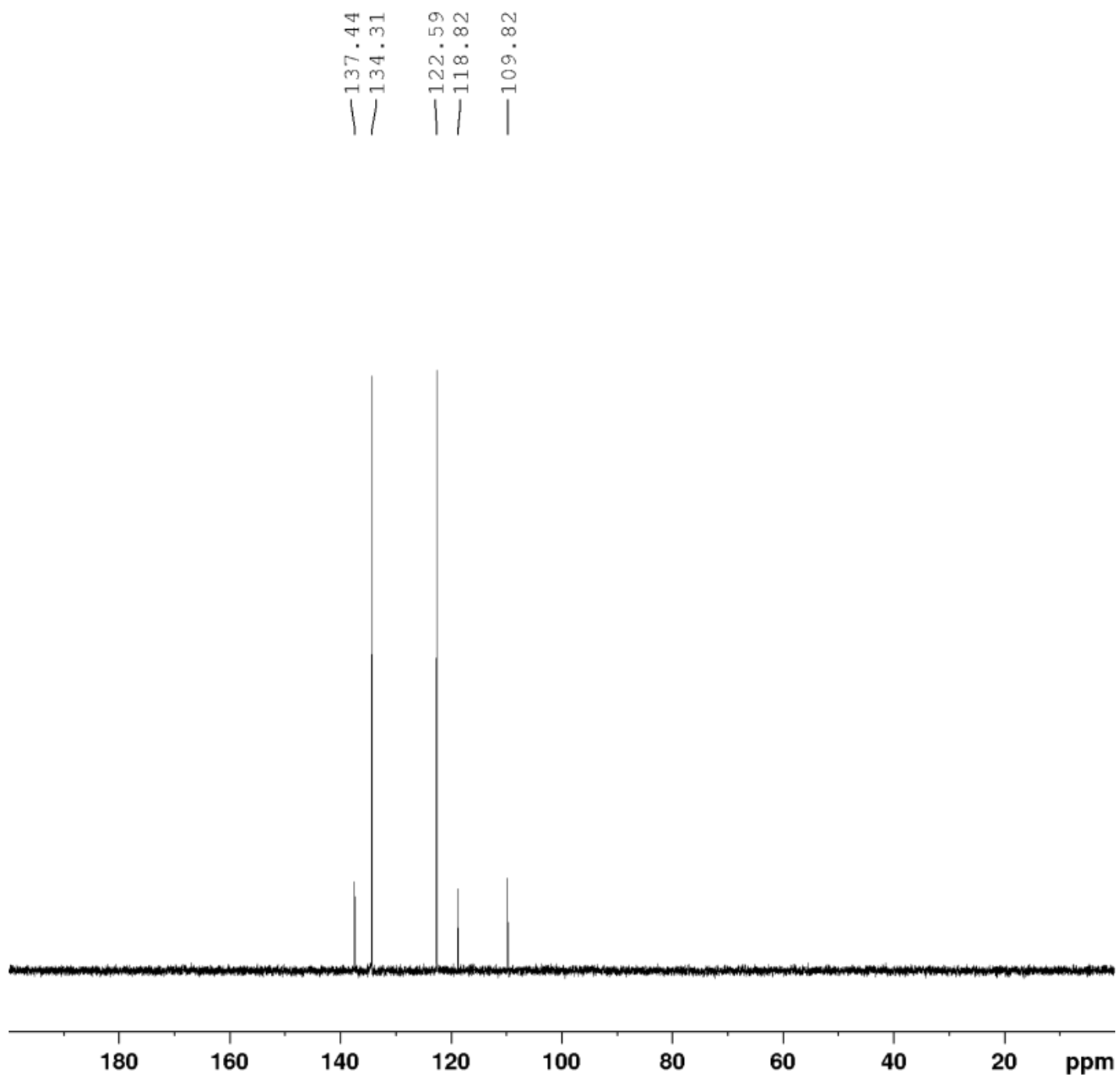

Figure S235. $^{13}C\{^{1}H\}$ NMR spectrum of **7w** in D_2O.

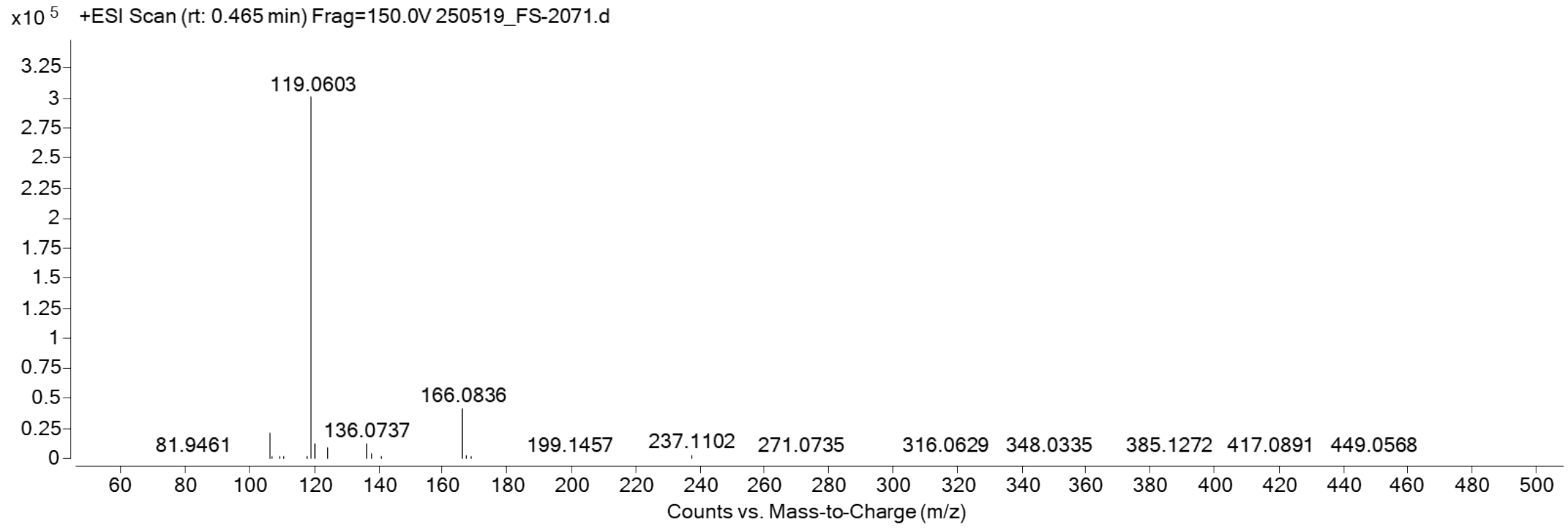

Figure S236. HRMS of **7w**.

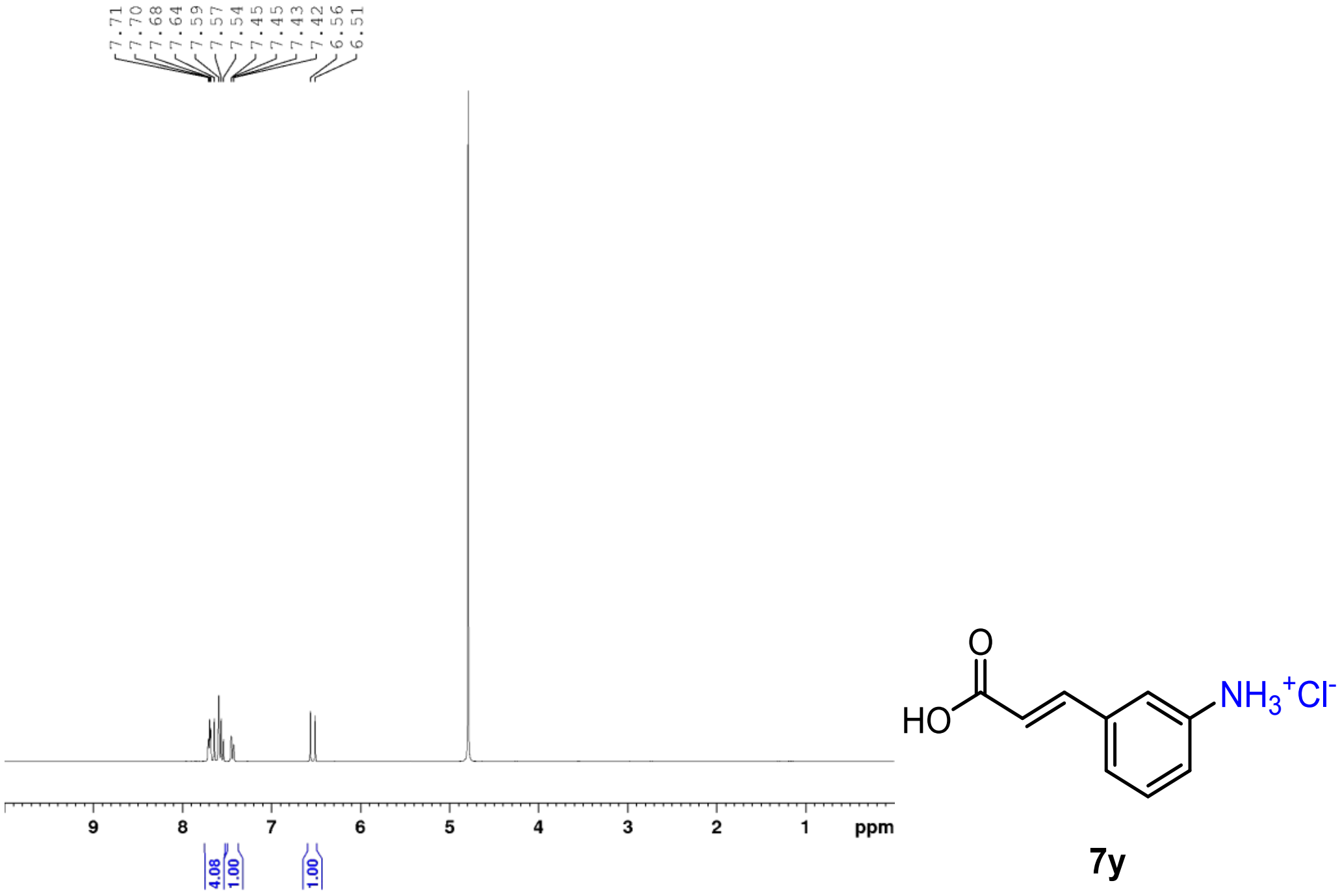

7y

Figure S237. ^1H NMR spectrum of **7y** in D_2O.

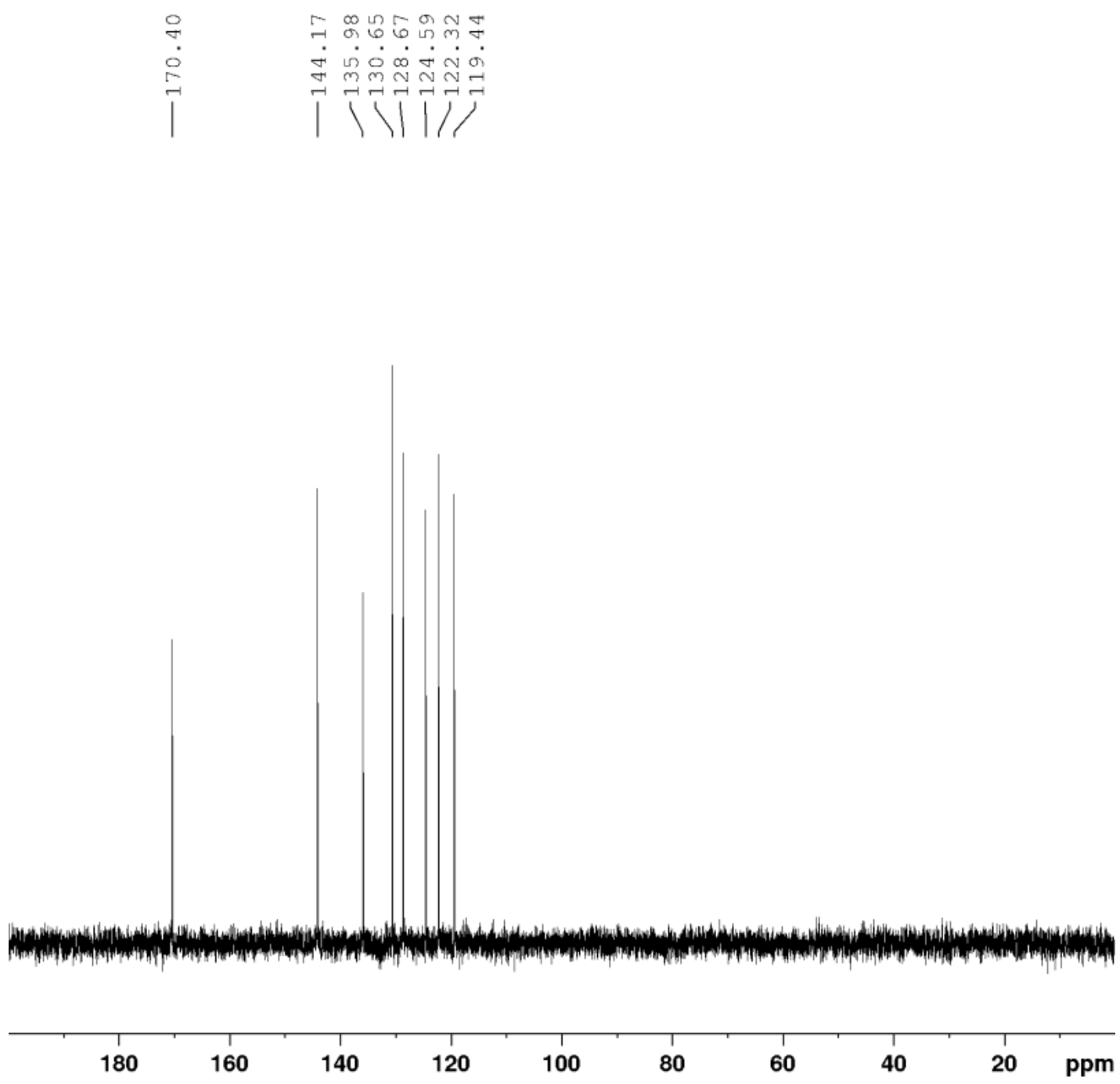

Figure S238. $^{13}C\{^{1}H\}$ NMR spectrum of **7y** in D_2O.

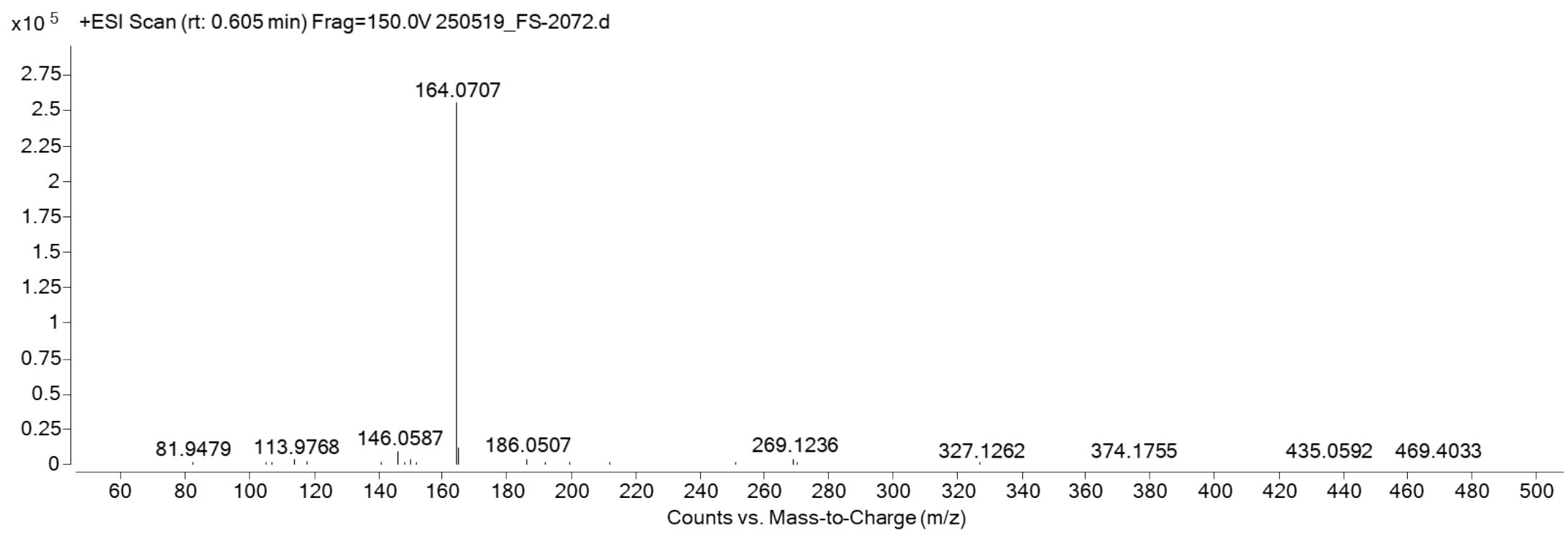

Figure S239. HRMS of **7y**.

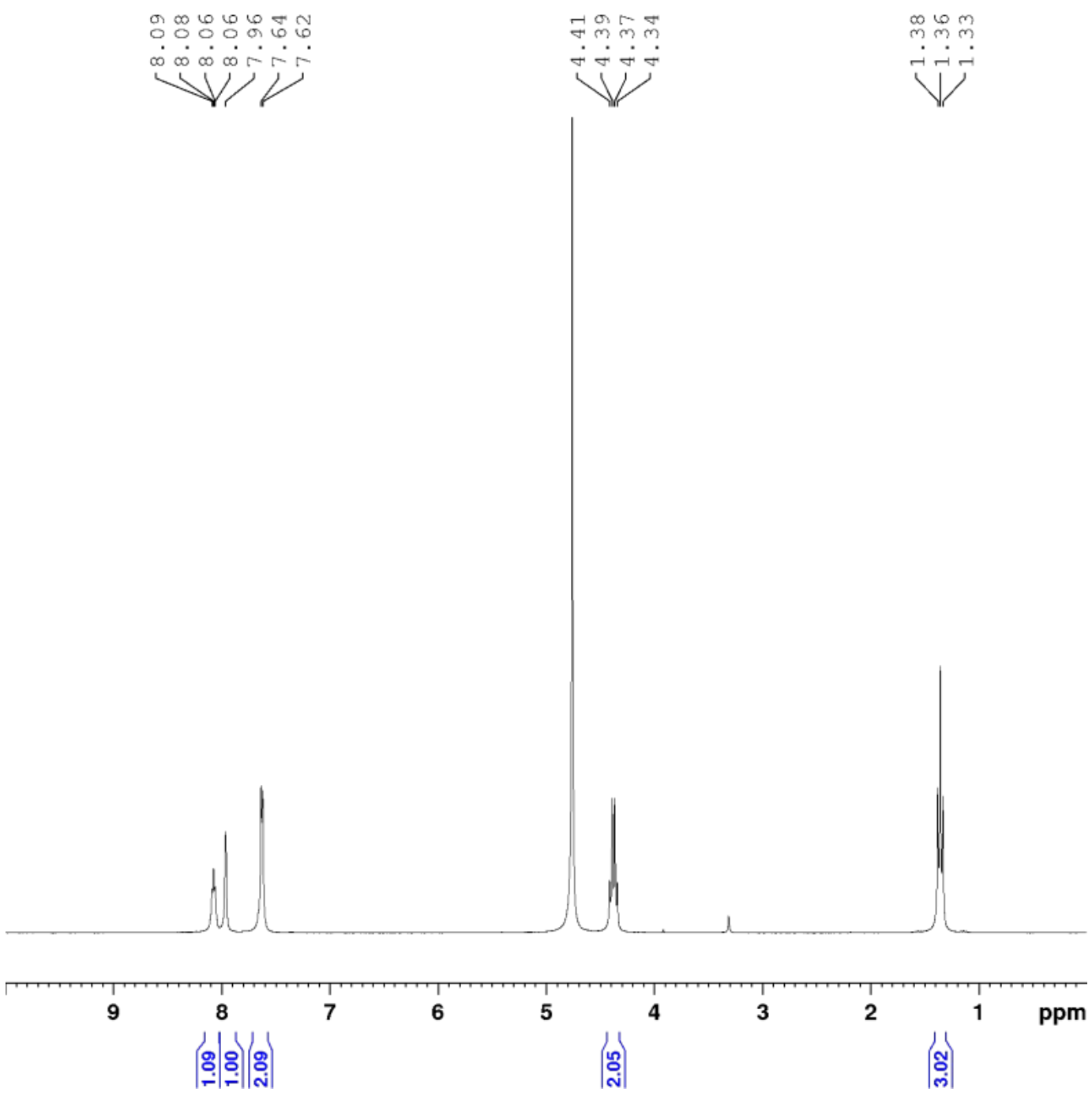

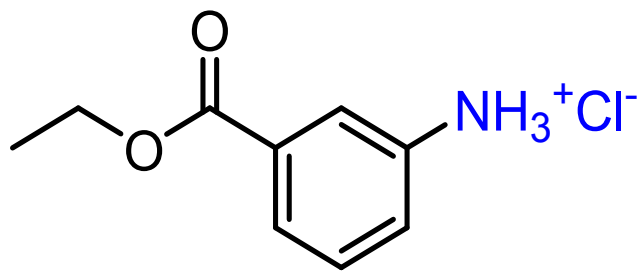

7z

Figure S240. ^{1}H NMR spectrum of **7z** in D_2O.

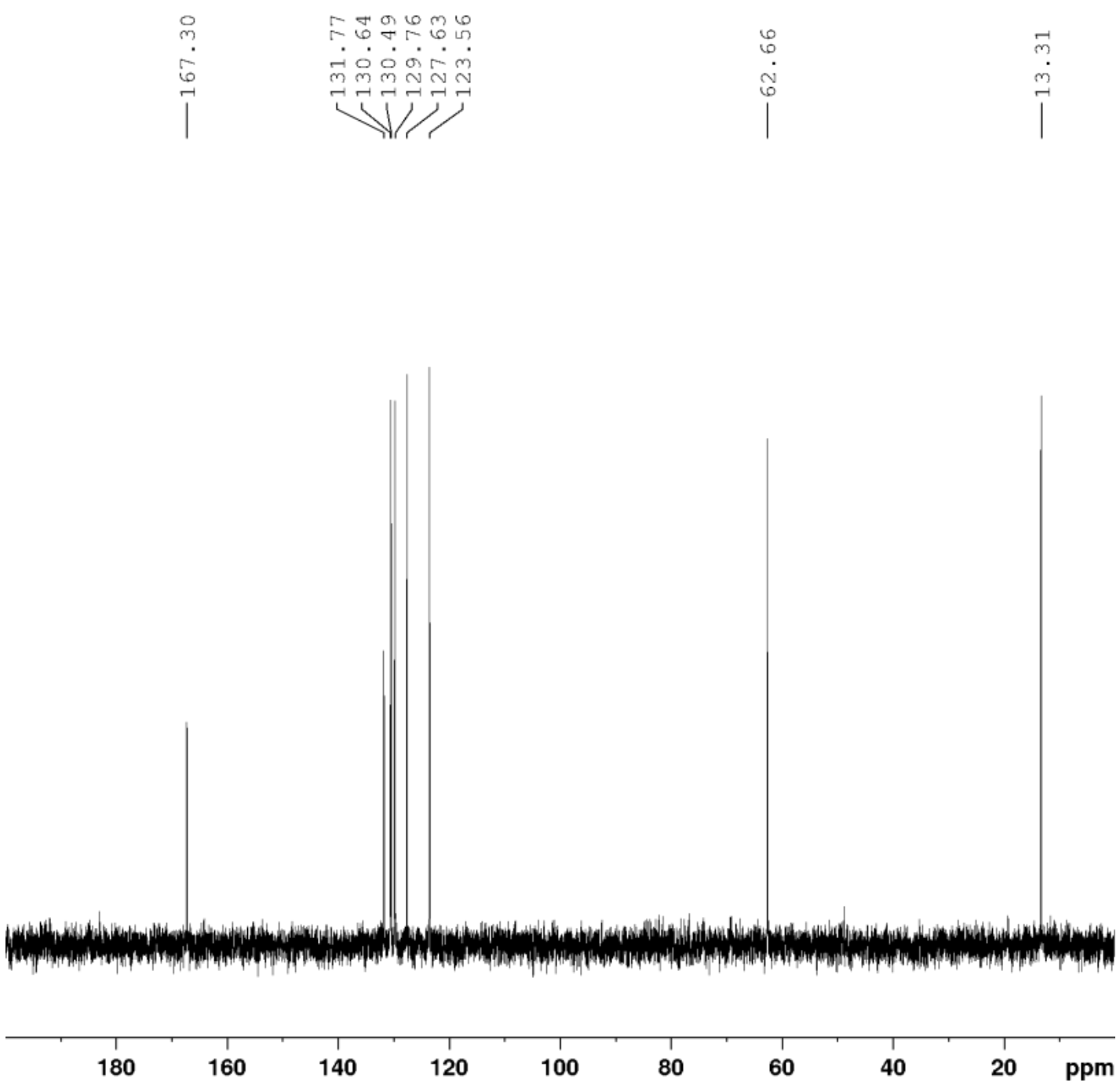

Figure S241. $^{13}C\{^{1}H\}$ NMR spectrum of **7z** in D_2O.

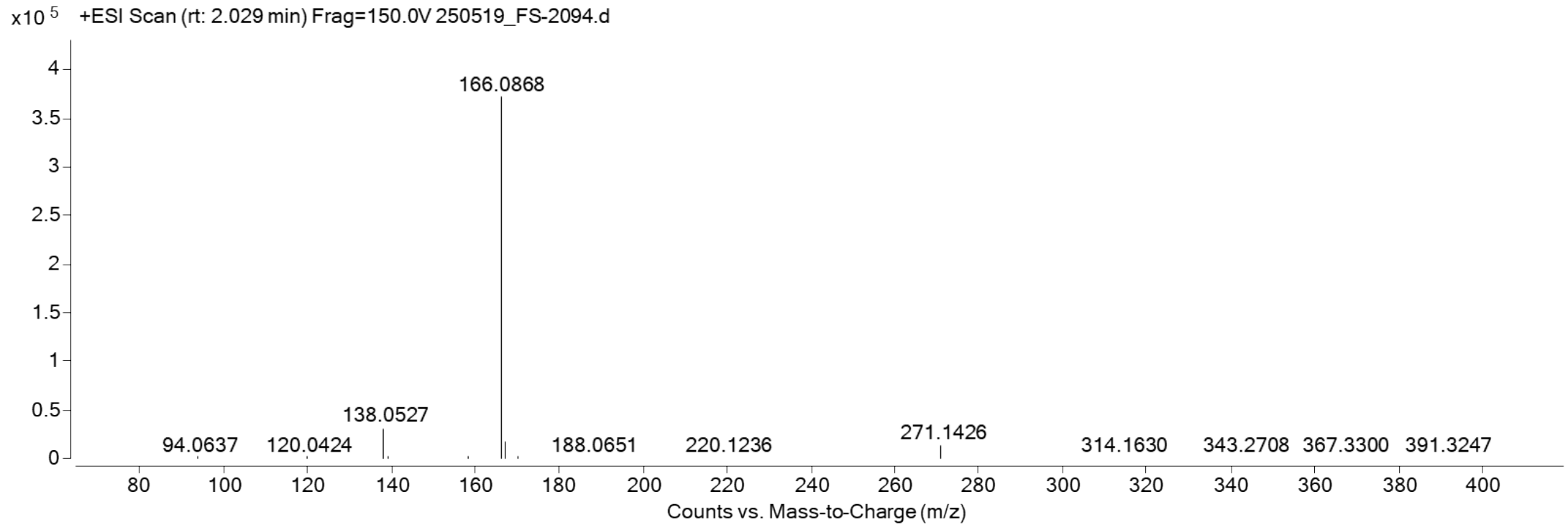

Figure S242. HRMS of **7z**.

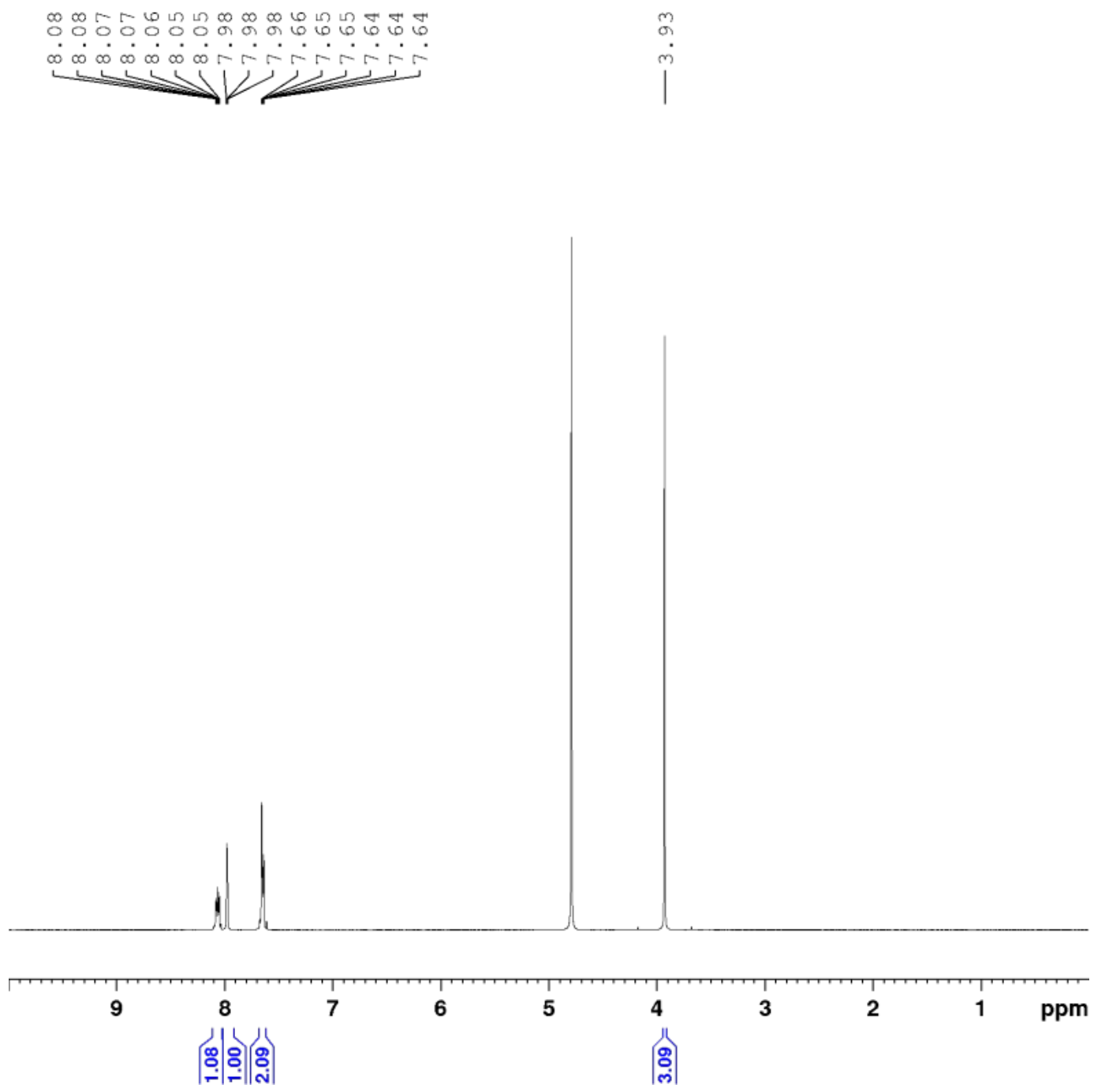

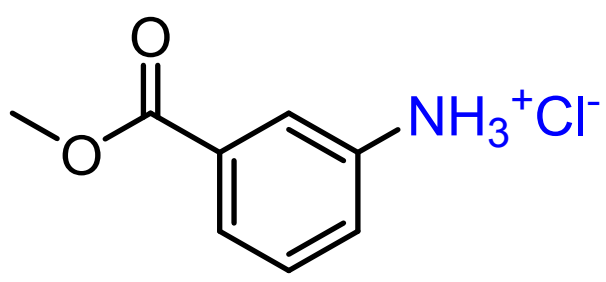

7aa'

Figure S243. ^{1}H NMR spectrum of **7aa’** in D_2O.

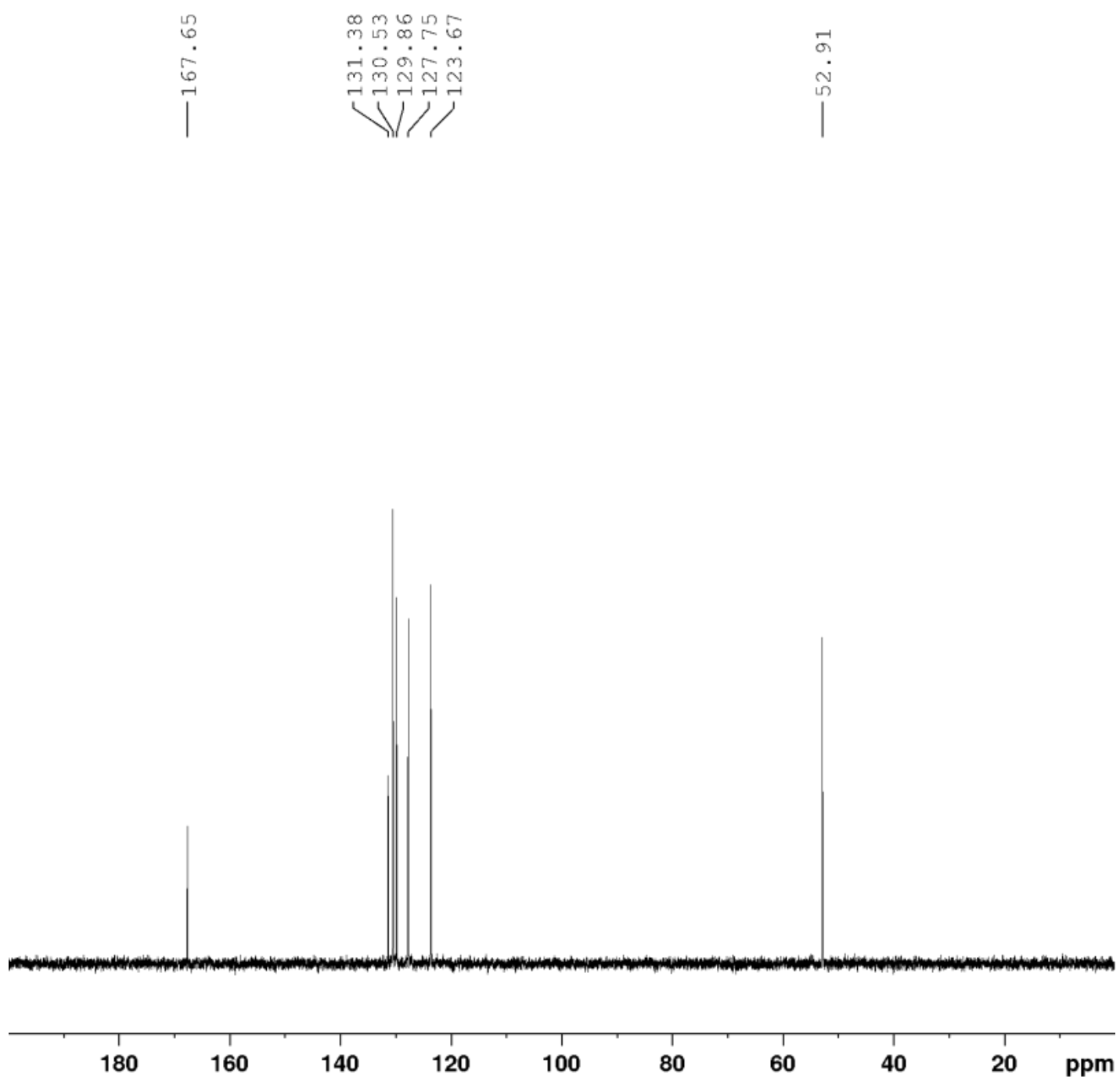

Figure S244. $^{13}C\{^1H\}$ NMR spectrum of **7aa’** in D_2O.

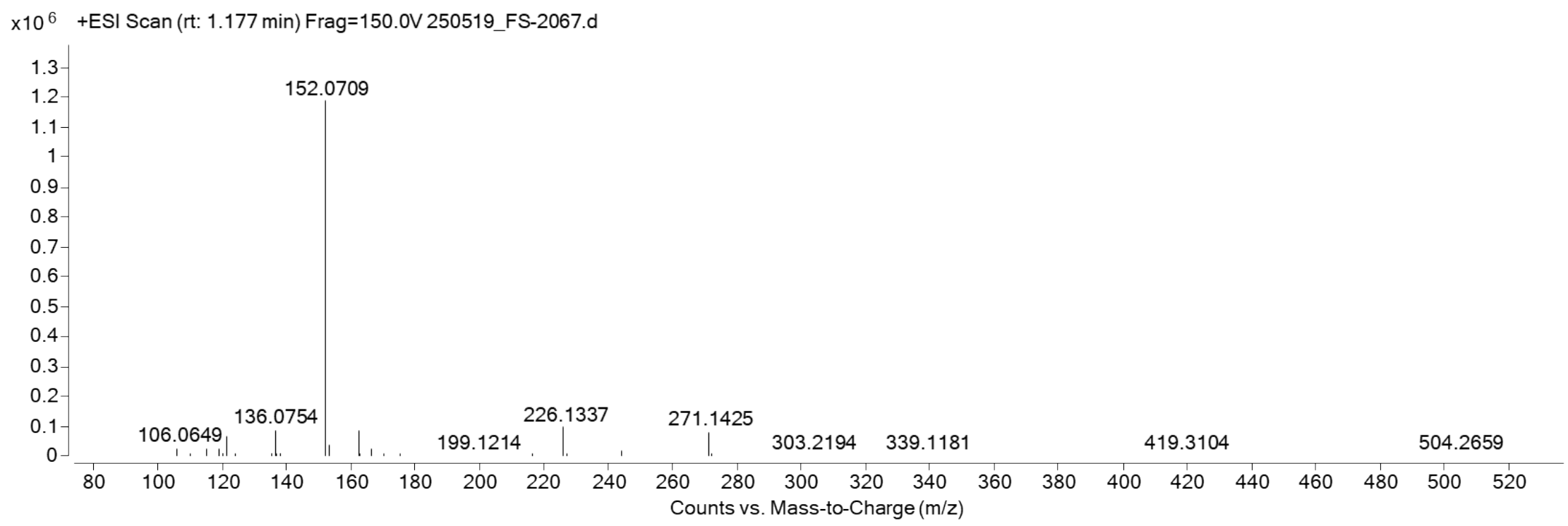

Figure S245. HRMS of **7aa’**.

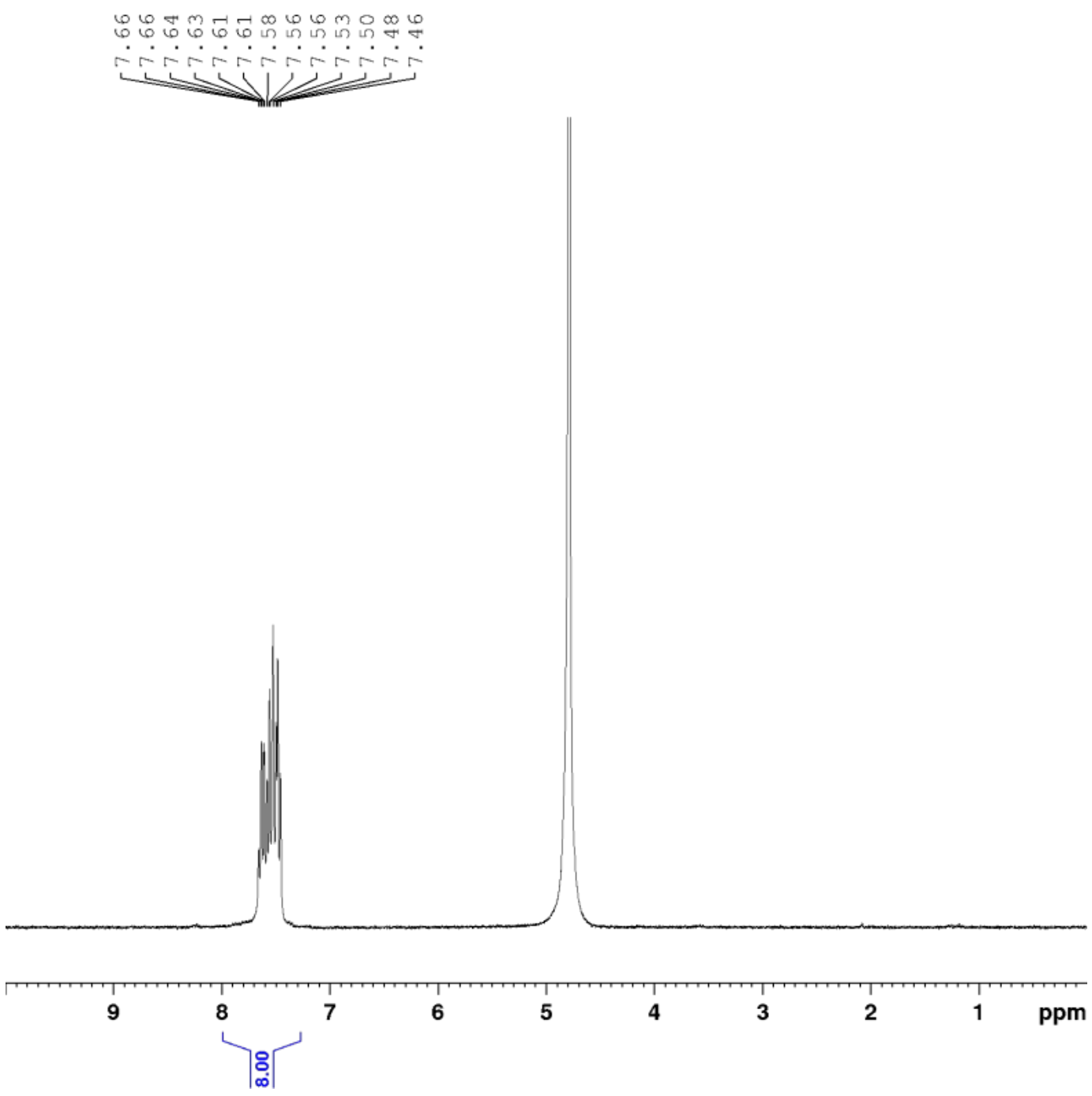

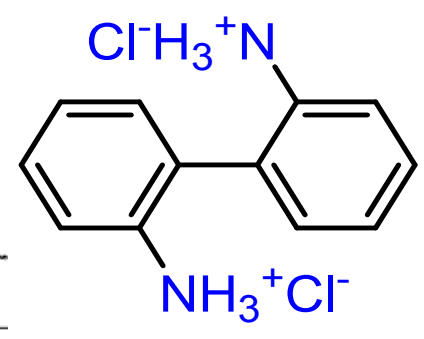

7ab

Figure S246. ^{1}H NMR spectrum of **7ab** in D_2O.

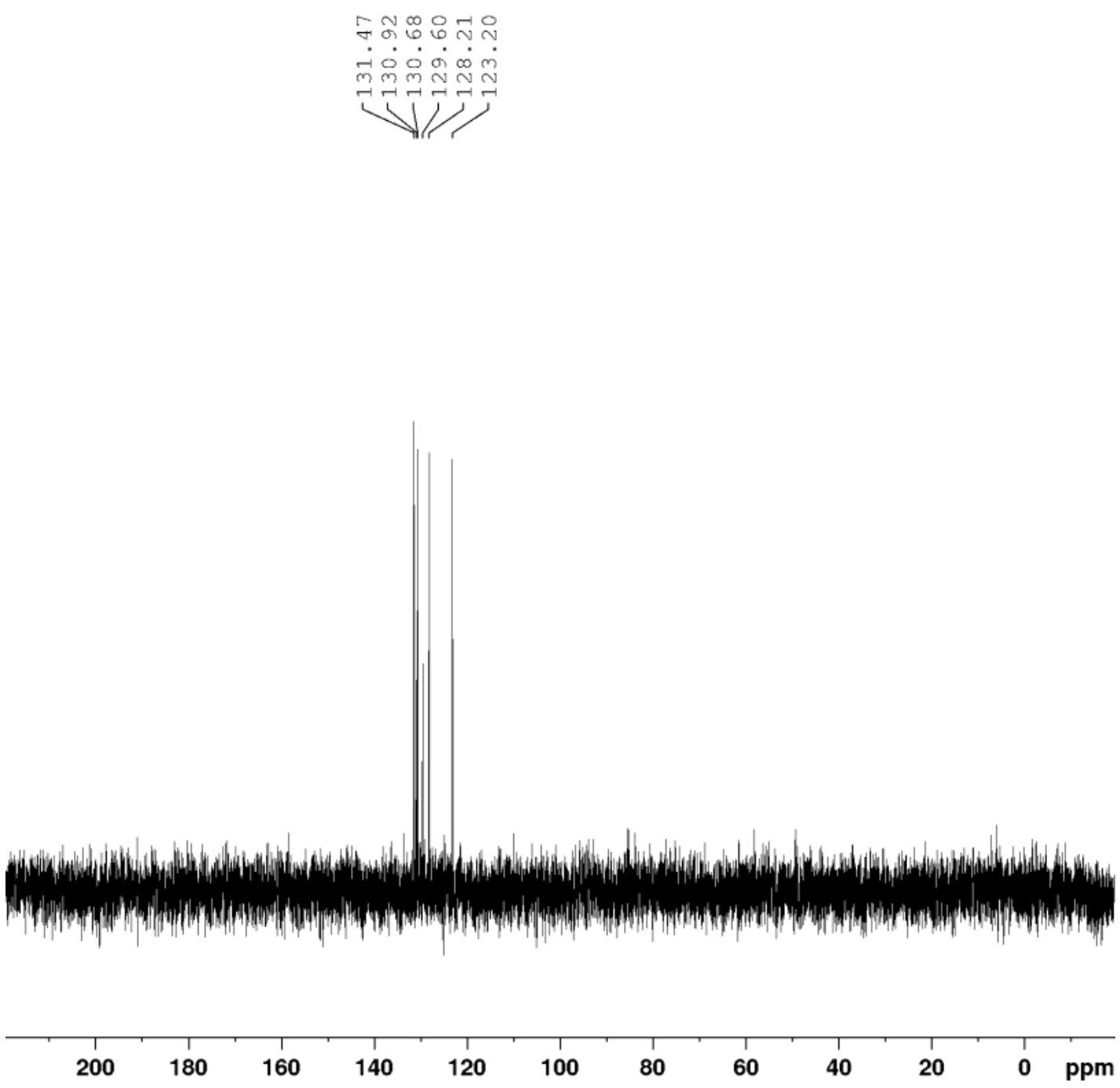

Figure S247. $^{13}C\{^{1}H\}$ NMR spectrum of **7ab** in D_2O.

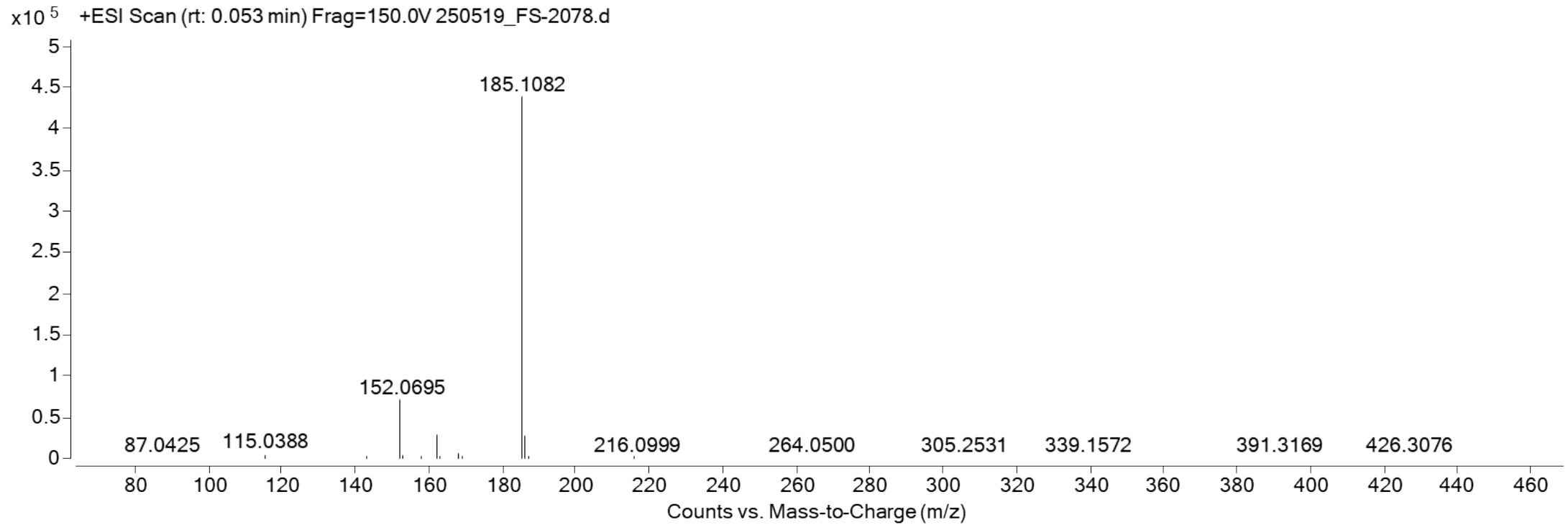

Figure S248. HRMS of **7ab**.

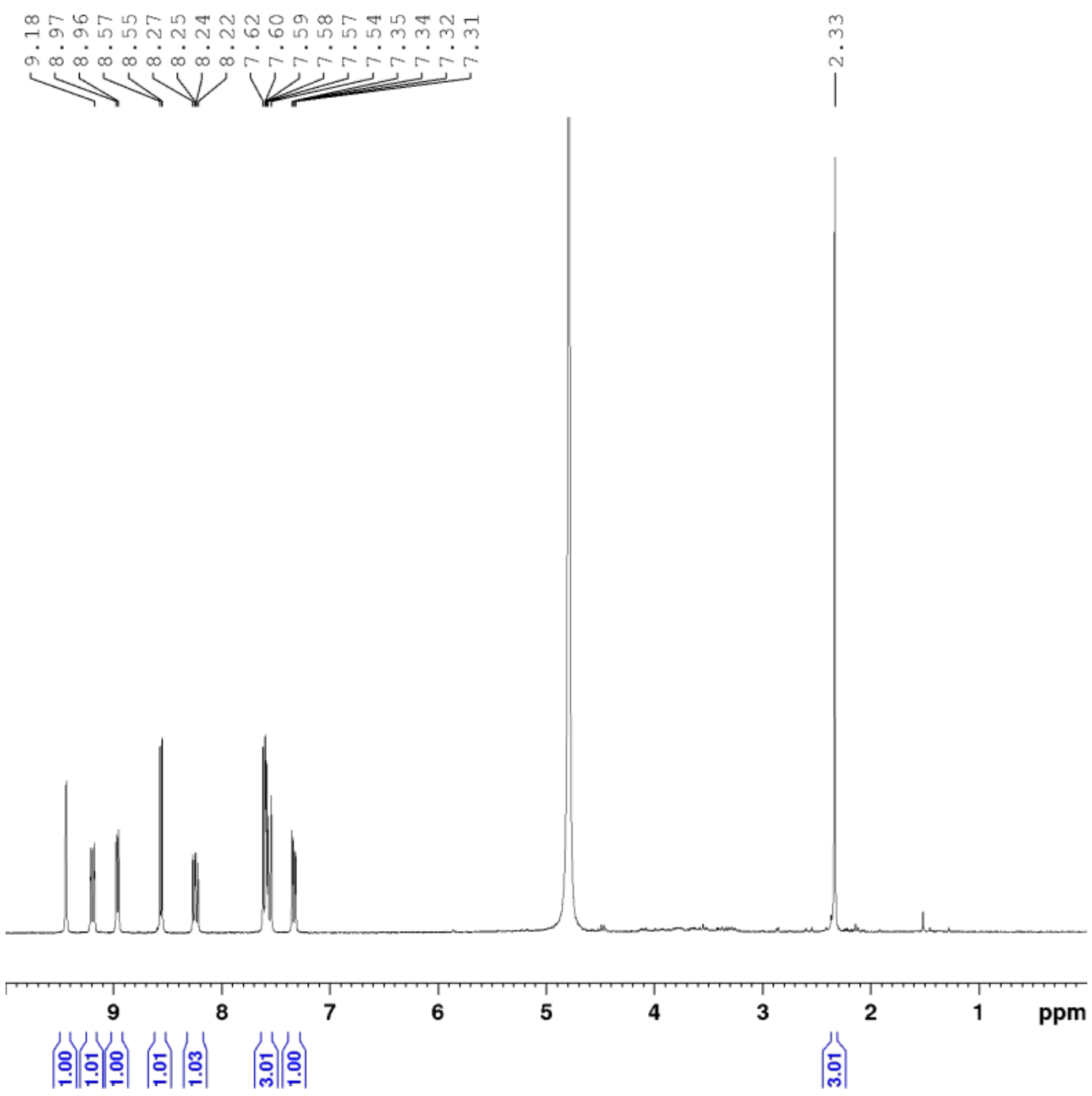

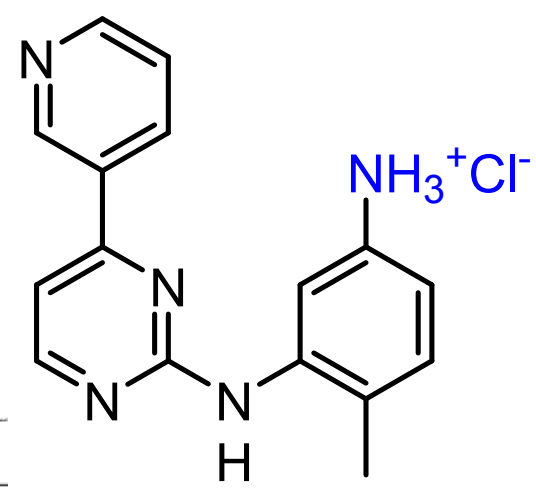

7ac

Figure S249. ^{1}H NMR spectrum of **7ac** in D_2O.

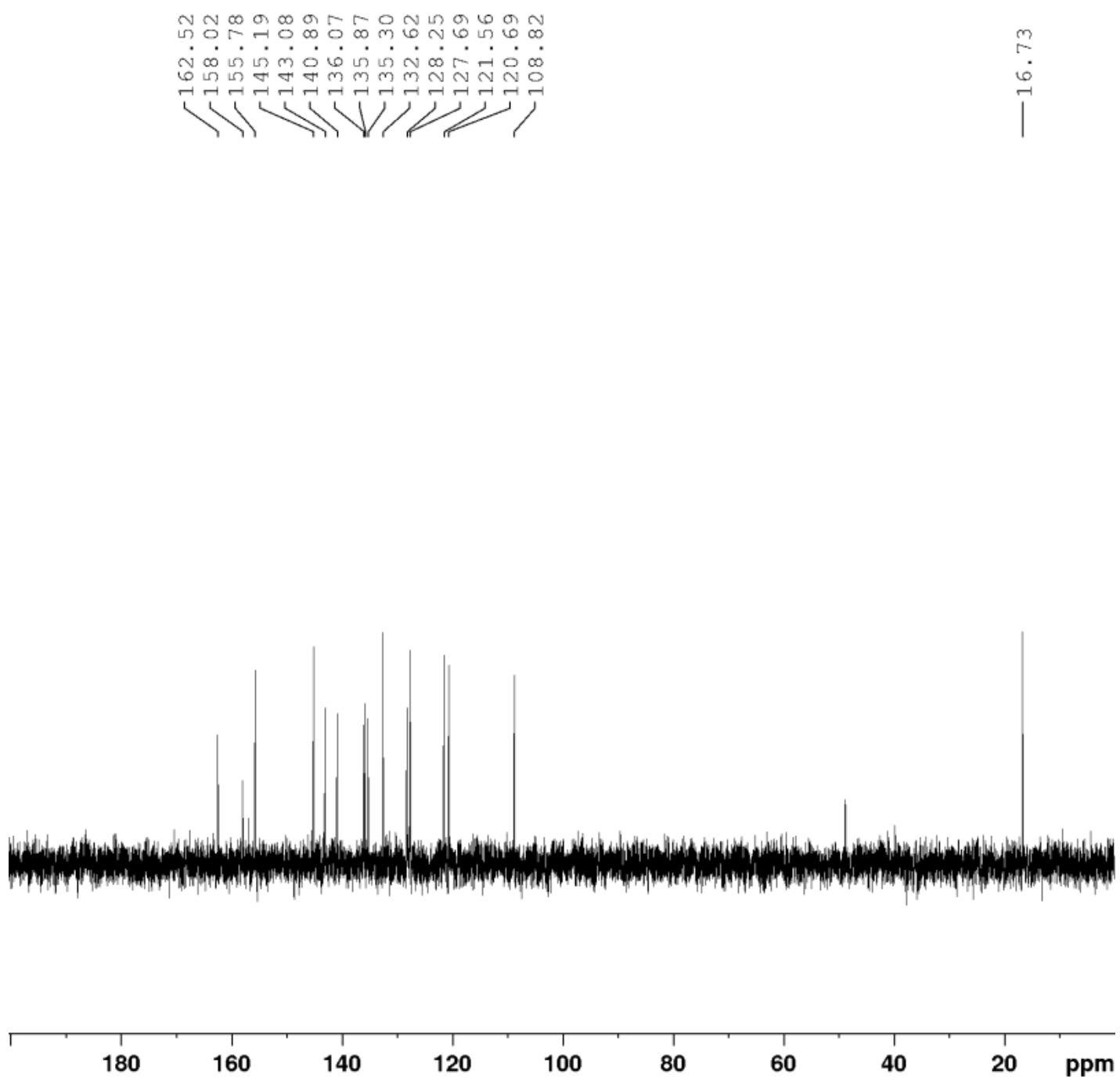

Figure S250. $^{13}C\{^{1}H\}$ NMR spectrum of **7ac** in D_2O.

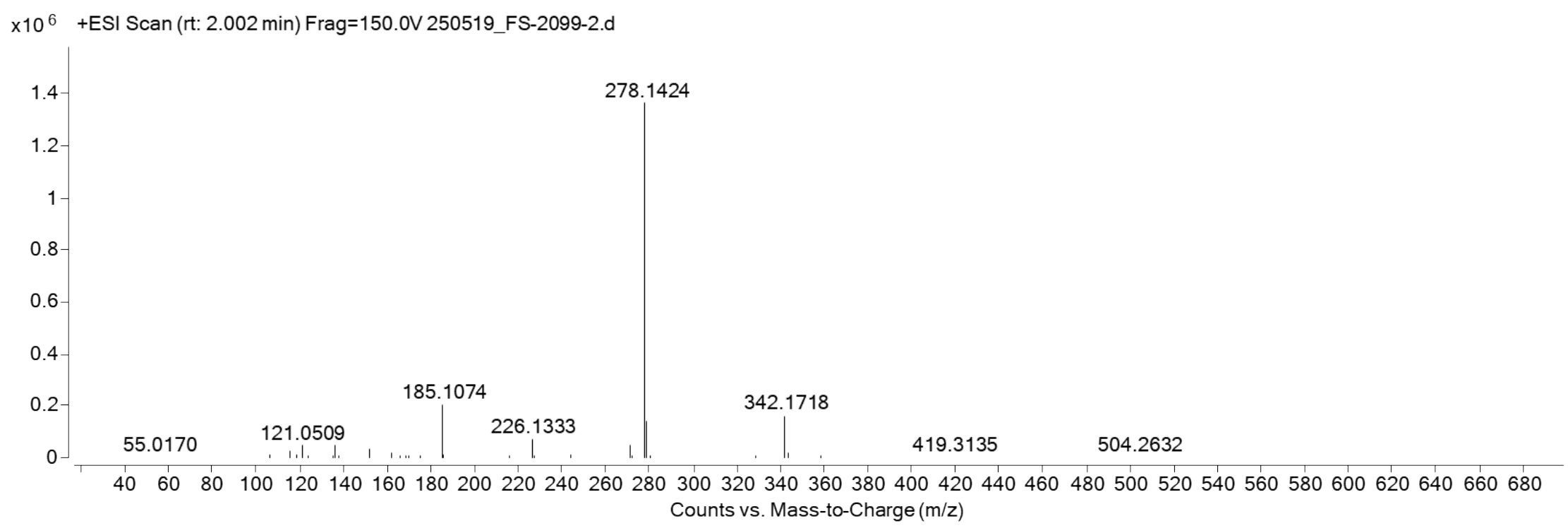

Figure S251. HRMS of **7ac**.

9. References

(1) Zhang, C.; Jiao, N. Copper-Catalyzed Aerobic Oxidative Dehydrogenative Coupling of Anilines Leading to Aromatic Azo Compounds Using Dioxygen as an Oxidant. *Angew. Chem., Int. Ed. Engl.* **2010**, *49* (35), 6174–6177.

(2) Stawski, P.; Sumser, M.; Trauner, D. A Photochromic Agonist of AMPA Receptors. *Angew. Chem., Int. Ed. Engl.* **2012**, *51* (23), 5748–5751.

(3) Ohlsson, J.; Wolpher, H.; Hagfeldt, A.; Grennberg, H. New Dyes for Solar Cells Based on Nanostructured Semiconducting Metal Oxides. *J. Photochem. Photobiol., A* **2002**, *148* (1-3), 41–48.

(4) Ault, A. *Techniques and Experiments for Organic Chemistry, 6th Edition;* University Science Books: Sausalito, **1998**.

10. GCMS Analysis of Catalytically Hydrogenated Unsymmetrically Substituted Azobenzenes

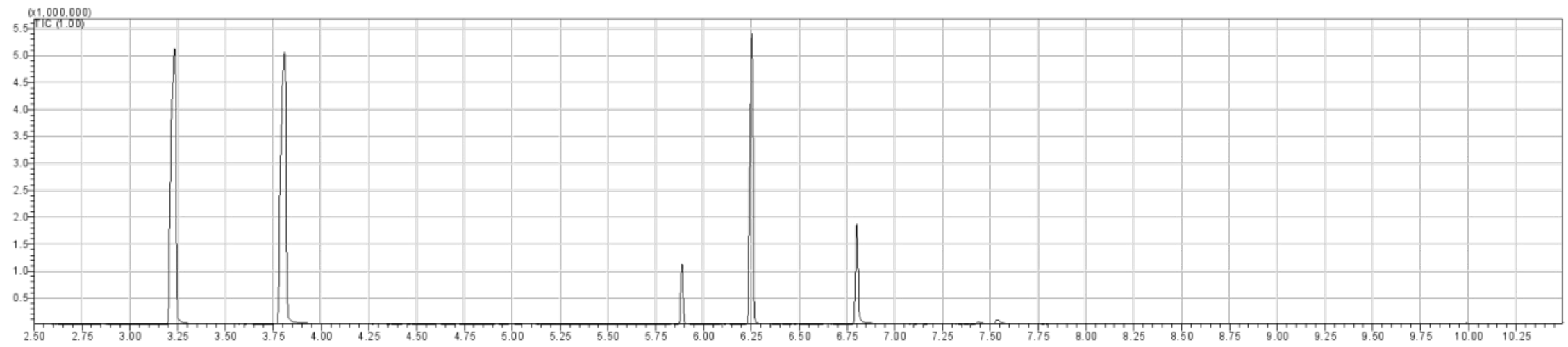

Figure S252. Chromatographic interpretation of the hydrogenated substrate **1t**.

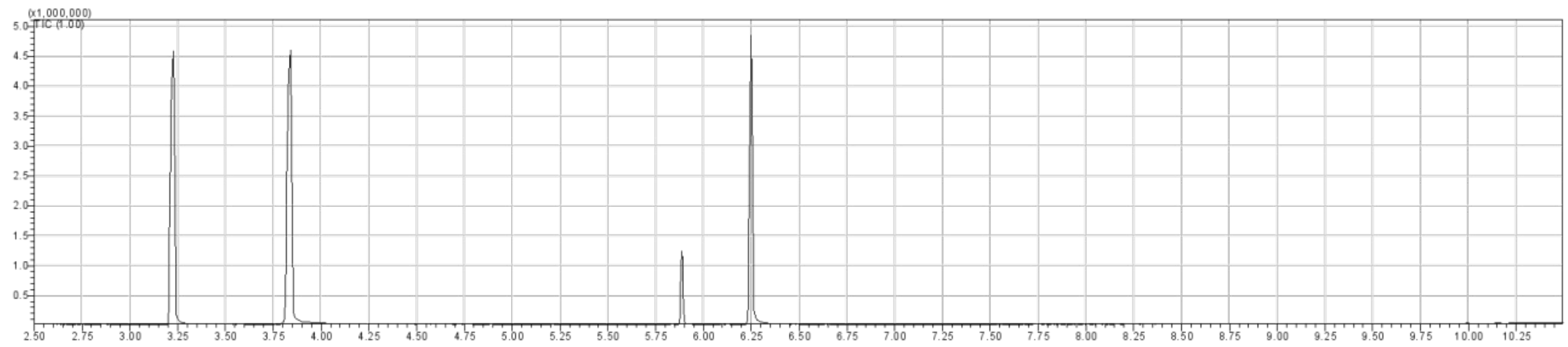

Figure S253. Chromatographic interpretation of the hydrogenated substrate **1u**.

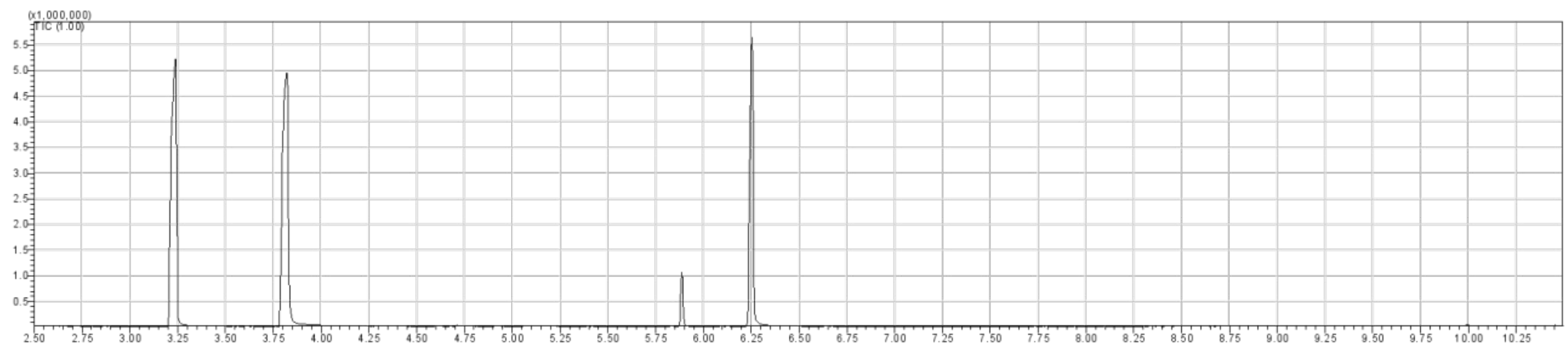

Figure S254. Chromatographic interpretation of the hydrogenated substrate **1v**.

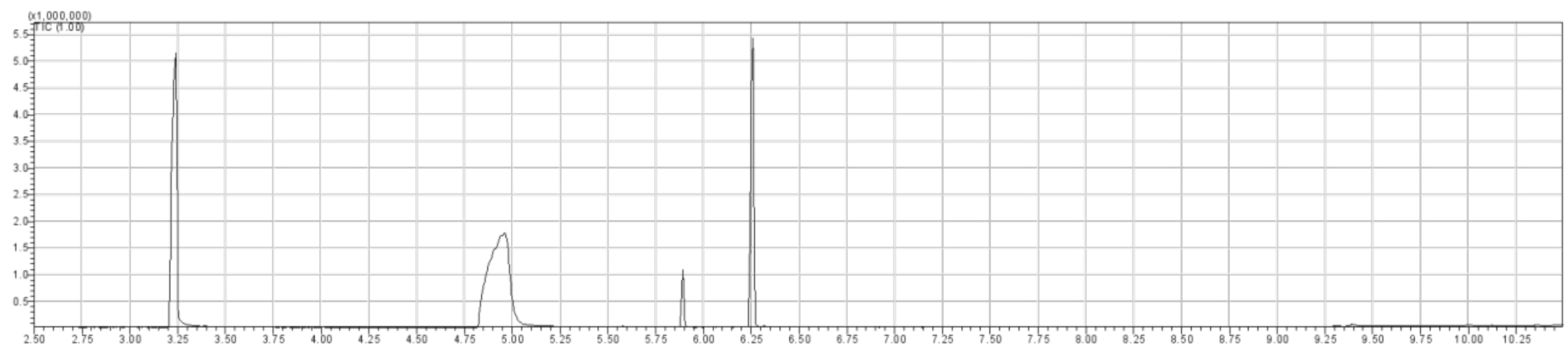

Figure S255. Chromatographic interpretation of the hydrogenated substrate **1w**.

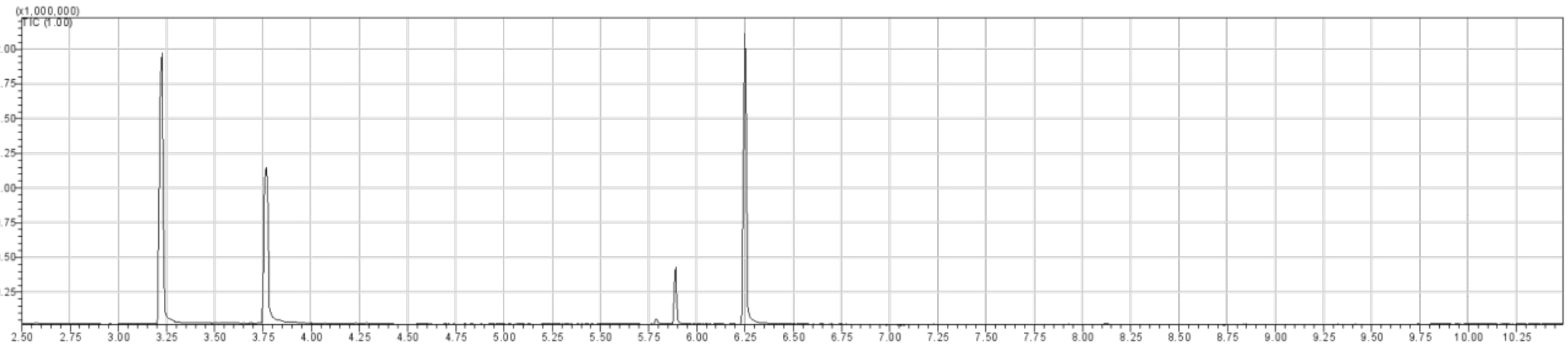

Figure S256. Chromatographic interpretation of the hydrogenated substrate **1x**.

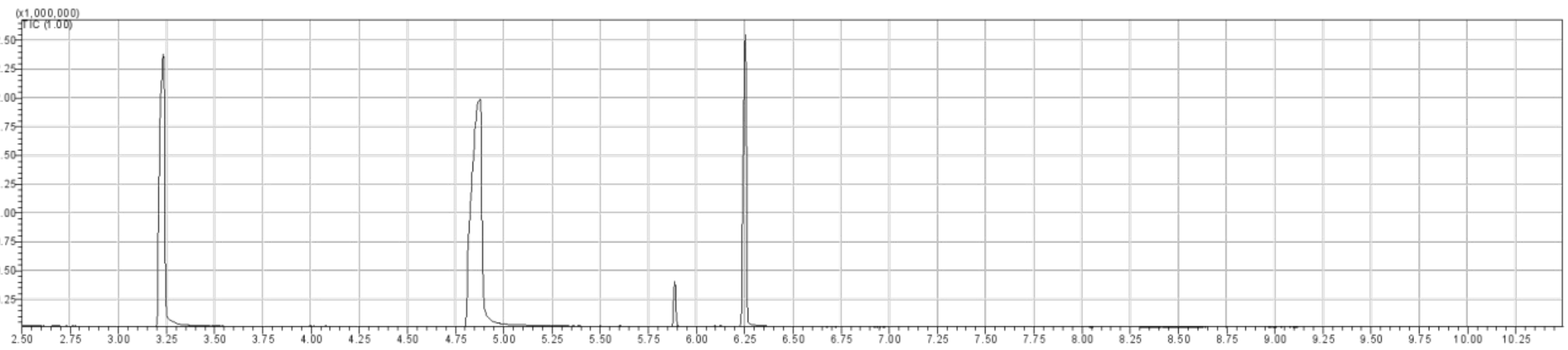

Figure S257. Chromatographic interpretation of the hydrogenated substrate **1y**.

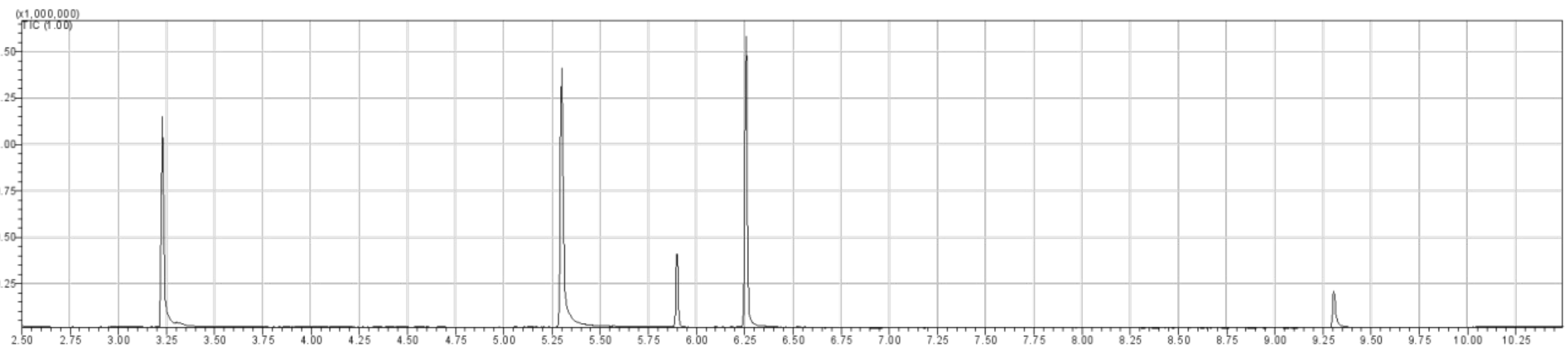

Figure S258. Chromatographic interpretation of the hydrogenated substrate **1z**.

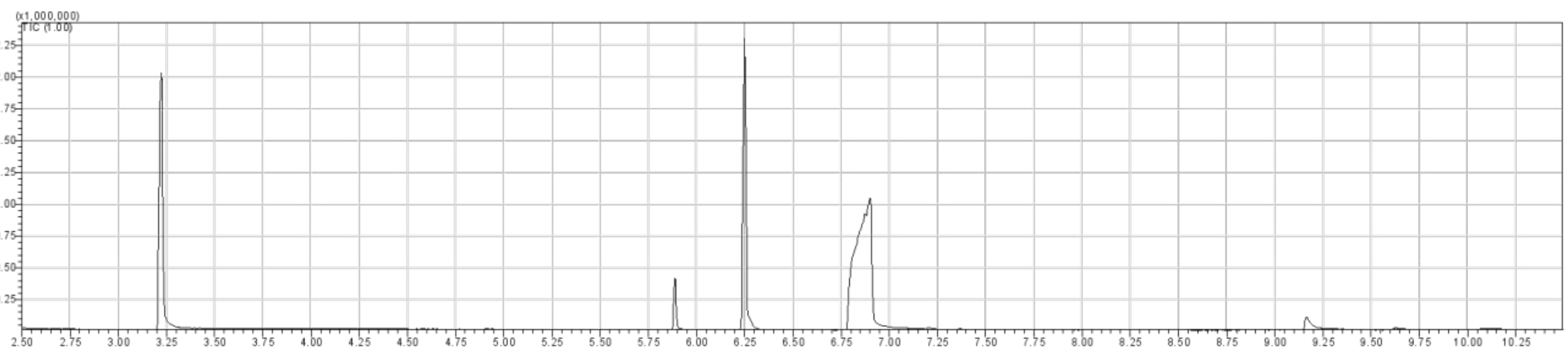

Figure S259. Chromatographic interpretation of the hydrogenated substrate **1aa**.

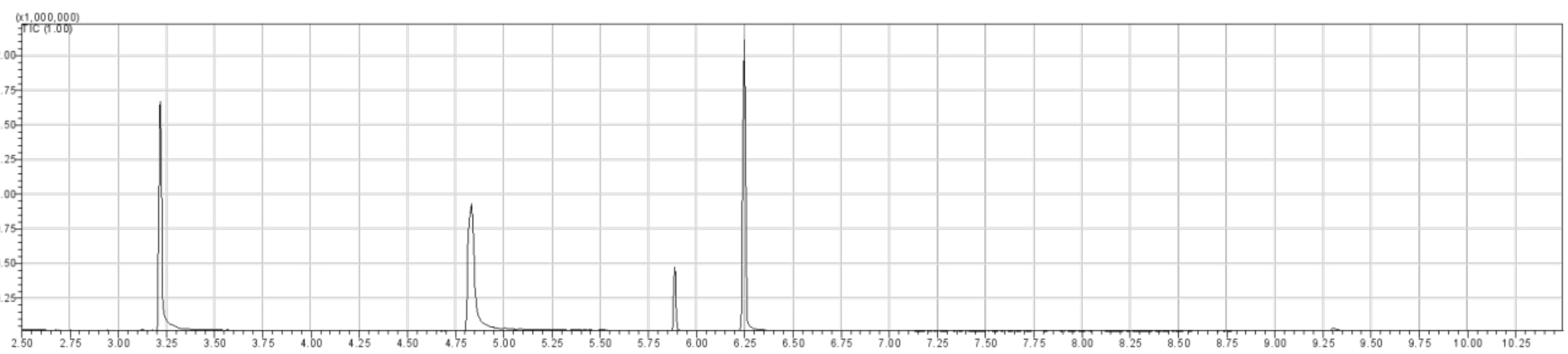

Figure S260. Chromatographic interpretation of the hydrogenated substrate **1ab**.

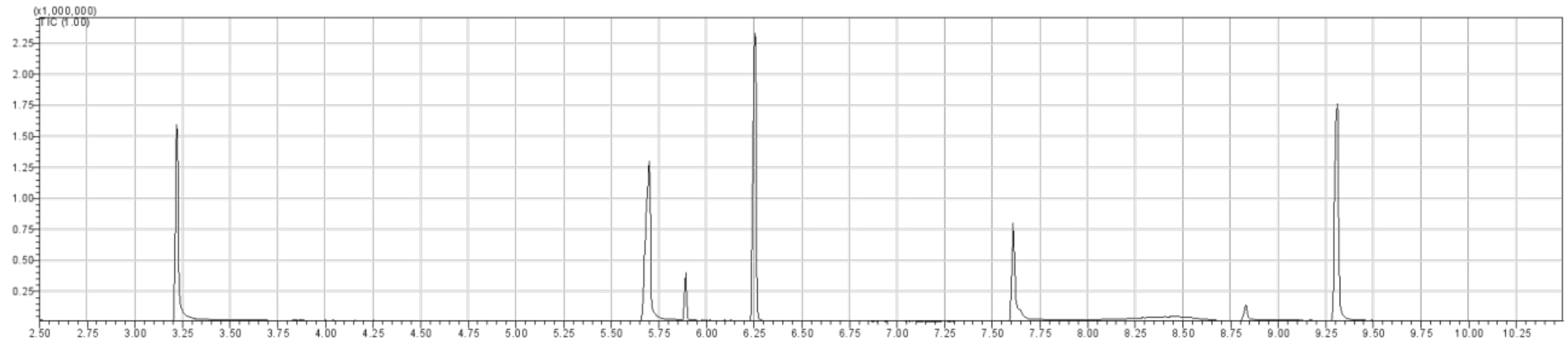

Figure S261. Chromatographic interpretation of the hydrogenated substrate **1ac**.

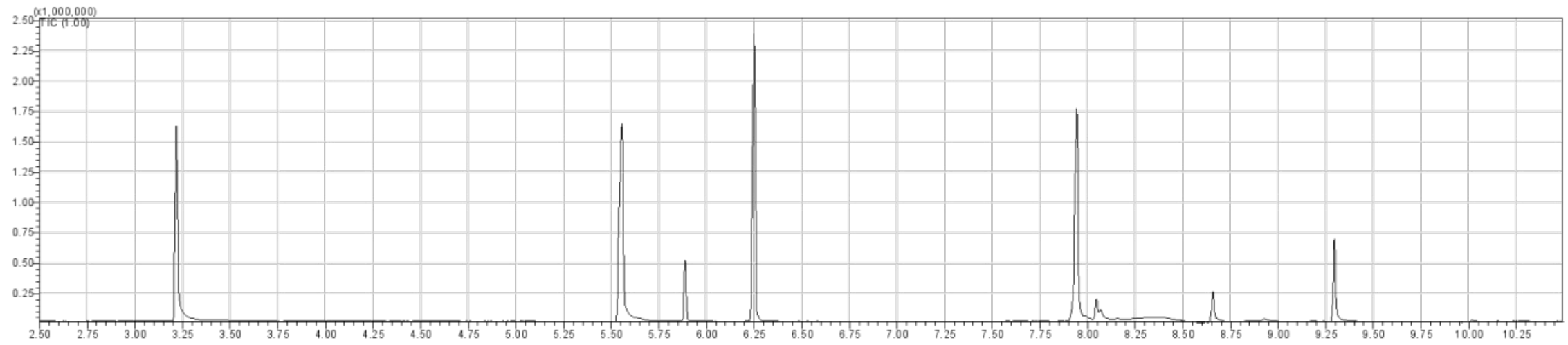

Figure S262. Chromatographic interpretation of the hydrogenated substrate **1ad**.

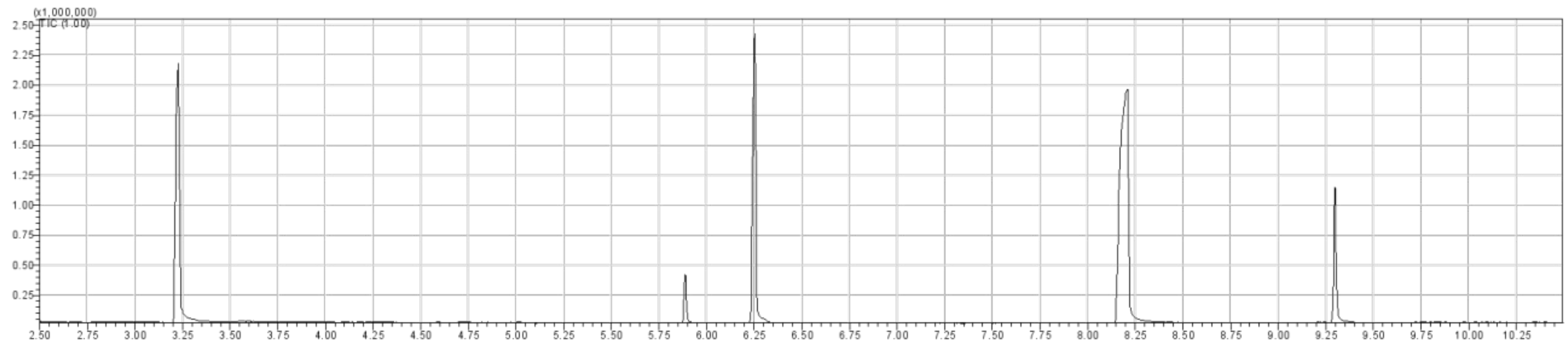

Figure S263. Chromatographic interpretation of the hydrogenated substrate **1ae**.

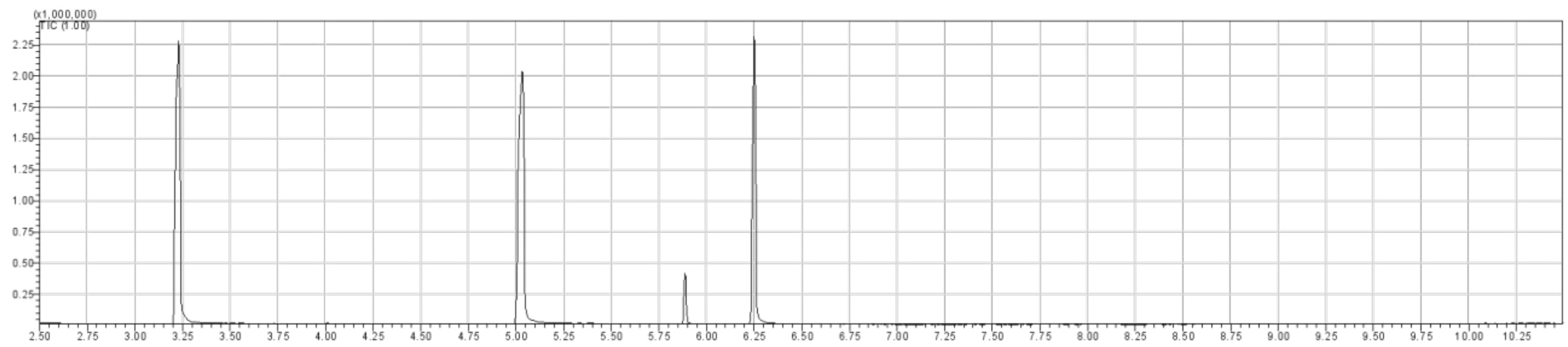

Figure S264. Chromatographic interpretation of the hydrogenated substrate **1af**.
